
JOURNAL INFORMATION
==============================
NLM Title Abbreviation: J Physiol Sci
Journal ID: J Physiol Sci
NLM Journal ID: 101262417

The Journal of Physiological Sciences : JPS

ISSN: 1880-6546
EISSN: 1880-6562
Publisher: Elsevier

ARTICLE INFORMATION
==============================
PMCID: PMC10717016
DOI: 10.1186/s12576-022-00851-3
Article ID: 851
Article version: 1
Subjects: Meeting Abstracts

Proceedings of the 99th Annual Meeting of The Physiological Society of Japan

Electronic publication date: 2022 Dec 23
Publication date: 2022
Volume: 72
Issue: Suppl 1
Issue sponsor: Publication costs for this supplement were funded by The Physiological Society of Japan.
Electronic Location ID: 33
Copyright: © The Author(s) 2022
License: Open AccessThis article is licensed under a Creative Commons Attribution 4.0 International License, which permits use, sharing, adaptation, distribution and reproduction in any medium or format, as long as you give appropriate credit to the original author(s) and the source, provide a link to the Creative Commons licence, and indicate if changes were made. The images or other third party material in this article are included in the article's Creative Commons licence, unless indicated otherwise in a credit line to the material. If material is not included in the article's Creative Commons licence and your intended use is not permitted by statutory regulation or exceeds the permitted use, you will need to obtain permission directly from the copyright holder. To view a copy of this licence, visit http://creativecommons.org/licenses/by/4.0/.
License URL: https://creativecommons.org/licenses/by/4.0/

Conference name: The 99th Annual Meeting of The Physiological Society of Japan
Conference location: Sendai, Japan
Conference date: 16-18 March 2022
issue-copyright-statement: © The Author(s) 2022

==============================

### Special Lectures
### Memorial Lectures

*J Physiol Sci* (2022) **72** (Suppl. 1):33
https://doi.org/10.1186/s12576-022-00851-3
Published: 23 December 2022

### Special Lecture 1

March 16(Wed), 11:15 - 12:15, Room A

### [SP01-01]

### Molecular mechanisms regulating body fluid ion environments and their physiological and pathophysiological meanings

*Yoshinori Marunaka[1,2,3]
*([1]Kyoto Industrial Health Association, [2]Ritsumeikan University, [3]Kyoto Prefectural University of Medicine)*

The ion environment plays key roles in regulation of our body functions. Specially, $H^+$ is one of the most important ions regulating our body functions via control of enzyme activities and protein-protein binding affinities. The pH in our body is controlled by the lung and the kidney: The lung excretes $CO_2$, one of the major acid sources, into the atmosphere, and the kidney excretes nonvolatile acids such as sulfuric acid, nitric acid and phosphoric acid into urine. The blood pH is controlled by strong pH buffers such as hemoglobin and albumin, while the interstitial fluid has relatively weak pH buffers (e.g., the $CO_2$-$HCO_3$ - buffer system) compared with hemoglobin and albumin. I have found that the interstitial fluid pH is lower in type 2 diabetes mellitus (DM) model OLETF rats than that in non-DM rats. In this special lecture, I will show the pathophysiological meaning of the lowered pH of interstitial fluids regarding insulin resistance in DM and the therapeutic method to improve the insulin resistance by elevating the lowered pH in DM. Supported by Grants-in-Aid for Scientific Research (B) from Japan Society of the Promotion of Science (JSPS KAKENHI JP18H03182 and JP21H03368 to Y.M.).

### Special Lecture 2

March 16(Wed), 14:30 - 15:30, Room A

### [SP02-01]

### Pysiological significance of the measurement of intracellular $Ca^{2+}$ concentration in muscle

*Satoshi Kurihara[1]
*([1]Department of Cell Physiology, The Jikei University School of Medicine)*

Intracellular $Ca^{2+}$ plays a pivotal role in excitation-contraction coupling in muscle. The measurement of the intracellular $Ca^{2+}$ concentration is essential for understanding the mechanism of excitation-contraction coupling. In the present lecture, the importance of measuring the intracellular $Ca^{2+}$ concentration with a $Ca^{2+}$-sensitive photoprotein, aequorin, will be presented and discussed. In mammalian cardiac muscle, intracellular $Ca^{2+}$ rapidly increases following an electrical stimulation and then exponentially decreases; this event is referred to as a $Ca^{2+}$ transient. Following a $Ca^{2+}$ transient, tension starts to increase and then decreases. When muscle is stretched from a slack length to a longer length, peak tension increases without a change in the peak of $Ca^{2+}$ transient, but the $Ca^{2+}$ transient decays more rapidly. Thus, the increase in twitch tension cannot be explained by the increase in the peak of a $Ca^{2+}$ transient, and the increased $Ca^{2+}$ binding to the contractile protein (a change in the $Ca^{2+}$ sensitivity) is considered to be the mechanism of the increase of twitch tension at a longer length. We measured $Ca^{2+}$ transients and tension under various conditions (in acidosis and, in the presence of autonomic neurotransmitters and inotropic agents). We discuss the intracellular mechanisms of these factors in relation to intracellular $Ca^{2+}$.

### Special Lecture 3

March 17(thu), 9:00 - 10:00, Room A

### [SP03-01]

### Second messenger and circulatory regulation

*Yoshihiro Ishikawa[1]
*([1]Yokohama City University School of Medicine)*

Cyclic AMP is the classic second messenger that regulates the intracellular signal. Upon activation of cell surface receptors by bioactive ligands, G proteins are activated. The production of various second messengers, including cAMP, is initiated by effector enzyme, leading to the activation of various kinases or further downstream molecules to trigger cellular responses. Thereby, many bioactive ligands, ranging from catecholamine to prostaglandin, can modulate such second messenger-mediated organ function. Their outcome varies among tissues, patho- or physiological status, or developmental stages. Under the physiological condition, cardiac function is activated by the sympathetic nerve system, leading to chronotropic and contractile changes. Parasympathetic nerve activation can reverse such changes. The dual regulation of the heart by sympathetic and parasympathetic nervous system can be explained, at least partially, by the presence of cardiac adenylyl cyclase isoform. Circulatory regulation by bioactive ligands also occurs during development. Upon birth, a rapid circulatory change, i.e., from fetal to adult circulation, occurs. Bioactive ligand, in particular prostaglandin from placenta, plays a major role in this adaptation process via its vasodilatory and, more importantly, intimal thickening effect.

### Special Lecture 4

March 17(thu), 15:15 - 16:15, Room A

### [SP04-01]

### Roles of excitatory and inhibitory neurotransmitters in learning

*Takeo Watanabe[1,2,3,4,5]
*([1]Brown University Department of Neuroscience, [2]Brown University Department of CLPS, [3]Brown University Medical School Department of Psyschiatry, [4]Advanced Communication Research Institute, [5]University of Paris)*

In this talk, I will discuss mechanisms of post-encoding stages of learning. Although mechanisms of encoding of learning have been investigated to a great degree, those in post-encoding stages (e.g., stabilization, consolidation, reactivation) have been less clarified. We have investigated how plasticity and stability occur in post-encoding stages, using paradigms developed in studies of visual perceptual learning (VPL; Watanabe, Nanez &Sasaki, *Nature*, 2001; Watanabe et al., *Nature. Neuroscience*, 2002; Seitz &Watanabe, 2003, *Nature* ; Tsushima, Sasaki & Watanabe, 2006, *Science*; Shibata et al, 2011, *Science*). By means of magnetic resonance spectroscopy, we obtained an E/I ratio, which refers to the concentration of glutamate and glutamine for excitation, divided by the concentration of GABA for inhibition in human visual areas at different postencoding stages of VPL. E/I ratios and. performance indicate that plasticity and stability occur in significantly different manners in different post-training stages (Shibata et al, *Nature Neuroscience*, 2017; Bang et al, *Nature Human Behavior*, 2018; Tamaki et al, *Nature Neuroscience*, 2020).

### Special Lecture 5

March 17(Thu), 16:30- 17:30, Room A

### [SP05-01]

### Physiological studies on ion channels involved in cell death induction - Hitherto and hereafter -

*Yasunobu Okada[1,2,3,4]
*([1]National Institute for Physiological Sciences, [2]Department of Physiology, School of Medicine, Aichi Medical University, [3]Department of Physiology, Kyoto Prefectural University of Medicine, [4]Cardiovascular Research Institute, Yokohama City University)*

The plasma membrane in animal cells is highly permeable to water. The extracellular and intracellular osmolarity is prone to change under physiological and pathological conditions. Thus, animal cell must cope with osmotic cell swelling and shrinkage, in a regulated manner, by losing intracellular ions such as $K^+$ and $Cl^-$ and by gaining extracellular ions especially $Na^+$ and $Cl^-$, thereby driving water efflux and influx, respectively. These volume-regulatory ion transport pathways are mainly provided by a number of types of ion channels ubiquitously expressed in animal cells. Control of the cell volume is essential for the survival of animal cells. Thus, the research on the mechanisms of cell volume regulation (CVR) inevitably leads to the research on the mechanisms of cell death induction. Here, following topics are to be touched on: how the research works on ion channels involved in the CVR mechanisms have hitherto been developed, and what is to be hereafter elucidated, with noting, in an episodic manner, how and why a physiologist came across the research subject of CVR and ended up that of cell death induction associated with pathological situations.

### Special Lecture 6

March 18(Fri), 11:00 - 12:00, Room A

### [SP06-01]

### My research in physiology ～ Search from the comparative analysis of "normal/abnormal" using cerebellar mutant mice ～ The discovery of ER-resident $IP_3$ receptor/channel and establishing a new paradigm

*Katsuhiko Mikoshiba[1]
*([1]SIAIS ShanghaiTech University)*

In order to understand the brain development and brain function, I performed a comparative analysis of "normal/abnormal" using cerebellar mutant mice. We discovered that 1) $P_{400}$ protein decreased in the Purkinje neuron-deficient ataxic mutant is the $IP_3R$ and that it is 2) a $Ca^{2+}$ channel and is 3) localized on the endoplasmic reticulum (ER) which is the place for protein synthesis and protein quality control. ER-resident $IP_3R$ channel regulates variety of cell functions such as development and behavior. $IP_3R1$ KO mice show abnormal neural plasticity: LTD in the cerebellum is blocked. LTP suppression and Depotentiation are attenuated in the hippocampus. Human $IP_3R1$ mutation causes Spinocerebellar ataxia 15 (SCA15) and 29 (SCA29). $IP_3R2$ & 3 KO causes exocrine secretion deficit and human $IP_3R2$ mutation exhibits sweat secretion deficit, suggesting $IP_3R2/3$ channel is surely involved in exocrine secretion. $IP_3R2$ KO shows blockage of taste perception. The $IP_3R$ channel offers a huge platform for forming a protein complex, suggesting a new concept of channel which carries a huge "signaling hub". In case of emergency such as ER stress, $IP_3R$ channel binds to chaperone GRP78, ERp44 and Bcl-2 apoptotic family members and protect neural cells from neurodegeneration.

### Memorial Lecture 1

S.Hagiwara Memorial Lecture

March 17(Thu), 10:45 - 11:30, Room A

### [ML01-01]

### A short review on fMRI from its genesis to current and future

*Seiji Ogawa[1]
*([1]Tohoku Fukushi University)*

In late 1980's, we observed MRI signal changes that were sensitive to the blood oxygenation in the mouse brain. We postulated the possibility that Blood Oxygenation Level Dependent (BOLD) phenomenon seen in MRI would give us a non-invasive way to visualize localized functional activity of the human brain. The dream became true in 1992. Nature provided us the neuro-vascular coupling process so that the onset of synaptic activity and neuronal firing in a group of neuronal assembly induces vascular changes (diameter of arterioles) and then BOLD signal. Although such functionally responding BOLD signal has many underlying factors influential to the degree of the MRI signal change, fMRI with given tasks has been used to find many functionally specific sites in the brain and contributed to promoting the understanding of how the human brain works. Accumulation of details of functional sites throughout the brain , such as their locations, functional characters, and their connections to other sites, enables to apply AI scheme to the brain. On-going clinical application of fMRI, however, is limited so far to pre-surgical examination of functional areas around the tumor targeted for removal. As a different approach, so-called resting state (rest-) fMRI, run without given tasks, has become a major player by showing the possibility of finding macroscopic functional networks. They are based on the functional connectivity that is indicated by the signal correlation of time series data at various sites. From such data, many functional networks analogous to those seen in task-fMRI have been shown. The brain seems to perform some organized functional activities spontaneously without any loaded tasks. Although the reason for this activity in the brain is still unknown and some controversies exist, the research activity in rds.-fMRI spread to wide area in brain science even to diagnosis of CNS diseases. In spite of some controversies and difficult problems, the acquisition method and data analysis of fMRI with better understanding of the signal characters will be improved to advance fMRI application. As an example, applying "yuragi" principle to the analysis of brain data seems to have big advantage over usual AI approach by more than an order of magnitude less sample size requirement. Back to the basics in fMRI data acquisition and analysis, there are some interesting and attractive topics to explore, like characteristics of the basal state signal which may carry the influence of the physiological environment mentioned earlier. Another topic is to find the information content of functional activation at a site, so that one can tell what the site has done by its processing activity. There may be some slight chance to see this at the output layer at the bottom of cortex.

### Memorial Lecture 2

S.Tawara Memorial Lecture

March 17(Thu), 11:30 - 12:15, Room A

### [ML02-01]

### Malfunction and dysregulation of cardiac ion channels as substrates for arrhythmogenicity

*Katsushige Ono[1]
*([1]Oita University)*

Professor Tawara is known for the discovery of the atrioventricular excitation conduction system in the heart. Tawara's groundbreaking work on the conduction system was the basis for the discovery of the sinus node and the interpretation of the modern heart's electrophysiology. In this Tawara Memorial Lecture, in relation to his monograph "The Conduction System of the Mammalian Heart", I will review recent understanding of cardiac electrical function and malfunction associated with ion channel regulation as a substrate of cardiac arrhythmias. The basic cellular unit of such activity is the action potential, which is shaped by specialized proteins, ion channels and transporters, that control the movement of ions across cardiac cell membranes in a highly orchestrated fashion. Cardiac disease modifies the operation of ion channels and transporters in a way that promotes the occurrence of cardiac rhythm disturbances, a process called arrhythmogenic changes. We consider key ionic changes that are associated with heart diseases, with the intention of identifying molecular mechanism for arrhythmias.

### Planned Symposia

### Joint Symposium 1

### [JO01]
### 【The Biophysical Society of Japan】
### Recent advances in biosensing research

March 16(Wed), 9:00 - 11:00, Room A

### [JO01-01]
**Cell thermal engineering: Measurement and control of subcellular temperature using functional dyes**

*Satoshi Arai[1] ([1]*Kanazawa University, Nano Life Science Institute (WPI-NanoLSI)*)

Temperature at intracellular microscale is of fundamental importance for cellular system. To unveil the system thermodynamically, the development of technology to measure and control subcellular temperature has been indispensable. Previously, using fluorescent dyes, we published success of organelle targetable fluorescent thermometers that allow the read-out of temperature at local organelles as a detectable fluorescent signal. Yet, targeted organelles were only limited to mitochondria and ER as well-known heat sources in thermogenic cells. Very recently, we extended this to several organelles including lipid droplets which have never been reached by the other sensing methods so far. We further demonstrated these thermometers in the subcellular thermometry of heat production in brown adipocytes using quantitative fluorescent lifetime imaging (FLIM). Other than that, the development of a means to control the intracellular temperature is also progressing using a functional photothermal dye. In this session, we will share recent progress on both sides of temperature control and thermometry in a live cell.

### [JO01-02]
**Highly sensitive biosensing technique using nanoscale quantum biosensors**

*Ryuji Igarashi[1] ([1]*National Institutes for Quantum Science and Technology*)

Fluorescence detection is an essential basic technology in sensitive clinical diagnostics. Biosensing technique using fluorescence, which is known to have the potential to detect a single molecule, is widely used to measure viruses and molecular biomarkers. However, the presence of "background light" such as autofluorescence, a light emission observed in positions containing no target molecules, may lead to false positives and conclusions. We recently developed an ultra-sensitive fluorescence detection technique that eliminates background light by using nano-sized fluorescent diamonds (fluorescent nanodiamonds) containing crystal lattice defects called nitrogen-vacancy centers (NV centers) as fluorescent reagents. In the technique, the fluorescence emitted by fluorescent nanodiamond is detected during quantum manipulation using microwave or laser pulses. Our method provides a more than 100-fold higher signal-to-background ratio compared to general fluorescence imaging. In recent years, such background-free biosensing techniques are attracting attention as "Quantum-Diagnostics Platform." Here, we describe the current methods and the future of the quantum-diagnostics platform.

### [JO01-03]
**In vivo cardiac nano-physiology: sarcomere synchronization and ventricular contractility**

*Fuyu Shimozawa Kobirumaki[1], Togo Shimozawa[2], Kotaro Oyama[3], Fukuda Norio[1] ([1]*The Jikei University School of Medicine, Department of Cell Physiology*, [2]*Technical Division, School of Scence, The University of Tokyo*, [3]*Quantum Beam Science Research Directorate, National Institute fo Quantum and Radiological Science and Technology*)

We analyzed dynamic behaviors of individual sarcomeres in a left ventricular (LV) myocyte of the in vivo beating mouse heart via expression of α-actinin-AcGFP in Z-disks. To quantify the contribution of sarcomeres to myofibrillar dynamics, we introduced "contribution index" (CI) to measure the synchrony in movements between a sarcomere and a myofibril [from -1 (full asynchrony) to 1 (full synchrony)]. First, CI varied markedly between sarcomeres, with an average value of 0.3 in normal systole. Second, when the movements between adjacent sarcomeres were asynchronous (CI<0), a sarcomere and the ones next to the adjacent sarcomeres and farther away moved in synchrony (CI>0) along a myofibril (i.e., distal inter-sarcomere interaction). Third, average CI was linearly decreased upon a decrease in LV developed pressure. Therefore, in the LV of the in vivo beating mouse heart, 1) sarcomeres heterogeneously contribute to myofibrillar dynamics due to an imbalance of active and passive force between neighboring sarcomeres, 2) the force imbalance is pronounced under depressed conditions, and 3) sarcomere synchrony via the distal inter-sarcomere interaction regulates the heart's pump function.

### [JO01-04]
**Visualizing ATP Dyanamics in Live Mice**

*Masamichi Yamamoto[1], Jungmi Choi[1] ([1]*National Cerebral and Cardiovascular Center Research Institute*)

ATP is directly used by all eukaryotes for various biological activities, including various enzymatic reactions and mechanical reactions such as muscle and cell migration. Therefore, ATP is called the "energy currency of the cell" common to all organisms. In recent years, with the development of metabolomic and mass spectrometric methods, we have made progress in understanding the state of energy metabolism and ATP metabolism within a certain population of cells and individuals, but the state and temporal changes at the level of a single cell within an individual are completely unknown. In order to elucidate this issue, we have constructed a system that can measure the behavior of energy metabolism and ATP metabolism at the single-cell level in the mouse body and throughout the body. In this article, I would like to introduce the performance of this mouse and the analysis that is currently underway.

### [JO01-05]
**Muscle diseases related to abnormal calcium homeostasis by ryanodine receptors**

*Toshiko Yamazawa[1] ([1]*The Jikei University School of Medicine*)

Type 1 ryanodine receptor (RyR1) plays a key role during excitation-contraction coupling of skeletal muscle. Mutations in RyR1 hyperactivate the channel to cause malignant hyperthermia (MH). MH is a serious complication characterized by skeletal muscle rigidity and elevated body temperature in response to commonly used inhalational anesthetics. Some heat stroke triggered by exercise or environmental heat stress is also related to MH mutations in the RyR1 gene. The only drug approved for ameliorating the symptoms of MH is dantrolene. Here we show that a novel RyR1-selective inhibitor, Compound 1 (Cpd1), effectively rescues MH and heat stroke in new mouse model (*RYR1*-p.R2509C) relevant to MH. Cpd1 has great advantages of higher water solubility and shorter plasma half-life compared to dantrolene. Our data suggest that Cpd1 has the potential to be a promising new candidate for effective treatment of patients carrying RyR1 mutations. Finally, we have recently identified that heat directly activates RyR1, which induce $Ca^{2+}$ release from intracellular stores. Our results provide direct evidence that heat induces $Ca^{2+}$ release (HICR) from the SR through the mutants rather than wild-type RyR1, causing an immediate rise in the cytosolic $Ca^{2+}$ concentration.

### Joint Symposium 2

### [JO02]
### 【The Japan Society of Acupuncture and Moxibustion】
### Scientific basis of oriental medicine

March 16(Wed), 9:00 - 11:00, Room C

### [JO02-01]
### Distinctive regulatory system of blood flow in the orofacial area mediated by autonomic neural and humoral system

*Hisayoshi Ishii[1], Toshiya Sato[1] (*[1]Division of Physiology, Department of Oral Biology, School of Dentistry, Health Sciences University of Hokkaido*)

Blood flow (BF) is important in the maintenance of orofacial functions. Disturbance of BF is related to various dysfunctions in the orofacial tissues, such as epithelial tissues, muscles, and salivary glands. Marked BF changes mediated by the autonomic nervous system may be of importance in orofacial hemodynamics. Studies have shown that BF in the orofacial area is regulated by the neural system (the parasympathetic and sympathetic nerves) and the humoral system, especially the sympathoadrenal system. Parasympathetic vasodilator fibers have been reported to originate from the pterygopalatine, otic and internal carotid ganglia. Sympathetic vasoconstrictor fibers arise in the superior cervical and stellate ganglia. It has also been reported that the release of circulating adrenaline is involved in a β-adrenergic vasodilation in the masseter muscle. This presentation focuses on: (i) the distinctive regulatory system of BF in the orofacial area, mediated by the autonomic neural and humoral systems; (ii) their interactions, and; (iii) the involvement of autonomic vasomotor responses in the physiological role of the orofacial functions, and in the etiology of orofacial dysfunctions related to disturbances of the autonomic nervous system. The authors declare that they have no conflicts of interest, financial or otherwise, regarding this article.

### [JO02-02]
### Effects of acupuncture treatment using thermograph on the sequelae of facial paralysis

*Mari Nakamura[1] (*[1]Mari Acupuncture Clinic*)

Purpose: As an aid to the mechanism of the effect on the sequelae of facial paralysis, we measured the facial area with a thermograph before and after one acupuncture treatment combined with moxibustion, and examined changes in skin temperature. Method: The subjects were seven patients (average: 36.6 years old) diagnosed with facial paralysis. Acupuncture was applied to improve the overall condition of patients by therapy against the symptom as fundamental treatment and needles were inserted at ten acupuncture points on the face including the non-paralyzed side as local treatment. Also, indirect moxibustion was applied to two points on the paralyzed side. A thermograph was used to measure the average skin temperature on the forehead and both cheeks. Results: There was no significant difference in skin temperature between the forehead and cheek areas before and after treatment. The skin temperature of each area after treatment was higher on the paralyzed side, but the effect size ($d$) was 'small' at 0.2. Only the buccal skin temperature on the paralyzed side showed an upward trend after treatment ($P$ = 0.09). Conclusion: In this study, due to the requirements of the subjects and the features of the treatment, there was no significant difference in both the skin temperature between both sides and the increase in skin temperature on the paralyzed side due to acupuncture treatment.

### [JO02-03]
### Effects of acupuncture stimulation on the cardiovascular responses.

*Hidehiro Nakahara[1], Tadayoshi Miyamoto[2] (*[1]Morinomiya University of Medical Sciences, [2]Osaka Sangyo University*)

The purpose of this study was to examine whether the manual acupuncture (MA) stimulation of different acupuncture points evokes different responses by the heart and vasculature. Sixty healthy subjects were randomly divided into a control group and MA stimulation groups at the lower leg, ear, abdomen, and forearm. MA was performed at 1 Hz for 2 min. A depressor response was observed only in the lower leg stimulation group, in which mean blood pressure significantly decreased ($p < 0.05$). A bradycardic response was elicited in all MA stimulation groups. There was no significant differences in the magnitude of the bradycardic response between groups. These results provide fundamental evidence that bradycardic and depressor responses are effectively produced by acupuncture in humans.

### [JO02-04]
### Anti-stress effect of acupuncture treatment -evaluating using stress model animals-

*Masataka Sunagawa[1], Mana Tsukada[1], Aki Fujiwara[1], Oyunchimeg Chuluunbat[1], Tadashi Hisamitsu[1] (*[1]Department of physiology, school of medicine, Showa university*)

Orexin is a neuropeptide secreted in the hypothalamus and involved in stress response induction. We investigated the effect of acupuncture on stress responses and the involvement of orexin secretion regulation as a mechanism of action using chronic or acute stress model rats.

Press tack needle (PTN) were used for acupuncture treatment. A social isolation stress model was used as a chronic stress model. Seven-day stress loading increased aggression, corticosterone and orexin secretion. However, PTN treatment at acupoint GV20 suppressed the increase in orexin secretion.

As acute stress models, a restraint stress model and an acute pain stress model were used. The secretion of orexin was increased by PTN treatment, unlike the chronic stress model.

The stress reaction is a physiological function acquired to adapt to emergency situations. Acupuncture treatment was considered to have promoted the reaction during acute stress and suppressed the unnecessary stress reaction during chronic stress. Acupuncture is thought to have controlled the stress response through the regulation of orexin secretion.

### [JO02-05]
### Clinical research on massage therapy: Effectiveness and biological mechanisms

*Nozomi Donoyama[1] (*[1]Department of Health, Faculty of Health Sciences, Tsukuba University of Technology*)

The number of academic articles on massage therapy has increased since the mid-1990s. According to review articles published by Dr. Field of the Touch Research Institute, massage therapy has beneficial effects on prenatal depression, pre- and full-term infants, enhanced attentiveness, autism, skin conditions, pain syndromes, hypertension, immune and autoimmune conditions, and aging problems. Studies have shown that massage therapy can increase vagal activity, increase numbers of natural killer cells and their activity, decrease cortisol levels, reduce heart rate, and alter electroencephalogram patterns, as part of a relaxation response. As for the underlying neurophysiological and biochemical mechanisms of these therapeutic effects, tactile and pressure stimulation induces stretch and somato-visceral reflexes, which regulate muscles and adjust the conditions of internal organs, respectively, and causes autonomic nervous responses through the thalamus and hypothalamus and affects the endocrine system via the pituitary-adrenal cortex system. Tactile and pressure stimulation might also release endogenous opioids and facilitate their participation in the descending inhibitory pathways. The author declares that there is no conflict of interest.

### Joint Symposium 3

### [JO03]
### 【Anatomical Science International】
### Integrated approach for age-dependent changes in brain structure and function: From embryonic and postnatal development to ageing

March 16(Wed), 15:45- 17:45, Room C

### [JO03-01]
### Neural development and programmed senescence in forebrain

*Kyoji Ohyama[1] ([1]*Department of Histology and Neuroanatomy, Tokyo Medical University*)

In this symposium, I present some of our work on hypothalamic development in chick and programmed senescence at hippocampal fissure in developing mice.
I firstly present some data, including scRNA-seq datasets, as to how hypothalamus is specified, regionalized, and then how hypothalamic neurons differentiate during development. I will discuss about the roles of signaling molecules and their downstream transcription factors. Then, I move to the second part where I introduce emerging concept of programmed senescence in development. Growing bodies of evidence show that programmed senescence occurs in signaling centres such as apical ectodermal ridge and hindbrain roof plate. However, it remains unclear whether programmed senescence takes place in developing forebrain. Here I show some evidence that programmed senescence also occurs in the forebrain, namely at hippocampal fissure, a signaling centre for hippocampal dentate gyrus development. I will discuss about its potential significance in neural development.

### [JO03-02]
### Regulation of the molecular basis of stem cell aging and its impact on organismal homeostasis

*Hayato Kaneda[1] ([1]*Shiga Univ. Med. Sci.*)

Stem cell aging reduces regenerative capacities and contributes to the disruption of tissue homeostasis. The age-related dysfunctions of various tissue stem cells and its molecular mechanisms have been studied. However, the common molecular basis of the stem cell aging remains largely unexplored. Here we present the results of our search for common molecules involved in the aging of different tissue stem cells and the effects of their regulation at the tissue and organismal levels. We isolated mesenchymal stem/stromal cells (MSCs) and hematopoietic stem/progenitor cells (HSCs) from young (2-3 months old) and old (>24 months old) mice and examined gene expression profiles comprehensively. As a result, we found that DNA damage response (DDR) is impaired in aged stem cells by a common molecular mechanism. Loss of function of the molecule induced DDR impairments in young stem cells both *in vitro* and *in vivo*, similar to those in senescent stem cells. Moreover, the precocious dysregulation of DDR in intestinal stem cells remotely induced inflammation in the brain.

### [JO03-03]
### Influence of cerebral cholinergic system modulation and amyloid β deposition on cerebrovascular response to transient brain ischemia

*Nobuhiro Watanabe[1], Kaori Iimura[1], Harumi Hotta[1] ([1]*Department of Autonomic Neuroscience, Tokyo Metropolitan Institute of Gerontology*)

Alzheimer's disease (AD) is thought to be triggered by abnormal deposition of amyloid β (Aβ) in the brain parenchyma. Recently, cerebrovascular dysfunction such as cerebral ischemia is also considered as a risk factor of AD. Pathological traits in AD related to vasculature include; (i) a loss of basal forebrain cholinergic (vasodilating) neurons, and (ii) Aβ deposition around cerebral arteries. In this talk, we will introduce our recent studies on (1) the role of the cholinergic system in cerebral blood flow (CBF) control during ischemiareperfusion, and (2) the influence of Aβ deposition on pial artery responses. Cerebral ischemia was induced by transient occlusion of unilateral common carotid artery in anesthetized mice. To measure CBF, laser-speckle contrast imaging was used. CBF decreased in the ipsilateral cortex during occlusion and increased after reperfusion. The CBF increase after reperfusion was attenuated by intracisternal administration of a cholinergic blocker. Conversely, a Kampo medicine with cholinergic stimulating effect enhanced the CBF increase. In a separate experiment, cerebrovasculature and Aβ deposition were imaged using two-photon microscopy. Pial arteries dilated during occlusion and reverted after reperfusion. In AD model mice, the vasodilation at Aβ deposition site was diminished. These results suggest that attenuation of cholinergic function and Aβ deposition around cerebrovasculature may be involved in vulnerability to cerebral ischemia in AD.
This study was partly funded by Tsumura Co.

### [JO03-04]
### Involvement of ROS in aging and plasticity of cerebellar synapses

*Sho Kakizawa[1] ([1]*Dept. Biol. Chem., Grad. Sch. Pharmaceu. Sci., Kyoto University*)

Reactive oxygen species (ROS) is a redox-signaling molecules, and indicated to be involved in various pathophysiological events in organisms, such as aging and lifestylerelated diseases. In addition, recent studies suggest involvement of ROS in physiological events. For example, expression of ROS synthases (NADPH oxidase (Nox) and dual oxidase (Duox)) in various tissues are reported. These facts indicate that ROS has dual functions, physiological and pathophysiological functions, in organisms. However, molecular mechanisms of ROS-related aging and physiological functions of endogenous ROS are yet to be determined, especially in brain systems. Plasticity at cerebellar synapses are cellular basis for cerebellar-dependent motor learning, and both long-term depression (LTD) and long-term potentiation (LTP) are identified at parallel fiber (PF) -to- Purkinje cell synapse in the cerebellum. In this symposium, I will introduce our recent studies indicating that involvements of ROS-related signaling in inhibition of PF-LTP in aged cerebellum and induction of PF-LTD at young-adult cerebellum. Possible molecular mechanisms are also proposed. Taken together, these observations suggest dual functions of ROS in physiological and pathophysiological events in brain systems.

### Joint Symposium 4

### [JO04]
### 【Belgium & Japan Joint Symposium】
### Hierarchical control of locomotion

March 16(Wed), 15:45 - 17:45, Room D

### [JO04-01]
**Flexible neurotransmitter phenotype of defined excitatory interneurons determines the ability to walk after spinal cord injury**

*Aya Takeoka[1,2] ([1]*NeuroElectronics Research Flanders/VIB,* [2]*KU Leuven, Department of Neuroscience, Leuben Brain Institute*)

Complete spinal cord injury to the mature nervous system leads to irreversible paralysis below lesion. However, adult rodents receiving a complete thoracic lesion just after birth demonstrate proficient hindlimb locomotion without input from the brain. How the spinal cord achieves such striking functionality remains unknown. In this study, we uncover age of injury-dependent divergent synaptic connectivity from interneurons to motor neurons. Adult injury prompts neurotransmitter phenotype switching of genetically specific excitatory interneurons to promote inhibition. In contrast, neonatal injury causes synaptic sprouting of the identical excitatory populations to motor neurons. Ablation of proprioceptive afferents that otherwise densely innervate these subsets of excitatory interneurons leads to neurotransmitter phenotype switch, and loss of glutamatergic phenotype after neonatal injury. Furthermore, genetically forcing neurotransmitter switch from excitation to inhibition abrogates autonomous locomotor functionality. Together, our study demonstrates that glutamatergic neurotransmitter phenotype maintained by proprioceptive afferents shapes spinal circuits to walk without the brain after neonatal injury.

### [JO04-02]
**Plastic change of spinal locomotor circuits in humans**

*Yukio Nishimura[1] ([1]*Tokyo Metropolitan Institute of Medical Science*)

Motor cortex shows drastic reorganization of somatotopic mapping after brain damage and repetitive training. However, it is elusive that such plastic change in the functional mapping occurs at spinal level. Here we show the existence of functional mappings in the spinal cord which depends on locomotion patterns and its flexible change. Non-invasive magnetic stimulation on human lumbar vertebra at the level of L1-L3 induces alternative left-right walking-like behavior in legs, while stimulation at L3-L5 induces hopping behavior. Such functional mapping for locomotion rapidly changes. The spinal site that was inducing walking changed to induce hopping after volitional hopping for a few minutes without stimulation. Furthermore, repetitive stimulation over the lumbar cord enables individuals with spinal cord injury to control gait behavior. In addition to the stimulationinduced gait improvement, long-term intervention improved the stimulation-induced gait and facilitated functional recovery of volitional gait control without stimulation. Thus, the functional module for locomotion in human lumber cord is reorganized and it is associated with changes in locomotion patterns.

### [JO04-03]
**Cortical control of bipedal gait in primates: primary motor cortex (M1) of Japanese monkeys**

*Katsumi Nakajima[1], Marc Maier[2], Takashi Suzuki[1], Kei Mochizuki[1], Kazunori Morita[1], Yoshiro Suzuki[1], Masahiko Inase[3] ([1]*Iwate Medical University,* [2]*Universite de Paris,* [3]*Kindai University*)

Japanese monkeys are quadrupedal (QP) animals that are considered facultative bipeds. To investigate how cortical networks deal with instability of upright posture during bipedal (BP) gait, we recorded the activity of 165 cells from hindlimb/trunk regions of M1 in two monkeys walking on a treadmill. Concurrent EMG activity was also recorded. Most M1 cells modulated their activity in a phasic or phasic/tonic manner for both QP and BP gait. During BP (vs. QP) gait, M1 population activity showed higher peak discharge frequency during the step cycle. The peak occurred predominantly during BP stance in 64% of cells (vs. 48%), particularly during double stance and around lift off (48 vs. 34%). A half of tested cells (n = 32/66) showed a significant correlation between their step-by-step peak firing rate and the concurrent peak activity of at least one hindlimb muscle for QP and/or BP gait. These results suggest that monkey M1 cells are significantly involved in generating step-by-step locomotor outputs via spinal circuits in a manner differently for QP and BP gait, and they contribute, particularly for BP, to secure the smooth transfer of the center of mass from one limb to the other.

### [JO04-04]
**Neural substrates for deciding timing of self-initiated locomotion**

*Masayoshi Murakami[1], Fanny Cazettes[2], Zachary Mainen[2], Kazuo Kitamura[1] ([1]*University of Yamanashi,* [2]*Champalimaud Research*)

Decision-making involves the selection of goals or actions, but it also requires determination of the timing of action. Action timing is especially important when there are no immediate sensory stimuli to trigger an action and timing must therefore rely on internal processes. How does the brain decide the timing of such self-initiated actions?
We set out to investigate this issue using a combination of behavior, pharmacology and electrophysiology in rats performing a waiting task in which a rat frequently gave up waiting for a delayed large reward and locomote toward a reward port for a smaller reward in a self-initiated manner. Multi-neuron recordings from the frontal motor cortex during this task identified neural activities that predicted the timing of self-initiated locomotion, including activities that ramped up during waiting and reached a threshold just before the action. The results reinforce the generality of ramp-to-threshold mechanism for decision-making.
We are currently investigating how the decision signals in the frontal cortex influence downstream locomotion circuits and eventually affect final choice using a head-restrained mouse model.

### Joint Symposium 5

### [JO05]
### 【The Japanese Medial Science Fedeation - committee for Science and Research】
### Frontier of interdisciplinary research on Frailty and Locomotive syndrome towards extending healthy life expectancy

March 17(Thu), 8:30 - 10:30, Room E

### [JO05-01]
### Development of an exercise program to prevent sarcopenia and locomotive syndrome

*Shuichi Machida[1] ([1]*Graduate School of Health and Sports Science, Juntendo University*)

Skeletal muscles, responsible for the motor functions of the body, decline with age. This age-related muscle atrophy—sarcopenia—is considered to be one of the causes of the decline in activities of daily living and quality of life. Locomotive syndrome (LS) is defined as "a state of reduced mobility due to disorders of the locomotor apparatus, including bones, muscles, and joints," and sarcopenia is a disorder of locomotion. As sarcopenia and LS progress, walking and balancing functions deteriorate due to disease and functional decline of the locomotor system. Moreover, assistance is required for daily activities such as indoor and outdoor mobility, going to the toilet, bathing, and dressing. In addition, the inability to move the body as desired can make going out seem like a chore, leading to a tendency to stay indoors which results in an increase in "prolonge d sedentary behavior." In this talk, I describe the "Sarcopenia and Locomotive Syndrome Prevention Exercise Program" that we have been developing and discuss the importance of exercise to prevent and improve sarcopenia and LS as an approach to prevent the necessity of nursing care for the older adult. The importance of exercise for the prevention and improvement of sarcopenia and LS as an approach to prevent the need for nursing care for the older adult will be discussed. In addition, examples of social implementation initiatives that we have implemented during the COVID-19 pandemic, such as the creation of DVDs and online exercise classes, will be discussed.

### [JO05-02]
### Mechanism of action of pharmacotherapy for osteoporosis

*Sakae Tanaka[1] ([1]*Sensory & Motor System Medicine, Graduate School of Medicine, The University of Tokyo*)

Osteoporosis is a serious health concern in the global community. Approximately 13 million osteoporosis patients are estimated to live in Japan, and the burden of osteoporosis with its associated morbidity and mortality issues due to fractures has become a critical socioeconomic problem. Skeletal integrity is maintained through a balance of bone resorption and bone formation. The bone turnover process, called bone remodeling, continues throughout life. Osteoclasts are primary cells for bone resorption, and osteoblasts regulate bone formation. The length of the resorption phase is very short compared with that of the formation phase, and the life span of osteoclasts is much shorter than that of osteoblasts. Therefore, increased bone remodeling necessarily leads to increased bone resorption and negative bone mass balance. Recently, a number of anti-osteoporosis drugs with excellent anti-fracture effects have been developed. They are mainly classified into two groups according to their effects on bone remodeling: anti-catabolic agents and anabolic agents. Anti-catabolic agents suppress bone resorption, and therefore reduce bone remodeling, while anabolic agents enhance bone remodeling by increasing bone formation more than bone resorption. In this talk, the molecular mechanisms of action of anti-osteoporosis drugs will be discussed.

### [JO05-03]
### Neural substrate linking motivation and motor outputs.

*Yukio Nishimura[1] ([1]*Neural Prosthetics Project, Department of Brain & Neurosciences, Tokyo Metropolitan Institute of Medical Science*)

The aging process implies physiological and psychological changes that hence affect the general health and mental states of elder individuals. Physical exercise is a renowned strategy that delays and alleviates the adverse consequences of the aging process. Here I show neural substrate bridging mind and motor outputs. Employing retrograde transneuronal labeling with rabies virus, we found the existence of multisynaptic projections from the dopamine neurons in the ventral midbrain (VM) to the spinal cord in monkeys. Electrical stimulation of the VM induced muscle responses in the forelimb. Thus, a multisynaptic VM.spinal pathway drives motor outputs. From these evidences, we hypothesized that motivational signals activate the VM, which has an impact on motor outputs. We conducted a fMRI experiment while subjects performed a ready-set-go task with monetary incentives. Grip force and reaction time were improved according to the anticipated monetary reward. The VM and motor cortex showed motivation level-dependent activity at the pre-movement. Such pre-movement activity in the VM correlated with subsequent grip force, while that in the motor cortex was associated with subsequent grip force and reaction time. These findings suggest that the VM is a source of force generation driven by motivation.

### [JO05-04]
### Geriatric management of frailty and locomotive syndrome

*Hiromi Rakugi[1] ([1]*Department of Geriatric and General Medicine, Osaka University Graduate School of Medicine*)

Frailty refers to a state of physical, psychological, and social vulnerability in older people due to a decline in the reserve capacity to cope with stress, resulting in various functional declines in daily life. Frailty is a transition between robustness and disability, and the critical point is that frail status is expected to improve with appropriate intervention. The assessment items of frailty include the decline in mobility which is linked to the locomotive syndrome. The decline in mobility often precedes frailty, and the frequency of the locomotive syndrome is higher than that of frailty. It is important to take measures for frailty from the stage of the locomotive syndrome. Furthermore, efforts should be taken for older people and at each stage of the life span, including childhood and adolescence. Under the call of the Japan Medical Science Federation, 79 organizations have agreed to promote measures against frailty and locomotive syndrome in a cross-disciplinary manner, and it is hoped that the medical community will encourage these measures.

### [JO05-05]
### Epidemiology of locomotive syndrome, sarcopenia, and frailty

*Noriko Yoshimura[1] ([1]*Department of Preventive Medicine for Locomotive Organ Disorders, 22nd Century Medical and Research Center, The University of Tokyo*)

According to the recent National Livelihood Survey by the Ministry of Health, Labour, and Welfare, Japan, frailty is the third leading cause of disability. Sarcopenia is characterised by generalised loss of skeletal muscle mass as well as muscle strength and/or function. The diagnostic criteria for sarcopenia overlap with those of physical frailty, suggesting that sarcopenia and frailty are strongly associated. Further, in the above-mentioned National Livelihood Survey, osteoporotic fractures were ranked fourth and osteoarthritis was ranked as the fifth cause of disability. In this context, the term 'locomotive syndrome' was defined as a condition of reduced mobility due to impairment of the skeletal and neuromuscular structures, such as the bones, joints, muscles, and nerves. Advancement of this syndrome results in limited independence and subsequent disability. To prevent disability, it is important to examine epidemiological indices, such as the prevalence of diseases that result in disability. However, little information is available regarding the prevalence and interaction between diseases that lead to disability because only a few population-based studies have been conducted. In this talk, I will present the epidemiological indices and coexistence of locomotive syndrome, sarcopenia, and frailty, based on data collected from individuals in the population-based cohort study entitled ROAD study, which started in 2005.

### Joint Symposium 6

### [JO06]
### 【The Japanese Journal of Physical Fitness and Sports Medicine】
### How would physiology and fitness science benefit the society?

March 17(Thu), 8:30 - 10:30, Room F

### [JO06-01]
### Features and limitations of epidemiological studies from the perspective of history of physical activity guidelines

*Haruki Momma[1] ([1]*Tohoku University Graduate School of Medicine*)

Epidemiology is a method of causal reasoning in the real world but not in an experimental laboratory. Because epidemiological studies can provide evidence to solve problems among populations, many current guidelines, including the Physical Activity Guideline for Health Promotion 2013 (a Japanese physical activity guideline), are generally established based on accumulated epidemiological findings. In sports science and medicine, epidemiology is a relatively new approach compared with other approaches, such as physiology. Although the context of physical activity guidelines shifted to the epidemiological standpoint, early physical activity guidelines mainly based on the findings of physiological studies focused on exercise for performance improvement or cardiac rehabilitation. This paradigm shift allowed end-users, including policy makers, health and non-health professionals, and the public to easily understand the message derived from scientific research, although the explanation on why physical activity is associated with a lower risk of premature death and noncommunicable diseases tends to be neglected. In this symposium, we will introduce the features and limitations of epidemiological studies from the perspective of history of physical activity guidelines.

### [JO06-02]
### The relationship between physical fitness and risk of lifestyle-related diseases: Introduction of epidemiological studies and importance of clarifying the mechanism

*Yuko Gando[1] ([1]*Surugadai University*)

Many epidemiological studies on physical fitness and risk of lifestyle-related diseases have been reported. For example, cardiorespiratory fitness, muscle strength, muscle power, flexibility, reaction time, and balance have been shown to be associated with risk of lifestyle-related diseases. However, epidemiological studies cannot reveal the mechanisms that explain why low physical fitness is associated with lifestyle-related diseases. It is important to provide more convincing scientific evidence when encouraging physical fitness, physical activity, and exercise. Physiological studies play an important role in explaining the mechanism associated with the results from epidemiological studies. Careful explanation of epidemiology and physiology related to risk of lifestyle-related diseases is necessary to encourage the importance of physical fitness and exercise and motivate individuals to engage in regular exercise. In this symposium, we will introduce epidemiological research on physical fitness and risk of lifestyle-related diseases and consider the importance of physiological research to clarify the mechanisms.

### [JO06-03]
### Effects of chroic stress and exercise on the development of hypertension: Involvement of D1 dopamine receptors in the nucleus of the solitary tract

*Ko Yamanaka[1], Makoto Suzuki[1], Keisuke Tomita[1], Miwa Takagaishi[2], Kei Tsukioka[1], Thu Nguyen[1], Linh Pham[3], Sabine Gouraud[4], Hidefumi Waki[1]
([1]*Juntendo Univ.*, [2]*Kansai Univ. of Health Sci.*, [3]*Ochanomizu Univ.*, [4]*Int. Christ. Univ.*)

Chronic stress is a major risk factor for developing hypertension, while daily exercise is useful for stress relief. However, the underlying physiological mechanism remains unclear. In this study, we examined the gene expression profiles of the nucleus of the solitary tract (NTS) of rats subjected to stress and exercise, and determined NTS function on cardiovascular regulation of related genes. Wistar rats were allocated into one of three groups: sedentary (SED), restraint stress for 1 h a day over 3 weeks (ST), and restraint stress with voluntary exercise (ST + EX). Blood pressure was significantly higher in the ST than the SED, with no increase in the ST + EX. Using PCR analysis, we determined that the expression levels of six genes in the NTS, including the dopamine receptor D1 gene, were significantly affected by stress and suppressed by exercise. We observed significantly greater expression of dopamine D1 receptor (D1R) in NTS neurons in the ST than the SED. Furthermore, D1R agonist microinjection into the NTS in anesthetized rats induced hypotensive effects. These results suggest that NTS D1R plays a role in the counteracting processes of stress-induced hypertension.

### [JO06-04]
### mtDNA Polymorphism in the Mitochondrial-Derived Peptide, MOTS-c, is associated with diabetes as well as physical performance

*Noriyuki Fuku[1] ([1]*Juntendo University Graduate School of Health and Sports Science*)

Type 2 diabetes mellitus (T2DM) is an emerging public health problem in the world, especially, in Asia. Although East Asians have lower body mass index compared with Europeans, the prevalence of T2DM is slightly higher in East Asians than in Europeans. An East Asian specific-mtDNA polymorphism: m.1382A>C, causes a K14Q in MOTS-c, which is an insulin sensitizing mitochondrial-derived peptide. Meta-analysis of three cohorts (J-MICC, MEC, and TMM combined-n=27,527) revealed that men with the C allele exhibit a higher prevalence of T2DM than those with A allele. Furthermore, in the J-MICC cohort, only men with the C allele in the lowest tertile of objectively measured physical activity exhibited increased prevalence of T2DM, demonstrating a kinesio-genomic interaction. High-fat fed, male mice injected with wild type MOTS-c showed improved glucose tolerance, but not K14Q MOTS-c mutant. In addition of this finding, C allele of m.1382A>C was associated with higher fast twitch muscle fibers and sprinting performance. These results based on human and animal studies suggest that the MOTS-c K14Q amino acid replacement by m.1382A>C polymorphism is associated with susceptibility to T2DM and sprinting performance in East Asian men, possibly via muscle function such as fiber type composition.

### Joint Symposium 7

### [JO07]
### 【The Japanese Association of Anatomists】
### Recent advances in synapse remodeling

March 18(Fri), 8:30 - 10:30, Room A

### [JO07-01]
### Mechanism to regulate inhibitory synapse formation via dynamic microtubules

*Hirohide Iwasaki[1] ([1]*Gunma University School of Medicine*)

Neurons are connected via synapses to form neural circuits to generate cognitive functions, such as learning, memory and emotion. There are two types of synapses in the mammalian central nervous system; excitatory synapses and inhibitory synapses. Recent studies have revealed that the balance of excitatory and inhibitory synapses is important for normal brain function and the imbalance of them may be causative to some of the psychiatric diseases. In this study, we focused on Teneurin-2, one of the transmembrane protein localized at synapses and play a pivotal role in synapse maturation of both excitatory and inhibitory synapses. Particularly, we focused on the interaction between Teneurin-2 and dynamic microtubules and their role in inhibitory synapse maturation. We found that the inhibition of the interaction between Teneurin-2 and dynamic microtubules reduced the clustering of gephyrin, a marker for inhibitory synapses. We also investigated the precise role of Teneurin-2 on the regulation of dynamic microtubules by using COS-7 cells.

### [JO07-02]
### Activity-dependent dendrite remodeling of developing cerebellar Purkinje cells

*Yukari Takeo[1], Michisuke Yuzaki[1] ([1]*Keio University Graduate School of Medicine*)

Neurons develop stereotyped dendritic morphology specific to their cell type and function. Precise formation of the dendritic arbor is a prerequisite for proper neural circuit assembly. While it is widely accepted that remodeling of synapses depends on neuronal activities, whether and how dendritic structures are dynamically regulated by neuronal activities remains less well-characterized. To address this question, we focused on dendritic development of the cerebellar Purkinje cell *in vivo*. Before forming their distinctive single and flat dendritic trees, immature Purkinje cells exhibit a stellate shape with numerous short dendrites oriented in all directions. Using *in vivo* two-photon time-lapse imaging, here we show how Purkinje cells undergo massive pruning of all but one stellate dendrite and the remaining dendrite grows into the single mature dendritic tree. Overexpression of Kir2.1 suppressed the dendritic pruning, indicating that this process requires Purkinje cells' neuronal activity. Interestingly, deletion of NMDA receptor GluN1 subunit or CaMKII in developing Purkinje cells also disrupted dendritic pruning, suggesting that NMDA receptor/ CaMKII signaling is a key mechanism for the activity-dependent dendritic remodeling. While NMDA receptor is implicated in activity-dependent circuit development in several other brain regions, its role in the Purkinje cells had been elusive. Our results not only uncover the dynamic dendrites but also highlight the general importance of the NMDA receptor in the activity-dependent mechanism of circuit development.

### [JO07-03]
### How do neurons in the brain decide to refine their connections?

*Hisashi Umemori[1] ([1]*Boston Children's Hospital, Harvard Medical School*)

Formation of appropriate synaptic connections is critical for the proper functioning of the brain. Initially, neurons form a surplus of immature synapses. To establish efficient, functional neural networks, neurons selectively stabilize active synapses and eliminate less active ones. This process is known as activity-dependent synapse refinement. However, the manner and molecules by which synapse refinement is regulated remain to be elucidated. We have established mouse *in vivo* systems and showed that inactive synaptic connections are eliminated only when there are other active connections to compete with. Thus, when a subset of inputs is inactive, the inactive inputs are eliminated; contrarily, when all inputs are inactive, elimination does not occur. This suggests that active connections send a "punishment" signal to inactive ones and instruct them to leave by triggering "elimination" signals within the inactive synapses. At active synapses, connections are kept by the presence of "stabilization" signals. In this talk, I will discuss the identification of these signals and how they interact with each other to determine whether to eliminate or stabilize the synaptic connections.

### [JO07-04]
### Astrocyte-Neuron signaling in developmental synapse remodeling

*Naofumi Uesaka[1], Masanobu Kano[2] ([1]*Tokyo Medical and Dental University*, [2]*University of Tokyo*)

The precise formation of neural circuits in the brain during animal development enables animals to achieve various brain function such as perception of their environment and appearance of intelligence. Elucidating the fundamental principles of how neural circuits are formed is an important theme in the field of neuroscience, and will greatly contribute to the development of treatments for neurodevelopmental disorders and the development of innovative artificial intelligence. During postnatal development, neural circuits in the brain are elaborated and brain functions are expressed. One of these cellular basis is "synapse elimination" which is an essential process for the maturation of immature neural circuits into functional neural circuits. Abnormalities in synapse elimination during development and after maturation can lead to various diseases such as autism spectrum disorders, schizophrenia, and dementia, suggesting the importance of synapse elimination. However, the full picture of synapse elimination remains unclear. One of the key to unlock the full picture of synapse elimination is glia. Here, I will present our recent results on the role of astrocytes in the developmental synapse elimination, with a particular focus on astrocyte activity.

### [JO07-05]
### Presynaptic control of spine dynamics for learning and memory in neocortex

*Yoshiyuki Kubota[1,2], Sohn Jaerin[1], Yasuo Kawaguchi[1] ([1]*National Institute for Physiological Sciences*, [2]*SOKENDAI*)

Dendritic spine plasticity is thought to be the cellular basis of learning and memory involving a dynamic orchestration of plasticity in existing spines and the formation and elimination of new spines. Motor learning is correlated with a significant increase in formation of new spines on the apical tuft of layer 5 pyramidal cells in the primary motor cortex (M1) (Xu, T. Nature 2009) and selective artificial shrinkage of dendritic spines in M1 that are potentiated during motor learning disrupts an acquired motor skill (Hayashi- Takagi, A. Nature 2015), indicating that the spine plasticity during motor learning is indispensable for an animal's skill acquisition. However, despite abundant evidence for the correlation between motor learning and postsynaptic spine plasticity, the origin of synaptic inputs to newly-formed spines remains elusive. Spine plasticity depend on the characteristics of presynaptic neural circuits. M1 forms corticocortical networks as well as cortico-subcortical loops of wiring via the thalamus. Understanding network plasticity during learning, therefore, requires concurrent analysis of postsynaptic spine dynamics and presynaptic cell characteristics. We found that distinct neural circuits are involved in formation of new spines and in their maintenance for motor learning and memory. Post hoc characterization of the presynaptic cell types revealed that motor skill improvement coincided with selective formation of spines innervated by corticocortical axons. Chemogenetic silencing of corticocortical input to the motor cortex impaired both motor learning and spinogenesis. Fewer thalamocortical synapses were generated during learning but survived longer with increased spine size compared to new corticocortical synapses. These findings suggest that intracortical circuits that guide the activity of the motor cortex for motor skill acquisition are taken over by inputs from subcortical circuits for subliminal habituation of the acquired skill.

### Joint Symposium 8

### [JO08]
### 【The Chinese Physiological Society (CPS) - The Korean Physiological Society (KPS) - The Physiological Society of Japan (PSJ) joint symposium】
### Ion channels in physiology and diseases

March 18(Fri), 14:15 - 16:15, Room A

### [JO08-01]
### New role of TRPM7 in cell volume regulation mechanism

*Tomohiro Numata[1] ([1]*Department of Neurophysiology, Akita University School of Medicine*)

Animal cells regulate their volume after swelling by the regulatory volume decreases (RVD) mechanism. In epithelial cells, RVD is achieved by KCl release mediated via volume-sensitive outward rectifier $Cl^-$ channels (VSOR) and $Ca^{2+}$ activated $K^+$ channels. It has been previously known that activation of TRPM7 channels induced by cell swelling causes $Ca^{2+}$ influx, thereby activating $K^+$ channels. However, the relationship between TRPM7 and VSOR remained unexamined because VSOR activity is insensitive to intracellular $Ca^{2+}$. Here, we investigated the essential role of TRPM7 in the activation of VSOR. When TRPM7 expression was knocked down in HeLa cells or knocked out in DT40 cells, not only TRPM7 activity and RVD ability but also VSOR activity was suppressed. Furthermore, heterologous expression of TRPM7 in TRPM7-deficient DT40 cells was accompanied by increased expression of LRRC8A, the core molecule of VSOR, and rescued both VSOR activity and RVD ability. It was shown that the promotion of VSOR activity caused by TRPM7 is due to the enhancement of LRRC8A expression mediated by steady-state $Ca^{2+}$ influx and the stabilization of plasma membrane expression of LRRC8A protein by the interaction with the C-terminal domains of TRPM7. From these results, it was newly revealed that TRPM7 plays a role as an essential regulator of VSOR activity in the RVD mechanism.

### [JO08-02]
### Not too little, not too much: protein homeostasis and CLC chloride channel diseases

*Chih-Yung Tang[1] ([1]*Department of Physiology, College of Medicine, National Taiwan University*)

Transmembrane passage of $Cl^-$, the most abundant anion, is mediated by active transporters and ion channels. Despite the unanimous presence of high extracellular $Cl^-$ concentration, a wide range of intracellular $Cl^-$ concentrations exist in different cells, which is attributed to differential expression of various active $Cl^-$ transporters. Consequently, opening of $Cl^-$ channels may lead to either membrane hyperpolarization or depolarization. One of the best studied $Cl^-$ channels/transporters is the CLC gene family, comprising voltage-gated $Cl^-$ channels at the plasma membrane, as well as $2Cl^-/H^+$ exchangers of intracellular organelles. The ClC-1 channel is exclusively expressed in skeletal muscles, whereas the ClC-2 channel is found in virtually all tissues. Mutations in the human genes encoding ClC-1/2 channels are linked to the diseases myotonia congenita, leukodystrophy, and aldosteronism. In addition to anomalous channel gating function, emerging evidence supports the notion that disease-associated mutations may instigate aberrant protein homeostasis of ClC-1/2 channels. In this talk, I will provide an overview of the molecular mechanisms governing ClC-1/2 protein homeostasis.

### [JO08-03]
### Voltage-clamp fluorometry analysis of the hyperpolarized-induced structural rearrangements of ATP-gated channel P2X2

*Rizki Tsari Andriani[1,2], Yoshihiro Kubo[1] ([1]*Division of Biophysics and Neurobiology, National Institute for Physiological Sciences,* [2]*Current affiliation: Integrative Physiology, Graduate School of Medicine, Osaka University*)

P2X2 is a ligand-gated ion channel activated primarily by ATP. The gating of this channel has been shown to be not only dependent on [ATP] but also membrane voltage. Upon hyperpolarization, there is a gradual increase in the inward current in the presence of ATP, despite the absence of a canonical voltage-sensor domain. We aimed to analyze the structural movements of [ATP]- and voltage-dependent gating of rat P2X2 receptor by voltage-clamp fluorometry utilizing fluorescent unnatural amino acid (fUAA). We observed fast and linearly voltage-dependent fluorescence intensity (F) changes at Ala337 and Ile341 in the TM2 domain, which could be due to the electrochromic effect, reflecting the presence of a converged electric field. We also observed slow and voltage-dependent F changes at Ala337, which reflect hyperpolarized-induced structural rearrangements. Furthermore, we identified that the interaction between Ala337 in TM2 and Phe44 in TM1, which are in proximity in the ATP-bound open state, is critical for activation. Taking these results together, we propose that the voltage dependence of the interaction within the converged electric field underlies the voltage-dependent gating. (COI: No)

### [JO08-04]
### Temperature, pH, redox and $Ca^{2+}$: physicochemical regulation of CALHM channel

*Sung Joon Kim[1] ([1]*Department of Physiology, Seoul National University College of Medicine*)

Calcium homeostasis modulator (CALHM) channels show unselective permeability to various ions. CALHM1 channels are unusually slowly activated by depolarization ($I_{CALHM1}$). There are four conserved Cys residues in the extracellular domain that form two intramolecular disulfide bonds. We found that the intramolecular disulfide bonds are essential for the multimerization and membrane trafficking. We found that physiological temperature greatly facilitates $I_{CALHM1}$; leftward shift of voltage-dependence ($V_{1/2}$) and acceleration of activation. A heteromultimeric expression of CALHM1/3 also shows faster activation, and was not thermosensitive. $I_{CALHM1}$ was markedly facilitated by increasing either $pH_o$ of $pH_i$. Based on a homology structure model, we conducted the site-directed mutagenesis of the water-accessible charged amino acids in hCALHM1. We found four charged residues gathering closely in the intracellular space responsible for the alkali-induced facilitation of $I_{CALHM1}$. In addition to pHi, we found that intracellular $Ca^{2+}$ facilitates $I_{CALHM1}$, which was different from previous report. Taken together, we report the remarkable physico-chemical regulation of CALHM1, which might be responsible for the modulation of neurons, taste receptors or immune cells expressing the CALHM family proteins.

### [JO08-05]
### The channel synapse mediates epithelial chemosensory neurotransmission

*Akiyuki Taruno[1,2,3] ([1]*Department of Molecular Cell Physiology, Kyoto Prefectural University of Medicine,* [2]*JST PRESTO,* [3]*JST CREST*)

We originally identified CALHM1 as a pore-forming subunit of a slowly-activating voltage-gated ATP-permeable channel. Subsequently, we discovered that CALHM1 and CALHM3 oligomerize to form a fast-activating voltage-gated channel, CALHM1/3. Both subunits are expressed in taste bud cells (TBCs) and knockout of either subunit abolishes the release of neurotransmitter ATP from TBCs and, consequently, the perception of tastes. Remarkably, instead of synaptic vesicles, TBCs employ a noncanonical chemical synapse involving ion channels as the conduit for neurotransmitter release. We termed this unique chemical synapse "channel synapse". Although the CALHM1/3 channel synapse potentially mediates various cell-cell communications throughout the body, it remains unclear where it exists and functions outside the tongue. Here, we generated a reporter mouse model for *Calhm1* and *Calhm3*, screened > 40 organs for reporter protein expression, and revealed tissue distribution of cells expressing CALHM1/3. In one of those CALHM1/3+ tissues, we genetically, anatomically, and functionally identified the presence of channel synapses and its physiological relevance. The discovery, function, structure, and extra-oral distribution and function of the channel synapse of will be discussed.

### Joint Symposium 9

### [JO09]
### 【The Japanese Pharmacological Society】
### Deciphering inter-organ network: From pathophysiology dissection to drug discovery

March 18(Fri), 14:15 - 16:15, Room D

### [JO09-01]
### Metabolic crosstalk between gut microbiota and hosts

*Takashi Miki[1], Eunyoung Lee[1], Hayato Yoshikawa[1], Ryo Hatano[1], Junki Miyamoto[2], Ikuo Kimura[2,3] ([1]*Department of Medical Physiology Chiba University, Graduate School of Medicine*, [2]*Department of Applied Biological Science, Graduate School of Agriculture, Tokyo University of Agriculture and Technology*, [3]*Laboratory of Molecular Neurobiology, Graduate School of Biostudies, Kyoto University*)

Oral sugar ingestion induces secretion of incretin hormones from enteroendocrine cells. GLP-1 and GIP are the principal incretins that suppress postprandial hyperglycemia. We previously showed that administration of maltose together with $\alpha$-glucosidase inhibitor ($\alpha$- GI) (acarbose or miglitol) increases GLP-1 secretion and that miglitol is more effective than acarbose via its action on SGLT3 in enterochromaffin cells. By contrast, co-administration of maltose plus $\alpha$-GI significantly suppressed GIP secretion. In the present study, we explored the mechanism for the suppression of GIP secretion by maltose and miglitol. We hypothesized that utilization of maltose by gut microbiota may be involved in the GIP suppression. As expected, the suppression of GIP secretion was absent in germ free mice.
Measurement of short chain fatty acids (SCFAs) in the portal vein revealed that an acute, single administration of maltose plus miglitol significantly increased plasma SCFAs levels in specific-pathogen-free mice, but not in GF mice. In addition, the GIP suppression was not induced in mice deficient in GPR41, a SCFA receptor, suggesting that maltose/miglitol administration suppresses GIP secretion through activation of GPR41 by the SCFAs produced by microbiome. Accordingly, sugar metabolism in microbiome plays a critical role in modulating the counterpart in the host through SCFA signaling.

### [JO09-02]
### Role of Brain.Liver Interaction in obesity-related hepatic disorders

*Hiroshi Inoue[1], Emi Hashiuchi[1], Yuka Inaba[1] ([1]*Kanazawa university*)

The brain detects whole-body energetic status and regulates glucose and lipid metabolism in the liver. In this brain-liver interaction, the vagus nerve plays an essential role. We have uncovered the mechanism of the brain-liver interaction and found that the central sensing of increases in plasma levels of nutrients and hormones results in the suppression of the vagal activity, which in turn decreases hepatic glucose production (HGP) through alpha 7 nicotinic acetylcholine receptor (A7nAchR) in Kupffer cells. Activation of A7nAchR is known to suppress inflammatory cytokines expression in Kupffer cells. The acute suppression of hepatic vagal activity releases Kupffer cells from A7nAchR-dependent suppression of inflammatory cytokines, resulting in the IL-6 increase from Kupffer cells, followed by HGP suppression.
Obesity and insulin-resistance impairs the brain-liver interaction. In obese mice, the failure of vagal response to energetic status results in the smoldering activation of Kupffer cells and blunting of acute activation of hepatic IL-6 action and of suppression of HGP. Furthermore, obesity-induced failure of vagal control of Kupffer cell can exacerbates steatohepatitis induced by an atherogenic-diet. These may indicate that the impeded brainliver interaction serves a pathogenic role in hepatic chronic inflammation induced by obesity.

### [JO09-03]
### Organ crosstalk and macrophage in cardiovasdular homeostasis and multimorbidity

*Ichiro Manabe[1] ([1]*Chiba University*)

Macrophages are versatile cells that play central roles in physiological and pathological processes in various organs. Recent studies have unraveled their diverse phenotypes and functions. Macrophage phenotypes are dynamically regulated by microenvironmental cues, including metabolic cues, as well as their differentiation origins. Multimorbidity is a rapidly growing medical and social challenge reflecting in part the aging of society. Heart failure is one of the key diseases of multimorbidity and causes multiple comorbidities, including chronic kidney disease, frailty syndrome, depression, and cancer. Moreover, hospitalization for heart failure is a major future risk for the disease's recurrence and worsening. While these suggest close links among the pathologies, how heart failure increases the future risk of its comorbidities and itself is poorly understood. We found that macrophages are essential for maintaining cardiac homeostasis and play critical roles in the organ crosstalk in multimorbidity. For instance, we previously showed that cardiac macrophages are regulated by the heart-brain-kidney organ network and play a vital role in the cardiac adaptive response to pressure overload. We also showed that cardiac macrophages are essential for the maintenance of healthy electrical conduction. Accordingly, cardiac macrophage dysfunction would lead to cardiac diseases, such as heart failure, which in turn may promote multimorbidity. I will discuss the roles of macrophages and organ crosstalk in heart failure and multimorbidity.

### [JO09-04]
### Mitochondrial Medicine

*Takaaki Abe[1] ([1]*Division of Medical Science, Tohoku University Graduate School of Biomedical Engineering/Medicine*)

Mitochondrial disease is a rare disorder that causes damage to mitochondria, the energy-producing factories in cells, resulting in decreased energy production (ATP) in the neuromuscular, cardiovascular, metabolic, renal-urinary, hematological, visual, endocrine, and digestive systems. At present, however, there is no treatment other than taurine that has been proven to be effective in a rigorous clinical trial. We have developed Mitochonic acid-5 (MA-5), a novel compound for mitochondrial diseases with a completely new mechanism different from existing drugs. MA-5 inhibits cell death in cultured cells derived from patients with mitochondrial disease. Moreover, it improved cardiac and renal respiratory function and increased survival rates in mitochondrial disease mouse models by accelerating ATPase dimerization through binding with mitofilin/Mic60.
With the support of AMED Moonshot Research and Development Project. we conduct a Phase I clinical trial from January 2022 to confirm the safety of MA-5, which is expected to be the world's first mitochondrial disease treatment from Japan, in healthy adult subjects. This clinical trial of MA-5 is expected to lead to the inhibition of the progression and treatment of mitochondrial diseases, which are rare and intractable diseases for which there is currently no effective treatment, and in the future, MA-5 is expected to be helpful in the prevention and treatment of diseases such as cardiac failure, deafness, sarcopenia, ALS, and Parkinson's disease, which are also caused by reduced mitochondrial function.

### Local Meeting 1

**【The Physiological Society of Chubu Area】**
**The research projects developing in The Chubu Physiological Society**

March 16(Wed), 9:00 - 11:00, Room B

### [LM01-01]
### Thermal physiology research in Nagoya University

*Kazuhiro Nakamura[1] ([1]*Department of Integrative Physiology, Nagoya University Graduate School of Medicine*)

The history of thermal physiology research at the Department of Physiology, Nagoya University School of Medicine was founded by Dr. Yas Kuno, who became the professor in 1937. Prof. Kuno conducted the pioneering research on human perspiration (sweating), the results of which have been recognized worldwide and constitute the basis of our current knowledge on human thermoregulation. For this achievement, Prof. Kuno was awarded the Order of Culture in 1963 and was nominated for the Nobel Prize. Prof. Kentaro Takagi inherited the history of thermoregulation research in the department in 1955 and further developed it into research on autonomic reflexes. Profs. Kuno and Takagi produced many students who became professors in physiology laboratories all over Japan. Unintentionally, Kazuhiro Nakamura, who is not a pupil of Prof. Kuno and his successors, became a professor of the department in 2015 and is currently conducting research on the central circuit mechanism of thermoregulation in rodents. This circuit has now been found to also function to protect the organism from various environmental stresses, such as infectious diseases, psychological stress and starvation.

### [LM01-02]
### Remodeling of Cortical Circuits in vivo: Glia-Neuron Interaction

*Junichi Nabekura[1] ([1]*National Institute for Physiological Sciences*)

Following a brief introduction of recent approach of NIPS on physiological sciences, two topics regarding the contribution of microglia and astrocyte to the remodeling of cortical synapses in vivo will be introduced. Microglial contact onto the synapses facilitates the synaptic transmission. (Wake 2009, Akiyoshi 2018). Microglial contact onto the damaged neurons prevents the neuronal excitotoxicity (Kato 2016). In immature brain, microglial contact onto the neuronal dendrite induces the formation of synapses and contributes cortical local circuits (Miyamoto 2016). In chronic pain model mice, the enhanced activity of astrocyte during the developmental phase of allodynia accelerates synapse remodeling in the primary somatosensory cortex (S1), possibly leading to the formation of pathological circuits (Kim 2011, 2016). Recently, we attempt to remove the pathological circuits with reactivation of astrocyte in the maintenance phase. Reactivation of astrocyte in combination of peripheral nerve block successfully eliminate the synapses possibly related to hypersensitivity and recover the normal sensation. This could be the new therapeutic approach of chronic pain in clinics.

### [LM01-03]
### $Cl^-$ homeodynamics and Multimodal GABA actions: research began in Chubu

*Atsuo Fukuda[1] ([1]*Hamamatsu University School of Medicine*)

GABA generally induces hyperpolarization and inhibition in the adult brain, but causes depolarization (and can be excitatory) in the immature brain. Because $GABA_A$ receptors are $Cl^-$ channels, alternating GABA actions between hyperpolarization ($Cl^-$ influx) and depolarization ($Cl^-$ efflux) are induced by changes in the $Cl^-$ gradient, which is regulated by $Cl^-$ transporters, e.g., $Cl^-$ exporter KCC2 and importer NKCC1. Thus, the dynamics of neural functions are modulated by "active" $Cl^-$ homeostasis (*$Cl^-$ Homeodynamics*), alternating inhibition and excitation, and could underlie the modal shifts in cellular and network oscillations. This depolarizing or hyperpolarizing mode of GABA actions change during development and in phathogenesis. In addition to *$Cl^-$ Homeodynamics*, synaptic or nonsynaptic/extrasynaptic mode of GABA actions (*Multimodal GABA*) also change during development and in pathogenesis, e.g., ambient GABA before synaptogenesis is essential agonist for neurogenesis and migration and extrasynaptic spillover GABA is an essential mode of GABAergic functions. Above ontogenic modal shift in GABA actions is required for normal development, but occasionally adversive reverse modal shift in adult also occur. Thus disturbances in this ordinal developmental GABA modal shift and abnormal temporal window of depolarized (excitatory) GABA action could underlie the pathogenesis of diverse neurodevelopmental disorders and neurological diseases.

### [LM01-04]
### Contribution of the Chubu Physiological Society to develop new research projects

*Yasutake Shimizu[1], Shiina Takahiko[1] ([1]*Dept Veterinary Physiology, Faculty of Applied Biological Sciences, Gifu University*)

Originally, we conducted biochemical experiments, but for various reasons we started research on the gastrointestinal motility. We considered that it would be more appropriate to present our data at the meeting of the Physiological Society, so we joined the Society in 2007. However, for newcomers, the annual meeting of the Society was too huge to build up our research network. In contrast, the local association, the Chubu Physiological Society, was of great value. Since there is only one venue at the meeting of the Chubu Physiological Society, we can see all the presentations, being helpful to know who is doing what kind of research. In addition, in a social gathering held after presentation, we can discuss specific points in a relaxed state. Advises during such a gathering were valuable to develop new research projects. In some cases, undergraduate students showed preliminary data, and thanks to frank advice from the members of the Chubu Physiological Society, they got motivated and went on to the PhD course. Including such episodes, we will show the outline of our gastrointestinal research.

### [LM01-05]
### Physiological research for recovery of motor disturbance: research developed in Chubu

*Hideki Hida[1] ([1]*Dept Neurophysiol & Brain Sci, Nagoya City Univ Gad Sch Med Sci*)

We are challenging three projects of animal function in physiology; transplantation of oligodendrocyte progenitor cells to neonatal white matter injury for the recovery of motor dysfunction, analysis of the recovery mechanism by rehabilitation after intracerebral hemorrhage (ICH), and research of the formation mechanism in emotion during development. All project data were presented in The Chubu Physiological Society to obtain constructive suggestion, followed by preparation to publish. In this meeting, we will present recent progress of our research, especially focusing on the recovery mechanism by forcedlimb use (FLU) after ICH. FLU caused the recovery of forelimb function in rats, revealing causal relationship between red nucleus and functional recovery. As the selective blockade of the cortico-rubral tract (CRT) was gradually disappearing, additional corticoreticular tract (CReT) was blocked by DREADD system, resulting in severe impairment of the forelimb function again. Thus, CRT has causal link to FLU-induced recovery of forelimb function after ICH, indicating more dominant role in FLU-induced reorganization and recovery compared to CReT.

### Local Meeting 2

**【The Physiological Society of Tokyo Area】**
**Towards the next decade of the Regional Physiological Society Meeting of Tokyo Area**

March 16(Wed), 15:45 - 17:45, Room B

### [LM02-01]

### Functional analysis of enteroendocrine cells by live cell imaging

*Kazuki Harada[1], Takashi Tsuboi[1] ([1]*Department of Life Sciences, Graduate School of Arts and Sciences, The University of Tokyo*)

Enteroendocrine cells, especially L cells in the small intestine sense nutrients, hormones and neurotransmitters to secrete glucagon-like peptide-1 (GLP-1) to promote insulin secretion from pancreatic β cells and reduce appetite via vagus nerve. However, precise mechanisms underlying GLP-1 secretion are largely unknown. We have performed visualization of intracellular signaling molecules, cytoskeleton, ion channels, and GLP-1 vesicles to elucidate the GLP-1 secretion mechanisms. Firstly, we investigated the effect of lysophosphatidylinositol (LPI) on GLP-1 secretion. We found that LPI induced the translocation of transient receptor potential vanilloid 2 (TRPV2) channels to the plasma membrane and increased intracellular $Ca^{2+}$ levels to promote GLP-1 secretion. Secondly, we developed cAMP, cGMP, lactate, pyruvate, and glucose indicators to visualize their intracellular dynamics during GLP-1 secretion. In fact, we found that particular secretagogues induced the increase in both intracellular $Ca^{2+}$ and cAMP and GLP-1 secretion in L cells, which is also regulated by actin polymerization. In this symposium, we will introduce and discuss the results of these studies.

### [LM02-02]

### Physiological roles and regulation of gluconeogenesis in the kidney

*Ryo Hatano[1], Eunyoung Lee[1], Takashi Miki[1] ([1]*Department of Medical Physiology, Graduate School of Medicine, Chiba University*)

The advent of $Na^+$-glucose cotransporter 2 (SGLT2) inhibitor brings innovative therapeutic options for the treatment of Diabetic Mellitus, and it has brought attention to renal glucose metabolism. Under normal circumstances, virtually all of glucose filtered by the glomerulus is substantially reabsorbed in the renal proximal tubules. This reabsorption is mediated by SGLT2 (located at S1, and S2 segments of proximal tubules) and SGLT1 (located at S3 segment). Proximal tubules play major roles in the glucose reabsorption, whereas those cells do not metabolize glucose for the energy production due to the loss of glycolytic enzymes. Fatty acids are used as main fuel source for the generation of high amounts of ATP in the proximal tubules. On the other hand, proximal tubules express gluconeogenic enzymes including G6Pase and PEPCK, rate-limiting enzymes for gluconeogenesis, and capable of supplying glucose to the body. Thus, renal proximal tubules play important roles in the regulation of glucose homeostasis in the body.Under fasting conditions, expressions of gluconeogenic enzymes are induced in the both liver and kidney. During several hours of fasting, hepatic glycogenolysis and gluconeogenesis are induced. In prolonged fasting, kidney participation in gluconeogenesis is increased, suggesting that renal gluconeogenesis is controlled by the different mechanism from hepatic gluconeogenesis. We recently found a novel regulatory mechanism of renal gluconeogenesis, mediated via inter-organ crosstalk. In this session, I would like to discuss the physiological roles of renal gluconeogenesis in the blood glucose control.

### [LM02-03]

### Verification of potential hypotheses related to skeletal muscle involvement in Nuclear envelopathy

*Eiji Wada[v], Yukiko Hayashi[1] ([1]*Tokyo Medical University, Department of Pathophysiology*)

Mutations in *LMNA* encoding lamin A/C and *EMD* encoding a nuclear membrane protein, emerin, cause myopathy called Emery-Dreifuss muscular dystrophy (EDMD). Potential hypotheses are proposed to underpin the pathogenesis of this complicated disease: 1) the structural hypothesis focuses on abnormal nuclear shape associated with fragile nuclear membrane; 2) the gene expression hypothesis concerns impairment of chromatin organization and signaling pathways; 3) the stem cell hypothesis relates to abnormality in skeletal muscle stem cells. We recently generated a suitable murine model (Emd-/-/Lmna$^{H222P/H222P}$; EH) for studying skeletal muscle involvement in EDMD. In this report, the structural hypothesis and the stem cell hypothesis were examined by recovery from cardiotoxin-induced skeletal muscle damage in EH mice. Function of satellite cells was maintained in EH mice that muscle pathology and function were significantly recovered after CTX injection. Surprisingly, abnormalities in nuclear shape were also improved. Although nuclei were still maintained in good shape after 126 days post CTX injury, muscular dystrophy was progressed in EH mice. Therefore, disease-associated dystrophic phenotypes in EDMD cannot be explained by structural and/or stem cell hypotheses.

### [LM02-04]

### The history of the Regional Physiological Society Meeting of Tokyo Area and the personal history of physiological research

*Fusao Kato[1] ([1]*Dept Neurosci, Jikei Univ Sch Med*)

The Regional Physiological Society Meeting of Tokyo Area (RPSM-Tokyo), which started before WWII, always had a policy of assuring liberty of organizing the Meeting according to the organizers' interest, which might be biased towards specific subjects. This policy was made possible by a large diversity of physiologists with a wide range of research directions in the Kanto region. Many researchers enjoyed this policy. The first RPSM-Tokyo I attended was the 217th Meeting (Showa Univ.) in June 1984, and my first presentation was on the spectral analysis of nerve discharges recorded in anesthetized rabbits at the 219th Meeting (Nihon Univ. Hospital) in June 1985. In 2005, I organized the 241st Meeting at the newly built auditorium in the Jikei Univ. In 2008, I was invited to give a Special Lecture on "astrocyte-neuron interaction at the central synapses" at the 242nd Meeting (Keio University). In September 2015, I gave a symposium talk on "Visualizing pain network - from optogenetics to ultra-high filed MRI" in the 246th Meeting (Toho Univ.). Everyone should feel that the history of one's presentations at RPSMs-Tokyo reflects the evolution of the physiological research, which is made possible by that liberal policy of this regional Meeting.

### Local Meeting 3

### 【The Physiological Society of Kinki Area】 "Developing new ideas based on study of the past" in Kinki Physiological Society

March 17(Thu), 8:30 - 10:30, Room B

### [LM03-01]

**Central gustatory processing elucidated by optogenetic identification of the gustatory pathway**

*Shogo Soma[1], Kengo Nomura[1], Naofumi Suematsu[2], Tatsuro Murakami[1], Akiyuki Taruno[1,3,4] ([1]*Department of Molecular Cell Physiology, Kyoto Prefectural University of Medicine*, [2]*Department of Bioengineering, University of Pittsburgh*, [3]*PRESTO, JST Sakigake*, [4]*JST CREST*)

Gustation controls feeding behaviors. A gustatory experience consists of three processes: detection of chemicals by taste cells in the taste buds, cognitive process and value judgment in the brain. Intensive studies at the periphery have recently identified the cellular and molecular mechanisms of all five basic tastes. In contrast, the neural mechanisms of gustatory information processing in the brain remain enigmatic from previous small-scale neuronal recordings, prone to contamination from other sensory inputs, i.e., touch. In this study, we developed a method for identifying "true" taste-responsive brain neurons, combining optogenetic activation of taste cells on the tongue and simultaneous large-scale electrophysiological neuronal recording in the brain. Recordings were made in mice with neurolept anesthesia. Responses to five basic tastes were measured in several brain regions, including the brainstem (NST), pons (PbN), thalamus (VPMpc), and amygdala (CeA). The data demonstrate that each region contains various taste-responsive cells with a distinct pattern and suggest the logic of multi-layered gustatory information processing.

### [LM03-02]

**Correlation between activity and spatial dynamics of ion channels mediated by inositol phospholipids**

*Daisuke Yoshioka[1], Yasushi Okamura[1] ([1]*Integrative Physiology, Graduate School of Medicine, Osaka University*)

Potassium voltage-gated (Kv) channels regulate electrical excitation in a various tissue. PIP2, a phosphatidylinositol lipid, is involved in the regulation of Kv channels, through two possible mechanisms: channel gating control and spatial dynamics control. While many studies have focused on the former, verification of the latter has been left behind. PIP2 also regulate exo/endocytosis and shows a heterogeneous distribution on cell surface. The PIP2-localized regions are likely to effect on the channel dynamics, and the dynamics changes are expected to be related to the channel activity. In this study, we will analyze the channel-PIP2 interaction using single molecule imaging, and try to clarify the correlation between PIP2-mediated channel activation and dynamics.

### [LM03-03]

**Emerging roles of neuronal $Ca^{2+}$ sensor-1, a $Ca^{2+}$ regulatory protein in excitable tissues**

*Tomoe Y Nakamura-Nishitani[1] ([1]*Dept. Pharmacol. Wakayama Medical Univ. Sch. Med.*)

Intracellular $Ca^{2+}$ regulates a variety of cellular processes including muscle contraction and synaptic transmission. The functions of $Ca^{2+}$ are mediated by a large family of $Ca^{2+}$-sensor proteins. One of these, neuronal $Ca^{2+}$ sensor-1(NCS-1) is predominantly expressed in neuronal tissues and is suggested to play an important role in neuronal functions, such as synaptic plasticity. However, its detailed mechanism or molecular targets as well as the functions of NCS-1 in other tissues were almost unknown. In these 20 years, we have investigated novel roles of NCS-1 in excitable tissues, and found that NCS-1 is a regulator of Kv4 $K^+$ channels, it works as a survival factor up-regulated in injured neurons, and contributes to spatial learning and memory in mice. Not only in neuronal tissues, we found some previously unrecognized functions of NCS-1 in the heart. NCS-1 is highly expressed in immature hearts, and promotes contraction and cardiac hypertrophy via activation of $IP_3$R-mediated intracellular $Ca^{2+}$ signals. We are now investigating other emerging roles of NCS-1 including regulation of energy metabolism and pain relief. In this symposium, I will summarize what we have clarified so far and what we are planning to do about NCS-1 and related proteins, and discuss how whole-body function is precisely regulated by $Ca^{2+}$ signals.

### [LM03-04]

**Cortical mechanism of binocular stereopsis**

*Ichiro Fujita[1] ([1]*Osaka University Graduate School of Frontier Biosciences*)

Binocular disparity is a precise cue for depth perception. To encode binocular depth correctly, the visual system needs to find corresponding visual features in the left-eye and right-eye images. The task of finding this binocular correspondence is not achieved at the initial stage of binocular processing, the primary visual cortex (V1). V1 neurons are sensitive to disparities of falsely matched features in addition to disparities of correctly matched features, because they detect binocular disparity by computation similar to interocular cross-correlation. Further computation is required for an accurate representation of the disparity by discarding responses to false matches (matching computation). For perceiving depth, the visual system exploits both the primitive, correlation-based signal and the more elaborate, match-based signal. Human observers judge depth based on a weighted sum of outputs from the two computations. The relative weight varies under different stimulus conditions. Studies of single neuron responses in the monkey visual cortex suggest that area V4 neurons carry signals consistent with the solution to the correspondence problem, whereas area V5/MT neurons shows responses intermediate between the correlation-based and match-based representations. The correlation-based and match-based signals in these cortical areas are likely to contribute to stereo perception in a parallel manner.

### [LM03-05]

**My research in the Kinki Physiological Society**

*Harunori Ohmori[1] ([1]*Kyoto University*)

After joining the Kinki Physiological Society in 1991, I have studied properties of brainstem auditory neurons and neural circuits until 2015 when I retired the Kyoto university. In this symposium, I will summarize my research during that period. Major finding is that auditory signals are processed in a sound frequency dependent manner in coordinated activity of several ion channels and synapses. For example, in the NL where binaural sound signals are processed, the low voltage activated Kv1.2 channels are expressed rich in the midfrequency neurons and improve the precision of ITD (interaural time difference) processing. HCN channels improve the processing of ITD in the high-frequency neurons.

The processing of low-frequency ITD is improved by the inhibitory inputs from SON in a sound level dependent manner. Moreover, the phase locked activity of the inhibitory interneuron between NM and NL improves the ITD sensitivity of the low-frequency NL neurons when the sound level is low. Accordingly, the precision of ITD processing in NL is improved through the coordinated activity of several ion channels and inhibitory neural circuits in the brainstem auditory nuclei.

### Local Meeting 4

### 【The Physiological Society of West Japan Area】 Scientific Activity in The Physiological Society of West Japan: Past, Present and Future

March 17(Thu), 16:00 - 18:00, Room B

### [LM04-01]
### Dawning Age of The Physiological Society of West Japan

*Masaki Kameyama[1] ([1]*Department of Physiology, Kagoshima University Graduate School of Medical and Dental Sciences*)

In this talk, I will review activities in the early stage (1949-1984) of the Physiological Society of West Japan. The first meeting of the society was held in November 1949 in the campus of School of Medicine, Kyushu University as "The Colloquium of Physiology in Kyushu", and organized by Profs. A. Seo and N. Toida, Kyushu University. At that time, institutions of physiological sciences in Kyushu and its neighbor area were only 7, i.e., Physiology Departments of Kyushu University, Kumamoto University, Nagasaki University, Yamaguchi Medical School, Kurume Medical School and Kagoshima Medical School, and Mitsui Institute of Occupational Medicine. Since then, 27 meetings were organized until 1976 almost annually on a rotation system, and Kyushu Dental University (1961 and 1970) and Tottori University (1966) have joined the Society and organized the meetings. In that period, 20-40 oral presentations were given in two days. Some meetings were held in resort (hot spa) places or in Chugoku region (Western Honshu). In 1970-80's, a number of new medical and dental faculties/schools has been established in Kyushu/Okinawa area, i.e., University of Occupational and Environmental Health, Fukuoka University, Fukuoka Dental College, Saga Medical School, Oita Medical University, Miyazaki Medical College, Ryukyu University. Furthermore, departments of physiological sciences of medical/dental faculties/schools and nursing/co-medical schools, as well as other areas have joined the Society. Then, number of presentations increased up to 74, and poster presentations are introduced in addition to oral ones. I have no conflict of interests.

### [LM04-02]
### Tradition of the Studies on the Cardiac Pacemaker in The Physiological Society of West Japan

*Makoto Takano[1], Kensuke Oshita[1,2], Noriyuki Nakashima[1] ([1]*Department of Physiology, Kurume University School of Medicine,* [2]*Department of Anesthesiology, Kurume University School of Medicine*)

In the normal heart, the hyperpolarization activated, cyclic nucleotide sensitive, nonselective cation channels (HCN1-4) are specifically expressed in the cardiac conduction system (CCS), and HCN4 is major subtype in the sinoatrial node (SAN). In the heart failure, the expression of HCN2 is reportedly increased in the ventricular myocytes. To explore the pathophysiological role of HCN channel family, we generated the transgenic mouse in which the expression level of HCN4 could be changed between 0 to ~3 times that of wild type mice with doxycycline. We also generated the transgenic mouse in which HCN2 was specifically overexpressed in the heart. We found that HCN4 antagonized the hyperpolarization induced by acetylcholine, and attenuated the negative chronotropic effect of parasympathetic nerve stimulation. Thereby, HCN4 stabilized spontaneous firing of SAN. In the ventricular myocytes, HCN2 channel was constitutively activated by adrenergic stimulation or by hypokalemia-induced membrane hyperpolarization. We concluded that HCN2 channel overexpressed in the ventricular myocyte increased the arrhythmogenicity under these conditions. (COI:No)

### [LM04-03]
### Peptides Researchers in Action on The Physiological Society of West Japan

*Yoichi Ueta[1] ([1]*Department of Physiology, School of Medicine, University of Occupational and Environmental Health, Japan*)

Among the members of The Physiological Society of West Japan, there are a number of physiological researchers who study physiological roles of various peptides. Distinguished peptide hunters who discovered novel peptides such as ghrelin, natriuretic peptide family, adrenomedullin, neuromedins and PACAP have acted in Kyushu Islands. In addition, there are also peptide researchers who study popular peptides such as orexins (identical to hypocretins) and neurohypophysial hormones (vasopressin and oxytocin). Our laboratory has consistently studied the hypothalamic-pituitary system which is a typical neuroendocrine system. More than thirty years ago, it was memorable that I gave an oral presentation for the first time at the 37th annual meeting of The Physiological Society of West Japan. Since then, I have continued to study the physiological properties of vasopressin and oxytocin neurons in the hypothalamus, using electrophysiology, molecular physiology and genetically modified animals. In this presentation, the current status of peptide researches will be reviewed in Kyushu Islands, including our recent studies. I declare that I have no conflicts of interests.

### [LM04-04]
### The Role of Sympathetic Nervous System and Cell Death Signaling in Development of Heart Diseases

*Takayuki Fujita[1] ([1]*Fukuoka University*)

The purpose of our research is to improve current therapy for heart diseases including heart failure and arrhythmias. We focus on sympathetic nervous system, cell death signaling, etc.

Exchange protein directly activated by cAMP (Epac) is one of the target molecules of cAMP, an important second messenger. In various types of cells including cardiomyocyte, sympathetic activation-induced cAMP elevation causes important effects through Epac, independent of PKA. We found the pivotal role of Epac in development of heart failure and arrhythmias. In addition, we reported usefulness of Epac inhibiting therapy in prevention of atrial and ventricular arrhythmias in mouse study.

Translationally controlled tumor protein (TCTP) is one of the a highly conserved protein that is expressed ubiquitously in mammalian tissues including heart. Although TCTP is thought to be involved in regulation of cell death and survival, there had been no report on the function in cardiomyocyte. Recently, we found crucial role of TCTP in cardiomyocyte survival.

Based on these researches, we aim to develop more effective and safe treatment for cardiovascular diseases.

### Local Meeting 5

### 【The Physiological Society of Chugoku-Shikoku Area】
### Encouragement to young investigators in Chugoku-Shikoku region

March 18(Fri), 8:30 - 10:30, Room B

### [LM05-01]
**History of endeavor to encourage young investigators in the Chugoku-Shikoku region**

*Sei Kobayashi[1,2] ([1] *Department of Advanced Preventive Medicine, Yamaguchi University School of Medicine,* [2]*Department of Molecular and Cellular Physiology, Yamaguchi University Graduate School of Medicine*)

In the Chugoku-Shikoku region of the Physiological Society of Japan, with the great helps with Prof. Tokuda (Kagawa Univ.) and Prof. Matsui (Okayama Univ.), we started and have been trying to encourage young investigators by several endeavors, including the establishment of the young investigator awards, increase in chances of communication between established scientists and young investigators, etc. In this symposium, we are going to explain the history of these exciting endeavors.

### [LM05-02]
**Signal transduction pathways for perioral sensory signals to the cerebellar Purkinje cells**

*Reika Kubo[1], Kouichi Hashimoto[1] ([1] *Department of Neurophysiology, Hiroshima University Graduate School of Biomedical & Health Sciences*)

Perioral sensory signals are transmitted to the cerebellar Purkinje cells (PCs), generating complex spikes (CSs) via climbing fibers from the inferior olive (IO), and modulating simple spikes (SSs) activities via mossy fibers emerging from the pontine nuclei. However, signal transduction pathways from the perioral area to these precerebellar nuclei have not become clear. To find these anatomical pathways, we recorded CSs and SSs evoked by electrical stimulations of the infraorbital nerve (ION) from the PCs in CrusII in anaesthetized mice. We found that CS generations by ION stimulations were strongly suppressed by local injections of a $GABA_A$ receptor agonist, muscimol, into around the mesencephalic reticular nucleus (MRN). The ION stimulations evoked early and late peaks of SS firing, followed by a period when SS firing was silenced (SS silence). The late SS peak and the following SS silence were also suppressed by local muscimol injections into around the MRN. These data suggest that the mesodiencephalic area involving the MRN is crucial for signal transduction from the perioral area to the cerebellar PCs.

### [LM05-03]
**Therapeutic effect of *Ophiocordyceps sinensis* on pulmonary hypertension via TRPM7 inhibition**

*Keizo Hiraishi[1], Lin-Hai Kurahara[1], Katsuya Hirano[1] ([1] *Department of Cardiovascular Physiology, Faculty of Medicine, Kagawa University*)

**Background and purpose:** Pulmonary hypertension (PH) comprises a multifactorial group of pulmonary vascular disorders that cause pulmonary vascular remodeling and right heart failure. As a promising therapeutic target, we explored the potential significance of Transient receptor potential melastatin subfamily member 7 (TRPM7) channel, which promote the cardiovascular remodeling. In this study, we explored the therapeutic potential of TRPM7 antagonist *Ophiocordyceps sinensis* (OCS), an entomopathogenic fungus, for PH.
**Method:** OCS treatment was performed in the monocrotaline-induced PH rats. The monocrotaline pyrrole-induced PH model was created in genetic TRPM7 knockout mice. Electrophysiological experiments were performed to evaluated the effect of OCS on TRPM7 channel activity. TGF-β2-induced endothelial-to-mesenchymal transition (EndoMT) in human pulmonary arterial endothelial cell (HPAEC) was assessed by western blot and immunocytochemistry. Proliferation of the PH patient-derived pulmonary arterial smooth muscle cells (HPASMC) was assessed by MTT assay.
**Results:** OCS ameliorated the development of PH, right ventricular hypertrophy and dysfunction in the PH rats and mice. The genetic knockout of TRPM7 in mice attenuated the development of PH. TRPM7 expression is detected in intimal and medial walls of pulmonary artery and plexiform lesions of both PH rats and patients. OCS suppressed the TRPM7 channel activity and TGF-β2-induced EndoMT in HPAEC. OCS suppressed proliferation of HPASMC.
**Conclusion:** OCS mitigates vascular remodeling associated with pulmonary hypertension. The inhibition of TRPM7 is suggested to underlie the therapeutic effect of OCS.

### [LM05-04]
**Efforts to foster basic M.D. researchers and my own career choice**

*Kazuya Miyanishi[1] ([1]*Ph.D. Program in Humanics, School of Integrative and Global Majors, University of Tsukuba*)

In recent years, the number of M.D. researchers from medical schools has been decreasing dramatically. Amid calls for a "medical crisis" in obstetrics/gynecology and emergency medicine, the other medical crisis due to a shortage of basic M.D. researchers has not received much attention. The shortage of M.D. researchers is expected to lead to the decline of basic medical research and translational research and the collapse of medical education for the next generation.
In this symposium, I will present the efforts of my alma mater Ehime University and The Physiological Society in Chu-shikoku region to foster young M.D. researchers, as well as how I entered graduate school directly after graduating from the medical school without junior resident. In particular, Ehime University provides opportunities to engage in specialized medical research in basic and clinical laboratories from the first grade as a compulsory lecture in order to foster research-oriented medical students. To foster M.D. researchers and medical students with a research mindset, it is essential to design a system that promotes motivation to engage in basic research after graduation throughout medical schools, both clinical and basic.

### Local Meeting 6

### 【The Physiological Society of Tohoku Area】 What we want to share with you based on our research so far

March 18(Fri), 14:15 - 16:15, Room B

### [LM06-01]
**Past, present, and perspective of brain research on motor learning**

*Kiyoshi Kurata[1] ([1]*Hirosaki University Graduate School of Medicine*)

While I studied brain mechanism of sensorimotor transformation in cortical motor areas, namely ventral premotor cortex (PMv), of nonhuman primates, I have been having a special interest how motor learning is achieved especially in prism adaptation that is quickly accomplished within around 10 trials, perhaps by readily switching of neural circuits. Although the PMv plays a crucial role in the behavior, it became more evident that cerebrocerebellar loop is involved in such type of motor learning. For the adaptive behavior, it seems essential that, when motor errors are generated, spatial difference between target and reaching locations should be evaluated immediately after the reaching was completed. It has been revealed recently that motor error signals are reflected in complex spikes of Purkinje cells in the cerebellar cortex. However, little is yet known how the motor error is precisely computed and utilized for the adaptation in brain. I will propose what should be directed to solve this important issue in future studies.

### [LM06-02]
**Physiology in the coming space era - looking back on the resarch life of an old physiologist**

*Tsuyoshi Shimizu[1,2] ([1]*Shimizu Institute of Space Physiology Suwa Maternity Clinic* , [2]*Fukushima Medical University(Prof. emeritus)*)

Almost 60 years have passed since my research life started at the 2nd Department of Physiology Shinsyu University School of Medicine under Prof. K.Miyakawa. The main subject of my research was the regulation of cardiovasucular functions. On the way to the present time I was interested in postnatal development of the aortic baroreflex, and to desolve a question occured in this particular topic, I had an opotunity to perform animal experiments with the space shuttle Columbia in 1998. With this oppotunity my interest in effects of the space embilonment on the life sprread out widly, although actually, it was a small experiment which I did at a laboratory of University of Illinois under Prof. F.R.Steggerda that I first realized real activitues of humankind in the space environment. The experiment was related to intestinal gas production of space food prepared for astronauts of Apollo 11 who were planning to land on the moon. It is now the time when activities of human being in the space environment increase more extensively. In this symposium I will discuss about what research in physiology and learning/studies in the future should be, introducing shortly my research experiences. The most emphatic message of my wishes is that any physiological research should not be performed nor be utilized for war.

### [LM06-03]
**"Everything physiology", "problem orientation", "scrap and build", and "optogenetics"**

*Hiromu Yawo[1] ([1]*The University of Tokyo*)

I had three mentors during my early carrier. Prof. Akira Inoue in Kyoto University Department of Physiology did not give any research theme to the students. Each graduate student had to find what to do by oneself. He welcomed any kind of research under one' s self-responsibility. His successor, Prof. Motoy Kuno had a motto that we have to research in a problem-oriented manner and should not be method-oriented. That is, we should apply any materials and methods to solve an unanswered issue. On the other hand, we should not apply the same methodology to investigate other similar problems. When being in Washington University, St. Louis as a postdoc, I learned from Prof. Dale Purves that there are important unsolved problems where it appears to be solved at one glance. But, to study such difficult issues, you should not adjust your experiments to the equipment, rather you have to build and optimize the total experimental system to the purpose. I have been guided by these lessons throughout my academic life. In this way, the novel methodology now called optogenetics, was born to open the new horizon of physiology to investigate the rules underlying biological systems.

### [LM06-04]
**That's not true! A magic wand he gave me.**

*Katsuya Yamada[1] ([1]*Department of Molecular Transport, Hirosaki University Graduate School of Medicine*)

It was an unforgettable experience. For the first time when I collaborated with a guy in the foreign institute, the lab I stayed was filled with joy. Because we were informed that his paper was just accepted by Science. But he never bragged about it. He told me during a coffee break outside of the lab that he was very much impressed by a Japanese movie named, the Ballad of Narayama, and said that was also a serious story in his culture. We became friends. We designed and performed a new *in vivo* experiment together combining his setup and a machine I carried from Japan. At midnight on one day, we thought we succeeded to get the first convincing result. The lab director soon returned from a party held by a politician. We wanted him to be glad and said "We obtained a very beautiful data." However, he said unexpected words, "I don't believe it. That's not true!". We're super pissed off, and discussed immediately how to completely shut him up. We performed two very strong control experiments. A young programmer helped us by making a new software. When we presented the control data, he was grinning at us and said, "Let's publish!" He gave me a magic wand through this impressive lesson.

### Planning Symposium 1

### [PS01]
### 【Committee for Young Physiologists】
### Revisiting the History of Physiology

March 16(Wed), 9:00 - 10:00, Room D

### [PS01-01]
### First and last experiments in my research world on muscle contraction

*Haruo Sugi[1] ([1]*Teikyo University School of Medicine, Emeritus Professor*)

When a muscle is stimulated with two successive stimuli at short intervals, the second twitch starts from the falling phase of the first twitch. A.V. Hill, who was regarded as a king by physiologists, estimated the amplitude of the second twitch by algebraically subtracting the first twitch from the summated twitches. As a result, the second twitch was larger than the first one at appropriate stimulus interval. I felt that his estimation was wrong, and in 1963 started my first experiment using isolated single crayfish muscle fibers as material. During the course of this experiment, I found that, when a muscle fiber was stimulated with transversely applied currents, contraction first started under depolarized fiber membrane, and then spread towards the hyperpolarized fiber membrane. Then, I made another experiment, in which the fiber was locally depolarized by passing outward current though a micropipette in contact with the fiber surface, and found ring-shaped local contractions taking place in a nearly all-or-none manner. This work attracted attention of many investigators, including A.F. Huxley, and I was recognized as a prominent muscle physiologist in Japan.
My last experiment is concerned with electron microscopic visualization and recording of myosin head movement, coupled with ATP hydrolysis. My success in this experiment is unduly ignored by physiologists and biophysicists, but highly evaluated by material scientists and a limited number of investigators, including A.F. Huxley and H.E. Huxley.

### [PS01-02]
### History and future prospects of pain physiology

*Natsu Koyama[1] ([1]*Shiga University of Medical Science*)

Learning the history of physiology maybe very interesting, but core curriculum of physiology cannot afford to mention history of physiology. I would like to introduce relation between the history of physiology and the history of pain treatment/research. One topic is that application of electricity date back to antiquity. It is well known that the discovery of Volta's battery, which mimicked the electric organ of the torpedo fish, led to the discovery of electrophysiology, but ancient Roman physician had discovered that electrical impulses emitted from torpedo fish was used for treatment of gout and headache. Jean-Paul Marat performed electrotherapy with Leyden jars and electrostatic generator, before French revolution. Today, magnetic stimulation as well as electrical stimulation is used for treatment of intractable pain. Another topic is about mysterious pain. Sensations usually result that sensory receptors are triggered by the *adequate stimulus*, but neuropathic pain and phantom limb pain are not involved in nociceptors. The words "phantom pain" and "causalgia" were named by Silas Weir Mitchell, who treated the burning pain of soldiers with nerve injury or amputation during the American Civil War. Mitchell was a physiologist and novelist. It is also interesting that Mitchell has developed different therapies between female and male neurasthenia after war.

### Planning Symposium 2

### [PS02]
### 【Committee for Promotion of Gender Equality】
### What do you think of your second career?
### - Hints for developing a new career

March 16(Wed), 12:30 - 13:30, Room J

### [PS02-01]
### Everything leads to a bright future

*Yuko Sekino[1] ([1]*Graduate School of Pharmaceutical Sciences, the University of Tokyo*)

It has been 42 years since I started my research life. The motivation for my research was "to understand the brain", and I was particularly interested in the mechanism of memory. I am 65 years old and will retire in March of this year. I was able to satisfy my curiosity, but I am still dissatisfied. I want to use my research results, experience, and knowledge for the benefit of society. After retirement, I would like to promote the social implementation of our research results. By the way, I think that what everyone is interested in at this symposium is how I survived as a researcher. I almost gave up many times. But I always tell myself, "You need big hope to make small hope come true". It rarely goes as expected. We also know that if we get stuck, it's time for something new to happen. Experience is a treasure. Any little thing can be the source of your life. A lot of unplanned things have happened in my life, but now all the little things are connected and nothing is wasted. Therefore, it can be said that "everything leads to a bright future".

### [PS02-02]
### Seamless transition from academia to business world to enjoy the second half of life.

*Tomoaki Shirao[1] ([1]*AlzMed, Inc.*)

After graduating from medical school, I went on to graduate school, where I received my Ph.D. in 1984 for my discovery of "drebrin", a protein that transiently appears as the brain develops. After I got the Ph.D., I fortunately continued all the life to conduct basic research at the medical school for almost 40 years until I retired from the university. At the time, I believed that if I developed my research out of curiosity, the results would one day be developed by future generations of researchers and lead to the happiness of mankind. Certainly, drebrin research has spread widely throughout the world, and people have been elucidating new facts one after another. However, I was concerned about the fact that these works were still in the realm of academic research and did not result in the social implementation and contribution to the well-being of humanity. The results of drebrin study showed the possibility of developing a new and completely different drug for Alzheimer's disease, and I tried to persuade the industry to proceed this way, but it was not that easy. I realized that the fact that drebrin drug discovery was not accepted by the present industry meant that there was a so-called "blue ocean" out there, and I decided to start my own business in the latter half of my life and aim to implement the research results of academia in society. I'd like to introduce you to the joys of jumping into a new world after retirement.

### [PS02-03]
### What kinds of supports are needed to facilitate participation in the annual meetings?

*Misa Shimuta[1] ([1]*The Jikei University of School of Medicine*)

Last January, as the Committee for Promotion of Gender Equality, we conducted a survey on "the reasons for giving up participation in the Annual Meeting and the supports needed for them" to make a support plan for society members to attend annual meetings. This survey revealed that childcare was one of the most frequent reasons the members had to give up participation. In this presentation, we will show the analyzed result of this survey and provide information on the life event support created based on members' voices.

### [PS02-04]
### Support activities in the Physiological Society of Japan that transcend gender and generation

*Hiroko Izumi-Nakaseko[1] ([1]*Dept. Pharmacol., Faculty Med., Toho Univ.*)

In the Physiological Society of Japan, the Committees of the Young Physiologists, the Women in Physiology of Japan, and the Committee for Promotion of Gender Equality are currently providing supports for the members of the society to be active in the field of physiology. In this symposium, we will introduce the activities of these Committees and the results of their support so far.

### Planning Symposium 3

### [PS03]
### Messages from the Pioneers in Electrophysiology Research in Japan to Young Physiologists

March 16(Wed), 15:45 - 17:45, Room E

### [PS03-01]
### Electrophysiology: fun and future potentials of ion channel study

*Masahiro Sokabe[1] ([1]*Nagoya University Graduate School of Medicine*)

Electric signals are fundamental in the regulation of our circulation and nervous systems, without which we cannot live even for a minute. Most attractive feature of electrophysiology is that it enables us to make a quantitative measurement of bioelectric signals with a quite high time resolution, even at the single ion channel level, telling us a physical basis of life. We should also keep in mind that quantitative stimulation is indispensable for a physical characterization of the targeted channel. As an example I will tell you how to apply a quantitative mechanical stimulus to mechanosensitive ion channels by using a state of the art multimodal microscopy. This technology can also be applied for analyzing the downstream intra- and inter-cellular signaling through the measurements of Ca-transient and ATP release, respectively. Ion channel study involves other promising potentials: combination with molecular simulations will soon achieve an ultimate understanding of structure-function of ion channels at the atomic level, an example for channel mechanogating will be presented. Furthermore, by introducing photo-activated ion channels into targeted organs/tissues we would be able to treat a variety of diseases.

### [PS03-02]
### Fascinated by studies on synaptic transmission

*Sumiko Mochida[1] ([1]*Tokyo Medical Univ.*)

"Study of synaptic transmission" was the theme through out of my research life. When I started it in 1975, sympathetic ganglia were studied as a model of brain, using Air-gap, Sucrose-gap or a micro-pipette. In 1980s, it was thought that culture of adult neurons is impossible, but I cultured adult sympathetic neurons and studied the intracellular signal transduction.

In 1988-1990, I studied mechanism of neurotoxins which block neurotransmitter release, injecting their fragments or the mRNA into presynaptic neurons of *Aplysia*, and analyzing postsynaptic currents with the voltage-clamp method.

In 1990, I started a new approach to study neurotransmitter release from a mammalian neuron. I made synapses between sympathetic neurons in a culture dish, injected blockers into presynaptic neurons, and analyzed the effect on postsynaptic potentials. In 1990s, many proteins were identified in presynaptic neurons. Targets of the neurotoxins were release machinery proteins. Since then, to know protein function in neurotransmitter release, peptides, proteins or DNAs were injected into presynaptic neurons.

In this symposium, I would introduce fossilized methods and materials of electrophysiology.

### [PS03-03]
### My experiments of Electrophysiology in the past and present

*Harunori Ohmori[1] ([1]*Kyoto University*)

I learned the Hodgkin-Huxley theory by reading a review article published in the Scientific American for the first time in a university seminar (around 1968). The phenomenon of membrane excitability was totally beyond my understanding at that time, but it left a big question on neurons in my mind. I have systematically studied electrophysiology of ion channels and neurons in the graduate school and later in US. After coming back from US in 1982, I focused on studies of mechano-electrical transducer (MET) mechanism of the hair cell. In this symposium, I will talk briefly about the electrophysiological experiments in my early career, and some fundamental experiments on the MET channel. I will extend the talk to a recent story on the localization of the MET channel. I will skip other works on the brainstem auditory nuclei. Instead, I will talk my recent study on the auditory cortex, which I have conducted by using a technology of patch-electrode photometry after retirement from a university.

### Planning Symposium 4

### [PS04]
### Regulation of neural function by nicotinic acetylcholine receptors

March 16(Wed), 15:45 - 17:45, Room F

### [PS04-01]
### Roles of nicotinic acetylcholine receptors in modulation of neural circuit function in the insular cortex

*Toyoda Hiroki[1] ([1]*Osaka University Graduate School of Dentistry*)

Nicotinic ACh receptors (nAChRs) play key roles in neuonal circuit development, drug addiction and cognitive functions and behaviors including attention, learning, memory and motivation. The insular cortex is an important brain region associated with sensory perception, self-awareness, cognitive function, motor control, and drug addiction. The activation of nAChRs in the insular cortex modulates its microcircuits to perform various functions. Therefore, it is crucial to understand the cholinergic modulation of microcircuits in the insular cortex. We examined which type of neurons express functional nAChRs in layer III, V, and VI of the mouse insular cortex and how activation of nAChRs modulates synaptic transmission and plasticity in the layer III, V, and VI pyramidal cells. We found that activation of nAChRs layer-specifically modulates synaptic transmission and plasticity in the mouse insular cortex. These findings are critical to understand the modulating effects of ACh or nicotine on physiological and pathophysiological functions associated with the insular cortex.

### [PS04-02]
### Nicotinic regulation of sensory filtering in mouse auditory cortex

*Hideki Derek Kawai[1] ([1]*Soka University*)

Systemic nicotine exposure regulates sound information processing in the central auditory pathway. In the primary auditory cortex (A1), where neurons are spatially arranged tonotopically from low to high frequency, nicotine enhances the synaptic activities evoked by tone stimuli of the specific frequency with the lowest sound intensity called characteristic frequency (CF), while it suppresses the synaptic activities evoked by the tone frequency that is two or more octaves above or below CF (non-CF). The molecular, cellular, and circuit mechanisms underlying this nicotinic regulation of sensory filtering are unclear. Experimental evidence indicates that the enhancement of CF tone-evoked responses is carried out by nicotinic regulation along the thalamocortical pathway. Meanwhile, the nicotinic suppression of non-CF tone responses may depend on sensory filtering along the auditory pathway prior to A1, though nicotinic regulation of cortical neurons could also contribute to it. Our recent studies indicate that biochemical and functional alterations of glutamatergic synapses in A1 contribute to the nicotinic regulation of sensory filtering.

### [PS04-03]
### Physiological functions of the cholinergic system in the basal ganglia

*Masami Miura[1], Ritsuko Inoue[1] ([1]*Tokyo Metropolitan Institute of Gerontology, Aging Neuroscience*)

The striatum is the input stage of the basal ganglia, and its physiological functions are strongly regulated by acetylcholine as well as dopamine. Acetylcholine is derived from intrinsic cholinergic interneurons in the striatum, whereas dopamine is derived from midbrain dopaminergic neurons. In this talk, we will introduce the nicotinic receptormediated physiological function of cholinergic interneurons and its relation to the compartmental structure of the striatum.
The striatum does not form a layered or columnar structure, so at first glance it appears to have no anatomical structure. Still there are two distinct compartments: the striosomes (or patches) and the surrounding matrix. Cholinergic interneurons are found predominantly around the striosomes, while cholinergic innervation is relatively dense in the matrix. The distribution of cholinergic neurons is not uniform along the anterior-posterior axis but is denser rostrally. In addition, in the caudal striatum, there is a gradient of their distribution along the mediolateral axis. Since these anatomical features of the cholinergic innervation of the striatum lead to local differences in the action of nicotinic drugs, they would lead to different modulatory effects on regional functions of the striatal compartments.

### [PS04-04]
### Facilitative effects of nicotinic acetylcholine receptors on synaptic plasticity in the hippocampus

*Yoshihiko Yamazaki[1], Hiroki Fujiwara[1], Jun-Ichi Goto[1], Satoshi Fujii[1]
([1]*Department of Physiology, Yamagata University School of Medicine*)

Acute and chronic nicotine exposure lowers the threshold for long-term potentiation (LTP) induction in the rat hippocampal CA1 region. In this presentation, we reveal the potential mechanisms underlying the effects of nicotine. Nicotinic acetylcholine receptors are abundantly expressed in inhibitory interneurons and they exhibit characteristic responses to nicotinic stimulation depending on the subtypes, e.g., rapid desensitization or continuous activation. Acute application of nicotine modifies inhibitory synaptic transmission and increases the NMDA receptor (NMDAR) activity on the pyramidal cells (which is a key component of synaptic LTP induction) in two different ways. In addition, nicotine enhances NMDAR-mediated responses through the activation of muscarinic receptors. Chronic *in vivo* nicotine exposure induces the enhancement of NMDAR responses via a mechanism that occludes muscarinic receptor-mediated potentiation. The GluN2B subunit is the target of nicotinic receptor activation during *in vivo* nicotine exposure, and the Src signaling pathway is involved in the enhancement of GluN2B-NMDAR responses.
Thus, nicotine regulates the balance of excitatory and inhibitory transmission in the hippocampus and facilitates LTP induction in diverse ways.

### Planning Symposium 5

### [PS05] 【Committee for Research Ethics】 Education Seminar

March 17(Thu), 8:30 - 9:30, Room D

**[PS05-01]**
**The ethics and laws for human and animal researches -The special meaning of them in Japan-.**
*Norio Higuchi[1] ([1]*Law Department, Musashino University*)

### Planning Symposium 6

### [PS06]
### 【Committee for Editorial Board of the Journal of Physiological Sciences】
### How to prepare a good physiology paper

March 17(Thu), 8:30 - 10:30, Room G

### [PS06-01]
### Current status of the Journal of physiological sciences (JPS)

*Motohiko Sato[1] ([1]*Physiology, Aichi Medical University*)

The Journal of physiological sciences (JPS) is the official journal of Japanese Physiological Society. JPS was first issued in 1950, since then, JPS has been contributing to the development of research in the physiological fields. JPS covers over all fields of physiology including adaptation and environment, autonomic nervous function, biophysics, cell sensors and signaling, central nervous system and brain sciences, endocrinology and metabolism, excitable membranes and neural cell physiology, exercise physiology, gastrointestinal and kidney physiology, heart and circulatory physiology, molecular and cellular physiology, muscle physiology, physiome/systems biology, respiration physiology and senses. In 2018, all of JPS articles were published online, and 2 years later, JPS restarted as an open access journal. JPS published 53 papers submitted from 11 countries in 2020. In this section, the current status of JPS will be reported including the acceptance rate, citations and impact factor etc. Standard reviewing process and tips for smooth entry to reviewing process will also be reviewed.

### [PS06-02]
### how publishing a work improves the quality and impact of the presentation

*Sharona Gordon[1] ([1]*Biological Physics, Structure and Design, University of Washington*)

The publishing world is facing disruptions, with an increase in the number of journals and preprint servers offering unprecedented choices for authors. In this talk, Professor Gordon will discuss how publishing your work can take advantage of the numerous choices to improve the quality and impact of your presentation. Physiology papers can achieve both rigor of data and conclusions and be of broad interest without sacrificing the need to publish in highly regarded journals. This talk will discuss how to balance the value of the work with the short-term and long-term career goals of the authors.

### [PS06-03]
### Let's prepare a great physiology paper

*Makoto Tominaga[1,2] ([1] *Div. Cell Signaling, National Institute for Physiological Sciences,* [2] *Thermal Biology Group, Exploratory Research Center on Life and Living Systems*)

I am working as an editor in chief of the official journal of The Physiological Society of Japan, The Journal of Physiological Sciences, and have chances to read a lot of manuscripts submitted to the journal. Through the experience, I would like to discuss how to prepare a great physiology paper with two experts, Prof. Motohiko Sato who is working as editors for several international journals and has experience to teach students as an associate professor in the united states, and Prof Sharona Gordon who was working as a chief editor of Journal of General Physiology published by Rockefeller University Press.

### Planning Symposium 7

### [PS07]
### 【Committee for Women in Physiology of Japan】 Neural mechanism for surviving a stressed society

March 17(Thu), 16:00 - 18:00, Room C

### [PS07-01]
### Neural mechanisms of the link between social stress and aggression

*Aki Takahashi[1] ([1]*Faculty of Human Sciences, University of Tsukuba*)

Social stress can lead to the development of psychological problems ranging from exaggerated anxiety and depression to antisocial and violence-related behaviors. Violence incidents are often triggered by social instigation or frustration. In animal models, a brief encounter with a potential rival induces aggressive arousal which increases subsequent aggressive behavior, phenomena referred to as social instigation (or provocation). Previously, we have shown that the level of excitatory glutamate input into the dorsal raphe nucleus (DRN) was increased during social instigation-heightened aggression in the male mouse. The DRN receives glutamatergic projections from the lateral habenula (LHb), the brain area that is implicated in negative emotion and stress. By using optogenetis and DREADD techniques, we found that the LHb neurons that project to the DRN (LHb-DRN projection) are involved in the escalation of aggressive behavior induced by social instigation. In this talk, the role of LHb-DRN projection in terms of the link between social stress and aggression will be discussed.

### [PS07-02]
### The roles of inflammation in repeated stress-induced behavioral changes

*Shiho Kitaoka[1] ([1]*Hyogo College of Medicine*)

Social and environmental stress activates the sympathetic nervous and neuroendocrine systems as a survival mechanism. However, prolonged or excessive stress induces behavioral changes such as social avoidance and elevated anxiety. We previously reported that acute stress activates the mesocortical dopaminergic pathway to suppress the induction of social avoidance. Repeated stress suppresses the mesocortical dopaminergic pathway to induce social avoidance. Prostaglandin E2, an inflammatory molecule, suppressed this pathway. These findings proposed the involvement of inflammation in stress-induced behavioral changes. Indeed, repeated stress induced neuroinflammation originating from microglia in the medial prefrontal cortex, leading to social avoidance. Additionally, we found that repeated stress increased the number of leukocytes in the anterior cingulate cortex. Consistent with this, repeated stress increased monocyte and neutrophil in the bone marrow and the blood. In this symposium, I'd like to introduce our findings regarding inflammation in the central nervous system and peripheral tissue.

### [PS07-03]
### Exploring molecular pathways involved in central amygdaladependent control of emotional behaviors

*Sayaka Takemoto-Kimura[1] ([1]*Nagoya Univ.*)

Emotional and social behavior disabilities are exhibited in multiple stress-associated psychiatric disorders and it is important to understand the specific machinery governing these behaviors at the subnucleus level in critical brain regions such as the amygdala. Within the amygdala, the basolateral amygdala (BLA)-central nucleus of amygdala (CeA) circuit is known to mediate fear and anxiety responses. Furthermore, the bed nucleus of the stria terminalis (BNST) is also included in the central extended amygdala together with the CeA, comprising a functional-anatomical macrosystem. Both regions have complex subnuclear organizations. Recently, we revealed differential engagement of distinct subnuclei of the central extended amygdala, critical for regulation of fear- and anxietyrelated behaviors in mice. In addition, the amygdala is crucial for social processing. To investigate the molecular basis of CeA-dependent social and emotional behaviors, we focused on a $Ca^{2+}$-dependent phosphorylation pathway, which is highly expressed in the CeA. Genetic and virus-mediated molecular manipulations of the kinase induced behavioral anomalies in emotional and social behavioral tasks, suggesting the kinase constitutes a novel amygdala $Ca^{2+}$-dependent signaling pathway that controls behavioral modifications triggered in response to external social environment.

### [PS07-04]
### Circulating apelin modulates central nervous system regeneration

*Rieko Muramatsu[1] ([1]*National Center of Neurology and Psychiatry*)

Age-related regeneration failure in the central nervous system can occur as a result of a decline in remyelination efficacy. The responsiveness of myelin-forming cells to signals for remyelination is affected by aging-related epigenetic modification; however, the molecular mechanism is not fully clarified. In the present study, we report that the apelin receptor (APJ) mediates remyelination efficiency with age. APJ expression in myelin-forming cells is correlated with age-associated changes in remyelination efficiency, and the activation of APJ promotes remyelination through the translocation of myelin regulatory factor. APJ signaling activation promoted remyelination in both aged mice with toxin-induced demyelination and mice with experimental autoimmune encephalomyelitis. In human cells, APJ activation enhanced the expression of remyelination markers. Impaired oligodendrocyte function in aged animals can be reversibly reactivated; thus, the results demonstrate that dysfunction of the apelin.APJ system mediates remyelination failure in aged animals, and that their myelinating function can be reactivated by APJ activation.

### Planning Symposium 8

### [PS08]
### 【Committee for Promotion of Physiome and Systems Biology】
### Physiological and social systems breakdown

March 17(Thu), 16:00 - 18:00, Room D

### [PS08-01]
**Diseases as Catastrophes of Biological Systems and Detection of Early Warning Signals**

*Kazuyuki Aihara[1] ([1]*University of Tokyo*)

I will talk about our DNB(Dynamical Network Biomarkers) theory and its applications toward preventive precision medicine. The DNB theory can discover pre-disease states of some acute and chronic diseases by detecting early warning signals of imminent diseases leading to catastrophes of biological systems. Further, I will also discuss a possibility to extend DNB to DNM(Dynamical Network Markers) by detecting early warning signals for imminent catastrophes of complex systems in general.

### [PS08-02]
**Data analysis and modeling of systemic failure phenomena of complex systems**

*Misako Takayasu[1] ([1]*Tokyo Institute of Technology, IIR*)

In this presentation we will review systemic failure phenomena of complex systems from the viewpoint of data analysis and mathematical modeling. Complex systems in the real world produce various functions through their sophisticated structures. Such systems grow over a long period of time interacting with the outside world, but often lose their normal activity in a very short time with no or hard-to-detect symptoms. Bubbles and crashes in financial markets, chain failure of business firms and stagnation of traffic logistics due to disasters are typical examples.
The first step of the study is to extract empirical laws from noisy raw data of a phenomenon. We apply various basic statistical methods to observe statistical properties such as distributions and correlations. After establishing basic properties we construct a mathematical model that can reproduce those properties. In order to describe systemic failure we need to introduce a nonlinear model which shows a phase transition behavior, that is, the model's property changes qualitatively for continuous change of parameters. As a real world example, we introduce our model of the transaction network consisted of about 1 million business firms in Japan. Money flow among firms can be estimated from this model, and we can simulate chain failure of firms. By changing parameters of the model we find a condition that failure of a firm can expand to the whole system. Also, based on this model, we can estimate the economic effect of an imaginary disaster of any size at any place. As shown in this example, modeling is the key step of study on systemic failure as we can find hidden properties of the system through numerical simulation of the mathematical model.

### [PS08-03]
**Breakups of excitation propagation in the heart: cardiac arrhythmias**

*Kunichika Tsumoto[1] ([1]*Kanazawa Medical University*)

The heart is a huge and hierarchical system consisting of molecules, cells, tissues, and organs, and each hierarchy individually forms an extremely elaborate system. Collapse events occurring in the heart first remind us of ischemic heart diseases. The coronary arteries are occluded by thrombosis or vasospasm and a myocardial infarction occurs, which not only damages myocardial tissues but also increases the risk of sudden cardiac death. Then, the damaged heart progresses into a state of heart failure. This can be seen as a state in which the heart has already collapsed. On the one hand, cardiac arrhythmias can also be considered a system failure, meaning that the coordinated propagation of electrical excitation is impaired. It is believed that spiral reentrant excitation waves rotating within the ventricle and their random breakup are involved in fatal arrhythmias such as ventricular tachycardia (VT) and ventricular fibrillation (VF). We have theoretically analyzed the developmental mechanisms of triggered activities, e.g., afterdepolarizations in the cellular level and phase-2 reentry in the multi-cellular level, which have been considered to become the seed that causes degenerations into VT and VF in long QT syndrome and Brugada syndrome. In addition, how triggered activities as the seed of arrhythmias link to the onset of arrhythmias is also examined recently. In this symposium, we would like to briefly review the current positions of system electrophysiology in the heart and introduce our recent results.

### [PS08-04]
**Early warning detection of disease onset and pathological transitions by intensive longitudinal biomedical data**

*Toru Nakamura[1] ([1]*Osaka University*)

Recent advances in biomedical sensing technologies based on IoT (Internet of Things) have made it possible to measure high-quality, very long-term, continuous biomedical signals, referred to as biomedical intensive longitudinal data (ILD), in daily life. To link ILD to clinical applications, such as early detection of disease onset and/or disease prevention, the development of a new data mining framework is thought to be important. In this talk, we briefly introduce two applications of biomedical ILD. The first is an early warning detection of pathological changes in a patient with bipolar disorder based on alterations of dynamical properties in spontaneous physical activity (SPA) continuously measured in daily life (> 1year). The second shows a potential to detect disease onset of alcoholism; the analysis of ILD of drinking behavior and SPA recorded from an animal model of alcohol addiction showed a transition into addictive behavior preceded by early-warning signals, such as instability of drinking patterns and circadian rhythms, and a resultant incase in ultradian rhythms. The both provide an adaptable framework for examining disease dynamics and early-warning signals in the biomedical field.

### Planning Symposium 9

### [PS09]
### EEG/LFP oscillations underlying higher brain functions

March 17(Thu), 16:00 - 18:00, Room E

### [PS09-01]
**Neural oscillations: Toward a unified understanding of "communication"**

*Hiroaki Mizuhara[1] ([1]*Kyoto University*)

Neuronal oscillations are observed as local field potential (LFP) and electroencephalography (EEG) when membrane potential fluctuations are synchronized in a neural population. The phase synchronization of neural oscillations has been reported to realize not only information communication between neural populations in the local brain, but also information communication across brain regions. Furthermore, it appears that phase synchronization of neural oscillations could be a common principle that can explain not only information communication within the brain, but also cross-brain situations, i.e., our human communication. I will introduce some examples of research for a unified interpretation of these information communications. Based on those examples, I will discuss how it is possible to describe the communication within and between brains in a unified framework.

### [PS09-02]
**Unraveling the functional role of transient oscillatory synchronization in human brain activity**

*Keiichi Kitajo[1] ([1]*National Institute for Physiological Sciences*)

In this presentation, I propose a hypothesis regarding the role of the transient synchronization of oscillatory brain activity in information processing in the human brain. First, I introduce the theoretical background of transient synchronization in mathematical models such as metastable chimera states of synchrony. Second, I introduce experimental data demonstrating that the human brain exhibits transient large-scale synchronization in electroencephalographic (EEG) activity. I also provide empirical evidence that the transient nature of neural synchrony is associated with individual differences in psychological traits and brain disorders. Finally, I propose a hypothesis on the role of transient synchronization in large-scale neural activity. Specifically, I hypothesize that transient synchronization of neural activity is mediating the flexible networking among distant brain areas associated with information processing in various brain functions. I speculate that individual differences in human brain functions are associated with the individual feature of transient neural dynamics in the brain.

### [PS09-03]
**Traveling waves instantaneously coordinate the large-scale cortical network: A connectome-based model simulation**

*Naoyuki Sato[1] ([1]*Future University Hakodate*)

Neural synchronization contributes to a variety of functions, including perceptual binding, information transfer control, and synaptic plasticity. Recent human studies using electrocorticography have demonstrated that alpha and theta band oscillations form traveling waves on the cortical surface. According to neural synchronization theories, the cortical traveling waves apparently group local cortical regions and sequence them by phase synchronization, although these contributions have not been assessed. This study aimed to evaluate the functional contribution of traveling waves using connectome-based network modeling. In the simulation, we observed stable traveling waves on the entire cortical surface, wherein local regions with similar phases were temporally grouped by the phase. Interestingly, the topographical pattern of these phases was substantially correlated with the empirically observed resting-state networks. Simultaneously, individual local regions were instantaneously sequenced by their internal frequencies. This phase configuration was thought to produce unidirectional communication between the arbitrary paired regions. In conclusion, the cortical traveling waves were functional in the grouping and sequencing of cortical regions, which would coordinate large-scale networks in the cerebral cortex.

### [PS09-04]
**A Systematic Metadata Generation Scheme for the Hypothesis-Driven Inference System Contributing to the Advancement of Neuroinformatics Databases**

Manu Shrivastava[1], Kota Seri[1], Kosei Shibata[1], *Hiroaki Wagatsuma[1,2]
([1]*Kyushu Institute of Technology*, [2]*RIKEN-CBS*)

With respect to classical hypothesis-driven approaches for scientific findings, expectations to data-driven approaches are increasing as ways to find a hidden relationship among data. However, the simple data-driven approach is not effective without appropriate annotation of data, which usually requires human operations according to expert knowledge and abilities. A hybrid, or fusion method of hypothesis- and data-driven approaches is highly important, called data-driven ontology discovery scheme. As a new computer-based hypothesis-driven approaches will be developed as a linkage system for multi-dimensional data for a meaningful reconstruction of what the whole structure represents as a scientific evidence, which not only contributes to the design of the standard data format to connect multiple datasets with different resolutions in time and spatial domains by effective indexing methods, but also a data searching technology across multiple databases by the establishment of hierarchical metadata descriptions. We have focused on a systematic metadata sharing scheme based on the NIX/odML standard data format was developed. A recent topic is a systematic analysis tool for the expert knowledge extraction was proposed by using ontology and semantic rules as OWL/SWRL. Natural language sentences from a public publication contains a logical structure of the publication, and it might be possible to describe in the first-order logic in the OWL/SWRL format. Thus, an advanced semantic-web technology realizes data-driven ontology discovery by solving indexing problem and providing effective annotations which can be mapped on the brain atlas with 3-D reference models.

### [PS09-05]
**Local field potential modulations in the lateral prefrontal cortex during behavioral planning**

*Kazuhiro Sakamoto[1,2], Norihiko Kawaguchi[2], Hajime Mushiake[2]
([1]*Faculty of Medicine, Tohoku Medical and Pharmaceutical University*, [2]*Tohoku University School of Medicine*)

The lateral prefrontal cortex (lPFC) plays an important role in executive function and behavioral planning by adaptively storing varieties of information as working memory. Neural mechanisms associated with local field potentials (LFPs) may underlie the adaptive properties of the lPFC. Here, we analyzed how the LFPs recorded from the monkey lPFC are modulated by the crucial factors of a shape manipulation task. In this task, the test shape is transformed by manipulating a lever to match the size and orientation of the sample shape. The subject is required to temporarily memorize the rules such as the armmovement- manipulation relationship and the sample shape to generate the sequential behavior of operations. We found that the transformed shape in the sample period strongly affected the theta oscillations in the delay period on the ventral side, and the armmanipulation assignment influenced the low gamma components in the sample period on the dorsal side. These findings suggest that area- and frequency-selective LFP modulations are involved in recruiting different task relevant information in the lPFC for adaptation to ever-changing environments.

### Planning Symposium 10

### [PS10]
### 【Committee for 100th anniversary】 What is the 100th anniversary of Physiological Society of Japan for us?

March 18(Fri), 8:30 - 10:30, Room D

### [PS10-01]
### How can we maintain our progress as PSJ?

*Yoshihiro Ishikawa[1] ([1]*Yokohama City University School of Medicine*)

In 1922, soon after the general assembly of the Japanese Association of Medical Sciences (JAMS) in Kyoto, physiologists from several universities decided to establish the Physiological Society of Japan (PSJ), which is independent from JAMS. It was during the era of founding new medical schools in Japan, and there were 19 medical schools by 1922. On the 10th of October 1922, they had the first scientific meeting of PSJ in Tokyo. In that meeting, there were only 37 presentations because there were not many physiologists. Fifty years later at the PSJ meeting at Fukuoka in 1973, the number of presentations increased to 405. Today, we have nearly 1000 presentations at each meeting and the number of our Society members has increased to 2700. This is a great success story for a medical society, which will celebrate its 100th anniversary in 2022. At the same time, we are facing a new dilemma; how to maintain our progress during the next century. The number of medical societies within JAMS has increased to 138: we hold the membership number of "3", as we are one of the oldest. However, this does not guarantee that we can continue growing as scientific society. We need to expand and deepen our activity and membership to new fields, such as physiology education. We appreciate your participation and considering our future together in this symposium.

### [PS10-02]
### What do we aim with the 100th Annual Meeting of the Japan Physiological Society?

*Tadashi Isa[1] ([1]*Kyoto University, Graduate School of Medicine*)

In July 2022, the Physiological Society of Japan (PSJ) will celebrate the centenary, and the annual meeting in March 2023 will be the 100th Memorial meeting. We, as the organizing committee of the 100th Annual Meeting of PSJ, decided the theme of the meeting to be "Homeostasis for Sustainability; Toward the next century of physiological sciences." The key concept of the modern physiology has been "homeos tasis" which was established by Claud Bernard in the 19th century and Walter Cannon in the early 20th century. Homeostasis meant that the internal environment of the animal's body is maintained stable independent of the external environment.

In the 100th meeting, we wish to look back the history of the physiology during the last 100 years and look to its next century. For that, we may need to view not only the homeostasis of individual subjects, but also homeostasis of the human society, global environment or even that of the universe, for their sustainability. Taking the pportunity of discussion in the 99th meeting, I would like to argue on the concept of "Homeostasis for Sustainability" with the participants of the symposium toward the 100th meeting.

### [PS10-03]
### A Congratulatory Message from APS

*Jennifer Pollock[1] ([1]*American Physiological Society*)

Many congratulations on your upcoming centennial anniversary. The American Physiological Society was founded in 1887 with only 28 members. Twenty one scientists were graduates of medical schools, but only 12 had studied in institutions that had a professor of physiology. Thus, alike the Physiological Society of Japan, our Society was founded at a time when very few physiological laboratories existed in the country and there were few investigators. Our Society, alike your Society, was one of the earliest national disciplinary societies in the sciences, the first society in the biomedical sciences, and likely the first to require its members to publish original research. The stated object of the Society has been to promote the advancement of physiology and to facilitate discourse among American physiologists. There was a conscious effort to ensure representation of all areas within physiology, encompassing topics as diverse as neurology, psychology, ophthalmology, pathology and therapeutics, as well as plant physiology and animal biology. We wish to grow together with you and all other physiological societies in the world for the next century.

### Planning Symposium 11

### [PS11]
### Laboratory crisis management for safety research

March 18(Fri), 8:30 - 10:30, Room E

### [PS11-01]
### Crisis managements in the laboratory and student education - Great East Japan Earthquake and Novel Coronavirus Infection -

*Naoto Ishii[1] ([1] *Department of Microbiology and Immunology, Tohoku University Graduate School of Medicine*)

I have been hosting a laboratory of immunology since 2009, and have also served as the chair of the education committee at Tohoku University School of Medicine since 2015. The Great East Japan Earthquake in 2011 forced us to suspend our research for various reasons, including the loss of a large number of mice kept in our laboratory. Furthermore, the novel coronavirus infection spread after 2019 greatly disrupted not only research but also student education, especially practical training, and we struggled to cope with it. In this talk, I would like to introduce and discuss our various experiences of disaster response and crisis management in our laboratory and medical school during the two major disasters.

### [PS11-02]
### Academic harassments and crisis management

*Ken-ichi Honma[1] ([1] *Hokkaido University Graduate School of Medicine*)

Academic harassments crash not only faculty members but also a laboratory itself. I will report two cases of such crashes which I experienced while I was a dean of the Graduated School of Medicine in Hokkaido University. They happened in 2005 to 2008, the time when the National University became Corporation Aggregate. The one case is a typical example of the so-called cultural discrepancy and the other is a typical example of generation gap. However, in both cases, the laboratory activities were substantially disturbed for a long period and enoumus efforts should have been paied for the restration. To prevent or to minimize such struggles, we should realize the underlying changes of adademic society.

### [PS11-03]
### Crisis Management to Protect Tohoku Medical and Pharmaceutical University from Natural Disasters

*Motoaki Takayanagi[1] ([1] *Tohoku Medical and Pharmaceutical University*)

Considering major earthquakes off the coast of Miyagi occur in a cycle of approximately 30 years, all the buildings of the Tohoku Medical and Pharmaceutical University were designed to be earthquake-resistant. Accordingly, damage was successfully minimized when the Great East Japan Earthquake occurred in 2011.
We are proud of our strength on the construction side, however, we identified several weaknesses on the logistics side: delays in setting up headquarters for disaster control; prolonged confirmation of the safety of students, faculty, and staff; and complications dealing with students and residents in the neighborhood seeking shelter on campus. After the 2011 earthquake, we created a crisis management committee to enhance our crisis management system. The formation of the committee allowed us to implement a more advanced cross-sectoral decision-making process.
Construction of the newly accredited medical school in the Fukumuro region was completed in 2016. During construction, we strove to make the campus resistant to earthquakes, storms, and floods because it is located in a low-lying region facing the Nanakitada River. Concerning crisis management of our new university hospital, we came up with solutions to continue uninterrupted medical care for patients during a natural disaster. I will introduce further details in my presentation.

### [PS11-04]
### Changeable and unchangeable mind in physiological research found with COVID-19

*Toshio Ohhashi[1] ([1] *Shinshu University School of Medicine, Department of Innovation of Medical and Health Sciences Research*)

I have conducted the research for microcirculation and lymph circulation during 40 years in Department of Physiology in Shinshu University School of Medicine. After the retirement, I have continued the physiological research more than 7 years in the established Department of Innovation of Medical and Health Sciences Research with the donation of BOURBON Co. Ltd. in Kashiwazaki, Niigata and Aizawa hospital, Matsumoto, Nagano. I have also promoted The Japanese Society of Lympholology as President. With the experience with COVID-19, I have reconsidered the changeable and unchangeable mind in physiological research from points of view; (1) presentation of research, (2) continuation of research, (3) keeping research group, (4) utilizing research time, (5) getting research grant, (6) utilizing research fruit, (7) international communication with research collaborator, etc. In this symposium, I will present my opinion and discussed the mind with the audience in the symposium.

### Planning Symposium 12

### [PS12]
### RNA molecular physiology in COVID-19 research

March 18(Fri), 8:30 - 10:30, Room F

### [PS12-01]
### 5' cap in mRNA vaccines

*Yasuhiro Furuichi[1,2] ([1]*Niigata Phamaceutical Univ.* , [2]*GF Mille Corporation Co., Ltd.*)

Cap structures of the type m7GpppNm are present widely at the 5' ends of nearly all eukaryotic cellular and viral messenger RNAs (mRNAs). The caps were found by authors in 1975 at National Institute of Genetics in Mishima/Japan and Roche Institute of Molecular Biology at Nutley NJ/USA during studying genomic structure of insect virus. The major biological functions of cap were later identified to stabilize mRNA molecules by protection against 5' exonucleolytic attack and to enhance protein synthesis by binding to ribosomes. This fundamental information of cap triggered various other findings in 1970s and have also been very helpful to understand the mechanism underlying nuclear RNA splicing, battles between cells and various viruses, and many other problems involved in the cascade reactions of gene expression. Strikingly, the cap is now used as essential chemical component of Covid-19 mRNA vaccines which proven to be so efficacious as they saved millions of people from the corona pandemic in the past two years. On the impact of serendipity in science, and a lucky fate of cap from basic to application, I will be pleased to share with you these provocative subjects at the conference.

### [PS12-02]
### RNA metabolism and COVID-19

*Fanyan Wei[1] ([1]*Tohoku University, Institute of Development, Aging and Cancer*)

A variety of chemical modifications of RNA are essential for RNA stability and translation. The COVID-19 mRNA vaccine, which has been administered worldwide, is also artificially modified and is essential for its effectiveness. Thus, RNA modification has become become a next-generation drug discovery technology. Recently, we have conducted research on RNA modification from the viewpoint of metabolism, and discovered that RNA bases containing modifications, which are produced as a result of RNA metabolism, activate receptors as a humoral factor outside the cell and induce an immune response. In this symposium, we would like to share our latest findings for a comprehensive understanding of RNA metabolism and RNA-based medicine for post-COVID-19 era.

### [PS12-03]
### Drug repositioning study focusing on risk factors for aggravation and sequelae of COVID-19

*Motohiro Nishida[1,2], Yuri Kato[1], Kazuhiro Nishiyama[1], Akiyuki Nishimura[2], Yasunari Kanda[3] ([1]*Department of Physiology, Graduate School of Pharmaceutical Sciences, Kyushu University,* [2]*Division of Cardiocirculatory Signaling, National Institute for Physiological Sciences (Exploratory Research Center on Life and Living Systems), National Institutes of Natural Sciences,* [3]*Division of Pharmacology, National Institute of Health Sciences*)

Two years have passed since SARS-CoV-2, the cause of the new coronavirus infection (COVID-19), was discovered in Wuhan in 2019. The number of COVID-19 patients is declining in Japan may be thanks to the development of vaccines and the promotion of vaccination, but the severity and sequelae after COVID-19 infection remain as major issues. We investigated the mechanism of myocardial damage, a severe symptom of COVID-19, and found that several risk factors for COVID-19 cardiac severity, such as smoking, hyperglycemia, and hypertension, commonly caused interactions between TRPC3 channel protein and NADPH oxidase (Nox) 2 protein on the myocardial plasma membrane. The formation of TRPC3-Nox2 protein complex increased the expression of the SARS-CoV-2 host receptor, ACE2. From the top 13 approved drugs that can inhibit TRPC3-Nox2 complex formation, we screened a drug that can additionally suppress the internalization of ACE2 by the exposure to recombinant Spike protein of SARS-CoV-2. As a result, we found that clomipramine, a tricyclic antidepressant, showed a strong potency to inhibit SARS-CoV-2 entry and infection in human iPS-derived cardiomyocytes (hiPSCMs). Clomipramine also attenuated the Spike protein-exposed impairment of cardiomyocyte oxidative metabolism and contractility, as well as production of cytokines and reactive oxygen species. In this symposium, we will discuss the potentiality of repurposing clomipramine for the treatment of COVID-19 severity and sequelae.

### [PS12-04]
### A novel method for diagnosis and determining severity and therapeutic effect of COVID-19

*Kazuhito Tomizawa[1] ([1]*Department of Molecular Physiology, Faculty of Life Sciences, Kumamoto University*)

At the bedside of COVID-19 patients, one severe problem is that PCR and antigen tests for COVID-19 show only negative or positive results and they are not suitable for determining the severity. We have shown that many modified nucleosides, degradation products of RNA, are abundant in extracellular regions such as blood and urine. Recently, unknown highly modified area was reported from genome RNA of SARS-CoV-2. From these backgrounds, we made the hypothesis that the blood and urine of COVID-19 patients may present a COVID-19-specific profile of modified nucleosides. We searched for COVID-19 specific modified nucleosides from serum and urine of the patients using liquid chromatogram mass spectrometry system (LC-MS), and found that two modified nucleosides were specifically increased in serum and urine of COVID-19 patients. Moreover, these elevations were associated with severity of COVID-19. After the infection improved, the amount of the modified nucleoside was decreased. In the serum of COVID-19 patients infected with mutant strains, equivalent modified nucleosides elevations were observed. These results suggest that the measurement of modified nucleosides is useful for accurate assessment of COVID-19 severity and treatment efficacy in addition to the diagnosis.

### [PS12-05]
### Breath sulfur omics and its application to COVID-19

*Takaaki Akaike[1] ([1]*Tohoku University Graduate School of Medicine*)

Our recent discovery of the significant physiological relevance of supersulfides in energy metabolism prompted us to innovate a novel translational and clinical approach to explore an exhalation metabolism analysis, that is now called as breath sulfur omics. In this breath analysis, the exhaled aerosols are collected as the exhaled breath condensate (EBC) and applied to LC-MS/MS analysis. We thus successfully detected many sulfur metabolites such as sulfite, thiosulfate, and hydropersulfide in the human exhaled air. Especially, the levels of thiosulfate and hydropersulfide in human aerosols collected from the COVID-19 were significantly higher than that of healthy people. The breath sulfur analysis also revealed that the amount of endogenous metabolites derived from the sulfur respiration correlates well with the disease severity of the COVID-19 patients. These preliminary data suggest that supersulfides and related sulfur metabolites may become excellent and promising biomarkers for airway infections like COVID-19 and even other various diseases, in which the endogenous supersulfide production is affected and thereby involved in their pathogenesis.

### Planning Symposium 13

### [PS13]
### Decoding astrocyte information

March 18(Fri), 8:30 - 10:30, Room G

### [PS13-01]
### Tripartite synaptomics discovers astrocytic control of inhibition *in vivo*

*Tetsuya Takano[1,2] ([1]*Department of Neurophysiology, Keio University School of Medicine,* [2]*PRESTO・JST*)

Perisynaptic astrocyte processes are an integral part of central nervous system synapses; however, the molecular mechanisms governing astrocyte-synapse adhesions and how astrocyte contacts control synapse formation and function are largely unknown. Here we develop an *in vivo* specific cell-type interface proteomic approach, Split-TurboID, that uses a cell surface fragment complementation strategy. We thus identify a proteome enriched at astrocyte-neuron junctions *in vivo*, including Neuronal Cell Adhesion Molecule (NRCAM). We find that NRCAM is expressed in cortical astrocytes, localized to perisynaptic contacts, and is required to restrict neuropil infiltration by astrocytic processes. Furthermore, we show that astrocytic NRCAM transcellular interacts with neuronal NRCAM that is coupled to gephyrin at inhibitory postsynapses. Depletion of astrocytic NRCAM significantly reduces inhibitory synapse numbers without altering glutamatergic synaptic density. Moreover, loss of astrocytic NRCAM dramatically reduces inhibitory synaptic function with minor effects on excitation. Thus, our results present a proteomic framework for how astrocytes interface with neurons and reveal how astrocytes control GABAergic synapse formation and function.

### [PS13-02]
### Astrocytic regulation of homeostatic learning

*Teppei Kanaya[1], Ko Mastui[1,2] ([1]*Super-network Brain Physiology, Graduate school of Medicine, Tohoku University,* [2]*Super-network Brain Physiology, Graduate school of Life Science, Tohoku University*)

Memory formation depends on the state of mind. Memory can rapidly be formed during training (online learning) and memory can gradually solidify in between trainings (offline learning). Using a mouse model of cerebellar motor learning paradigm, we found that these two memory processes are often compensatory. The early bloomers with booming shortterm memory often have a suppressed long-term memory formation and late bloomers with no apparent acute training effect often exhibit boosted offline learning results. We hypothesized that the switch between these strategies for acquisition of adaptive behavior depends on the actions from the glial cells. Glia-specific knockout of an anion channel subunit LRRC8A resulted in suppression of online learning while the enhanced offline learning allowed the training effect to catch up to the control mice in between the sessions. Optogenetic activation of channelrhodopsin-2 (ChR2) in cerebellar glia results cytosolic acidification followed by glial glutamate release via anion channels. Glial ChR2- photoactivation resulted in boosted online learning. On the other hand, glial archaerhodopsin-T (ArchT) photoactivation, which results in glial alkalization, suppressed the online learning. Interestingly, glial ChR2-photoactivation in between training sessions had no effect on the offline learning, whereas ArchT-photoactivation allowed offline learning to flourish. These results suggest that the efficacy of both the online/offline learning is affected by the activity of glial cells and the mode of adaptive behavior depends on the state of glial cells.

### [PS13-03]
### Spatiotemporal dynamics in glia-neuron communication through ATP signaling

*Eiji Shigetomi[1,2], Schuichi Koizumi[1,2] ([1]*GLIA center, Interdiscipl Grad Sch of Med, Univ Yamanashi,* [2]*Dept Neuropharmacol, Interdiscipl Grad Sch of Med, Univ Yamanashi*)

Communication between glia and neurons is one of keys for development, function and disease of the brain. Glia utilize diverse types of chemical mediators such as neurotransmitters, neuromodulators, and cytokines, to communicate with neurons, and its actions to neurons are diverse, too. However, how the communication is regulated in time and space is not well understood. We have focused on extracellular ATP and its receptor to ask how the ATP signaling works in glia-neuron communications. To do so, we performed electrophysiology, state-of-art imaging, transcriptomics, and immunohistochemistry. We have made three key observations as follows. *First*, ATP which is released in actionpotential manner reached to neuronal components first and subsequently to astrocytes, *Second*, ATP action through astrocytic P2Y1 receptor regulate expression of a previously undescribed secretory protein, which enhances neuronal excitability through increase in excitatory synaptic transmission, *Third*, microglia regulate ATP dynamics by degrading ATP and have a negative impact on astrocytic P2Y1 receptor expression. Our data show that spatiotemporal ATP dynamics and its function are tightly regulated through the interaction with astrocytes and microglia.

### [PS13-04]
### Behavioral physiology with new perturbation and dissection tools for astrocytes

*Jun Nagai[1] ([1]*RIKEN Center for Brain Science*)

Astrocytes tile the entire brain and serve multifaceted roles therein. Using the dorsal striatum as a model microcircuit for exploring astrocyte biology, I integrated multiple approaches, including calcium imaging, electrophysiology, opto/pharmaco-genetics, mouse behavioral tests, RNA-seq and new tools for molecularly dissecting and physiologically perturbing astrocytes that have been recently developed. I will report latest insights on astrocyte roles in behavioral physiology. First, I will describe mechanisms of bi-directional neuron-astrocyte communications that lead to hyperactivity and disrupted attention via an astrocyte synaptogenic cue (Cell 2019). Second, I will present how astrocytes respond to distinct perturbations and how we can use the molecular signaling information for phenotypic benefits in neurodegenerative disease mouse models, e.g. Huntington's disease (Neuron 2020). Third, I will report a validation work for a new effective, specific and consequential attenuation tool of astrocyte Gq-GPCR signaling in vivo (Neuron 2021). Together, data demonstrate that signaling from astrocytes to neurons is sufficient per se to modulate local circuits and, animal behavior.

### [PS13-05]
### Insights from computational approaches on astrocytic calcium activity at the nanoscale

*Audrey Denizot[1], Erik De Schutter[1] ([1]*Okinawa Institute of Science and Technology*)

Astrocytes are essential to brain function, from synaptogenesis to higher-brain functions such as learning. One astrocyte contacts hundreds of thousands of synapses, potentially orchestrating information integration and transmission within various neural circuits simultaneously. Yet, the mechanisms that govern neuron-astrocyte communication and its implications in brain function remain unclear. Astrocytes communicate with neighboring cells through $Ca^{2+}$ signals that display complex, diverse spatio-temporal characteristics. 80% of astrocytic $Ca^{2+}$ signals are confined into small compartments within astrocytes that cannot be resolved with conventional light microscopy, hindering our ability to make sense of those signals. Here, we present how stochastic computational approaches provide new insights to astrocyte activity at the nanoscale. We use STEPS to perform simulations with high spatial resolution in three dimensions. Automatic handling of astrocyte meshes reconstructed from electron microscopy allows us to finely test the effect of morphological features on $Ca^{2+}$ activity. Our work contributes to the global effort towards decoding astrocyte activity and its functional implications.

### Symposia

### Public Symposium 1

### [SY01]
### The importance of magnesium homeostasis in physiological functions

March 16(Wed), 9:00 - 11:00, Room E

### [SY01-01]
### Reactive oxygen species disrupt magnesium homeostasis in rat ventricular myocytes

*Michiko Tashiro[1], Utako Yokoyama[1] ([1]*Department of Physiology, Tokyo Medical University*)

Magnesium deficiency is reported as a precipitating factor in ischemic heart disease. The pathogenesis of ischemic heart disease may be related to calcium overload induced by reactive oxygen sepsis (ROS) in cardiomyocytes. Because $Mg^{2+}$ can antagonize $Ca^{2+}$, $Mg^{2+}$ is speculated to protect cardiomyocytes from ROS-mediated damages. To study the effects of ROS on $Mg^{2+}$ dynamics in cardiomyocytes, we measured intracellular free Mg concentration ($[Mg^{2+}]_i$) after hydrogen peroxide ($H_2O_2$) administration in acutely isolated rat ventricular myocytes. $H_2O_2$ decreased $[Mg^{2+}]_i$ in a concentration-dependent manner with an $EC_{50}$ of ∼400 μM. The maximum rate of change in $[Mg^{2+}]_i$ calculated from the concentration-response curve was 0.77 μM/sec, that could result in ∼50% decrease in $[Mg^{2+}]_i$ in 10 min. Although a $Na^+$-dependent $Mg^{2+}$ extrusion system has been identified as a pivotal mechanism for the maintenance of Mg homeostasis in cardiomyocytes, this $H_2O_2$- mediated decrease in $[Mg^{2+}]_i$ was also observed in the absence of extracellular $Na^+$. To investigate whether the ROS-activated decrease in $[Mg^{2+}]_i$ was caused by $Mg^{2+}$ extrusion, we perfused rat hearts with the $Ca^{2+}$-free Tyrode' s solution containing $H_2O_2$ on the Langendorff apparatus. After 500 μM $H_2O_2$ stimulation, $Mg^{2+}$-concentration was significantly increased in the perfusate. These results suggest the existence of a $Na^+$-independent $Mg^{2+}$-extrusion system activated by ROS. ROS-induced myocardial dysfunction may be associated with a newly identified $Na^+$-independent $Mg^{2+}$-extrusion. (COI: No)

### [SY01-02]
### Pathological roles of $Mg^{2+}$ influx regulating by TRPM6 channel in cancer chemotherapy.

Aya Manabe[1], Saki Onuma[1], *Yuta Yoshino[1], Hajime Hasegawa[3], Satoshi Endo[1], Toshiyuki Matsunaga[2], Akira Ikari[1] ([1]*Laboratory of Biochemistry, Department of Biopharmaceutical Sciences, Gifu Pharmaceutical University* , [2]*Education Center of Green Pharmaceutical Sciences, Gifu Pharmaceutical University,* [3]*Department of Nephrology and Hypertension, Saitama Medical Center, Saitama Medical University*)

Several anticancer drugs including anti-epidermal growth factor receptor (EGFR) drugs induce hypomagnesemia. However, it remains fully uncertain whether $Mg^{2+}$ deficiency affects chemosensitivity of cancer cells. We firstly investigated the effect of low $Mg^{2+}$ concentration (LM) on proliferation and chemosensitivity using human lung adenocarcinoma A549 cells. LM suppressed cell injury induced by anticancer drugs. LM induced chemoresistance mediated by reactive oxygen species production and G1 arrest. Moreover, LM-induced cancer stemness properties were mediated by p38 MAPK. Next, we investigated the effects of novel candidate drugs on the expression of transient receptor potential melastatin 6 (TRPM6) $Mg^{2+}$ channel suppressed by anti-EGFR drugs in renal tubular epithelial NRK-52E cells. Rosiglitazone, an antidiabetic drug, and all-trans-retinoic acid (ATRA), a vitamin A derivative, induced elevation of mRNA level, $Mg^{2+}$ influx, and promoter activity of TRPM6 in the presence of erlotinib, an EGFR inhibitor. In conclusion, it should be necessary to prevent the reduction of body $Mg^{2+}$ content, to obtain the maximum effect in cancer chemotherapy. Furthermore, rosiglitazone and ATRA might be effective candidates to restore the reduction in renal $Mg^{2+}$ reabsorption caused by anti-EGFR drugs.

### [SY01-03]
### Maintenance of magnesium homeostasis by CNNM and various diseases caused by its disruption

*Yosuke Funato[1], Osamu Hashizume[1], Daisuke Yamazaki[1], Hiroaki Miki[1,2] ([1]*Department of Cellular Regulation, Research Institute for Microbial Diseases, Osaka University,* [2]*Center for Infectious Disease Education and Research (CiDER), Osaka University*)

Cyclin M (CNNM) is a family of membrane proteins with domain structure conserved from prokaryotes to humans, and there are 4 genes (CNNM1-CNNM4) in mammals. From various studies including elemental analyses and imaging experiments with $Mg^{2+}$-specific probes, we have shown that CNNM plays an important role in the maintenance of intracellular $Mg^{2+}$ homeostasis through its function as a $Mg^{2+}$-exporting transporter. Knockout mouse studies have also revealed that CNNM4 and CNNM2 are important for (re)absorption of $Mg^{2+}$ in the intestine and kidney, respectively. In addition to these physiological functions, the relationship between CNNM and the pathogenesis of various diseases has also becoming clear. Phosphatase of regenerating liver (PRL), which is highly expressed in various malignant cancers including colorectal metastasis, binds to CNNM and inhibits $Mg^{2+}$ efflux function of CNNM. The inhibition leads to the increase of intracellular $Mg^{2+}$ levels, which is crucial for PRL-induced malignant progression. The relationship of CNNM with blood pressure regulation and schizophrenia has also been pointed out, which will be also discussed in this symposium.

### [SY01-04]
### Clinical implications of magnesium in chronic kidney disease

*Yusuke Sakaguchi[1] ([1]*Department of Inter-Organ Communication Research in Kidney Diseases, Osaka University*)

Despite the importance of magnesium for the human body, the role of magnesium in the field of nephrology has not been well studied. Hypomagnesemia is not rare among patients with chronic kidney disease (CKD) despite their deteriorated glomerular filtration rates. Renal tubular dysfunction due to tubulointerstitial fibrosis might play a pivotal role in the development of hypomagnesemia in CKD by inducing urinary magnesium wasting.

A remarkable feature of magnesium in the context of CKD is its effect on vascular calcification. We conducted the first randomized controlled trial showing the efficacy of magnesium oxide in preventing the progression of coronary artery calcification among pre-dialysis patients with CKD (Sakaguchi Y. J Am Soc Nephrol 2019). We also found that hemodialysis patients with mild hypermagnesemia have a better overall survival than those with normal or low serum magnesium levels (Sakaguchi Y. Kidney Int 2014). Nevertheless, the underlying mechanism for the inhibition of vascular calcification by magnesium remains elusive. Concentrations of serum calcium and phosphate exceed the solubility product for hydroxyapatite. In order to prevent calcium-phosphate crystal formation, circulating calcium-phosphate complexes are absorbed by fetuin-A to form soluble colloidal particles (calciprotein particles [CPPs]) that are composed of amorphous calcium phosphate (CPP1). In CKD, hyperphosphatemia promotes calcium-phosphate crystallization inside CPPs, resulting in the formation of crystalline CPPs (CPP2). Notably, CPP2, but not CPP1, strongly induces calcification of vascular smooth muscle cells. Magnesium is known to inhibit CPP crystallization and thus alleviate the calcification stress induced by CPP2.

Recently, it has been shown that crystalline CPPs induce renal tubular injury. Therefore, magnesium might also be protective against phosphate-induced kidney injury by suppressing the CPP toxicity to the kidney. In fact, low-magnesium diet aggravated high-phosphate diet-induced kidney fibrosis (Sakaguchi Y. Nephrol Dial Transplant 2019). Furthermore, magnesium prevented high phosphate-induced cell death and proximal tubular cell inflammation (Sakaguchi Y. Kidney Int 2015).

Taken together, magnesium may exert favorable effects on both renal and cardiovascular outcomes. Further studies should be conducted to elucidate the clinical efficacy of magnesium in patients with CKD.

### Public Symposium 2

### [SY02]
### We research Nursing Science !

March 16(Wed), 9:00 - 11:00, Room F

### [SY02-01]
### Current treatment and potential therapeutic targets of biofilms in chronic nonhealing wounds

*Emi Kanno[1] ([1]*Department of Science of Nursing Practice, Tohoku University Graduate School of Medicine*)

Chronic nonhealing wounds are usually colonized with bacteria and subsequent infection may develop. Previously, it has been reported that the presence of biofilms in 60% to 100% of chronic wounds, such as pressure ulcers and diabetic foot ulcers. Recently, the prevention and management of biofilm in chronic wounds is rapidly becoming a primary objective of wound care. The Global Wound Biofilm Expert Panel states that biofilm-based wound care is predicated on using multiple different treatment strategies simultaneously including antibiotics, anti-biofilm agents, selective antimicrobials and frequent debridement. Moreover, other researchers caution that while focused activity against the biofilm in paramount, maximizing the host response must also be addressed with attention paid to all local and underlying causes of delayed wound healing. Our team identified *Peudomonas aeruginosa* biofilms on experimental rat wounds within 8 h by accompanying the local accumulation of neutrophils. These findings suggest that neutrophilic inflammatory responses may be involved in induction of biofilm formation. I would like to discuss in potential therapeutic targets about treatment of biofilms.

### [SY02-02]
### Cold-Sensitivity Constitution (*Hiesho*) is a decrease in peripheral blood flow and skin surface temperature caused by sympathetic nervous hyperactivity

*Kaori Kono[1] ([1]*Dokkyo Medical University*)

Cold-sensitivity constitution (CSC), known as "*Hiesho*" in Japanese, is a sensation of feeling cold, whereby the individuals are unable to warm themselves. Largely seen in menopausal women, CSC is being frequently observed even among younger women of late. CSC is generally an indefinite complaint with an unclear mechanism. Therefore, an objective criteria for its identification and treatment methods have not yet been established. Data from 20 healthy women in their twenties were studied. Based on the responses to a CSC questionnaire, they were categorized into CSC and non-CSC groups. Comparisons were drawn regarding, heart rate, autonomic nervous activity, blood pressure, skin surface temperature, skin blood flow, and tympanic membrane temperature. Women with CSC had lower parasympathetic nervous activity, higher sympathetic nervous activity, decreased blood flow, and lower skin temperature in peripheral circulation, compared to women in the non-CSC group. The results also indicated a difference in temperature of 6 ℃ or more between the tympanic membrane temperature and the skin temperature of the toes, among women with CSC; a useful criterion for CSC. It was suggested that CSC is a significant decrease in skin blood flow and skin surface temperature due to the contraction of peripheral blood vessels caused by sympathetic nervous hyperactivity. There are no conflict of interests regarding this study.

### [SY02-03]
### Development of an educational program to care for patients with infectious diseases

*Michiko Saito[1] ([1]*Dokkyo Medical University School of Nursing*)

Patients infected with multidrug-resistant organisms are recommended to follow standard and contact precautions. Isolation in a single room is essential considering the risk of infection to other patients. Nurses are required to learn basic knowledge and skills of infection control measures, such as practicing hand hygiene, choosing personal protective equipment (PPE), and wearing the PPE correctly. The mental state of patients in isolation should also be considered. Therefore, it is necessary to develop and evaluate educational programs for nurses, which aim at practicing infection control measures and mental care for isolated patients, and to evaluate their effects. This presentation reports the process of developing an educational program using methods that motivate nurses to learn (ARCS model) and solve problems by acquiring new knowledge (the first principal in the instructions). Moreover, the effects of the educational program on nurses taking care of isolated patients were evaluated by comparing the implementation and nonimplementation groups. There is a possibility that the process for developing and evaluating these educational programs may soon be applied in caring for patients with the coronavirus disease-2019.

### [SY02-04]
### For safe early mobilization after surgery - Is it possible to predict the onset of postoperative atrial fibrillation by evaluating preoperative cardiac autonomic nervous activity?

*Konosuke Sasaki[1] ([1]*Tohoku University Graduate School of Medicine*)

Postoperative atrial fibrillation (POAF) can cause hemodynamic deterioration. It is important for registered nurses to assess the risk of POAF in patients undergoing cardiovascular surgery to promote early mobilization efficiently. The postoperative change in autonomic nervous activity (ANS) is the one of the risk factors for POAF; however, preoperative ANS has not been evaluated in relation to POAF. We aimed to clarify the association between POAF and preoperative cardiac ANS. This observational study consisted of randomly selected 56 patients who underwent various cardiovascular surgeries. ECG was recorded preoperatively to evaluate ANS by using spectral analysis of heart rate variability (HRV). POAF occurred in 22 patients (39.3%). The high frequency component of HRV, an index of cardiac parasympathetic nervous activity, was classified into quartile. The incidence of POAF was variable among the patients in each quartile; 64.3% in the first quartile, 28.6% in the second, 14.3% in the third, and 50 % in the fourth quartile ($p=0.034$). Multiple regression analysis revealed that the first quartile which reflects the low parasympathetic nervous activity was detected as an independent risk factor for POAF. Preoperatively assessed low parasympathetic nervous activity can identify the subgroup of patients at high risk for POAF, which may facilitate personalized rehabilitation.

### [SY02-05]
### Exploring a new therapeutic approach focusing on leukocyte function

*Hiromasa Tanno[1] ([1]*Department of Science of Nursing Practice, Tohoku University Graduate School of Medicine*)

Skin wound healing process consists of inflammation, proliferation and remodeling phase. Wounds are categorized as acute or chronic based on the time it takes them to heal. Acute wounds generally heal within 2 weeks whereas chronic wounds can take several months or longer. Patients with chronic wounds, including diabetic ulcers and pressure ulcers, suffer from many problems, such as an increased risk of infection and impaired leukocyte function. Although multiple wound therapies have been developed, such as debridement, and treatment with antibacterial dressing, there is no therapies that focuses on leukocyte function. Leukocytes play an important role in skin wound healing and host defense against bacterial infection. Recently, we showed that activation of invariant natural killer T (iNKT) cells, an innate immune leukocyte, resulted in acceleration of healing process. Furthermore, we also reported that topical administration of heat-killed lactic acid bacteria (LAB) promotes this process by enhancing leukocyte function. In this presentation, I will talk about the effects of iNKT cell activation and topical administration of heat-killed LAB on acute wound and chronic wound models.

### Public Symposium 3

### [SY03]
### Mereological approach for understanding the brain

March 16(Wed), 9:00 - 11:00, Room G

### [SY03-01]
### Representation of fear memory by inter-regional coactivations of local cell assemblies

*Kenji Mizuseki[1] ([1]*Osaka City University*)

The amygdala, hippocampus, and prefrontal cortex are involved in fear memory. However, how these brain areas co-operate to support fear memory remains elusive. To address this question, we performed simultaneous large-scale electrophysiological recordings from the basolateral amygdala, ventral hippocampus, and prefrontal cortex of fear conditioned rats. Recordings were performed continuously throughout baseline, conditioning, context retention, cue retention and extinction, retention of extinction, and homecage sessions preceding, interleaved, and following the behavioral sessions. The proportion of time spent in freezing behavior indicated that the rats had learned an association between the cue and shock, and they retrieved the association during retention sessions. Based on our preliminary results, we hypothesize that elements of a given memory are instantly encoded by local cell assemblies within various brain regions, whereas the inter-regional coactivation of the distributed information develops in an experience-dependent manner during memory consolidation.

### [SY03-02]
### Synaptic mechanisms underlying aversive valence modulation and memory update

*Ayako M. Watabe[1] ([1]*The Jikei University School of Medicine*)

Aversive sensory stimuli can potently induce adaptive behaviors *via* emotional memory formation. While the neural mechanisms underlying fear memory formation has been intensively studied, the neuronal mechanisms of valence-related regulation are only partly understood. The pontine parabrachial nucleus (PB) receives diverse sensory signals, and broadcasts them to the multiple downstream targets, including the central amygdala (CeA), the bed nucleus of stria terminalis, the ventral posteromedial thalamic nucleus, and the parasubthalamic nucleus. While the PB-CeA pathway is involved in the emotional aspect of pain, how other target regions are involved in regulating aversive valence are yet to be elucidated. To tackle these problems and dissect the neuronal circuits underlying aversive valence modulation and memory update, we have recently invented a new ChR2 variant, which is precisely localized at axonal terminals with limited expression in soma and dendrites. While optogenetics is widely used for circuit mapping and manipulation, ChR2 variants selectively transported to long-range axonal projections for presynaptic activation have remained lacking. As a result, ChR2 activation is often contaminated by the spurious activation of *en passant* fibers that compromise the accurate interpretation of functional effects. Therefore, it would provide a powerful tool optimized for axon terminal activation and circuit mapping, thereby providing abundant possibilities for optogenetic experiments.

### [SY03-03]
### A neuro-mereology starting from the striatum to understand the neural processes supporting trial-and-error-based memory acquisition and habit formation

*Yoshio Iguchi[1] ([1]*Fukushima Medical University*)

In the basal ganglia, where the striatum plays as the input layer, we observe a drastic decrease in the number of neurons parallel to the circuit flow. This structure seems suitable for mapping environmental stimuli input through the cortex and thalamus to a few behavioral options. Memories for the stimulus-response connection are classified as procedural memory, and previous studies have reported that the lateral part of the striatum is involved. In contrast, the medial striatum has been implicated in the declarative memory processes, allowing computation of the consequence of behavior (e.g., reward and punishment). Adaptive behaviors acquired through trial-and-error, which lead organisms to reward and keep them from punishment, are supported by the declarative memory at the beginning and the procedural one after repeated experiences of the same situation. This "habit formation" is basically an adaptive shift of neural circuit though paradoxically enhanced by addictive drugs or stresses. In elucidating the paradox of habit formation, it is required to change the scope of research from one that focuses on the functions of the striatal sub-regions to one that reveals the global mechanisms of the circuit shift. We talk about our projects directing the "mereological" understanding of trial-and-error-based memory acquisition and habit formation and its prospects for the future.

### [SY03-04]
### Multiscale imaging to avoid mereological fallacy in neurophysiology

*Makoto Osanai[1,2,3] ([1]*Osaka Univ.*, [2]*Tohoku Univ.*, [3]*CiNet*)

"Mereological fallacy" (Smit & Hacker, 2014) is ascribed from the idea that the whole can be represented by a set of parts. The nervous system has a highly hierarchical structure and interactions within and between each level of the hierarchy. To avoid mereological fallacy and to reveal the function expression mechanisms of the brain, it is necessary to observe multiscale signals and their interactions. Along with the idea, we have been developing multiscale imaging methods. For in vivo whole-brain activity analysis, we developed and used the quantitative activation-induced manganese-enhanced MRI (qAIMMRI). qAIM-MRI is based on the use of $Mn^{2+}$ as a surrogate marker of $Ca^{2+}$ influx. $Mn^{2+}$ shortens the longitudinal relaxation time (T1) of $H^{+}$. Therefore, qAIM-MRI can measure the history of neuronal activities non-invasively. For in vivo local circuit imaging, we are developing the ultra-thin fluorescence endoscope imaging system (U-FEIS). U-FEIS can record the multicellular neuronal activities from the deep brain region. To reveal the cellular and molecular mechanisms for exhibiting brain function, we have been conducting $Ca^{2+}$ imaging on brain slice preparations. These three imaging methods can be applied to the same animal and can combine with behavioral and biochemical studies. This strategy may provide a research foundation for understanding the relationship between parts and the whole brain, avoiding mereological fallacies in neurophysiology.

### [SY03-05]
### Data-driven Approach for Multi-dimensional and Multi-scale Data Analysis

*Toshiaki Omori[1] ([1]*Kobe University*)

Due to recent developments in measurement technology, data that we have to deal with have become high-dimensional. Therefore, it is important to establish a method for extracting essential elements underlying such observable high-dimensional data. Moreover, in order to understand complex systems with hierarchy such as neural systems, it is also necessary to extract functional interactions across multi-scale levels. In this study, we discuss a data-driven method for analyzing multi-dimensional and multi-scale data in neural systems in order to explore a mereological approach for understanding the brain.

### [SY03-06]
### Closing remarks

*Hajime Mushiake[1] ([1]*Tohoku University, Graduate School of Medicine*)

In this symposium, we attempted to understand brain function from the perspective of the relationship between parts and the whole.Front-line researchers introduced interesting research results, focusing on the memory system.
We had interesting discussions from the perspective ofthe dynamics of the network, without being lmiting to the conventional classification of memory systems. There are many clinical problems related to memory, including PTSD.
We are looking forward to better understanding of memory in basic research from the new perspectives.

### Public Symposium 4

### [SY04]
### Hot topics on muscle research using zebrafish models

March 16(Wed), 9:00 - 11:00, Room H

### [SY04-01]
### Drug screening for muscle atrophy using transgenic zebrafish model.

*Genri Kawahara[1], Yukiko Hayashi[1] ([1]*Department of Pathophysiology, Tokyo Medical University*)

Zebrafish is an excellent animal model for human diseases and drug screening. To monitor the expression of Muscle RING-finger protein-1 (*MuRF1*) gene, which is one of marker molecules of muscle atrophy, a transgenic zebrafish line was created with microinjection of *murf1* promoter-EGFP cDNA construct using tol2 transposon system.During early development in the transgenic fish (*murf1*:EGFP) line, EGFP signals were observed in skeletal muscle and heart from 1 day post-fertilization (dpf). RT-PCR analysis confirmed that the *murf1* gene expression was corresponded with EGFP expression after 1 dpf. In the adult transgenic fish, *murf1* expression corresponding with EGFP were mainly observed in skeletal muscle and heart. Treatment with dexamethasone solution at 4 dpf for 24 hours induced up-regulation of EGFP expression in *murf1*:EGFP zebrafish. These results indicated that the *murf1* expression could be monitored using the *murf1*:EGFP fish. Using the *murf1*:EGFP zebrafish, we have screened 1,280 drugs to discovery chemicals to reduce the expression of zebrafish *murf1*, and five candidate chemicals were identified. Treatment with these candidate chemicals reduced *murf1* expression induced in dexamethasone treated zebrafish.Our *murf1*:EGFP fish line might be excellent tool to evaluate the expression of *murf1* and is useful to therapeutic drug screening for muscle atrophy.

### [SY04-02]
### A novel zebrafish model of aortic aneurysm

*Shota Tanifuji[1], Genri Kawahara[2], Takashi Nakamura[1], Saki Iida[1], Yukiko K Hayashi[2], Utako Yokoyama[1] ([1]*Department of Physiology, Tokyo Medical University,* [2]*Department of Pathophysiology, Tokyo Medical University*)

Aortic aneurysm (AA) is characterized by progressive aortic wall degeneration, leading to loss of structural integrity and fatal aortic rupture. AA is a progressive lethal disease, but no effective pharmacological therapy to inhibit its progression is currently available. Although several rodent models of AA have been developed, high-throughput drug screening is not feasible in these models. We, therefore, aimed to develop a novel model for phenotypebased drug discovery using optically transparent zebrafish. We microinjected angiotensin II (AngII) into *Tg* (*kdrl:EGFP*) zebrafish embryos in which vascular endothelial cells are labeled with EGFP at the 1 cell stage. The diameter of the dorsal aorta was measured at five locations using confocal microscopy at 5 days post-fertilization (dpf). The average aortic diameter was significantly increased in *Tg* (*kdrl:EGFP*) zebrafish injected with AngII (160 ng) compared to buffer injected controls (controls, 19.1±0.8 μm; AngII 160 ng, 23.2 ±0.7 μm; n=13-18, $p<0.05$). Elastica van Gieson staining revealed that elastic fiber formation of the dorsal aorta was significantly attenuated in AngII-injected zebrafish at 8 week-old (1.9±0.1-fold vs controls, n=5-12, $p<0.05$). These results suggest that AngIIinduced aortic diameter expansion at 5 dpf may associate with aortic elastic fiber dysregulation. AngII-injected *Tg* (*kdrl:EGFP*) zebrafish may be useful for high-throughput drug screening.

### [SY04-03]
### Zebrafish models of nuclear envelopathies and hereditary neuromuscular diseases

*Hiroaki Mitsuhashi[1] ([1]*Department of Applied Biochemistry, School of Engineering, Tokai University*)

Muscular dystrophies and related myopathies are genetic disorders that affect skeletal muscle. Mice have been widely used as animal models of the diseases; however, the zebrafish has recently received much attention as a new model system complementary to mammalian models. Zebrafish have unique advantageous attributes such as high reproductivity, rapid development, transparency of the embryos, ease of genetic manipulation, and the high conservation of neuromuscular genes. We aim to apply zebrafish to the model of human hereditary neuromuscular diseases. We generated laminopathy models that stably express mutant lamin A fused with EGFP specifically in skeletal muscle by the Tol2 transposon-mediated transgenic system. These model fish show that aggregates of the mutant lamin A and abnormal morphology of myonuclei. We have also generated zebrafish where muscle mitochondria and neuromuscular junctions are visualized with fluorescent proteins. These zebrafish will be useful to elucidate the pathogenesis of mitochondrial diseases and congenital myasthenia syndrome. In addition, we achieved to introduce microdeletion to causative genes of neuromuscular diseases by genome editing with CRISPR/Cas9 system. The combination of these genetic engineering technologies will accelerate the validation of the pathogenicity of genetic mutations and the search for therapeutics.

### [SY04-04]
### Elastin b is critical for the differentiation of smooth muscle cells in outflow tract of zebrafish

*Kazuko Koshiba-Takeuchi[1] ([1]*Department of Applied Biosciences Faculty of Life Sciences Toyo University*)

Teleost fish have a unique outflow tract called as bulbus arteriosus (BA). BA is a pearshaped structure and functions as a capacitor thought to be important for maintaining continuous blood flow into a gill. Non-teleost fish, such as *Polypterus* and gar, have conus arteriosus (CA). CA is composed of myocardium and have contraction ability, in contrast, BA is composed of smooth muscle, rich in elastin and have no contraction ability. BA is thought as an "evolutional novelty", but it is unclear how teleost develops such a unique outflow tract. In vertebrate evolution, the 3 round-whole genome duplication (3R-WGD) have specifically occurred in the teleost lineage. As a result, teleosts have two elastin genes, *elastin a* (*elna*) and *elastin b* (*elnb*), and *elnb* was strongly expressed in the BA. The *elnb* knockdown experiments induced hypoplasia of BA, and interestingly the ectopic cardiac muscle differentiation occurred in the BA. While the knockdown of *eln1* did not cause cardiac muscle differentiation. These results suggest that *elnb* has an important role in the differentiation of cardiac precursor cells into smooth muscles in the outflow tract and formation of BA. In zebrafish heart development, *fibulin 5* (*fbln5*) is also expressed in the BA. We found that both *elnb* and *fbln5* were regulated by TGF-β signaling in outflow tract development.

### Public Symposium 5

### [SY05]
### New insights into the function and regulation of TRP channels in pathology

March 16(Wed), 9:00 - 11:00, Room I

### [SY05-01]
**Müller glial s.elling activates TRPV4 and increases photoreceptor cell death in retinal detachment**

*Koji Shibasaki[1] ([1]*Laboratory of Neurochemistry, Graduate School of Human Health Science, University of Nagasaki*)

Using region-specific injection of hyaluronic acid, we developed a mouse model of acute retinal detachment (RD) to investigate molecular mechanisms of photoreceptor cell death triggered by RD. We focused on the TRPV4 ion channel, which functions as a thermosensor, osmosensor and/or mechanosensor. Following RD, the number of apoptotic photoreceptors was reduced by ~50% in TRPV4KO mice relative to wild type mice, indicating the possible involvement of TRPV4 activation in RD-induced photoreceptor cell death. Furthermore, TRPV4 expressed in Müller glial cells can be activated by mechanical stimuli caused by RD-induced swelling of these cells, resulting in release of the cytokine MCP-1, which is reported as a mediator of Müller glia -derived strong mediator for RDinduced photoreceptor death. We also found that the TRPV4 activation by the Müller glial swelling was potentiated by body temperature. Taken together, our results suggest that RD adversely impacts photoreceptor viability via TRPV4-dependent cytokine release from Müller glial cells and that TRPV4 is part of a novel molecular pathway that could exacerbate the effects of hypoxia on photoreceptor survival following RD.

### [SY05-02]
**Pathophysiological significance of reactive oxygen species-sensitive TRP channels in chronic cerebral hypoperfusion-related CNS diseases.**

*Hisashi Shirakawa[1] , Shuji Kaneko[1] ([1] *Department of Molecular Pharmacology, Graduate School of Pharmaceutical Sciences, Kyoto University*)

Emerging evidence indicates that chronic cerebral hypoperfusion (CCH), resulting in an inadequate oxygen and nutrient supply to the brain, is one of the factors that contribute to the onset or aggravation of various CNS diseases including neurodegenerative and mental disorders with cognitive impairment. Reactive oxygen species (ROS) could be involved in this process; however the pathological mechanism remains unclear. We employed bilateral carotid artery stenosis (BCAS) mice as a model of relatively mild chronic cerebral hypoperfusion, and found that minor disruption of the blood-brain barrier and generation of reactive oxygen species were observed, leading to brain inflammation and white matter damage, which in turn led to cognitive dysfunction. In the pathogenesis of BCAS-induced cognitive impairment, we investigated the involvement of TRPM2, a ROS-sensitive $Ca^{2+}$- permeable cation channel, and found that TRPM2 mediates microglial activation and contribute to the exacerbation of the symtoms. Furthermore, we have recently found that TRPA1, another ROS-sensitive TRP channel expressed in other brain cells, may be involved in the pathogenesis of BCAS, and we will also introduce our recent findings on TRPA1. These findings suggest that TRPM2 and TRPA1 could be a therapeutic target for CCH-related CNS diseases.

### [SY05-03]
**TRPC3-Nox2 complex formation in muscle atrophy**

*Yuri Kato[1], Kazuhiro Nishiyama[1], Motohiro Nishida[1,2] ([1]*Kyushu university*, [2]*National Institutes of Natural Sciences*)

Striated muscles, mainly cardiomyocytes and red muscles, play pivotal roles in systemic motor function, blood circulation, and energy metabolism. Muscle atrophy associated with aging or bedridden by sickness causes a decline in these systemic functions. We investigated the mechanism underlying induction of myocardial atrophy by anti-cancer drug treatment, and found that formation of protein signaling complex between transient receptor potential canonical (TRPC) 3 and NADPH oxidase 2 (Nox2) caused reactive oxygen species (ROS)-dependent myocardial atrophy in mice. We revealed that extracellular adenosine 5'-triphosphate (ATP) mediated the formation of TRPC3-Nox2 complex and resultant cardiomyocyte atrophy. Knockdown of either TRPC3 or Nox2 suppressed nutritional deficiency-induced ATP release, as well as ROS production and neonatal rat cardiomyocytes atrophy. Moreover, TRPC3-Nox2 complex formation was promoted in mice with muscle dystrophy (mdx). We identified that ibudilast, an already approved anti-inflammatory drug, was able to inhibit TRPC3-Nox2 complex formation in cardiomyocytes. In fact, treatment with ibudilast prevented the progression of skeletal muscle atrophy in mdx mice. These results suggest that targeting TRPC3-Nox2 protein complex will become a breakthrough strategy for the treatment of muscle atrophy-related refractory diseases.

### [SY05-04]
**Oxidative stress regulates TRPM7, a calcium influx pathway in adipocytes**

*Hana Inoue[1], Takashi Murayama[2], Takuya Kobayashi[2], Masato Konishi[1], Utako Yokoyama[1] ([1]*Department of Physiology, Tokyo Medical University*, [2]*Department of Cellular and Molecular Pharmacology, Juntendo University Graduate School of Medicine*)

TRPM7 is a $Ca^{2+}$-permeable, nonselective cation channel that is ubiquitously expressed but is predominantly expressed in adipose tissue in human adults. In mature adipocytes, the intracellular concentration of $Ca^{2+}$ affects insulin signaling, lipolysis, and secretion of adipokines. Using patch-clamp and $Ca^{2+}$ imaging techniques, we revealed that TRPM7 is functionally expressed in mature adipocytes and serves as a $Ca^{2+}$ influx pathway. We also found that TRPM7 is inhibited by hydrogen peroxide ($H_2O_2$), which has been reported to be generated in adipose tissues under physiological and pathological conditions. To further investigate the mechanisms underlying TRPM7 inhibition by oxidative stress, we cloned TRPM7 from mouse mature adipocytes. TRPM7 and its mutant channels were expressed in HEK293 cells and the effect of $H_2O_2$ on the channel activity was assessed. TRPM7 channel activity was inhibited by intracellular $Mg^{2+}$, and $H_2O_2$ enhanced this $Mg^{2+}$ -induced TRPM7 inhibition. TRPM7 is comprised of two functional domains: the channel domain and the kinase domain. The structural interaction between the domains attenuated the $Mg^{2+}$-induced inhibition of TRPM7 channel activity. Oxidative stress may disrupt the interdomain interaction by oxidizing cysteines of the zinc-binding motif in the kinase domain, resulting in inhibition of TRPM7 channel activity.

### Public Symposium 6

### [SY06]
### New perspectives on cerebellar computational mechanisms

March 16(Wed), 9:00 - 11:00, Room J

### [SY06-01]
### Plasticity of the intrinsic excitability in the acute inflammation and psychiatric disease models

*Gen Ohtsuki[1] ([1]*Department of Drug Discovery Medicine, Kyoto University Graduate School of Medicine*)

Intrinsic plasticity is the long-term modulation of intrinsic membrane excitability, which may underlie psychiatric-disease symptoms. In the cerebellar Purkinje cells, a couple of forms of intrinsic plasticity are identified: modulations of the firing frequency of soma (Belmeguenai et al., 2010; Shim et al., 2017) and an increase in the excitability of dendrites (Ohtsuki et al., 2012; Ohtsuki, 2020). Recently, we have revealed the induction of intrinsic plasticity by immune cells, such as microglia, in the cerebellum and the longlasting increase of the Purkinje-cell excitability via SK-channel downregulation. Animals with acute immune activation in the cerebellar anterior vermis showed depression-like behaviors and disruption of the cerebella-frontal cortical functional overconnectivity (Yamamoto et al., 2019). In contrast, in the medial prefrontal cortex, the activation of microglia induced hypoexcitability plasticity in the pyramidal cells, but not in fast-spiking interneurons. This form of immune-triggered plasticity was presumably mediated by TNF-$\alpha$ and SK1 (Yamawaki et al., *in revision*). Therefore, our results suggest that the directionality of the intrinsic plasticity by microglia is not consistent, depending on the brain region and the cell type. Further, in this talk, I will show the intrinsic plasticity and immunetriggered plasticity of the Purkinje cells in the mouse models of psychiatric diseases by both single- and multiple-immune stresses.

### [SY06-02]
### Extreme adaptation and ultimate glial meta-plasticity control

*Daichi Sasaki[1], Teppei Kanaya[1], Ko Matsui[1] ([1]*Tohoku University*)

Dynamic adaptation of the outlived neuronal circuit is the only path of survival after brain damage. To adapt to the new environment, the brain reboots its hidden capacity of remodeling that were apparently concealed after early development. Here, we investigate whether such enhanced potential of plasticity is triggered by ischemia. We focused on the cerebellar motor learning and the memory process associated with horizontal optokinetic response (HOKR). We found that the memory formed during training (online learning) and the memory solidified between training (offline learning) were compensatory. The online learning was accelerated with photoactivation of ChR2 expressed in cerebellar glial cells. In contrast, ArchT photoactivation resulted in online learning suppression but enhancement of offline learning. ChR2 and ArchT photoactivation result in intracellular acidification and alkalization, respectively. In the event of ischemic stroke, glial cells could become alkalized by the activation of Na-bicarbonate cotransporter. Loss of oxygen supply would lead to acidification by accumulation of lactate. These changes in the glial pH could become the trigger for the enhanced adaptive plasticity. Using fiberphotometry, spatiotemporal profile of glial pH and calcium were investigated over multiple days to understand the mechanisms underlying the dynamic changes in plasticity.

### [SY06-03]
### A novel role of ionotropic glutamate receptors in cerebellar synaptic plasticity and motor learning.

*Wataru Kakegawa[1,2], Michisuke Yuzaki[1,3] ([1]*Keio University*, [2]*JST-ERATO*, [3]*JST-CREST*)

Ionotropic glutamate receptors (iGluRs) mediate a fast excitatory transmission and synaptic plasticity such as long-term potentiation and depression (LTD), a molecular basis of learning and memory, in the CNS (Kakegawa and Yuzaki, *Brain and Nerve, 2018*). Accumulating evidence suggested that iGluRs behave not only as ion channels but also as non-ionotropic receptors. However, it remains unknown whether and how iGluRs serve as non-ionotropic receptors *in vivo*. Recently, we found that kainate-type iGluRs (KA-Rs) are selectively and functionally expressed at the synapses between climbing fiber (CF) and Purkinje cells in the cerebellum. Interestingly, mutant mice lacking GluK4, a high-affinity type of KA-R subunit, exhibited an impaired LTD at parallel fiber (PF)-Purkinje cell synapses and poor performance in cerebellum-dependent motor learning. Despite its importance, GluK4-containing KA-R is involved in less than 5% of CF-mediated excitatory postsynaptic currents (CF-EPSCs). Furthermore, applying a selective KA-R blocker effectively blocked a KA-R component of CF-EPSCs but not LTD in wild-type cerebellar slices. These results raise the possibility that KA-R in Purkinje cells works *in vivo* in an ion channel-independent manner. In this talk, we would like to discuss a novel role of KA-R in cerebellar synaptic plasticity and motor learning.

### [SY06-04]
### Modulation of cerebellar synaptic plasticity by thyroid hormone signaling

*Nobutake Hosoi[1], Ayane Ninomiya[2], Izuki Amano[2], Michifumi Kokubo[2], Hirokazu Hirai[1], Noriyuki Koibuchi[2] ([1]*Department of Neurophysiology and Neural Repair, Gunma University Graduate School of Medicine*, [2]*Department of Integrative Physiology, Gunma University Graduate School of Medicine*)

Thyroid hormones (THs) regulate gene expression by activating nuclear TH receptors (THRs) and TH signaling plays critical roles in cerebellar development. However, cell-type specific roles of TH signaling in the cerebellum remains unknown, because THRs are expressed all over in the cerebellum during the entire life span. Thus, in order to examine the roles of TH signaling in cerebellar Purkinje cells (PCs), we generated a transgenic mouse model which lacks TH signaling specifically in cerebellar PCs. This mutant mice displayed delayed cerebellar morphological development, and the morphological abnormalities were normalized by approximately 5 weeks of age (young adult age). However, this mutant mice showed motor defects still at adult stages, and we found that the mutant mice have abnormal long-term synaptic plasticity at parallel fiber(PF)-PC synapses, where typical long-term depression (LTD)-inducing protocol evokes long-term potentiation (LTP) in the TH signal-deficient mice. We also found reduced Ca responses to burst PF stimulation in PCs of the mutant mice. These results suggest that TH signaling in PCs plays important roles not only in cerebellar development but also in regulating Ca dynamics at PF-PC synapses and subsequently modulating the direction of synaptic plasticity at PF-PC synapses.

### Public Symposium 7

### [SY07]
### Strategy for health and longevity from the aspect of organ failure

March 16(Wed), 15:45 - 17:45, Room G

### [SY07-01]
### Anti-inflammatory effect via C1 neurons

*Chikara Abe[1] ([1]*Gifu University*)

Activation of the immune system via the autonomic nerves including sympathetic and parasympathetic nerves is one of a possibility for the preventive medicine. Electrical stimulation of the vagal afferents and/or efferents reduces subsequent inflammation. In the pathway through vagal afferent, stimulation of C1 neuron in medulla oblongata, which is one of the centers in autonomic nerves system, is important for anti-inflammatory effect. Furthermore, activation of immune cells in the spleen via the splenic sympathetic nerves is indispensable for the protective effect. Focusing on kidney injury and pneumonia, neural mechanism of the anti-inflammatory via C1 neurons will be explained in the symposium. On the other hand, C1 neurons are activated by peripheral vestibular stimulation. Thus, it is possible that peripheral vestibular stimulation induces anti-inflammatory effect. This possibility will be also discussed in this symposium.

### [SY07-02]
### Understanding of normal oral microbiota for oral and systemic health promotion

*Toru Takeshita[1] ([1]*Section of Preventive and Public Health Dentistry, Faculty of Dental Science, Kyushu University*)

Oral cavity is densely colonized by diverse microorganisms, which constitute complex but stable indigenous microbiota on various intra oral surfaces. Two major oral diseases, dental caries and periodontitis are caused by these bacteria, and strongly involved in tooth loss resulting in undernutrition, as well as poor quality of life. The oral microorganisms are also constantly ingested with saliva and transported into the airway and the intestinal tract. Recently, it is noted that the ingested oral bacteria contribute to respiratory diseases such as aspiration pneumonia and gut microbiota dysbiosis. Therefore, maintenance of the normal oral indigenous microbiota is required for oral and systemic health. However, the normal oral indigenous microbiota and its variation among healthy individuals remains uncharacterized. To obtain insights into oral indigenous microbiota associated with health conditions, we have conducted various epidemiological studies on oral microbiota in multiple research fields using comprehensive microbial community analyses based on DNA sequencing. This lecture introduces our finding on the overall structure of the oral microbiota associated with oral and systemic health for developing novel oral and systemic health promotion strategies.

### [SY07-03]
### Noisy Galvanic Vestibular Stimulation for the Treatment of Balance Disorders

*Chisato Fujimoto[1] ([1]*Department of Otolaryngology and Head and Neck Surgery, Graduate School of Medicine, The University of Tokyo*)

Noisy galvanic vestibular stimulation (nGVS) is a procedure that applies electrical current as zero-mean current noise to the vestibular system through electrodes placed over the bilateral mastoid process. With regard to the postural control system, stability in standing posture and gait performance were improved during the application of imperceptible optimal level of nGVS in healthy subjects and in patients with bilateral vestibulopathy (BVP). On the other hand, a further increase of nGVS intensity degrades stability of standing posture. Although the mechanism of the effect of nGVS on postural stability is not clear, stochastic resonance (SR) theory has been proposed, in which the presence of an optimal amount of noise enhances the subthreshold signal of a nonlinear system. nGVS also led to a post-stimulation ameliorating effect of the postural stability that lasts for several hours in healthy elderly adults and BVP patients, even after the cessation of the stimulus. This result that the ameliorating effect lasts for several hours even after the stimulation is ceased is considered to be a new phenomenon that cannot be explained by SR, which is assumed to be the mechanism of the effect during stimulation. nGVS is a promising candidate for a novel treatment of refractory postural instability due to vestibulopathy. Further clinical studies are needed to increase the evidence level of the therapeutic effects of nGVS. It is also expected to be applied to the treatment of agerelated balance disorders.

### [SY07-04]
### Pathophysiology and Therapeutic Strategies for the Heart Failure Pandemic in the Elderly

*Keita Saku[1] ([1]*National Cerebral and Cardiovascular Center*)

*Heart failure* is a major medical problem associated with high medical costs and mortality. It demonstrates high morbidity in elderly, with the disease occurring in 10% of those over 80 years of age (10 times more than those in 50s). In addition, aging is associated with an increased risk of cardiovascular events and short- and long-term mortality in heart failure patients. Although the expression "heart failure pandemic" has been used to sound the alarm, there is currently a lack of solutions to attenuate the pandemic because of superaging society in Japan. Minimally invasive therapies, such as catheterization and implantable cardiac devices, have made it possible for the elderly to be no longer excluded from active treatment for heart failure and improve the quality of life (QOL) and prognosis of high-risk elderly patients. However, it can also lead to futile prolongation of life, increased medical costs, and exacerbate the social problems associated with the heart failure pandemic. Thus, it is necessary to consider comorbidities, frailty, and the patient's or family's will when selecting treatment especially in heart failure elderly patients. In this session, we will review the characteristics of heart failure in the elderly, the position of current treatments in Japan, and new treatment strategies developed worldwide.

### [SY07-05]
### Renal failure prevention strategy by autonomic nerve control

*Tsuyoshi Inoue[1] ([1]*Nagasaki Univ.*)

The kidney is a highly developed organ, and has various functions such as regulation of fluid and electrolyte, regulation of blood pressure, production of erythropoietin, and activation of vitamin D. Progression to kidney disease is caused by various causes such as diabetes and hypertension, hereditary disease, and glomerulonephritis characterized by urine protein. At present, there is no drug for chronic kidney disease other than angiotensin II receptor antagonist (ARB), which has an inhibitory effect on the progression of kidney disease. Recent advances have shown that neural pathways are able to regulate immunity and inflammation. The cholinergic anti-inflammatory pathway is a well-studied neural circuit involving the vagus nerve that is thought to contribute to the response to inflammatory disorders. Indeed, we have so far elucidated the renal protective effect of vagus nerve stimulation and the importance of $\alpha 7$ nicotinic acetylcholine receptor in macrophages, and we identified novel factors existing downstream of $\alpha 7$ nicotinic acetylcholine receptor. Furthermore, we found that sympathetic nerves are important for the renal protective and anti-inflammatory effects of C1 neurons in the medulla oblongata, and that macrophages exert anti-inflammatory and renal protective effects through $\beta 2$ adrenergic receptors. In this session, I will briefly show anti-inflammatory and kidney protection mechanisms mediated by the autonomic-immune system. In particular, I will present the latest findings including the data obtained by single cell RNA-seq and I would like to discuss them with you.

### Public Symposium 8

### [SY08]
### Mind-body interaction and mental conditioning

March 16(Wed), 15:45 - 17:45, Room H

### [SY08-01]
### Perspectives on psychosomatic research

*Naoya Kataoka[1,2], Kazuhiro Nakamura[1] ([1] *Department of Integrative Physiology, Nagoya University Graduate School of Medicine*, [2] *Nagoya University Institute for Advanced Research*)

Psychosomatic correlation has been known as a phenomenon in which mental states and emotions affect the mechanisms for physiological regulations of the body, such as the autonomic and behavioral motor systems. Physiological research on psychosomatic correlation is important in understanding not only the fundamental neural circuit mechanisms of physiological responses to emotional stimuli and psychological stress, but also the etiologies of stress-related disorders and symptoms. Recent studies using rodents discovered important central circuit connections from the corticolimbic emotion and stress circuits to the hypothalamic and brainstem systems that control vital functions and behavior. We will briefly preview recent advances in psychosomatic research and discuss the potential directions of future research toward total understandings of the central circuitry for psychosomatic correlation and the etiologies of relevant disorders.

### [SY08-02]
### How emotions and breathing interact in the brain -View of olfactory system-

*Yuri Masaoka[1] ([1] *Department of Physiology, Showa University School of Medicine*)

The breathing pattern changes in a variety of emotions, such as fear, anxiety, joy and pleasure. Negative emotions, for example, anxiety increase respiratory frequency, showing rapid and shallow breathing patterns. Voluntary slowdown in respiratory rate has been demonstrated to reduce anxiety and stress. On the one hand, olfactory stimuli applied in aromatherapy are quite effective in modifying such negative emotions and physical state associated with stress. Olfactory perception depends on respiration activity, and pleasant olfactory stimuli unconscious change our breathing slower. Such physiological factors may interact with emotion regulation. In this study, we used functional magnetic resonance imaging (fMRI) to investigate the relationship between slow breathing and emotions, and breathing change and brain activations during odor stimuli which associated with individual emotional memory. fMRI analysis indicated activation in the left orbitofrontal region, and this area of activation strengthened the connectivity with the precuneus, medial frontal gyrus (MFG) and parahippocampus. The slow breathing observed during the odor stimuli was correlated with MFG activation. The MFG directly influences amygdala activity during emotion regulation. Our results suggest that MFG is a potential source of emotion regulation that can result from respiratory change.

### [SY08-03]
### Future subjective well-being can be predicted by the caudate volume and regulatory focus

*Masahiro Matsunaga[1], Hirohito Tsuboi[2], Kohta Suzuki1, Yohsuke Ohtsubo[3], Keiko Ishii[4], Haruto Takagishi[5] ([1] *Department of Health and Psychosocial Medicine, Aichi Medical University School of Medicine*, [2] *Institute of Medical, Pharmaceutical & Health Sciences, Kanazawa University*, [3] *Graduate School of Humanities and Sociology, The University of Tokyo*, [4] *Department of Cognitive and Psychological Sciences, Graduate School of Informatics, Nagoya University*, [5] *Brain Science Institute, Tamagawa University*)

Higgins' regulatory focus theory posits two self-regulatory orientations that people use to obtain their goals: promotion and prevention. A promotion focus entails a sensitivity to the presence and absence of positive outcomes. Previous studies have suggested that promotion focus positively predicts happiness. Tamagawa research project initiated in May 2012, the 10$^{th}$ wave of which has been underway as of March 2021. In this project, 470 participants underwent magnetic resonance imaging (MRI) and filled out a questionnaire regarding happiness and promotion focus in 2012. The same participants reported their subjective well-being (life satisfaction) in 2014 and 2018. Using these longitudinal data, we examined whether the MRI images and promotion focus measured in 2012 could predict subjective well-being after 2 and 6 years. Voxel-based morphometry analysis demonstrated that gray matter density (volume) of the caudate nucleus was positively correlated with rating scores of promotion focus and happiness in 2012. The regression analysis also demonstrated that both rating score of promotion focus and caudate volume predicted future subjective well-being. We found the possibility that the caudate volume and promotion focus can predict future subjective well-being.

### [SY08-04]
### Memory consolidation initiated by central inspiratory signals from the PreBötzinger complex.

*Nozomu Nakamura[1], Hidemasa Furue[2], Kenta Kobayashi[3], Yoshitaka Oku[1] ([1] *Department of Physiology, Hyogo College of Medicine*, [2] *Department of Physiology, Hyogo College of Medicine*, [3] *Section of Viral Vector Development, National Institute of Physiological Sciences*)

There is growing evidence that memory performance is modulated by timings of respiratory cycles. We previously showed that performance accuracy was decreased when the onset of inspiration emerged in the middle of memory processing. To elucidate a temporal role of central inspiratory signals during memory consolidation, we used the optogenetic manipulation in genetically modified mice and controlled *in vivo* activation of the PreBö tzinger complex (PreBö tC), which is the primary inspiratory rhythm generator in the medulla oblongata. Regarding object recognition memory, novel object detection was impaired during a retrieval process, even though 67% of object exploration time was covered with PreBö tC-induced apnea during an encoding process. However, since object exploration in rodents may partially contain their olfactory strategies, we then designed a new paradigm of non-olfactory fear conditioning. Surprisingly, the mice with PreBö tC-induced apnea during an encoding process did not freeze during conditioned stimuli of fear (CS+). Immediate-early gene *Arc* catFISH method showed that the positive cell populations in CA3 of the hippocampus were decreased during conditioned inhibition (CS-) in wild-type controls, suggesting cell-wide inverse synaptic tagging. Meanwhile, PreBö tC-induced apnea during an encoding process did not alter the population during CS- and CS+. Rather, this apnea caused more cells co-expressed Arc between CS- and CS+, supporting a deficit of associative memory. The current study shows that central inspiratory signals from the PreBö tC are required for CA3 synaptic tagging and subsequent memory consolidation. These findings would contribute to understanding mind-body interactions underlying mechanisms to cope with stress and maintain successful performance.

### Public Symposium 9

### [SY09]
### Up-to-date of physiology and pathophysiology of smooth muscle

March 16(Wed), 15:45 - 17:45 Room I

### [SY09-01]
### Dysmotility of gastrointestinal smooth muscles through $Ca^{2+}$ sensitization/desensitization pathways

*Masumi Eto[1] ([1]*Okayama University of Science*)

Dysmotility of gastrointestinal smooth muscles is a cause of malfunctions of the digestive tract. Accumulating lines of evidence suggest links between gastrointestinal dysmotility and perturbations in phosphorylation signaling for myosin regulatory light chain (RLC20). RLC20 phosphorylation is defined by balanced RLC20 kinase (MLCK) and RLC20 phosphatase (MLCP) activities. Changes in the kinase/phosphatase activities lead to an increase and a decrease in the $Ca^{2+}$ sensitivity of RLC20 phosphorylation. In tonic smooth muscles, such as artery and airway, we have shown that GPCR stimulation activates PKC and ROCK leading to phosphorylation of CPI-17, inhibition of MLCP and an elevation in $Ca^{2+}$ sensitivity "$Ca^{2+}$ sensitization". By sharp contrast, "$Ca^{2+}$ desensitization" occurs through delayed inactivation of MLCK through CaMKKβ signaling in phasic muscles including gastrointestinal smooth muscles. In the presentation, pathophysiological significances of $Ca^{2+}$ sensitization/desensitization pathways, plus recent findings of ROCK signal disturbance in gastric dysmotility will be discussed. No COI

### [SY09-02]
### Tangled? Complex and thus potentially specific therapeutic targets of hypertension.

*Ko Momotani[1], Kumiko Sakai[1] ([1]*Sanyo-Onoda City University, Faculty of Pharmaceutical Sciences*)

Recent recognition of specific RhoA GTP exchange factors (GEFs) unique to vasoconstriction, governed by vascular smooth muscle (VSM) contraction/relaxation, sheds novel light on its peculiar regulatory mechanisms. One of its canonical pathways is the $Ca^{2+}$-(de)sensitization initiated by multiple agonists activating G protein-coupled receptors (GPCRs). Activation of GPCRs is transmitted to the downstream effectors; cascaded activations of RhoA, Rho Kinase (ROCK), and inhibitory phosphorylation of Myosin light chain (MLC) phosphatase had been well studied. Inhibition of MLC phosphatase increases net MLC phosphorylation and thus induces VSM contraction. On the other hand, the upstream player(s) between GPCRs and RhoA in this pathway had not been known until researchers newly narrowed down the GEFs, filling these missing pieces. Here, we present a brief history of how this missing step has been elucidated and the resulting impact on the understanding of vascular physiology. A good example is leukemiaassociated RhoGEF (LARG) in salt-dependent hypertension reported by the Offermanns group. The other is p63RhoGEF, first identified by our group. Unpublished data suggest activating another GEF, p63RhoGEF, first identified by our group, triggering vasoconstriction in response to increased internal pressure of small vessels. This observation mimics the vascular tone and suggests p63RhoGEF's contribution to blood pressure regulation. Illumination on multiple GEFs raises an intuitive question asking why an array of GEFs is there, leading to a single outcome, vasoconstriction, in the end. One of the current views suggests that each GEF is responsible for a specific physiological and possibly pathological cue. In other words, multiple GEFs are backing diversity in physiological responses in vasculatures. If this hypothesis stands, it opens the doors to develop specific drags each for a specific pathophysiological condition, such as hypertension, by targeting a particular GEF while circumventing unwanted side effects.

### [SY09-03]
### The therapeutic potential of ω-3 fatty acid for the treatment of pulmonary hypertension

*Lin Hai Kurahara[1], Keizo Hiraishi[1], Katsuya Hirano[1] ([1]*Kagawa University, School of medicine*)

Pulmonary hypertension (PH) is characterized by a progressive increase in pulmonary vascular resistance due to pulmonary arterial vasoconstriction and vacular remodeling. The advancement of drug therapy, including prostacyclin analogue, endothelin receptor antagonist, phosphodiesterase inhibitor and guanylyl cyclase activator, has significantly improved the prognosis of PH patients. However, saving severely ill patients requires further innovation of therapeutic strategy that targets both vasoconstriction and vascular remodeling. Eicosapentaenoic acid (EPA), one of ω-3 fatty acids, has been reported to mitigate cerebral vasoconstriction after subarachnoid hemorrhage and vascular remodeling following balloon injury. We have demonstrated EPA and its metabolite resolvin E1 (RvE1) ameliorated the development of pathophysiology of PH in the rat PH model (Kurahara et al., J Mol Cell Cardiol 148: 50-62, 2020). Mechanistically, they exerted vasorelaxant effect in the human pulmonary artery, inhibited enhanced proliferation of pulmonary arterial smooth muscle cells of the PH patients, prevented the TGF-β2-induced endothelial-tomesenchymal transition in human pulmonary arterial endothelial cells, and inhibited STAT3 signaling in both endothelial and smooth muscle cells. We propose ω-3 fatty acids, such as EPA, to be a promising agent for the treatment of PH.

### [SY09-04]
### The novel signaling pathway to regulate both vasospastic abnormal contraction and cell migration

*Sei Kobayashi[1,2] ([1] *Department of Advanced Preventive Medicine, Yamaguchi University School of Medicine,* [2]*Department of Molecular and Cellular Physiology, Yamaguchi University Graduate School of Medicine*)

The Rho-kinase (ROK)-mediated $Ca^{2+}$-sensitization of vascular smooth muscle contraction plays an important role in the abnormal vasocontractions causing vasospasm. When we discovered the ROK-mediated $Ca^{2+}$-sensitization, we demonstrated that constitutively active ROK can induce the maximal contraction of membrane-permeabilized vascular smooth muscle even in the complete absence of $Ca^{2+}$, buffered with 10mM EGTA. Thus, we started to explore the novel upper stream signaling pathway for ROK without activation of G-proteins. Then, we finally discovered sphingosylphosphorylcholine (SPC)/Fyn tyrosine kinase/paxillin to activate Rho-kinase completely independent of $Ca^{2+}$. Surprisingly this pathway also regulates pathological cell migration. After extensive screening for the specific inhibitors for this pathway, we, fortunately, found several compounds.

### Public Symposium 10

### [SY10]
### Multifaceted perspective of torpor and hibernation in mammals

March 16(Wed), 15:45 - 17:45, Room J

### [SY10-01]
### Approaches with Syrian hamster, a model mammalian hibernator, to molecular mechanisms enabling hibernation

*Yoshifumi Yamaguchi[1] ([1]*Institute of Low Temperature Science, Hokkaido University*)

Hibernation is an adaptive strategy to survive during harsh season with cold and little food by suppressing metabolisms and thermogenesis, thereby being hypothermia for a long period. Mammalian hibernators, including hamsters, squirrels, bats, bears, etc. can endure severe hypothermia, whereas many mammals, including humans, mice, and rats, are damaged by such prolonged hypothermia and cannot hibernate. Mammalian hibernators should possess properties enabling hibernation; tolerance to cold and rewarming stresses, efficient burning mechanism of stored fat, resistance to muscle disuse atrophy, body temperature regulation allowing hypothermia and rewarming. These properties are fascinating from both biological and clinical aspects, but the molecular mechanisms of the properties that enable hibernation are not well understood. We have studied mechanisms of seasonal body remodeling and cold tolerance using Syrian hamsters as a model mammalian hibernator, since they can remodel their bodies from summer-like to winterlike, and hibernate in response to short days and cold under a laboratory condition. In this talk, I will introduce our recent findings on properties enabling hibernation.

### [SY10-02]
### Quantitative analysis for pattern of body temperature fluctuation during hibernation

*Shingo Gibo[1], Yoshifumi Yamaguchi[2], Gen Kurosawa[1] ([1]*Interdisciplinary Theoretical and Mathematical Sciences Program, RIKEN,* [2]*Institute of Low Temperature Science, Hokkaido University*)

Under cold and short photoperiodic conditions, Syrian hamster enters hibernation. During hibernation, their body temperature (Tb) does not remain constant, but shows fluctuation between euthermia and hypothermia with an interval of several days. It is called torporinterbout arousal (IBA) cycle. The rules of torpor-IBA cycles have been unknown. In this study, we aim to reveal a principle governing Tb fluctuation during hibernation by theoretically analyzing the data of Syrian hamster. First, to understand complex and variable patterns of torpor-IBA cycles, we used general harmonic analysis (GHA) which was developed in acoustic engineering field. We found that the period of torpor-IBA cycle gradually changes at hundreds-days scale. Then, to understand biological processes behind gradual change of the torpor-IBA cycle, we identified a simple model which reproduces Tb data during hibernation. By applying statistical analysis methods, we found that the period of torpor-IBA cycles is modulated by longer period of 120-430 days. Quantitative analysis revealed a circannual period in a hibernator thought not to have circannual rhythms.

### [SY10-03]
### Muscle atrophy resistance to prolonged physical inactivity in hibernating animals

*Mitsunori Miyazaki[1] ([1]*Department of Integrative Physiology, Graduate School of Biomedical and Health Sciences, Hiroshima University*)

For all living organisms, adaptation to the internal and external environment is an extremely important task for maintaining individual life and the species. In particular, for hibernating animals, hibernation is considered to be a survival strategy to overcome extreme winter conditions such as food deprivation and cold temperatures. Surprisingly, despite experiencing several months of fasting and physical inactivity, hibernating animals successfully minimize the decline of their physical functions during and following hibernation. In our previous study on hibernating black bears, we found that disuse atrophy of skeletal muscle in hibernating black bears is very limited compared to other animal species, including humans (Miyazaki et al., PLOS ONE 2019). This result suggests the research hypothesis that resistance to disuse syndrome, mainly in skeletal muscle, is developed by some physiological adaptation induced during hibernation in bears. In this symposium, we are presenting the latest research findings in hibernating animals including black bears and Syrian hamsters, focusing on physiological comparisons between the active and hibernation periods. We will also present some data regarding the effects of hibernating bear serum on cultured human skeletal muscle cells. Through this presentation, we would like to discuss the possibility of identifying hibernation-inducible factors for the development of skeletal muscle atrophy resistance and the maintenance of human physical performance, which will contribute to the prevention of human bedriddenness and the effective rehabilitation strategy.

### [SY10-04]
### $Ca^{2+}$ signaling for cold adaptation: temperature compensation of circadian clock

*Naohiro Kon[1], Takahiro Iwamoto[2], Yoshitaka Fukada[3] ([1]*Nagoya University,* [2]*Fukuoka University,* [3]*University of Tokyo*)

Circadian rhythms are generated by transcriptional rhythms based on transcriptional and translational feedback loops (TTFL). Importantly, period lengths of the rhythms are constant even in hibernating animals. Because biochemical reactions are sensitive to temperature, mechanism of the temperature compensation is an attractive issue to understand cellular response for cold adaptation. We screened small-molecule inhibitors by using Rat-1 fibroblasts. The temperature compensation of the transcriptional rhythms was compromised by an inhibitor of NCX or CaMKII. Further analysis revealed that temperature lowering enhances NCX-dependent $Ca^{2+}$ influx to activate CaMKII. CaMKII facilitates heterodimerization of CLOCK and BMAL1, bHLH transcription factors, thereby activating gene expression. Thus, NCX-dependent cold $Ca^{2+}$-CaMKII signaling compensates for deceleration of the TTFL at lower temperature. Importantly, the role of cold $Ca^{2+}$ signaling is conserved among animals, plants, and cyanobacteria, suggesting that the cold $Ca^{2+}$ signaling is an ancestral cold response inherited from a common ancestor of life (Kon *et al.*, ***Genes and Development***, 2014; Kon *et al.* ***Science Advances***, 2021).

### [SY10-05]
### Can a hibernation-like state delay disease progression?

*Genshiro A. Sunagawa[1] ([1]*RIKEN BDR*)

Hibernation is a regulated hypometabolism. Animals lower the basal metabolic rate and exhibit low body temperature as a consequence. How the peripheral tissues tolerate low metabolism during hibernation remains unanswered. When peripheral tissues face a prolonged shortage of oxygen for any reason, the tissue will suffer from hypoxic damage. Shocks and respiratory failures are good examples, although they have distinct etiologies. Modern medicine attempts to recover the impaired oxygen supply when patients suffer from a mismatch of oxygen demand and supply. Hibernators face a shortage of food during winter, making it impossible for the animal to meet the regular energy demand at the peripheral tissues. They overcome such energy shortages by reducing their basal metabolic rate rather than hunting food in the winter. Suppose critically ill patients can reduce their metabolic rate as hibernators. In that case, the illness will recover to some extent because the body will need less oxygen. This talk will share evidence that artificially induced hibernation-like hypometabolism — Q neurons.induced hypometabolism or QIH—can delay disease progression. This will be a proof of concept of the clinical application of hibernation.

### Public Symposium 11

### [SY11]
### Smooth muscle-afferent nerve axis 'Beyond Contractile Machinery'

March 17(Thu), 8:30 - 10:30, Room H

### [SY11-01]
### Comparison of spinal afferent nerve endings in mouse colon and bladder and similarities in their mechanisms of activation?

*Nick John Spencer[1] ([1]*Flinders University*)

Over the past 8 years, our laboratory has focused on identifying the nerve endings of spinal afferent neurons in visceral organs, like the uterus, bladder and colon. This was accomplish using an anterograde tracing technique from dorsal root ganglia *in vivo*, developed in our laboratory. Four distinct morphological types of spinal afferent axons were identified. Three types existed in the detrusor muscle and one major type in the urothelium. In contrast to the bladder, spinal afferent nerve endings in the mouse colon were found to have an unexpectedly diverse and complex array of different types of nerve endings (13 different types, in all anatomical layers). The vast majority of spinal afferent nerve endings in bladder, colon and uterus were peptidergic (immunoreactive to CGRP). Electrophysiological recordings have shown that the vast majority of spinal afferents to the bladder and colon are activated by both low levels of distension and spontaneous dynamic contractions of the muscle layers. Spinal afferents to these organs are primarily low threshold, wide dynamic range sensory neurons that express Trpv1. We discuss here the characteristics of spinal afferent nerve endings in the uterus, colon and urinary bladder and how similar mechanisms of activation appear to underlie their mechanosensitivity.

### [SY11-02]
### Functional analysis of bladder afferent activity in relation to smooth muscle contraction

*Naoki Aizawa[1], Tomoe Fujita[1] ([1]*Dokkyo Medical University*)

Excitation of the mechanoreceptors in sensory nerve endings plays an important role in triggering the micturition reflex. During urine storage phase, the bladder is mechanically stretched, and sensory afferent impulses conveyed by the pelvic afferent nerves (myelinated A$\delta$ -fiber and unmyelinated C-fiber) reach to the central nerves system, whereas the bladder contracts by pelvic efferent nerve excitation during urine voiding phase. We previously confirmed that mechanosensitive afferent activities of both A$\delta$ - and C-fibers of the normal rat bladder are capable of being responsive to both stretch and contractile stimuli, suggesting that the activation of bladder afferent nerve fibers facilitates bladder contractions during the urine voiding phase by providing a sensory input that is used as positive feedback to maintain the contraction of the bladder. Moreover, it has been suggested that bladder myogenic microcontractions or micromotions may partly contribute to the development of urinary urgency (hyperactive bladder sensation). In our results, some of drug (e.g., $\beta$ 3-adrenoceptor agonists) inhibited the bladder afferent activities through the suppression of the bladder myogenic microcontractions in normal or pathophysiological conditions. In this symposium, we show our data obtained by functional analysis of mechanosensitive bladder afferent activities in relation to the bladder contraction.

### [SY11-03]
### Non-locomotive roles of smooth muscle as a trigger of afferent signals

*Hikaru Hashitani[1], Retsu Mitsui[1] ([1]*Nagoya City University*)

Afferent nerves have an 'efferent' function to modulate smooth muscle contractility by releasing neurotransmitters. It is also becoming evident that smooth muscle contractions trigger afferent signals. Thus, in addition to 'locomotive' spontaneous contractions to propel, mix or hold the luminal contents, smooth muscles also develop asynchronous spontaneous contractions. In the bladder, such contractions of detrusor smooth muscles (DSM) mechanically stimulate afferent nerves resulting in the physiological sensation of bladder fullness, while aberrant contractions cause unrainy urgency. The muscularis mucosae (MM), the thin smooth muscle meshwork in the bladder mucosae, is also spontaneously contractile, and could stimulate mucosal afferent nerves. The contractility of both DSM and MM appears to be modulated by humoral substances released from the urothelium, and thus the urothelium, smooth muscles and afferent nerves act as an integrated sensory unit. The smooth muscle-induced afferent signals may function in other smooth muscle organs such as the gastrointestinal tract or blood vessels where afferent nerves are densely distributed, and could represent a novel therapeutic target.

### [SY11-04]
### Urothelial factors that may influence bladder afferent nerve activity indirectly via changes in smooth muscle activity?

*Russ Chess-Williams[1], Donna Sellers[1] ([1]*Bond University*)

The epithelial inner layer of the bladder (urothelium) plays an active role in the sensing of stretch and the presence of neurotransmitters, hormones and paracrine mediators. In response to these stimuli, the urothelium releases a large range of mediators that can influence the activity of sensory nerves directly (eg ATP), but may also affect afferent nerve activity indirectly via the activation or inhibition of contractile activity of the local smooth muscle in the muscularis mucosa and the detrusor. Thus, urothelial-derived ATP, acetylcholine and prostaglandin E2 and F2alpha enhance detrusor contractile activity, whilst urothelial-derived nitric oxide, hydrogen sulphide, prostaglandins I2 and D2 appear to inhibit contraction. A number of other factors (interleukins, growth factors) are also released from the urothelium, but their actions on the contractile activity of the detrusor and muscularis mucosa smooth muscle have yet to be established. In this presentation the evidence to support urothelial influences on bladder contractile activity and ultimately afferent nerve activity will be considered.

### Public Symposium 12

### [SY12]
### Gold anniversary of the discovery of the suprachiasmatic nucleus: Current study of circadian rhythm

March 17(Thu), 8:30 - 10:30, Room I

### [SY12-01]
### Circadian output pathways from the suprachiasmatic nucleus that control sleep and wakefulness

*Daisuke Ono[1] ([1]*Nagoya University*)

The suprachiasmatic nucleus (SCN) plays a crucial role in the timing of physiology and behavior, such as sleep/wakefulness. Anatomically, neuronal outputs from the SCN have been identified, and suggested to be involved in the regulation of sleep and wakefulness. However, it is largely unclear which neuronal pathways are critical for the regulation. Using optogenetics, pharmacogenetics, fiber photometry, and optical imaging with manipulation technique, we firstly identified circadian output pathways from the SCN that control sleep and wakefulness. Circadian regulation of wakefulness is regulated via corticotropinreleasing factor (CRF) neurons in the paraventricular nucleus (PVN) of the hypothalamus to orexin neurons in the lateral hypothalamus. Importantly, neuronal activity of CRF neurons in the PVN is negatively regulated by GABAergic neurons in the SCN. Our findings provide significant insights into circadian regulation of sleep/wakefulness in mammals.

### [SY12-02]
### Critical roles of AVP neurons in the central circadian clock of the SCN

*Michihiro Mieda[1] ([1]*Dept. of Integrative Neurophysiology, Kanazawa Univ.*)

As the central pacemaker in mammals, the circadian clock in the suprachiasmatic nucleus (SCN) is a heterogeneous structure consisting of multiple types of GABAergic neurons. Although individual cells have a molecular clock driven by autoregulatory transcriptional/ translational feedback loops of clock genes, intercellular communication among SCN neurons is essential for the SCN to generate a highly robust circadian rhythm. However, such network mechanisms of the SCN remain unclear. We have been focusing on the roles of arginine vasopressin (AVP)-producing neurons in the SCN shell. Disruption of molecular clocks, specifically in AVP neurons, attenuated circadian behavior rhythm. In addition, the period of molecular clocks in AVP neurons is likely the primary determinant of the ensemble period of the SCN network. Furthermore, GABAergic transmission from AVP neurons regulates the timing of SCN neuronal firing to temporally restrict circadian behavior to appropriate time windows in SCN molecular clocks. Thus, AVP neurons of the SCN may be an essential component for the generation and period-setting of circadian rhythm and the coordination of the time at which the SCN allows the animal' s daily behavior.

### [SY12-03]
### Physiological function of Gastrin-releasing peptide producing neurons in the circadian clock

*Arisa Hirano[1], Ruth Li[3], Ran Inoue[2], Hisashi Mori[2], Takeshi Sakurai[1] ([1]*Faculty of Medicine, University of Tsukuba,* [2]*Faculty of Medicine, University of Toyama,* [3]*International Institute for Integrative Sleep Medicine, University of Tsukuba*)

The suprachiasmatic nucleus (SCN) is known as the master clock responsible for generating circadian rhythms. It is composed of several populations of neurons, characterized by expression pattern of neuropeptides. Compared to arginine vasopressin (AVP)- and vasoactive intestinal peptide (VIP)-producing neurons, gastrin-releasing peptide (GRP)-producing neurons have a smaller population, and the roles they play in regulating circadian rhythm remain largely unknown. In this study, we clarified the role of GRP-producing neurons in the SCN by using *Grp-iCre* knock-in (KI) mice. We traced the neuronal projections of SCN GRP-producing neurons and found that they project mostly to the thalamus and hypothalamus. We next examined the effect of inhibition of GRPproducing neurons on behavioral rhythms. The mice introduced with tetanus toxin light chain (TexLC) in the SCN GRP-producing neurons showed decreased activity during the dark phase and attenuated behavioral rhythmicity. We also examined PERIOD2 (PER2) protein rhythms in the SCN slice prepared from the TexLC expressing mice. Compared to the control mice, the mice expressing TexLC showed decrease in the amplitude of PER2 expression rhythm. From these results, we postulated that the SCN GRP-producing are essential in regulating behavioral rhythm and is also necessary for sustaining SCN rhythmicity.

### [SY12-04]
### Long days restore regular estrous cyclicity in mice lacking circadian rhythms

*Takahiro J. Nakamura[1], Nana Takasu[2], Wataru Nakamura[2] ([1]*Laboratory of Animal Physiology, School of Agriculture, Meiji University,* [2]*Department of Oral-Chrono Physiology, Graduate School of Biomedical Sciences, Nagasaki University*)

Many female mammals have recurring cycles of ovulation and sexual behaviors that are regulated by reproductive hormones and confer reproductive success. In addition to sexual behaviors, circadian behavior rhythms of locomotor activity also fluctuate across the estrous cycle in rodents. Moreover, there is a bidirectional relationship between circadian rhythms and estrous cyclicity since mice with disrupted circadian rhythms also have compromised estrous cycles resulting in fewer pregnancies. In the present study, we assessed whether extending day length, which alters circadian rhythms, streamlines estrous cyclicity in mice. We found that Period (Per) 1/2/3 triple knockout mice, that have disabled canonical molecular circadian clocks, have markedly disrupted estrous cycles. Surprisingly, extending the day length by only 2 hours per day restored regular 4- or 5-day estrous cycles to Per1/2/3 KO mice. Longer days also induced consistent 4-day, rather than 5-day, estrous cycles in wild-type mice. These data demonstrate that extending daytime light exposure could be used for enhancing reproductive success.

### [SY12-05]
### Inputs determining SCN clock outputs

*Rae Silver[1] ([1]*Columbia University*)

We are drowning in explanations of how output signals from central brain clock in the suprachiasmatic nucleus (SCN) serve as drivers of circadian rhythms. We are greatly impressed by multiple lines of evidence showing that the SCN clock is robust against the numerous signals, such as adrenal corticoids or food-derived cues, that synchronize and/or disrupt peripheral clocks. Regarding the circadian timing system from the point of view of endocrinologists however, leads us to evidence that hormonal signals reaching the systemic circulatory system act directly on the subpopulations of neurons that make up the SCN. The evidence to be discussed shows how circulating hormones provide input signals that act directly on the SCN to alter its outputs. This evidence leads to a more informed understanding of how cues originating internally in the body and those derived from the external environment are integrated in the SCN, and provide insight into the function of heterogeneity among SCN neurons.

### Public Symposium 13

### [SY13]
### Navigating the Social World: From Relationships to Empathy

March 17(Thu), 8:30 - 10:30, Room J

### [SY13-01]
### Oxytocin and the neural mechanisms of social bonding, social loss and empathy.

*Larry James Young[1,2] ([1]*Center for Translational Social Neuroscience, Emory University,* [2]*Center for Social Neural Networks, University of Tsukuba*)

Monogamous prairie voles are useful for identifying neural circuit and genetic mechanisms underlying social bonding and empathy. OXT receptor (OXTR) signaling in nucleus accumbens (NAcc) and prefrontal cortex is critical for pair bond formation in voles. OXT links the neural encoding of the identity of the partner with the rewarding aspects of mating through interactions with dopamine and by coordinating communication across a neural network linking social salience with reward. Diversity in OXTR expression in brain contributes to diversity in social behaviors across and within vole species. Genetic polymorphisms in *Oxtr* predict natural variation in OXTR expression in NAcc, which resilience to neonatal social neglect. BAC vole-*Oxtr* transgenic mice reveal that diversity in brain OXTR distribution may be due to transcriptional sensitivity of the *Oxtr* gene to structural variation. OXT signaling in anterior cingulate cortex mediates empathy-based consoling behavior toward a distressed partner in voles. The absence of OXTR signaling in the NAcc after loss of a partner results in depressive-like "grieving" behavior, which may serve to maintain social bonds. Understanding the brain mechanisms of social behavior in animals may improve treatments for social deficits in psychiatric disorders such as autism.

### [SY13-02]
### Neural mechanism of affective empathy

*Hee-Sup Shin[1], Seongwook Kim[1], Minsoo Kim[1], Jinhee Baek[1] ([1]*Institute for Basic Science*)

Empathy, the capacity to recognize and share emotions with others, is crucial for social interaction among social animals. Observational fear is based on emotional congagion, a basic form of affective empathy, and is conserved from rodents to humans. Establishing the observational fear assay in the mouse has opened up an opportunity to study the neural mechanism of affective empathy at the molecular, cellular, and circuit levels. In my presentation, I will try to present recent advances in neurobiology of affective empathy based on the observational fear paradigm in rodents.

### [SY13-03]
### Newly Identified Role of Estrogen Receptor β Expressing Neurons in Social Behavior Neural Network

*Sonoko Ogawa[1] ([1]*University of Tsukuba*)

We have been studying neuroendocrine mechanisms of social behavior by focusing on the role of the two types of estrogen receptors, ERα and ERβ. In contrast to ERα, brain mechanisms of estrogenic regulation mediated by ERβ have not been well understood. Based on our findings of differential distribution of ERβ from ERα with the use of ERβ-RFP transgenic mice, we further investigated function of ERβ expressing neurons in brain regions in the social behavioral neural networks in both sexes of mice. Chemogenetic manipulation as well as fiber photometry recording of neuronal activity in ERβ-cre male mice revealed that ERβ expressing cells in the posterodorsal part of the medial amygdala (MeAPD) might play a role for establishment of sexual preference toward receptive females. Detailed analysis including examination of neuronal projection patterns of ERβ expressing neurons identified differential involvement of MeAPD and downstream brain regions for preference toward receptive females over nonreceptive females and that over males. A separate line of investigation in female mice using credependent pharmacogenetic manipulation of neuronal activity revealed that ERβ expressing cells in the midbrain dorsal raphe nucleus might be involved in inhibitory regulation of female sexual behavior induced by ERα-mediated estrogen action. These findings collectively suggest that ERβ expressing neurons in the social behavioral neural networks may play a significant role for adaptive expression of social behavior in both male and female mice. (Supported by KAKENHI 15H05724 to SO)

### [SY13-04]
### Social memory representation in the hippocampus

*Teruhiro Okuyama[1] ([1]*The University of Tokyo, Institute for Quantitative Biosciences (IQB)*)

For social animals, it is crucial to remember and recognize different conspecific individuals (i.e., having "social memory") and exhibit appropriate social behaviors, such as preference behavior or avoidance behavior, to each individual. In rodents, we can quantify the degree of memory of individuals by the social discrimination behavioral assay since mice naturally tend to spend more time interacting with novel mice rather than familiar mice. Using the behavioral assay, we demonstrated that vCA1 pyramidal neurons in the hippocampus store social memory (social memory engram). Even when the memory seemed lost after long separation periods, optogenetic activation of the social engram can fully restore that social memory.
Additionally, one tiny dissonance in social memory can disrupt the appropriate social behavior, even for humans. Social impairments caused by a genetic mutation, especially those related to the familiarization with other individuals, are commonly exhibited by patients diagnosed with autism spectrum disorder (ASD). In this meeting, we will show our recent findings regarding neural mechanisms underlying social familiarization impairments observed in ASD model Shank3 knockout mice.

### [SY13-05]
### Formation of emotional bond in the early postnatal period examined by physiological changes during physical contact between parents and infants

*Sachine Yoshida[1], Hiromasa Funato[1,2] ([1]*Faculty of Medicine, Toho University,* [2]*International Institute for Integrative Sleep Medicine, University of Tsukuba*)

Physical contact between caregivers and infants encourages infants to form an emotional bond with their caregivers. However, it is not well understood how natural physical contact with parents in daily life has an immediate effect on infants. We examined heart rate responses evaluated from R-R interval (RRI) in the first-year infants during a hug, a common parent-infant physical contact. We found that infants older than four months showed an increased RRI during a hug, indicating reduced heart rates and pronounced parasympathetic activity, while infants younger than four months did not. Younger infants showed the RRI decrease by pressurization derived from a hug, but older infants did not. Decision tree classification suggested that the RRI increase ratio can be predicted if both the infant's age and the head movement type immediately before hugging are known. Such a context-dependent RRI change was apparent both in maternal and paternal hugs, but absent in younger infants and older infants hugged by female strangers. Parents' heart rates were also reduced by hugging their infants. The parent-infant hug may begin to function as a form of communication for around four months.

### Public Symposium 14

### [SY14]
### As yet unexplored crucial aspects of $Ca^{2+}$ signaling: its novel mechanism and functional outcome

March 17(Thu), 16:00 - 18:00, Room F

### [SY14-01]
### Molecular Mechanism Underlying Initiation and Maturation of Heartbeat

*Mitsuhiko Yamada[1], Tomoe Nakamura-Nishitani[2] ([1]*Dept. Mol. Pharmacol., Shinshu Univ. Sch. Med.*, [2]*Dept. Pharmacol., Wakayama Med. Univ. Sch. Med.*)

The heart starts beating at the very beginning of development. First, unsynchronized $Ca^{2+}$ transients emerge in the cardiac crescent and soon evolve to synchronized caudal-torostral beating of the linear heart tube. However, the excitation-contraction coupling (ECC) at this stage significantly differs from the adult one in the degree of involvement of inositol 1,4,5-triphosphate receptors, $Na^{+}$-$Ca^{2+}$ exchangers, and $Ca_V1.3$ vs. $Ca_V1.2$ L-type $Ca^{2+}$ channels (LTCC). In later gestation, the sarcoplasmic reticulum (SR), SR $Ca^{2+}$ pump, and $Ca_V1.2$ LTCC increasingly play an important role in ECC. However, it is likely that ECC at this stage is still distinct from the adult one especially in that the trigger ($Ca_V1.2$ LTCC) and the source (SR) of the $Ca^{2+}$-induced $Ca^{2+}$ release (CICR) are separately regulated by receptor-mediated beta-arrestin and $G_{q/11}$ signaling, respectively. After birth, ventricular myocytes elaborate SR, a sarcolemmal invagination t-tubules, and their junctions to ensure the strong power stroke enabling the active extrauterine life. In this stage, both the trigger and source of CICR are regulated in an integrated fashion by a sole beta-adrenergic receptor/$G_s$ signaling driven by the newly emerging sympathetic nerve. This talk will introduce our recent findings on molecular mechanisms underlying these processes of cardiac ECC development.

### [SY14-02]
### Reconstitution of skeletal muscle excitation-contraction coupling: toward understanding of molecular mechanism and related diseases.

*Takashi Murayama[1] ([1]*Juntendo Univ.*)

In skeletal muscle excitation-contraction coupling, depolarization of transverse tubule membrane causes conformational change in dihydropyridine receptor (DHPR), which in turn opens type 1 ryanodine receptor (RyR1) to release $Ca^{2+}$ from sarcoplasmic reticulum. This 'depolarization-induced $Ca^{2+}$ release' (DICR) occurs through a supramolecular complex composed of several proteins including RyR1, Cav1.1, β1a, Stac3, and junctophilin. However, it remains so far unclear about molecular mechanism of DICR, especially conformational change in RyR1. Recently, successful reconstitution of DICR was reported by co-expressing these essential components in tsA201 cells. In this study, we developed a high-throughput platform of reconstituted DICR in HEK293 cells. Baculovirus infection of essential components into HEK293 cells expressing RyR1 greatly increased their transduction efficiency, and fluorescent ER $Ca^{2+}$ indicator (R-CEPIA1er) quantitatively measured $Ca^{2+}$ release without contaminant of $Ca^{2+}$ influx. We demonstrated $[K^{+}]_o$-dependent $Ca^{2+}$ release by chemical depolarization in the baculovirus-infected cells, indicating a successful reconstitution of DICR. Our high-throughput platform will accelerate elucidation of molecular mechanism and related diseases in DICR.

### [SY14-03]
### Inositol 1,4,5-trisphosphate receptors in cardiovascular development and diseases

*Keiko Uchida[1,2] ([1]*Keio University School of Medicine*, [2]*Keio University Health Center*)

The inositol 1,4,5-trisphosphate receptors of three subtypes ($IP_3R1$, 2, and 3) are intracellular $Ca^{2+}$ channels, releasing $Ca^{2+}$ from the $Ca^{2+}$ store to regulate various vital processes including cardiovascular physiology and pathology. During embryogenesis, the dynamic and complementary expression patterns of three subtypes are observed in the heart from the early stage. Although single subtype-knockout mice show no developmental disorders, $IP_3R1$ and $IP_3R3$ double knockout mice show developmental defects of outflow tract region of the heart associated with abnormality of Mef2C.Smyd1 signaling pathway, while $IP_3R1$ and $IP_3R2$ double knockout mice show defects of the atrioventricular canal and ventricular myocardium of the heart due to disruption of the calcineurin/NFAT signaling. In development of the pulmonary artery (PA), $IP_3R2$ is expressed specifically in the PA smooth muscle cells (PASMC), and $IP_3R2$-LacZ mice, harboring the LacZ reporter gene inserted at the initiation site of $IP_3R2$ locus, are useful for visualization of PA development. Moreover, $IP_3R2$ knockout exacerbated chronic hypoxia-induced pulmonary arterial hypertension, by suppression of apoptosis and enhancement of store-operated $Ca^{2+}$ entry in the PASMC. The multiple pivotal roles of $IP_3Rs$ for intracellular signals in the processes of development and diseases in the heart and vessels will be discussed.

### [SY14-04]
### Gene regulation of excitation-contraction coupling initiated by cell-cell fusion of skeletal myogenesis

*Taichiro Tomida[1], Kimitaka Yamaguchi[1], Yoshinori Mikami[1], Daisuke Ohshima[1], Satomi Adachi-Akahane[1] ([1]*Department of Physiology, School of Medicine, Faculty of Medicine, Toho University*)

Control of muscle contraction is a conserved role of $Ca^{2+}$ in the muscle, although the mechanism in regulating $Ca^{2+}$ signaling varies considerably depending on the structural and functional property of muscle types. In the skeletal muscle, E-C coupling machinery characterized by voltage-induced $Ca^{2+}$ release is acquired during myogenesis, in which mono-nucleated muscle progenitor cell is differentiated into the elongated muscle fibers containing multiple nuclei. The most striking feature of skeletal muscle is its regenerative capability in adults. Recently discovered transmembrane protein Myomixer (Mymx) initiates myogenesis by executing cell-cell fusion at either embryonic development or adult muscle regeneration. However, the physiological significance of Mymx-dependent cell fusion on muscle formation is not clear. In this study, we elucidated the regulation of Mymx gene and Mymx-dependent gene expression to understand the underlying mechanism and the physiological importance of cell-cell fusion on myogenesis. Using in vitro myogenesis cell model, we demonstrated that the activity of p38 MAPK is responsible for the induction of Mymx gene expression and the myoblast fusion. By comparing the transcriptome of Mymx- KO cells and Mymx-rescued cells, we found that a set of genes related to EC coupling are upregulated in association with cell-cell fusion. We thus identified a novel regulatory link between cell-cell fusion and EC coupling, which is responsible for skeletal myogenesis.

### Public Symposium 15

### [SY15]
### Exploring Pathophysiological Mechanism of Organ Fibrosis

March 17(Thu), 16:00 - 18:00, Room G

### [SY15-01]
### Doxorubicin (DOX) Induces Fibrotic Change in Cardiac Fibroblasts

*Masanari Umemura[1], Narikawa Masatoshi[2], Fumina Suzuki[1], Hiroko Nemoto[1], Akane Nagasako[1], Rina Nakakaji[1], Yoshihiro Ishikawa[1]
(*[1]Cardiovascular Research Institute, Yokohama City University Graduate School of Medicine, [2]Department of Medical Science and Cardiorenal Medicine, Yokohama City University Graduate School of Medicine*)

**Introduction** Doxorubicin induced heart failure has a poor prognosis. Principal mechanisms of cardiotoxicity were considered as oxidative stress and apoptosis in cardiomyocytes. However, little information is available regarding the effect of DOX on cardiac fibroblasts. We investigated the effect of DOX on the function of human cardiac fibroblasts.
**Materials and Methods** Real.time PCR (RT-PCR) and Western blotting analysis were performed. Interleukin.6 (IL-6) production was measured by RT.PCR and ELISA. MitoTracker Red was used to analysis mitochondrial membrane potential. Reactive oxygen species was measured by electron spin resonance (ESR).
**Results** DOX enhanced $\alpha$-SMA protein (a marker of trans.differentiation) in HCFs culture cells, not in normal human dermal fibroblast. These results indicated that DOX induced the trans-differentiation of HCFs into myofibroblasts. DOX increased IL.6 via transforming growth factor-$\beta$ (TGF-$\beta$)/Smad pathway. DOX also increased the mRNA and protein expression of matrix metalloproteinase.1 (MMP-1). Furthermore, DOX induced the mitochondrial damage and increased the expression of Interleukin.1 (IL-1). ESR showed that DOX rapidly increased ROS production. Animal study showed that DOX (4 mg/kg/week, *i.p.*) for 3 weeks enhanced the perivascular fibrosis of mice heart (n=4). A peroxisome proliferator.activated receptor gamma (PPAR $\gamma$) agonist, pioglitazone hydrochloride attenuated the expression of $\alpha$-SMA. Pioglitazone attenuated DOX.induced early fibrotic response in heart of mice.
**Conclusion** These findings suggested that DOX induced reactive fibrotic change of cardiac fibroblasts. There may be potentially novel mechanisms of DOX.induced cardiotoxicity.

### [SY15-02]
### PDGF-CaSR signaling on pulmonary vascular remodeling in pulmonary arterial hypertension

*Aya Yamamura[1] (*[1]Department of Physiology Aichi Medical University*)

Pulmonary arterial hypertension (PAH) is a progressive and fatal disease in the pulmonary artery. The major pathogenesis is sustained pulmonary vasoconstriction and pulmonary vascular remodeling, which are largely mediated by an elevated cytosolic $Ca^{2+}$ concentration in pulmonary arterial smooth muscle cells (PASMCs). We have previous demonstrated that the expression of the $Ca^{2+}$-sensing receptor (CaSR) is upregulated in PASMCs from idiopathic pulmonary arterial hypertension (IPAH) patients, contributes to enhanced $Ca^{2+}$ signaling, and results in pulmonary vascular remodeling. In this study, the molecular mechanisms underlying the upregulation of CaSR expression were examined in PASMCs from normal subjects and IPAH patients. In normal- PASMCs, the expression of CaSR was upregulated by platelet-derived growth factor (PDGF) stimulation, which is known as a pathological signal associated with IPAH. The expression of PDGF receptors was higher in IPAH-PASMCs than in normal-PASMCs. PDGF-induced activation of PDGF receptors and its downstream molecules (ERK1/2, p38, Akt, and STAT1/3) sustained longer in IPAH-PASMCs. In addition, PDGF stimulation facilitated both proliferation and migration of normal-PASMCs. On the other hand, siRNA knockdown of PDGF receptors attenuated the upregulation of CaSR expression in IPAH-PASMCs. Imatinib (a tyrosine kinase inhibitor of PDGF receptors) and NPS2143 (an antagonist of CaSR) blocked the PDGF-induced CaSR upregulation in IPAH-PASMCs. Our results strongly suggest that PDGF signal activates the upregulation mechanism of CaSR in IPAH-PASMCs. In conclusion, PDGF-CaSR signaling is a novel pathological mechanism contributing to pulmonary vascular remodeling in PAH. This finding may provide a novel therapeutic target for PAH.

### [SY15-03]
### The roles of gap junctional intercellular communication in nonalcoholic steatohepatitis (NASH) and liver fibrosis

*Aya Naiki-Ito[1] (*[1]Department of Experimental Pathology and Tumor Biology, Nagoya City University Graduate School of Medical Sciences*)

Non-alcoholic steatohepatitis (NASH) is a common risk factor for fibrosis, cirrhosis, and a predisposing factor for the development of hepatocellular carcinoma. Increase of incidence of NASH has become as a major worldwide public health problem. Connexin (Cx) 32, a hepatocyte gap-junction protein, plays an important role in liver tissue homeostasis. However, the precise contribution of Cx32 in the development of NASH and fibrosis has not been established. Therefore, roles of Cx32 on NASH and fibrosis was explored by using Cx32 dominant negative transgenic (Cx32ΔTg) rat model. Extensive fibrosis and cirrhosis were evident in Cx32ΔTg as compared to wild-type (Wt) rats; the developing fibrous septa were extended not only from the portal area to the centrilobular zone but also to adjacent portal tracts. Elevation of reactive oxygen species, inflammatory cytokine expressions (*Tnfα*, *Il6*, *Tgfβ*, *Il1β*, *Timp2* and *Col1a1*), and NF-κB activation were clearly induced in Cx32ΔTg rats and these changes and fibrosis was suppressed by some antioxidants. These results suggest that oxidative stress induced by Cx32 dysfunction contributes to fibrogenic remodeling in the liver. We would like to discuss about molecular mechanisms and chemoprevention of NASH and liver fibrosis.

### [SY15-04]
### Exploring mechanisms of renal fibrosis progression

*Daisuke Nakano[1] (*[1]Department of Pharmacology, Kagawa University*)

Kidney is the organ that has one of the most complicated architecture in the body. The sophisticated architecture of nephron made its physiology difficult to unravel and the variety for experimental approach for renal (patho)physiology is limited so far. At this symposium, I would like to discuss the recent progress in our understanding of renal fibrosis, including microenvironment around peritubular capillaries and the tubules. Renal peritubular capillary plays a role in many biological functions, including supplying oxygen to tubular and interstitial cells and recycling reabsorbed electrolytes, glucose, and amino acids. We focus on the transportation and subsequent phenotype changes of cells in this environment, which play important roles in the balance between recovery versus development of fibrosis after renal acute kidney injury. Another hand, one of the biggest problem on the anti-renal fibrotic drug development is lacking the good animal model. While there are several models for rodents, each model has limitation to develop broad fibrotic changes in the kidney, such as high mortality and skewing the degree of fibrosis. We developed a model makes easier to design the experimental protocol for assessing the therapeutic potential of the specific drug being developed for the purpose of treating renal fibrosis. I would like to discuss what we should do for beating the organ fibrosis.

### Public Symposium 16

### [SY16]
### The CNS-adipose network for the adaptation to the thermal environment and metabolic diseases

March 17(Thu), 16:00 - 18:00, Room H

### [SY16-01]
### Induction of hibernation-like hypometabolic state by manupulating hypothalamic neurons

*Takeshi Sakurai[1] ([1]*Faculty of Medicine, University of Tsukuba*)

Animals in hibernation are in a state of hypothermia, hypometabolism, and low activity, but even under these conditions, they can adapt to changes in the environment and spontaneously return to their original state without any tissue damage. If the oxygen demand of animals could be safely lowered as in hibernating animals, various applications are possible. We recently found that chemogenetic/optogenetic excitation of Qrfpexpressing neurons (Q neurons) in a region of the mouse hypothalamus (anterior ventral periventricular nucleus) induces sustained hypothermia and hypometabolism, which we named QIH. The QIH was accompanied by a significant decrease in body temperature and oxygen consumption rate. A battery of behavioral tests was performed on the QIHexperienced and QIH-naïve groups, but no differences were found between the two groups, nor were there any differences in histological observations of the brain, heart, muscles, or other organs. The fact that QIH can be repeated in the same individual suggests that QIH is a reversible and safe hypometabolic state, i.e., a hypometabolic state similar to hibernation. Histological and photogenetic analyses suggested that Q neurons operate mainly through the DMH. QRFP is widely conserved in mammals, suggesting that Q neurons may be a hypometabolism-inducible neural pathway that is widely conserved in mammals. Physiologically, Q neurons may be involved in rapid shifts in body temperature set points and have been shown to be involved in circadian control of body temperature.

### [SY16-02]
### Cold sensitivity and thermosensitive TRP channels

*Makoto Tominaga[1,2] ([1] *Div. Cell Signaling, National Institute for Physiological Sciences,* [2] *Thermal Biology Group, Exploratory Research Center on Life and Living Systems*)

Cold sensing ability is very important to maintain our body temperature properly. However, body temperatures have to be kept in the cold range in the specific conditions such as hibernation. Sensory neurons detect cold ambient temperature with specific ion channels activated by temperature changes, so called thermosensing TRP channels. There are now 11 thermosensitive TRP channels (TRPV1, TRPV2, TRPV3, TRPV4, TRPM2, TRPM3, TRPM4, TRPM5, TRPM8, TRPC5, TRPA1), and only TRPM8 and TRPC5 were clearly shown to have cold sensitivity. Although rodent TRPA1 was initially reported as a noxious cold sensor in 2003, the temperature sensitivity of mammalian TRPA1 is now quite controversial. Detection of high brain temperature with TRPM2 was reported to drive hypothermia. The 2021 Nobel Prize Physiology or Medicine was awarded to David Julius for his discovery of heat receptor TRPV1 and cold receptor TRPM8. I will talk about the current understanding of cold sensitivity with some TRP channels.

### [SY16-03]
### Identification of a phosphatase that integrates a histone demethylase and YAP mediated actomyosin pathway to orchestrates uncoupled respiration in beige adipogenesis

*Juro Sakai[1,2], Hiroki Takahashi[1], Ge Yang[1], Takeshi Yoneshiro[2], Chaoran Yang[1], Ryo Ito[1], Yoshihiro Matsumura[2] ([1]*Division of Molecular Physiology and Metabolism, Tohoku University Graduate School of Medicine,* [2]*Division of Metabolic Medicine, RCAST, The University of Tokyo*)

Beige adipogenesis requires changes in epigenetic status and transcriptional cascades under chronic cold stress. We recently showed that histone demethylase JMJD1A induces beige selective gene inductions through a protein kinase A-mediated JMJD1A phosphorylation (1$^{st}$ step) and the following demethylation of a repressive histone mark H3K9me2 from these gene loci to activate transcription of a thermogenic *Ucp1* by JMJD1A (2$^{nd}$ step). However, the phosphatase of phospho- Ser265-JMJD1A has not been identified. Here, we show that the myosin light chain phosphatase complex (MYLP) is a phosphatase of phospho-JMJD1A. Depletion of either MYPT1 (a regulatory subunit) or PP1 β (catalytic subunit) increased Ser265 phosphorylation of JMJD1A, decreased H3K9me2 modification on thermogenic gene loci, and induced thermogenic genes. In addition, MYPT1-PP1 β also targets phosphorylation of the myosin regulatory light chain (RLC) to regulate *Ucp1* transcription through the modulation of the actomyosin tension mediated TAZ/YAP pathway. Importantly, the thermogenic gene induction by MYPT1 depletion is hardly observed in preadipocytes expressing catalytically inactive JMJD1A mutant, indicating that changes in chromatin architecture by JMJD1A mediated demethylation preceded to actomyosin-YAP/TAZ mediated gene induction. In addition, β-adrenergic stimulation downstream to cold stress phosphorylated MYPT1 at T694, which reportedly inhibits PP1 β activity by acting as a pseudosubstrate. Thus, MYPT1 / PP1 β phosphatase complex, regulated by cold stress, coordinately regulates histone demethylation step and subsequent actomyosin mediated thermogenic gene inductions for beige adipogenesis.

### [SY16-04]
### NFIA controls the brown-fat-specific gene program and energy homeostasis

*Hironori Waki[1] ([1]*Department of Metabolism and Endocrinology, Akita University Graduate School of Medicine*)

Brown adipocytes dissipate energy in the form of heat in response to environmental changes and attract attention as a therapeutic target for obesity. We recently conducted an epigenetic analysis of brown adipocytes and discovered that a transcription factor NFIA plays an important role in controlling the brown-adipocyte-specific gene program (1,2). Mechanistically, NFIA facilitates the recruitment of the adipogenic master regulator PPAR γ to the brown-adipocyte-specific enhancers. NFIA knockout mice exhibited impaired expression of the brown-adipocyte-specific genes and inappropriate upregulation of muscle genes in adipose tissue but die within a week after birth due to neurological deficit. To circumvent such lethality, we created adipocyte-specific NFIA transgenic and knockout mice. We observed expression of genes specific to brown adipocytes, genes involved in lipid metabolism and oxidative phosphorylation, and inflammation was changed in white adipose tissues of adult mice under a high-fat diet. The transgenic mice were protected from obesity under a high-fat diet compared with the controls while the knockout mice were prone to obesity, suggesting NFIA is a critical factor that maintains energy homeostasis under a high-fat diet. Recent epigenome-wide analysis of human body mass index found DNA methylation of the NFIA gene is strongly associated with obesity (3). Manipulation of NFIA may be useful to develop a new strategy to treat obesity.

1. Nat Cell Biol 19(9):1081-1092, 2017
2. PLoS Genet. 16(9):e1009044, 2020
3. Nature. 541(7635):81-86, 2017

### Public Symposium 17

### [SY17]
### Roles of glucose metabolism in neural activity under physiological and pathological conditions

March 17(Thu), 16:00 - 18:00, Room I

### [SY17-01]
### Exogenous pyruvate maintains glycolysis-TCA cycle flux through PARP-dependent and independent cascades under high-glucose condition

*Hideji Yako[1], Sango Kazunori[1] ([1]*Tokyo Metropolitan Institute of Medical Science*)

We observed that starvation of extracellular pyruvate under high-glucose conditions disturbed glycolysis-TCA cycle flux, leading to rapid and massive IMS32 Schwann cell death; however, the precise mechanisms of how exogenous pyruvate maintains the flux remain unclear. Poly (ADP-ribose) polymerase (PARP) consumed NAD to mediate poly ADP-ribosylation of the target proteins. Excessive activation of PARP induced by oxidative stress depleted NAD and ATP levels, leading to cell death. Poly ADP-ribosylation catalyzed by PARP lowered GAPDH activity under high-glucose conditions, resulting in the inhibition of glycolytic flux. Therefore, the involvement of PARP in the cell death under high-glucose pyruvate-starved conditions defines the aim of this study. Administration of rucaparib, a PARP inhibitor, to Schwann cells under those conditions prevented decreases in cell viability, GAPDH activity, glycolytic flux, and NAD level, but not decreases in pyruvate dehydrogenase activity and mitochondrial respiration. These findings suggest that exogenous pyruvate plays a pivotal role in maintaining glucose metabolism through PARPdependent (glycolysis) and independent (TCA cycle) cascades under high-glucose conditions.
COI: Sumitomo Dainippon Pharma Co., Ltd.

### [SY17-02]
### Pathogenesis of diabetic neuropathy induced by blood glucose fluctuation and hypoglycemia: oxidative stress and mitochondrial dysfunction

*Ayako Kato[1], Koichi Kato[1] ([1]*Laboratory of Medicine, Aichi Gakuin University, School of Pharmacy*)

It is known that oxidative stress is a major cause of the onset and progression of diabetic neuropathy. One of the mechanisms of hyperglycemia-induced oxidative stress is associated with increased mitochondrial ROS. It has been reported that an increase in mitochondrial ROS due to chronic hyperglycemia is involved in the development of diabetic complications in endothelial cells, and that the increase in mitochondrial ROS due to hyperglycemia also causes insulin resistance in pancreatic beta cells. Our data demonstrated that not only constant high glucose, but also glucose fluctuation and hypoglycemia enhanced oxidative stress and induced cell death in immortalized adult mouse Schwann (IMS32) cells. Moreover, as a mechanism for this increased oxidative stress, mitochondrial ROS were induced not only by short-term high glucose, but also by short-term low glucose. These findings suggest that mitochondrial dysfunction due to increased mitochondrial ROS caused by hypoglycemia and blood glucose fluctuations might cause nerve deficits in diabetes, leading to the onset and progression of diabetic neuropathy.

### [SY17-03]
### Enhancement of glucose uptake protects neurons against energetic stresses in aging and disease

*Mikiko Oka[1], Kanae Ando[1] ([1]*Tokyo Metropolitan University*)

Brain functions are supported by the systemic supply of glucose and its metabolism in neurons and glia. Aging and pathological conditions reduce glucose delivery to the brain and compromise pathways of energy production. However, how they affect cellular ATP levels, organismal aging and disease pathogenesis are not fully understood. We recently reported that neuronal glucose uptake is critical for maintenance of ATP levels during aging. By using ATP imaging in the *Drosophila* brain, we found that ATP levels in neurons, decline during aging. Increasing glucose uptake by neuron-specific expression of glucose transporter was sufficient to counteract agedependent declines in ATP and improved locomotor functions in aged animals. We also found that increasing glucose uptake protects against neurodegeneration in a fly model of tauopathy. Glucose transporter overexpression suppressed degeneration of photoreceptor neurons induced by human tau. Production of toxic cytokines and phagocytosis by glia were attenuated, suggesting glucose metabolism regulates glial inflammatory responses. Our results suggest that efficient glucose utilization counteracts organismal aging and disease conditions.

### [SY17-04]
### Motor system dysfunction caused by diabetes mellitus

*Ken Muramatsu[1] ([1]*Dept Physical Therapy, Kyorin University*)

Although patients with diabetes, especially those with diabetic neuropathy (DN), can have several motor dysfunctions, such as increased risk of falling, altered gait and balance, and increased body sway, the effects of diabetes on the motor system are thought to be less than those on the sensory system. A main symptom of DN is a diffuse peripheral disorder with "stocking and glove" loss of sensation despite relatively preserved motor function. Additionally, in contrast to primary afferent neurons, it was believed that motor system is protected from DN because they are located within the central nervous system, which is relatively protected from hyperglycemic conditions by the blood-brain barrier. Therefore, motor deficits are considered to be only due to sensory and muscle impairment. However, our recent experimental studies have revealed that the motor cortex, corticospinal tract and spinal motoneurons, which are involved in the motor control of voluntary movements, are also affected by DN. In this symposium we focus on the cortico-muscular pathways, such as corticospinal tract and spinal motoneuron abnormalities. Specifically, axonal damage occurs in the corticospinal tract and motoneurons with long axons, impairing the transmission of motor commands from the brain to the muscles. These findings provide a new perspective for explaining motor deficits in humans with diabetes.

### Public Symposium 18

### [SY18]
### Physiology of hyper-adaptability; from the higher brain function to the reconstruction of motor function after brain damage

March 18(Fri), 8:30 - 10:30, Room H

### [SY18-01]
### Cortical contributions to the adaptation of gait in the cat

*Trevor Drew[1] ([1]*Universite de Montreal*)

In my presentation I will review work from my laboratory showing the importance of different cortical regions to the control of visually-guided locomotion. I will focus on the results from experiments in which cats walk on a treadmill and must use vision to adapt gait in order to negotiate an obstacle that is attached to the treadmill. I will concentrate on three different cortical regions that are involved in the planning and execution of this behavior: the primary motor cortex (Brodmann's area 4); the posterior parietal cortex (area 5); and the premotor cortex (area 6). Each of these areas makes a particular contribution to visually-guided gait adaptations. The motor cortex is responsible for producing the changes in muscle activity that appropriately modify limb trajectory in each limb as the cat steps over the obstacle. Area 5 provides a global signal that provides the location of the obstacle with respect to the body, while our recent work suggests that area 6 contributes to the sensorimotor transformation of this global signal into the muscle-related signals that are found in the motor cortex.

### [SY18-02]
### Global disinhibition associated with recovery of hand movements after spinal cord injury

*Reona Yamaguchi[1], Toshinari Kawasaki[2,3], Zenas Chao[2,5], Masahiro Mitsuhashi[2,4], Satoko Ueno[2], Tadashi Isa[1,2,6] ([1]*Institute for the Advanced Study of Human Biology (WPI-ASHBi), Kyoto Univ,* [2]*Dept Neuroscience, Grad Sch of Med, Kyoto Univ,* [3]*Dept of Neurosurgery, Grad Sch of Med, Kyoto Univ,* [4]*Dept of Neurology, Grad Sch of Med, Kyoto Univ,* [5]*International Research Center for Neurointelligence (IRCN), Univ of Tokyo,* [6]*Human Brain Research Center, Grad Sch of Med, Kyoto Univ*)

Recent studies revealed that a variety of brain areas, which are not involved in the normal motor control, become activated and contribute to motor recovery from brain and spinal cord injury. Such a remote effect of a neuronal injury to the areas which are indirectly connected with it is classically called "diaschisis", and is considered to underlie the recovery from the neuronal injury. However, the fundamental mechanism is still elusive. Here, we propose "global disinhibition" which releases the workspace for re-learning of motor control strategies to recruit novel areas for recovery. In this study, extensive cortical electrical stimulation through each electrocorticography electrodes was tested to assess the connectivity between motor cortex and motoneurons before and after sub-hemisection at C4/C5 in two macaque monkeys. Muscle twitch responses of affected hand and arm gradually spread from the proximal to distal muscles including digits from the contralesional PM/M1 after the lesion and became inducible also from ipsilesional PM/M1. These results suggested that disinhibition occurred widely in bilateral PM/M1 after the lesion, which may underlie the recovery process.

### [SY18-03]
### A Control Model incorporating Physiological Knowledge for Elucidating Postural Control in Human

*Ryosuke Chiba[1], Kohei Kaminishi[2], Yuichiro Omura[2], Hitohiro Etoh[2], Jun Ota[2], Kaoru Takakusaki[1] ([1]*Asahikawa Medical University,* [2]*The University of Tokyo*)

In recent years, the constructivist approach has become popular to elucidate physiological mechanisms in human. In other words, this approach can be described as a "construct and understand" approach. This methodology utilizes the control engineering and the systems engineering methods to construct the human body and the neural controllers which reproduce human behavior under various conditions computationally and lead to physiological hypotheses for humans. However, not a few computational models are only representations of human behavior on the computer and cannot be understood as entities, because these models are constructed in "black box". As a result, these models can be utilized for only predictive simulations, not for human understandings including the hyperadaptation. We introduce our studies which utilized a detailed musculoskeletal model to construct a proper neural controller that incorporates physiological knowledge for postural control in "grey box" and the controller reflects the physiological knowledge and its significance can be understood. And, we will discuss the further incorporation of physiological knowledge and prospections for their applications.

### [SY18-04]
### Adaptive change of body-specific attention to paretic hand in stroke patients

*Naoki Aizu[1], Ryoji Otaki[2], Kouji Yamada[1], Shin-ichi Izumi[2] ([1]*School of Health Siences, Faculty of Rehabilitation, Fujita Health University,* [2]*Department of Physical Medicine and Rehabilitation, Tohoku University Graduate School of Medicine*)

The brain generates a motor program to induce body movement. It directs attention to the body, regulating information from effectors to support motor control. Previous research showed that visual detection task can measure the body-specific attention. Patients with stroke-induced paralysis have greatly reduced quality of life. Using a paralyzed limb in rehabilitation improves motor function and enables daily living activities. Reports on how patients direct body-specific attention to paretic limbs are unclear. Here, body-specific attention to paretic hand is lowest at 2 weeks after onset and peaks at 1 month when motor function improvement occurs. Increased attention up to 1 month after onset correlates with increased arm use after 6 months, suggesting how attention predicts future arm use. Bodyspecific attention is important for paretic limb use. In chronic stage, without rehabilitation, patients will exhibit decline of body-specific attention to the paretic hand, which correlates with duration since onset (Aizu et al., 2018). In addition, we showed that chronic phase intensive rehabilitation not only improve motor function but also increases body-specific attention. Retaining body-specific attention must be important to preserve and improve paretic limb function.

### Public Symposium 19

### [SY19]
### Physiological functions based on robust sustainability and dynamic adaptability of the molecular clock

March 18(Fri), 14:15 - 16:15, Room E

### [SY19-01]
### Consideration of the interface between thyroid function and circadian oscillation mechanism

*Masaaki Ikeda[1], Megumi Kumagai[1], Shinnosuke Yanagisawa[2,1], Yasuhiro Takenaka[3], Yoshihiro Nakajima[4] ([1]*Dept. of Physiology, Saitama Medical University,* [2]*Dept. of Diabetes and Endocrinology, Saitama Medical University,* [3]*Dept. of Physiology, Nippon Medical School,* [4]*AIST Health & Medical Research Institute*)

Thyroid hormones play an important role in metabolism, cell development, differentiation and growth, and hypofunction of these hormones can induce depression and impair the body's ability to maintain homeostasis. Thyroid function is controlled by the hypothalamic. pituitary.thyroid (HPT) axis; the endocrine system, which includes the HPT axis, is strongly regulated by circadian rhythms. Although the circadian rhythms of thyrotropin-releasing hormone (TRH), thyroid-stimulating hormone (TSH), $T_4$, and $T_3$ in the blood have been well studied, there are few reports on the effects of these hormones on the circadian control mechanisms of peripheral organs and cells. In this presentation, we will provide an overview of the HPT axis and circadian rhythms and present recently obtained data on the effects of thyroid hormones on the molecular clockwork of cells.

### [SY19-02]
### Elucidating mechanisms of the synchronous cellular clock oscillation to overcome time difference disorders

*Teruya Tamaru[1], Yasufumi Shigeyoshi[2], Genki Kawamura[3], Takeaki Ozawa[3], Hikari Yoshitane[4], Kimiko Shimizu[5], Yoshitaka Fukada[5], Ken Takamatsu[1] ([1]*Department of Physiology, Toho University, School of Medicine,* [2]*partment of Anatomy & Neurobiology, Kindai University, School of Medicine,* [3]*Department of Chemistry, The University of Tokyo,* [4]*Tokyo Metropolitan Institute of Medical Science,* [5]*Department of Biological Science, The University of Tokyo*)

Molecular/cellular clocks in the whole body are temporal basis for the harmonized physiological functions and adaptation to the environments, by their autonomous oscillation machinery with clock genes/proteins such as BMAL1 and synchronizing ability to the environments. On the other hand, current sudden environmental changes in the current world possibly cause various health problems and diseases by time difference disorders such as jet lag. As the evidence, we demonstrate that clock synchronization responses cooperating with numerous adaptation systems simultaneously drive adaptation systems, and impairments of these processes result in decreased adaptabilities. This research is planned to elucidate initial clock protein events driving synchronous cellular clock oscillation as the basis for environmental adaptation, and its physiological significances, In this topic, I will report about BMAL1-S region (BMAL1-S) essential for the clock synchronization, circadian phosphorylation and environmental change-responsive localization change (ISR) as the key events, presenting the data obtained using genetic engineering, live cell imaging and novel clock inhibitor which applied to peripheral and central clock (SCN). Our research products expectedly make unique developments various health problems and diseases (cancers, lifestyle-related diseases, mental diseases, etc.) by overcoming time difference disorders.

### [SY19-03]
### Multiple regional oscillators in the suprachiasmatic nucleus, the center of the mammalian circadian clock

*Yasufumi Shigeyoshi[1] ([1]*Department of Anatomy and Neurobiology, Faculty of medicine, Kindai University*)

In this symposium, I will focus on multiple regional oscillators in the suprachiasmatic nucleus (SCN). The SCN in the hypothalamus is the mammalian circadian clock center. Although almost all tissues other than the SCN have peripheral clocks with the sufficient molecular components of a circadian clock, the SCN is distinct from peripheral clocks in its ability to maintain long-term circadian rhythms when isolated from adjacent tissues. Further, different from peripheral oscillators, the SCN is consists of many subregional oscillators with different periods and functions. The appearance of such small areas of oscillators is often revealed by sudden changes of environmental light conditions. For example, a sudden shift in the light/dark cycle of the environment produces a state of jet lag. The circadian rhythm of the ventral lateral nucleus (VLSCN, or Core), which receives direct projection from the retina, shifts rapidly, whereas the circadian rhythm of the dorsomedial part of the SCN (DMSCN, or Shell), which does not receive direct projection from the retina, show phase shift for two hours per day at most and this slowness causes jet lag. Moreover, recent findings suggested that both VLSCN and DMSCN seems to have multi oscillators which have independent functions. With all, the SCN is a collection of such multiple regional oscillators, which seems to allow it to adapt to changes in the environment.

### [SY19-04]
### Regulation of circadian pacemaker neurons via mitochondrial cation antiporter Letm1

Masayuki Ikeda[1], *Eri Morioka[1] ([1]*Faculty of Science, University of Toyama*)

Various cellular activities are governed by circadian clocks for optimization of activities and anticipation of cyclic environmental fluctuations. Cellular metabolic activity is one of typical activities and circadian rhythms in ATP synthesis has been demonstrated in ubiquitous cells. The core mechanisms underlie transcriptional translational feedback loops (TTFLs) of clock genes. Mitochondrion is an organelle for ATP synthesis, buffering and transporting intracellular ions. How metabolic rhythms together with TTFLs interact with mitochondrial ion flux has not yet been clearly described. Here, we demonstrate critical functions of mitochondrial cation antiporter (LETM1). *Letm1* knockdown in *Drosophila* pacemaker neurons reduced circadian cytosolic $H^+$ rhythms and prolonged nuclear PER/TIM expression rhythms and locomotor activity rhythms. In rat pacemaker neurons in the hypothalamic suprachiasmatic nucleus (SCN), circadian rhythms in cytosolic $Ca^{2+}$ and *Bmal1* transcription were dampened by *Letm1* knockdown. Mitochondrial $Ca^{2+}$ uptake peaks late during the day were also observed in rat SCN neurons following photolytic elevation of cytosolic $Ca^{2+}$. We propose that LETM1 integrates metabolic, ionic and molecular clock rhythms in the central clock system.

### [SY19-05]
### Diurnal rhythm of remote spatial memory formation and its regulation by neurosteroids in mice

*Kimiko Shimizu[1], Kanako Maehata[1], Tomoko Ikeno[1], Qiuyi Wang[2], Zefeng Wei[1], Ayaka Sakurai[1], Tosifumi Takao[2], Yoshitaka Fukada[1] ([1]*Dept. Biological Sciences, School of Science, The University of Tokyo,* [2]*Laboratory of Protein Profiling and Functional Proteomics, Institute for Protein Research, Osaka University,*)

It has become clear that the circadian clock controls the rhythm of sleep, metabolism, and higher brain functions such as emotions and memory formation in mammals. Here we examined the diurnal changes in spatial memory and found a diurnal rhythm in long-term memory retention (remote memory) that is the most efficient at dawn, but no diurnal changes in learning ability or recent memory. In the process of working on this mechanism, we found that two 7a-hydroxylated neurosteroids, 7a-hydroxypregnenolone and 7ahydroxydehydroepiandrosterone, synthesized in the hippocampus were up-regulated following spatial learning at dawn. Neurosteroids are steroid hormones that are synthesized in the brain. *Cyp7b1*-deficiency, which cannot synthesize the two 7a-hydroxylated neurosteroids, impaired remote spatial memory with recent memory mostly unaffected. The hippocampal dendritic spine densities were reduced in *Cyp7b1*-deficient mice, and the training no more increased them. Furthermore, chronic intracerebroventricular administration of the two neurosteroids rescued the deteriorated remote memory performance in *Cyp7b1*-deficient mice. It is concluded that the 7a-hydroxylated neurosteroids are required for longterm maintenance of spatial memory, and we suggest that these neurosteroids may induce synaptic remodeling to maintain the hippocampal function.

### Public Symposium 20

### [SY20]
### Mechanism maintaining Pi homeostasis in our bodies - toward comprehensive understanding of its functions from molecule to whole body

March 18(Fri), 14:15 - 16:15, Room F

### [SY20-01]
### Toward understanding of transport mechanism of SLC34 $Na^+$/Pi cotransporter

*Yoshifumi Okochi[1], Junxian Zhou[1], Natsuki Mizutani1, Yuji Shiozaki[2], Hiroko Segawa[2], Yasushi Okamura[1] ([1] *Integrative Physiology, Graduate School of Medicine, Osaka university,* [2] *Department of Applied nutrition, Institute of Biomedical Sciences, Tokushima University Graduate School Tokushima*)

SLC34 family of sodium-coupled inorganic phosphate (Pi) transporters (NaPi-IIa, b, c) plays a central role for absorption and reabsorption of Pi in intestine and kidney, respectively. Mutations in SLC34 are known to cause diseases such as hypophosphatemic rickets with hypercalciuria (HHRH) and pulmonary alveolar microlithiasis. However, how Pi is transported through SLC34 remains unknown. PI(4,5)$P_2$ is an important regulator of ion channel activities at the plasma membrane. We therefore asked whether PI(4,5)$P_2$ regulates NaPi-II and addressed the question by performing electrophysiological experiments. Of the NaPi-II isoforms, IIa and IIb are electrogenic, but IIc is electroneutral. To study IIc, we used electrogenic mutant of mouse IIc. We found NaPi-IIb, but not IIa and IIc, is regulated by PI(4,5)$P_2$. Next, because many mutations in IIc have been identified in human patients, we introduced mutations into mouse electrogenic IIc to examine the effect on transport capacity. Surprisingly, we found a mutant (I196V) that exhibited enhanced transport activity, but with plasma membrane localization comparable to the electrogenic IIc. This result indicates that the I196V mutation only influences transport capacity of IIc. Overall, our results advance our understanding of the mechanism underlying Pi transport of SLC34.

### [SY20-02]
### Regulation of Phosphate Transporters in Health and Disease

*Hiroko Segawa[1], Sumire Sasaki[1], Megumi Koike[1], Yuji Shiozaki[1], Ken-ichi Miyamoto[2] ([1] *Department of Applied nutrition Institute of Biomedical Sciences, Tokushima University Graduate School ,* [2] *Graduate School of Agriculture, Ryukoku University*)

Phosphorus is required by all living organisms, including animals and plants, and plays a role in maintaining biological functions, such as energy metabolism, cell membranes, and bone mineralization. Various factors in the intestine, kidneys, and bones regulate the homeostasis of the inorganic phosphate (Pi) concentration in the body. SLC34A1/NaPi2a and SCL34A3/ NaPi2c sodium-dependent phosphate transporters responsible for Pi reabsorption in the kidney, are essential molecules for regulating the plasma Pi concentration. Both transporters are predominantly expressed at the apical side in the proximal tubules of the kidney. Parathyroid hormone (PTH) and fibroblast growth factor 23 (FGF23) are the main contributing hormones regulating the renal NaPi2a and NaPi2c transporters. With a high dietary Pi load, plasma PTH and FGF23 levels are increased to promote the internalization of NaPi2a and NaPi2c in the proximal tubular cells. As a result, urinary Pi excretion is enhanced, and the onset of hyperphosphatemia is prevented. However, the mechanisms underlying both the induction of phosphaturic hormones by dietary Pi and regulation of the serum Pi concentration remain unclear. In this symposium, we review recent findings of the regulation of Pi transporters in the body.

### [SY20-03]
### Physiology and pathophysiology of the FGF23-Klotho endocrine system

*Makoto Kuro-o[1] ([1] *Jichi Medical University*)

Endocrine fibroblast growth factors (FGFs) require Klotho proteins as an obligate co-receptor to bind to and activate FGF receptor tyrosine kinases. FGF23 is a bone-derived hormone secreted in response to phosphate intake and acts on renal tubules that express aKlotho to increase urinary phosphate excretion per nephron and maintain the phosphate balance. FGF21 is secreted from the liver upon fasting and acts on the suprachiasmatic nucleus in the brain where bKlotho is expressed to induce responses to stress, including activation of the hypothalamus-pituitary-adrenal axis and the sympathetic nervous system. FGF21 is regarded as an anti-aging hormone, because mice overexpressing FGF21 live longer than wildtype mice. Both FGF23 and FGF21 start increasing since early stages during the course of chronic kidney disease (CKD) progression. The increase in FGF23 compensates for decrease in the functional nephron number by increasing phosphate excretion per nephron, thereby maintaining phosphate homeostasis. However, FGF23-induced increase in phosphate concentration in the renal tubular fluid damages tubular cells and triggers interstitial fibrosis. In addition, FGF23 causes decrease in the serum level of active vitamin D followed by increase in parathyroid hormone, leading to mineral and bone disorders (CKDMBD). The increase in FGF21 is necessary to survive CKD, because CKD mice lacking FGF21 exhibit poorer prognosis than wild-type CKD mice. However, FGF21-induced activation of the sympathetic nervous system results in blood pressure dysregulation. Thus, pathophysiology of CKD can be viewed as adverse effects associated with adaptive responses of the FGF-Klotho endocrine system to maintain survival and phosphate homeostasis. Once CKD progresses to renal failure, hyperphosphatemia ensues due to impaired urinary phosphate excretion. Because the blood is super-saturated in terms of phosphate and calcium ions, an increase in blood phosphate concentration triggers precipitation of calciumphosphate. The calcium-phosphate precipitates are adsorbed by serum protein fetuin-A to form colloidal nanoparticles called calciprotein particles (CPPs). CPPs have the ability to induce cell damage, calcification, and inflammatory responses in cultured cells. Thus, we hypothesize that CPPs may behave like a pathogen that causes arteriosclerosis and non-infectious chronic inflammation, thereby accelerating aging eventually. We propose that CPPs may be an effective therapeutic target to improve clinical outcomes of patients with end-stage renal disease.

### [SY20-04]
### Maternal-fetal mineral transport for fetal bone development

*Yoshiro Suzuki[1] ([1] *Department of Physiology, Iwate Medical University*)

Homeostasis of minerals including calcium ($Ca^{2+}$) and inorganic phosphate ($P_i$) is maintained by the coordination between intestinal absorption, renal reabsorption, and bone remodeling. In fetus, large amount of these minerals is needed because fetal bone has to be mineralized during development. Therefore, it has been suggested that there are transcellular mineral transport mechanisms through the placenta. Transient receptor potential cation channel, subfamily V, member 6 (TRPV6) is an epithelial $Ca^{2+}$-selective channel which belongs to the TRP channel family. Trpv6 knockout fetal mice exhibited hypocalcemia with a half amount of bone minerals, and their maternal-fetal $^{45}Ca^{2+}$ transport through the placenta was shown to be actually smaller compared to WT or heterozygous mice. Interestingly, these mice were almost completely recovered after birth. Recently, TRPV6 gene mutations have been found from patients with transient neonatal hyperparathyroidism (TNHP). TNHP patients have extremely high blood PTH level with bone abnormalities, but they were recovered within several months after birth. These findings suggest that TRPV6 is involved in the maternal-fetal $Ca^{2+}$ transport which is critical for fetal bone mineralization.

### Public Symposium 21

### [SY21]
### New trends in computational neurobiology

March 18(Fri), 14:15 - 16:15, Room G

### [SY21-01]
### A computational approach for analyzing subthreshold voltage signaling along axon

*Haruyuki Kamiya[1] ([1]*Hokkaido University Graduate School of Medicine*)

Computer simulation is a powerful tool of neurobiology for quantitative interpretation of experimental findings. In physiological studies of axons, simulation has been widely adopted as a supplementary approach alongside fine electrophysiological experiments. Understanding axon physiology remains to be accomplished since extremely small structures hamper detailed examination in experiments. Due to accumulating data on the electrical properties as well as its microstructure, the hippocampal mossy fiber is one of the best-studied axons in the central nervous system, and therefore suited to construct a realistic model incorporating the experimentally defined properties of ionic channels on axonal membranes. Here we focused on the subthreshold axonal voltage signaling by dendritic synaptic inputs, namely excitatory presynaptic potentials (EPreSPs) propagation which enhanced transmitter release by subsequent action potentials. Preceding EPreSPs slightly reduced the action potential-evoked presynaptic calcium currents, while they elicited small calcium currents by themselves. Potential mechanisms underlying the EPreSP-induced enhancement of transmitter release will be discussed. (COI: No)

### [SY21-02]
### Computational modeling of age-dependent tonic inhibition in the cerebellar granule cells in a network context

*Sungho Hong[1], Jae Kwon[2], Junsung Woo[3], Sunpil Kim[1], Erik De Schutter[1], C. Justin Lee[2] ([1]*Okinawa Institute of Science and Technology*, [2]*Institute for Basic Science*, [3]*Baylor College of Medicine*)

Tonic inhibition is the slowest form of inhibitory signaling between neurons, mediated by extra-synaptic GABA receptors. It originates from different sources: the GABA spillover from synapses and the ambient GABA from glial cells (Farrant and Nusser, 2005), which are regulated by developmental processes (Wall and Usowicz, 1997). However, its functional implications at the network level are not understood well. We addressed this question by intracellular recording of tonic inhibition in granule cells in the cerebellar cortex and by physiologically detailed computational modeling of the network (Sudhakar et al., 2017). We found that, during maturation from adolescence, a significant decrease in the synaptically driven component of the tonic inhibitory current (TIC) shifts the network activity from a network-driven to a more input-driven regime, even when the total TIC remains the same. In a similar investigation on KO animals with impaired ambient GABA, the network activity changed on a much smaller scale. Therefore, our results demonstrated that the normal development of the ambient GABA is crucial in developing the computational function of the network.

### [SY21-03]
### Data-driven Modeling of Neuronal Nonlinear Dynamics

*Toshiaki Omori[1] ([1]*Kobe University Graduate School of Engineering*)

Revealing nonlinear dynamics in neural systems is one of the essential subjects in neuroscience. Developments in recording technologies enable us to access spatiotemporal responses from neural systems. To extract nonlinear dynamics underlying such neuronal responses, we have proposed combined approaches with computational models and datadriven methods for simultaneously estimating multi-dimensional latent variables and underlying biophysical parameters. We also discuss frameworks for extracting substantial nonlinear membrane currents from candidates, and for estimating neuronal dynamics under nonlinear observation in imaging recording by means of model-driven and datadriven approaches.

### [SY21-04]
### Dynamical richness defined by modular organization in engineered neuronal networks

*Hideaki Yamamoto[1], Ayumi Hirano-Iwata[1,2], Shigeo Sato[1] ([1]*RIEC, Tohoku Univ.*, [2]*WPI-AIMR, Tohoku Univ.*)

Neurons in dissociated culture play irreplaceable roles in molecular and cellular neuroscience. However, their use in the network-level has been challenged by the substantial difference in the network structure from the actual brain. The structural difference results in the generation of excessively synchronized bursting in cultured neuronal networks that are rarely observed in vivo. To overcome this issue, we developed a series of surface engineering technologies to extrinsically guide the development of cultured cortical neurons. We focus on the modular organization that are evolutionarily conserved in the nervous system of animals and investigated how network modularity at the mesoscopic scale influences the network dynamics in cultured neuronal networks. We show that induction of modular organization suppresses coherent bursting and promotes coexistence of coherent and incoherent states, thereby increasing the dynamical richness. The results demonstrate that microfabrication provides a unique tool in neuroscience to constructively study the structure-function relationships in living neuronal networks, bridging in vivo studies to computational modelling.

### [SY21-05]
### Simulation of spatiotemporal $Ca^{2+}$ dynamics in morphologically realistic dendrites

*Hidetoshi Urakubo[1] ([1]*National Institute for Physiological Sciences*)

Neuronal dendrites have characteristic structure called the spines, which hold biochemical signaling to enable simulation site-specific occurrence of synaptic plasticity. However, this spatial compartmentalization is imperfect, and synaptic plasticity thus partly spreads into neighboring unstimulated synapses, which results in heterosynaptic plasticity. We are conducting computer simulation on the working hypothesis that the imperfect spatial compartmentalization depends on morphological characteristics of spines. We target spines in hippocampal CA1 region, and are examining how spine shapes contribute to the biochemical compartmentalization. I would like to report the current status of this project. The shapes of spines were obtained from volumetric images of electron microscopy, which were based on the technologies for EM connectomics. We would also introduce such information technologies for neuroscience.

### Public Symposium 22

### [SY22]
### Microglial decoding of brain information

March 18(Fri), 14:15 - 16:15, Room H

### [SY22-01]
### Microglial process dynamics to orchestrate spine plasticity in concert with other brain cells

*Ako Ikegami[1], Daisuke Kato[1], Hiroaki Wake[1] ([1]*Department of Anatomy and Molecular Cell Biology, Nagoya University Graduate School of Medicine*)

Microglia monitor and maintain neuronal homeostasis by constant surveillance and contact with synapses. Dynamic microglia process promotes synapse formation synapse elimination and thus contribute on the regulation of synapse number. In addition, accumulated evidence reveals microglial processes respond to injuryand modulated by and several neuro- / glio-transmitters. Still, whether these dynamics contribute to structural synaptic plasticityremains largely unknown.We investigated microglial dynamics and spine formation / elimination in learning through in vivo two-photon imaging. Mice were trained for 14 days to pull a lever to get a water reward. Rapid spine formation occurred in the early phase, followed by elimination in the later phase of learning. Microglial process dynamicsexpressed distinct differences at each stage; in the early phase, random but prolonged interaction with dendrites induced spine protrusion, and in the later phase, processes made repetitive contact with specific spines that were later eliminated. $Ca^{2+}$ imaging and cell-specific inhibition of other brain cells suggested that astrocytic activity induced prolonged microglial contact in the early phase, while pre-synaptic activity repetitively drew microglial processes toward specific synapses in the later phase.We discovered microglial process dynamics involveorchestration with other brain cells to induce spine formation and elimination. This provides a new perspective on microglial contributions to pathologies such as schizophrenia and Alzheimer's disease.

### [SY22-02]
### A specific morphological phenotype of hippocampal microglia associated with resiliency to social defeat stress, with reference to synaptic boutons

*Risako Fujikawa[1], Jinno Shozo[1] ([1]*Dept Anat and Neurosci, Grad Sch Med, Kyushu Univ*)

Social stresses are associated with the risk of mental health disorders. In this study, we aim to elucidate the potential role of microglia, resident immune cells of the brain, in determining the vulnerability and resiliency to social defeat stress (SDS). Male C57BL/6J mice were repeatedly subjected to SDS (1 min/day) by an aggressive male ICR mouse for five consecutive days. After SDS, about half of stressed animals showed social avoidance behaviors (vulnerable mice), while remaining animals showed no such behaviors (resilient mice). The spatial densities of microglia in the CA1 region of the hippocampus were higher in than in resilient mice. The total process lengths of microglia were shorter in vulnerable mice than in controls, while they were comparable between resilient mice and controls. According to the hierarchical cluster analysis of morphological characteristics, microglia were objectively categorized into three groups. Interestingly, the type of microglia mainly found in resilient mice displayed increased complexity, like hyper-ramified microglia. Synaptic boutons colocalized with microglia were increased in resilient mice compared to vulnerable mice. These results suggest that a specific morphological phenotype of hippocampal microglia may be associated with resiliency to SDS through interaction with synaptic boutons.

### [SY22-03]
### Decoding brain environment through single-cell analysis of microglia

*Takahiro Masuda[1] ([1]*Kyushu Univ.*)

Microglia play crucial roles not only in neural development and homeostasis, but also in diseases of the central nervous system. The highly diverse and specialized functions of microglia in different situations can be achieved through their phenotypic shift with a robust change in gene expression in response to the surrounding environment by virtue of their plasticity. Recent emergence of single-cell technologies allowed us assessing gene expression profile at the single-cell level and enhanced our understanding of the functional heterogeneity and plasticity of microglia during health and perturbation. In this symposium, I will discuss the current knowledge about functional diversity and plasticity of microglia during development, homeostasis and disease.

### [SY22-04]
### Local apoptosis promotes complement tagging of highly-active synapses for microglial phagocytosis

*Megumi Andoh[1], Ryuta Koyama[1,2] ([1]*Laboratory of Chemical Pharmacology, Graduate School of Pharmaceutical Sciences, The University of Tokyo,* [2]*Institute for AI and Beyond, The University of Tokyo*)

Microglia refine neural circuits through complement-dependent synaptic phagocytosis. Additionally, local apoptosis at synapses could induce complement tagging. However, whether microglia phagocytose apoptotic synapses has not been determined by live imaging, and the underlying mechanism remains unveiled. Here, we found that elevated neuronal activity induced synaptic caspase expression, which promotes complementdependent synaptic phagocytosis by microglia. Live imaging of a newly-developed neuronglia co-culture system revealed that microglia pinch off complement-tagged synapses with their ramified processes without snipping off axons. The induction of complement tagging at a synapse required coincident timing of upregulation of cleaved caspase-3 at that synapse, which was regulated by elevated neuronal activity. Finally, experimental febrile seizures induced activity-dependent local apoptosis at inhibitory synapses and temporallylinked complement cascade activation in microglia, resulting in phagocytosis of inhibitory synapses and increased seizure sensitivity. We propose that caspase and complement orchestrate phagocytosis of highly-active synapses by microglia and modulate brain function.
Matters requiring disclosures of COI with regard to the presentation are as follows; Takeda Pharma Co., Ltd., Sumitomo Dainippon Pharma Co., Ltd., Astellas Pharma Inc.

### Award Presentation

| | |
|---|---|
| AP-1 ～ AP-2 | 23rd Promotion Award of the Physiological Society of Japan for Young Scientists |
| AP-3 ～ AP-4 | 12th Hiroshi and Aya Irisawa Memorial Promotion Award for Young Physiologists |
| AP-5 ～ AP-6 | 12th Aya Irisawa Memorial Promotion Award for Excellence by Women Physiologists |
| AP-7 ～ AP-8 | 12th Hiroshi and Aya Irisawa Memorial Award for Excellent Papers in The Journal of Physiological Sciences |

### Award Presentation

### 23rd Promotion Award of the Physiological Society of Japan for Young Scientists

### [AP-1] (OP14-02)

**Cardiac ion channel remodelling associated with miRNA upregulation underlies exercise-induced bradyarrhythmias**

*Shu Nakao[1,2], Teruhisa Kawamura[1], Halina Dobrzynski[2], Mark Boyett[2], Alicia D'Souza[2] ([1]*Ritsumeikan Univ.*, [2]*Univ. of Manchester*)

Veteran endurance athletes are prone to bradyarrhythmias including sinus bradycardia and atrioventricular (AV) block, and have a high incidence of pacemaker implantation. Our recent work examined the underlying mechanism of resting bradycardia in human athletes and trained mice. The study demonstrates that the resting bradycardia by endurance exercise is due to ion channel remodelling associated with alterations in microRNAs (miRs) and transcription factors in the sinus node (SN), the primary pacemaking site in the heart. Transcriptome analysis in trained SN suggest metabolic remodelling as a causal factor. Our follow-up study using racehorses and trained mice as animal models of human athletes further explained that exercise-induced AV block also resulted from ion channel remodelling in the AV node. Moreover, miR profiling found 31 miRs significantly upregulated by exercise in AV node. We identified that miR-211-3p and miR-432 targeted $Ca_V1.2$ and HCN4 channels, key pacemaking ion channels. In addition, antimiRs against these miRs reversed exercise-induced SN and AV node dysfunction. Altogether, our data provide new insight into underlying mechanisms of exercise-induced bradyarrhythmias and a potential anti-miRNA therapy for cardiac arrhythmias. (COI:No)

### [AP-2] (OP21-01)

**Multiple cortical processing streams support response inhibition in humans**

*Takahiro Osada[1], Akitoshi Ogawa[1], Akimitsu Suda[1], Koji Nakajima[1,2], Masaki Tanaka[1], Satoshi Oka[1], Koji Kamagata[1], Shigeki Aoki[1], Yasushi Oshima[2], Sakae Tanaka[2], Nobutaka Hattori[1], Seiki Konishi[1] ([1]*Juntendo University*, [2]*The University of Tokyo*)

Response inhibition supports adaptive behavior by suppressing inappropriate behavior. Multiple areas are involved in response inhibition. However, it is poorly understood how the processing streams among the areas are implemented to achieve response inhibition. In this study, we first identified essential areas for response inhibition during a stop-signal task via fMRI. Next, single-pulse transcranial magnetic stimulation (spTMS) was administered during the task. Each of the areas was stimulated in half of the trials, and trials with and without stimulation were intermixed within runs. Prolonged stop-signal reaction time (SSRT) was observed in spTMS to the ventral posterior inferior frontal cortex (vpIFC) at the time window of -90 to -60 ms, with the end of the SSRT for no-stimulation trials defied as zero. The dorsal posterior inferior frontal (dpIFC), presupplementary motor area (preSMA), and intraparietal sulcus area (IPS) showed a transient disruption effect at the subsequent time windows: -60 to -30 ms for dPIFC and preSMA, and -30 to 0 ms for IPS. Finally, we tested whether the information flowed effectively from the vpIFC to the preSMA and from the vpIFC to the dpIFC. SpTMS following suppression of the vpIFC with repetitive TMS showed that prolonged SSRT was still observed by spTMS to the dpIFC, but not to the preSMA. These results suggest two parallel processing streams that act concurrently during response inhibition (vpIFC-preSMA vs. dpIFC-IPS).

### Award Presentation

### 12th Hiroshi and Aya Irisawa Memorial Promotion Award for Young Physiologists

### [AP-3] (OP20-03)

**The mechanism of mitochondrial dynamics regulation via PPI**

*Sho Aki[1], Kazuaki Yoshioka[2], Yoh Takuwa[2], Tsuyoshi Osawa[1] ([1]*Division of Integrative Nutriomics and Oncology RCAST, The University of Tokyo*, [2]*Department of Physiology Kanazawa University School of Medicine*)

The balance between fusion and fission of mitochondria that maintain the morphology and function of mitochondria, but the mechanism for maintaining mitochondrial homeostasis (mitochondrial dynamics) unclear. We found that polyphosphoinositide (PPI) generated by Mitochondrial Fusion Activating Kinases 1 and 2 (MFAK1 / 2) promote mitochondrial fusion. MFAK1 / 2 double knockdown increased excessive mitochondrial division and fragmentation, significantly reduced the function of fragmented mitochondria (membrane potential), and accumulation of reactive oxygen species (ROS) was observed. The expression levels of mitochondrial fusion factor (Mitofusin1 / 2, OPA1) and fission factor (Drp1, Dynamin2, MFF) did not change in MFAK1 / 2 double knockdown cells. In addition, although Mfn was recurited to the mitochondrial fusion site, mitochondrial fusion was inhibited, suggesting that MFAK1 / 2 metabolite PPI is essential for mitochondrial fusion. In addition, cardiomyocyte-specific Mfak1/2 double knockout (KO) mice showed accumulation of fragmented mitochondria in cardiomyocyte and died within 1-2 days after birth due to Abnormal cardiac contraction. These observations indicate that MFAK1 / 2 is a novel kinase that promotes mitochondrial fusion through PPI production and controls mitochondrial dynamics.

### [AP-4] (2P04-03)

**CMOS-based bio-image sensor reveals spatiotemporal proton dynamics in the living brain**

*Hiroshi Horiuchi[1], Masakazu Agetsuma[1], Junko Ishida[1], Dennis Cheung[1], Sawada Kazuaki[2], Junichi Nabekura[1] ([1]*National Insititute for Physiological Sciences*, [2]*Toyohashi University of Technology*)

The regulation of proton concentration (pH) in the brain is important for maintaining normal brain function. In the brains of healthy subjects, intracellular pH is maintained at 6.8.7.0, whereas extracellular pH is maintained at 7.2.7.4. While the homeostatic importance of pH regulation has long been appreciated, more recent studies have shown that protons can also directly participate in neurotransmission. This suggests an added dimension in terms of the relevance of pH changes to brain function under both physiological and pathological conditions. Double barreled and concentric microelectrodes can only measure pH at a single point, thus their utility is limited to correlating proton changes with globalized brain activity during seizures and ischemia. In contrast, magnetic resonance imaging (MRI) is able to simultaneously measure the distribution of protons in the entire brain and is thus able to detect regional variations in pH. However, to further investigate regional and neural activity-dependent proton dynamics in the brain, the development of a device with both wide-area detectability and high temporal-spatial resolution is necessary. Therefore, we developed a novel image sensor with a high spatial-temporal resolution specifically designed for measuring protons in vivo. Here, we demonstrate that spatially deferent neural stimulation by visual stimulation induced distinct patterns of proton changes in the visual cortex. This result indicates that our biosensor can detect micrometer and millisecond scale changes of protons across a wide area. To our knowledge, this is the first report showing that a CMOS-based proton image sensor with high spatial and temporal precision can be used to detect pH changes associated with biological events. Thus, we believe that our sensor may have broad applicability in future biological studies.

### Award Presentation

### 12th Aya Irisawa Memorial Promotion Award for Excellence by Women Physiologists

### [AP-5] (PS07-01)

**Neural mechanisms of the link between social stress and aggression**

*Aki Takahashi[1] ([1]*Faculty of Human Sciences, University of Tsukuba*)

Social stress can lead to the development of psychological problems ranging from exaggerated anxiety and depression to antisocial and violence-related behaviors. Violence incidents are often triggered by social instigation or frustration. In animal models, a brief encounter with a potential rival induces aggressive arousal which increases subsequent aggressive behavior, phenomena referred to as social instigation (or provocation). Previously, we have shown that the level of excitatory glutamate input into the dorsal raphe nucleus (DRN) was increased during social instigation-heightened aggression in the male mouse. The DRN receives glutamatergic projections from the lateral habenula (LHb), the brain area that is implicated in negative emotion and stress. By using optogenetis and DREADD techniques, we found that the LHb neurons that project to the DRN (LHb-DRN projection) are involved in the escalation of aggressive behavior induced by social instigation. In this talk, the role of LHb-DRN projection in terms of the link between social stress and aggression will be discussed.

### [AP-6] (OP22-03)

**Early phase of pupil dilation is mediated by the peripheral parasympathetic pathway.**

*Chinatsu Marumo[1], Tamami Nakano[2] ([1]*Faculty of Medicine, Osaka Univ.*, [2]*Graduate School of Frontier Bioscience, Osaka Univ.*)

Pupil diameter fluctuates in association with changes in brain states induced by the neuromodulator systems. However, it remains unclear how the neuromodulator systems control the activity of the iris sphincter (constrictor) and dilator muscles to change the pupil size. The present study compared temporal patterns of pupil dilation during movement when each muscle was pharmacologically manipulated in the human eye. When the iris sphincter muscle was blocked by tropicamide, the latency of pupil dilation was delayed and the magnitude of pupil dilation was reduced during movement. In contrast, when the iris dilator muscle was continuously stimulated by phenylephrine, the latency and magnitude of rapid pupil dilation did not differ from the untreated control eye, but sustained pupil dilation was reduced until the end of movement. These results suggest that the iris sphincter muscle, which is under the control of the parasympathetic pathway, is quickly modulated by the neuromodulator system and plays a major role in rapid pupil dilation. However, the iris dilator muscle receives signals from the neuromodulator system with a slow latency and is involved in maintaining sustained pupil dilation.

### Award Presentation

### [JPS]
### 12th Hiroshi and Aya Irisawa Memorial Award for Excellent Papers in The Journal of Physiological Sciences

March 17(Thu), 13:00 - 13:30, Room A

### [AP-7] (JPS-01)

**Chemogenetic activation of endogenous arginine vasopressin exerts anorexigenic effects via central nesfatin-1/NucB2 pathway**

*Kenya Sanada[1,2], Mitsuhiro Yoshimura[1,5], Naofumi Ikeda[3], Kazuhiko Baba[3], Haruki Nishimura[3], Kazuaki Nishimura[1], Yuki Nonaka[1], Takashi Maruyama[1], Tetsu Miyamoto[2], Masatomo Mori[4], Becky Conway-Campbell[5], Staford Lightman[5], Masaharu Kataoka[2], Yoichi Ueta[1] ([1]*Department of Physiology, School of Medicine, University of Occupationaland Environmental Health*, [2]*Second Department of Internal Medicine, School of Medicine, University of Occupational and Environmental Health*, [3]*Department of Orthopaedic Surgery, School of Medicine, University of Occupational and Environmental Health*, [4]*Research Institute for Metabolism and Obesity*, [5]*Translational Health Sciences, Bristol Medical School, University of Bristol*)

We examined whether the chemogenetic activation of endogenous arginine vasopressin (AVP) afects central nes fatin-1/NucB2 neurons, using a transgenic rat line that was previously generated. Saline (1 mL/kg) or clozapine-N-ox ide (CNO, 1 mg/mL/kg), an agonist for hM3Dq, was subcutaneously administered in adult male AVP-hM3Dq-mCherry transgenic rats (300.370 g). Food and water intake were signifcantly suppressed after subcutaneous (s.c.) injection of CNO, with aberrant circadian rhythmicity. The percentages of Fos expression in nesfatin-1/NucB2-immunoreactive neurons were signifcantly increased in the hypothalamus and brainstem at 120 min after s.c. injection of CNO. Sup pressed food intake that was induced by chemogenetic activation of endogenous AVP was ablated after intracere broventricularly administered nesfatin-1/NucB2-neutralizing antibody in comparison with vehicle, without any altera tion of water intake nor circadian rhythmicity. These results suggest that chemogenetic activation of endogenous AVP afects, at least in part, central nesfatin-1/NucB2 neurons and may exert anorexigenic efects in the transgenic rats.

### [AP-8] (JPS-02)

**TMC4 is a novel chloride channel involved in high-concentration salt taste sensation**

*Yoichi Kasahara[1], Masataka Narukawa[1,2], Yoshiro Ishimaru[3], Shinji Kanda[4], Chie Umatani[4], Yasunori Takayama[5], Makoto Tominaga[5,6], Yoshitaka Oka[4], Kaori Kondo[7], Takashi Kondo[7], Ayako Takeuchi[8], Takumi Misaka[1], Keiko Abe[1,9], Tomiko Asakura[1] ([1]*Department of Applied Biological Chemistry, Graduate School of Agricultural and Life Sciences, The University of Tokyo*, [2]*Department of Food and Nutrition, Kyoto Women's University*, [3]*Department of Agricultural Chemistry, Meiji University*, [4]*Department of Biological Sciences, Graduate School of Science, The University of Tokyo*, [5]*Division of Cell Signaling, National Institute for Physiological Sciences, National Institutes of Natural Sciences*, [6]*Thermal Biology Research Group, Exploratory Research Center On Life and Living Systems (ExCELLS), National Institutes of Natural Sciences*, [7]*Laboratory for Developmental Genetics, RIKEN-IMS*, [8]*Department of Integrative and Systems Physiology, Faculty of Medical Sciences, and Life Science Innovation Center, University of Fukui*, [9]*Kanagawa Institute of Industrial Science and Technology (KISTEC)*)

"Salty taste" sensation is evoked when sodium and chloride ions are present together in the oral cavity. The presence of an epithelial cation channel that receives $Na^+$ has previously been reported. However, no molecular entity involving $Cl^-$ receptors has been elucidated. We report the strong expression of transmembrane channel-like 4 (TMC4) in the circumvallate and foliate papillae projected to the glossopharyngeal nerve, mediating a high-concentration of NaCl. Electrophysiological analysis using HEK293T cells revealed that TMC4 was a voltage-dependent $Cl^-$ channel and the consequent currents were completely inhibited by NPPB, an anion channel blocker. TMC4 allowed permeation of organic anions including gluconate, but their current amplitudes at positive potentials were less than that of $Cl^-$. *Tmc4*-defcient mice showed signifcantly weaker glossopharyngeal nerve response to high-concentration of NaCl than the wild-type littermates. These results indicated that TMC4 is a novel chloride channel that responds to highconcentration of NaCl.

### Oral Presentation

### Oral Presentation 1

### [OP01]
### Behabior, Biological rhythm, Sleep

March 16(Wed), 11:15 - 12:15, Room D

### [OP01-01]
### Cold-tolerant circadian $Ca^{2+}$ rhythms in the master clock neurons

*Ryosuke Enoki[1], Naohiro Kon[2,3], Yoshifumi Yamaguchi[4], Tomomi Nemoto[1] ([1]*NIPS/ExCELLS,* [2]*ITbM, Nagoya University,* [3]*Nagoya university graduate school of bioagricultural sciences,* [4]*ILTS, Hokkaido University*)

During the harsh season in winter, many species undergo hypothermia by reducing the thermogenesis and metabolic rate. Deep hibernators (ex., Syrian hamsters), control the core body temperature approaching ambient temperature which lasts for many months. Some animals (ex., mice) enter a daily torpor that reduces the energy expenditure to survive when food is unavailable at any time of year. During the torpor, the core body temperature drops down to room temperature and naturally back to a normothermic state.
In mammals, the circadian rhythms in physiology and behavior are controlled by the master clock located in the suprachiasmatic nucleus (SCN) in the hypothalamus. The SCN has a temperaturecompensated circadian clock, which is believed to rely on biochemical oscillations composed of clock genes and their protein products. It has long been debated whether the master circadian clock remains functional under hypothermic conditions. However, the SCN rhythmicity in a cold environment has not been examined directly.
In the present study, we performed dual-color fluorescence imaging of clock protein BMAL1 and $Ca^{2+}$ in the SCN slices prepared from mice and the Syrian hamster in a cold environment. We systematically changed the environmental temperature and found that cold exposure (20-28°) significantly alters the BMAL1 rhythms compared to $Ca^{2+}$ rhythms in terms of phase, period, amplitude, and expression level, but the rhythms persisted. Importantly, the BAML1 and $Ca^{2+}$ rhythms stop ticking time at 15 °C, and rewarming resets the phase of the rhythms. From these observations, we propose a model in which the master clock neurons have a "coldtolerant $Ca^{2+}$oscillator" coupled with clock proteins.

### [OP01-02]
### Cellular senescence triggers altered circadian clocks with a prolonged period and delayed phases

*Yasukazu Nakahata[1,2], Rezwana Ahmed[1,2,3], Yasumasa Bessho[2], Kazuyuki Shinohara[1] ([1]*Nagasaki Univ.,* [2]*NAIST,* [3]*North South Univ.*)

Over the last decade, a wide array of evidence has been accumulated that disruption of circadian clock is prone to cause age-related diseases and premature aging. On the other hand, aging has been identified as one of the risk factors linked to the alteration of circadian clock. These evidences suggest that the processes of aging and circadian clock feed back on each other at the animal level. However, at the cellular level, we have revealed that the primary fibroblast cells derived from *Bmal1*$^{-/-}$mouse embryo, in which circadian clock is completely disrupted, do not demonstrate the acceleration of cellular aging, i.e., cellular senescence. Furthermore, little is known about the impact of cellular senescence on the circadian clock. Here, we show that senescent cells, irrespective of the replicative senescence or the stress-induced premature senescence, possess a longer circadian period with delayed peak-time compared to their proliferative counterparts. Furthermore, we show that the alteration of the circadian clock properties in senescent cells might be reversed by the boost of $NAD^+$ through activations of $NAD^+$-dependent enzymes.

### [OP01-03]
### Comprehensive analysis identified the circadian clock and global circadian gene expression in human corneal endothelial cells.

*Yoshiki Tsuchiya[1], Hiroko Nakai[1], Nobuya Koike[1], Taiki Asano[1], Morio Ueno[1], Yasuhiro Umemura[1], Yuh Sasawaki[1], Ryutaro Ono[1], Junji Hamuro[1], Chie Sotozono[1], Kazuhiro Yagita[1] ([1]*Kyoto Prefectural University of Medicine*)

In ocular tissues, daily variation in physiology and morphology such as changes in corneal thickness have been observed. However, the existence and the possible role of the local circadian clock in corneal endothelial cells have not been revealed yet. Here we investigated circadian clock oscillation and circadian global gene expression in cultured human corneal endothelial cells (cHCECs). As a result, cHCECs exhibited clear circadian oscillation of *Bmal1:luciferase* reporter bioluminescence. The core clock genes and clockrelated genes showed high-amplitude robust circadian mRNA expression rhythms. Moreover, RNA-seq analysis identified 329 genes that exhibited circadian mRNA expression rhythms. These genes are involved in various physiological processes including glycolysis, mitochondrial function, anti-oxidative systems, hypoxic responses, apoptosis, and extracellular matrix regulation.
Our study identified a robust and functional circadian clock in cHCECs. The fact that a large number of genes exhibit circadian mRNA expression rhythms in cHCECs suggests a potential contribution of circadian regulation to fine-tuning of HCEC functions for daily changes in the environment.

### Oral Presentation 2

### [OP02]
### Circulation

March 16(Wed), 11:15 - 12:15, Room E

### [OP02-01]
### Improved method for isolating mouse heart cells by Langendorff-free antegrade perfusion technique - Get more cells in a shorter time

Mariko Omatsu-Kanbe[1], *Ryo Fukunaga[1], Xinya Mi[1], Hiroshi Matsuura[1] ([1]*Shiga University of Medical Science*)

Langendorff-based retrograde perfusion of the heart is a gold standard for isolating heart cells from rabbits, guinea-pigs or rats. However, a high degree of skill is required when this method is used with a small mouse heart. To solve this problem, we developed a Langendorff-free antegrade perfusion technique for the isolation of mouse cardiomyocytes. We herein report the improved method for preparing more live cells in a short time. There exist four important points before starting antegrade perfusion, namely the way of excising the heart intactly, the location of clamping the aorta, the direction of placing the clamped heart and the position of the needle insertion in the left ventricle. We show the knacks in each step to lead successful cell preparation. The perfusate can be changed depending on the purpose of the experiments. This improved method could open the door for the researchers in other field to start cardiac research using mice.

### [OP02-02]
### Estimation of ionic channel conductance by applying parameter optimization to various action potential configurations of human iPS cell-derived cardiomyocytes

*Yukiko Himeno[1], Hirohiko Kohjitani[2], Futoshi Toyoda[3], Yixin Zhang[1], Shigeya Koda[1], Akinori Noma[1], Akira Amano[1] ([1]*Ritsumeikan Univ.*, [2]*Kyoto Univ.*, [3]*Shiga Med. Univ.*)

Human-induced pluripotent stem cell-derived cardiomyocytes (hiPSC-CMs) are known to exhibit variable automaticity. If the mechanisms of the variation of action potential (AP) configurations in hiPSC-CMs are revealed, our understanding of the ionic mechanisms of normal and pathophysiological cardiac automaticity will be deepened. The ionic channels expressed in hiPSC-CMs are coded by the same set of genes as in human cardiomyocytes. Therefore, we hypothesized that the variation of the AP configurations was derived from various expression levels of functional ionic channels, which defines the maximum conductance of each ionic channel ($G_X$), whereas the gating kinetics of channels remained the same as in the matured cardiomyocytes. Firstly, we manually adjusted the $G_X$ of a mathematical human ventricular cell model (HuVEC model, Himeno et al., 2015) to fit roughly to AP data recorded from hiPSC-CMs using a priori knowledge in cardiac electrophysiology. Secondly, the $G_X$ of several major currents adjusted manually was iteratively randomized within the range of ± 5 ∼ 20% and then optimized computationally. By conducting the optimization procedure in two steps, it was possible to obtain a reliable final set of $G_X$ values independent of initial values of $G_X$ and reproduce APs recorded from hiPSC-CMs with high accuracy.

### [OP02-03]
### Autonomous oscillation characteristics of the heart measured by the new electron microscope live imaging method

*Seine A. Shintani[1] ([1]*Department of Biomedical Sciences, College of Life and Health Sciences, Chubu University*)

The presenter found that warmed cardiomyocytes produce Hyperthermal Sarcomeric Oscillations (HSOs) with a cycle close to the heartbeat. Then, by mathematical model analysis, we obtained the estimation result that HSOs are necessary properties for rapid expansion of each heartbeat. We would like to verify this estimation, but it is difficult to measure structures and three-dimensional movements below 200 nm by light microscopy. On the other hand, the scanning electron microscopy has a deep depth of focus and a fine minimum resolution of 0.5 nm. However, there is a drawback that it can only be observed as a dead body that has been fixed and dehydrated. Therefore, the presenter has developed an electron microscope live imaging method that enables measurement of the structure and movement of a sample immersed in a solution using a thin film with high electron beam permeability and deformability. By this method, the reciprocating motion of the saw waveform accompanied by micro-vibration of the mouse-excised heart was measured. This movement is likely to be derived from HSOs.

### [OP02-04]
### Molecular mechanism configuring $Ca_V1.2$ $Ca^{2+}$ channel current in cardiac ventricular action potential.

*Takuro Numaga-Tomita[1], Toshihide Kashihara[3], Hiroyuki Kawagishi[1], Tsutomu Nakada[2], Mitsuhiko Yamada[1] ([1]*Shinshu university school of medicine, Department of molecular pharmacology*, [2]*Shinshu University Research center for advanced science and technology*, [3]*Kitasato University School of pharmacy*)

Cardiac action potential (AP) induces an inward $Ca^{2+}$ current ($I_{AP}$) of $Ca_V1.2$ L-type $Ca^{2+}$ channels to evoke contraction. $I_{AP}$ configuration is critical for the robust contraction and stable rhythm of the heart. The initial prompt depolarization of AP causes an inward 'spike' of $I_{AP}$, leading to the $Ca^{2+}$-induced $Ca^{2+}$ release from the sarcoplasmic reticulum (SR). The following long AP plateau generates a 'dome' shape of $I_{AP}$, which provides $Ca^{2+}$ to reload SR for the next contraction and also determines the length of the refractory period. In this study, we analyzed the molecular mechanism of inactivation determining this 'spike-and-dome' configuration of $I_{AP}$ by applying a representative guinea-pig ventricular AP to recombinant LTCCs lacking different inactivation mechanisms. Specifically, the inactivation caused by the cytoplasmic linker between domains I and II ($L_{I-II}$) and the distal cytoplasmic C terminus (DCT) of $Ca_V1.2$, and calmodulin (CaM) preassociated $Ca_V1.2$ was inhibited by the type 1 Timothy syndrome (TS) mutation, deletion of DCT, and dominant negative CaM ($CaM_{1234}$), respectively. Through comparing their $I_{AP}$, we found that $L_{I-II}$ and DCT caused the inactivation independently of each other in the presence of $CaM_{1234}$ but cooperatively in its presence. Thus, so called the $Ca^{2+}$- and voltage-dependent inactivation relies on the overlapping molecular mechanisms and may not be independent of each other at least in AP. This cooperative interaction played a significant role in configuring the descending limb of the 'spike' and the amplitude of the 'dome' of $I_{AP}$. The TS mutation particularly severely impaired inactivation partly because of the increased open probability of LTCC caused by CaM-dependent kinase II and partly because its own effect on an inactivation gate. These results indicate that the inactivation arisen from the $Ca^{2+}$/CaMdependent cooperative interaction of $L_{I-II}$ and DCT is crucial for physiological configuration of $I_{AP}$ and thus, physiological cardiac function.

### [OP02-05]
### Mechanistic insight into $Ca^{2+}$ oscillation and ER $Ca^{2+}$ level in RyR2 expressing cells

*Nagomi Kurebayashi[1], Takashi Murayama[1], Ryosaku Ohta[2], Junji Suzuki[3], Kazunori Kanemaru[3], Masamitsu Iino[3], Fumiyoshi Yamashita[2] ([1]*Juntendo Univ*, [2]*Kyoto Univ*, [3]*Nihon Univ*)

Ryanodine receptor 2 (RyR2) is a cardiac $Ca^{2+}$ release channel in the ER. Mutations in RyR2 are linked to catecholaminergic polymorphic ventricular tachycardia (CPVT), which is associated with enhanced spontaneous $Ca^{2+}$ release. This spontaneous $Ca^{2+}$ release tends to occur when ER $Ca^{2+}$ ($[Ca^{2+}]_{ER}$) reaches a certain threshold level. CPVT mutations are reported to lower this threshold. There are two explanations for the lowered threshold: mutations enhance $[Ca^{2+}]_{ER}$- or cytosolic $[Ca^{2+}]$ ($[Ca^{2+}]_{cyt}$)-dependent RyR2 activity. We explored the mechanism relating the change in $[Ca^{2+}]_{cyt}$-dependent activity of RyR2 and the threshold $[Ca^{2+}]_{ER}$ by cell-based experiments and model-based simulations. WT and CPVT-mutant RyR2s were expressed in HEK293 cells, and $[Ca^{2+}]_{cyt}$ and $[Ca^{2+}]_{ER}$ were measured using fluorescent $Ca^{2+}$ indicators. CPVT cells showed higher oscillation frequency and lower threshold $[Ca^{2+}]_{ER}$ compared with WT cells. The $[Ca^{2+}]_{cyt}$-dependent activity at resting $[Ca^{2+}]_{cyt}$ determined by $[^3H]$ryanodine binding was inversely correlated with threshold $[Ca^{2+}]_{ER}$. A model-based simulation successfully reproduced $Ca^{2+}$ oscillations and $[Ca^{2+}]$ER changes in WT and CPVT cells. Our results suggest that the changes is threshold $[Ca^{2+}]_{ER}$ for $Ca^{2+}$ release is explained by the changes in $[Ca^{2+}]_{cyt}$-dependent activity without considering an alteration in the $[Ca^{2+}]_{ER}$ sensitivity of RyR2.

### Oral Presentation 3

### [OP03]
### Nutritional and metabolic physiology, Thermoregulation

March 16(Wed), 11:15 - 12:15, Room F

### [OP03-01]
### Role of circulating endocannabinoids in patients with cancer cachexia

*Kazuki Ota[1,2], Taeko Ota[3], Shin-Ichiro Nitta[4], Tetsuya Ueda[4], Tetsuji Yamashita[4], Taketoshi Ozawa[2] ([1]*Dept Physiol, Grad Sch Humanity and Life Sci, Tokyo Kasei Univ,* [2]*Megumi Home Clinic,* [3]*Med Health Eval Promot Center, Fujisawa-Junten Clinic,* [4]*LSI Medience Corporation*)

**Background:** Endocannabinoids (eCBs) are involved in various physiological functions such as stress response, metabolism, and inflammation. Although deterioration of these functions is often observed in patients with cancer cachexia (CC), the relationship between circulating eCBs and CC remains unknown.
**Methods:** Blood levels of N-arachidonoylethanolamine (AEA) and 2-arachidonoyl glycerol (2-AG) were measured in participants with CC and controls via LC-MS/MS method. Relationships between eCBs and clinical findings were also examined.
**Results:** Serum levels of AEA and 2-AG were significantly higher in the CC group. Multiple linear regression analyses showed that dietary intake was negatively, and serum cortisol and C-reactive protein (CRP) levels were positively, associated with AEA levels, while serum triglyceride and CRP levels were positively associated with 2-AG levels.
**Conclusion:** Circulating levels of eCBs were significantly increased in patients with CC. There is a possibility that both AEA and 2-AG participate in inflammation, and that AEA participates in stress conditions including anorexia, whereas 2-AG participates in lipid metabolism, in patients with CC. (COI:No)

### [OP03-02]
### Different roles of histone demethylase JMJD1A for adaptive thermogenesis in brown and white adipose tissue

*Ryo Ito[1], Shiyu Xie[1], Mygmal Tumenjargal[1], Chaoran Yang[1], Takeshi Yoneshiro[2], Yoshihiro Matsumura[2], Juro Sakai[1,2] ([1]*Molecular Physiology and Metabolism, Tohoku University Graduate School of Medicine,* [2]*Division of Metabolic Medicine, Research Center for Advanced Science and Technology, The University of Tokyo*)

Brown and beige adipocytes express uncoupling protein 1 (UCP1) and contribute to generate heat for maintaining body temperature in cold environment. We have reported that the environment-sensitive histone demethylase JMJD1A regulates obesity and metabolism, and also induces *Ucp1* expression using different mechanisms in acute and chronic cold response (Nat Commun 2015, 2018). Through badrenergic receptor (βAR) signaling induced by cold exposure JMJD1A is phosphorylated at serine 265 (S265). Phosphorylated JMJD1A facilitates heat generation in brown adipose tissue (BAT) in acute response to cold through the change of high-order chromatin structure. In the chronic response, JMJD1A induces the expression of thermogenic genes in white adipose tissue (WAT) through histone demethylation mediated by interaction with nuclear receptor, PPARγ. However, the role of histone demethylation in each adipose tissue still remains to be cleared. In this study, we generated *Jmjd1a*- H1122Y (HY) mice lacking enzymatic activity and analyzed the function of histone demethylation in BAT and WAT. The BAT function of HY mice maintained their body temperature as well as the wild type under acute cold exposure. In the WAT of HY mice under chronic cold exposure, the expression of the thermogenic genes was decreased, it suggested that the beige formation was disturbed. Furthermore, transcriptome analysis and chromatin analysis revealed that JMJD1A co-localized with nuclear receptor ERRα on chromatin and regulated the expression of mitochondrial function genes. Taken together, JMJD1A regulated the expression of thermogenic genes through a two-step regulatory mechanism in vivo, and new role of JMJD1A in regulating mitochondrial function genes mediated with ERRα was suggested.

### [OP03-03]
### The effect of claudin-15 deletion on digetstion and energy metabolism

*Wendy Leanne Hempstock[1,2], Nozomi Nagata[1], Noriko Ishizuka[1], Hisayoshi Hayashi[1] ([1]*Laboratory of Physiology, Graduate School of Nutritional and Environmental Sciences, University of Shizuoka,* [2]*Department of Nursing, School of Nursing, University of Shizuoka*)

The intestine plays important roles in nutrient absorption, electrolyte and water homeostasis, as well as a barrier to noxious substances. Tight junctions play an important role in nutrient uptake in the small intestine. Claudin-15 is a member of the claudin protein family and it forms a $Na^+$ pore in tight junctions (TJ) in the small intestine, cecum, and large intestine. Deletion of claudin-15 impairs nutrient absorption due to the loss of the intestinal $Na^+$ recycling mechanism which occurs mainly via claudin-15. In addition, claudin-15 deletion results in the formation of a megaintestine, suggesting that the intestine adapts to low nutrient absorption by increasing luminal diameter, villi length, as well as intestinal length. However, despite the decrease in nutrient absorption, claudin-15 knockout (*Cldn15* KO) mice are able to live and age seemingly normally. This study explored whether deletion of claudin-15 affects the metabolism of *Cldn15* KO mice. The luminal contents of the cecum were harvested for metabolomic analysis. *Cldn15* KO mice and WT controls were also subjected to indirect calorimetry using gas exchange metabolic cages, and body fat, specifically retroperitoneal and peri genital fat pads were investigated by microCT scanning.

### [OP03-04]
### Inhibition of mitochondrial fission decreases catecholaminestimulated lipolysis via impairment of glucose utilization in 3T3-L1 adipocytes.

*Nodoka Takeuchi[1], Kazuhiko Higashida[1], Naoya Nakai[1] ([1]*The University of Shiga Prefecture*)

Adipose tissue plays a major role in the regulation of energy metabolism. Recent studies have reported the relationship between mitochondrial dysfunction in adipocytes and various metabolic diseases. Mitochondria are dynamic organelles, which maintain their function by constant fusion and fission. However, the role of mitochondrial dynamics in energy metabolism, especially lipolysis, in adipocytes is still unknown. In this study, we differentiated 3T3-L1 cells in the presence of Mdivi-1, an inhibitor of the mitochondrial fission protein dynamin-related protein 1 (Drp1), and measured lipolysis. Since we have previously shown that glucose plays an important role in promoting lipolysis, we also evaluated glucose utilization in the presence of Mdivi-1. Treatment of Mdivi-1 decreased catecholamine-stimulated glycerol release, however, Mdivi-1 treatment didn't affect intracellular ATP levels or lipase expression. On the other hand, inhibition of Drp1 reduced glucose utilization and lactate production during lipolysis. Therefore, our results indicated that inhibition of mitochondrial fission decreases lipolysis via impairment of glucose utilization.

### [OP03-05]
### Energy-Sensing Histone Demethylase Facilitates Glycolysis for Lipid Storage during Adipogenesis

*Yoshihiro Matsumura[1], Eko Fuji Ariyanto[1], Yang Chaoran[2], Zhang Ji[1,2], Soga Tomoyoshi[3], Sakai Juro[1,2] ([1]*Research Center for Advanced Science and Technology, The University of Tokyo,* [2]*Tohoku University Graduate School of Medicine,* [3]*Institute for Advanced Biosciences, Keio University*)

Adipocytes uptake glucose and convert it to lipids when the intake energy becomes excessive. However, the mechanisms how adipocytes acquire such ability in response to energy availability is unknown. Here, we demonstrate that histone demethylase KDM3A mediates demethylation of histone H3K9 di-methylation (H3K9me2) from glycolysis genes in response to extracellular glucose to maximize glucose metabolism during adipogenesis. KDM3A is recruited to glycolysis genes in preadipocytes before differentiation induction. Extracellular glucose elevates cellular TCA cycle metabolites (e.g. α-ketoglutarate) and facilitated KDM3A-mediated demethylation of H3K9me2 from glycolysis genes. Glucose restriction or knockdown of KDM3 demethylases did not affect demethylation of H3K9 trimethylation from master adipogenic genes (i.e. *Cebpa, Pparg*) but inhibited demethylation of H3K9me2 from glycolysis genes, indicating gene-specific regulation of histone demethylation. These results indicate that KDM3A is an energy-sensing histone demethylase that regulates glucose metabolism and lipid storage during adipogenesis.

### Oral Presentation 4

### [OP04]
### Neurons, Synapses, Glia

March 16(Wed), 11:15 - 12:15, Room D

### [OP04-01]
### A neurological analysis of GAD67 knockout rats

*Dongyu Liu[1], Tomokazu Ohshiro[1], Kazuyuki Fujihara[2], Yuchio Yanagawa[2], Hajime Mushiake[1] ([1]*Tohoku University*, [2]*Grad Sch Med, Univ of Gumma*)

An inhibitory neurotransmitter, gamma-aminobutyric acid (GABA) is synthesized by two isozymes, glutamic acid decarboxylase (GAD) 65 and 67. GAD65 deficiency causes severe tonic-clonic seizures in mice and rats. It is not known, yet, wether GAD67 deficiency causes any epilepsy in these animal models. Here, we show that GAD67 knockout rats exhibit the spike-wave discharge (SWD), an epileptic form of brain activity, abundantly in EEG. The SWDs appear as early as in their infancy, while GAD67 heterozygous (+/-) rats and wild-type rats develop similar but much shorter and less frequent SWDs after their adulthood. SWDs are observed in human patient with absence epilepsy. It has been argued that SWDs in EEG may not indicate absence epilepsy in rats, because spontaneous SWDs have been demonstrated even in wild caught rats, and genetic absence seizure model rats could still respond to and recognize external stimuli while SWDs appear. We tested whether the GAD67 rats could respond to the acoustic stimulation (a loud buzzer tone), while we live-monitor the EEG and observe the behavior by video-taping. We found that SWDs were readily interrupted by the buzzer tone in control rats, but they were more difficult to be interrupted immediately in GAD67 rats. We also tested the effect of two major antiepileptic drugs on these SWDs. An acute administration of ethosuximide (250mg/kg, i.p.), which blocks the T-type Ca2+ channels, could completely suppress the SWDs in both GAD67 rats and control rats for 6~8h, suggesting an involvement of T-channels in SWD generation. On the other hand, valproate (VPA; 10g/L solution, per os) which reduces GABA metabolism, suppressed the SWDs only in the wildtype rats, indicating a difference in responsiveness to VPA. Interestingly, GAD67 rats never exhibited tonic-clonic seizures as in the GAD65 rats, which can be well suppressed by VPA. Our results suggest that the GABA produced by GAD67 along with T-channels is important to suppress SWDs, consistent with other studies showing the essential role of inhibitory neurotransmission in the cortico-thalamic loop to produce the SWDs and our finding that GAD67 is abundantly expressed in inhibitory neurons in the reticular thalamic nuclei. Our result also enigmatically indicates that the impairment of the inhibitory neurotransmission in GAD67 rats is rather resistant to VPA treatment.

### [OP04-02]
### Tonotopic tuning of GABAergic inhibition in avian cochlear nucleus.

*Rei Yamada[1], Mohammed Al-Yaari[1], Chikao Onogi[1], Ryota Adachi[1], Daiya Kondo[1], Hiroshi Kuba[1] ([1]*Department of Cell Physiology, Graduate School of Medicine, Nagoya University*)

Auditory pathways are organized tonotopically and process each frequency of sound in parallel circuit. The neurons in avian cochlear nucleus are functionally differentiated according to their tuning frequency for precise temporal coding. For example, the high frequency neurons (HF neurons) receive a large excitatory input for high-fidelity transmission, whereas the low frequency neurons (LF neurons) integrate multiple small inputs for improving time accuracy. In this study, we examined the tonotopic differentiations in feedforward GABAergic inhibition and explored its contribution to the temporal coding. We found that the unitary GABAergic currents were larger in number but smaller in size in the LF neurons. In addition, their reversal potential was close to the resting potentials, which enabled fine regulation of the firing. In the HF neurons, the unitary GABAergic currents were larger in size and highly depolarizing, which enabled powerful inhibition via potassium channel activation. Thus, we conclude that these differentiations of GABAergic synapses maximize the effects of inhibition at each frequency neuron and ensure the accurate temporal coding for wide frequency and intensity ranges.

### [OP04-03]
### Myelinating co-culture system with immortalized dorsal root ganglion neurons and Schwann cells as a beneficial tool for the study of peripheral sensory neuropathies

*Kazunori Sango[1], Shizuka Takaku[1] ([1]*Diabetic Neuropathy Project, Tokyo Metropolitan Institute of Medical Science*)

Co-culture models of neurons and Schwann cells have been used for the study of myelination during development, demyelinating disorders, and remyelination with axonal regeneration in the peripheral nervous system; in most of the previous studies, however, these cells were obtained by primary culture with immature animals. Spontaneously immortalized adult Fischer rat Schwann cells IFRS1 established in our laboratory have shown to possess fundamental ability to myelinate neurites in cocultures with adult rat dorsal root ganglion neurons, nerve growth factor-primed PC12 cells, and immortalized mouse motor neurons NSC-34. Our current investigation focuses on the establishment of stable co-culture system with IFRS1 cells and immortalized rat dorsal root ganglion neurons ND7/23. ND7/23 cells were seeded at a low density ($2 \times 10^3/cm^2$) and maintained for 5-7 days in serum-containing medium supplemented with non-essential amino acids and nerve growth factor (NGF; 10 ng/mL) and a Rho kinase inhibitor Y27632 (5 $\mu$M). Upon observation of neurite outgrowth under a phase-contrast microscope, the ND7/23 cells were exposed to an anti-mitotic agent mitomycin C (MMC; 1 $\mu$g/mL) for 48 h, then co-cultured with IFRS1 cells ($2 \times 10^4/cm^2$) and maintained in serum-containing medium supplemented with ascorbic acid (50 mg/mL), NGF (10 ng/mL), and ciliary neurotrophic factor (CNTF; 10 ng/mL). MMC pre-treatment was effective for the prevention of ND7/23 cell overgrowth, whereas CNTF appeared to alleviate the MMC cytotoxicity during the co-culture. Double-immunofluorescence staining carried out at day 21 of the co-culture showed peripheral myelin protein 22-immunoreactive IFRS1 cells surrounding $\beta$III tubulinimmunoreactive neurites emerging from ND7/23 cells. This co-culture system can be a beneficial tool to study the pathogenesis of a variety of peripheral sensory neuropathies and novel therapeutic approaches against them.

### [OP04-04]
### Microglial homeostasis through sympathetic nervous system may contribute to brain immunity under stress conditions

*Shuei Sugama[1], Yoshihiko Kakinuma[2] ([1]*International University of Health and Welfare*, [2]*Nippon Medical School*)

Microglia are immunocompetent cells in the brain that contribute to a wide variety of roles, such as monitoring or nurturing neuronal activity, structural remodeling of neurons, surveillance of the neuronal milieu, neuroinflammation, phagocytosis, and gliosis. Recent animal studies have shown that exposure to either acute or chronic stress induces robust microglial activation in the brain. The purpose of the present study was to investigate the underlying mechanism of brain microglial activation by acute stress. We examined the effects of the adrenergic receptors, beta and alpha type, on microglial activation using pharmacological intervention as well as adrenergic receptors knockout mice. The results demonstrated that the blockade of beta-receptor resulted in substantial inhibition on microglial activation in terms of morphology and cell count. However, the blockade of alpha type did not show any inhibition. Furthermore, in contrast to WT mice, double KO mice showed significant inhibition of stress-induced microglial activation in the brain. Thus, we suggest that noradrenaline may play a key role in inducing microglial activation through beta-type adrenergic receptor.

### Oral Presentation 5

### [OP05]
### Motor function, Plasticity

March 16(Wed), 11:15 - 12:15, Room J

### [OP05-01]
### Is the Oriental bodywork a practice to reactivate the body trunk motor function-Basal ganglia and MMC(Locomotion CPGs)? : A hypothesis

*Toshihiro Nukiwa[1], Yoshito Furuawa[1], Hatsumi Yoshii[1], Timur Ucmak[1], Hajime Mushiake[1] ([1]*Tohoku University*)

Practicing Nishino Breathing Methods (NBM) as a bodywork activity for decades, many trainees feel that the NBM causes an unique sense of impact and whole-body exhilaration especially by mutual manipulation called Taiki, but the physiological background is unclear. The undulation movement of the lamprey is controlled by the system of basal ganglia and spinal locomotion CPGs (Grillner S, 2020), which was recently identified as an evolutionarily old medial motor column (MMC) by neural gene expression. Similarly, the lateral motor column (LMC) has been identified as a motor system that evolved with fins and quadruped. The Oriental bodywork has a mysterious practice as single and a twoperson mutual practice such as Tui-shou (or push hands) in Tai Chi and Taiki in NBM. In contrast to the LMC system for the training of Western sports, Oriental bodywork likely reactivate the MMC system that controls the trunk motor muscles. Furthermore, in the latter, the involvement of a physiologically unknown mutual signaling (afferent path) is expected, and possible medical intervention can be applied to maintain motion capability in the global aging of the 21st century society. We will discuss this hypothesis using NBM as an example, introducing by movies of the Fascia-linked rotation breathing, paired signaling in mutual Taiki maneuver, that induces reactions related to locomotion, such as fast stepping.

### [OP05-02]
### Brainstem neural circuits for triggering saccades

*Mayu Takahashi[1], Yuriko Sugiuchi[1], Yoshikazu Shinoda[1] ([1]*Tokyo Medical and Dental University*)

In the brainstem saccade system, one group of neurons was different from others in that they show tonic activity during fixation, and stop firing during saccades in all directions (OPNs, omnipause neurons). Stimulation of the OPNs could prevent saccades, suggesting that pontine excitatory (EBNs) and inhibitory burst neurons (IBNs) are under inhibitory control by OPNs during fixation, and their inhibition must be suppressed for saccade triggering. Many researchers have tried to identify inhibitory interneurons that inhibit OPNs, but neural substrates for triggering saccades still remain unsolved. To determine the saccade triggering signal to suppress OPNs, we investigated inputs to OPNs and IBNs from the superior colliculi (SCs) by recording intracellular potentials in anesthetized cats. OPNs received monosynaptic excitation from the bilateral rostral SCs and terminated on EBNs and IBNs. In contrast, OPNs received disynaptic inhibition via IBNs from the caudal SC, because intracellular staining of single IBNs with HRP revealed their extensive axonal projection onto OPNs. These findings show that IBNs activated by caudal SC shut down OPN firing and help trigger saccades and suppress ('latch') OPN activity during saccades.

### [OP05-03]
### Influence of muscle contraction modes on the corticospinal excitability during an attentional focus task

*Amiri Matsumoto[1], Akari Ogawa[1], Chihiro Oshima[1], Keisuke Irie[1], Nan Liang[1] ([1]*Cognitive Motor Neuroscience, Department of Human Health Sciences, Graduate School of Medicine, Kyoto University*)

It is known that the external focus (EF) leads better motor performance rather than the internal focus (IF). It remains unclear, however, whether the muscle contraction modes contribute to the effectiveness of the attentional focus. By using transcranial magnetic stimulation (TMS), we examined the modulation of the corticospinal excitability with different muscle contraction modes under the EF and IF conditions. The healthy participants were asked to perform a dynamic or static right index finger abduction under the EF (focus on the pressure on the object) and IF (focus on the finger movement) conditions. TMS was applied over the motor hotspot of the first dorsal interosseous (FDI) muscle in the premotor time, and the motor evoked potential (MEP) was recorded from the FDI muscle. MEP amplitude with the EF condition was larger than that with the IF condition in the isotonic contraction task, while no difference was observed between the EF and IF conditions in the isometric contraction task. Our results suggest that the impact of attentional focus on the corticospinal excitability might be influenced by different muscle contraction modes (COI: NO).

### [OP05-04]
### Dynamic changes of spontaneous activities in cerebral cortex induced by electrical stimulation of the periodontal ligament.

*Shutaro Kobayashi[1], Kazunori O'Hashi[1], Masayuki Kobayashi[1] ([1]*Department of Pharmacology, Nihon University School of Dentistry*)

Chronic pain is characterized by persistent pain without signs of peripheral tissue damage. Recent noninvasive neuroimaging technology has revealed that spontaneous cortical activities plays a key role to diagnose patients suffering from the pain. However, mechanisms of the emergence of chronic pain-related spontaneous cortical activities remain to be established. In the present study, we investigated spontaneous cortical activities in sessions before and after electrical stimulation of the periodontal ligament (PDL) by applying wide-field and two-photon calcium imaging to anesthetized transgenic mice, whose excitatory neutrons expressed GCaMP6s. We first identified three cortical areas sequentially responding to PDL stimulation: primary (S1), secondary somatosensory (S2) and insular cortices (IC). We then found that spontaneous IC activities that exhibiting a similar cortical pattern to evoked activities by PDL stimulation were increased in the session after repetitive PDL stimulation. At the single-cell level, repetitive PDL stimulation augmented the synchronous neuronal activity. These results suggest that cortical plasticity induced by repetitive PDL stimulation increases IC activities similar to the PDL stimulationevoked response. This augmented nociceptive-related spontaneous activities may lead the induction of chronic pain.

### [OP05-05]
### Optical inactivation technology for GluA2/3 AMPA receptor.

*Susumu Jitsuki[1,2], Takuya Takahashi[2], Kiwamu Takemoto[1] ([1] *Mie University*, [2]*Yokohama City University*)

The AMPA type glutamate receptors (AMPA-Rs), which are well known to be important glutamate receptors for synaptic plasticity, are composed of variable combinations of four subunits, GluA1-4. The combinations of GluA1 homomer, GluA1/2 and GluA2/3 were known to be expressed in adult brain. While the GluA1 subunits of AMPA-Rs require plasticity-inducing stimulation to be driven into synapses and serve to strengthen neurotransmission, GluA2/3 complex continuously replace synaptic receptors in a manner that maintains transmission (Shi et al. Cell 2001, Takahashi et al. Science 2003 etc.). These findings suggest that AMPA-Rs complexes should have different physiological functions *in vivo*. To elucidate their complex-specific functions *in vivo*, we have developed an optical technology for acute inactivation of synaptic GluA1 homomeric AMPA-Rs *in vivo* by chromophore assisted light inactivation (CALI) method (Takemoto et al. Nat. Biotechnol. 2017). We found that GluA1 homomer requires acquisition of hippocampus-dependent fear memory. Based on this finding, we have currently developed a CALI method for GluA2/3. To achieve inactivation of GluA2/3, We screened GluA3 antibody by using flow cytometry and electrophysiology. We obtained antibodies that showed high CALI efficiency of GluA2/3. Using this antibody could be clarify GluA2/3-specific physiological functions *in vivo*.

### Oral Presentation 6

### [OP06]
### Embryology, Regenerative Medicine, Development, Growth, Aging

March 17(Thu), 10:45 - 11:45, Room D

### [OP06-01]
### Intracellular glucose metabolism is enhanced with activation of hypoxia-inducible factor-1α (HIF-1α) in a rat embryonic heart primordium after heartbeat initiation.

*Tatsuya Sato[1], Nobutoshi Ichise[1], Hiroyori Fusagawa[1,2], Hiroya Yamazaki[1], Taiki Kudo[1], Izaya Ogon[1,2], Noritsugu Tohse[1] ([1]*Department of Cellular Physiology and Signal Transduction, Sapporo Medical University School of Medicine,* [2]*Department of Orthopedic Surgery, Sapporo Medical UnivSapporo Medical University School of Medicine*)

**Background:** We have demonstrated that heartbeat in a rat embryonic heart begins at around embryonic day 10.0 (E10.0) via extracellular calcium influx through L-type calcium channels; however, it remains unclear how increased energy demand is covered after heartbeat initiation to maintain heartbeat.
**Methods:** Embryos of Wistar rats at E10.0 were divided into two groups by the heart primordium before and after heartbeat initiation and metabolic characteristics with their upstream signals were assessed.
**Results:** Principal component analysis of metabolomic analysis revealed that increased levels of ATP, a major product of glucose catabolism, reduced glutathione, a by-product of the pentose phosphate pathway, and increased level of GTP were the top three determinants in the heart primordium after heartbeat initiation, whereas phosphoribosyl pyrophosphate, fructose 6-phosphate, and fructose 1,6- bisphosphate, which are intermediate metabolites in nucleotide biosynthesis and glycolysis, were the top three metabolites contributing more to the total variance for the pre-heartbeat group. Measurement using an extracellular flux analyzer revealed that glycolytic capacity and ATP synthesis-linked mitochondrial respiration were significantly increased in isolated cells from the heart primordium after heartbeat initiation. The DNA-binding activity and protein expression level of hypoxia-inducible factor-1α (HIF-1α) were significantly higher in the post-heartbeat group than in the pre-heartbeat group. Finally, a glucose transporter and rate-limiting enzymes of the glycolytic and pentose phosphate pathways, which are HIF-1α-downstream targets, were upregulated in the heart primordium after heartbeat initiation.
**Conclusions:** The results indicate that the HIF-1α-mediated enhancement of glycolysis with activation of the pentose phosphate pathway, potentially leading to antioxidant defense and nucleotide biosynthesis, covers the increased energy demand in the heart primordium after heartbeat initiation.

### [OP06-02]
### Novel regulatory mechanism of hemogenic endocardium during cardiovascular development

*Norika Liu[1,2], Naofumi Kawahira[2], Yasuhiro Nakashima[3,2], Haruko Nakano[2], Susumu Minamisawa[1], Atsushi Nakano[1,2] ([1]*The Jikei University,* [2]*University of California, Los Angeles,* [3]*Kyoto University*)

A subset of endocardial cells is hemogenic during early embryogenesis. Hemogenic endocardial cells are enriched in the cushion and undergo endocardial-hematopoietic transition via Nkx2-5-dependent manner, suggesting that Drosophila *tinman*-dependent cardio-hematopoietic program is conserved in the mammals.
In order to further examine the regulatory network of Nkx2-5-dependent endocardial hematopoiesis, we analyzed scRNA-seq data from wildtype and Nkx2-5-null embryonic hearts. Consistent with previous observations, Nkx2-5-null hearts were devoid of clusters for hemogenic endocardium (e.g. $Runx1^+$, $Cd41^+$) and cushion endocardium (e.g. $Twist1^+$, $Msx1^+$). The analysis further revealed that genes related to Notch signaling pathway are significantly downregulated in Nkx2-5-null endocardium. To examine whether Notch signaling induces endocardial hematopoiesis downstream of Nkx2-5, we performed genetic rescue experiment by activating Notch intracellular domain (NICD) in Nkx2-5-null background. Overexpression of NICD not only restored the cushion formation but also drastically increased the number of hemogenic endocardial cell, resulting in the increase in macrophages in the cardiac valve region.
In summary, this study demonstrated that the Nkx2-5/Notch signaling axis plays a pivotal role in endocardial-hematopoietic transition during early embryogenesis.

### [OP06-03]
### Induction of hypothamic paraventricular nucleus and supraoptic nucleus neuron by direct reprogramming

*Toshiki Kameyama[1], Yoshinari Mera[1], Shunya Tsukamoto[1], Tsukushi Suzuki[1], Miho Kawata[1], Yu Kodani[1], Kanako Saito[1], Akira Nakashima[2], Hiroshi Nagasaki[1] ([1]*Department of Physiology, School of Medicine, Fujita Health University,* [2]*Department of Physiological Chemistry, School of Medicine, Fujita Health University*)

The hypothalamus, the most ventral part of the forebrain consisting of multiple nuclei and diverse neuronal subtypes, plays an important role in maintaining physiological homeostasis and controlling various behaviours such as food and water intake, body temperature regulation, and reproductive behaviour. Because of the importance of its function, disruption of hypothalamic function directly leads to disease. Hormone replacement therapy is generally used to treat patients; however, precise regulation is difficult to achieve. To overcome this problem, regenerative medicine is desired.
To induce the functional hypothalamic neuron from pluripotent stem cells in vitro, a serumfree floating culture of embryoid body-like aggregates with quick reaggregation (SFEBq) is now widely used. While SFEBq method has the advantage of mimicking the normal developmental process, it induces the differentiation of multiple neural subtypes simultaneously.
Recently, the direct neuronal reprogramming method, like induction of iPS cells, has been attracting attention. In this study, we report the successful induction of hypothalamic paraventricular nucleus and supraoptic nucleus neuron directly from mouse ES cells by the direct neuronal reprogramming method. With further improvement, we expect to be able to directly reprogram specific hypothalamic neurons from adult skin fibroblasts.

### Oral Presentation 7

### [OP07]
### Muscle, Physical fitness and sports medicine, Oral physiology

March 17(Thu), 10:45 - 11:45, Room E

### [OP07-01]
### mTORC1 inhibition during denervation-induced muscle atrophy affects mitochondrial dynamics

*Kazuki Uemichi[1], Takanaga Shirai[1,2], Riku Tanimura[1], Tohru Takemasa[1] ([1]*University of Tsukuba*, [2]*Japan Society for the Promotion of Science*)

Mechanistic target of rapamycin complex 1 (mTORC1) signaling is a molecular pathway that plays an important role in regulation of muscle protein synthesis, but is also activated during muscle atrophy caused by denervation. Mitochondrial dynamics, which involves mitochondrial fusion and fission, is an important mechanism for maintaining healthy skeletal muscle quality. We have demonstrated that mitochondrial dynamics during muscle hypertrophy is affected by mTORC1, but we do not know this will also happen during denervation-induced muscle atrophy. Therefore, we designed experiment to determine the role of mTORC1 on skeletal muscle mitochondrial dynamics under denervation. Denervation using sciatic nerve transection was performed on 7-week-old male ICR mice for 14 days. Intraperitoneal administration of rapamycin, an mTORC1 inhibitor, was performed every 24 hours after the surgery. The results showed that mTORC1 inhibition suppressed the increase in the expression of mitochondrial fissionrelated signaling associated with denervation, indicating that mTORC1 may play a role in the regulation of mitochondrial fission during denervation-induced muscle atrophy.

### [OP07-02]
### The role of vimentin cleavage and calpain in the $Ca^{2+}$-sensitization of vascular smooth muscle contraction

*Hiroko Kishi[1], Qian Lu[1], Tomoka Morita[1], Ying Zhang[1], Nan Li[1], Sei Kobayashi[2] ([1]*Department of Molecular and Cellular Physiology, Yamaguchi University Graduate School of Medicine*, [2]*Department of Advanced Preventive Medicine, School of Medicine, Yamaguchi University*)

Rho-kinase (ROK) modulates the phosphorylation level of myosin light chain (MLC) and plays a critical role in the signal transduction of $Ca^{2+}$-sensitization of vascular smooth muscle (VSM) contraction leading to vasospasm. Previously we identified sphingosylphosphorylcholine (SPC)/Fyn/ROK pathway which mediates $Ca^{2+}$-sensitization of VSM contraction. As possible downstream targets of Fyn tyrosine kinase, we identified vimentin by focused proteomics. Interestingly, SPC induced limited proteolysis of vimentin in human coronary artery smooth muscle cells (CASMCs) and VSM strips of the porcine coronary artery (PCA). Since vimentin is reported as the target of calpain, we examined the involvement of calpain. In CASMCs, SPC increased calpain activity, which was blocked by PD150606, a calpain inhibitor. Furthermore, PD150606 inhibited the SPC-induced VSM contractions but not high $K^{+}$ depolarization-induced $Ca^{2+}$-dependent contraction in PCA. In the present study, we investigated the effect of PD150606 in the SPC-induced ROK activation and myosin light chain (MLC) phosphorylation by western blot using antibodies against phosphorylated myosin phosphatase targeting subunit 1 (MYPT1) at Thr853 and phosphorylated MLC at Ser19. As the results, we revealed that PD150606 inhibited the phosphorylations of MYPT1 and MLC, suggesting that calpain is involved in the $Ca^{2+}$- sensitization of VSM contraction mediated by the SPC/Fyn/ROK pathway.

### [OP07-03]
### The effect of mouse serum under calorie restriction on mTOR signaling and mitochondrial biogenesis in C2C12 myotube

*Takanaga Shirai[1,2], Tomohiro Iwata[3], Riku Tanimura[3], Kazuki Uemichi[3], Tohru Takemasa[1] ([1]*Faculty of Health and Sport Sciences, University of Tsukuba*, [2]*Research Fellow of Japan Society for Promotion Science*, [3]*Graduate School of Comprehensive Human Sciences, University of Tsukuba*)

Calorie restriction (CR) reduces calorie intake without altering nutritional balance, and has many beneficial effects, such as improving oxidative metabolism and extending lifespan. On the other hand, CR causes a decrease in skeletal muscle mass and fat mass in correlation with food intake reduction. Therefore, the metabolic profile of the whole body as well as the tissues is also greatly altered during CR. We investigated whether changes in secretory factors in CR affects skeletal muscle cells. In this study, C57BL6/J male mice were used and were subjected to ad libitum (AL) or 60% CR for eight weeks. C2C12 myotubes were subsequently treated with media containing 10% mouse serum from AL or CR for 24 h. Eight weeks of CR decreased skeletal muscle mass and protein synthesis response compared with AL. Interestingly, myotube added CR serum elevated protein synthesis response compared with AL. Mitochondrial biogenesis and respiratory capacity were elevated in CR mice and CR serum treated cells. These results suggested that CR decreased protein synthesis response but secretory factors during CR can activate protein synthesis and mitochondrial biogenesis.

### [OP07-04]
### Plastic fate transition from pericyte to odontoblast

*Takehito Ouchi[1], Ryuya Kurashima[2], Maki Kimura[1], Yoshiyuki Shibukawa[1] ([1]*Department of Physiology, Tokyo Dental College*, [2]*Tokyo Dental College Suidobashi Hospital*)

Odontoblasts are terminal differentiated cells showing tall columnar shape, and insert their cellular processes into dentinal tubules. Recently, presence of pericytes located around odontoblasts and vascular networks of the dental pulp in the mouse incisor has been described (Khatibi Shahidi et al., 2015). Although it indicates that pericytes interact with odontoblasts in some reasons, its detailed profiles in physiological and pathological as well as developmental conditions are still remained unclear. To address these questions, we used acutely isolated rat odontoblasts and immortalized human odontoblast cell line, and analyzed cellular antigen of pericyte markers by immunostaining. These cells showed immunopositive for dentin sialophosphoprotein (DSPP) which is odontoblast specific marker protein. Pericyte marker NG2 was colocalized with DSPP in several cells of these cells, suggesting that subpopulation of NG2-positive pericytes definitely transited to odontoblasts. Further analysis revealed that both NG2-negative and -positive odontoblasts expressed mechanosensitive ion channel, Piezo1. Our data indicate that odontoblasts are classified into two distinct fractions, which are NG2-positive/Piezo1-positive odontoblasts and NG2-negative/Piezo1-positive odontoblasts. Additional detailed analysis on cellular plasticity, regulator of fate determination, and physiological functions will provide new insights of odontoblast differentiation from pericyte.

### [OP07-05]
### Effects of exercise intensity on white adipose tissue browning and its regulatory signals in mice

*Riku Tanimura[1], Leo Kibayashi[1,2], Takanaga Shirai[3,4], Tohru Takemasa[4] ([1]*Graduate School of Comprehensive Human Sciences, University of Tsukuba*, [2]*JIJI PRESS Ltd*, [3]*Research Fellow of the Japan Society for the Promotion of Science*, [4]*Faculty of Health and Sports Sciences, University of Tsukuba*)

Browning white adipose tissue (WAT) can produce heat by increasing mitochondria contents and expression of uncoupling protein 1 (UCP1) in response to cold exposure or exercise. Although exercise is known as a potent trigger for browning, the effects of exercise intensity on browning of WAT are unclear. We aimed to examine the effects of high or low intensity exercise on browning of WAT. We performed high or low intensity treadmill running on mice 3 days/week for 4 weeks. We found that 4 weeks running did not reduce wet weight of inguinal white adipose tissue (iWAT), but significantly reduced adipocytes size of iWAT regardless of exercise intensity. The protein expression levels of UCP1 were significantly increased in iWAT depending on exercise intensity. In addition, the protein expression levels of OXPHOS in iWAT were significantly increased by high intensity running. These results mean that high intensity exercise may be effective for increasing mitochondrial contents and heat production capacity in iWAT. In conclusion, we suggest that high intensity exercise is effective for browning of WAT.

### Oral Presentation 8

### [OP08]
### Nutritional and metabolic physiology, Thermoregulation

March 17(Thu), 10:45 - 11:45, Room F

### [OP08-01]
### Refeeding activates neurons in the dorsomedial hypothalamus to inhibit food intake

*Chitoku Toda[1] ([1]*Hokkaido University*)

[Objective] The regulation of food intake is a major research area in the study of obesity. Gene targeting studies have clarified the roles of hypothalamic neurons in feeding behavior, but the deletion of a gene has a long-term effect on neurophysiology. Hence, our understanding of short-term changes such as appetite under physiological conditions is still limited.
[Methods] Targeted recombination in active populations (TRAP) is a newly developed method for labeling active neurons by using tamoxifen-inducible Cre recombination controlled by the promoter of activity-regulated cytoskeleton-associated protein (Arc/Arg3.1). Transgenic mice for TRAP were fasted overnight, re-fed with normal diet and injected with 4-hydroxytamoxifen one hour after the refeeding to label the active neurons.
[Results] Fasting-refeeding activated and labeled neurons in the dorsomedial hypothalamus (DMH). Chemogenetic activation of the labeled DMH neurons decreased food intake and developed place preference. The labeled DMH neurons expressed prodynorphin (pdyn), gastrin-releasing peptide (GRP), cholecystokinin (CCK) and thyrotropin-releasing hormone receptor (Trhr) genes.
[Conclusions] We identified a novel cell type of DMH neurons that can inhibit food intake and promote feeding-induced positive valence.

### [OP08-02]
### The central roles of NPGL/NPGM system in energy homeostasis

*Kenshiro Shikano[1], Ikuko Morisaki[2], Ryoko Higa[1], Hitoshi Teranishi[1], Takaki Yahiro[3], Mitsuhiro Yoshimura[4], Toshikatsu Hanada[2], Kazuhiro Nakamura[3], Yoichi Ueta[4], Reiko Hanada[1] ([1]*Department of Neurophysiology, Faculty of Medicine, Oita University,* [2]*Dept Cell Biol, Grad Sch Med, Oita Univ,* [3]*Dept Integrative Physiol, Grad Sch Med, Nagoya Univ,* [4]*Dept Physiol, Sch Med, UOEH*)

Hypothalamus is the center of energy metabolism and feeding behavior, and several hypothalamic neural substrates regulate energy metabolism. Neurosecretory protein GL (NPGL) and neurosecretory protein GM (NPGM) were found as a novel neuropeptide from hypothalamus in 2014. Previous data showed that NPGL or NPGM administration promotes feeding behavior and fat accumulation. However, the mechanisms of energy metabolism mediated by NPGL/NPGM system have not yet been totally elucidated. To investigate the loss of function of NPGL/NPGM system, we have established NPGL and NPGM double knockout mice (NPGL/NPGM dKO). NPGL/NPGM dKO had a lean phenotype under a high fat diet condition because of decreased food intake and increased energy expenditure compared with wild type mice (WT). Furthermore, NPGL/NPGM dKO showed not only decreasing fat deposition but also augmenting thermogenic uncoupling protein 1 expression in brown adipose tissue (BAT). Mitochondrial density of BAT was higher in NPGL/NPGM dKO mice than WT mice. These results imply that endogenous NPGL/NPGM system has important roles in central thermoregulation.

### [OP08-03]
### The role of TH neurons in the PVH in the control of feeding behavior

*Winda Ariyani[1], Haruka Tsuneoka[1], Chiharu Yoshikawa[1], Hiroshi Ichinose[2], Tadahiro Kitamura[1], Daisuke Kohno[1] ([1]*Metabolic Signal Research Center, IMCR, Gunma University* , [2]*School of Life Science and Technology, Tokyo Institute of Technology*)

The paraventricular hypothalamus (PVH) plays a vital role in feeding regulation. Our previous study showed that the deletion of DNA methyltransferase 3a (Dnmt3a) in the PVH highly increased the tyrosine hydroxylase (TH) expression level in the PVH and induced obesity. This study explored the role and characteristics of TH neurons in the PVH in the context of feeding behavior. The PVH specific Th knockout mice (Th$^{lox/lox/}$Sim1- Cre) did not show the difference in body weight, oxygen consumption, and respiratory exchange ratio compared to control mice (Th$^{lox/lox}$). However, Th$^{lox/lox/}$Sim1-Cre mice had different feeding pattern. In addition, Th$^{lox/lox/}$Sim1-Cre mice had higher locomotor activity during the dark period. Immunohistochemical analysis showed that TH neurons in PVH were surrounded by NPY and POMC fibers. Moreover, TH neurons were highly colocalized with DAT and GAD67. These results suggest that TH neurons in PVH are dopaminergic/GABAergic neurons that are potentially a part of the melanocortin pathway and play an important role in controlling feeding behavior.

### [OP08-04]
### The exposure to dashi through the lactating mother alters appetite of offsprings for oil during their adulthood.

*Shunsuke Fushimi[1], Tsutomu Sasaki[1] ([1]*Laboratory of Nutrition Chemistry, Division of Food Science and Biotechnology Graduate School of Agriculture, Kyoto University*)

[Background] Japanese diets are eaten over generations despite their low-fat content. Although the ingestion of dashi, a characteristic of the Japanese diet, has been reported to have a negative correlation with indicators of obesity, how dashi controls remains elusive. This study hypothesized that maternal exposure to dashi affects children's appetite. [Methods] Pregnant mice (C57BL6) were exposed to 10% concentrated bonito broth for a specific period (embryonic & lactation, embryonic, lactation). The control (non-exposed) group received water ad libitum. After pups reached adulthood, a licking test was performed to assess appetite in terms of palatability and motivation. To assess the appetite for oil., a 2.5% solution of corn oil was presented in the light phase for 15 minutes to assess the appetite for oil. [Results] Burst size and the number of bursts, representing palatability and motivation, respectively, were significantly increased in the embryonic & lactation and the lactation groups compared to the non-exposed and the embryonic groups. [Conclusion] The exposure to dashi through the lactating mother alters appetite of offsprings for oil during their adulthood.

### [OP08-05]
### Two-weeks heat exposure alters the heat-escape behavior and the possible involvement of the thermo-TRP channels

*Yuta Masuda[1], Naotoshi Sugimoto[2], Kei Nagashima[3] ([1]*Graduate School of Human Sciences, Waseda University,* [2]*Faculty of Medicine, Institute of Medical, Pharmaceutical and Health Sciences,* [3]*Faculty of Human Sciences, Waseda University*)

Introduction. We aimed to evaluate the heat-escape behavior in heat exposure mice and tested the hypothesis that the expression of TRPV1 channels affects the behavioral response. **Methods.** Male C57BL/6 mice (n=22; age, 7 w) were divided to heat exposure and control groups (HE and CON groups, respectively). They were individually housed for 2 w at 33℃ and 25℃ in the HE and CON groups, respectively. After the period, mice were assessed behavioral response with the cross-shaped system for 90 min, which consisted of five Peltier boards (10×10 cm) arranged in a cross. The temperature setting was that any one of 4 boards located in the end of the cross was 32℃ and the others 38℃. The board set at 32℃ was randomly changed every 5 min. Abdominal temperature ($T_{abd}$) and the ratio to the total at which mice stayed on the 32℃ board were assessed (heat-escape behavior). Mice were killed by overdose anesthetics and the dorsal root ganglia was excised, and the protein expression of the TRPV1 and V4 were evaluated by immunohistochemistry and Western blotting. **Results & Discussion.** The ratio of 32℃ selection was smaller in the HE than in the CON group (58 ± 12 and 78 ± 11%, respectively). The expression of the TRPV1 was smaller in the HA than in the CON group, but there were no differences in that of the TRPV4. These results may suggest that continuous heat exposure changes heat-escape behavior. Moreover, decreased expression of the TRPV1 channels may be involved in the mechanism in a part.

### Oral Presentation 9

### [OP09]
### Membrane transport, Others

March 17(Thu), 10:45 - 11:45, Room G

### [OP09-01]
### Bicarbonate transport of airway surface epithelia in luminallyperfused mice bronchioles

*Libin Liu[1], Akiko Yamamoto[1], Makoto Yamaguchi[1], Itsuka Taniguchi[1], Nao Nomura[1], Miyuki Nakakuki[1], Yuka Kozawa[1], Mayuko Higuchi[1], Erina Niwa[1], Tsutomu Tamada[2], Hiroshi Ishiguro[1] ([1]*Dept Human Nutrition, Grad Sch Med, Nagoya Univ*, [2]*Dept Respiratory*)

$HCO_3^-$ concentration of airway surface liquid is important for antimicrobial activity and mucociliary clearance in airway. In the present study, bronchioles (150-180 μm diameter) were dissected from the lungs of wild-type and $Cftr^{tm1Kth}$ mice (CF mice). The lumen was microperfused using concentric holding and perfusion pipettes. Epithelial cells were loaded with BCECF-AM from the lumen and changes in intracellular pH ($pH_i$) were measured by microfluorometry at 37℃.
When the bath and lumen were first perfused with $HCO_3^-$-buffered solution (25 mM $HCO_3^-$-5% $CO_2$) and the luminal perfusate was switched to $HCO_3^-$-$CO_2$-free Hepes-buffered solution, $pH_i$ transiently increased due to $CO_2$ diffusion out of the cell and then gradually decreased due to $HCO_3^-$ secretion. Stimulation with forskolin (5 μM) accelerated the rate of pHi decline. The presence of $CFTR_{inh}$-172 (5 μM), $H_2DIDS$ (200 μM), and amiloride (1 μM) in the lumen significantly inhibited forskolin-accelerated $pH_i$ decline. In bronchioles from CF mice, basal and acetylcholine-stimulated $HCO_3^-$ secretion was substantially impaired, but transient acceleration of $HCO_3^-$ secretion by forskolin was comparable to wild-type bronchioles.
In summary, our data indicate that CFTR, $Cl^-$-$HCO_3^-$ exchanger and ENaC are involved in $H^+/HCO_3^-$ transport across the apical membrane of airway surface epithelia in mice distal airway. The data of CF mice suggested the presence of a crosstalk of cAMP- and $Ca^{2+}$- mediated pathways of $HCO_3^-$ secretion.

### [OP09-02]
### Inhibition of translocation of intracellular $Na^+$,$K^+$-ATPase α3-isoform by cardiac glycosides in cancer cells

*Takuto Fujii[1], Mizuki Kato[1], Takahiro Shimizu[1], Shushi Nagamori[2], Keiichi Koizumi[3], Junya Fukuoka[4], Yoshiaki Tabuchi[5], Akira Sawaguchi[6], Okumura Tomoyuki[7], Kazuto Shibuya[7], Tsutomu Fujii[7], Hiroshi Takeshima[8], Hideki Sakai[1] ([1]*Dept. Pharm. Physiol., Fac. Pharm. Sci., Univ. Toyama*, [2]*Dept. Lab. Med., Jikei Univ. Sch. Med.*, [3]*Lab. Drug Dis. Dev. for Pre-disease, Univ.*, [4]*Dept. Pathol., Nagasaki Univ.*, [5]*Div. Mol. Genet. Res., Life Sci. Res. Centr., Univ. Toyama*, [6]*Dept. Anat., Fac. Med., Univ. Miyazaki*, [7]*Dept. Surg. Sci., Fac. Med., Univ*, [8]*Grad. Sch. Pharm. Sci., Kyoto Univ.*)

$Na^+$,$K^+$-ATPase α3-isoform (α3NaK) is abnormally expressed in human cancer cells. We previously suggested that α3NaK has an ATP-hydrolyzing (ATPase) activity in the tissues and cells of human liver cancers. Recently, we found that the α3NaK-expressin g intracellular vesicles translocate to the plasma membrane upon cancer cell detachment, and that α3NaK has an essential role for escaping apoptosis of floating cancer cells. Here, we report that cardiac glycosides, such as ouabain and oleandrin, were effective for inhibiting the α3NaK translocation in the cancer cells. The [$^3$H]-ouabain uptake was increased in a time- and temperature-dependent manner in the cancer cells. Interestingly, oleandrin significantly inhibited the detachment-induced $Ca^{2+}$-mobilization which elicits α3NaK translocation. In addition, increase in the membrane capacitance corresponding to increased exocytosis was observed upon cancer cell detachment and significantly inhibited by oleandrin. These results suggest that the cardiac glycoside-induced inhibition of ATPase activity of intracellular α3NaK may elicit apoptosis of floating cancer cells.

### [OP09-03]
### Peroxisome-mediated cholesterol trafficking into primary cilium membrnae

*Tatsuo Miyamoto[1], Kosuke Hosoba[2], Takashi Yamamoto[2], Takeshi Itanashi[3], Atsuko H Iwane[3] ([1]*Department of Molecular and Cellular Physiology, Yamaguchi University Graduate School of Medicine*, [2]*Graduate School of Integrated Sciences for Life, Hiroshima University*, [3]*RIKEN Center for Biosystems Dynamics Research*)

Primary cilium is an antenna-like organelles on the surface of most mammalian cells that receive extracellular mechanical and chemical information. In the context of embryogenesis and carcinogenesis, primary cilia mediate sonic hedgehog (Shh) signal. Cellular cholesterol acts as a direct activator of a seven-transmembrane oncoprotein called Smoothened (Smo) to induce Smo accumulation on the ciliary membrane where it transduces the Shh signal. However, how cholesterol is supplied to the ciliary membrane remains unclear. Here we demonstrate that peroxisomes supply cholesterol into the ciliary membrane. Zellweger syndrome is a peroxisome-deficient hereditary disorder with the typical ciliopathy-associated features such as polycystic kidney and retinitis and cells from these patients showed a reduced cholesterol level in the ciliary membrane. Reverse genetics approaches revealed that the GTP exchange factor Rabin8, the Rab GTPase Rab10, and the microtubule minus-end-directed kinesin KIFC3 form a peroxisomeassociated complex to control the movement of peroxisomes along microtubules to the ciliary pocket, and that a cholesterol transfer protein ORP3 is located at the ciliary pocket to uptake cholesterol from the peroxisomes. These findings suggest that insufficient ciliary cholesterol levels underlie ciliopathy-related features in Zellweger syndrome.

### [OP09-04]
### Research on the regulatory factors involved in differentiation of multiciliated ependymal cells

*Takuya Hirao[1], Beak Gyu Kim[1], Kotoku Kawaguchi[1], Shinji Asano[1] ([1]*College of Pharmaceutical Sciences, Ritsumeikan University*)

Multiciliated ependymal cells (MCCs) lining on the ventricular surface have essential roles in cerebrospinal fluid flow. To study the development and functional regulation of MCCs precisely, it is necessary to prepare the in vitro polarized primary culture system which reflects the ependyma in vivo. In this study, we prepared the primary culture of mouse MCCs using a permeable support filter and studied the roles of FBS and cytokines in differentiation.
The cells prepared from the whole brain of a newborn mouse were proliferated in the medium containing 10% FBS and seeded on Transwell permeable support filter. We studied the effects of FBS and cytokines in the upper chamber (ventricle side) on the differentiation of MCCs.
The percentage of MCCs was gradually increased in 4 weeks of culture. The number of MCCs cultured in FBS-free conditions was significantly higher than that cultured in 10 % FBS-containing conditions. These results suggest that FBS inhibited the differentiation of MCCs. TGF-b which is one of the major cytokines in FBS also inhibited the differentiation. Differentiation of MCCs was inhibited by FBS in the ventricles side. TGF-b also inhibited the differentiation of MCCs.

### [OP09-05]
### Analysis of the dynamics of insulin secretory granules

*Daisuke Ohshima[1], Yoshinori Mikami[1], Taichiro Tomida[1], Satomi Adachi-Akahane[1] ([1]*Div. Phys., Toho Univ. Med.*)

Calcium signaling through voltage-dependent calcium channel (VDCC) is essential for insulin secretion from pancreatic β-cells. Fine-tuning of calcium signaling is required for sufficient insulin secretion. However, the details of the molecular mechanism have still been unclear. Here, we analyzed the dynamics of insulin secretory granules in order to clarify the relationship to calcium signaling. We generated MIN6 cell lines stably expressing mCherry-labeled insulin and calcium indicator, GCamp7. We stimulated cells with high glucose (25 mM) to induce calcium signaling and insulin exocytosis. We acquired the time-lapse imaging using total internal reflection fluorescence microscopy (TIRF) to trace the behavior of insulin secretory granules just below the cell membrane. For the analysis of imaging data, we used machine learning as a novel approach to detect and track the insulin secretory granules. We found novel features of the calcium-dependent dynamics of insulin secretory granules. We will discuss them in detail under various cellular conditions.

### Oral Presentation 10

### [OP10]
### Behabior, Biological rhythm, Sleep

March 17(Thu), 10:45 - 11:45, Room I

### [OP10-01]
**Single neuronal activity in the dorsomedial hypothalamic nucleus of rats across sleep-wake cycles**

*Kazumi Takahashi[1], Yumiko Kato[1], Satoshi Eifuku[1] ([1]*Fukushima Medical University*)

The dorsomedial hypothalamic nucleus (DMH), which consists of considerably various neurochemical subpopulations, is a center of autonomic functions and involved in instinctive behaviors. It is known that optogenetic activation of two different populations of galanin-expressing GABA neurons in the DMH induces either slow-wave sleep (SWS) or paradoxical sleep (PS). However, characteristics of spontaneous activity of entire DMH neurons across sleep-waking cycles have not been elucidated. We recorded state dependent changes in neuronal activity of the DMH through a glass electrode along with cortical EEG, neck muscle activity and electrocardiogram in head-restrained rats (n = 7). Thirteen of 54 successfully recorded neurons (24 %) showed the highest firing rate during PS, and their tonic activity started prior to PS onset. The remaining 41 neurons were wakefulness (W)-active (n = 12, 22 %), W/SWS-active (n = 5, 9 %), W/PS-active (n = 4, 7 %), SWS/PS-active (n = 4, 7 %), SWS-active (n = 1) and no-changed (n = 15, 28 %). Seven neurons exhibited preceding activity to transient elevation of heart rate during SWS (n = 2) or during PS (n = 5). These results suggest that some subpopulations of DMH neurons may play important roles both in generation of sleep/wake states and in control of the sympathetic nervous system.

### [OP10-02]
**Vasopressin neurons in the paraventricular hypothalamus promote wakefulness via lateral hypothalamic orexin neurons**

*Md Tarikul Islam[1], Florian Rumpf[1,2], Yusuke Tsuno[1], Shota Kodani[1], Takashi Maejima[1], Michihiro Mieda[1] ([1]*Kanazawa University*, [2]*University of Wuerzburg*)

Arginine vasopressin neurons in the paraventricular hypothalamus ($PVH^{AVP}$) regulate various physiology and behaviors, such as body-fluid homeostasis, stress response, social interaction, and feeding. Changes in arousal state often accompany these $PVH^{AVP}$ mediated adaptive responses. However, the role of $PVH^{AVP}$ neurons in sleep-wake regulation has remained unknown. Here, we report the involvement of these neurons in arousal promotion. Optogenetic and chemogenetic activation of $PVH^{AVP}$ neurons promoted wakefulness. In contrast, chemogenetic inhibition of $PVH^{AVP}$ neurons reduced wakefulness. Monosynaptic rabies virus tracing revealed that $PVH^{AVP}$ neurons receive projections from multiple brain regions involved in sleep-wake regulation. We observed dense projections of $PVH^{AVP}$ neurons in the lateral hypothalamus, close to orexin ($LH^{Orx}$) neurons. The optogenetic stimulation of $PVH^{AVP}$ neuronal fibers in the LH promoted wakefulness as well. Blocking orexin receptors attenuated the arousal effect of $PVH^{AVP}$ neuronal activation drastically, suggesting LHOrx neurons mediate the arousal effect of $PVH^{AVP}$ neurons. Moreover, $PVH^{AVP}$ neurons mediated the arousal induced by the melanocortin agonist, at least partially. Our data suggested that $PVH^{AVP}$ neurons promote wakefulness via $LH^{Orx}$ neurons in the physiological regulation of sleep-wake and melanocortin-induced arousal.

### [OP10-03]
**Role of estrogen receptor beta expressing neurons in the medial amygdala in the control of sexual preference in male mice.**

*Satoshi Takenawa[1], Yutaro Nagasawa[2], Aki Takahashi[1], Sonoko Ogawa[1]
([1]*Laboratory of Behavioral Neuroendocrinology, University of Tsukuba*, [2]*Supportive Center for Brain Research, National Institute for Physiological Sciences*)

Sexual preference is known to be important for choosing the most appropriate target to exhibit typical sexual and aggressive behaviors in male mice. Our previous study has demonstrated that the existence of estrogen receptor beta (ERβ) in the medial amygdala (MeA) is necessary for the male to show behavioral preference towards a receptive female (RF). However, the exact role of ERβ neurons ($ER\beta^+$ neurons) expressed in the MeA for the control of sexual preference is not known. In this study, we first established a mice line (ER β-iCre mice) to specifically target $ER\beta^+$ neurons. We then performed a series of experiments including recording of neuronal activity of MeA $ER\beta^+$ neurons during sexual preference tests and examination of effects of chemogenetic manipulation of $ER\beta^+$ neurons on preference behavior. We found neuronal activity of $ER\beta^+$ neurons in the MeA was clearly different in preference tests for a RF vs a non-receptive female (XF) from those for a RF vs an intact male (IM). Inactivation of MeA $ER\beta^+$ neurons affected male preference toward RF in RF vs XF tests, but not in RF vs IM tests, and neuronal activity of brain areas on the downstream pathways of the MeA. These findings suggest that activation of $ER\beta^+$ neurons in the MeA plays an important role in the control of male sexual preference toward receptive female mice. (Supported by KAKENHI #15H05724 to SO.)

### [OP10-04]
**Involvement of trabecular meshwork phagocytic suppression by sympathetic norepinephrine in nocturnal intraocular pressure rise**

*Keisuke Ikegami[1], Satoru Masubuchi[1] ([1]*Department of physiology, Aichi Medical University*)

Intraocular pressure (IOP) is important in glaucoma development and depends on aqueous humor (AH) dynamics, involving inflow from the ciliary body and outflow through the trabecular meshwork (TM)/Schlemm' s canal. IOP has a circadian rhythm entrained by sympathetic noradrenaline (NE) or adrenal glucocorticoids (GCs). Here, we investigated the involvement of GC and NE in AH outflow. Pharmacological prevention of inflow/ outflow in mice indicated an increase in AH outflow during diurnal low IOP. Although TM phagocytosis can determine AH drainage, real-time monitoring of phagocytosis using immortalized human TM cells (iHTMCs) revealed a non-self-sustained inhibitory effect of only NE stimulation. Signaling pathway analysis using a pharmacological approach and RNA interference in iHTMC identified β1-adrenergic receptor (AR)-mediated cAMP-EPACSHIP1 signal activation by ablation of phosphatidylinositol triphosphate, which is necessary for phagocytic cup formation. Furthermore, pharmacological instillation in mice was performed to assess the role of β1-AR-EPAC-SHIP1 pathway in nocturnal IOP rise. These results suggest that nocturnal IOP induction is partially regulated by this pathway. This first demonstration of TM phagocytosis suppression by NE could be useful in glaucoma management.

### Oral Presentation 11

### [OP11]
### Endocrine, Stress, Environmental physiology

March 17(Thu), 10:45 - 11:45, Room J

### [OP11-01]
### Functional analysis of a lncRNA THRB-AS2

*Sumiyasu Ishii[1], Noriyuki Koibuchi[1] ([1]*Department of Integrative Physiology, Gunma University Graduate School of Medicine*)

**Background:** Long non-coding RNAs (lncRNAs) have diverse functions. Here, we describe a novel lncRNA identified in a patient with syndrome of resistance to thyroid hormone. **Methods:** RNAs from leukocytes of a patient with resistance to thyroid hormone were sequenced and a novel lncRNA from the antisense strand of the thyroid hormone receptor beta (*THRB*) gene (THRB-AS2) was identified. The levels of THRBAS2 were examined by RT-PCR. Transcriptional regulation by THRB-AS2 was assessed by reporter assays. Knockdown experiments were performed in THP-1 cell line. **Results:** THRB-AS2 was expressed in both patient and control leukocytes, indicating that THRB-AS2 is not involved in the etiology of the disease. THRB-AS2 stimulated transcription from multiple promoters irrespective of thyroid hormone response elements or thyroid hormone. THRB-AS2 was highly expressed in spleen. The levels of THRB-AS2 decreased after differentiation of THP-1 cells. Depletion of THRB-AS2 did not affect the mRNA levels of THP-1 cell differentiation markers. **Conclusion:** THRB-AS2 might be a marker of undifferentiation in THP-1 cells, although its function is not fully understood yet. **COI:** No.

### [OP11-02]
### Roles of PMP22 in the alterations of muscle and bone induced by exercise in mice

*Miku Kawaguchi[1], Naoyuki Kawao[1], Masafumi Muratani[2], Takafuji Yoshimasa[1], Ishida Masayoshi[1], Yuya Mizukami[1], Ohira Takashi[1], Kaji Hiroshi[1] ([1]*Department Physiolgy & Regen Med, Kindai Univ Fac Med*, [2]*Department of Genome Biology, University of Tsukuba*)

Exercise is important for the prevention of sarcopenia and osteoporosis. Although the interactions between muscle and bone have been recently noted, myokines linking muscle to bone during chronic exercise remain unknown. Herein, we identified peripheral myelin protein 22 (PMP22), which is known as Charcot-Marie-Tooth disease-related gene, as a novel humoral factor whose expression was induced by chronic treadmill exercise for 8 weeks in the gastrocnemius muscles of mice from an RNA sequence analysis. Chronic exercise significantly blunted ovariectomy-induced bone loss in mice. PMP22 suppressed osteoclast formation as well as the expressions of NFATc1, cathepsin K and DC-STAMP induced by RANKL in mouse bone marrow cells. Moreover, it suppressed the mitochondrial biogenesis and ERK1/2, but not p38MAP kinase, in the presence of RANKL in these cells. On the other hand, PMP22 significantly inhibited the differentiation, alkaline phosphatase activity and mineralization in mouse osteoblasts. A simple regression analysis revealed that PMP22 mRNA levels in the gastrocnemius muscles were positively related to cortical bone mineral density at the femurs of mice. In conclusion, we first showed that PMP22 is a novel myokine linking muscle to bone induced by chronic exercise in mice.

### [OP11-03]
### Estradiol triggers cell differentiation and matrix mineralization in osteoblast-like cell line MC3T3-E1 under the ultra-low estrogen concentration environment.

*Hiraku Suzuki[1], Sumiyasu Ishii[1], Izuki Amano[1], Yuki Fujiwara[1], Noriyuki Koibuchi[1] ([1]*Gunma University*)

It is reported that reduction of estrogen secretion increases women's risk for osteoporosis and fractures, thus estradiol therapy is effective in the prevention and the treatment of those bone disorders. The function of estrogen is well known to suppress the bone resorption by osteoclast; however, it is unclear for bone forming cell, osteoblast. In this report, we focused on the estrogen included in the fetal bovine serum (FBS), that is essential for differentiation of the bone forming cell. Removal of the estrogen from culture as possible enabled us to show that 1-10nM estradiol stimulation induces matrix mineralization, final stage of bone formation, in osteoblast like cell line MC3T3- E1 under the culture condition with the "stripped FBS". Under normal condition, bone forming cells gradually differentiate in an autocrine manner *in vitro*. MC3T3-E1 cultured in the medium including "stripped FBS" did not express some functional proteins including bone sialo-protein (BSP) and osteocalcin (OCN) enough without estradiol stimulation. Our method and the results may contribute to elucidation of bone metabolism and development of osteoporosis treatment.

### [OP11-04]
### Possible relay projection to the medulla from the hypothalamus on the pressor response during social defeat stressin rats

*Mio Matsuyama[1], Joji Horiuchi[1] ([1]*Department of Biomedical Engineering, Toyo University*)

It has been shown that the sympathetic vasomotor pathway of psychological stress is mediated via neurons in the rostroventral medulla (RVM) indirectly from the hypothalamic stress center. In our previous study, we have indicated that the relaying area to the RVM is in the midbrain lateral/ventrolateral periaqueductal grey matter (l/vlPAG). Also, the excitatory amino acid injection into the l/vlPAG caused decent increases in blood pressure and the renal sympathetic activity in anesthetized rats. In the present study, direct projections to the l/vlPAG and distribution of c-Fos, a neuroexcitatory marker, expressed neurons were investigated during the social defeat stress (SDS) in conscious rats. The rat was injected FluoroGold (FG), a neural tracer, into the unilateral l/vlPAG and then exposed to the SDS. The double-labeled (c-Fos and FG) neurons were locally distributed within the dorsomedial area (DMH) and the perifornical area (PeF) in the hypothalamus. Therefore, these results suggested that the pressor response during acute psychological stress, like the SDS, may be mediated from the hypothalamus to the medulla via neurons in the l/vlPAG.

### [ST03-05]
#### ([OP11-05])
### The space flight induces the morphological changes of the lipid droplet in the liver hepatocyte of mouse.

*Takanobu Haraguchi[1], Hiroki Bochimoto[1], Daisuke Kondoh[2], Susumu Minamisawa[1] ([1]*Division of Aerospace Medicine, Department of Cell Physiology, The Jikei University School of Medicine*, [2]*Laboratory of Veterinary Anatomy, Obihiro University of Agriculture and Veterinary Medicine*)

**Abstract**
**Background:** Although space flight affects the lipid metabolism, it is equivocal whether microgravity directly causes the changes. To solve the problem, we analyzed the ultrastructural changes occurred in the lipid droplet (LD) in hepatocytes reflecting with their lipid metabolism of mice on board the international space station (ISS).
**Methods:** Six of C57BL/6 J male mice were kept under microgravity (MG) or on artificial earth-gravity by using centrifugation cages (AEG) in ISS for 35 days. In addition, 6 mice were kept on ground as control. Two days after landing, all mice were euthanized and dissected, the liver tissues were excised and fixed by fixative of 4 % paraformaldehyde, and were morphologically analyzed by electron microscopy.
**Results:** The number of LD in hepatocytes were increased after spaceflight under both of MG and AEG groups compared with control group. However, the LD size of hepatocytes was increased in only MG group.
**Conclusion:** Our data suggest that the non-microgravity-related stress of space flight increased the number of LD in hepatocytes. Additionally, exposure to artificial earth-gravity may reduce the risk of the perturbation of lipid metabolism in ISS because only the microgravity of spaceflight lead to the increase in the size of LD in hepatocytes.

### Oral Presentation 12

### [OP12]
### Ion channels, Receptors

March 18(Fri), 8:30 - 9:30, Room I

### [OP12-01]
### The effects of estrogen on mouse and human TRPA1 currents.

*Mami Kato[1,2], Yasunori Takayama[1], Masataka Sunagawa[1] ([1]*Dept Physiol, Showa Univ Sch Med,* [2]*Dept Mol & Syst Pharmacol, Grad Sch Pharm Sci, Kyushu Univ*)

Transient receptor potential ankyrin 1 (TRPA1), a non-selective cation channel, is known that a sensor for wasabi, cold temperature, and some endogenous gases including oxygen. The activation of TRPA1 induces neuronal excitation of primary sensory neurons, and modulates physiological functions such as pain and itch. We previously reported the inhibitory effect of estrogen on transmembrane 16A (TMEM16A), which is a calciumactivated chloride channel involved in pain generation. Here, we investigated whether the four endogenous estrogens including estrone (E1), estradiol (E2), estriol (E3), and estetrol (E4) inhibit TRPA1 currents. In this experiment, we performed whole-cell patch-clamp recordings in HEK293T cells expressing mouse or human TRPA1. In these results, E3 inhibited mouse TRPA1 currents under calcium free bath solution condition, although E3 did not inhibit mouse TRPA1 currents under bath solution containing calcium (2 mM) condition. In contrast, E3 activated human TRPA1 under bath solution containing calcium condition. E3 is highly synthesized during pregnancy. Thus, our findings indicate that human TRPA1 activation by E3 could be involved in physiological functions during pregnancy.

### [OP12-02]
### Experimental and numerical investigations of brady-arrhythmogenicity induced by a gain-of-function mutation in TRPM4 channel

*Yaopeng Hu[1], Qin Li[2], Yanghua Shen[2], Takayuki Fujita[1], Xin Zhu[2], Ryuji Inoue[1] ([1]*Dept. Physiol., Sch. Med., Fukuoka Univ.,* [2]*Dept. Biomed. Info. Technol., Aizu Univ.*)

The $Ca^{2+}$-activated cation channel TRPM4 is abundantly expressed in the heart. It is reported that a single gain-of-function mutation identified in its distal N-terminus, ‘E7K’, causes conduction failure presumably because of enhanced cell-surface expression. To more rigorously elucidate the mechanism underlying, we performed voltage-jump experiments in HEK293 cells expressing E7K-TRPM4 channels under the ionomycin-perforated cell-attached recording to mathematically formulate the altered gating of the E7K mutant. The results demonstrated that, compared with the wild-type, a sojourn in the open state is much more stabilized in the E7K mutant with increased voltage-and $Ca^{2+}$-sensitivities. This was consistent with the observations from the power spectrum and single channel analyses.

We next incorporated the obtained gating parameters into the Trovato 2020 model, a most updated human Purkinje fiber single-cell action potential (AP) model, and performed numerical simulations. As compared with the wild-type, the facilitated opening of the E7K mutant strikingly prolonged the AP duration with a depolarizing shift of the resting membrane potential in a manner dependent on the channel density or its maximal activity. Moreover, 1D-cable simulations with the modified Trovato model displayed that increasing the density/activity of E7K (but not of wild-type) TRPM4 channels progressively reduced the AP conduction velocity, eventually culminating in complete conduction block. These results clearly indicate that the pathologically enhanced activity of E7K mutant channel can account for its observed bradyarrhythmogenicity. (COI: NO)

### [OP12-03]
### Study on the expression of Angiotensin converting enzyme 2, ACE2 in the primary culture of human nasal and bronchial epithelial cells

*Kasane Yasuoka[1], Kyoko Matsuda[1], Kotoku Kawaguchi[1], Shinji Asano[1] ([1]*College of Pharmaceutical Sciences, Ritsumeikan University*)

Background

Cell entry of SARS-CoV2 depends on binding of the viral spike (S) proteins to Angiotensinconverting enzyme 2 (ACE2) as the essential host receptor, and on S protein priming by host cell protease, transmembrane serine 2 (TMPRSS2). We studied the expression of ACE2 and TMPRSS2 in Normal Human Bronchial Epithelial cells (NHBE) and ciliated Human Nasal Epithelial Cells (cHNECs). We found their expression changes associated with the differentiation to ciliated cells by Air-Liquid-Interface (ALI), and discussed the possibility of viral infection in the respiratory system.

Methods

NHBE and cHNECs were cultured until monolayer was formed, and the medium of upper chamber was removed to contact with air. The lysates were prepared, and the expressions of ACE2, TMPRSS2 were detected by Western blotting (WB).

Results / Discussion

Cilia were detected in 7 days after starting ALI by immunohistochemistry and increased over 21days. By WB, the expression of ACE2 increased markedly by 7 days and it became constant in high level. Expression of TMPRSS2 was detected before ALI and no big difference in its expression was observed over time. These results are consistent with the fact that SARS-CoV-2 enters through the bronchial and nasal mucosal epithelium.

### [OP12-04]
### $Na^+$ ions slowly permeate through the KcsA $K^+$ channel along a tortuous path

*Takashi Sumikama[1], Kenichiro Mita[2], Shigetoshi Oiki[2] ([1]*Kanazawa University,* [2]*University of Fukui*)

The selectivity of the $K^+$ channels is critical for cellular functions such as maintaining the resting potential. Most textbooks state that its $K^+$selectivity is 10,000-fold over $Na^+$; however, there is currently no evidence from the single-channel data. Here, we measured single-channel $Na^+$ and $K^+$ conductance through the KcsA $K^+$ channel and investigated the permeation mechanism at the atomic level by molecular dynamics simulations. The singlechannel current showed that the conductance ratio ($K^+/Na^+$) was 78.3, which is more than two orders of magnitude smaller than expected. The selectivity evaluated by the simulation was 38.5, reproducing the experimental data. An analysis of the simulation data showed that trajectories of $Na^+$ permeation were closer to the pore wall than $K^+$ trajectories due to the smaller size of $Na^+$, resulting in a tortuous path for $Na^+$, whereas a straight path for $K^+$. Furthermore, we found that the free energy barrier at the entrance for $Na^+$ permeation due to the dehydration penalty is important for rejecting most $Na^+$ from getting into the filter. Thus, the rejection at the filter entrance and the slow permeation in the filter govern the potassium channel’s selectivity.

### [OP12-05]
### Voltage-Sensing Phosphatase (VSP) Promotes Endocytosis-Dependent Nutrient Absorption in Enterocytes

*Adisorn Ratanayotha[1,4], Makoto Matsuda[1], Yukiko Kimura[2], Md. Israil Hossain[1], Shin-ichi Higashijima[2], Takafumi Kawai[1], Michio Ogasawara[3], Yasushi Okamura[1] ([1]*Laboratory of Integrative Physiology, Department of Physiology, Graduate School of Medicine, Osaka University,* [2]*National Institutes of Natural Sciences, Exploratory Research Center on Life and Living Systems, National Institute for Basic Biology,* [3]*Department of Biology, Graduate School of Science, Chiba University,* [4]*Department of Anatomy, Faculty of Medicine Siriraj Hospital, Mahidol University*)

Voltage-sensing phosphatase (VSP) is a membrane protein that translates membrane electrical activities to intracellular phosphoinositide signalings. VSP orthologs from various species have been intensively studied in vitro for their biophysical properties. However, their physiological role in native cells remains largely unknown. Here we report that zebrafish VSP (Dr-VSP) is functionally expressed in lysosome-rich enterocytes (LREs) that mediate dietary protein absorption. Dr-VSP is mainly localized on intracellular vesicles of LREs. Loss of Dr-VSP remarkably reduces initial endosomal vesicles, potentially leading to nutrient malabsorption and higher mortality in zebrafish larvae. Our comparative study on a marine invertebrate Ciona intestinalis VSP (Ci-VSP) also revealed a homologous expression in absorptive epithelial cells of the Ciona digestive tract, corresponding to zebrafish LREs. These findings signify a crucial role of VSP in promoting endocytosisdependent nutrient absorption in enterocytes.

### Oral Presentation 13

### [OP13]
### Blood, Lymph, Immunity, Respiration, Urinary organ, Renal function, Urination

March 18(Fri), 8:30 - 9:30, Room J

### [OP13-01]
### Differential regulation of coagulation and fibrinolysis through thrombomodulin domain- and concentration-dependent activation of thrombin-activatable fibrinolysis inhibitor

*Liina Mochizuki[1,2], Hideto Sano[1], Naoki Honkura[1], Kazuma Masumoto[2], Tetsumei Urano[1,3], Yuko Suzuki[1] ([1]*Department of Medical Physiology, Hamamatsu University School of Medicine*, [2]*Department of Dentistry and Oral and Maxillofacial Surgery, Hamamatsu University School of Medicine*, [3]*Shizuoka Graduate University of Public Health*)

Vascular endothelial cells-expressed thrombomodulin (TM) functions as a dual modulator .via anticoagulant and antifibrinolytic activities.by activating protein C and thrombinactivatable fibrinolysis inhibitor (TAFI). The regulatory mechanisms of these two physiologically important phenomena remain to be fully elucidated. We analyzed the effects of domain-deletion variants of TM on coagulation and fibrinolysis at different concentrations in two methods: turbidimetric assay of platelet-containing plasma clot formation and lysis, and direct visualization of the activated platelet-enhanced dense fibrin network formation and plasminogen accumulation (as a marker of TAFI activity) using confocal microscopy. Variants containing E3456 (minimum domains to activate protein C and TAFI) showed antifibrinolytic activity through TAFI activation at low concentrations; however, those were limited in high concentrations. Variants containing E456 (domains to activate protein C only) delayed fibrin formation at a higher concentration than those required to activate TAFI through the E3456 domain. Such reduction in coagulant activity also reduced TAFI activation. These findings suggest that the balance between the anticoagulant and antifibrinolytic activities of TM is controlled in the domain- and concentration-dependent manners.

### [OP13-02]
### Functional analysis of TRPV4 in human monocytes and macrophages

*Yukiko Atsumi[1,2], Manami Toriyama[1,2,3], Hiroko Kato[1,2], Fumitaka Fujita[1,2,5,7], Fumihiro Okada[1,7], Makoto Tominaga[4,5,6], Ken J. Ishii[2,9,8] ([1]*Graduate School of Pharmacological Sciences, Osaka University*, [2]*National Institutes of Biomedical Innovation, Health and Nutrition*, [3]*Graduate School of Science and Technology, Nara Institute of Science and Technology*, [4]*National Institute for Physiological Sciences, National Institutes of Natural Sciences*, [5]*Exploratory Research Center on Life and Living Systems, National Institutes of Natural Sciences*, [6]*Department of Physiological Sciences, SOKENDAI*, [7]*Mandom Corporation*, [8]*WPI Immunology Frontier Research Center (IFReC), Osaka University*, [9]*The Institute of Medical Science, The University of Tokyo*)

Immune cells, including monocytes and macrophages, protect our body from infections. Even though its necessity, abnormalities in immunity cause various inflammatory diseases such as atopic dermatitis and psoriasis in skin. It is widely accepted that macrophages are referred to as classically activated M1 macrophages and alternatively activated M2 macrophages, however, the mechanism of how macrophages sense changes in the surrounding environment and change their functions remains unclear. Transient Receptor Potential (TRP) channels are non-selective cation channels responding not only to chemical stimuli but also to physical stimuli such as temperature, mechanical stimuli, and potential change. However, the function of TRPV4 in human macrophages remains unclear. Here, we found that TRPV4 activation decreased inflammatory cytokine expression in both human macrophages and monocytes, while selective antagonist canceled this effect. When monocytes were differentiated into M1/ M2 macrophages with TRPV4 agonist, M1 differentiation, but not M2 differentiation was inhibited. Furthermore, in atopic dermatitis dermis, M1 macrophages tended to increase compared to healthy skin. In addition, this tendency was shown in macrophages without TRPV4 particularly. Our results suggest that TRPV4 regulates the balance of macrophage differentiation, raising the potential of TRPV4 as anti-inflammatory target.

### [OP13-03]
### Roles of Ezrin in regulation of ciliary beating in airway ciliary cell

*Kotoku Kawaguchi[1], Daichi Saito[1], Kasane Yasuoka[1], Takashi Nakahari[2], Shinji Asano[1] ([1]*Col. Pharm. Sci., Ritsumeikan Univ.*, [2]*Res. Org. Sci. Tech., Ritsumeikan Univ.*)

The mucociliary clearance is the first line of defense mechanism in the airway epithelium. The beating cilia are the key apparatus to conduct the mucociliary clearance. Ezrin, a crosslinker between membrane proteins and actin cytoskeleton, is located in the basal bodies and microvilli in airway ciliary cells. It is also likely that ezrin may play the important role of apical localization of $\beta_2$-adrenergic receptor ($\beta_2$AR) in airway ciliary cells. Here we studied the physiological roles of ezrin by using the trachea and airway epithelial cells prepared from the ezrin-knockdown ($Vil2^{kd/kd}$) mice. The trachea and airway epithelial cells of $Vil2^{kd/kd}$ mice represented normal morphology and basal body orientation with scanning and transmission electron microscopy, suggesting that ezrin is not directly involved in development and planer cell polarity of cilia. We observed that procaterol stimulates ciliary beating (frequency and amplitude) *via* $\beta_2$AR in the airway ciliary cells using the video microscopy equipped with high-speed camera. In the $Vil2^{kd/kd}$ mice, airway ciliary beating stimulated with procaterol was partly inhibited due to the impairment of cell surface expression of $\beta_2$AR. These results suggest that ezrin regulates the beating of airway ciliary cells by promoting the apical surface localization of $\beta_2$AR.

### [OP13-04]
### Roles of PTHrP in stabilising bladder micromotions and associated afferent nerve activity in rats

*Ayu Sugiura[1], Retsu Mitsui[1], Hikaru Hashitani[1] ([1]*Department of Cell Physiology, Graduate School of Medical Sciences, Nagoya City University*)

Parathyroid hormone-related peptide (PTHrP) is known as an endogenous detrusor smooth muscle (DSM) relaxant. Here, effects of exogenous PTHrP on bladder micromotions-induced afferent nerve activity were investigated. In anesthetised rats, changes in the intravesical pressure were measure during bladder filling, while afferent nerve activity was recorded from centrally-disconnected pelvic nerves. Spontaneous and nerve-evoked contractions of DSM strips were isometrically recorded. The distribution of PTHrP receptors (PTHrPRs) was also examined by fluorescence immunostaining. In the bladders in which contralateral pelvic nerve was centrally-disconnected, intravenously administered PTHrP suppressed nifedipine-sensitive micromotions and associated afferent nerve activity. In the bladders with centrally-connected contralateral pelvic nerve, in which atropine-sensitive larger micromotions were developed, PTHrP diminished the micromotions-induced afferent nerve activity. Bath-applied PTHrP (10 nM) suppressed spontaneous contractions, while less affecting nerve-evoked contractions. PTHrPR was expressed in DSM and nerve cell bodies in pelvic ganglia. In conclusion, PTHrP is capable of suppressing micromotions-induced afferent nerve activity to facilitate the urinary accommodation.

### [OP13-05]
### Enhanced filtration barrier function via renal podocyte TRPC6 activation by mechanical and receptor stimulations

*Jun Ichikawa[1], Midori Nakagawa[2], Ryuji Inoue[2] ([1]*Sano Nihon Univ. Coll.*, [2]*Dept. Physiol., Fukuoka Univ. Sch. Med.*)

A canonical subfamily member of the transient receptor potential protein TRPC6 is abundantly expressed in podocytes which form glomerular slit diaphragm, and its activity is susceptible to both receptor and mechanical stimulations. However, how this property affects the glomerular filtration remains poorly understood. To address this point, we performed the albumin-permeation assay using cell culture-insert membranes on which mouse podocytes stably expressing either wild-type (wt) TRPC6 or its mutant associated with focal segmental glomerulosclerosis (FSGS), M131T were grown. Compared to no stimulation or angiotensin II (Ang II) alone, stimulation by Ang II together with a membraneexpanding agent 2,4,6-trinitrophenol (TNP) reduced the leak of FITC-labelled albumin across the insert membranes in both wt-TRPC6- and M131T-expressing podocytes. Simultaneous application of a TRPC6-specific inhibitor SAR7334 counteracted this effect, which was more prominent in M131T. These results suggest that simultaneous receptor and mechanical stimulations may reinforce the filtration barrier function via podocyte TRPC6 activation, and this may be impaired by the FSGS-associated mutation M131T. COI:NO

### Oral Presentation 14

### [OP14]
### Circulation

March 18(Fri), 9:45 - 10:45, Room I

### [OP14-01]
### A non-neuronal cardiac cholinergic system is downregulated during the diabetes progression but its activation attenuates diabetesinduced cardiac dysfunction via sustaining cardiac homeostasis

*Yoshihiko Kakinuma[1], Eng Saw[2], Yuko Kai[1], Shino Oikawa[1], Martin Fronius[2], Rajesh Katare[2] ([1]*Department of Bioregulatory Science, Nippon Medical School,* [2]*Department of Physiology, School of Biomedical Sciences, University of Otago*)

Previously we advocated a non-neuronal cardiac cholinergic system (NNCCS), addressing cardiomyocytes can synthesize ACh independently of the parasympathetic nervous system and identified that NNCCS is critical for cardiac homeostasis, e.g., NNCCS indispensable for cardiac glucose utilization and accelerating its angiogenesis, shown by a model of NNCCS gain (heart specific ChAT tg mice) or loss of function. However, it remains to be unknown whether NNCCS influences diabetic status and the complication. Then, we crossed db/db mice with heart specific ChAT tg mice to obtain db/db ChAT tg mice, which overexpressed ChAT gene in the heart, with homogeneously lost leptin receptors. db/db mice decreased protein expression of NNCCS components, i.e., ChAT, M2 receptor, AChE, and ACh content as well as Glut4 and glucose content in the heart, and showed cardiac dysfunction during the diabetic progression and body weight gain, suggesting that NNCCS is disturbed in diabetic status. In contrast, db/db ChAT tg mice sustained protein expression of M2 receptors, HIF-1a, GLUT4, pAkt/Akt to wild type mice levels and upregulated cardiac glucose contents, VEGF levels as well as cardiac function. Furthermore, coronary artery branches, which were decreased in db/db mice, were sustained well in db/db ChAT tg mice revealed by coronary angiography and capillary density measurement. On the other hand, cardiac fibrosis levels, aggravated in db/db mice, were attenuated in db/db ChAT tg mice. These results suggest that NNCCS is indispensable for sustaining cardiac homeostasis even in a diabetic condition.

### [OP14-02]
### Cardiac ion channel remodelling associated with miRNA upregulation underlies exercise-induced bradyarrhythmias

*Shu Nakao[1,2], Teruhisa Kawamura[1], Halina Dobrzynski[2], Mark Boyett[2], Alicia D'Souza[2] ([1]*Ritsumeikan Univ.,* [2]*Univ. of Manchester*)

Veteran endurance athletes are prone to bradyarrhythmias including sinus bradycardia and atrioventricular (AV) block, and have a high incidence of pacemaker implantation. Our recent work examined the underlying mechanism of resting bradycardia in human athletes and trained mice. The study demonstrates that the resting bradycardia by endurance exercise is due to ion channel remodelling associated with alterations in microRNAs (miRs) and transcription factors in the sinus node (SN), the primary pacemaking site in the heart. Transcriptome analysis in trained SN suggest metabolic remodelling as a causal factor. Our follow-up study using racehorses and trained mice as animal models of human athletes further explained that exercise-induced AV block also resulted from ion channel remodelling in the AV node. Moreover, miR profiling found 31 miRs significantly upregulated by exercise in AV node. We identified that miR-211-3p and miR-432 targeted CaV1.2 and HCN4 channels, key pacemaking ion channels. In addition, antimiRs against these miRs reversed exercise-induced SN and AV node dysfunction. Altogether, our data provide new insight into underlying mechanisms of exercise-induced bradyarrhythmias and a potential anti-miRNA therapy for cardiac arrhythmias.
(COI: NO)

### [OP14-03]
### Donepezil Markedly Suppresses the Progression of Chronic Heart Failure and Improves the Prognosis in Spontaneously Hypertensive Rats with Myocardial Infarction

*Meihua Li[1], Can Zheng[1], Toru Kawada[1], Masaru Sugimachi[1], Masaru Sugimachi[1] ([1]*Department of Cardiovascular Dynamics, National Cerebral and Cardiovascular Center*)

We have demonstrated that acetylcholinesterase inhibition by donepezil improves the longterm survival of chronic heart failure (CHF) rats with myocardial infarction (MI). This study investigated whether donepezil is applicable to the treatment of CHF complicated with hypertension. CHF was induced by permanent MI in spontaneously hypertensive rats. Survived animals were randomly assigned to the untreated (UT) or donepezil treated (DT, 5mg/kg/day) group. We evaluated hemodynamics, neurohumoral factors, immunohistochemistry, morphology, and survival rate after 7 weeks. Compared with UT, DT significantly decreased the heart rate. DT also improved the 50-day survival rate (76% vs. 43%, P = 0.006), by suppressing the progression of cardiac hypertrophy and cardiac dysfunction. DT not only decreased plasma levels of norepinephrine, BNP, and AVP but also improved systemic inflammation. Donepezil treatment prevented the progression of cardiac remodeling, cardiac dysfunction and improved the prognosis of CHF in spontaneously hypertensive rats with MI, suggesting that donepezil may be used as a new pharmacotherapy for patients with CHF complicated with hypertension.

### [OP14-04]
### Determining the effect of orthostatic stress on cerebrovascular $CO_2$ reactivity: does the methodological approach matter?

*Shigehiko Ogoh[1], Hironori Watanabe[1], Shotaro Saito[1], Erika Iwamoto[2] ([1]*Toyo University,* [2]*Sapporo Medical University*)

Cerebrovascular carbon dioxide ($CO_2$) reactivity (CVR) has prognostic utility and is widely used to identify the effect of the physiological perturbation on cerebrovascular function. Several methodological approaches are commonly used to assess CVR. The aim of the present study was to determine whether the effect of physiological stimulation on CVR is dependent on the method used to quantify CVR. In twenty-one healthy participants (23 ± 3 years), the effect of orthostatic stress on CVR was assessed using both ramp and step inhalation $CO_2$, along with transfer function analysis (TFA) between spontaneous changes in middle cerebral artery mean blood velocity (MCA $V_{mean}$) and predicted partial pressure of arterial-$CO_2$ ($PaCO_2$). CVR determined from a ramp-change in $CO_2$ decreased from supine to 50° HUT ($P$=0.037). Similarly, CVR determined from a step-change in $CO_2$ decreased from supine to 50° HUT ($P$=0.001). In contrast, TFA gain (dynamic magnitude response of CVR) was not different ($P$=0.702). These findings suggest that as well as that CVR attenuated during orthostatic stress, the magnitude and time responses of CVR or dynamic response of CVR-determined by a spontaneous change in $PaCO_2$ and $CO_2$ inhalation displayed a similar trend of change during orthostatic stress. However, individual change in the time response of CVR was not matched with that of the magnitude response of CVR, and also individual dynamic CVR at the operating point (TFA phase-determined by spontaneous $PaCO_2$ change) did not represent that of dynamic CVR determined by $CO_2$ inhalation (the time response of CVR). In summary, these findings highlight the importance of considering the specific CVR property a given analytical approach assesses and the associated methodological limitations, when quantifying the CVR response to physiological stress such as orthostasis.
(COI: NO)

### Oral Presentation 15

### [OP15]
### Autonomic nervous system

March 18(Fri), 9:45 - 10:45, Room J

### [OP15-01]
### Fos expression in lateral hypothalamic neurons projecting to the mesencephalic locomotor region of rats following voluntary wheel running

*Emi Narai[1], Tatsuo Watanabe[2], Satoshi Koba[2] ([1]*Graduate school, Tottori University,* [2]*Tottori University* )

Central circuitry mechanisms underlying autonomic adjustments to exercise are not fully understood whereas orexinergic neurons in the lateral hypothalamus (LH) reportedly mediate locomotion and sympathoexcitation. This study aimed to identify efferent outputs from LH neurons that exhibit excitabilities in response to voluntary exercise. In Sprague Dawley rats, we first studied a neural population postsynaptic to LH neurons via LH injection with a Cre-encoding adeno-associated virus (AAV) vector serotype 1 for anterograde transsynaptic tagging. Significant Cre immunoreactivities were found in a midbrain area called mesencephalic locomotor region (MLR). Moreover, confocal imaging showed close association of LH-derived axons in the MLR labelled by LH injection with an AAV2 vector encoding eYFP with a sympathoexcitatory area rostral ventrolateral medulla (RVLM)-projecting MLR neurons retrogradely labelled by RVLM injection of cholera toxin subunit B; these suggest synaptic connections of MLR-projecting LH neurons (LH→MLR neurons) with RVLM-projecting MLR neurons. Next, to determine if LH→MLR neurons become excited in association with exercise, we investigated the effect of voluntary locomotion on expression of a neuronal activation marker Fos in LH→MLR neurons labelled by MLR injection with a Cre-encoding, retrograde AAV vector. In rats allowed free access to a running wheel for 90 min, increased Fos immunoreactivities were found in LH→MLR orexin-positive neurons, as compared to control rats. We hypothesize that excited orexinergic LH→MLR neurons may mediate sympathetic cardiovascular responses during locomotor exercise. (COI: No)

### [OP15-02]
### Intravenous injection of GLP-1 induces adrenalin secretion and thermogenesis via sensory.central.sympathoadrenal reflex in rats

*Yusaku Iwasaki[1], Kazuyo Ozawa[2], Mamoru Tanida[3], Toshihiko Yada[4] ([1]*Kyoto Prefectural University,* [2]*Jichi Medical University,* [3]*Kanazawa Medical University,* [4]*Kansai Electric Power Medical Research Institute*)

Meal-evoked glucagon-like peptide-1 (GLP-1) release is implicated in postprandial glucose disposal and satiety. On the other hand, it remains unclear whether postprandial GLP-1 release is involved in diet-induced heat production. Here we examined the mechanisms underlying GLP-1-dependent regulation of thermogenesis and energy expenditure. Intravenous injection of GLP-1 activated the neural activities of sympathetic nerve innervating the adrenal gland and increased adrenaline secretion, $O_2$ consumption ($VO_2$) and rectal temperature (RT) in anesthetized rats. The effects of GLP-1 on adrenalin, thermogenesis and $VO_2$ were inhibited by systemic pretreatment with capsaicin that selectively degenerates sensory nerves including vagal afferents. GLP-1-induced adrenaline secretion was inhibited by cholinergic blockers and neurectomy of adrenal sympathetic nerves. The GLP-1-induced increases in $VO_2$ and RT were markedly attenuated by beta-adrenergic blockade. There results indicate that exogenous GLP-1 increases energy expenditure and heat production by releasing adrenaline via a sensory afferent-adrenal axis.

### [OP15-03]
### Regulation of feeding and glucose metabolism by gastrointestinal distension, and involvement of intestinal hormone GLP-1

*Kento Ohbayashi[1], Toshihiko Yada[2], Yusaku Iwasaki[1] ([1]*Anim Sci, Grad Sch Life Env Sci, Kyoto Pref Univ,* [2]*. Div Integr Physiol, Kansai Electric Power Med Res Ins*)

Gastrointestinal distension is known as a signal that promotes meal termination. Reportedly, taking low-calorie and high-volume foods before a meal is not only suppressing feeding but also postprandial rise in blood glucose. However, the underlying mechanisms remain poorly understood. We examined how gastrointestinal distension regulates energy homeostasis, using the pectin-containing carbonated solution (ISF; inflating stomach formulation) which forms stable gel bubbles under acidic condition in the stomach. Single intragastric injection of ISF temporarily expanded the stomach and proximal intestine, elevated plasma GLP-1 concentration, and activated vagal afferents. ISF suppressed feeding and improved glucose tolerance via enhancing insulin sensitivity. These effects partially or completely blunted by treatment with GLP-1 receptor antagonist or denervation of capsaicin-sensitive sensory nerves. Subchronic administration of ISF ameliorated hyperphagic obesity in diet-induced obese mice. There results indicate that gastrointestinal distension enhances GLP-1 release and activates vagal afferents, thereby preventing/ameliorating hyperphagic obesity and improving glucose tolerance.

### Oral Presentation 16

### [OP16]
### Pathophysiology

March 18(Fri), 10:45 - 11:45, Room D

### [OP16-01]
**The study of cerebrospinal fluid dynamics revealed by thoracic spinal MR images.**
**-Dural Sac Shrinkage Signs(DSSS) are the useful findings for the diagnosis of headache and we modified Monro-Kellie doctrine -**

*Takashi Kawahara[1], Masamichi Atsuchi[1], Kazunori Arita[2], Koji Yoshimoto[2] ([1]*Atsuchi Neurosurgical hospital*, [2]*Department of Neurosurgery, Graduate School of Medical and Dental Sciences, Kagoshima University*)

**(Introduction)** Spontaneous intracranial hypotension(SIH) and over-drainage(OD) after Lumboperitoneal shunt(LPS) are the condition of subnormal intracranial pressure. Patients with these disorders present with an orthostatic headache, neck pain, dizziness, tinnitus and visual disturbances. It sometimes leads to altered consciousness. The characteristic findings of SIH and OD after LPS on cranial magnetic resonance imaging (MRI) include dural enhancement, chronic subdural hematoma, subdural fluid collection, and downward displacement of the brain. However, these signs are occasionally obscure or negative. We here report the characteristic signs of SIH and OD after LPS that are found on thoracic spine MRI, are easy to identify. **(Methods)** This retrospective study included 27 consecutive patients with symptoms of SIH. **(Results)** The Dural Sac Shrinkage Signs were frequently observed on sagittal T2-WI at the thoracic level of patients with SIH. Anterior shift of the spinal cord was observed in 26 patients (96.3%), and anterior shift of the posterior dura mater was observed in 22 patients (81.5%). In 21 patients (77.8%), the DSSS were accompanied by CSF collection in the posterior epidural space. A dilated epidural venous plexus was also observed in 21 patients (77.8%). **(Discussion)** The shrinkage of the dural sac is compensated by the enlargement of the surrounding venous plexus, since it becomes an area of lower pressure, as explained likely to intracranial theory by the Monro.Kellie doctrine. Dural sac acts as a buffer in preventing intracranial tissue deformation.

### [OP16-02]
**Cigarette smoke-induced nuclear and mitochondrial DNA damage**

*Mari Ishida[1], Keitaro Ueda[1], Chiemi Sakai[1], Yusuke Kobayashi[2], Yukiko Nakano[2], Masao Yoshizumi[1], Takafumi Ishida[3] ([1]*Department of Cardiovascular Physiology and Medicine, Hiroshima University*, [2]*Department of Cardiovascular Medicine, Hiroshima University*, [3]*Department of Cardiovascular Medicine, Fukushima Medical University*)

[Objective] Smoking is known to be a risk factor for cardiovascular diseases. DNA damage is increased in peripheral mononuclear cells of smokers compared to non-smokers, and the relationship between DNA damage and atherosclerosis has been gaining attention in recent years. In this study, we examined the mechanisms by which smoking habit causes vascular inflammation, focusing on DNA damage and subsequent cellular responses. [Methods and Results] Cigarette smoke extract (CSE) was prepared and added to human umbilical vein endothelial cells. CSE increased nuclear DNA double-strand breaks and oxidative DNA damage in the nucleus and mitochondria (Mito), leading to Mito dysfunction. Mito dysfunction decreased the cytoplasmic protein level of ICAD, an inhibitor of caspaseactivated DNase (CAD), and increased nuclear CAD which fragments nuclear DNA, suggesting that this is one of the mechanisms of nuclear DNA double-strand breaks. DNA fragments were accumulated in the cytoplasm, and real-time PCR analysis revealed that they were not only nuclear- but also Mito-derived DNA. CSE increased the production of cGAMP, a second messenger of the cGAS signaling pathway. The mRNA expression of IL-6, downstream of the cGAS-STING pathway, was increased by CSE, and the increase was suppressed by si-cGAS. This study suggests that sustained exposure to CSE induces nuclear and Mito DNA damage in endothelial cells, leading to DNA accumulation in the cytoplasm and inducing chronic inflammation via the cGAS-STING pathway.

### [OP16-03]
**IL-1 $\beta$ enhances osteoclastgenesis by upregulating the expressions of IGF2 and chemokines in non-osteoclastic cells**

*Yuto Otsuka[1], Yoh Goto[2], Hiromasa Aoki[1], Nagaya Yuko[3], Ken Miyazawa[2], Shigemi Goto[2], Mineyoshi Aoyama[1] ([1]*Department of Pathobiology, Nagoya City University Graduate School of Pharmaceutical Sciences*, [2]*Department of Orthodontics, School of Dentistry, Aichi- Gakuin University*, [3]*Department of Orthopedics, Nagoya City University East Medical Center*)

Bone remodeling mediated by bone-forming osteoblasts (OBs) and bone-resorbing osteoclasts (OCs) is important for maintaining bone structure and function. Excessive activation of OCs leads to bone destruction disease, such as osteoporosis and rheumatoid arthritis (RA). OCs are differentiated from bone marrow cells (BMCs) under the regulation of bone microenvironment. Interleukin-1 $\beta$ (IL-1 $\beta$) is a pro-inflammatory cytokine reported to enhance osteoclstgenesis and play important roles in the bone loss associated RA. In the present study, we investigated the effect of IL-1 $\beta$ on OC formation via microenvironmental cells. Addition of IL-1 $\beta$ to mouse BMCs in the presence of receptor activator of NF-$\kappa$B ligand (RANKL) and macrophage colony-stimulating factor (M-CSF) increased number of total and large OCs. Quantitative RT-PCR revealed that IL-1 $\beta$, IL-1RI and IL-1RII expressed higher in non-OCs than OCs. IL-1 $\beta$ treatment upregulated expression of iNOS, IGF2 and chemokines including SDF-1, CX3CL1 and CXCL7 in non-OCs. These results suggested that IL-1 $\beta$ enhance osteoclastgenesis through increasing the expressions of IGF2 and chemokines in non-OCs.

### [OP16-04]
**Animal model of insomnia with sleep apnea**

*Satoru Masubuchi[1] ([1]*Physiology, Aichi Medical University*)

Obstructive sleep apnea (OSA) patients are exposed to nighttime hypoxia during sleep by intermittent airway closure and feel daytime strong sleepiness. Strangely, insomnia cooccur in some OSA patients, which is called co-morbid insomnia and sleep apnea (COMISA). Here, we show activity responses to daytime hypoxia (DHx) in nocturnal mice were comparable to daytime sleepiness and co-occurring nighttime insomnia in COMISA. DHx reduced activity in active phase and increased following activity in activity ending phase. This down-and-up activity response by DHx was also observed in molecular clock deficient Cry1 and Cry2 double knockout mice (CryDKO) expressing nighttime activity rise under light-dark cycle (LD) and not observed in arrhythmic CryDKO under constant darkness (DD). When daytime timing hypoxia was exposed at transition from LD to DD, ~6 h interval down and up and down wavelike activity responses appeared in arrhythmic CryDKO. We analyzed this COMISA model animal and propose pathophysiology of COMISA.

### Oral Presentation 17

### [OP17]
### Study Methodology, Others

March 18(Fri), 10:45 - 11:45, Room E

### [OP17-01]
**Methodology for estimating the quality of itchiness from repeated patterns of scratching behavior in mice**

*Kotaro Honda[1,2], Mitsutoshi Tominaga[1,2], Kenji Takamori[1,2] ([1]*Institute for Environmental and Gender-Specific Medicine, Graduate School of Medicine, Juntendo University*, [2]*Juntendo Itch Research Center*)

The scratching behavior evoked by itch emotion is assumed to play a role in removing parasites attached to the skin. However, since skin parasites such as mites are smaller than the size that can be recognized by the five senses such as touch and vision, it is unlikely that repeated scratching behavior can eliminate the danger. If so, why do we repeat the scratching behavior? In the present study, we hypothesized that the pattern of the number of repeats of scratching behavior for a duration of itch emotion represents the quality of itchiness. As a result, we found that the proportion of itch episodes in which the number of repeats of scratching behavior was 1, 2, 3, ... was close to $(0.5)^1$, $(0.5)^2$, $(0.5)^3$, ... both in male and female mice with back 2x2 $cm^2$ shaved. This was comparable to a graph of the geometric distribution with the success probability at 0.5. Assuming that it was the geometric distribution, the success probabilities were calculated by maximum likelihood estimation, and they were 0.44 for males and 0.42 for females. When dry skin was induced in these mice, the success probabilities were 0.21 for males and 0.26 for females. The success probabilities of 3-week-old male and female mice were calculated to be 0.49 and 0.50, respectively. These results suggest that itch has a physiological role in risk estimation using the success probability in repeated Bernoulli trials of scratching behavior.

### [OP17-02]
**Analysis of the mechanisms underlying induction of the static offset by acoustic stimuli in cochlear sensory epithelium**

*Takeru Ota[1], Samuel Choi[2], Fumiaki Nin[3], Hiroshi Hibino[1] ([1]*Osaka Univ.*, [2]*Niigata Univ.*, [3]*Gifu Univ.*)

Mammalian hearing is triggered by sound-evoked nanoscale sinusoidal vibrations in the cochlear sensory epithelium. The vibrations deflect hair bundles at the apical surface of hair cells comprising the epithelium. This event results in entry of cation through ion channels on the top of the bundles, depolarizing the cells. Using a unique laser interferometer that we developed, we previously identified that, in the epithelium at cochlear basal turn of live guinea pigs, high-pitch sounds elicit a static offset, which represents the baseline shift of the sinusoidal vibrations. We also showed that the offset depends on voltage-driven motor protein in the hair cells. Theoretically, this motion is likely to protect the hair cells from being injured by loud sounds as well as expand the dynamic range of hearing. To further clarify the mechanisms underlying the offset, we analyzed the hair bundle, actin/myosin-based organelle that can actively modulate the cation entry through the channels. Perturbation of the activity of myosin by inhibition of its phosphorylation or by increase of the intracellular [$Ca^{2+}$] prominently reduced the magnitude of the offset. Therefore, this motion may rely on the bundle' s tensional homeostasis controlled by the myosin actin interaction.

### Oral Presentation 18

### [OP18]
### Embryology, Regenerative Medicine, Development, Growth, Aging

March 18(Fri), 10:45 - 11:45, Room F

### [OP18-01]
### Zebrafish pancreatic β cells regenerate function and morphology in a stepwise manner using Neurod1 expressing cells from different cell lineage

*Hiroki Matsuda[1] ([1]*Ritsumeikan University*)

Pancreatic β cells, which produce Insulin, play a central role for glucose homeostasis. Regenerative capacity of mammalian β cells is limited and that loss of β cells causes diabetes. In contrast, even adult zebrafish has high regenerative capacity of pancreatic islets, including β cells, making them an attractive model for the study of β cell regeneration. However, fundamental questions remain, such as when β cell regeneration is completed and what is the cellular source of regenerating β cells. Here I showed that pancreatic β cell regeneration is complete at 14 days after β cell ablation by two-step regeneration process, first regenerating function and then regenerating morphology. In addition, I found that all regenerating pancreatic β cells arose from Neurod1 expressing cells, which contacted with pancreatic α cells directly. Notably, Neurod1 expressing cells, which has already existed in pancreatic islet, contributed to functional regeneration. New Neurod1 expressing cells for morphological regeneration generated during functional regeneration. Altogether, my results shed light on the basic cellular mechanisms underlying β cell regeneration.

### [OP18-02]
### Spermidine ameliorates sarcopenia through activation of mTORindependent autophagy in mice

*Tomohiro Iwata[1], Takanaga Shirai[2,3], Kazuki Uemichi[3], Riku Tanimura[1], Shunsuke Sugiyama[4], Tohru Takemasa[3] ([1]*Graduate School of Comprehensive Human Sciences, University of Tsukuba,* [2]*Research Fellow of the Japan Society for the Promotion of Science,* [3]*Faculty of Health and Sport Sciences, University of Tsukuba,* [4]*School of Physical Education, Health and Sport Sciences, University of Tsukuba*)

Recent study reported that suppression of mTOR signaling, a major regulatory pathway for cell growth, inversely ameliorates age-related skeletal muscle atrophy (sarcopenia). Autophagy, which is negatively controlled by mTOR, is an important waste disposal mechanism for maintaining the intracellular environment. Therefore, we hypothesized that mTOR inhibition ameliorates sarcopenia through activating autophagy and activation of mTOR-independent autophagy effectively ameliorates sarcopenia compared with mTOR inhibition. In this study, we examined this hypothesis in a rodent model using mTOR inhibitor (rapamycin: RA) or mTOR-independent autophagy activator (spermidine: SP) on aged skeletal muscle in mice. Compared with RA injection, SP injection significantly suppressed the decrease in muscle fiber cross-sectional area during aging. Protein expression analysis confirmed that RA injection inhibits mTOR signaling and activates autophagy, and SP injection activates autophagy independent of mTOR. These results suggest that activation of mTOR-independent autophagy effectively ameliorates sarcopenia compared with mTOR inhibition.

### [OP18-03]
### Sympathetic regulation of skeletal muscle contractile force decreases with muscle atrophy in aged rats

*Harumi Hotta[1], Kaori Iimura[1], Nobuhiro Watanabe[1], Harue Suzuki[1], Kazuhiro Shigemoto[2] ([1]*Department of Autonomic Neuroscience, Tokyo Metropolitan Institute of Gerontology,* [2]*Department of Geriatric Medicine, Tokyo Metropolitan Institute of Gerontology*)

We have recently shown that reflex excitation of the muscle sympathetic nerves triggered by muscle contractions contribute to the maintenance of contractile force of the hindlimb muscles in rats. We hypothesized that this mechanism declines during aging in association with a loss of the muscle mass. In this study, we examined modulatory ability of sympathetic nerves on the muscles contractile force in aged rats of 32-33 months old and compared to that in young rats of 4-9 months old. Tetanic contractions of the triceps surae muscles were induced by electrical stimulation of the tibial nerve. The contractile force was measured before and after cutting or stimulating a lumbar sympathetic trunk (LST). We found that both a decrease and an increase in force by cutting or stimulating the LST, respectively, were markedly attenuated in aged rats with severe loss of muscle mass, while well maintained in other aged rats with mild loss of muscle mass. These results indicate that sympathetic ability to regulate muscle function weakens in association with muscle atrophy. This age-related decline of the feedback system between skeletal muscle and muscle sympathetic nerves would be a factor to accelerate sarcopenia, a degenerative loss of skeletal muscle mass and strength in the elderly.

### Oral Presentation 19

### [OP19]
### Neural network, Sensory function, Sensory organ

March 18(Fri), 10:45 - 11:45, Room G

### [OP19-01]
### Organization of the entorhinal cortex layer V in macaque monkeys based on cell-type-specific marker and connectivity

*Shinya Ohara[1], Rintaro Yoshino[1], Kei Kimura[2], Taichi Kawamura[1], Soshi Tanabe[2], Andi Zheng[2], Shinya Nakamura[1], Ken-ichi Inoue[2], Masahiko Takada[2], Ken-Ichiro Tsutsui[1,3], Menno Witter[1,3,4] (*[1]Laboratory of Systems Neuroscience, Graduate School of Life Sciences, Tohoku University, [2]Systems Neuroscience Section, Department of Neuroscience, Primate Research Institute, Kyoto University, [3]Laboratory of Systems Neuroscience, Graduate School of Medicine, Tohoku University, [4]Department of Developmental Neuroscience, Graduate School of Medicine, Tohoku University*)

The entorhinal cortex (EC), in particular neurons in layer V (LV), is considered to play an important role in transferring hippocampal information to the downstream neocortical networks for long-term memory formation. Recent studies in rodents have shown that EC LV is composed of layers Va (LVa) and Vb (LVb), which exhibit distinct patterns of neuronal connectivity and gene expression. Neurons in LVb, immunopositive for Purkinje cell protein 4 (PCP4), are the main recipient of hippocampal projections, whereas LVa neurons, immunonegative for PCP4, originate the main outputs of EC. In addition, we recently showed that LVb neurons mediate the hippocampal outputs to forebrain structures through projections to LVa, and that these intrinsic connections differ between EC subdivisions. Whether monkey EC shares a similar organization of LV as present in rodents remains unknown. To confirm the organization of monkey EC LV, here we examined the distribution of PCP4-positive neurons and forebrain-projecting neurons across all EC subdivisions in macaque monkeys. We found that LV organization is different between the rostral and caudal EC in monkeys, and that a clear laminar arrangement of LV neurons, which is evident in rodent LEC, is absent in the rostral EC of monkeys. Although further investigations are required, these results suggest that the rostral EC of primates may have developed into a unique information processing system.

### [OP19-02]
### Memory-supporting inter-regional networks among local cellensembles developed in experience dependent manner

*Hiroyuki Miyawaki[1], Kenji Mizuseki[1] (*[1]Osaka City University, Graduate School of Medicine*)

The accumulating evidence indicates that memories are represented as combinations of active neurons, known as cell ensembles. Fear memory related ensemble activities have been reported in various brain regions, however, it is still unclear how the distributed information is integrated to support memory. To clarify this, we performed large-scale electrophysiological recording in the basolateral amygdala (BLA), ventral hippocampus CA1 region (vCA1), and prelimbic region of medial prefrontal cortex (PL) of fear conditioned rats. We revealed that coactivation of BLA-PL ensemble pairs developed rapidly during the conditioning and persisted in the subsequent sleep. Additionally, vCA1- PL ensemble coactivation occurred in rudimentary manner during the conditioning and a subset of the pairs coactivated more prominently during the following sleep. These coactivations accompanied fast network oscillations and reappeared during memory retrieval. Furthermore, ensembles in BLA and PL, but not in vCA1, were more active than chance level even in sleep prior to the conditioning. These findings suggest that elements of memories are captured quickly by pre-configured local networks and newly developed inter-regional network develops to bind distributed information together.

### [OP19-03]
### Hypothalamic orexinergic neurons inversely regulate pain and itch: pain relief and itch exacerbation

*Tatsuroh Kaneko[1], Tomoyuki Kuwaki[1], Hideki Kashiwadani[1] (*[1]Department of Physiology, Graduate School of Medical and Dental Sciences, Kagoshima University*)

Pain and itch are discomfort sensations which are antagonistically regulated, as pain stimulation suppresses itch and inhibition of pain provokes itch. Although the neural mechanisms underlying the interaction between pain and itch have been unveiled at the levels of the spinal cord and the lower brainstem, evidence is lacking regarding the upper central nervous system. In this study, we focused on the orexin (ORX) neurons in the lateral hypothalamus (LH), which mediate various "defense responses" when animals confront stressors. Here, we found that ORX neurons were activated by pruritic stimulation. Furthermore, we found that the scratching behaviors induced by the pruritogen were significantly suppressed in ORX-neuron-ablated (ORX-AB) mice. This suppression was not observed in ORX-peptide-knockout mice, indicating that non-ORX co-transmitter/ modulators expressed in ORX neurons contribute to the neural processing of itch. The exaggerated pain behavior and attenuated itch behavior observed in ORX-AB mice indicated that ORX neurons modulate pain and itch in an opposite way, i.e., pain relief and itch exacerbation. Our results suggest that ORX neuron inversely regulate pain- and itchrelated behaviors.

### [OP19-04]
### Effects of sleep deprivations on nociception threshold and neural activity in the anterior cingulate cortex of mice

*Kosuke Nakano[1,2], Keisuke Koga[1], Manabu Kadoya[2], Hidenori Koyama[2], Hidemasa Furue[1] (*[1]Dept Neurophysiol, Hyogo College of Medicine, [2]Dept Internal Medicine, Hyogo College of Medicine*)

Sleep deprivation (SD) can be both a cause and a consequence of pain. However, it remains unclear how sleep deprivation alters nociceptive responses and neuronal activities in the CNS. We investigated how SDs (duration and quality) lower nociceptive behavioral threshold in mice, and examined whether neuronal activates in the anterior cingulate cortex (ACC) are altered following sleep deprivations. In mice received a SD, nociceptive mechanical threshold was decreased, and after the SD it gradually returned to nearly control level. On the other hand, in mice received a random SD, the nociceptive threshold was also decreased during the deprivation, but it was not recovered. ACC neurons in random SD mice, responded to nociceptive mechanical stimulation with a long-lasting after discharge. When ACC neuronal activity was inhibited with a chemogenetic approach, the nociceptive neuronal responses in the ACC was decreased, and behavioral nociceptive threshold was also increased. These results suggest that nociceptive responses elicited in ACC neurons are increased following random SD, and their excitability may be involved in the induction of sleep deprivation-induced pain.

### [OP19-05]
### Segregation of $Ca^{2+}$ signalings in olfactory signal transduction

*Hiroko Takeuchi[1], Takashi Kurahashi[1] (*[1]Graduate School of Frontier Biosciences, Osaka University*)

Olfactory signal transduction is conducted through a cAMP-mediated second messenger cascade at the cilia of the olfactory receptor cell. The cytoplasmic $Ca^{2+}$ concentration increases through the opening of CNG channels, which then underlies two major functions, namely, olfactory adaptation and signal boosting. Olfactory adaptation is regulated by $Ca^{2+}$ feedback to the CNG channel, whereas signal boosting is achieved by an additional opening of the $Ca^{2+}$-activated $Cl^-$ channel. Thus, the influx of $Ca^{2+}$ and the resultant increase in cytoplasmic $Ca^{2+}$ levels play seemingly opposing effects: increasing the current while reducing the current through adaptation. The two functions could be interpreted to compensate for each other; however, in real cells, both functions should be segregated. $Ca^{2+}$ dynamics in olfactory cilia need to be directly measured, but technical difficulties accompanying the thin structure (100 nm diameter) of olfactory cilia have prevented systematic analyses. In this study, we used a combination of electrophysiology, local photolysis of caged cAMP, and $Ca^{2+}$ imaging on the very restricted local area within the single cilium, and found that free $Ca^{2+}$ in the local ciliary cytoplasm decreased along with a reduction in the current that contains $Ca^{2+}$-activated $Cl^-$ current, whereas $Ca^{2+}$- dependent adaptation persisted for a longer period. Two $Ca^{2+}$ signalings could be clearly segregated by the molecular dynamics.
Authors have no COI to disclose in relation to the presentation.

### Oral Presentation 20

### [OP20]
### Circulation

March 18(Fri), 10:45 - 11:45, Room H

### [OP20-01]
### Paroxetine improves right ventricular-pulmonary artery coupling in a rat model of pulmonary hypertension

*Mark Waddingham[1], Hirotsugu Tsuchimochi[1], Takashi Sonobe[1], James Pearson[1], Takeshi Ogo[1] ([1]*National Cerebral and Cardiovascular Center* )

Right ventricle (RV) failure is an independent predictor of mortality in pulmonary hypertension (PH). Overactivation of the sympathetic nervous system (SNS) and metabolic alterations are central in the evolution of RV failure in PH. G-protein coupled receptor kinase 2 (GRK2) drives SNS overactivation and induces metabolic aberrations in cardiomyocytes in left heart failure. In this study, we hypothesized that chronic GRK2 inhibition with paroxetine would improve RV function in a rat model of PH.
PH was induced in rats using sugen/10% chronic hypoxia (SuHx), followed by 2 weeks of normoxia. PH rats were then randomized to receive paroxetine (5mg/kg/day, s.c.) or vehicle (10% DMSO) and followed for a further 4 weeks. Global RV function was measured in anesthetized rats using cardiac catheterization.
When compared to control rats, PH rats exhibited significantly greater relative RV mass (P<0.0001) and RV systolic pressure (P<0.0001), consistent with the classic PH phenotype. Both were unaffected by paroxetine treatment. PH was also associated with RV systolic dysfunction compared to control rats as demonstrated by reduced stroke volume (P<0.01) and cardiac output (P<0.001), along with elevated RV afterload (Ea, P<0.0001 vs. control). Chronic paroxetine treatment in PH rats significantly increased stroke volume and cardiac output (both P<0.05) and reduced RV afterload (Ea, P<0.01) compared to vehicle-treated PH rats. Moreover, total pulmonary resistance was also significantly reduced after 4 weeks of paroxetine treatment (P<0.05 vs. PH vehicle rats).
Chronic paroxetine treatment significantly improved the RV-pulmonary artery coupling in a rat model of PH. The underlying mechanisms are currently being investigated.

### [OP20-02]
### Selective activation of adrenoceptors potentiates $I_{Ks}$ current in pulmonary vein cardiomyocytes through the protein kinase A and C signaling pathways

*Xinya Mi[1], Wei-Guang Ding[1], Futoshi Toyoda[1], Mariko Omatsu-Kanbe[1], Akiko Kojima[2], Hiroshi Matsuura[1] ([1]*Department of Physiology, Shiga University of Medical Science,* [2]*Department of Anesthesiology, Shiga University of Medical Science*)

Delayed rectifier $K^+$ current ($I_{Ks}$) is a key contributor to repolarization of action potentials. This study investigated the mechanisms underlying the adrenoceptor-induced potentiation of $I_{Ks}$ in pulmonary vein cardiomyocytes (PVC). PVC were isolated from guinea pig pulmonary vein. The action potentials and $I_{Ks}$ current were recorded using perforated and conventional whole-cell patch-clamp techniques. The expression of $I_{Ks}$ was examined using immunocytochemistry and Western blotting. *KCNQ1*, a $I_{Ks}$ pore-forming protein was detected as a signal band approximately 100 kDa in size, and its immunofluorescence signal was found to be mainly localized on the cell membrane. The $I_{Ks}$ current in PVC was markedly enhanced by both $\beta_1$- and $\beta_2$-adrenoceptor stimulation with a negative voltage shift in the current activation, although the potentiation was more effectively induced by $\beta_2$-adrenoceptor stimulation than $\beta_1$-adrenoceptor stimulation. Both $\beta$- adrenoceptor-mediated increases in $I_{Ks}$ were attenuated by treatment with the adenylyl cyclase (AC) inhibitor or protein kinase A (PKA) inhibitor. Furthermore, the $I_{Ks}$ current was increased by $\alpha_1$-adrenoceptor agonist but attenuated by the protein kinase C (PKC) inhibitor. PVC exhibited action potentials in normal Tyrode solution which was slightly reduced by HMR-1556 a selective $I_{Ks}$ blocker. However, HMR-1556 markedly reduced the $\beta$-adrenoceptor-potentiated firing rate. The stimulatory effects of $\beta$- and $\alpha_1$-adrenoceptor on $I_{Ks}$ in PVC are mediated via the PKA and PKC signal pathways. HMR-1556 effectively reduced the firing rate under $\beta$-adrenoceptor activation, suggesting that the functional role of $I_{Ks}$ might increase during sympathetic excitation under *in vivo* conditions.

### [OP20-03]
### The mechanism of mitochondrial dynamics regulation via PPI

*Sho Aki[1], Kazuaki Yoshioka[2], Yoh Takuwa[2], Tsuyoshi Osawa[1] ([1]*Division of Integrative Nutriomics and Oncology RCAST, The University of Tokyo,* [2]*Department of Physiology Kanazawa University School of Medicine*)

The balance between fusion and fission of mitochondria that maintain the morphology and function of mitochondria, but the mechanism for maintaining mitochondrial homeostasis (mitochondrial dynamics) unclear. We found that polyphosphoinositide (PPI) generated by Mitochondrial Fusion Activating Kinases 1 and 2 (MFAK1 / 2) promote mitochondrial fusion. MFAK1 / 2 double knockdown increased excessive mitochondrial division and fragmentation, significantly reduced the function of fragmented mitochondria (membrane potential), and accumulation of reactive oxygen species (ROS) was observed. The expression levels of mitochondrial fusion factor (Mitofusin1 / 2, OPA1) and fission factor (Drp1, Dynamin2, MFF) did not change in MFAK1 / 2 double knockdown cells. In addition, although Mfn was recurited to the mitochondrial fusion site, mitochondrial fusion was inhibited, suggesting that MFAK1 / 2 metabolite PPI is essential for mitochondrial fusion. In addition, cardiomyocyte-specific Mfak1/2 double knockout (KO) mice showed accumulation of fragmented mitochondria in cardiomyocyte and died within 1-2 days after birth due to Abnormal cardiac contraction. These observations indicate that MFAK1 / 2 is a novel kinase that promotes mitochondrial fusion through PPI production and controls mitochondrial dynamics.

### [OP20-04]
### Pre-administration of a carboxypeptidase inhibitor enhances tPAinduced thrombolysis in mouse microthrombi: evidence from real-time intravital imaging analysis

*Nitty Skariah Mathews[1], Yuko Suzuki[1], Naoki Honkura[1], Hideto Sano[1], Tetsumei Urano[1] ([1]*Department of Medical Physiology, Hamamatsu University School of Medicine*)

Introduction: Thrombolysis using recombinant tissue-type plasminogen activator (rt-PA) is the pharmacological treatment of choice in acute thrombotic events. However, a narrow therapeutic window and bleeding complications limit its use. We describe the role of carboxypeptidase inhibitor from potato tuber (PTCI), an inhibitor of activated thrombin-activatable fibrinolysis inhibitor (TAFIa), on Glu-plasminogen accumulation and microthrombus dynamics in vivo and demonstrate its influence on rt-PA-mediated thrombolysis. Materials and Methods: In conjunction with real-time intravital two-photon excitation fluorescence microscopy, we produced and imaged laser-induced microthrombi in the mesenteric venules of Green Fluorescent Protein (GFP)-expressing mice. We examined microthrombus dynamics and thrombolysis patterns in vivo by measuring the changes in the fluorescence intensity of labeled Glu-plasminogen following administration of epsilon aminocaproic acid (EACA), PTCI, and rt-PA. Results: PTCI enhanced Glu-plasminogen accumulation at the core of the thrombus by inhibiting TAFIa, while EACA inhibited this process. Exogenous rt-PA effectively triggered Glu-plasminogen activation within the thrombus and promoted thrombolysis. Administration of PTCI and rt-PA together showed no significant benefit on thrombolysis compared to rt-PA administration alone. However, early-phase systemic administration of PTCI before thrombolytic therapy by rt-PA expedited clot lysis as evidenced by significantly faster time to reach peak Glu-plasminogen fluorescence intensity and shorter time to achieve near-complete clot lysis ($P$ = 0.014 and $P$ = 0.003, respectively). Conclusions: PTCI potentiates rt-PA-mediated thrombolysis when administered early in acute thrombotic events. Further studies are warranted to explore the potential of TAFI inhibitors as adjunct agents in thrombolysis or thromboprophylaxis.

### [OP20-05]
### Changes in the transcriptional profile of rat pulmonary veins before and after birth

*Daiki Seya[1], Toru Akaike[1], Akiyasu Iwase[2], Hiroki Kurihara[2], Susumu Minamisawa[1] ([1]*Dept. of Cell Physiology, The Jikei University School of Medicine,* [2]*Dept. of Physiological Chemistry and Metabolism, Graduate School of Medicine, The University of Tokyo*)

The pulmonary circulation system is the main site of blood gas exchange, and the pulmonary veins (PVs) are important low-pressure functional vessels that support the pulmonary circulation. In the perinatal period, when the placental circulation is switched to the pulmonary circulation, the hemodynamics of the PVs changes dramatically. However, little is known about the changes in gene expression in the PVs that are characteristic of this period. Therefore, we examined the transcriptional profiles of each pooled tissues from the proximal- and distal-PVs in late-fetal and neonatal Wistar rats by DNA microarray analysis (SurePrint G3 Rat). We first focused on whether the expression of genes known to be involved in hypoxia-dependent regulation is altered in PVs before and after birth. In proximal- or distal-PVs, 309 probes including Kcnk2, Edn1, and Pgf were upregulated after birth, while 770 probes including Aqp3 and Ddit4 were downregulated. In common with the proximal- and distal-PVs, the expression of genes corresponding to 65 probes was upregulated after birth, and the expression of genes corresponding to 144 probes was downregulated. Pathway analysis showed that genes with upregulated expression were enriched for genes related to regulation of cell growth and the vascular endothelial growth factor receptor signaling pathway, while those with downregulated expression were enriched for genes related to acute-phase response and negative regulation of endopeptidase activity, respectively.

### Oral Presentation 21

### [OP21]
### Higher brain function

March 18(Fri), 11:00 - 12:00, Room I

### [OP21-01]
### Multiple cortical processing streams support response inhibition in humans

*Takahiro Osada[1], Akitoshi Ogawa[1], Akimitsu Suda[1], Koji Nakajima[1,2], Masaki Tanaka[1], Satoshi Oka[1], Koji Kamagata[1], Shigeki Aoki[1], Yasushi Oshima[2], Sakae Tanaka[2], Nobutaka Hattori[1], Seiki Konishi[1] ([1]*Juntendo University*, [2]*The University of Tokyo*)

Response inhibition supports adaptive behavior by suppressing inappropriate behavior. Multiple areas are involved in response inhibition. However, it is poorly understood how the processing streams among the areas are implemented to achieve response inhibition. In this study, we first identified essential areas for response inhibition during a stop-signal task via fMRI. Next, single-pulse transcranial magnetic stimulation (spTMS) was administered during the task. Each of the areas was stimulated in half of the trials, and trials with and without stimulation were intermixed within runs. Prolonged stop-signal reaction time (SSRT) was observed in spTMS to the ventral posterior inferior frontal cortex (vpIFC) at the time window of -90 to -60 ms, with the end of the SSRT for no-stimulation trials defied as zero. The dorsal posterior inferior frontal (dpIFC), presupplementary motor area (preSMA), and intraparietal sulcus area (IPS) showed a transient disruption effect at the subsequent time windows: -60 to -30 ms for dPIFC and preSMA, and -30 to 0 ms for IPS. Finally, we tested whether the information flowed effectively from the vpIFC to the preSMA and from the vpIFC to the dpIFC. SpTMS following suppression of the vpIFC with repetitive TMS showed that prolonged SSRT was still observed by spTMS to the dpIFC, but not to the preSMA. These results suggest two parallel processing streams that act concurrently during response inhibition (vpIFC-preSMA vs. dpIFC-IPS).

### [OP21-02]
### Low-frequency rTMS targeting ventral medial frontal cortex leads to depression-like behavioral and physiological states in monkeys

*Shinya Nakamura[1], Ken-Ichiro Tsutsui[1] ([1]*Laboratory of Systems Neuroscience, Graduate School of Life Sciences, Tohoku University*)

The medial frontal cortex (MFC), especially its ventral part, has been implicated in controlling negative emotion and mood as well as in autonomic functions. Here, we aimed to causally examine how the inhibition of the ventral MFC (vMFC) affects behavioral and physiological states of monkeys by using low-frequency repetitive transcranial magnetic stimulation (LF-rTMS). Following the LF-rTMS targeting the vMFC, we observed a striking change in the spontaneous behavior of monkeys in their home cage during daytime. We found a significant decrease in the duration showing active behaviors, e.g., hanging on the cage and walking around, and a significant increase in the duration showing inactive behaviors, e.g., sitting still with a hunched posture and lying down. Such a shift to an inactive state by the vMFC stimulation was also confirmed by an automated measurement of the movement activity level by an accelerometer. We also found a significant increase in an evening plasma cortisol level. On the other hand, such drastic changes were not observed by the LF-rTMS targeting the dorsal or posterior MFC or sham stimulation. Furthermore, it is a noteworthy that the administration of antidepressant, ketamine, ameliorated the abnormal behavioral and physiological states induced by the vMFC stimulation. Taken together, our findings suggest that the LF-rTMS targeting the vMFC leads to depression-like behavioral and physiological states in monkeys.

### [OP21-03]
### Effect of spirituality, exercise, music and engagement with nature on intelligence, stress and cognition

*Mohita Singh[1] ([1]*GMC Jammu*)

BACKGROUND AND AIMS Spiritual beliefs include the relationship to a superior being and recognition of a sense that there is something greater than myself. Physical exercise is any bodily activity that enhances or maintains physical fitness and overall health and wellness. Music is a universal language to express mind through tone and emotions as it helps to express feelings. Music communicates the message. Nature is the natural, physical world or the universe. Intelligence is an abstract ability which is easily identifiable and recognizable but interestingly very hard to define. Emotional intelligence is the ability to understand one's own emotions and emotions of others and use emotional information to guide thinking and behaviour. Adversity quotient deals with ability of a person to deal with adversities in life. An individual encounters stress when put under situations surpassing his abilities to deal with it. Cognition refers to mental process of acquiring knowledge and understanding through thought, experience and senses. The present study was designed to study the effect of different interventions on IQ, EQ, RS, stress parameters and cognitive functions. MATERIAL AND METHOD The present study was performed on 150 subjects in age group of 30-39 years. Their basal level of IQ, EQ, RS, ASS, PSS, IHG and stroop tests was recorded. Based on median split method, they were divided into high and low groups for each parameter. The 144 subjects were divided into 4 groups of 36 each. Each group included equal number of subjects from high and low groups for every parameter on random bases. Groups were allocated four different interventions of spirituality practice, moderate intensity physical exercise, receptive music and engagement with nature for one month. Statistical analysis Paired t test was used for analysing baseline and post intervention effect for every parameter. Results between groups for parameters was analysed via Kruskal wallis test. The results were computed as significant at $p < 0.05$ level, more significant at $p < 0.01$ level and highly significant at $p < 0.001$ level. RESULT Statistically significant and non significant results were obtained between parameters at baseline and post intervention for every parameter and also on multiple comparison of each parameter in one groups with other three groups at different level of significance. IQ exhibited maximum increment post spirituality; EQ with music intervention, resilience score and stress parameters with spirituality, acute stress with exercise and different cognitive functions subtests with different intervention. CONCLUSION Though every intervention had different and positive effects on parameters, but overall spiritual practices had most impact.

### [OP21-04]
### Task-dependent neural representation of behavioral factors in primate medial prefrontal cortex

*Muhammad Ali Haider Awan[1], Hajime Mushiake[1], Yoshiya Matsuzaka[2] ([1]*Tohoku University*, [2]*Tohoku Medical and Pharmaceutical University*)

Higher mammals can learn and perform diverse tasks governed by different rules. We studied the neural mechanism underlying such flexible switching of cognitive behavior. Our specific questions were as follows: Do neurons playing a particular role (e.g. working memory) under a particular behavioral condition change their functions across different conditions? Or, alternatively, are different sets of neurons recruited for a particular function across different conditions?
To address these questions, we recorded neuronal activity in the medial frontal cortex of a primate during the performance of a two choice arm reaching task. The task required the monkey to reach to and press either a left or a right push button. The target button was determined under two conditions.
Prior to response initiation, neurons in the posterior medial prefrontal cortex flexibly switched representation of the task-relevant information between the conditions, indicating the same neuronal population reconfigured their function per task requirement. Our finding indicates that neurons in this area play multifaceted roles across various behavioral context.

### Oral Presentation 22

### [OP22]
### Autonomic nervous system

March 18(Fri), 11:00 - 12:00, Room J

### [OP22-01]
**Studies on the relationship between olfaction and cognitive function in the elderly**

*Sae Uchida[1], Fusako Kagitani[1] ([1] *Tokyo Metropolitan Institute of Gerontology*)

The cholinergic neurons originating in the basal forebrain send projections to the neocortex, hippocampus, and olfactory bulb that contribute to cognition (especially attention), memory, and olfactory function, respectively. This study investigates the relationship between olfaction and cognitive function in the elderly. In elderly people living in the community (n=12, 70-90 years, doing voluntary work), the olfaction [identification threshold for rose odor] and cognitive function [general cognitive ability assessed by Mini- Mental State Examination (MMSE), its sub-domains, and attentional ability assessed by Trail Making Test -part A; TMT-A] were assessed. Subjects with a higher olfactory threshold (≧5) declined more in the performance speed of TMT-A (73% ± 7%, p = 0.05) compared with those subjects with a lower threshold (≦4) (averaged value was set at 100%). Other cognitive statuses assessed by MMSE tended to decline in subjects with higher thresholds. Our preliminary study suggests that olfactory impairment links to the decline in cognitive function, particularly of attention-relating cholinergic function.

### [OP22-02]
**Imaging neural activity in nucleus tractus solitarii of live mice as an afferent target of vagus nerve by wide field and cellular resolution two photon microscopy in the brainstem**

*Masakazu Agetsuma[1,2], Daisuke Yamada[3,4], Hiroshi Kuniishi[3], Eri Takeuchi[3], Tomoko Kobayashi[1], Ryo Fujikawa[4], Fumiko Kito[4], Akiyoshi Saitoh[4], Junichi Nabekura[1], Masayuki Sekiguchi[3] ([1] *National Institute for Physiological Sciences,* [2] *Medical Institute of Bioregulation, Kyushu University,* [3] *National Center of Neurology and Psychiatry,* [4] *Faculty of Pharmaceutical Sciences, Tokyo University of Science*)

The brain and internal organs communicate with each other, and recent studies has suggested the importance in regulating mental health. One of the main systems for this bidirectional communication is the vagus nerve, which contains both efferent and afferent fibers. The artificial vagus nerve stimulation (VNS) has been clinically utilized in the treatment of various psychiatric diseases including epilepsy and depression, indicating the importance of the afferent signal transmission from the internal organs to the brain trough the vagus nerve. However, the underlying mechanism remains unclear. Although the nucleus tractus solitarii (NTS) is proposed to be a brainstem structure receiving the direct signal from vagus nerve afferent and relaying it to the whole brain emotional regulatory networks, direct evidence is limited due to the anatomical complexity and difficulty in accessing to the deep-brain NTS region of living animals. To address this point, here we developed a wide-field and deep-brain two photon imaging method, enabling us to identify cellular-resolution neural activities of the NTS and surrounding regions in live mice. In the presentation, we will report the evidence demonstrating that the NTS neurons receive the signal from the vagus afferent fibers.

### [OP22-03]
**Early phase of pupil dilation is mediated by the peripheral parasympathetic pathway.**

*Chinatsu Marumo[1], Tamami Nakano[2] ([1] *Faculty of Medicine, Osaka Univ.,* [2] *Graduate School of Frontier Bioscience, Osaka Univ.*)

Pupil diameter fluctuates in association with changes in brain states induced by the neuromodulator systems. However, it remains unclear how the neuromodulator systems control the activity of the iris sphincter (constrictor) and dilator muscles to change the pupil size. The present study compared temporal patterns of pupil dilation during movement when each muscle was pharmacologically manipulated in the human eye. When the iris sphincter muscle was blocked by tropicamide, the latency of pupil dilation was delayed and the magnitude of pupil dilation was reduced during movement. In contrast, when the iris dilator muscle was continuously stimulated by phenylephrine, the latency and magnitude of rapid pupil dilation did not differ from the untreated control eye, but sustained pupil dilation was reduced until the end of movement. These results suggest that the iris sphincter muscle, which is under the control of the parasympathetic pathway, is quickly modulated by the neuromodulator system and plays a major role in rapid pupil dilation. However, the iris dilator muscle receives signals from the neuromodulator system with a slow latency and is involved in maintaining sustained pupil dilation.

### Poster Presentation

### Day 1
(March 16, 12:30～14:30)

[1P01] Ion channels, Receptors, Membrane transport
[1P02] Ion channels, Receptors
[1P03] Molecular physiology, Cell biology, Others
[1P04] Neural network
[1P05] Neural network, Neurons, Synapses
[1P06] Neurons, Synapses
[1P07] Autonomic nervous system, Others
[1P08] Endocrine, metabolic physiology,Thermoregulation
[1P09] Nutritional and metabolic physiology,Thermoregulation
[1P10] Environmental physiology
[1P11] Embryology, Regenerative Medicine,Development, Growth, Aging, Medical education, Medical histology

### Poster Presentation 1

### [1P01]
### Ion channels, Receptors, Membrane transport

March 16(Wed), 12:30 - 14:30, Zoom P1

### [1P01-01]
### Effects of GLP-1, GIP, and glucagon on fluid secretion in interlobular ducts isolated from guinea pig pancreas

*Mayuko Higuchi[1] ([1]*Depertment of Human Nutrition, Nagoya University Graduate School of Medicine*)

Roles of glucagon-like peptide-1 (GLP-1), gastric inhibitory polypeptide (GIP) and glucagon in the regulation of pancreatic fluid secretion are not understood. In the present study, we have examined the effects of GLP-1, GIP, and glucagon on fluid secretion and intracellular $Ca^{2+}$ concentrations ($[Ca^{2+}]_i$) in isolated pancreatic duct. Interlobular pancreatic ducts (diameter: 100.150 $\mu$m) were dissected from guinea pig pancreas and cultured for 2-3 hours until both ends were sealed spontaneously. Sealed ducts were superfused with standard $HCO_3^-$-buffered solution at 37℃. The rate of fluid secretion was determined from changes in luminal volume. Changes in $[Ca^{2+}]_i$ were estimated by using fura-2. GLP-1, GIP, and glucagon dose-dependently increased the fluid secretory rate with $IC_{50}$ of ～30pM, ～100pM, and ～100pM, respectively. In ducts pretreated with H89 (30 $\mu$M, an inhibitor of protein kinase A), GLP-1, GIP, and glucagon failed to affect fluid secretion. GLP-1 (1 nM), GIP (1 nM), and glucagon (1 nM) did not affect $[Ca^{2+}]_i$. While GLP-1 (100 pM) and GIP (100 pM) enhanced secretin (30 pM)-stimulated fluid secretion, glucagon (1 nM) inhibited secretin (30 pM)-stimulated fluid secretion. In conclusion, our data suggest that GLP-1, GIP, and glucagon regulate fluid secretion by pancreatic ductal epithelium.

### [1P01-02]
### Transport mechanism of various sugars by sodium-glucose cotransporters (SGLTs)

*Kazuyo Kamitori[1,2], Matsuyuki Shirota[3], Yuichiro Fujiwara[1,2] ([1]*Laboratory of Molecular Physiology & Biophysics, Faculty of Medicine,Kagawa University,* [2]*International Institute of Rare Sugar Research and Education, Kagawa University,* [3]*Division of Interdisciplinary Medical Sciences, United Centers for Advanced Research and Translational Medicine, Tohoku University Graduate School of Medicine*)

Sodium-glucose cotransporters (SGLTs) take part in the sugar absorption in human bodies. They also contribute to the extensive glucose uptake during tumor growth. These roles made SGLTs clinical targets. Understanding structural basis of SGLTs sugar selectivity, which is precisely regulated, would provide important information for clinical strategies.

We have reported a homology model for the sugar binding states of human SGLT1, which transports glucose and galactose. Here we show electrophysiological data using various sugars and mutants. The results showed that a mutant at the residue forming hydrophilic interactions with the sugar transported mannose and allose. Another mutant changed the glucose/galactose transport ratio, which reproduces the sugar selectivity of the vibrio galactose transporter. A mutant at three residues reproduced the SGLT4 sugar selectivity, which transports mannose and fructose. These results revealed the structural basis of the selective sugar transport by SGLT, which may be useful for drug design targeting SGLTs. COI;NO

### [1P01-03]
### SLC26A6, a $Cl^-$/H $CO_3^-$ . exchanger, is functionally expressed in the apical membrane of bovine parotid acinar cells

*Soichiro Yamaguchi[1], Tatsuki Inoue[2], Yano (Nashimoto) Saori[1] ([1]*Laboratory of Physiology, Department of Basic Veterinary Sciences, Faculty of Veterinary Medicine, Hokkaido University,* [2]*Laboratory of Physiology, Department of Basic Veterinary Sciences, School of Veterinary Medicine, Hokkaido University*)

The parotid acinar cells of ruminants secrete primary saliva containing a high concentration of $HCO_3^-$. We previously reported that an electrogenic $Na^+$-$HCO_3^-$. cotransporter is functionally expressed at the basolateral membrane of the bovine parotid acinar cells. However, the exit pathway of $HCO_3^-$ in the apical membrane has not been elucidated. Recently, a $Cl^-$/$HCO_3^-$. exchanger, SLC26A6, was reported to be expressed in the apical membrane of mouse mandibular acinar cells. Therefore, in this study, we investigated whether SLC26A6 is functionally expressed in the apical membrane of the bovine parotid acinar cells. First, two splice variants of bovine *SLC26A6* were detected by RT-PCR. Second, western blotting and immunostaining showed that SLC26A6 was expressed in the bovine parotid gland and was localized mainly to the apical membrane of the acinar cells. Finally, to evaluate the function of SLC26A6, we measured intracellular $Cl^-$ concentration and intracellular pH of the HEK293T cells which overexpressed the bovine SLC26A6 variants and freshly-isolated bovine parotid acinar cells. In both cells, the intracellular $Cl^-$ concentration was reduced by extracellular application of $HCO_3^-$. and oxalate, which SLC26A6 can transport. The intracellular pH was increased by the reduction of the extracellular $Cl^-$ concentration in the presence of $HCO_3^-$. These results suggest that SLC26A6 is functionally expressed in the apical membrane of the bovine parotid acinar cells and plays a role in the transport of $HCO_3^-$.

### [1P01-04]
### Effects of hypotonic stress on prostaglandin $E_2$ transport and Maxi-Cl channel activities of SLCO2A1

*Yoshinobu Nakamura[1], Masa-aki Ito[2], Toshiaki Okada[3], Yasunobu Okada[4], Isao Matsuoka[2], Takeo Nakanishi[1] ([1]*Laboratory of Membrane Transport for Biopharmaceutics, Faculty of Pharmacy, Takasaki University of Health and Welfare,* [2]*Laboratory of Pharmacology, Faculty of Pharmacy, Takasaki University of Health and Welfare,* [3]*Veneno Technologies Co. Ltd.,* [4]*National Institute for Physiological Sciences (NIPS)*)

SLCO2A1 is a high-affinity prostaglandin (PG) transporter, and was recently reported to be a major component of Maxi-Cl channel by forming a complex with annexin A2 (Anxa2) in murine mammary tumor line C127 cells, which serves as an effective transport pathway for inorganic and organic anions induced by hypotonic stress. We aimed to study the impact of hypotonicity on Maxi-Cl channel and PG transport activities of SLCO2A1 in C127 cells. Patch-clamp analysis showed that hypotonic stress induced Maxi-Cl currents in C127 cells. On the other hand, $PGE_2$ uptake by C127 cells could be observed in both hypotonic and isotonic medium. Hypotonicity enhanced $PGE_2$ accumulation in the steady sate, but failed to affect its initial uptake rate. In *Slco2a1*-knockout C127 cells, the Maxi-Cl current and $PGE_2$ uptake were both significantly reduced, demonstrating that SLCO2A1 exhibits both activities in the identical cells. Moreover, *Anxa2*-deficiency decreased Maxi-Cl currents in C127 cells, but the rate of initial $PGE_2$ uptake was little affected. Thus, PG transport and Maxi-Cl channel activities of SLCO2A1 are independently regulated by hypotonic stress.

### [1P01-05]
### A mathematical model of solutes and water transport in proximal tubule

*Yuto Kunimasa[1], Taiki Tahara[1], Taiki Nishizuka[1], Yukiko Himeno[1], Junichi Taniguchi[2], Akira Amano[1] ([1]*Ritsumeikan University,* [2]*Jichi Medical University*)

The transport of solutes and water in renal proximal tubule (PT) has been experimentally confirmed but has not been quantitatively elucidated. For quantitative analysis, we simulated the transport of solutes and water in the PT. The model of PT is supposed as follows. Epithelial cells are connected by tight junction (TJ). The luminal membrane contains SGLT, NHE, $K^+$ channels. In the basolateral membrane, NaK pump supplied with sufficient ATP, GLUT, NBC, and $K^+$ channel were placed. $CO_2$ generated from luminal $NaHCO_3$ diffuses into the cell. Ion transport via ion channel and TJ follows Goldman- Hodgkin-Katz equation. Water is osmotically transported via water channel and TJ, which is accompanied by solvent drag of solutes. Available transport parameters obtained by experiments were used in our study. Luminal and interstitial concentrations of each solute and intracellular pH were constant. In our model solutes and water except $K^+$ was reabsorbed along the PT. $K^+$ was secreted at low concentration in the lumen supposing early PT, but it was reabsorbed at high concentration supposing late PT. Our model realized the transports of solutes and water along the PT.

### [1P01-06]

### Analysis of transport mechanism of SLC34 $Na^+$/Pi cotransporter

*Junxian Zhou[1], Yoshifumi Ookouchi[1], Yasushi Okamura[1], Natsuki Mizutani[1], Hiroko Segawa[2], Yuji Shiozaki[2] (*[1] Integrative Physiology, Graduate school of medicine, Osaka university, [2] Department of Applied nutrition, Institute of Biomedical Sciences, Tokushima University Graduate School* )

Sodium-phosphate (NaPi-II) cotransporters maintain phosphate homeostasis by mediating intestinal absorption (NaPi-IIb) and renal reabsorption (NaPi-IIa and IIc) of inorganic phosphates (Pi). Many mutations in IIc cause hereditary hypophosphatemic rickets with hypercalciuria (HHRH); however, much of the mechanism that underly Pi transport in IIc remains unclear. Here, we performed whole-cell patch clamp recordings paired with fluorescent imaging to analyze the transport function of IIc in relation to its localization at the plasma membrane. Due to the electroneutral nature of IIc, we used an electrogenic mutant form of the mouse IIc, which was previously established by Bacconi et al. in 2005. Here, we show that two mutations that are known to cause HHRH (S137F and R563C), exhibited not only reduced membrane expression but also reduced Pi-induced currents. This result suggests that the reduced trafficking of IIc to the plasma membrane causes the reduced currents as reported previously. We investigated another mutation, which was recently discovered in a patient experiencing hyperphosphatemia with hypercalciuria. In contrast to S137F and R563C, I196V exhibited enhanced Pi-induced currents despite normal membrane expression. Our finding may explain the mechanism causing hyperphosphatemia in the patient. Our results also provide a clue to understand the mechanism of Pi transport in IIc.

### [1P01-07]

### miRNA status in the extracellular vesicles released from cadmium exposed hepatocytes

*Wataru Miyazaki[1], Jo Sakuragi[1], Kyoko Mekata[2], Daisuke Matsumaru[2], Tsuyoshi Nakanishi[2] (*[1]Department of Bioscience and Laboratory Medicine, Hirosaki University Graduate School of Health Sciences, [2]Laboratory of Hygienic Chemistry and Molecular Toxicology, Gifu Pharmaceutical University*)

Exosomes, one of the extracellular vesicles (EVs), play an essential role in the cellular signaling pathway for organ homeostasis. Several stimuli, including environmental pollutants, induce the release of EVs from the injured cells, and the released EVs may cause adverse effects on the target cells. Previously, we reported that the exposure of cadmium (Cd), heavy metal and known as a causative agent of Itai-Itai diseases, induced the release of abnormal EVs from hepatocytes, and the EVs suppressed the differentiation of osteoblasts. Although the toxicities by the EVs may be a new toxicological pathway, it is still unknown the mechanisms through the EVs.
In this study, to clarify the mechanisms of the EVs from Cd-exposed cells on osteoblast differentiation, we focused on the RNA status, especially micro-RNA (miRNA) in the EVs released from human hepatocellular carcinoma cells: HepG2, and conducted the miRNAseq. The 273 or 553 miRNAs in the Cd-exposed cells were four times lower or higher than no-Cd, respectively. To estimate the target mRNAs of these miRNAs, we searched them using the database (TargetScan and miRDB) and found that several mRNAs, including for osteoblast differentiation, may be the targets for these miRNAs. These results indicated that the miRNAs in the EVs may be one of the critical regulators for bone metabolism.

### [1P01-08]

### Estimation of FRET efficiency between B-type GABA receptor and type-1 metabotropic glutamate receptor by gradual acceptor photobleaching

*Kengo Kirinoki[2], Takuya Mori[3], Taito Takahashi[3], Yuji Kamikubo[4], Hakushun Sakairi[4], Takashi Sakurai[4], Toshihide Tabata[1] (*[1]Lab. Biol. Info. Processing, Fac. Eng., Univ. Toyama, [2]Grad. Sch. Sci. & Eng., Univ. Toyama, [3]Dept. Eng., Univ. Toyama, [4]Dept. Pharmacol., Juntendo Univ. Sch. Med.*)

In cerebellar Purkinje cells, interaction between B-type gamma-amino butyric acid receptor (GABAbR) and type-1 metabotropic glutamate receptor (mGluR1) leads to facilitation of the longterm depression of postsynaptic glutamate responsiveness, a cellular basis for cerebellar motor learning. Here we examined how efficiently GABAbR can interact with mGluR1 using modified gradual acceptor photobleaching. We created HEK293 cells co-expressing GABAbR subunit 1 with a Halo-tagged N-terminus, GABAbR subunit 2, and mGluR1 subunit with a SNAP-tagged Nterminus. The corresponding subunits were labeled with membrane-impermeant donor (HaloTag Alexa Fluor 488, Promega) and acceptor (SNAP-Surface 594, New England Biolabs) fluorescent substrates. Under confocal microscopy, serial image capture and photobleaching were performed at an interval of 3 s using 488 nm (power, 1%) and 638 nm (power, 100%; pixel dwell time, 20 μs) lasers. For each fluorescent punctum, a function of the following form was fitted to the timecourse of the donor' s fluorescence intensity: $I_{donor}(i) = I_{donor}(0) \cdot [1-E(0) \cdot \exp(-i/tau_{acceptor})] \cdot [A_{fast} \cdot \exp(-i/tau_{donor,fast}) + A_{slow} \cdot \exp(-i/tau_{donor,slow})] + C$, where $i$, $E(0)$, *tau's*, *A's*, and $C$ were the frame number, initial Förster resonance energy transfer efficiency, decay time constants measured by photobleaching the bare donor and acceptor, scale factors, and offset, respectively. The $E(0)$ estimated from the fitted functions was $0.55 \pm 0.24$ (n = 82), suggesting a massive GABAbR-mGluR1 interaction occurring on the cell surface.

### [1P01-09]

### Currents of mechanosensitive channels induced by using single glass pipette

*Yasunori Takayama[1], Mami Kato[1,2], Kenta Maruyama[1,3], Masataka Sunagawa[1] (*[1] Dept Physiol, Showa Univ Sch Med, [2] Dept Mol & Syst Pharmacol, Grad Sch Pharm Sci, Kyushu Univ, [3]Div Cell Signal, Nat Ins Physiol Sci*)

Findings of mechanosensitive channels located on plasma membrane electrified us. This kind of ion channel has been suggested the importance in physiological or pathological mechanisms including touch sensation and cancer proliferation. However, the mechanosensitivity of channels is inconsistent between previous reports, although there are several methods to mechanically stimulate cell membrane. To further investigate the mechanosensitivity, we modified method of mechanical stimulation, and re-analyzed the currents of mechanosensitive channels including Piezo1, Piezo2, and TACAN (also called TMEM120A) in HEK293T cells. We performed whole-cell patch-clamp recording, and also used the recording electrode for mechanical stimulation to the cells. Furthermore, we compared the coverslip coating conditions by using Matrigel or polyethylenimine. In the results, the averaged amplitude was approximately -350 pA (maximum: -500 pA) at -90 mV holding potential in non-coated coverslips. We observed approximately -10 nA current in Matrigel-coated coverslips although the current size was not changed in polyethyleniminecoated coverslips. Piezo2 and TACAN currents were not observed in any experimental conditions. Totally, only Piezo1 is clearly mechanosensitive channel in our method.

### [1P01-10]

### TRPA1 activation by food intake promotes intestinal motility

*Koji Shibasaki[1], Amane Tateishi[1], Hitomi Murata[1], Shunsuke Sudo[1] (*[1]Laboratory of Neurochemistry, Graduate School of Human Health Science, University of Nagasaki*)

It has been reported that TRPA1 is activated by various stimuli such as cold (<17°C), Allyl isothiocyanate (AITC in Wasabi and mustard oil), extracellular alkaline condition and mechanical stimulus. All TRP channels have unique properties called as synergistic effects. If we apply two different agonists, thresholds of each agonist can be effectively reduced. Thus, we can observe significant TRP channel activation by combination of two different agonists. These backgrounds indicate that TRPA1can be potentiated by weak alkaline condition. In this study, we examined the possibility by an electrophysiological experiment. We ectopically expressed mouse TRPA1 in Xenopus oocytes, and examined the effects of extracellular alkaline condition on TRPA1 activation by AITC. Although we failed observed TRPA1 activation in weak alkaline condition, AITC-activated TRPA1 currents were significantly potentiated in the alkaline condition compared with those in normal pH (pH7.4). These results indicate that alkaline condition significantly reduces the thresholds for AITC responses. Furthermore, we revealed that these properties promotes intestinal motility.

This work is supported by a Research Grant from Urakami Foundation for Food and Food Culture Promotion.

### Poster Presentation 1

### [1P02]
### Ion channels, Receptors

March 16(Wed), 12:30 - 14:30, Zoom P2

### [1P02-01]
**The proper distance between the S1 segment and KCNE3 is crucial for the constitutively open nature of the KCNQ1-KCNE3 $K^+$ channel.**

*Go Kasuya[1], Koichi Nakajo[1] ([1] *Division of Integrative Physiology, Department of Physiology, Jichi Medical University School of Medicine*)

The KCNQ1-KCNE3 channel is a constitutively open $K^+$ channel expressed in the basolateral side of epithelial cells, where it plays a crucial role in $K^+$ recycling and thereby facilitates $Cl^-$ secretion from the apical side of epithelial cells. The KCNQ1-KCNE3 channel is composed of two transmembrane proteins, KCNQ1 and KCNE3. KCNQ1 is a voltagegated $K^+$ channel containing a $K^+$ selective pore and four identical voltage sensors. KCNE3 is a single-transmembrane protein that binds to the KCNQ1 voltage sensor to modulate its movements and thus makes KCNQ1 a constitutively open channel. However, how KCNE3 protein modulates the KCNQ1 voltage sensor is almost unknown. In this study, by utilizing the recently determined KCNQ1-KCNE3 structure, we identified a series of amino acid residues on KCNE3, which faces the S1 segment in the KCNQ1 voltage sensor, is responsible for the constitutive activity. By changing the side-chain bulkiness of these interacting amino acid residues, we found that the distance between the S1 segment and KCNE3 is tightly coupled for the constitutive activity. In addition, we identified some pairs of KCNQ1 and KCNE3 mutants that partially recovered constitutive activity by coexpression. Our work suggests tight binding of the S1 segment and KCNE3 is crucial to control the voltage sensor domains.

### [1P02-02]
**Pharmacological properties and physiological roles of two zebrafish HCN4 channels**

*Jiaying Liu[1], Go Kasuya[1], Buntaro Zempo[1], Koichi Nakajo[1] ([1] *Division of Integrative Physiology, Department of Physiology, Jichi Medical University*)

HCN4 channel is a vital component for controlling the heart rate by producing pacemaker potential in specialized myocardial cells such as the sinoatrial node. Zebrafish is an attractive model animal for cardiovascular research because of its transparency during the early stages. However, most zebrafish cardiac ion channels' electrophysiological and pharmacological properties remain less studied. In addition, zebrafish have two HCN4 genes (*DrHCN4* and *DrHCN4L*), and their physiological roles have not been examined yet. We analyzed the pharmacological properties of the two zebrafish HCN4 channels expressed in Xenopus oocytes and found that they were sensitive to the known HCN4 inhibitors ($Cs^+$, ZD7288 and ivabradine). We next examined the effects of the inhibitors on the heart rate of zebrafish during early developmental stages and found that ivabradine and ZD7288 reduced the heart rate of zebrafish. Finally, we assessed the physiological roles of each HCN4 gene during early developmental stages by knocking down the HCN4 genes using anti-sense morpholino. We found that knocking down either of the HCN4 genes at least temporarily reduced the heart rate of zebrafish. Interestingly, knocking down of DrHCN4L tended to induce pericardial edema and early death, implying its importance for early heart development. These results suggest that both DrHCN4 and DrHCN4L play significant roles in regulating the zebrafish heart rate.

### [1P02-03]
**Interaction of the voltage sensor domain with the cytoplasmic catalytic region in voltage-sensing phosphatase analyzed by a fluorescent unnatural amino acid**

*Natsuki Mizutani[1], Akira Kawanabe[1,2], Yasushi Okamura[1] ([1] *Integrative Physiol, Grad Sch Med, Osaka Univ,* [2] *Present address: Mol Physiol and Biophys, Fac Med, Kagawa Univ*)

Voltage-sensing phosphatase (VSP) consists of a voltage sensor domain (VSD) and the cytoplasmic catalytic region (CCR), exhibiting the voltage-dependent phosphatase activity. Activation of the VSD couples with the phosphatase activity (VSD-CCR coupling). A hydrophobic part in the CCR, hydrophobic spine (HS), which plays critical roles in the coupling with its hydrophobicity has been reported, however, the detailed mechanism of the coupling is still unclear. We have found another hydrophobic part (I233 and F234) at the C-terminal end of the fourth segment of the VSD, S4, in *Ciona intestinalis* VSP critical for the coupling based on the findings that hydrophilic substitutions of these residues diminished the coupling. To reveal the relationship between both parts, we analyzed structural rearrangements of I233 and F234 replaced with a fluorescent unnatural amino acid, Anap, using voltage clamp fluorometry method on *Xenopus* oocytes. Attenuation of I233Anap and F234Anap fluorescence upon membrane depolarization was more remarkable with Trp mutation in the HS, consistent with the fluorescence quenching and interactions between both parts. Fluorescence spectroscopic analysis showed a depolarization-dependent leftward-shift in I233Anap emission spectrum in agreement with an innate profile of Anap spectrum sensitive to hydrophilicity-hydrophobicity exposure, suggesting the C-terminal end of S4 accesses the HS. These suggest that voltage-induced interactions of the VSD with the CCR mediates the electrochemical coupling in VSP.

### [1P02-04]
**Evaluation of the membrane curvature-dependent conformational change of the KcsA potassium channel using a fluorescence probe**

Misuzu Ueki[1], *Masayuki Iwamoto[1] ([1] *Univ. Fukui*)

Biomembranes are never flat lipid bilayers but have localized structures with large curvatures, such as caveolae, transport vesicles, and exosomes. Inside the highly curved lipid bilayer, the tension in the outer leaflet is asymmetrically increased, activating mechanosensitive ion channels. However, the effects of membrane curvature on the structure and function of membrane proteins other than mechanosensitive channels remain elusive.
This study aims to clarify the membrane curvature effects on the structure of the KcsA, a prototypical potassium channel that consists only of essential ion channel structures. We reconstituted the KcsA into liposomes of different diameters and evaluated the curvaturedependent conformational change using a fluorescent probe labeled on the activation gate. We found that the KcsA takes inside-out orientation in the liposomal membrane regardless of the liposome size, indicating that the activation gate interacts with the outer leaflet of the liposome. The fluorescence measurements showed that the membrane curvature affected the conformation of the activation gate at acidic pH, where the KcsA is in the active state but not at neutral pH, where it is in the resting state.

### [1P02-05]
**Search for intracellular signals affecting the activity of acid-sensitive outwardly rectifying anion channel (ASOR)**

*Kaori Sato-Numata[1,2], Tomohiro Numata[2], Yasunobu Okada[3,4,5] ([1] *Japan Society for the Promotion of Science,* [2] *Department of Neurophysiology, Graduate School of Medicine, Akita University,* [3] *National Institute for Physiological Sciences,* [4] *Department of Physiology, School of Medicine, Aichi Medical University,* [5] *Department of Physiology, Kyoto Prefectural University of Medicine*)

The acid-sensitive outwardly rectifying anion channel (ASOR) is an anion channel, that is activated when the extracellular pH becomes acidic, causing anion transport in the cell. The core molecule of ASOR was identified in 2019 as TMEM206. However, the detailed molecular mechanisms of ASOR activation including intracellular signaling remain unresolved, although ASOR activity is known to be independent of intracellular $Ca^{2+}$. In the present study, we evaluated the effects of intracellular signal inhibitors on ASOR activity using epithelial HeLa cells by a patch-clamp method. The results showed that acidactivated ASOR currents were significantly suppressed in cells pretreated with either inhibitors of PI3K, Akt or p38, compared with those in control cells. In contrast, there was no difference in ASOR activity between cells pretreated with inhibitors of RIPK1, PKC or MEK and control cells. In addition, there was no difference between the ASOR activities observed in the presence and absence of $H_2O_2$ in HeLa cells. These data suggest that ASOR is activated via PI3K-Akt and p38 signaling in a manner independent of RIPK1, PKC, MEK and ROS.

### [1P02-06]
### Regulation of muscarinic acetylcholine receptor M2 by Sigma-1 receptor

*Chang Liu[1,2], I-Shan Chen[1,2,3], Ruth Murrell-Lagnado[4], Yoshihiro Kubo[1,2] ([1]*Div. Biophys and Neurobiol, NIPS,* [2]*Physiol. Sci, SOKENDAI, ,* [3]*Dept. Pharmacol. Wakayama Med. Univ.,* [4]*School of Life Sciences, University of Sussex*)

Sigma-1 receptor (S1R) is a protein mainly expressed on the endoplasmic reticulum membrane and relevant to many psychiatric and neurological disorders. It has been reported to colocalize with muscarinic acetylcholine receptor M2 (M2R) on the soma of motoneurons in the mouse brainstem and spinal cord while the functional aspects remain unknown. To clarify whether and how could S1R interact with M2R, we performed experiments and observed the results as follows. (1) By patch-clamp, we confirmed S1R down regulates the function of only M2R among other types of $G_{i/o}$ coupled receptors in HEK 293T cells. But the natural disease mutant S1R E102Q did not regulate the function of M2R. (2) By making various chimeras and mutants between M2R and M4R, we identified E172 and E175 on the extracellular loop 2 of M2R are essential for the regulation by S1R. (3) By immunohistochemical staining and confocal imaging, we observed that the expression level of M2R on the plasma membrane (PM) is not down regulated by S1R and that S1R localizes close to the PM. Taken together, our data shows that S1R down regulates, not the expression on the PM, but the function of M2R via the extracellular loop 2.

### [1P02-07]
### Identification of mechanisms underlying the abnormal ion selectivity induced by gene mutations of GIRK2 channel

*I-Shan Chen[1,2,3], Yoshihiro Kubo[1,2] ([1]*NIPS,* [2]*SOKENDAI,* [3]*Wakayama Med. Univ.*)

Ion selectivity in $K^+$ channels is known to be largely determined by the structure of the selectivity filter (SF). However, some mutations located outside the SF region are also reported to alter the ion selectivity. In this study, we aim to clarify the mechanisms underlying the abnormal ion selectivity of GIRK2 channel triggered by mutations in non-SF region. A previous study proposed that the abnormal ion selectivity of L173R, a hyperkinetic movement disorder-related mutation located in the transmembrane domain, is due to a formation of a novel electrostatic interaction between L173R and E150 in the pore helix. To confirm this, we replaced E150, and its well-known ionic pair R160 in WT located above the SF, with neutral or opposite charged amino acid residues and observed the following results: (1) L173R, E150Q and L173R-E150Q mutants show an abnormal large Rb+ current, suggesting that the electrostatic interaction between L173R and E150 is not essential to trigger the abnormal ion selectivity; (2) R160Q shows an extremely large $Li^+$ current, while L173R-R160Q shows a much smaller $Li^+$ current than that of R160Q and a large $Rb^+$ current. Taken together, the results show that the abnormal ion selectivity in L173R is not due to a formation of the electrostatic interaction between R173 and E150, but due to a loss of that between E150 and R160. It is likely that a complicated interaction network behind the SF plays important roles in modulating SF conformation.

### [1P02-08]
### Generation of a novel mouse line for optogenetic inhibition and its functional assessment using electrophysiology

*Yan Li[1], Yasutaka Mukai[1], Manabu Abe[2], Kenji Sakimura[2], Keiichi Itoi[3], Akihiro Yamanaka[1] ([1]*Nagoya University,* [2]*Niigata University,* [3]*Tohoku University*)

Until recently, in the field of optogenetics, to silence the activity of cells, people used lightinducible chloride ion pump, halorhodopsin (HR), or light-inducible proton pump, archaerhodopsin (Arch). In 2015, anion channelrhodopsin, or ACR, was discovered. ACR is a light-inducible channel permeates chloride ions, and has 2 subtypes, ACR1 and ACR2. Compared to HR and Arch, ACR has extremely high light sensitivity and operates rapidly according to previous studies.Until now, however, there are no transgenic mice expressing ACR2. Therefore, adeno-associated virus (AAV) injection is used to express ACR2, which requires time and technique. Hence, in this study, we generated a novel transgenic mouse line, *Rosa26-LSL-ACR2-EYFP(LSL-ACR2),* which expresses ACR2 in Cre-inducible manner.We mated the line with a noradrenergic (NA) Cre-driver line (*NAT-Cre*) to obtain *LSL-ACR2;NAT-Cre* mice. By immunohistochemistry, we confirmed ACR2 was exclusively expressed in NA neurons. Through slice patch-clamp recordings, we assessed the changes of current and membrane potential under different intensity and duration of 470 nm light stimuli. As a result, the activity of NA neurons expressing ACR2 could be inhibited. These results provided a theoretical basis for the later measurement of behavior.In conclusion, we generated *LSL-ACR2* mouse line, which is expected to solve the time and technical limitations of AAV injection, and reduce the phototoxicity to cells.

### [1P02-09]
### Negative allosteric modulation of nicotinic acetylcholine receptors by pancuronium

*Souhei Sakata[1], Ono Fumihito[1] ([1]*Dep.of Physiol., Faculty of Medicine, Osaka Medical and Pharmaceutical University*)

Pancuronium is a muscle relaxant and was believed to competitively inhibit the muscle nicotinic acetylcholine receptor (nAChR). Recent works using zebrafish demonstrated that nAChR pentamers in fast fibers contain one $\varepsilon$ and one $\delta$ subunit, while those in slow fibers have two $\delta$ subunits instead. We designate these two types of nAChRs as $\varepsilon$-type and $\delta$-type, respectively. In this study, we examined the inhibitory effect of pancuronium against these two types of nAChRs using the Xenopus oocytes heterologous expression system. The half maximal inhibition concentration ($IC_{50}$) of pancuronium was $1.5 \pm 0.2$ $\mu$M and $9.6 \pm 4.0$ $\mu$M for $\varepsilon$-type and $\delta$-type, respectively. Chimera experiments between the $\varepsilon$- and the $\delta$-type nAChR revealed that $IC_{50}$ was associated not with the extracellular ligand binding region but the transmembrane region. Further analysis of chimeras showed that intracellular region of nAChR was associated with the affinity against pancuronium, raising a possibility that pancuronium does not work as a competitive inhibitor but a negative allosteric modulator.

### [1P02-10]
### Activation of mechanosensitive Piezo1 channel suppresses brown adipocyte differentiation

*Manato Kenmochi[1], Kawarasaki Satoko[2], Okamura Kazuhiko[3], Goto Tsuyoshi[2], Uchida Kunitoshi[1,4] ([1]*Grad. Sch. Integr. Pharm. Nutr. Sci., Univ. Shizuoka,* [2]*Div. Food Sci. and Biotec., Grad. Sch. Agric., Kyoto Univ.,* [3]*Dept. Morphol. Biol. Fukuoka Dent. Col.,* [4]*Lab. Funct. Physiol., Dept. Env. Life Sci., Sch Food Nutr. Sci., Univ. Shizuoka*)

Brown adipocytes cause an energy consumption by heat production and are thought to be a target for the prevention of obesity and related metabolic disorders. Piezo1 is a $Ca^{2+}$- permeable non-selective cation channel and activated by mechanical stimuli. Piezo1 has been reported to be involved in mechano-sensation in non-sensory tissues. However, the expression and roles of Piezo1 in brown adipocytes have not been well clarified. Here, we evaluated a brown adipocytes line from UCP1-mRFP1 transgenic mice. Using this cell, we showed that Piezo1 was expressed in pre-adipocytes. Application of Yoda-1, a Piezo1 agonist suppressed brown adipocytes differentiation in a dose-dependent manner. This suppression was significantly recovered by co-application with a Piezo1 antagonist and a calcineurin inhibitor. Furthermore, knock-down of Piezo1 impaired Yoda-1-induced suppression of brown adipocyte differentiation. These results suggest that activation of Piezo1 might suppress the differentiation through calcineurin pathway in brown preadipocytes. COI: NO.

### Poster Presentation 1

### [1P03]
### Molecular physiology, Cell biology, Others

March 16(Wed), 12:30 - 14:30, Zoom P3

### [1P03-01]
**Involvement of intracellular second messengers and cytoskeleton in the exocytosis of intestinal hormone**

*Kazuki Harada[1], Maoko Takashima[1], Tetsuya Kitaguchi[2], Takashi Tsuboi[1] ([1]*Department of Life Sciences, Graduate School of Arts and Sciences, The University of Tokyo,* [2]*Laboratory for Chemistry and Life Science, Institute of Innovative Research, Tokyo Institute of Technology*)

Glucagon-like peptide-1 (GLP-1) is one of incretin hormone, and is released from entetoendocrine L cells in the intestine. When these cells sense various intraluminal nutrients and chemicals, changes in the levels of $Ca^{2+}$ and cAMP, and reorganization of cytoskeleton regulate the GLP-1 secretion. However, it is unclear how intracellular second messengers (i.e., $Ca^{2+}$ and cAMP) and cytoskeleton collaborate to regulate GLP-1 secretion. Here we used live cell imaging with mouse enteroendocrine L cell line GLUTag cells and monitored the dynamics of intracellular $Ca^{2+}$ and cAMP, and GLP-1 vesicles to elucidate the regulatory mechanisms. We used high $K^+$ stimulation (high $K^+$) which induces an increase of only intracellular $Ca^{2+}$, and deoxycholic acid (DCA) which stimulates both intracellular $Ca^{2+}$ and cAMP elevation. We next visualized GLP-1-containing vesicles by total internal reflection fluorescence microscopy, and classified individual exocytotic events based on the duration of docking to the plasma membrane. We found that the vesicles which are already docked with the membrane or not docked at all were dominantly released immediately after stimulation, under either high $K^+$ or DCA. Interestingly, inhibition of actin polymerization with latrunculin A lowered the ratio of those exocytotic events. These results suggest that GLP-1 exocytosis is induced by $Ca^{2+}$ and cAMP, but the types of exocytotic events are determined by actin dynamics upon stimulation.

### [1P03-02]
**Thermogenic gene regulation via MYPT1-PP1 β phosphatase complex during beige adipocyte differentiation**

*Hiroki Takahashi[1], Ge Yang[1], Takaheshi Yoneshiro[2], Ryo Ito[1], Yoshihiro Matsumura[2], Juro Sakai[1,2] ([1]*Tohoku University,* [2]*The University of Tokyo*)

When animals are chronically exposed to cold temperatures, thermogenic adipocytes termed "beige adipocytes are recruited to subcutaneous white adipose tissue (scWAT). Recently, we have shown that, under chronic cold stress, JMJD1A, a histone H3 lysine 9 (H3K9) demethylase, induces beige-ing through β-adrenergic-dependent phosphorylation of S265 by PKA and following H3K9me2 demethylation. These results prompt us to hypothesize that inhibition of the pS265-JMJD1A phosphatase might maintain phosphorylation of JMJD1A and would result in the induction of beige-ing through the stabilization of epigenetic change induced by pS265-JMJD1A in response to cold stress. However, phosphatase of S265 phosphorylation of JMJD1A and its role in beige-ing has not been investigated. Here we identified MYPT1-PP1 β as a phosphatase of pS265-JMJD1A. Depletion of MYPT1-PP1 β induces JMJD1A phosphorylation and beige-selective genes, which accompanies a decrease in H3K9me2 on these gene loci. MYPT1-PP1 β also targets phosphorylation of RLC to regulate *Ucp1* transcription through the modulation of actomyosin tension. Under β-adrenergic stimulation, MYPT1 is phosphorylated by PKA at T694, which is associated with the inhibition of dephosphorylation activity. Our findings suggest a mechanism of beige-selective gene induction in which MYPT1-PP1 β activity is suppressed under cold stress to ensure efficient phosphorylation of JMJD1A and RLC, orchestrating chromatin architecture and actomyosin tension mediated transcriptional network.

### [1P03-03]
**Cesium application depresses glycolysis pathway in HeLa cell**

*Daisuke Kobayashi[1], Natsumi Nishimura[1], Akihiro Hazama[1] ([1]*Dept. Cellular and Integrative Physiology, School of Medicine, Fukusima Medical Univ.*)

Cesium (Cs) is not essential alkali metal element for human, and it has no known beneficial function in human verified by clinical research. Although Cs causes toxicity at excessive dosage, it has been used an alternative therapy for treating cancer. We should clarify the biological effects of Cs on cells to use Cs in clinical treatment. On the other hand, Cs was able to suppress human cervical cancer cells proliferation in a dose-dependent manner and it was assumed that Cs inhibited glycolysis pathway. In this study, we clarified which step of the glycolysis pathway was affected by Cs. We investigated an effect of Cs on glycolysis enzyme expressions, activities, and metabolite contents in HeLa cells by treatment with Cs for 3 days. Cs-treatment decreased transcriptional and expression levels of glycolytic enzyme activity. The determination of glycolytic pathway metabolite revealed that lactate content was decreased and $NAD^+$ was increased by Cs-treatment; however, pyruvate and NADH content did not changed. Increase of the $[NAD^+]/[NADH]$ ratio indicated that aerobic glycolysis pathway was inhibited by Cs-treatment. Cs-treatment showed inhibition of pyruvate kinase activity and increase of the $[NAD^+]/[NADH]$ ratio; therefore, glycolytic pathway, especially aerobic glycolysis inhibited by Cs. These results suggested that suppression of cell proliferation by treatment with Cs in HeLa cells was caused inhibition of aerobic glycolysis by Cs.

### [1P03-04]
**Organization and dynamics of cortical actin cytoskeleton regulated by cytosolic $Ca^{2+}$ in mammalian eggs**

*Hideki Shirakaw[1], Kento Kondo[1], Hinako Muratani[1] ([1]*Dept. Eng. Sci., Univ. Electro-Comm.*)

Cortical actin filaments (F-actin) beneath the plasma membrane of mammalian eggs are involved in various events at fertilization, such as sperm incorporation and cortical reaction. We investigated cortical F-actin dynamics in mouse eggs and their $Ca^{2+}$-dependent changes during egg activation. Visualization of F-actin by fluorescent probes revealed the directional flow of cortical actin, which starts and spreads radially from the actin cap (AC), a thick layer of F-actin at the animal pole. As leaving the AC region, bundles of F-actin are organized to form microvilli on the egg surface. Repetitive transient rises in cytosolic $Ca^{2+}$ concentration induced by egg-activating protein PLC ζ pause the cortical F-actin flow temporally, and it ceases when the AC is disrupted after a few $Ca^{2+}$ rises. Fluorescence of actin probes at the cortex increases transiently at each $Ca^{2+}$ rise, indicating $Ca^{2+}$- dependent reorganization of microvillar F-actin bundles. The experiments with inhibitors suggested that the cortical F-actin flow is driven by Arp2/3 and formins are also necessary to maintain the flow. Calcium-dependent reorganization of microvillar F-actin is not affected by Arp2/3 or formin inhibitors, and may be mediated by $Ca^{2+}$-sensitive actin-bundling proteins such as plastins.

### [1P03-05]
**Physiological functions of diacylglycerol acyl transferase (DGAT) in thermotaxis in *Drosophila melanogaster***

*Xiangmei Deng[1], Takuto Suito[1], Takaaki Sokabe[1] ([1]*NIPS*)

Thermotaxis is important for survival and reproduction. In *Drosophila melanogaster* larvae, recent studies showed that the temperature preference shifted when an exogenous polyunsaturated fatty acid (PUFA) generation enzyme was introduced into TRPA1-expressing neurons. However, how endogenous lipid enzymes contribute to thermotaxis and whether their functions are conserved among species remains unknown.
We sought lipid enzyme coding genes involved in PUFA metabolisms in *D. melanogaster* by testing the mutant larvae on a temperature gradient plate. We found that knocking out of three DGAT coding genes, *cool-1*, *cool-2* and *cool-3*, induced a cooler temperature preference compared to the control. We knocked down these genes in the TRPA1-expressing neurons and observed similar cool preference seen in the mutants. These results suggest that DGAT may play regulatory roles in the temperature sensation. We also tested a closely related species of *D. melanogaster, D. simulans*, and the larvae preferred cooler temperature than *D. melanogaster*. We will discuss the mechanisms of DGATdependent thermotaxis and its potential role in the different preferable temperatures among *Drosophila* species.

### [1P03-06]

### Acetylcholine autocrine regulation of airway ciliary beating mediated via α7-nicotinic receptor/Cav1.2

*Daichi Saito[1,2], Kotoku Kawaguchi[1,2], Shinji Asano[1,2], Yoshinori Marunaka[1,3], Takashi Nakahari[1] ([1]*Res. Org. of Sci. and Tech., Ritsumeikan Univ.*, [2]*Dept. Pharm. Sci., Ritsumeikan Univ.*, [3]*Kyoto Industrial Health Association*)

Acetylcholine (ACh), which is a neurotransmitter stimulating muscarinic or nicotinic ACh receptor (nAChR), activates airway ciliary beating via an increase of intracellular $Ca^{2+}$ concentration ($[Ca^{2+}]_i$). ACh (1 μM) increased CBF (ciliary beat frequency) and CBD (ciliary bend distance, an index of ciliary beat amplitude) to 125% in mice airway cilia, and then, further addition of nifedipine (20 μM, an inhibitor of voltage-gated $Ca^{2+}$ (Cav) channels) or MLA (50 nM, an inhibitor of α7-nAChR) decreased them to 115%. An α7-nAChR agonist, PNU282987 (1 μM), did not significantly increased $[Ca^{2+}]_i$, but increased CBF and CBD to 110%, which was inhibited by nifedipine. In experiments using high $K^+$ solutions, as extracellular $K^+$ concentration increased, CBF, CBD and $[Ca^{2+}]_i$ increased, suggesting that depolarization increases $[Ca^{2+}]_i$. Immunoblotting and immunofluorescence studies revealed that Cav1.2 and α7-nAChR exist in the airway cilia. Thus, an activation of α7-nAChR increases $[Ca^{2+}]_i$ via Cav1.2 in airway cilia, not cell body, leading to CBF and CBD increases. Moreover, stimulation with IL-13 increased CBF and CBD in the airway beating cilia, which was inhibited by MLA. This result suggests that IL-13 stimulates ACh release from airway ciliary cells. In conclusion, ACh /α7-nAChR/Cav1.2, a novel autocrine mechanism localized in the cilia, enhances airway ciliary beating.

### [1P03-07]

### Measurement of circular and longitudinal contractions of the muscularis to determine the effects of nitrergic neurotransmission in the murine colon.

*Yao Yu[1], Shinsuke Nakayama[1], Chiho Takai[1], Nao Iwata[1] ([1]*Cell Physiology*)

NO (Nitric oxide) is a major inhibitory neurotransmitter which plays a crucial role in various physiological functions. In the GI (Gastrointestinal) tract, NO participates in the relaxation of smooth muscle via cGMP pathway, which cooperates in regulating GI pacemaker cells referred to as ICC (Intestine of Cajal cell) that generating slow waves in the animal colon. NO is mainly synthesized by NOS (Nitric oxide synthase). Use of L-NNA, the inhibitor of NOS, can therefore block the synthesis of nitric oxide, leading to smooth muscle contraction. In the colon, motility is complex and region-specific. We thus use three different regions of the colon: proximal, middle and distal regions, to analyze the movement of smooth muscle at circular and longitudinal layers by image tracking method. We compared to movements of the colon in the absence and presence of L-NNA, and also performed intracellular $Ca^{2+}$ imaging and potential mapping. With the analysis, these experiments exhibited showed that in the presence of L-NNA, vertical movement was more prominent than lateral movement in the middle and distal regions. In contrast, lateral movement was prominent in the proximal region. It was suggested that L-NNA causes different degrees of influence in circular and longitudinal muscularis. MEA (microelectrode array) potential mapping showed significant changes, indicating that L-NNA affects not only smooth muscle cells but pacemaker interstitial cells.

### [1P03-08]

### Preparation of enzymatically active complex of myosin light chain phosphatase by using yeast expression system

*Tetsuo Yamashita[1], Masumi Eto[2], Katsuya Hirano[1] ([1]*Department of Cardiovascular Physiology, Faculty of Medicine, Kagawa University*, [2]*Biochemistry Unit, Faculty of Veterinary Medicine, Okayama University of Science*)

Myosin light chain phosphatase (MLCP), composed of a catalytic subunit PP1c and two regulatory subunits MYPT1 and M20, dephosphorylates 20 kDa myosin light chain (MLC20) and suppresses vascular smooth muscle contraction. To better understand the molecular and structural base for the suppression of MLCP activity and increase in vascular tone, which lead to the development of cardiovascular disease, preparation of a large amount of the enzymatically active complex of MLCP is a prerequisite. The mammalian expression system has been successful in expressing the enzymatically active MLCP, but in a small scale. The present study reports a new method for preparing a large amount of the enzymatically active complex composed of PP1c and MYTP1 in yeast. In this expression system, PP1c and MYPT1 subunits were expressed as recombinant proteins tagged with N-terminal Myc-His10 and with N-terminal FLAG and C-terminal maltose-binding protein (MBP), respectively, in *S. cerevisiae*. In amylose resin column chromatography for purifying MBP, not only MYPT1 subunit but also PP1c subunit was simultaneously eluted by increasing maltose. The fractions containing both subunits had a purity of about 90%. Phos-tag SDS-PAGE analysis revealed the enzymatic activity of this complex to dephosphorylate MLC20. The results prove the usefulness of the yeast expression system to prepare a large amount of the enzymatically active complex of MLCP for investigating the molecular mechanism of the regulation of MLCP activity.

### [1P03-09]

### Decreased intracellular chloride concentration enhances cell migration and invasion via activation of the ERK signaling pathway in human prostate cancer cell line, DU145

*Junichi Sato[1], Hiroaki Miyazaki[1] ([1]*Div. Life Sci., Grad. Sch. Sci. & Eng., Setsunan Univ.*)

Our previous studies indicated that reducing the intracellular $Cl^-$ concentration of the prostate cancer cell line DU145 facilitates cell migration and invasion, but its mechanism remains unclear. Therefore, we investigated the mechanism of enhancing cell migration and invasion of DU145 cells in the low $Cl^-$ condition. Since the previous studies suggested that ERK/MAPK signaling has been involved in cell migration and invasion in several types of cancer, we first examined the effect of $Cl^-$ on the activation of ERK. In the low Clcondition, phosphorylation levels of ERK were significantly upregulated. The inhibition of ERK activation by the application of MEK inhibitor, U0126, completely abolished the enhancement of cell migration and invasion in the low $Cl^-$ condition. We then performed RT-PCR to determine the mRNA levels of c-*Fos* and MMP-1, both of which were wellknown downstream targets of ERK and were involved in tumor metastasis. The mRNA levels of c-*Fos* and MMP-1 also increased significantly in the low $Cl^-$ condition. In addition, these upregulation of c-*Fos* and MMP-1 mRNA levels were completely eliminated by the application of U0126. From these results, we concluded that the enhancement of cell migration and invasion of DU145 cells in the low $Cl^-$ condition is due to the increase of c- *Fos* and MMP-1 expression by the activation of ERK. (COI: No)

### [1P03-10]

### Effect of Intracellular $Cl^-$ Concentration Changes on apoptotic signaling pathway in MDA-MB231 breast cancer cell

*Mizuki Sada[1], Hiroaki Miyazaki[1] ([1]*Div. Life Sci., Grad. Sch. Sci. & Eng., Setsunan Univ.*)

Apoptotic cell death is performed using a programed signaling pathway, accompanied apoptotic volume decrease (AVD) during induction of apoptosis. AVD is induced by activation of the volume-sensitive outwardly rectifying (VSOR) anion channel and $K^+$ channels, which cause effluxes $Cl^-$, $K^+$ and $H_2O$, leading to cell volume decreases. Therefore, this $K^+$ and $Cl^-$ efflux may induce the reduction of intracellular $Cl^-$ concentration, which is possibly affect the signal pathway of apoptosis. Thus, the aim of this study is to investigate the effect of intracellular $Cl^-$ concentration changes on apoptotic signaling pathways in MDA-MB231 breast cancer cells. We investigated the effect of intracellular $Cl^-$ on staurosporine (STS)-induced activation of caspase-3 and externalization of phosphatidylserine (PS) on the cell plasma membrane, which were observed in the early stage of apoptosis. As a result, the activation of caspase-3 and the externalization of PS were detected earlier in cells cultured in the low $Cl^-$ medium than those in the normal $Cl^-$ medium after induction of apoptosis with STS. These results suggest that the intracellular $Cl^-$ functions as an important regulator of apoptotic signaling pathways. (COI: No)

### Poster Presentation 1

### [1P04]
### Neural network

March 16(Wed), 12:30 - 14:30, Zoom P4

### [1P04-01]
### BTBR T+tf/J mice: Autism-relevant behaviors and neural circuit dysfunctions in the medial prefrontal cortex

*Minobu Ikehara[1], Kazuhiko Yamamuro[1], Kazuya Okamura[1], Yuki Noriyama[1], Nozomi Endo[2], Taketosi Sugimura[3], Mayumi Nishi[2], Yasuhiko Saito[3], Manabu Makinodan[1] (*[1]Department of Psychiatry, Nara Medical University, [2]Department of Anatomy and Cell Biology, Nara Medical University, [3]Department of Neurophysiology, Nara Medical University*)

Previous several studies reported that disrupting cross-hemispheric $\gamma$ synchrony between prefrontal parvalbumin interneuron impairs rule shift learning and enhances persistent behavior. In human, patients with autism spectrum disorder exhibit obsessional behavior. Although BTBR mice, a mouse model of autism, also showed obsessional behavior, the neural circuit has been unknown. In this study, we performed Water T maze and found that persistent behavior was significantly enhanced in BTBR mice compared to C57BL/6 mice at p60-70. We sought to determine the cellular and circuit-level mechanisms that underlie the failure to fully active pyramidal neurons in layer V/VI of the medial prefrontal cortex(mPFC). patch-clamp recording revealed that both spontaneous and miniature excitatory post-synaptic currents frequencies significantly increased in BTBR mice compared to C57BL/6 mice. In contrast and spontaneous and miniature inhibitory postsynaptic currents frequencies significantly reduced in BTBR mice. Together, these changes contributed to a significant increase in the ratio of sEPSC/sIPSC frequency (E/I ratio) in BTBR mice. These results indicate that disrupted excitatory/inhibitory balance in synaptic transmission in pyramidal cells of layer V/VI of the mPFC in BTBR, and may be involved in the enhancement of persistent behavior in BTBR mice.

### [1P04-02]
### The I1166T mutation in Cav1.2 associated with Timothy Syndrome impairs neuronal migration and callosal projections in developing mouse neocortex

*Nao Nakagawa Tamagawa[1,2], Emi Kirino[1], Kohtaroh Sugao[3], Hidetaka Nagata[3], Yoshiaki Tagawa[1] (*[1]Kagoshima University, [2]RIKEN CBS, [3]Sumitomo Dainippon Pharma Co., Ltd.*)

Timothy syndrome (TS) is a multisystem disorder associated with cardiac manifestations, including long QT syndrome, and neurological symptoms, including autism spectrum disorder. The L-type calcium channel Cav1.2 plays key roles in neural development and its mutation causes TS. Recently, a gain-of-function mutation, I1166T, in Cav1.2 was identified in patients with TS-like disorder. Though its channel properties have been analyzed *in vitro, in vivo* effects of this mutation on brain development remain unexplored. In the present study, *in utero* electroporation was performed to express $Cav^{1.2I1166T}$ in layer 2/3 excitatory neurons of the primary somatosensory area, and analyzed neuronal migration and callosal projection. Approximately 20% of $Cav^{1.2I1166T}$-expressing neurons showed impaired migration, and their callosal projection was also markedly reduced. Inhibition of both $Ca^{2+}$ influx and $\beta$-subunit interaction recovered migration and projection. These results suggest that the I1166T mutation impairs neuronal development, which is mediated by both $Ca^{2+}$ influx- and $\beta$ subunit-dependent pathways downstream of Cav1.2. The results of G406R (original mutation of TS), I1166V, and other mutations will also be shown in the presentation. (COI: YES)

Reference: Tamagawa N.N., Kirino E., Sugao K., Nagata H., Tagawa Y. Involvement of Calciumdependent Pathway and $\beta$ subunit-interaction in Neuronal Migration and Callosal Projection deficits caused by the Cav1.2 I1166T Mutation in Developing Mouse Neocortex. Front Neurosci, in press. doi: 10.3389/fnins.2021.747951

### [1P04-03]
### Elucidation of circuitry basis associated with sensory deficits of the 22q11.2 deletion syndrome.

*Yousuke Nakao[1], Shouta Sugio[1], Daisuke Kato[1], Itaru Kushima[2], Daisuke Mori[2], Norio Ozaki[2], Hiroaki Wake[1] (*[1]Nagoya University Graduate School of Medicine, Department of Anatomy and Molecular Cell Biology, [2]Nagoya University Graduate School of Medicine, Department of Psychiatry*)

The 22q11.2 deletion syndrome (22q11.2DS) is a disorder caused by a microdeletion in human chromosome 22q11.2 region. This syndrome displays multiple physical abnormalities such as cardiac malformation, hypocalcemia. In addition, 22q11.2DS is known to increase the risk of developing a variety of psychiatric and developmental disorders. Particularly, the risk of schizophrenia imposed by this deletion is higher than any other schizophrenia-associated single genetic variations that have been reported. In addition, a mice model of 22q11.2 DS (22qdel) shows a broad range of sensory processing similar as in schizophrenia, such as reduction in prepulse inhibition and reduced amplitude of visual evoked potentials. However, the neural circuitry associated with sensory deficits of the 22q11.2 DS are not well understood. Here, we visualized neuronal population activities in the barrel field of the primary somatosensory cortex (S1BF) in 22qdel using *in vivo* two-photon calcium imaging. The amplitude of $Ca^{2+}$ transients in 22qdel are higher than those in control mice, but AUC (Area Under Curve) and frequency did not show a significant difference. These results suggests that abnormal excitation which may be caused by inhibitory dysfunction contribute to the abnormal sensory processing in the 22qdel and that resulted in excitation/inhibition (E-I) imbalance seen in schizophrenia. We are performing extracellular recordings to reveal the firing patterns of neurons and aim to elucidation of the circuitry basis of schizophrenia. This study suggests the abnormal neuronal basis for schizophrenia and future therapeutic targets.

### [1P04-04]
### Elucidating the pathological mechanism of ASD from sensory abnormalities through the visualization of S1 neuronal activity

*Midori Shibushita[1], Daisuke Kato[1,2], Ikuko Takeda[1,3], Daisuke Mori[4], Itaru Kushima[4], Norio Ozaki[4], Hiroaki Wake[1,2] (*[1]Department of Anatomy and Molecular Cell Biology, Nagoya University Graduate school of Medicine, [2]Division of Multicellular Circuit Dynamics, National Institute for Physiological Sciences, [3]Division of Homeostatic Development, National Institute for Physiological Sciences, [4]Department of Psychiatry, Nagoya University Graduate school of Medicine*)

Autism Spectrum Disorder (ASD) is a neurodevelopmental disorder with impaired social interactions and communications, limited interests and repetitive behaviors. ASD is caused by abnormal neural circuit formation due to genetic and environmental factors such as maternal inflammation. One notable symptom in ASD patients is sensory abnormalities. Although neuronal overactivity in the thalamus-sensory cortex has been reported, the pathological mechanism of neuronal circuitry activity that causes sensory abnormalities in ASD patients is not clear.In this study, we used two mouse models of ASD; maternal immune activation (MIA) mice with Poly(I:C) and 3q29 deletion mice, a genetic model of ASD. We visualized the spontaneous and sensory stimulus-induced neuronal activities in the primary somatosensory cortex barrel field(S1BF) using *in vivo* two-photon microscopy combined with virus targeted fluorescent expression. ASD model mice showed low reproducible neural response to whisker stimuli due to its high spontaneous activity, reflecting the variance of the sensory input reception in ASD, that may cause the higher variance of higher order brain function expression. Furthermore, twophoton holographic microscopy revealed the increased neuronal functional connectivity of S1BF local neuronal circuits, which may base on the high spontaneous activity in ASD model mice. This study examines ASD from the perspective of sensory abnormalities, providing a new perspective on the pathogenesis of ASD and exploring new therapeutic targets.

### [1P04-05]
### Subregions of anterior cingulate cortex differentially project to basomedial and basolateral nuclei of amygdala in macaque monkeys

*Rintaro Yoshino[1], Kei Kimura[2], Soshi Tanabe[2], Andi Zheng[2], Shinya Nakamura[1], Shinya Ohara[1], Ken-Ichi Inoue[2], Masahiko Takada[2], Ken- Ichiro Tsutsui[1] (*[1]Laboratory of systems neuroscience, Department of life science, Tohoku University, [2]Systems Neuroscience Section, Primate Research Institute, Kyoto University*)

Medial frontal cortex (MFC) is thought to be involved in the regulation of negative emotion and mood as well as in autonomic responses via its projections to the amygdala. The MFC and amygdala can be divided into subregions and subnuclei, respectively, based on the cytoarchitecture. Clarifying the topographic projections from MFC to the amygdala may be a key to understanding the function of MFC and the pathogenesis of psychiatric disorders related to dysregulation of negative emotion and mood. Previous studies using conventional neural tracers reported that the projections from MFC to the amygdala mainly originate from the subgenual region of the anterior cingulate cortex (ACC) and terminate within the basal amygdaloid nucleus. While these studies have examined the distribution of cells projecting to the basomedial amygdala (BMA) among the basal nucleus, the cells of origin of projections to the basolateral amygdala (BLA) remain unknown. The present study aimed to comprehensively investigate the distribution patterns of MFC neurons projecting to BMA and BLA by using adeno-associated virus vectors that permit anterograde and retrograde tracings. We found that the subgenual region of ACC projects primarily to BMA, whereas the pregenual region of ACC projects to the intermediate part of BLA. Our result suggests that these ACC regions may be involved in distinct roles in the regulation of emotion and mood via different subnuclei of the basal amygdaloid nucleus.

### [1P04-06]

### Monosynaptic rabies virus tracing from projection-targeted single neurons

*Yuji Masaki[1], Masahiro Yamaguchi[1], Ryosuke Takeuchi[1], Fumitaka Osakada[1,2,3] ([1]*Lab. Cell. Pharmacol., Grad. Pharm. Sci., Nagoya Univ.*, [2]*Lab. Neural Info. Proc., Inst. Adv. Res., Nagoya Univ.*, [3]*NLS, Inst. Inn. Fut. Soc., Nagoya Univ.*)

The information received by sensory organs is converted into electrical signals and processed by hierarchically organized parallel circuits that consist of independent subnetworks. The understanding of neural circuits at a finer scale is achieved by a detailed analysis of the neuronal wiring diagram and function of individual presynaptic and postsynaptic neurons. Single cell-initiated monosynaptic G-deleted rabies viruses (RVΔG) tracing allows for understanding the fundamental basis of neural circuits at the cellular resolution. However, the experimental procedures of this method are complex, and the success rate is not high. There has been no validation to increase the accuracy of monosynaptic tracing from targeted neurons. In the present study, we developed an efficient RVΔG tracing method to label single neuronal networks. Molecular biological modifications in viral infection, as well as imaging systems with single-cell electroporation, made it possible to label a target single neuron and its presynaptic networks in various cortical areas, including the primary visual cortex (V1), posteromedial visual cortex (PM) and anteromedial visual cortex (AM). In addition, we labeled subnetworks of V1 single neurons projecting to the higher visual area PM by using AAV2-retro and RVΔG tracing. This method will characterize subnetworks that create the function of each higher visual area

### [1P04-07]

### Neurophysiological analysis of visual processing in Pcdh15 knockout mice.

*Reon Kondo[1,2], Daisuke Kato[2], Itaru Kushima[1], Daisuke Mori[1], Norio Ozaki[1], Hiroaki Wake[2] ([1]*Department of Psychiatry, Nagoya University Graduate School of Medicine,*, [2]*Department of Anatomy and Molecular Cell Biology, Nagoya University Graduate School of Medicine*)

Protocadherin 15 (Pcdh15) is a member of protocadherin family which constitutes the largest subgroup in cadherin superfamily, and the gene coding it is identified to have effects on the onset of various mental disorders. In previous study, Pcdh15 knockout mice showed periodic prominent hyperactivity, and activity-correlated change of c-fos protein expression in visual cortex. Hence, we hypothesized they had altered visual cognition that could affect their behavioral phenotypes that is seen in various mental disorders. Here, we studied visual cognition system of Pcdh15$^{-/-}$ mice. A go/no-go task was combined with in vivo two-photon $Ca^{2+}$ imaging to visualize neuronal activity during visual discrimination and orientation-selectivity in V1 neurons of Pcdh15$^{-/-}$ mice. Impaired visual cognition is reported in patients with neuropsychiatric disorders (such as schizophrenia, bipolar disorder, DLB) and is focused because of their contribution on the progression of other psychiatric symptoms. Therefore, our study contributes to the elucidation of neural basis involved in psychiatric symptoms.

### [1P04-08]

### Endocannabinoid-dependent formation of columnar axonal projection of layer 4 neurons in the mouse cerebral cortex

*Chiaki Itami[1,2], Naofumi Uesaka[3], Jui-Yen Huang[2], Hui-Chen Lu[2], Kenji Sakimura[5], Masanobu Kano[3,4], Fumitaka Kimura[6,7] ([1]*Saitama Medical University, Department of Physiology*, [2]*The Linda and Jack Gill Center for Biomolecular Sciences, Department of Psychological and Brain Sciences, Indiana University*, [3]*Department of Neurophysiology, Graduate School of Medicine, The University of Tokyo*, [4]*International Research Center for Neurointelligence (WPI-IRCN), The University of Tokyo*, [5]*Department of Cellular Neurobiology, Brain Research Institute, Niigata University*, [6]*Department of Molecular Neuroscience, Osaka University Graduate School of Medicine*, [7]*Laboratory of Neuroscience, Faculty of Medical Sciences, Jikei University of Health Care and Sciences*)

Cerebral cortices contain arrays of cortical columns, which are the fundamental units of cortical information processing. In the adult somatosensory cortex (S1), the excitatory feedforward relay is mediated by axons of layer 4 (L4) excitatory neurons projecting to L2/3 almost exclusively within the same column or home barrel column, which forms topographically precise columnar projections. Similar topographically precise columnar organization is seen throughout the neocortices of higher animals, and thus columnar projection is thought to be crucial for functions of the neocortex. However, the underlying mechanism of how such precise projections arise during development has not been intensively studied. Indeed, molecules that control this process have not yet been found. We previously showed that spike timing-dependent plasticity (STDP) contributes to neural circuit formation and axonal retraction by strengthening and weakening of synaptic connections, respectively. Endocannabinoid-dependent long-term depression (LTD) of STDP particularly plays a crucial role in the formation of barrel-specific targeting of thalamocortical axons, because it was disrupted in mice with knockout (KO) of type 1 cannabinoid receptor (CB1R) that mediates endocannabinoid signaling in the brain. These results indicate that CB1R plays an important role in fine-tuning the position of axonal projections during development. In the present study, we show that the endocannabinoid 2-arachidonoylglycerol (2-AG) plays a crucial role in shaping the columnar organization. Neurobiotin was injected into L4 spiny stellate cells using patch electrodes in slice preparations, and axonal morphology was examined under confocal microscopy after paraformaldehyde fixation. In animals genetically lacking diacylglycerol lipase α (DGLα), the major enzyme for 2-AG synthesis, the columnar projection of L4 axons was collapsed. In wild-type mice, projections were less organized until P12 and then became columnar after CB1R) became functional at L4 axon terminals. In contrast, this developmental change did not occur in DGLα knockout mice. Intraperitoneally administered CB1R agonists shortened axon length, whereas single-neuron knockout of CB1R by electroporation-mediated Cre expression in L4 spiny stellate cells of CB1R-floxed mice impaired its columnar projection. Our results suggest that endocannabinoid signaling is crucial for shaping columnar projection in the cerebral cortex.

### [1P04-09]

### The elimination of cortico-motoneuronal synapses during development: involvement of GluN2B containing NMDA receptor

*Takae Ohno[1], Satoshi Fukuda[1], Naoyuki Murabe[1], Kenji Sakimura[2], Toshhiro Hayashi[1], Masaki Sakurai[1] ([1]*Teikyo Univ.*, [2]*Niigata Univ.*)

We previously showed in our *in vitro* model of the corticospinal projections that corticospinal axons extend over the spinal gray matter during early stages of development but later regress from the ventral side. This elimination was further found to be dependent on postsynaptic GluN2B-containing NMDA receptors (2B). We then found that corticospinal axons make transient direct connections with motoneurons at an early postnatal stage in rodents by recording monosynaptic corticomotoneuronal EPSCs, which had been believed to be present only in higher primates. We studied the time course of positive ratio of the cortico-motoneuronal direct connections, which decreased from P14 toward P21 and disappeared thereafter. Here we studied the involvement of postsynaptic 2B on the corticomotoneuronal synapse elimination. When 2B gene was conditionally knocked out from the forearm motoneurons using 2B-floxed mice and Cre-loxP system, 2B component of NMDA currents were reduced to about 15% in 2B-KO motoneurons while those of WT were more than 80%. We then examined the developmental change of the positive ratio in 2B-KO motoneurons. Direct connections were present in about 30 % of 2B-KO MNs even after P21 and preserved at least until P48. These results suggest that the corticomotoneuronal synapse elimination is dependent at least partly on post-synaptic GluN2B-containing NMDA receptors.

### [1P04-10]

### Hippocampal CA1 spike activity and sharp-wave ripples related to reward prediction and acquisition

*Tomomi Sakairi[1], Masanori Kawabata[1], Alain Rios[1], Satoshi Kaneko[1], Yutaka Sakai[2], Yoshikazu Isomura[1] ([1]*Tokyo Medical and Dental University*, [2]*Tamagawa University Brain Science Institute*)

The hippocampus stores episodic experiences, which may be useful for predicting what outcome comes from an action in operant learning. However, it remains unclear how hippocampal neurons predict and respond to the outcome of the action. Here, we examined rat hippocampal CA1 activity related to outcome (reward) prediction and acquisition during a reward-alternation task under the head-fixed condition. In this task, Rats must release a pedal in response to a Go cue to obtain reward water. As the reward was given every other trial, they predicted reward or no reward prior to the action in each trial. We found hippocampal neurons activated in response to the reward delivery as well as before/around the Go cue presentation. Thus, they were involved in action preparation/ execution and reward acquisition. We also analyzed sharp-wave ripples (SWRs) in local field potential nearby those neurons. They occurred ahead of the Go cue presentation and after the cue of rewarding or not. Interestingly, the former was enhanced in the rewardpredictable condition, whereas the latter was rather inhibited in the presence of reward delivery. It means the discrepancy of reward responsiveness between the neuronal activity and SWRs.

### Poster Presentation 1

### [1P05]
### Neural network, Neurons, Synapses

March 16(Wed), 12:30 - 14:30, Zoom P5

### [1P05-01]
### Amygdala underlies the environment-dependency of defense responses induced via superior colliculus

*Kaoru Isa[1], Kota Tokuoka[1,2], Sakura Ikeda[1], Sara Karimi[3], Kenta Kobayashi[4], Thongchai Sooksawate[1,5], Tadashi Isa[1,6,7] ([1] *Grad. Sch. Med., Kyoto University,* [2] *Graduate School of Biostudies, Kyoto University,* [3] *Institute for Basic Sciences, Physiology Research Center, Kashan University of Medical Sciences,* [4] *Section of Viral Vector Development, National Institute for Physiological Sciences,* [5] *Department of Pharmacology and Physiology, Faculty of Pharmaceutical Sciences, Chulalongkorn University,* [6] *Human Brain Research Center, Graduate School of Medicine, Kyoto University,* [7] *Institute for the Advanced Study of Human Biology (WPI-ASHBi), Kyoto University*)

In our previous study, we showed that the defense responses induced by the selective optogenetic activation of the uncrossed output pathway from the deeper layer of the superior colliculus were environment-dependent in the mouse. In a small closed box, the stimulus frequently induced flight (fast forward run-away) responses, while in a large open field, the stimulus tended to induce backward retreat responses. We tested a hypothesis that the amygdala is involved in such environment-dependency of the innate defense responses. For this purpose, we made a bilateral lesion of the amygdala with ibotenic acid injections in male mice. As a result, in the mice with lesions of substantial portions of the basolateral and basomedial complex, the flight responses in the closed box disappeared and retreat responses were mainly induced. The retreat responses on the open platform were unchanged. Classically, the amygdala has been considered to be involved in the memory-dependent contextual modulation of the fear responses. In contrast, the present results suggest a novel view on the role of the amygdala in which the amygdala plays a key role in sensing the current environmental setting for making a quick decision of action upon emergency, which is critical for survival in the natural environment.

### [1P05-02]
### Reorganization of functional connectivity between cortical areas during functional recovery after ischemic stroke

*Seiichiro Sakai[1], Riina Ishiwatari[1], Takashi Shichita[1] ([1] *Tokyo Metropolitan Institute of Medical Science*)

Ischemic stroke causes neuronal cell death and destruction of neural circuits, resulting in impairment of motor, sensory and higher brain function. It has been thought that neurological functions are restored by the reorganization of neuronal circuits in the normal brain tissue remaining after stroke. However, the detail structure of the reconstructed neuronal circuits has not been elucidated. In the present study, we analyzed the remodeling of functional connectivity among cortical areas after ischemic stroke in mice. Focal ischemic stroke was induced by photothrombosis in the hindlimb region of primary somatosensory cortex or the forelimb region of primary motor cortex. Wide-field calcium imaging was performed in a mouse strain expressing GCaMP6f in excitatory neurons of neocortex for 8 weeks after stroke onset. Functional connectivity was calculated from the correlation of spontaneous activity under isoflurane anesthesia. The functional connectivity of large cortical areas was decreased one weeks after stroke and recovered 2-3 weeks after stroke onset. We then tested whether this increase in functional connectivity could be facilitated by the forced use of the impaired limb. The results showed that the forced limb use improved the recovery of functional connectivity and neurological function. These results indicate that reorganization of neuronal circuit occur in a large cortical area after ischemic stroke and that the neuronal circuit reorganization is activity-dependent.

### [1P05-03]
### Whole-hemisphere electrocorticography (ECoG) recording in the common marmoset performing visually-guided saccade task

*Kuan Ting Ho[1] ([1] *Kyoto University*)

Saccades are ballistic eye movements that demonstrate the elegance of our motor control system. Although the subcortical pathways for saccade control have been well documented, the visuomotor transformation across cortical areas has not been studied well. The current study takes advantage of the lissencephalic cortex of marmosets, which can be well covered by whole-hemispheric ECoG arrays to address this issue. During the visually-guided saccade task, the marmoset was first trained to fixate on a central stimulus for 250 ms. Then the marmoset had to make a saccade to a peripheral stimulus simultaneously as the offset of the central stimulus and keep fixating for 250 ms. In another condition, there was a 150-ms gap between the disappearance of the central stimulus and the appearance of the peripheral stimulus, during which the marmoset was required to keep fixation on where the central stimulus was. The results indicate a global inhibition of alpha band activity throughout the whole task period, and activation of multiple bands that shows target location selectivity not only in visual and motor areas, but also in other sensory and associative areas in particular phases of the task. Further analysis shows the propagation of bandwidth-specific activities across wide cortical areas, which may deepen our understanding of how cortical areas interact with each other and contribute to generation of saccades.

### [1P05-04]
### Distinct classes of projection neurons in anterior cingulate cortex differently encode spatial and reward information

*Yaolong Li[1], Kotaro Mizuta[1], Yasunori Hayashi[1], Alexander Schmidt[1,2] ([1] *Kyoto University,* [2] *Max Planck Institute for Dynamics and Self-Organization*)

The anterior cingulate cortex (ACC) plays important roles in multiple neuronal tasks such as decision making and spatial navigation. It sends projection to multiple functionally different brain regions such as dorsal-medial striatum (dmSTR), which relates to decision making and retrosplenial cortex (RSC), which is important in spatial navigation. It raises the question how different information is represented in ACC and projected to other brain regions. To address this question, we first retrogradely labeled the projection neurons in mouse ACC by introducing retrograde tracers into target areas including RSC and dmSTR. We found that ACC layer 2/3 neurons preferentially project to dmSTR, whereas layer 5 neurons preferentially project to RSC. To check whether these distinct projection neurons are functionally different, we injected AAV2-retro-CaMKII-GCaMP6f into RSC and dmSTR separately, and recorded activities of projection neurons during learning in the linear and square tracks in virtual reality under two-photon microscopic imaging. We found place cells in both ACC-RSC and ACC-dmSTR projection neurons. ACC-RSC place cells were highly enriched around reward location, whereas ACC-dmSTR place cells covered entire tracks. These results indicate that spatial and reward information might be encoded by different ACC neurons and sent to different brain region.

### [1P05-05]
### Graded sign inversion via inhibitory networks in cerebellar cortex

*Kaoru Beppu[1,2], Beverley Clark[1], Sara Rieubland[1], Charlotte Arlt[1], Arnd Roth[1], Michael Häusser[1] ([1] *Wolfson Institute of Biomedical Research, University College London,* [2] *Department of Physiology, Tohoku University School of Medicine*)

Inhibitory pathways are essential elements of the nervous system and the interaction between excitation and inhibition underlies fundamental operations performed by neuronal circuits. Inhibitory interneurons are connected to each other via chemical and/or electrical synapses. Understanding how combinations of these connections in inhibitory circuits shape network activity will provide fundamental insight into the impact of functional diversity in inhibitory connectivity. In the cerebellar cortex, the activity of Purkinje cells (PCs), the sole output neurons, is regulated by synaptic inhibition from molecular layer interneurons (MLIs). Paired recordings from MLIs and PCs in cerebellar slices showed not only the expected spike-triggered inhibitory connection but also an unexpected effect: a hyperpolarization of the MLI caused a depolarization of the PC. Triple recordings revealed that this was mediated through electrical coupling within the MLI network. Importantly, this disinhibition was both graded and bidirectional. To assess the impact of this effect on PC spiking, MLI membrane potential was modulated by injection of a subthreshold sinusoidal current. PC firing was sinusoidally and symmetrically modulated in phase with MLI membrane potential. These results help us understand general principles about the output of interneuron networks and how is inhibition delivered to principal cells at a network level.

### [1P05-06]

### Changes in action potential properties of striatal cholinergic interneurons in aged mice

*Etsuko Suzuki[1], Toshihiko Momiyama[1] ([1]*Jikei University, Sch. of Med. Dept. Pharmacology*)

A whole-cell patch-clamp study was carried out to investigate changes in firing properties of striatal cholinergic neurons during aging. Brain slices were prepared from 2-3-week-old, 5-6-week-old, 2-3-month-old, 6-7-month-old, 11-12-month-old and 16-18-month-old mice of either sex. The present results have shown that requency of spontaneous firing was low at 2-3 weeks of age (0.28 ± 0.04 Hz, n = 4) and increased significantly during postnatal development (11-12-month-old: 7.68 ± 1.64 Hz, n = 8, p = 0.016). The threshold of action potential was significantly increased at 16-18 months of age (-34.8 ± 2.13 mV, n = 5, p = 0.022 compared with 6-7-month-old: -42.9 ± 1.56 mV, n = 8). The rise time of action potential at 16-18 months of age (0.39 ± 0.05 ms) was significantly longer than that of at 2-3-month-old of age (0.26 ± 0.02 ms, p = 0.034). On the other hand, there were no differences in the amplitude of afterhyperpolarization or the sag ratio. These findings suggest prominent changes in firing properties of striatal cholinergic interneurons with aging.

### [1P05-07]

### Optical analysis of spontaneous oscillatory activity in the absence of external $Ca^{2+}$ observed in the embryonic chick cerebellum

*Katsushige Sato[1], Nana Takahashi[1], Yoko Momose-Sato[2] ([1]*Department of Health and Nutrition Sciences, Faculty of Human Health, Komazawa Women's University*, [2]*Department of Nutrition and Dietetics, College of Nutrition, Kanto Gakuin University*)

We applied 1020-site optical recording with a voltage-sensitive dye (NK2761, a merocyanine-rhodanine dye) to the embryonic 10 day brainstem-cerebellum preparation to examine functional organization of the cerebellum during embryogenesis. In normal physiological solution, electrical stimulation to the cerebellar peduncle elicited fast spikelike signals (corresponding to the action potential) followed by delayed long-lasting slow signals (corresponding to the excitatory postsynaptic potential (EPSP)) in the cerebellar hemisphere. When we replaced calcium ions in the external solution to magnesium ions, the slow signals were completely eliminated. In this condition, spontaneous oscillatory activity newly appeared near the midregion of the cerebellum. The incidence of the spontaneous oscillatory activity was dependent on the concentration of the external calcium ion, and the frequency was different between regions. We examined spatiotemporal patterns of the oscillatory activity by making pseudo-color images of the optical signal.

### [1P05-08]

### *In vivo* calcium imaging reveals dynamic neuronal glucose-sensing in the ventromedial hypothalamus

*Ming-Liang Lee[1,2,3], Ching Pu Chang[1,2,4], Tomomi Nemoto[1,2], Ryosuke Enoki[1,2] ([1]*National Institute for Physiological Sciences*, [2]*The Exploratory Research Center on Life and Living Systems*, [3]*JSPS International Research Fellow*, [4]*Graduate school of medicine, Hokkaido University*)

The ventromedial hypothalamus (VMH) is one of the core regions to regulate systemic glucose metabolism to maintain energy homeostasis. It is believed that the VMH detects systemic glucose levels by glucose-sensing (GS) neurons, which can be either excited or inhibited by glucose, called glucose-excited (GE) or glucose-inhibited (GI) neurons, respectively. Although neuronal GS has been well studied in acute slices and cell culture, the characteristics of hypothalamic GS neurons *in vivo* are still unknown. Here, we successfully imaged calcium activities of GS neurons in the VMH with UCLA miniscope. We found that the populations of GS neurons are different between *ab libtum* and fasted mice. Overnight fasting decreases the number of GE neurons and increases that of GI neurons, suggesting body energy status can organize the population of GS neurons. Moreover, repeated recording in the same mice showed the distribution of GS neurons is dynamic with constant population sizes of each type of GS neurons. Together, these results suggest neuronal glucose-sensing function is dynamic in the VMH to adapt to different energy statuses and environmental situations.

### [1P05-09]

### Function of metabotropic glutamate receptor 1 in the neonatal hippocampal marginal zone.

*Megumi Taketo[1] ([1]*Kansai Medical Univ.*)

Metabotropic glutamate receptors (mGluRs) are G-protein-coupled receptors that modulate neural excitability and synaptic transmission. Group I mGluRs consisting of mGluR1 and mGluR5, mainly couple to $G_{q/11}$ proteins and increase intracellular $Ca^{2+}$ concentration ($[Ca^{2+}]_i$). Group I receptors also regulate several channels and other signaling proteins. Cajal-Retzius (CR) cells in hippocampal marginal zone control neural migration by production and secretion of glycoprotein reelin. In addition to instruction of migration, CR cells also project their dendrites to other neurons and modulate network activity. In the present experiments, it was demonstrated that mGluR1 is functionally expressed by hippocampal CR cells; selective activation of mGluR1 by co-application of a group I mGluR agonist and a mGluR5 antagonist results in $[Ca^{2+}]_i$ elevation. The effects of ionotropic glutamate receptors and a $GABA_A$ receptor on $[Ca^{2+}]_i$ were also measured and compared with the mGluR1-mediated $Ca^{2+}$ elevation. In addition, possible interaction between mGluR1 and other receptors and effect of mGluR1 activation on membrane excitability of C-R cells were determined.

### [1P05-10]

### Occlusal Disharmony Decreases Cognition via Cognitive Suppressor Molecules

*Munehisa Maeshiba[1,2], Hiroshi Kajiya[1,3], Takashi Tsutsumi[4], Kazuko Goto-T[5], Fujio Okamoto[3], Koji Okabe[3], Takashi Tsuzuki[2], Jun Ohno[1] ([1]*Oral Med Centr, Fukuoka Dent Coll*, [2]*Dep Oral Grow Develop, Fukuoka Dent Coll*, [3]*Dep Physiol Sci Mol Biol, Fukuoka Dent Coll*, [4]*Dep Visiting Dentistry Center, Fukuoka Dent Coll*, [5]*Dep Dent Hygi, Fukuoka Coll Health Sci*)

**Background** Occlusal disharmony has been reported to be affected not only by cytokine and steroid hormone secretion and sympathetic activation in peripheral organs, but also by neurotransmitter release in the central nervous system. However, little is known about whether occlusal disharmony can decrease cognitive ability. We hypothesized that hyperocclusion decreases cognition via Alzheimer's diseaseassociated molecule expression in the brain. The present study is aimed to elucidate the relationships among occlusal disharmony, cytokine and cognitive-regulated molecule expression in the brain, and the impairment of learning and memory cognition.

**Materials and Methods** We examined the effect of hyperocclusion on the relationships among cytokine expression, cognitive suppressor molecules in the hippocampus, and cognition in behavior using Western blotting, q-PCR, and immunostaining in a mouse model of hyperocclusion.

**Results and Discussion** Hyperocclusion dramatically increased interleukin-1b expression in the serum and hippocampus 1 week after hyperocclusal loading in 2-month-old mice, but no effects were observed in 12-month-old mice. The expression levels of amyloid-β (1-42) and phosphorylated tau were significantly upregulated 1 week after hyperocclusal loading in the hippocampus of 2-month-old mice but were constant in 12-month-old mice. The social and long-term cognitive abilities of the 2-month-old mice were transiently downregulated close to the level of the 12-month-old mice 1 week after hyperocclusion and recovered to close to basal level via the expression of cognitive suppressor clearing proteins.

**Conclusion** Occlusal disharmony-induced interleukin-1β expression may contribute to accumulation of cognitive suppressor molecules such as amyloid-β and phosphorylated tau and activate their clearance proteins, resulting in protection against transient dementia in young but not older mice. No potential conflicts of interest that should be declared.

### Poster Presentation 1

### [1P06]
### Neurons, Synapses

March 16(Wed), 12:30 - 14:30, Zoom P6

### [1P06-01]
### Ghrelin enhances GABAergic transmission on cerebellar Purkinje cells

*Moritoshi Hirono[1], Ayaka Matsushita[1], Miku Suzuki[1], Miyawaki Riku[1], Yuta Yao[1], Masanori Nakata[1] ([1]*Dept Physiol, Wakayama Med Univ* )

Ghrelin is an orexigenic peptide and an endogenous ligand for growth hormone secretagogue receptor 1a (GHS-R1a). Ghrelin is distributed not only in the stomach but also in the brain. A recent study reported that rodent cerebellar Purkinje cells (PCs) express GHS-R1a and its activation facilitates spontaneous firing of PCs. However, little is known about whether ghrelin alters GABAergic transmission on PCs and modulates firing of PCs. We examined effects of ghrelin on the GABAergic transmission and spontaneous firing of molecular layer interneurons (MLIs) using patch clamp recordings applied to mouse cerebellar slices. We found that ghrelin increased significantly the frequency and amplitude of spontaneous inhibitory postsynaptic currents (IPSCs) in PCs. The peptide did not alter miniature IPSCs in PCs, whereas the peptide caused an increase in the firing rate of MLIs. The ghrelin-mediated effect on MLIs was blocked by a GHS-R1a antagonist JMV3002, suggesting that GHS-R1a is also expressed in MLIs. These results indicate that the ghrelininduced potentiation of spontaneous IPSCs involves presynaptic mechanisms underlying the facilitation of action potential induction most likely in somatodendritic sites of MLIs. Thus, ghrelin regulates firing of PCs directly and indirectly, and could contribute to fine tuning of motor coordination.
(COI:NO)

### [1P06-02]
### Essential Role of Somatic Kv2 Channels in High-Frequency Firing in Cartwheel Cells of the Dorsal Cochlear Nucleus

*Tomohiko Irie[1] ([1]*National Institute of Health Sciences*)

Among all voltage-gated potassium (Kv) channels, Kv2 channels are the most widely expressed in the mammalian brain. However, studying Kv2 in neurons has been challenging because of a lack of high-selective blockers. Recently, a peptide toxin, guangxitoxin-1E (GxTX), has been identified as a specific inhibitor of Kv2, thus facilitating the study of Kv2 in neurons. The mammalian dorsal cochlear nucleus (DCN) integrates auditory and somatosensory information. In the DCN, cartwheel inhibitory interneurons receive excitatory synaptic inputs from parallel fibers conveying somatosensory information. The activation of parallel fibers drives action potentials in the cartwheel cells up to 130 Hz *in vivo*, and the excitation of cartwheel cells leads to the strong inhibition of principal cells. Therefore, cartwheel cells play crucial roles in monaural sound localization and cancelling detection of self-generated sounds. However, how Kv2 controls the high-frequency firing in cartwheel cells is unknown. In this study, we performed immunofluorescence labeling with anti-Kv2.1 and anti-Kv2.2 antibodies using fixed mouse brainstem slice preparations. The results revealed that Kv2.1 and Kv2.2 were largely present on the cartwheel cell body membrane but not on the axon initial segment (AIS) nor the proximal dendrite. Whole-cell patch-clamp recordings using mouse brainstem slice preparation and GxTX demonstrated that blockade of Kv2 induced failure of parallel fiber-induced action potentials when parallel fibers were stimulated at high frequencies (30-100 Hz). Thus, somatic Kv2 in cartwheel cells regulates the action potentials in a frequency-dependent manner and may play important roles in the DCN function.

### [1P06-03]
### SP600125 Enhanced Neurite Outgrowth induced by Temperature-Controlled Repeated Thermal Stimulation in PC12-P1F1 Cells

*You-Ran Luo[1], Tada-aki Kudo[2], Kanako Tominami[2], Satoshi Izumi[2], Yohei Hayashi[3,4], Takuya Noguchi[5], Atsushi Matsuzawa[5], Junichi Nakai[2], Guang Hong[1] ([1]*Division for Globalization Initiative, Tohoku University Graduate School of Dentistry,* [2]*Division of Oral Physiology, Tohoku University Graduate School of Dentistry,* [3]*Cell Resource Center for Biomedical Research, Institute of Development, Aging and Cancer, Tohoku University,* [4]*Graduate School of Life Sciences, Tohoku University,* [5]*Laboratory of Health Chemistry, Graduate School of Pharmaceutical Sciences, Tohoku University*)

This study investigated the effects of a c-Jun N-terminal kinase (JNK) inhibitor SP600125 (SP), on neuronal differentiation of rat PC12-P1F1 cells, which can differentiate into neuron-like cells with a temperature-regulated thermal stimulation (TRTS) and neurotrophic factors including bone morphogenetic protein 4 (BMP4). Cells were incubated with TRTS and/or SP and neuritogenesis was evaluated. TRTS-mediated neuritogenesis was dosedependently enhanced by co-treatment of SP, although SP treatment failed to induce neuritogenesis without TRTS. Such event was not observed with other selective JNK inhibitors (AS601245, TCSJNK6o, and TCSJNK5a). To further clarify the mechanism of SP action, the effects of SP on intracellular signaling were tested with the following inhibitors: a selective extracellular signal-regulated kinase (ERK)1/2 kinase (MEK) inhibitor, U0126 and a BMP receptor inhibitor, LDN193189. SP-enhanced neuritogenesis induced by TRTS was robustly inhibited in the presence of U0126 or LDN193189. These findings suggest that SP has a potential to enhance TRTS-induced neuritogenesis, which might be related to ERK1/2 and BMP signaling.

### [1P06-04]
### Neuromedin U modulates hippocampal CA3-CA1 synaptic transmission.

*Sachie Sasaki-Hamada[1], Yoshimichi Maeno[1], Mizuki Yabe[1], Hitoshi Ishibashi[1] ([1]*Kitasato Univ.*)

Neuromedin U (NMU) is a neuropeptide that was initially isolated from the porcine spinal cord and later from several species. Although NMU receptors exist in the CA1 region of the hippocampus, the role of NMU in hippocampal synaptic transmission remains unknown. In the present study, we demonstrated that the colocalization ratio of NMU type 1 (NMUR1) or type 2 (NMUR2) receptors was higher with neuronal nuclei (a neuronal marker) than with glial fibrillary acidic protein (an astrocyte marker) in the CA1 region of rats. Moreover, we revealed that the bath application of NMU (1 mM) enhanced extracellular field excitatory postsynaptic potentials at Schaffer collateral-CA1 synapses in rat hippocampal slices (+28.9 ± 1.3 %; $P < 0.05$). After extracellular recordings, we examined the pattern of neuronal activation induced by NMU using c-Fos immunohistochemistry (Fos-IR). Histological analyses revealed that NMU increased Fos-IR in the CA1 region, but reduced the proportion of Fos-IR colocalized with glutamic acid decarboxylase (a GABA neuron marker). These results suggest that the activation of NMU receptors contributes to GABAergic neuronal activity in the CA1 region of the hippocampus.

### [1P06-05]
### Roles of glycine receptors in the hippocampus

*Shoichiro Ikeda[1], Kanaru Kimura[2], Masahiro Mori[1] ([1]*Kobe University Graduate School of Health Sciences , Faculty of Health Sciences,* [2]*Kobe University School of Medicine, Faculty of Health Sciences*)

Glycine is a major inhibitory neurotransmitter in the nervous system. In the hippocampus expression of glycine receptors and the responses by application of glycine were reported. Glycinergic synaptic transmission, however, has not been shown yet in the hippocampus. We recorded the responses by puff application of glycine (0.3 mM. 0.5-1 s) in CA3 pyramidal neurons of the cultured hippocampal slices. The glycine responses peaked within 7days after preparation of the hippocampal slice culture made from new born rats (P0-1). Then to study the roles of glycine receptors during the development of the hippocampus, we will two-dimensionally analyze several morphological parameters such as total length of dendrites and number of branching points in biocytin-labeled pyramidal neurons in the hippocampal slices cultured with strychnine (10 $\mu$M) or the normal control slices.

### [1P06-06]
### Roles of the elecrtrical synapses in spinal motoneurons
*Kanaru Kimura[1], Shoichiro Ikeda[2], Masahiro Mori[2] ([1]*Kobe University School of Medicine, Faculity of Health Sciences,* [2]*Kobe University Graduate School of Health Sciences, Faculty of Health Sciences*)

Electrical synapses are expressed in spinal motoneurons. Their expression is enhanced for several days after birth or spinal cord injury. Their roles, however, have not been well clarified yet. We recorded responses via electrical synapses in a pair of synaptically connected motoneurons of a cultured spinal cord slice. Carbenoxolone (0.1-0.3mM) did not inhibited the responses via electrical synapses. While meclofenamic acid (0.1 mM) partially inhibited the responses. To assess roles of electrical synapses in the spinal cord, spinal cord slices are to be cultured for 7 days with meclofenamic acid (0.1 mM). Then we will two-dimensionally measure several morphological parameters such as total length of dendrites and number of branching points in biocytin labeled motoneurons in the spinal cord slices cultured with meclofenamic acid or the normal control cultured slices.

### [1P06-07]
### cAMP attenuates action potential conduction in cerebellar Purkinje cell axons
*Kei Furukawa[1], Shin-ya Kawaguchi[1] ([1]*Department of Biophysics, Graduate School of Science, Kyoto University*)

An action potential is conducted in an axon to its terminals. Recent studies showed that increase in intracellular cAMP increases the action potential conduction velocity in axons of excitatory neurons, such as cerebellar mossy fibers and parallel fibers. However, it remains unknown whether and how cAMP modulates the action potential velocity in inhibitory neurons. Here, we performed simultaneous recordings from the soma and axon of a cultured Purkinje cell (PC), an inhibitory neuron in the cerebellum. Increase in intracellular cAMP was caused by application of forskolin, a cell-permeable adenylyl cyclase activator. Surprisingly, action potential propagation from a PC soma to an axon was delayed by forskolin in a manner dependent on the axon length, suggesting that cAMP decreases the action potential conduction velocity in a PC axon. Furthermore, our direct patch clamp recording from a PC axon demonstrated that forskolin attenuated action potentials. In addtion, simultaneous recordings from a PC soma and its target neuron showed that forskolin increased the synaptic delay. Thus, our results indicate a contrasting action of cAMP on the action potential propagation in inhibitory neurons compared to excitatory ones.

### [1P06-08]
### Orexin receptor activation induces a novel slow afterhyperpolarization in serotonergic dorsal raphe neurons.
*Masaru Ishibashi[1], Atsuo Fukuda[1], Christopher S. Leonard[2] ([1]*Department of Neurophysiology, Hamamatsu University School of Medicine,* [2]*Department of Physiology, New York Medical College*)

Serotonergic (5-HT) dorsal raphe (DR) neurons regulate numerous brain functions including sleep-wake states and mood. Moreover, loss of orexin signaling at 5-HT DR neurons appears critical in the sleep disorder narcolepsy. We recently reported that in addition to producing a slow depolarization, orexin-A enhances the post-spike afterhyperpolarization (oeAHP), which alters spike encoding by increasing spike frequency adaptation. Mechanistically we found that the oeAHP involved two distinct components that required $Ca^{2+}$ influx. The first was of medium-duration (tau ～ 0.5s) and involved apamin-sensitive SK $Ca^{2+}$-activated $K^+$ channels. The second was of longer duration (tau ～ 5s), was apamin-insensitive (termed the ai-oeAHP) and appeared similar to a slow AHP (sAHP). In this study we have utilized current clamp and dynamic clamp recordings in mouse brain slices to investigate the role of the ai-oeAHP in regulating 5-HT DR neuron firing. we found that the ai-oeAHP was not attenuated by a cesium-based patch solution as expected for a $K^+$ currents, but rather was blocked by substituting NMDG for $Na^+$ in the ACSF or by application of flufenamic acid (FFA), both of which attenuated the orexininduced inward current. Moreover, we found that the increase in baseline membrane conductance produced by orexin-A was reduced during the ai-oeAHP suggesting that the ai-oeAHP was mediated by a transient, $Ca^{2+}$-dependent closure of the cation channels activated by orexin. These results suggest that ai-oeAHP is a novel type of $Ca^{2+}$- dependent sAHP that is conditionally expressed following orexin-activation of non-selective cation channels.
(COI : NO)

### [1P06-09]
### Modulation of synaptic outputs in a cerebellar Purkinje cell by cannabinoid revealed by direct bouton recordings
*Takuma Inoshita[1], Shin-ya Kawaguchi[1] ([1]*Graduate School of Science, Kyoto University*)

Synaptic transmission is typically negatively controlled by cannabinoids at various synapses in the central nervous system, such as excitatory and inhibitory synapses on a cerebellar Purkinje cell (PC). Based on the previous indirect $Ca^{2+}$ imaging at axon terminals during action potentials, $Ca^{2+}$ influx into a presynaptic terminal has been thought to be reduced by cannabinoids, causing a reduction in transmitter release. To test whether the negative control of transmission by cannabinoids is common to a variety of synapses, and if so, to directly examine the underlying mechanism, we performed direct patch-clamp recordings from axon terminals of cultured PCs. Among subtypes of cannabinoid receptors, a PC has been shown to predominantly express cannabinoid receptors type 2 (CB2Rs), but not cannabinoid receptors type 1 (CB1Rs). Neither the presynaptic $Ca^{2+}$ current in a PC axon terminal nor synaptic transmission from a PC bouton was affected by pharmacological activation of CB1Rs and/or CB2Rs, in line with the previous slice patchclamp studies and the lack of both in a PC bouton shown by immunofluorescent staining. Alternatively, surprisingly, we found that atypical type of endocannabinoid receptor other than CB1Rs and CB2Rs clearly suppressed synaptic outputs from a PC bouton without affecting presynaptic $Ca^{2+}$ influx. Taken all these results together, our data demonstrate that there is a novel regulatory mechanism of synaptic transmission by cannabinoids, which is distinct from the reduction of presynaptic $Ca^{2+}$ influx by CB1Rs and/or CB2Rs.

### [1P06-10]
### Probing glutamate release sites at the ribbon-type synapses in the goldfish retinal bipolar cell terminal
*Tomoko Oshima-Takago[1,2], Hirokazu Sakamoto[2], Yukihiro Nakamura[2,3], Shigeyuki Namiki[2], Kenzo Hirose[2], Masao Tachibana[1,2,4], Hideki Takago[1,2] ([1]*Dept. of Rehabilitation for Sensory Functions, Research Inst., Nat'l Rehabilitation Ctr. for Persons with Disabilities,* [2]*Dept. of Pharmacol, Grad. Sch. of Med, the Univ. of Tokyo,* [3]*Dept. of Pharmacol, Jikei Univ. Sch. of Med.,* [4]*Ctr. for Systems Vision Science, Organization of Science and Technology, Ritsumeikan Univ.*)

The ribbon-type synapses in the retinal, cochlear, and vestibular organs enables continuous processing of sensory information utilizing $Ca^{2+}$-driven glutamatergic neurotransmission. These sensory cells exhibit kinetically separate components of glutamate release upon depolarization: the fast and slow components of evoked release. Notably, previous research using FM dye for visualizing synaptic vesicle fusion on TIRF microscopy indicates that the ribbon-associated and ribbon-free active zones in the retinal bipolar cell terminal may underlie the fast and slow components of evoked release, respectively. This hypothesis remains to be further tested ideally by visualizing the spatiotemporal dynamics of glutamate release from individual active zones in relation to the location of the synaptic ribbons. Using a retinal bipolar cell-targeted type of enhanced glutamate optical sensor (BC-eEOS), we find heterogeneous nature of the fast and slow components of glutamate release: the fast component of evoked release occurs predominantly at ribbon-associated sites and shows variability in its strength, whereas the slow component of evoked release occurs mainly at ribbon-free sites. The glutamate imaging using the BC-eEOS provides a novel platform to optically analyze spatiotemporal dynamics of glutamate release from individual active zones in the retinal bipolar cell terminal.
The authors declare no COIs.

### [1P06-11]
### Social stress-induced remodeling of neuronal circuits in the sensory thalamus.
*Hisako Nakayama[1], Mariko Miyata[1] ([1]*Tokyo Women's Medical University*)

It is familiar for us that mental stress induces abnormality of sensation, including hyperesthesia and hypoesthesia. However, the underlying neuronal mechanisms are almost unknown. In mice, tactile information from whiskers is sent to VPM neurons in the thalamus through medial lemniscus fibers (MLF). Most VPM neurons receive strong excitatory synaptic input from one MLF (mono-innervation) in mice after weaning age, while multiple MLFs (multiple-innervation) in the early postnatal stage. This mono-innervation is considered to maintain throughout life so far. We investigated how social stress affects the wiring patterns at MLF-VPM synapses with electrophysiological methods. Mice were reared in group-housed or socially-isolated conditions for four weeks after weaning of postnatal day 21. We found multiple-innervation at MLF-VPM synapses reappeared in sociallyisolated mice. The multiple-innervation was also induced in group-housed mice administered corticosterone orally by mixing with the drinking water. Interestingly, multipleinnervation was not induced when social isolation started after sexual maturation (two-month-old). These results suggest that MLF-VPM synapses are susceptible to social stress, exceptionally from weaning to sexual maturation.

### Poster Presentation 1

### [1P07]
### Autonomic nervous system, Others

March 16(Wed), 12:30 - 14:30, Zoom P7

### [1P07-01]
**Starburst amacrine cells in the early postnatal development form gap junctions**

*Takuma Maruyama[1], Toshiyuki Ishii[1], Sumiko Usui[1], Masumi Shimizu[1], Makoto Kaneda[1] ([1]*Nippon Medical School*)

In the central nervous system, neuronal networks via gap junctions are lost during postnatal development. On the other hand, in the retina, several types of neurons have known to use gap junctional coupling for signal processing in adulthood. Currently, starburst amacrine cells (SACs) are recognized as a neuron which does not form gap junctional coupling in adulthood. However, it remains unclear whether SACs form gap junctional coupling during the developmental stage. In the present study, therefore, we examined whether SACs form gap junctional coupling in developmental stage in the mouse retina. When we injected Neurobiotin into SACs, many tracer-coupled cells were detected before eye-opening. The tracer coupled cells were RBPMS-positive retinal ganglion cells, but not SACs. Number of the tracer coupled cells was significantly reduced after eye-opening. At the mRNA level, connexin 43 expression before eye opening was significantly higher than that after eye opening in SAC. An application of MFA, a gap junction blocker including connexin 43, to SACs resulted in a significant decrease in membrane capacitance, an indicator of gap junctional coupling, of SACs before eye opening but not after eye opening. Dark rearing did not change the extinction process of gap junctions during development in SAC. These results suggest that SACs form gap junctions in early postnatal period, and inherently reduce the connection to neighboring cells during the development.

### [1P07-02]
**Relationship between salivary cortisol and wet wipe materials in childcare**

*Miho Fukumoto[2], Hiroki Mori[2], Daisuke Miyake[2], Yuichiro Kikuno[1] ([1]*The University of Shimane*, [2]*Unicharm Corporation*)

"Changing diapers" is known as one of the main psychological stress factor of childcare. In particular, most baby wipes used during diaper changes are thin sheets, and it is easy to have anxiety about excrement soaking through the sheet and sticking to hands. In addition to the thickness of the sheet, the present study also focused on the stiffness of the sheet. The stiffness of the sheet was targeted because the stiffness allows the sheet to spread out when it is taken out of the packaging bag, and the sheet can be unfolded and wiped off without twisting, which may lower the psychological stress level.In order to quantify the effect of the different physical properties of the wipes on the alleviation of childcare stress, we divided the participants into two groups (inter-participant design) and instructed them to use either thin type (conventional product) or the improved product (1. thicker and 2. stiffer sheets). The results showed that after 4 weeks of use, the improved product showed a reduction in cortisol (stress) levels compared to the thin type. The present study suggest that the thickness and stiffness of the sheet may be related to stress levels in human.

### [1P07-03]
**Examination of environmental factors that promote the extinction of contextual fear memory in mice**

*Hiroshi Ueno[1], Kana Tomimoto[1], Yu Takahashi[2], Motoi Okamoto[3], Takeshi Ishihara[2] ([1]*KawasakiUniversity of Medical Welfare*, [2]*Kawasaki Medical School*, [3]*Okayama University*)

Fear is a protective reaction that occurs when people cannot escape harmful and dangerous situations. Fear memory is an instinctive life phenomenon that teaches living organisms to predict danger. Excessive fear experiences and chronic stress are factors of post-traumatic stress disorder (PTSD). One of the treatments for PTSD is continuous exposure therapy. Mice have a similar fear reactions as humans. In this study, we evaluated the conditions promoting the extinction of fear memory in mice. The mice were stimulated with environmental factors, and we observed how they promoted the extinction of fear memory. Our findings revealed that the mice memorized contextual fear and the freezing time decreased by placing the mice in the test box repeatedly. Moreover, we found that decreasing freezing time and extinction of fear memory are promoted by different environmental factors. The results of this research revealed that in continuous exposure therapy, the extinction of fear memory can be promoted by different environmental factors when recollecting past fear experiences or revisiting the place and repeating experiences.

### [1P07-04]
**Site-specific autonomic vasomotor responses and their interactions in rat gingiva**

*Yunosuke Okada[1], Toshiya Sato[2], Masato Saitoh[1], Hisayoshi Ishii[2] ([1]*Div. Pediatric., Dept. Sch. Dent., Health Sci Univ.*, [2]*Div. Physiol., Dept. Sch. Dent., Health Sci Univ.*)

Blood flow (BF) in the gingiva, which consists of interdental papilla (IPBF) and attached (AGBF) and marginal gingiva (MGBF), is important in the maintenance of gingival function. Marked BF changes mediated by the autonomic nervous system may be important for gingival hemodynamics. However, differences in autonomic vasomotor responses in different parts of the gingiva are unclear. We examined differences in autonomic vasomotor responses and their interactions in the gingiva of anesthetized rats. Electrical stimulation of the central cut end of the lingual nerve (LN) elicited frequency-dependent increases in IPBF, AGBF, and MGBF, with the increases being greatest in the IPBF. The increases evoked by LN stimulation were greatly reduced by hexamethonium (90%) and atropine (50%). Activation of the superior cervical sympathetic trunk decreased the gingival BF and significantly inhibited LN stimulation-induced BF increases. Our results indicate that parasympathetic reflex vasodilation evoked by trigeminal afferent inputs are more involved in the regulation of IPBF than AGBF or MGBF and that cholinergic and noncholinergic mechanisms may contribute to the responses.

### [1P07-05]
**Electrical stimulation to the lateral habenula evokes the cardiovascular response via multiple subtypes of dopamine receptors.**

*Yuma Sato[1,2], Tri Doan[1], Masayuki Matsumoto[3,4], Tadachika Koganezawa[1,4] ([1]*Dept Physiol, Fac Med, Univ Tsukuba*, [2]*Dr Prog Med Sci, Grad Sch Comp Human Sci, Univ Tsukuba*, [3]*Dept Cogn Behav Neurosci, Fac Med, Univ Tsukuba*, [4]*Transborder Med Res Ctr, Univ Tsukuba*)

The lateral habenula (LHb) is a small brain structure involved in behavioral responses to stress events. Midbrain dopaminergic system, which receives stress information from the LHb, is likely to mediate the behavior and autonomic responses. Although the behavioral change to cope with stress accompanies autonomic cardiovascular responses, it is little known how the LHb-dopaminergic system modulates the cardiovascular responses evoked by aversive events. Our study aimed to examine the role of autonomic cardiovascular responses induced by the activation of the LHb with the dopaminergic system regulated by the activity of the LHb. We performed electrical stimulations to the LHb of anesthetized Wistar male rats with recording heart rate and blood pressure. The electrical stimulation to the LHb caused bradycardia and a pressor response. To elucidate the involvement of dopamine receptor subtypes, we stimulated the LHb with the application of the following selective dopamine receptor antagonists: SCH23390, a D1 and D5 receptors antagonist; sulpiride, a D2 and D3 receptors antagonist; L-741626, a D2 receptor antagonist; L-745870, a D4 receptor antagonist. D2 antagonist attenuated bradycardia and enhanced the pressor response. The other antagonists depressed the pressor response but did not affect the heart rate response. These results suggested that multiple subtypes of dopaminergic receptors mediate the generation of the LHb-activated cardiovascular responses to stress events.

### [1P07-06]

### Do the responses of autonomic nervous activity during listening time differ depending on the progression of music?

*Junko Hoshi[1], Konosuke Sasaki[2], Shiori Yoshida[2], Fumiko Sato[2], Ryoko Maruyama[3,2] ([1]*Akamon College of Sendai,* [2]*Tohoku University Graduate School of Medicine,* [3]*Dokkyo Medical Uninersity*)

We evaluated temporal changes of heart rate and autonomic nervous system activity in healthy adults listening to music. Forty volunteers participated in three tasks consisting of three experimental conditions: 10 min of rest and test-to-load on sympathetic nervous activity while listening to Mozart' s Piano Sonata for Two Hands, D-major (K448); J. S. Bach's Brandenburg Concerto No. 4, G-major (BWV1049); or silence as the control for 8 min in a randomized order. Electrocardiography was continuously recorded from the start to the end of each data collection point. Low frequency (LF) / high frequency (HF) ratio increased significantly from rest to load in the control silence and BWV1049 listening groups, and decreased substantially from load to listening; LF/HF ratio increased significantly from rest to load in the K448 listening group but did not show significant change from load to listening. There was no significant difference over time among the three groups. Our findings suggest that temporal changes in responses while listening to music depended on the progression of the music being listened to. COI: The work was supported by JPSS KAKENHI Grant Number JP20K23214.

### [1P07-07]

### Involvement of the central nucleus of the amygdala in the responses of arterial pressure to noxious mechanical stimulation of the hindpaw in anesthetized rats

*Hana Yano[1], Hideshi Shibata[2], Mieko Kurosawa[3] ([1]*Dept. Occupational Ther., Intl. Univ. Health & Welfare,* [2]*Lab. Vet. Anat., Ins. Agric., Tokyo Univ. Agric & Tech.,* [3]*Bio-lab., Foundation Adv. Intl. Sci.* )

Noxious mechanical stimulation (pinching) of the hindpaw reflexively increases arterial pressure via the supraspinal structure in anesthetized rats. However, the neural mechanism of the brain in the reflex responses is not well understood. Recently we focused on the involvement of the lateral parabrachial nucleus (LPBN), and clarified that the reflexes are partly mediated via the LPBN, and that the involvement of the LPBN is prominent in the contralateral stimulus. The present study focused on the involvement of the central nucleus of the amygdala (CeA), to which neurons in the LPBN directly project, with special reference to stimulus laterality. Muscimol, a widely used neuronal inhibitor, was nanoinjected into the unilateral CeA. Administration of muscimol into the CeA had no influence on the tonic arterial pressure. On the other hand, the pressor responses to pinching of the hindpaw were significantly attenuated after administration of muscimol. The degrees of attenuation in the responses were not different between pinching of contralateral and that of ipsilateral to the site of muscimol injection. The present results demonstrate that the CeA is involved in the pressor reflex responses elicited by pinching of the hindpaw, irrespective of the stimulus laterality. The finding suggests that the spinal- LPBN-CeA pathway, which dominantly conveys contralateral stimulus inputs, is not the sole pathway to the CeA involved in the somato-pressor reflexes.

### [1P07-08]

### Simultaneous measurement of vagal and sympathetic nerve activity in conscious rats

*Kenju Miki[1], Wakana Miyaura[1], Shizuka Ikegame[1], Misa Yoshimoto[1] ([1]*Autonomic Physiology, Human Life and Environment, Nara Women's University*)

Autonomic nervous system activity is thought to be caused by the balance of sympathetic and parasympathetic nerve activity. Although sympathetic nerve activity has been extensively measured, few attempts have been made to directly measure parasympathetic nerve activity. In this study, we attempted to measure both cervical vagal nerve activity and renal sympathetic nerve activity in conscious, freely moving rats. Under anesthesia, the right cervical vagus nerve of male Wistar rats was dissected approximately 1 mm, and a bipolar electrode made of twisted stainless-steel wire was hooked onto the vagus nerve and fixed with silicone gel. A microcatheter was placed at least 5 mm peripherally from the electrode to administer a long-acting local anesthetic (levobupivacaine hydrochloride 0.75%). At peak-to-peak potential, the right cervical vagal nerve activity (rcVNA) showed continuous activity of approximately 20 μV. The rcVNA was reduced by approximately 80% after 30 μL of levobupivacaine was administered via the microcatheter. This allowed us to selectively measure the efferent rcVNA while blocking the afferent rcVNA. Using this method, we discovered that efferent rcVNA and renal sympathetic nerve activity decreased during REM sleep compared to NREM sleep. Instability in circulatory regulation has been reported during the REM phase of sleep, which may be due to suppression of both rcVNA and renal sympathetic nerve activity.

### [1P07-09]

### Response of sympathetic nerves activity by activating endogenous arginine vasopressin neurons in conscious transgenic rats with DREADDs system

*Misa Yoshimoto[1], Kaori Takai[1], Juri Takei[1], Wakana Miyaura[1], Natumi Morimoto[1], Shizuka Ikegame[1], Takashi Maruyama[2], Youichi Ueta[2], Kenju Miki[1] ([1]*Autonomic Physiology, Human Life and Environment, Nara Women's University,* [2]*Department of Physiology, School of Medicine, University of Occupational and Environmental Health*)

The paraventricular nucleus (PVN) of the hypothalamus regulates arginine vasopressin (AVP) secretion and sympathetic nerve activity (SNA), thus playing a key role in maintaining body fluid and electrolyte homeostasis. In this study, the AVP magnocellular neurons were activated directly in AVP-hM3Dq-mCherry transgenic rats, and the renal and lumbar SNA responses and cardiovascular functions were studied. At least one day before the experiment, electrodes for measurements of EEG, EMG, ECG, renal SNA (RSNA), and lumbar SNA (LSNA), catheters for arterial pressure measurement and intraperitoneal clozapine-N-oxide (CNO) administration, and thermocouples for intra-abdominal temperature measurement were chronically implanted under anesthesia. The RSNA decreased by CNO administration, while LSNA did not change significantly. Thereafter, the arterial pressure gradually increased, and concomitantly, heart rate decreased. The decrease in the RSNA preceded the increase in arterial pressure suggesting that direct stimulation of AVP neurons may suppress RSNA dominantly and promote sodium excretion via the kidneys.

### [1P07-10]

### How fear memory recall affects hypothalamic paraventricular nucleus neuronal activity and sympathetic nerve activity in conscious rats

*Shizuka Ikegame[1], Kenju Miki[1], Misa Yoshimoto[1] ([1]*Autonomic Physiology, Human Life and Environment, Nara Women's University*)

Fear memory recall activates sympathetic nerve activity (SNA) and influences cardiovascular functions. One of the major areas that influence sympathetic and cardiovascular regulation is the hypothalamic paraventricular nucleus (PVN). However, it remains unknown how the PVN neuronal activity (PVNNA) changes during fear memory recall to modulate sympathetic activity and cardiovascular function. The present study aimed to investigate the changes in the PVNNA and its role in the regulation of SNA, arterial pressure, and heart rate during fear memory recall in conscious rats. Male Wistar rats were chronically instrumented with multiple electrodes (100- μm stainless steel wires) to measure PVNNA, renal SNA (RSNA), lumbar SNA (LSNA), and electroencephalogram, electromyogram, and electrocardiogram data, as well as a catheter to measure arterial pressure (AP). In the fear conditioning trials, a tone (conditioned stimulus; CS) followed by a brief electrical shock (0.1 mA, 1s) was administered to rats twice a day for two days in a shock cage. Rats were presented CS in their home cage during the memory recalling trials. PVNNA did not differ significantly in response to CS presentation before and after fear conditioning but remained high after fear conditioning. With CS presentation, RSNA and LSNA increased. RSNA returned to control levels 20 seconds after the presentation, while LSNA remained high. Thus, these data suggest that recalling fearful memories elicited by conditioned stimuli activates the PVNNA, resulting in a state of readiness for fight or flight.

### Poster Presentation 1

### [1P08]
### Endocrine, metabolic physiology, Thermoregulation

March 16(Wed), 12:30 - 14:30, Zoom P8

### [1P08-01]
### Histone deacetylase 3 inhibition mitigates hypothyroidism-induced cerebellar developmental defects in mice

*Alvin Susetyo[1], Sumiyasu Ishii[1], Izuki Amano[1], Noriyuki Koibuchi[1] ([1]*Gunma University*)

**Background:** Perinatal hypothyroidism impairs cerebellar growth and results in motor coordination defects. In hypothyroid status, thyroid hormone receptor (TR) binds to histone deacetylase 3 (HDAC3) complex and act as a transcriptional repressor. In this research we aim to study the role of HDAC3 in cerebellar developmental defects induced by hypothyroidism.
**Methods:** An anti-thyroid agent propylthiouracil was administered to pregnant mice to induce perinatal hypothyroidism and cerebellar developmental defects in pups. The pups were further treated with an HDAC3 inhibitor RGFP966. Motor coordination was analyzed by three behavioral tests. Morphological changes of the cerebellums were assessed by cresyl violet staining. Cerebellar gene expression levels were measured by RT-qPCR.
**Results:** Treatment with RGFP966 significantly mitigated motor coordination defects, increased cerebellar weight, and improved cerebellar morphology in hypothyroid mice. Inhibition of HDAC3 activity also increased mRNA levels of TR-target genes as well as other cerebellar developmental genes.
**Summary:** The enzymatic activity of HDAC3 plays an important role in motor coordination defects induced by hypothyroidism.
**COI:** No

### [1P08-02]
### Role of the silencing mediator of retinoid and thyroid hormone receptors on brain development

*Izuki Amano[1], Ayane Ninomiya[1], Megan Ritter[2], Kristen Vella[2], Anthony Hollenberg[2], Noriyuki Koibuchi[1] ([1]*Department of Integrative Physiology Gunma University Graduate School of Medicine*, [2]*Division of Endocrinology, Weill Cornell Medicine*)

The silencing mediator of retinoid and thyroid hormone receptors (SMRT) is known as the essential coregulators of the thyroid hormone receptor (TR), mediating transcriptional repression via histone deacetylation. Thyroid hormones (THs) play an essential role in many physiological processes including brain development via the TR. However, the role of SMRT in the central nervous system is little known. Recently, de novo genetic variants in nuclear corepressors are found in pediatric patients with intellectual disabilities or autism spectrum disorders (ASD). Thus, we generated a mouse model to understand the role of SMRT in the brain. We used mice with conditional SMRT ($SMRT^{lox/lox}$) alleles in combination with mice that express *Cre* recombinase in a neuronal specific fashion (Snap25-Cre). Global deletion of SMRT during embryogenesis results in lethality. Now, we found that neuronal specific SMRT KO mice survived without obvious impairment of neuronal development. However, adult SMRT knock-out mice showed mild cognitive and social disability seen in ASD patients. SMRT may have important roles in maintaining normal neuronal functions in the developing brain.

### [1P08-03]
### The lactational perfluorooctane sulfonate (PFOS) exposure causes the aberrant development of mouse cerebellar function

*Ayane Ninomiya[1], Abdallah Mshaty[1], Asahi Haijima[2], Hiroyuki Yajima[1], Michifumi Kokubo[1], Miski Aghnia Khairinisa[1], Winda Ariyani[1], Yuki Fujiwara[1], Sumiyasu Ishii[1], Nobutake Hosoi[3], Hirokazu Hirai[3], Izuki Amano[1], Noriyuki Koibuchi[1] ([1]*Dept. Integrative Physiology, Grad. Sch. Medicine, Gunma Univ.*, [2]*Laboratory for Environmental Brain Science, Faculty of Human Sciences, Waseda University*, [3]*Dept. Neurophysiology and Neural Repair, Grad. Sch. Medicine, Gunma Univ.*)

Recent studies showed a possible association between perfluorooctane sulfonate (PFOS) and developmental disabilities. Developmental disabilities such as motor and social deficits are known to be caused by the aberrant cerebellar development. However, the neurotoxic effects of PFOS on the cerebellar functional development remain unclear. We examined the effect of early lactational PFOS exposure on motor coordination, social activity, and anxiety in male mice. We orally administered a PFOS solution to dams from postnatal day 1 to 14. At 10 weeks old, we conducted a behavior test battery to evaluate motor performance, social activity, and anxiety, followed by electrophysiology and Western blot analysis. PFOSexposed mice displayed impairments in motor coordination, but not much in social activity and anxiety. Whole-cell patch-clamp recordings from Purkinje cells revealed that the neurotransmitter release and long-term depression at parallel fiber-Purkinje cell synapses are affected by PFOS exposure. Western blot analysis indicated that PFOS exposure increased syntaxin binding protein 1 (Munc18-1) and glutamate metabotropic receptor 1 (mGluR1) protein levels. The present study demonstrates that lactational PFOS exposure may have disrupted the pre- and postsynaptic plasticity at parallel fiber-Purkinje cell synapses, causing profound, long-lasting abnormal effects on the cerebellar function.

### [1P08-04]
### A role of corticotropin-releasing factor type 1 receptor in the rat nodose ganglion

*Asuka Mano[1], Tamotsu Shibasaki[1], Yoshihiko Kakinuma[1] ([1]*Nippon Medical School*)

Objective
The aim of this study was to detect the expression of corticotropin releasing factor type 1 receptor ($CRF_1$)-like immunoreactivity (LI) in the rat nodose ganglion (NG) and to clarify the possible involvement of CRF/$CRF_1$ system in the signal transduction of visceral sensory information to central nervous system and the effects of stress exposure on vagal nerve function.
Methods
The characterization of $CRF_1$-LI and the expression of stress-induced phosphorylatedcyclic AMP responsible element binding protein (pCREB) in the rat NG were achieved by the immunohistochemical (IHC) method. Fast blue, retrograde tracer, was microinjected into the proximal colon to evaluate vagal innervation to proximal colon.
Results
IHC analysis revealed that $CRF_1$-LI was detected in the cholinergic neuron and immobilization stress increased the expression of pCREB on vagal neurons including of $CRF_1$-positive cells in the NG. Neuronal tracing study clarified that some $CRF_1$-positive vagal afferent neurons innervate proximal colon.
Conclusion
This study provides the possible role of CRF/$CRF_1$ system in the signal transduction of colonic sensory information to central nervous system via NG and the stress-induced vagal activity s probably mediated by $CRF_1$.

### [1P08-05]
### Single-molecule analysis for cytoskeletal dependence of intracellular insulin granule behavior

*Hiroyasu Hatakeyama[1], Tomomi Oshima[1], Shinichiro Ono[1], Noriko Takahashi[1] ([1]*Department of Physiology, Kitasato University School of Medicine*)

Insulin secretion from pancreatic $\beta$-cells is critical for glucose homeostasis, and the importance of insulin granule delivery to the plasma membrane in regulating insulin secretion has been suggested. By quantifying intracellular insulin granule movement based on single-molecule imaging of insulin granule membrane proteins labeled with Quantum dot fluorescent nanocrystals in rat INS-1 cells, we here analyzed the roles of cytoskeletal elements, microtubules and F-actin, on the movement. For microtubules, both a destabilizer nocodazole and a stabilizer paclitaxel significantly suppressed the movement, suggesting the importance of microtubule dynamics rather than static microtubule architectures. In contrast, an F-actin destabilizer latrunculin B and a stabilizer jasplakinolide facilitated and suppressed the movement, respectively, suggesting negative roles of F-actin. These observations were consistent with the data obtained by tracking insulin labeled with a conventional fluorescent molecule TMR. Our data demonstrate that both microtubules and F-actin are involved in the dynamic movement of insulin granules, but the roles are different from each other.

### [1P08-06]

### A novel nucleic acid analogue COA-Cl enhances glucose-dependent insulin secretion-II

*Ikuko Tsukamoto[1], Akram Hossain[1], Maki Takata[1], Wenhua Liu[2], Katsuya Hirano[1], Junsuke Igarashi[3], Masaaki Tokuda[1], Ryoji Konishi[1] ([1]*Fac. of Med., Kagawa Univ.*, [2]*Chinese Academy of Sciences*, [3]*Morinomiya Univ.of Med. Sci.*)

We previously reported that a newly synthesized nucleic acid analogue COA-Cl has angiogenic potency with the promotion of synthesis and secretion of vascular endothelial growth factor (VEGF). COA-Cl also exhibits neurotrophic/neuroprotective property. At the 97th annual meeting, we briefly showed the effects of COA-Cl on insulin secretion. This time we discuss about these effects again together with the recent results. *in vitro* study: In mouse insulinoma cells (MIN6), COA-Cl enhanced the secretion of insulin only under the high-glucose condition while a typical diabetes therapeutic drug Glibenclamide(SU) enhanced the insulin secretion regardless of glucose concentration. COA-Cl also enhanced the glucose-induced $Ca^{2+}$ influx in MIN6. Furthermore, enhancement of the glucosedependent insulin secretion was detected in primary cultured rat pancreatic islets. *in vivo* study: We performed the OGTT (oral glucose tolerance test) using normal rats. COA-Cl increased the insulin concentration in blood. However the effects of COA-Cl on blood sugar were unclear.

### [1P08-07]

### Suppression of maternal pituitary prolactin during late pregnancy does not disrupt the nurturing behavior in the offspring

*Taku James Sairenji[1], Shinnosuke Masuda[1], Kwan-Ee Oh[1], Yuya Higuchi[3], Takuya Araki[3], Noriaki Shimokawa[2,1], Noriyuki Koibuchi[1] ([1]*Dept Intgr Physiol, Med Grad Sch, Gunma Univ*, [2]*Dept Nutr, Takasaki Univ Health Welf*, [3]*Dept Clin Pharmacol Ther. Med Grad Sch, gunma Univ*)

Prolactin (PRL) secreted during late pregnancy is involved in initiating maternal behavior in rodents. We previously reported the possibility of this maternal PRL also to be is important in developing nurturing behavior in the offspring during the fetal stage. In this study, we aimed to gain an accurate view of pituitary PRL and placental PRL family secretion during late pregnancy and how the PRL concentration would affect the fetus, by using C57BL/6 wild type mice. First, we measured the plasma pituitary PRL concentration every 4 hours from gestational day 17(G17) to delivery. Second, we suppressed the PRL secretion during late pregnancy by bromocriptine (BC, dopamine agonist) injection. Then the nurturing behavior of the offspring were investigated when they matured. Third, we measured Prolactin-3b1(PRL3B1) secreted by the placenta. As a result, mice born to the BC injected dams did not show any abnormality in maternal behavior compared to the control group. The secretion of PRL3B1 is abundant till G19. These results suggest that the development of maternal behavior cannot be disrupted by only suppressing pituitary PRL secretion during late pregnancy.

### [1P08-08]

### Sex and age difference in the distribution of estrogen receptors throughout the development

*Larissa Campista Lana[1], Tetsu Hatsukano[1], Kazuhiro Sano[1], Mariko Nakata[1], Sonoko Ogawa[1] ([1]*University of Tsukuba, Laboratory of Behavioral Neuroendocrinology*)

Activation of estrogen receptors, ER$\alpha$ and ER$\beta$, are essential for the regulation of sociosexual behaviors in both female and male. However, their distribution during the development in female and male brain is still unclear. In this study, we aimed to map ER$\alpha$, ER$\beta$, and their colocalization in different age periods that are postnatal day 7, 14, 21, 28, 35, 42, and 56. A transgenic mice that express a red fluorescent protein in ER$\beta$-positive cells (ER$\beta$-RFP$^{tg}$ mice), were used. We analyzed the distribution of ERs in the sexually dimorphic brain areas, the Anteroventral Periventricular Nucleus (AVPV), Bed Nucleus of Stria Terminalis (BNST), Medial Amygdala (MeA) and Ventromedial Hypothalamus (VMH). Overall, females tended to show an increase in the number of ER$\alpha$ expressing neurons along the age in the AVPV, BNST, and VMH while this age-dependent increase was not observed in males. The number of ER$\beta$, and ER$\alpha$/$\beta$ expressing neurons decreased in both sexes along the age in the VMH. Our results indicated that the distribution of ERs expressing neurons changes in brain area- and sex-dependent manner throughout neonatal to young adult developmental period. (Supported by KAKENHI 15H05724 to SO)

### [1P08-09]

### Effect of estradiol on thermoregulatory responses in ovariectomized rats administrated TREK agonist

*Yuki Uchida[1], Shotaro Kamijo[1], Masahiko Izumizaki[1] ([1]*Department of Physiology, Showa University School of Medicine*)

**INTRODUCTION** The TWIK-related potassium (TREK) channels are reported as new cold receptors. The effect of estradiol ($E_2$), one of female hormones, on thermoregulatory responses via TREK has not been elucidated. **METHODS** Ovariectomized rats were implanted a silastic tube with or without $E_2$ (22.3mg) underneath the dorsal skin ($E_2$(+) and $E_2$(-) groups) and nano tag (Kissei Comtec) for body temperature ($T_b$) measurement into peritoneal cavity. After intraperitoneal administration of TREK agonist (Ostruthin Imperatorin, 4.2$\mu$g) or vehicle, rats were exposed to 27°C for 2 hours with continuous $T_b$, activity, tail skin temperature ($T_{tail}$), and thermoregulatory behavior assessed by tail-hiding behavior measurements. **RESULTS** In the preliminary experiment, we observed the increased $T_b$ and activity and the decreased $T_{tail}$ in the $E_2$(+) group administrated the TREK agonist; however, TREK agonist did not affect thermoregulatory behavior in the $E_2$(+) group. **CONCLUSION** Estradiol might affect thermoregulatory responses via TREK in ovariectomized rats.

### [1P08-10]

### Studies on the mechanism of body temperature determination, using insulin like growth factor binding protein 2 (*Igfbp2*) conditional knockout mouse

*Yuki Yoshimura[1], Kazuomi Nakamura[2], Akira Futatsugi[3], Katsuhiko Mikoshiba[4], Tatsuo Watanabe[1] ([1]*Tottori University*, [2]*Tottori University Hospital*, [3]*Kobe City College of Nursing*, [4]*Shanghai Tech University*)

Core body temperature (Tb) is set around at 37°C; however, the mechanisms for this setting are still unknown. To study the effect of pregnant mouse's body temperature on the Tb of its offspring, we cultured C57BL/6N mouse embryos *in vitro* at 38°C (38°C-group) or 37°C (the 37°C-control-group) from the pronuclear stage to blastocyst, transferring thereafter into uteri of pseudo-pregnant mice. The Tb of the male offspring of 9 weeks old was significantly lower in the 38°C-group than in the 37°C-control-group, with the higher expression of insulin-like growth factor (*Igf-1*) and Igf-binding protein 2 (*Igfbp2*) mRNAs in the hypothalamus of the 38°C-group. Therefore, we generated brain-specific *Igfbp2* knockout (KO) mice. When bred naturally, there was no difference in the Tb between the *Igfbp2* conditional KO mice and the control mice. On the other hand, when embryos were all cultured at 38°C, the *Igfbp2* condition KO mice showed the Tb that was significantly higher than the control mice. These results suggest that brain-specific (possibly, the hypothalamic) *Igfbp2* is responsible for the development of the lower Tb seen in the 38°Cgroup of C57BL/6N mice, and that this "binding protein" plays an important role in the Tb determination.

### [1P08-11]

### Live-cell imaging analysis of glucose metabolism in hepatocytes

*Mina Horikoshi[1], Saki Tsuno[2,3], Marie Mita[2], Tetsuya Kitaguchi[4], Kazuki Harada[2], Mitsuharu Matsumoto[3], Takashi Tsuboi[1,2] ([1]*Department of Biological Sciences, Graduate School of Science, The University of Tokyo*, [2]*Department of Life Sciences, Graduate School of Arts and Sciences, The University of Tokyo*, [3]*Dairy Science and technology Institute, Kyodo Milk Industry Co.*, [4]*Laboratory for Chemistry and Life Science, Institute of Innovative Research, Tokyo Institute of Technology*)

Metformin is a well-known anti-type 2 diabetes drug used in the last decades. It inhibits gluconeogenesis in the liver and results in lowering blood glucose level. Recent study suggested that metformin might antagonize hepatic glucagon-stimulated cAMP and glucose production. However, little is known about the effect of metformin on glucose metabolism in hepatocytes. Here we visualized glucose metabolism at the single-cell level in murine hepatocytes by using protein-based glucose indicator. After glucose starvation, extracellular glucose stimulated elevation of intracellular glucose level. In the next step, we will examine whether metformin alters glucagon-stimulated cAMP and glucose level. Our study will shed light on the mechanism of the well-used anti-diabetic drug.

### Poster Presentation 1

### [1P09]
### Nutritional and metabolic physiology, Thermoregulation

March 16(Wed), 12:30 - 14:30, Zoom P9

### [1P09-01]
### Visualization of intracellular glucose metabolism dynamics by red fluorescent protein-based glucose indicators

*Marie Mita[1], Izumi Sugawara[2], Kazuki Harada[1], Motoki Ito[2], Mai Takizawa[1], Kentaro Ishida[3], Hiroshi Ueda[4], Tetsuya Kitaguchi[4], Takashi Tsuboi[1,2] ([1]*Department of Life Sciences, Graduate School of Arts and Sciences, The University of Tokyo*, [2]*Department of Biological Sciences, Graduate School of Science, The University of Tokyo*, [3]*Myoridge Co. Ltd.*, [4]*Laboratory for Chemical and Life Science, Institute of Innovative Research, Tokyo Tech*)

Glucose metabolism is an important for production of ATP and maintaining energy homeostasis in the cells. The multicolor imaging using single fluorescent protein-based indicators is suitable for detecting the interplay of intracellular molecules involved in glucose metabolism in the cells. However, these indicators are mainly based on a green fluorescent protein and it has been difficult to monitor the dynamics of multiple molecules simultaneously. Here we developed a red fluorescent protein-based glucose indicators, named Red Glifons, and succeeded in visualizing the interplay between glucose and glucose metabolites by dual-color imaging in the living cells. We found that the intracellular glucose, ATP, and lactate levels were increased but pyruvate levels were maintained by high glucose stimulation in HeLa cells. The intracellular pyruvate levels were increased by high glucose stimulation with inhibition of monocarboxylate transporter or lactate dehydrogenase. Taken together, these data suggest that glucose was rapidly metabolized into lactate due to enhanced glycolysis, and pyruvate was transported into mitochondria for fuel of the citric acid cycle. The multicolor imaging using these indicators will shed light on the understanding of glucose metabolism and energy homeostasis in variety of the cells.

### [1P09-02]
### Novel Food preferences in vitamin C deficient rats

Toshiaki Yasuo[1], *Takeshi Suwabe[1], Fumihiko Nakamura[2], Noritaka Sako[1] ([1]*Asahi univ.*)

It is still unclear how animals regulate the injestion of deficient vitamin C (VC). Our previous behavioral studies using Osteogenic Disorder Shionogi (ODS) rats, which lack the ability to synthesize VC, have shown that the preference for familiar VC solutions is increased in ODS rats after VC deprivation compared to before (Yasuo et al., 2019). However, it is not clear whether VC deficient rats ingest novel VC contained diets selectively. In the present study, we conducted a food choice test in which VC deficient ODS rats were presented with a novel VC contained diet and a VC deficient diet (The ODS rats were naïve to them). The results showed that the novel VC contained diet was ingested more than the VC deficient diet on all experimental days after VC deprivation. These results suggest that the ODS rats may ingest VC during VC deficiency selectively, even if they have never ingested VC solution or VC contained food before VC deficiency.

### [1P09-03]
### Regulatory mechanism of glucagon-like peptide-1 secretion by L-phenylalanine in enteroendocrine cells

*Yuri Osuga[1], Kazuki Harada[1], Takashi Tsuboi[1] ([1]*Department of Life Sciences, Graduate School of Arts and Sciences, The University of Tokyo*)

Glucagon-like peptide 1 (GLP-1) is a gastrointestinal hormone secreted from small intestinal enteroendocrine L cells when they detect nutrients in the gastrointestinal lumen. GLP-1 promotes insulin secretion from pancreatic beta cells in a glucose-dependent manner, and activates the afferent vagus nerve, which is known to suppress appetite. However, the precise mechanism by which nutrients in the gastrointestinal lumen trigger GLP-1 secretion remains unclear. In the present study, we focused on L-phenylalanine (LPhe), a potent secretagogue of GLP-1 secretion from small intestinal enteroendocrine L cells, and used the intracellular $Ca^{2+}$ imaging to identify the pathway leading to GLP-1 secretion. Treatment of mouse small intestinal enteroendocrine L cell line STC-1 cells with L-Phe caused an increase in the intracellular $Ca^{2+}$ concentration ($[Ca^{2+}]_i$). Next, we coadministrated $G_q$ protein inhibitor or G protein-coupled receptor GPR142 antagonist with L-Phe, and found that the L-Phe-induced $[Ca^{2+}]_i$ increase was suppressed. Thus, GPR142 may be coupled with $G_q$ and a putative L-Phe receptor. Furthermore, application of L-Phe to the cells under low extracellular $Na^+$ conditions, which inhibited the function of the $Na^+$- dependent amino acid transporter, did not induce an increase in $[Ca^{2+}]_i$. These findings suggest that GPR142 is important for L-Phe-induced $[Ca^{2+}]_i$ and GLP-1 secretion.

### [1P09-04]
### *In vivo* imaging analysis of metabolic functions in the liver

*Saki Tsuno[1,2], Marie Mita[1], Mina Horikoshi[3], Tetsuya Kitaguchi[4], Kazuki Harada[1], Mitsuharu Matsumoto[2], Takashi Tsuboi[1,3] ([1]*Department of Life Sciences, Graduate School of Arts and Sciences, The University of Tokyo*, [2]*Dairy Science and Technology Institute, Kyodo Milk Industry Co. Ltd.*, [3]*Department of Biological Sciences, Graduate School of Science, The University of Tokyo*, [4]*Laboratory for Chemistry and Life Science, Institute of Innovative Research, Tokyo Institute of Technology*)

Overnutrition-related excess lipid accumulation in organs, such as the liver and skeletal muscles, leads to insulin resistance. This induces impaired glucose uptake and increased gluconeogenesis in the liver, resulting in increased blood glucose levels. However, the dynamics of intracellular molecules, including glucose, in hepatocytes remain unclear. Real-time *in vivo* imaging of metabolic molecules in hepatocytes is needed to elucidate the pathogenesis of various diseases caused by excess lipid accumulation. We attempted to establish a method for visualization of glucose level at the single-cell level in mice liver in real-time and to monitor the dynamics of intracellular glucose. The glucose sensor was expressed in hepatocytes in mice by orbital injection using adeno-associated virus. Glucose elevation in hepatocytes was monitored upon injecting 2 mg/g of glucose during fasting. Currently, we are investigating the conditions to work with fluorescent protein sensors targeting other metabolic factors and intracellular signaling molecules in mice hepatocyte to reveal mechanisms underlying glucose metabolism.

### [1P09-05]
### Search for amino acids that influence circadian rhythm

*Shinya Aoyama[1], Yasukazu Nakahata[1], Kazuyuki Shinohara[1] ([1]*Grad. Sch. of Biomed. Sci., Nagasaki Univ.*)

Several amino acids ingestion induces a phase-resetting of the peripheral circadian clock. However, the role of each amino acid on the period and amplitude of the circadian clock remains unclear. In this study, we investigated the effects of a single amino acid deficiency on circadian rhythm. We used a *Bmal1* promoter driven luciferase reporter (*Bmal1*-Luc) to detect and analyze the circadian rhythm of molecular clock in the human differentiated myotube cells. After synchronization of circadian clock by dexamethasone, the luminescence was recorded for 5 days using the real-time luminescence monitoring system (Kronos HT, ATTO). The medium deficient in single amino acid was supplemented with 2% dialyzed FBS. The screening of 15 kinds of single amino acid-deficient medium showed that methionine and cystine were the two amino acids that greatly affected the period of *Bmal1*-Luc compared to the complete medium. The period of myotubes cultured in methionine- or cystine-deficient medium was significantly longer than that in complete medium. The methionine and cystine are sulfur-containing amino acids, and are known to be involved in methylation and antioxidant functions, respectively. We will investigate the mechanism of circadian clock regulation by these two amino acids.

### [1P09-06]

### Properties and in vivo functions of EID1 in the suppression of lipid accumulation

*Itsuki Takahashi[1], Diana Vargas[1,2], Tomohiko Sato[1,2,3], Mitsue Miyazaki[1,4], Kaoru Utida[1], Izuki Amano[2], Ryosuke Kaneko[5], Fernando Lizcano[6], Noriyuki Koibuti[2], Noriaki Shimokawa[1,2] ([1]*Takasaki University Graduate School of Health and Welfare* , [2]*Gunma University Graduate School of Medicine,* [3]*Ota College of Medical Technology,* [4]*Hirosaki University Graduate School of Medicine,* [5]*Graduate School of Frontier Biosciences, Osaka University,* [6]*Universidad de La Sabana*)

Obesity is said to be associated with increased triglyceride levels in adipocyte and is a major factor for the development of lifestyle diseases, such as type 2 diabetes and cardiovascular diseases. We recently showed that EP300-interacting inhibitor of differentiation 1 (EID1) inhibits the accumulation of triglycerides in mouse pre-adipocyte 3T3-L1 cells through the downregulation of glycerol 3-phosphate dehydrogenase, which is a key enzyme in the synthesis of triglycerides. To clarify the function of EID1 in vivo, we generated EID1 transgenic mice (EID1 Tg mice) expressing EID1 in only adipose tissues. The gene expression of EID1 in EID1 Tg mice was significantly upregulated in both brown and white (subcutaneous and visceral fat) adipose tissues compared to wildtype mice. Interestingly, when these mice were exposed to a cold environment (4°C, 2 h) after administration of radiolabeled glucose (2-deoxy-2-[18F] fluoro-D-glucose), the uptake of the glucose was significantly increased in brown adipose tissue of the interscapular region. To determine the signal transduction pathways of the glucose uptake, we are currently making an exhaustive list of gene expression by DNA microarray analysis.

### [1P09-07]

### Effects of ketogenic diet on abnormal behaviors in a rat model of attention deficit hyperactivity disorder

*Masanori Ogata[1], Kei Eto[1], Hitoshi Ishibashi[1] ([1]*Department of Physiology, School of Allied health Sciences, Kitasato University*)

The ketogenic diet (KD) is a high fat, low carbohydrate and adequate-protein diet, and has been used in treatment-resistant epilepsy. In recent years, the effects of KD attracted much attention to the treatment of some other neurological disorders. Attention deficit hyperactivity disorder (ADHD) is one of the most common neurodevelopmental disorders, and the effects of KD on ADHD are not yet clarified. To evaluate the effect of KD on the abnormal behavior of ADHD model rats with neonatal dopamine (DA) depletion, open field (OF), elevated plus maze (EPM) and 24-hour home cage (24-h) tests were performed in the present study. The rats were fed normal diet (ND) or KD for 5 weeks after weaning. The ND-fed rats with DA depletion showed increases in locomotor activity and anxiolytic behaviors in the OF and/or EPM tests, and a decrease in locomotor activity in the 24-h test. The anxiolytic behavior in the EPM and hypo-locomotor activity in 24-h tests of the ND-fed rats with DA depletion were ameliorated by KD feeding. There was no significant difference in the behaviors between control rats fed with the KD and ND. The results suggest that KD is effective treatment for part of abnormal behaviors in the ADHD model rats.

### [1P09-08]

### Automated measurement of abdominal temperature in mice using microchip

*Ayato Nakata[1], Syunto Ishida[1], Takamichi Kondo[1], Daichi Tsujita[1], Kiyoshi Matsumura[1] ([1]*Osaka Institute of Technology*)

The IPTT-300 microchip for body temperature measurement in mouse (Bio Medic Data System) has advantages over the conventional body temperature measurement capsule (G2-E-mitter, STARR Life Science): small size (1/10 in weight), individual identification, and low cost (about 1/40). However, there are problems as follows. (1) It is necessary to bring the reading equipment close to a mouse and manually press the measurement button. This often excites the mouse resulting in unstable measurements. (2) It is complicated to manually measure the temperature in multiple mice at a certain interval. (3) The microchip moves around in the abdominal cavity and easily enters the scrotum in male mice. To solve these problems, we improved the surgical method and automated the measurement. The microchip was fixed to the abdominal muscle with sutures. The automation of the measurement was accomplished by controlling three servo motors with a CPU board for a robot (VS-RC003HV, Vstone). Two of the servomotors were used to move the reading equipment and scan the underside of the mouse cage in a plane, and the third servomotor was used to press the measurement button in synchronization. This made it possible to measure the temperature of the mouse abdominal cavity in six radially arranged cages every 10-minutes for several days. Using this system, we studied the febrile response to zymosan in mice and possible involvement of endocannabinoid pathway in it.

### [1P09-09]

### Effect of theobromine intake on learning and memory in senescence-accelerated mouse-prone 8 (SAMP8) mice

*Eri Sumiyoshi[1], Kentaro Matsuzaki[1], Naotoshi Sugimoto[1,2], Masanori Katakura[3], Osamu Shido[1] ([1]*Department of Environmental Physiology, Faculty of Medicine, Shimane University,* [2]*Department of Physiology, Graduate School of Medical Science, Kanazawa University,* [3]*Department of Nutritional Physiology, Faculty of Pharmaceutical Sciences, Josai University*)

Our prior research suggested that in normal mice and rats, theobromine (TB) diet intake improves cognitive function. This study investigated the effects of TB-containing diet on cognitive function of learning and memory deficits in the senescence accelerated mouse (SAM) -prone 8 (SAMP8) mouse. The SAM-resistant 1 (SAMR1) mice were used as normal aging control. SAMP8 mice (15 weeks old) were divided into two groups, a normal diet intake group (P8CN: n = 8) and a TB-containing (0.05%) diet intake group (P8TB: n = 8) for 50 days under the free-feeding and drinking water. Cognitive function was assessed using Novel object recognition (NOR) task. After the NOR task was completed, blood and the brain (cerebral cortex, hippocampus) were sampled. The brain-derived neurotrophic factor (BDNF) concentrations in the cerebral cortex and hippocampus were quantified by the ELISA method. The NOR task showed that short-term memory in the P8TB group was significantly improved compared to that in the P8CN group ($P < 0.05$). In addition, BDNF concentrations in the cerebral cortex and hippocampus were significantly increased in the P8TB group compared to the P8CN group ($P < 0.05$). These results suggest that intake of TB-containing diet may improve cognitive function even in learning and memory deficits SAMP8 mice.
COI: No

### [1P09-10]

### Elevation of α-Tocopherol level in plasma and tissues during hibernation period in a mammalian hibernator, Syrian hamster.

*Reo Otsuka[1], Yoshifumi Yamaguchi[2,1] ([1]*Graduate School of Environmental Science, Hokkaido University,* [2]*Institute of Low Temperature Science, Hokkaido University*)

Mammalian hibernators such as Syrian hamsters do not exhibit a sign of cell death and physiological damages by prolonged hypothermia and rewarming, both of which are experienced during hibernation and could lead to organ damages in non-hibernators such as human and mice. We previously reported that primary hepatocytes of Syrian hamsters exhibit resistance to cold-induced ferroptosis with the aid of a dietary α-toco pherol (αT), a Vitamin E isoform, as Syrian hamsters fed with low vitamin E diet lost cold resistance in their hepatocytes. However, they can successfully hibernate, suggesting unknown mechanisms to compensate for low αT intake *in vivo*. To examine the role of αT in hibernation *in vivo*, we measured αT levels during hibernation in Syrian hamsters. Plasma αT levels were significantly higher in hibernating state than in non-hibernating state. Interestingly, post-hibernating animals that quit hibernation for more than 2 months decreased plasma αT level o the same level as non-hibernating animals. These results suggest that hamsters can elevate circulating αT levels in animal bodies during hibernation, which may be contribute to *in vivo* cold resistance of Syrian hamsters.

### [1P09-11]

### Can EID1, a fat accumulation inhibitor in adipocytes, also suppress fat accumulation in hepatocytes?

*Mitsue Miyazaki[1,2], Wataru Miyazaki[1], Noriaki Shimokawa[2] ([1]*Department of Bioscience and Laboratory Medicine, Hirosaki University Graduate School of Health Science,* [2]*Department of Food and Nutrition, Takasaki University Graduate School of Health and Welfare*)

Overexpression of E1A-like inhibitor of differentiation 1 (EID1) in the process of adipocyte differentiation suppresses the accumulation of triglycerides in adipocytes. Our previous study found that the transcription factor EID 1 suppressed the transcription of glycerol-3- phosphate dehydrogenase 1 (GPD1), an essential enzyme in the synthesis of triglycerides, resulting in suppressed fat accumulation. In this study, we examined whether EID1 is involved in fat accumulation in hepatocytes that accumulate fat in cells as well as adipocytes. First, we added oleic acid to promote fat accumulation in EID1-overexpressed human hepatocyte, HepG2. We observed a higher accumulation of lipid droplets in the cells compared with the control. Next, we examined the mRNA expression levels of several genes, including GPD1, which was suppressed in adipocytes by EID1 overexpression, and a glucose transporter, which was unchanged in adipocytes, and the levels of these genes were decreased in the hepatocytes. On the other hand, overexpression of EID1 increased the expression of PCSK9, which is involved in cholesterol and fatty acid metabolism. These results indicated that EID1 may act as a regulator of both fat accumulation and glucose metabolism in hepatocytes.

### Poster Presentation 1

### [1P10]
### Environmental physiology

March 16(Wed), 12:30 - 14:30, Zoom P10

### [1P10-01]
### Functional connectivity between frontal medial cortex and angular gyrus contributes gender and age difference on odor sensitivity.

*Yusuke Takatsuru[1,2], Shunichi Mogi[3], Tatsuya Nishikata[4], Keita Yonemochi[5] ([1]*Dept. Nutr. Health Sci., Toyo Univ.* , [2]*Johmoh Hospital,* [3]*International University of Health and Welfare,* [4]*Josai Clinic,* [5]*Gunma Prefectural College of Health Sciences*)

Preference of odor is one of the keys for rehabilitation of swallowing. On the other hand, sensitivity is different between gender and decrease depend on age. These facts rely on the neuronal circuits in the specific brain region. However, it is not still fully understood the neuronal circuits which is the key of gender/age difference on odor sensitivity. In this study, we combined the odor sensitivity test and functional magnetic resonance imaging (fMRI) to find the neuronal circuits which contribute the gender/age dependency on odor sensitivity. The value of odor test score was significantly high in female than in male and showed age dependent decrease. We found the four functional connectivity which significantly different between male and female. One of them, connectivity of the left medial frontal cortex (MedFC.l) and left angular gyrus (AG. l) showed age dependent change. We concluded that functional connectivity of MedFC.l - AG.l is one of the important neuronal circuit which regulate the gender/age dependent odor sensitivity.

### [1P10-02]
### A histological study of vasoactive intestinal peptide neurons establishing sexually dimorphisms in the bed nucleus of the stria terminalis in mice

*Hideto Arai[1], Shinji Tsukahara[1] ([1]*Graduate School of Science and Engineering, Saitama University.*)

The bed nucleus of the stria terminalis (BNST) contains a sexually dimorphic nucleus having more vasoactive intestinal peptide (VIP) neuronal fibers in men than in women; however, such sex difference remains unclear in mice. In this study, we performed VIPimmunohistochemical analysis of the BNST in prepubertal (20-day-old), postpubertal (56- day-old), and adult (10-12-week-old) mice. Many VIP-immunopositive fibers were found in the oval nucleus of the BNST. The amount of VIP-immunopositive fibers was greater in adult males than in adult females, although such sex difference was not seen in prepubertal and postpubertal mice. Next, to identify the origin nucleus of VIP neurons projecting to the BNST, an AAV vector, which infects at nerve terminals and expresses tdTomato dependently of Cre recombinase, was injected into the BNST of transgenic mice that express Cre recombinase under the control of the VIP gene promoter. As a result, tdTomto-expressing neuronal cell bodies were observed in the basolateral amygdala. These results suggest that the BNST of mice contains a sexually dimorphic nucleus that contains many more fibers originating from VIP neurons of the basolateral amygdala in males than in females. This sex difference may be established at the adult period.

### [1P10-03]
### Role of calbindin neurons establishing sexually dimorphism of the medial preoptic area in the control of male sexual behavior

*Kaito Kobayashi[1], Moeri Mitsuzuka[1], Masahiro Morishita[1], Yosuke Tsuneoka[2], Shinji Tsukahara[1] ([1]*Saitama University graduate School of Science and Engineering* , [2]*Toho University faculty of Medicine*)

The medial preoptic area (MPA) is a sexually dimorphic region playing an important role in sex-specific social behaviors. Calbindin neurons showing morphological sex differences may contribute to the sexual dimorphism of the MPA; however, the roles in social behaviors remain unknown. In this study, c-Fos-analysis was performed to examine the activity of calbindin neurons in mice displayed social behaviors. Approximately half of calbindin neurons activated in males when displayed sexual behavior, but not in males when displayed aggressive behavior and in females when displayed sexual and maternal behaviors. Calbindin neurons in the MPA are divided into neurons projecting the ventral tegmental area (VTA) and interneurons, and the former are only in males. Therefore, we examined sexual behavior performance of males in which the activity of VTA-projecting calbindin neurons was chemogenetically suppressed. As a result, the first intromission and ejaculation were delayed in the males as well as a reduced frequency of intromission and ejaculation. These results suggest that calbindin neurons in the male MPA are involved in male sexual behavior. In addition, we will report the result of analysis of males in which the activity of VTA-projecting calbindin neurons is chemogenetically activated.

### [1P10-04]
### Endogenous oxytocin exerts anti-nociceptive and anti-inflammatory effects via neuronal and humoral pathways in rats

*Mitsuhiro Yoshimura[1,7], Haruki Nishimura[2,1], Kenya Sanada[1], Satomi Sonoda[8,1], Kazuaki Nishimura, Kazuhiko Baba[2,1], Naofumi Ikeda[2,1], Takashi Maruyama[1], Yuki Nonaka[1], Ryoko Baba[3], Tatsushi Onaka[4], Takafumi Horishita[5], Hiroyuki Morimoto[3], Yasuhiro Yoshida[6], Makoto Kawasaki[2], Akinori Sakai[2], Becky Conway-Campbell[7], Stafford Lightman[7], Yoichi Ueta[1] ([1]*Department of Physiology, School of Medicine, University of Occupational and Environmental Health, Japan,* [2]*Department of Orthopaedic Surgery, School of Medicine, University of Occupational and Environmental Health, Japan,* [3]*Department of Anatomy II, School of Medicine, University of Occupational and Environmental Health, Japan,* [4]*Division of Brain and Neurophysiology, Department of Physiology, Jichi Medical University,* [5]*Department of Anesthesiology, School of Medicine, University of Occupational and Environmental Health, Japan,* [6]*Department of Immunology and Parasitology, School of Medicine, University of Occupational and Environmental Health, Japan,* [7]*Translational Health Sciences, Bristol Medical School, University of Bristol,* [8]*Department of Internal Medicine I, School of Medicine, University of Occupational and Environmental Health, Japan*)

Oxytocin (OT) is involved in pain transmission, although the detailed mechanism has not been elucidated. We have generated a transgenic rat line that expresses both human muscarinic acetylcholine receptors (hM3Dq) and mCherry exclusively in the OT neurons in the supraoptic (SON) and paraventricular nuclei (PVN). Descending pain inhibitory system and L5 spinal cord were significantly activated after the subcutaneous (s.c.) injection of clozapine-N-oxide (CNO, 1 mg/kg), with altered gene expression in the L5 dorsal horn. Anti-nociceptive behaviors that were robustly exacerbated in pain models were significantly attenuated after the s.c. injection of CNO, of which effects were ablated by either intrathecal or intraperitoneal injection of OT receptor antagonist. Endogenous OT also exerted anti-inflammatory effects without altering hypothalamus-pituitary-adrenal (HPA) axis, while inhibition of degranulation from mast cells was involved in the response. The results suggest that endogenous OT may exert anti-nociceptive and anti-inflammatory effects via both neuronal and humoral pathways.

### [1P10-05]
### Effects of growth environment on sexual activity in transsexual female rats

*Kanta Wakayama[1], Shinji Tsukahara[1] ([1]*Saitama university graduate school of science and engineering*)

The sexual differentiation of the brain proceeds under the influence of the three critical factors, perinatal testicular androgens, peripubertal gonadal steroids, and sex chromosome genes. However, other factors may influence it. We hypothesized that growth environment affects the sexual differentiation of the brain. To test this hypothesis, transsexual females that are genetical males castrated neonatally and treated estrous hormones in the adult period were divided into two groups. One group were housed with female contemporaries until sexual maturation (female environment group), and another group were housed with male contemporaries (male environment group). These transsexual females were tested for female sexual behavior to compare the sexual activity. There was no difference in the frequency of lordosis, a consummatory behavior of female rats, between groups. However, the frequency of ear-wiggling, an appetitive behavior of female rats, was significantly lower in the male environment group than in the female environment group. This finding supports the notion that transsexual females living with females are more feminine than transsexual females living with males. Growth environment may be a factor altering the sexual differentiation of the brain.

### [1P10-06]

### Formaldehyde gas exposure altered reflexive eye movements in transgenic mice harboring human neuropathy target esterase (hNTE) gene

*Akira Katoh[1], Nami Motosugi[1], Kou Sakabe[1], Minoru Kimura[2] ([1] *Tokai University School of Medicine,* [2] *Tokai University, The Institute of Medical Sciences*)

Formaldehyde and organophosphorus compounds are widely used as insecticides, pesticides, and insect repellents in building materials, and are considered to be causative agents of sick building syndrome. Human Neuropathy Target Esterase (hNTE), an enzyme known to be involved in the process inducing an organophosphorus-induced delayed neuropathy that causes symptoms such as ataxia and paralysis in addition to acute toxicity when organophosphorus is administered, has been reported to be highly active in the blood of patients with sick building syndrome. In this study, we examined the effects of formaldehyde gas exposure on hNTE mice which carried the human PNPLA6 (Patatin-like phospholipase domain containing 6) gene encoding hNTE, we generated previously. We found NTE activity was increased in each tissue in hNTE mice that highly express NTE, several to 100-fold more than wild-type mice. The gain of optokinetic response (OKR) in hNTE mice exposed to 0.6-2 ppm of evaporated formaldehyde for 7 consecutive days, 18 hours a day, were gradually decreased during that exposure period, while no change was found in wild-type mice. Vestibulo-ocular reflex (VOR) was not changed in either mice. The animals were then kept under normal air conditions for 10 days, and impaired OKR found in hNTE mice was not recovered. HE-stained brain sections of hNTE mice showed atrophy of cerebellar Purkinje cells, abnormal laminar structure around the hippocampal dentate gyrus-CA3, and pyramidal cell atrophy in CA3. These results suggest that increased NTE activity in hNTE mice alters their sensitivity to formaldehyde in the neuronal circuit that regulates OKR.

### [1P10-07]

### Possible mechanisms in improved salivary IgA secretion in heatacclimated rats

*Kentaro Matsuzaki[1], Naotoshi Sugimoto[2], Eri Sumiyoshi[1], Masanori Katakura[3], Michio Hashimoto[1], Osamu Shido[1] ([1] *Shimane Univ.,* [2] *Kanazawa Univ.,* [3] *Josai Univ.*)

Salivary immunoglobulin A (IgA) plays a critical role in mucosal immunity. We have shown that salivary IgA secretion is promoted in heat-acclimated rats. However, the mechanism was not fully elucidated. Therefore, this study aimed assessing the mechanism of increased IgA secretion in heat-acclimated rats. Male Wistar rats (10 weeks old) were exposed to an ambient temperature (Ta) of 32 ± 0.2°C for 5 days (HE) for heat acclimation, while control rats were maintained at an Ta of 24 ± 0.1°C (CN). The rats were then anesthetized, pilocarpine (0.5 mg/kg) was intraperitoneally injected, and saliva was collected. Then, the submandibular glands (SMGs) were sampled. The salivary IgA concentration and IgA flow rate were significantly higher in the HE than in the CN. Similarly, the expressions of polymeric Ig receptor (pIgR), a mediator of mucosal IgA secretion, and Syndecan-1 (SDC-1), a useful biomarker for plasma cells, in the SMG were significantly higher in HE. The levels of interleukin (IL)-5 and IL-6, key cytokines of pIgR expression and plasma cell differentiation, were significantly greater in HE than in CN. Heat acclimation may enhance salivary IgA secretion through an increase in pIgR expression and the number of plasma cells in the SMGs.

### [1P10-08]

### Quantitative analysis of the transcripts of Cold-inducible RNA-binding protein gene in hibernation-like alternative splicing

*Yuuki Horii[1], Monami Shiraishi[2], Takahiko Shiina[2], Yasutake Shimizu[2] ([1] *Institute for Glyco-core Research (iGCORE), Gifu Univ.,* [2] *Lab. Vet. Physiol., Fac. Appl. Biol. Sci., Gifu Univ.*)

We reported that the expression pattern of alternative splicing of Cold-inducible RNAbinding protein (CIRP) changes during hibernation in the hibernating animal Syrian hamster. In addition, it was possible to artificially induce a hibernation-like expression pattern in mice as well. We expected that changes in alternative splicing might increase the amount of functional CIRP mRNA. Therefore, we investigated changes in the expression level of CIRP transcripts depending on the expression pattern of the CIRP gene in ddY strain mice. Mouse blood was collected, kept warm at 37℃, 28℃ or 15℃, and its expression was analyzed by RT-PCR. Electrophoresis of PCR products detected splicing variants along with CIRP mRNA when warmed at 37℃ and 15℃.On the other hand, when the temperature was kept at 28℃, a hibernation-like splicing pattern was confirmed in which the expression was concentrated only on CIRP mRNA. Quantitative analysis by real-time RT-PCR showed no change in the total amount of CIRP splicing variants and CIRP mRNA at all temperature conditions. On the other hand, the expression level of CIRP mRNA was higher at 28℃ than under the conditions of 37℃ and 15℃. It was suggested that the change in hibernation-like alternative splicing may be a mechanism that rapidly increases the expression efficiency of CIRP mRNA without changing the transcription amount.

### [1P10-09]

### Estimating Heatstroke Risk Using Electrocardiogram Signals

*Takashi Maruyama[1], Yoichi Ueta[1] ([1] *Department Physiology, University of Occupational and Environmental Health, Japan*)

Heatstroke is caused by the body overheating, dehydration, hyponatremia and poor health conditions. Sometimes it will be a fatal state, so the prevention for heat stroke is important issue. Usually as a result of exercise or working in high temperatures environment, the risk of heat stroke rises. A total of 12 healthy male aged 21.64 years performed an ergometric exercise load test in an environmental chamber adjusted to a temperature of 35°C and humidity of 50%. During the exercise load test, electrocardiogram and core body temperature (rectal temperature) were continuously measured. Core body temperature is one of the diagnostic factors for heatstroke. The amount of change in core body temperature was estimated using multiple regression analysis in which indexes derived from electrocardiograms were treated as the dependent variables. Changes in heart rate were related to heat production and yielded valid results. Based on the findings, we developed a core body temperature estimation model using the electrocardiogram signals by the Poincaré plot method. This estimation model suggested that continuous core body temperature can be estimated using electrocardiogram signals regardless of individual characteristics such as age and physique. Using the Poincare plot method, this study proposed an algorithm that estimates core body temperature based on electrocardiogram signals.

### [1P10-10]

### Relationship between temperature and humidity of intake air and humid sensation

*Issei Kato[1], Yuta Masuda[1], Kei Nagashima[1] ([1] *Waseda University*)

[Background] Recent studies have suggested that the nasal mucosa is involved in the regulation of humidity in exhaled air. We hypothesized that nasal inhalation might be involved in the humid sensation in the environment. [Methods] 16 healthy male and female subjects performed controlled breathing through a mask that covered only the nasal area. The inhalation air was temperature of 25 or 34℃ and relative humidity of 30 and 70%RH conditions. The dew point temperature ($T_d$) was determined from the atmospheric pressure, temperature and humidity of the inhalation air. The experimental participants rated the sensation of humidity (wet or dry) as length from the left end using the Visual Analog Scale (VAS) with a 10 cm straight line. An experiment with the same condition was also conducted while menthol was sublimated in the mask (menthol condition; N=8). [Results] There was a significant positive correlation between $T_d$ and the humid sensation (r=0.4336, p<0.01). A significant positive correlation was also found between the thermal sensation of the intake air and the humid sensation (r=0.666, p<0.01). In the menthol condition, there was also a significant positive correlation between thermal sensation and humid sensation (r=0.535, p<0.01). [Conclusion] The results suggest that the thermal sensation in the nasal cavity during inhalation is an important factor in the formation of the humid sensation in humans.

### Poster Presentation 1

### [1P11]
### Embryology, Regenerative Medicine, Development, Growth, Aging, Medical education, Medical histology

March 16(Wed), 12:30 - 14:30, Zoom P11

### [1P11-01]
### Vitamin E and rapamycin treatment attenuate cellular senescence induced by prolonged disturbance of proteostasis in human fibroblast

*Yasuhiro Takenaka[1,2], Ikuo Inoue[2], Masaaki Ikeda[2], Yoshihiko Kakinuma[1] ([1]*Nippon Medical School,* [2]*Saitama Medical University*)

To investigate how prolonged disturbances of proteostasis is involved in cellular senescence process in proliferating cell, we have established a *in vitro* senescence model in which young normal human fibroblasts, MRC-5, were treated with either of a reversible proteasome inhibitor, MG-132, and V-ATPase inhibitor, Bafilomycin A1 (BAFA1). Either drug treatment caused excess production of mitochondrial superoxide and intracellular ROS, and induced temporal mitochondrial dysfunction, mitochondrial accumulation, and eventually stress-induced premature cellular senescence (SIPS). SIPS induction by MG132 or BAFA1 was partially attenuated by co-treatment with vitamin E or rapamycin, in which the levels of ROS, mitochondrial accumulation, and protein aggregates were suppressed, implying the critical involvement of oxidative stress and mitochondrial function in SIPS progression. Rapamycin co-treatment also augmented the expression of HSP70 and activation of AKT, which could recover proteostasis and promote cell survival, respectively. Notably, both VE and rapamycin co-treatments could not completely stop senescence progression. Thus, we anticipate that there are factors, yet to be identified, other than ROS or mitochondria, promoting cellular senescence.

### [1P11-02]
### Heat Shock Factor 1 is involved in the expression of Yamanaka factors in zebrafish retina after optic nerve injury

*Kayo Sugitani[1], Takumi Mokuya[1], Shuichi Homma[1], Kazuhiro Ogai[2], Yoshiki Koriyama[3] ([1]*Div Health Sci, Grad Sch Med Sci, Kanazawa Univ.,* [2]*AI Hospital/Macro Signal Dynamics Res. Dev. Center, Kanazawa Univ.,* [3]*Faculty of Pharm, Suzuka Univ. of Med Sci.*)

Unlike mammals, fish exhibit regenerative capacity in the central nervous system after injury. In zebrafish, the retinotectal connection is can be re-established in 20-30 days after optic nerve transection. Due to their high regenerative capacity, the zebrafish optic nerve has been used as a useful model for understanding the molecular mechanisms of the central nervous system regeneration. Here, we show that upregulation of oct4 (also known as pou5f1), sox2 and klf4 genes in zebrafish retina within several hours after optic nerve injury. These transcription factors are subsets of Yamanaka factors, known as being essential for producing induced pluripotent stem cells and as regenerative factors in the mouse optic-nerve crush-injury model. In similar time course of the expression of these Yamanaka factors, heat shock factor 1 (HSF1) also upregulated in zebrafish retina in a very early stage after nerve injury. Pretreatment of HSF1 morpholino injection to eyeball prior to optic nerve crush, significantly suppressed the expression of oct4, sox2 and klf4 as well as downregulation of HSF1 in the zebrafish retina. These results indicate that the three Yamanaka factors may be regulated by HSF1 expression in early stage of optic nerve regeneration.

### [1P11-03]
### Establishment of methods to analyze the mechanism of fetal liver growth after blood perfusion

*Yoshiki Kuse[1], Erica Carolina[1], Shinya Matsumoto[1], Tomomi Tadokoro[2], Yasuharu Ueno[1], Hideki Taniguchi[1] ([1]*Division of Regenerative Medicine, Center for Stem Cell Biology and Regenerative Medicine, The Institute of Medical Science, The University of Tokyo,* [2]*Department of Regenerative Medicine, Yokohama City University Graduate School of Medicine*)

All tissues of the adult are efficiently supplied with oxygen and nutrients through blood circulation to maintain their functions. The fetus is received oxygen and nutrients in maternal blood through the placenta from the uterine artery. Placenta-derived blood first passes through the liver. It has been reported that impaired uteroplacental blood flow can affect the liver size. However, the mechanism of liver growth after blood perfusion is still unknown. In this study, we established a method to investigate the relationship between blood perfusion and hepatoblast proliferation in the liver. Perfusion of fluorescent-labeled CD31 antibody through umbilical vein visualized the blood perfused vessels in the liver. We clarified that embryonic day 10.5 (E10.5) was the stage of initiating blood perfusion using this method. Liver volume was increased after blood perfusion, and we confirmed hepatoblast proliferation in the vicinity of perfused vessels. Furthermore, we found that blood flow suppression by uterine artery ligation impaired the liver growth and formation of the blood vessel network at E10.5 but not E9.5. In summary, we demonstrated that blood perfusion derived from the placenta contributed to liver growth. In the future, we can apply these results to elucidate the mechanism of organ size regulation by blood perfusion.

### [1P11-04]
### Development of liver tissue reconstruction method using human iPS cell-derived liver organoids and decellularized organ.

*Souichiro Yamabe[1], Yoshiki Kuse[1], Megumi Matsuo[1], Takashi Okumura[1], Toshiharu Kasai[1], Kotaro Nishi[2], Tomonori Tsuchida[2], Toshinori Morisaku[2], Hiroshi Yagi[2], Hideki Taniguchi[1] ([1]*Division of Regenerative Medicine, Center for Stem Cell Biology and Regenerative Medicine, The University of Tokyo,* [2]*Department of Surgery, School of Medicine, Keio University*)

The decellularized organ retains the three-dimensional extracellular matrix and vascular structures of organ. Recent report shows that recellularized liver using hepatocytes exerts liver specific function after transplantation. However, the vascular structures are not reconstructed, and the function is limited after transplantation in conventional recellularized liver. In our laboratory, human iPS cell-derived liver organoids (hiPSC liver organoids) are generated (Takebe *et al. Nature* 2013, Takebe *et al. Cell Rep* 2017). Our hiPSC liver organoids have the vascular network and show multiple liver function. In this study, we challenged to infuse hiPSC liver organoids into the decellularized liver. hiPSC liver organoids were created by seeding the hepatic endoderm/endothelial cell/mesenchymal cell in patterned microwell plates. Cultured hiPSC liver organoids were infused after connection of the perfusion culture device to the rat decellularized liver. After 3 and 7 days of perfusion culture, histological and functional analyses were performed. Rcellularized liver infused with hiPSC liver organoids showed higher filling rate and higher human ALB production compared to the three types of cell-infused group. Human vascular structures were also observed in hiPSC liver organoid-infused group. By prolonging the perfusion culture period, human ALB production was increased. It was suggested that hiPSC liver organoids could be useful for liver reconstruction using decellularized liver.

### [1P11-05]
### Regulation of clock genes expression in human induced pluripotent stem cells and its application to directed differentiation

*Hitomi Kaneko[1], Taku Kaitsuka[2], Kazuhito Tomizawa[1] ([1]*Department of Molecular Physiology, Faculty of Life Sciences, Kumamoto University,* [2]*School of Pharmacy at Fukuoka, International University of Health and Welfare*)

It is important to elucidate the physiology of human induced pluripotent stem cells (hiPSCs) for directed differentiation by mimicking embryonic development. There are several reports showing that synchronized circadian rhythm is involved in the maturation of developing organs, but no protocols have been reported in which the circadian rhythm of hiPSCs is considered in differentiation steps. This may be due to a reason that circadian rhythm of clock genes expression is generally known to be absent in pluripotent stem cells (PSCs). In this study, we aim to clarify why such circadian rhythm is not oscillated in hiPSCs and to develop a novel protocol for directed differentiation by an use of the circadian rhythm. We speculated that one of the reasons for the absence of circadian rhythm in hiPSCs could be transcriptional repression of clock genes caused by hypermethylation of histone H3 at lysine 27 (H3K27), and another one is due to the low protein levels of BMAL1. Therefore, we generated BMAL1 overexpressing cells and pretreated those cells with GSK126, an inhibitor of EZH2 which is a methyltransferase of H3K27. As a result, significant circadian rhythms of BMAL1 and PER2 expression were observed by those two factors, suggesting candidate mechanisms of no rhythmicity of clock genes expression in hiPSCs. The effects of synchronized circadian rhythm on differentiation efficiency into endoderm lineages is under investigation.

### [1P11-06]
### Tumor suppressor homologue *let-7* is regulated across generations by starvation in *C. elegans*.

*Luna Izuhara[1], Sawako Yoshina[1], Shohei Mitani[1] ([1]*Tokyo Women's Medical University*)

*let-7* is a microRNA found in *C. elegans*, whose human homologue functions as a tumor suppressor. Recent studies in *C.elegans* suggest an intrinsic strategy in which parental experiences during developmental stages form transmissible epigenetic memories, that elicit enhanced robustness and viability in their descendants. In this study, we investigated the transgenerational inheritance of the expression control of the nematode homologous gene *let-7*. We used a temperature-sensitive *let-7* mutant allele, which has sterile and vulval malformation at the restrictive temperature. We found that starvation suppresses the phenotypes of *let-7* mutant animals. Also, starvation changes the expression stage of *let-7*; this effect is inherited through the F4 generation. In addition, we found that downregulating the expression of certain genes involved in epigenetics repressed the infertility phenotype of *let-7* mutant animals. Thus, the regulation of *let-7* expression suggests that dependence of this microRNA on epigenetic regulation. It is of great interest how food-deprivation causes the epigenetic modulation of the *let-7* gene expression to be studied.

### [1P11-07]
### Properties of 3D-spheroid with vascular smooth muscle cells derived from rat ductus arteriosus

*Takahiro Inoue[1], Nur Khatijah Mohd Zin[1], Hiroki Bochimoto[1], Toru Akaike[1], Susumu Minamisawa[1] ([1]*Department of Cell Physiology, The Jikei University School of Medicine*)

**Background:** Recently, characters of vascular smooth muscle cells (VSMCs) in ductus arteriosus (DA) have been well demonstrated. However, the main problem of 2D-monolayer culture is to substantially differ in the environment from real in-vivo tissue.
**Materials and Methods:** VSMCs were isolated from DA and descending thoracic aorta (dAo) of Wistar rat fetuses at 21 gestational age and cultured in DMEM with/without 10% fetal bovine serum (FBS). After passages 4 to 6, 3D-spheroids ($4x10^4$cells/spheroid) were inducted in 96 wells with an U-shaped bottom and maintained for 7 days to assess properties of each spheroid.
**Results:** On day 7 after spheroid induction, spheroids from VSMCs cultured without FBS for 2 days before induction were well aggregated and bigger compared to those with 10% FBS. There was no significant difference in the size between DA and dAo spheroids at the endpoint. Following application of PGE1, the surface of DA spheroid was rough with bumps and dips at 6 hours, but it altered smooth with uniform formation at 48 hours. Production of hyaluronic acid per unit number of cells was comparable between 3D-spheroids and 2D-cells. On real-time PCR analysis, AP-2 beta and EP4 expression were significantly greater in DA than dAo spheroids, which were similar expression patterns both in cells and in tissues.
**Conclusion:** To our knowledge, this is the first study to create spheroids with single vascular smooth muscle cells in ductus arteriosus. Spheroid of DA-VSMCs can be a plausible bridge of major gap between 2D and in-vivo tissue and has more potential to clarify the mechanism of opening/closing of DA.
(COI:No)

### [1P11-08]
### Inhibition of N-myristoyltransferase Promotes Naive Pluripotency in Mouse and Human Pluripotent Stem Cells

*Kyoji Horie[1] ([1]*Department of Physiology II, Nara Medical University*)

Naive and primed states are distinct states of pluripotency during early embryonic development that can be captured and converted to each other in vitro. To elucidate the regulatory mechanism of pluripotency, we performed recessive genetic screening of homozygous mutant mouse embryonic stem cells (mESCs) and found that N-myristoyltransferase (Nmt) suppression promotes naive pluripotency. The disruption of Nmt1 in mESCs conferred resistance to differentiation, and Nmt suppression in mouse epiblast stem cells (mEpiSCs) promoted the conversion from the primed to the naive state. This effect was independent of Src, which is a major substrate of Nmt and is known to promote mESC differentiation. Nmt suppression in naive-state human induced pluripotent stem cells (hiPSCs) increased the expressionof the naive-state marker. These results indicate that Nmt is a novel target for regulating naive pluripotency conserved between mice and humans.

The author has no conflict of interest to disclose with respect to this presentation.

### [1P11-09]
### Regulation of the outer blood-retina barrier with mesenchymal stem cell derived extracellular vesicles

*Hisaki Hayashi[1], Motohiko Sato[1] ([1]*Department of Physiology, Aichi Medical University*)

There is growing evidence that LED blue light, with a wavelength between 380 and 450 nm, exposure causes severe stress on eyes. Blood-retina barrier is essential for maintenance of retinal homeostasis, whose disruption is associated with retinal edema or age-related macular degeneration. On the other, mesenchymal stem cells (MSC) have been intensively investigated for regenerative cell therapies at present. Extracellular vesicles (EVs) derived from MSC has also recently reported to keep a potential to regenerate damaged organs as well; however, roles of MSC derived EVs on blood-retina barrier has not been known yet. Here, we investigated an effect of EVs derived from MSC on blue light irradiation induced retinal barrier damage by measuring trans-epithelial electrical resistance (TEER) of retinal pigmental epithelial cell (RPE) on Transwell. The barrier function, represented by TEER, was significantly reduced by blue light irradiation. Interestingly, treatment of EVs from MSC recovered TEER. These observations suggested the presence of key molecules for the blood-retina barrier recovery enclosed in EVs from MSC.

### [1P11-10]
### Positive effect of extending the course duration on dissemination of educational content

*Fuminobu Tamalu[1], Hiromasa Satoh[1], Narumi Hirosawa[1], Hajime Hirasawa[1], Mitsuo Nagane[1], Ryohei Saito[1], Shu-Ichi Watanabe[1], Naofumi Miwa[1] ([1]*Saitama Med. Univ.*)

To our knowledge, many medical universities incorporate a physiological practice course in their curricula. Despite all the benefits of this course, proper organization is quite problematic due to time restrictions, the limited number of skilled instructors, and insufficient laboratory instruments. Therefore, the current challenge for the academic staff is finding effective ways to develop the course (e.g., extending the course time length) in order to maximize student' s learning outcomes. In this study, we changed the schedule, evaluated the self-administered questionnaires between two different years (pre/postchange), and examined whether the increased course time (from one to two days per theme) improved and/or affected students' learning outcomes, including their interest, understanding, and communication. Consequently, there was a slight increase in content understanding within the 2d course despite minor differences in the average Likert scores between the two courses. Further, we found a profound reduction in the SD values for every question in the 2d course, indicating that the educational content was imparted more efficiently to students in the 2d course. Thus, we concluded that extending the course time facilitated dissemination of educational content.

### [1P11-11]
### Approaches to health education for the general public and children based on physiology

*Akihiro Hazama[1] ([1]*Department of Cellular and Integrative Physiology, Fukushima Medical University, School of Medicine*)

Currently, in Japan, primary and secondary education textbooks teach the mechanisms of the human body in science classes, diseases in health classes. However, I have found in my classes for university students that young people who have graduated from high school do not retain much knowledge about diseases. We believe that this problem is due to the fact that they only learn the names of diseases without sufficiently connecting them to the mechanisms of the human body. In order to solve this problem as much as possible, we have started to provide health classes based on physiology to children and the general public. In this effort, we are asking young people who are studying medicine, such as medical and nursing students, to cooperate as teachers. We would like to introduce this initiative.
There is no conflict of interest regarding the content of the presentation.

### Poster Presentation

### Day 2
### (March 17, 12:00～14:00)

[2P01] Ion channels, Receptors
[2P02] Ion channels, Receptors
[2P03] Motor function
[2P04] Glia, Plasticity, Neurochemistry
[2P05] Sensory function, Sensory organ
[2P06] Circulation
[2P07] Respiration, Digestion, Digestive system
[2P08] Behabior, Biological rhythm, Sleep
[2P09] Behabior, Biological rhythm, Sleep, Pathophysiology
[2P10] Pathophysiology
[2P11] Undergaraduate students

### Poster Presentation 2

### [2P01]
### Ion channels, Receptors

March 17(Thu), 12:00 - 14:00, Zoom P1

### [2P01-01]
**Phosphoinositide-dependent modulation of the structural rearrangements of Two-pore channel 3 evoked by depolarization**

*Ki-ichi Hirazawa[1,2], Michihiro Tateyama[1,2], Takushi Shimomura[1,2], Yoshihiro Kubo[1,2] ([1] *National Institute for Physiological Sciences,* [2] *The Graduate University for Advanced Studies*)

Two-pore channel 3 (TPC3) is a voltage-gated cation channel and its polypeptide consists of two repeats of canonical 6 transmembrane motif. The 4th helix in the 2nd repeat (the 2nd S4) in TPC3 is important for sensing membrane voltage, while phosphoinositide (PI) binds to the 1st repeat to potentiate the voltage dependence of TPC3. To reveal the effect of PI on the structural rearrangement of the 2nd S4, we performed voltage clamp fluorometry of TPC3 that was labeled by fluorescent molecules at the top (Q507) or at the bottom (S527) of the 2nd S4 using *Xenopus* oocyte expression system. The structural rearrangements of the 2nd S4 were successfully detected as the fluorescence intensity changes (F changes). The F change-voltage relationship showed biphasic change. Gating charge measurement revealed that the second phase in the F change corresponds to the gating charge movement. PI binding potentiated the voltage-dependence of the second phase in the F changes. In addition, the accessibility of a covalent modifier of Cys (MTSES) to the introduced Cys at the top of the 2nd S4 (D511C) was analyzed by standard two electrode voltage-clamp technique. Both depolarization and PI binding accelerated the MTSESmodification, showing that the top of the 2nd S4 becomes more exposed to the extracellular medium by these stimuli. Taken together, we conclude that PI binding to the 1st repeat can potentiate the structural rearrangements of the 2nd S4.

### [2P01-02]
**The 4th transmembrane domain of THIK-1 channel plays critical roles in the regulation of the channel activity**

*Michihiro Tateyama[1,2], Kubo Yoshihiro[1,2] ([1] *Dev. of Biophysics and Neurobiology, National Institute for Physiological Sciences,* [2] *Dept. of Physiological Sciences, SOKENDAI*)

A member of two-pore domain $K^+$ (K2P) channels, THIK-1, is known to be activated by Gi/ o coupled receptors. We previously found that THIK-1 is also activated by Gq-coupled receptors. In K2P channels other than THIK-1, the 4th transmembrane (TM4) domain is known to be a critical domain for the regulation of the channel activity. Here we investigated the role of the THIK-1 TM4 domain in the channel activity by introducing 4 amido-phenyalanine (AzP), which becomes highly reactive upon UV-exposure, to bulky residues at the lower part of TM4, one at a time. Stimulation of Gq-coupled adrenergic receptor α1A-AR increased the current amplitudes of eleven AzP mutants but it slightly decreased that of V278AzP, suggesting a critical role of Val278 in the Gq-R dependent activation. UV-exposure increased the current amplitude of eleven AzP mutants and decreased that of L275AzP channel, possibly through the UV-induced cross-linking reaction which locked the channel in a more or less conductive conformation, respectively. Stimulation of α1A-AR after UV-exposure failed to increase the current amplitude of three 4AzP mutants that showed a UV-induced marked current increase, suggesting that a highly conductive conformation is locked in the three mutants. Taken together, this study showed that the twelve bulky residues of TM4 play important roles in the regulation of THIK-1 channel activity.

### [2P01-03]
**Analysis of the relationship between channel opening and position of the second S4 helix in two-pore $Na^+$ channel 3**

*Takushi Shimomura[1,2], Ki-ichi Hirazawa[1,2], Yoshihiro Kubo[1,2] ([1] *Division of Biophysics and Neurobiology, National Institute for Physiological Sciences,* [2] *Department of Physiological Sciences, The Graduate University for Advanced Studies*)

Two-Pore $Na^+$ Channels (TPCs) contain two repeats of a unit consisting of 6 transmembrane helices. Domain I (DI) is responsible for the phosphoinositide (PI) binding, while DII plays a major role in the voltage sensing. We previously showed that, in *Xenpus* TPC3 expressed in *Xenopus laevis* oocyte, Phe514 in DII-S4 is in the close proximity to Glu438 in DII-S1 in the open state. We found in this study that TPC3 can open in a different conformation, with Phe514 close to Glu447 in DII-S2 that is located at a position more inside in the transmembrane region than Glu438. First, F514R showed a biphasic activation pattern in its voltage dependence, i.e., an additional component is observed at the very low membrane voltage along with the usual component. Noticeably, this component was lost in the absence of PI binding. Second, it was still observed in E438Q/ F514R mutant, but was lost in E447Q/F514R. Third, a disulfide bond formation by oxidization of E447C/F514C increased the amplitude of the basal current in the presence but hardly in the absence of PI. These results show that, in the presence of PI, TPC3 opens even when DII-S4 is located at a more downward position in the membrane. (COI: NO)

### [2P01-04]
**The voltage-gated proton channel is regulated by ATP**

*Akira Kawanabe[1], Maki Takara[1], Yuichiro Fujiwara[1] ([1] *Kagawa University*)

The voltage-gated proton channel (Hv1) transports protons across the cell membrane in response to the membrane potential [Sasaki et al. 2006]. The activity of Hv1 is regulated by various divalent cations and small molecules ($Zn^{2+}$, an inhibitor (2GBI), unsaturated fatty acids, etc.) [Kawanabe&Okamura 2016, DeCoursey 2018]. Adenosine-5'-triphosphate (ATP) is an important molecule that acts as an energy source and a modulator for a variety of proteins, including ion channels. In the case of Hv1, no meaningful ATP effects have been suggested [DeCoursey 2008]. However, our preliminary analysis showed that the proton current amplitude of Hv1 was increased by ATP. In this study, we analyzed the proton currents of mouse Hv1 using the inside-out patch-clamp recording technique. The Application of ATP to the cytoplasmic side of Hv1 influenced the amplitude of the proton currents and its effect was reversible. The robust increases of the Hv1 current with ATP analogs (ADP, AMP-PCP) were shown, suggesting that ATP directly regulated the Hv1 current. We will discuss the molecular mechanisms and physiological significance of these regulations.
COI: No

### [2P01-05]
**Gating properties of ion-conductive aquaporin 6 reconstituted in lipid bilayers**

*Takahisa Maki[1], Shigetoshi Oiki[1], Masayuki Iwamoto[1] ([1] *Fukui Univ.*)

Aquaporins (AQPs) facilitate passive water transport across biomembranes, and their biological functions have been vigorously studied. On the other hand, it is not elucidated whether water permeation is gated in AQP molecules. Among the AQPs, AQP6 possesses a unique property of conducting ions in addition to water. Such a feature of AQP6 suggests that its gating machinery for a water-conducting pore can potentially be evaluated from the ionic current. This study aims to elucidate the gating property of AQP6 by electrophysiological approach in reconstitution membranes. As the first step, we constructed an expression system of human AQP6 (hAQP6) protein using budding yeast. We successfully purified hAQP6 from the yeast membrane while retaining its water permeation activity. Next, we reconstituted the purified hAQP6 into the lipid bilayer. We succeeded in measuring the water permeability of reconstituted hAQP6 by the moving membrane method (Yano et al., *J. Membr. Sci.*, 2021). We are currently trying to measure the ionic current through hAQP6 in the lipid bilayer. Our further strategies for characterizing AQP using reconstituted membranes will be discussed.

### [2P01-06]
### Investigation of the interaction between TRPV3 and TMEM79 in mouse skin keratinocytes

*Jing Lei[1,2,3], Takeshi Matsui[4,5], Masayuki Amagai[4,6], Makoto Tominaga[1,2,3] ([1]*Div Cell Signal, NIPS,* [2]*Dept Physiol Sci, SOKENDAI,* [3]*Thermal Biol, ExCELLS,* [4]*Skin Homeost, RIKEN,* [5]*Evol Cell Biol of Skin, Sch of Biosci and Biotech, Tokyo Univ of Tech,* [6]*Dept Derm, Keio Univ Sch of Medicine*)

Itch-specific therapies of skin disease have been put in great effort to investigate the underlying mechanisms and potential targets. Cation-permeable transient receptor potential V3 (TRPV3) is predominantly expressed in skin keratinocytes, participating in physiological progress ranging from somatosensation to inflammation. TRPV3 level is increased in isolated keratinocytes from mouse model of atopic dermatitis (AD), a most common chronic skin disease with spontaneous itch and skin barrier disfunction, and gain of function mutation of TRPV3 is known to be related with a hereditary skin disease, Olmsted syndrome with severe itch. However, the underlying mechanism of how TRPV3 is implicated in itch signaling is still poorly understood. Interestingly, a less known transmembrane protein 79 (TMEM79) has been introduced to play roles for pathogenesis of AD in mice. These facts promoted us to consider a possible interaction between TRPV3 and TMEM79 in skin keratinocytes. In this study, we have found that mouse TMEM79 is capable of suppressing 2-APB-induced currents in HEK293 cells transiently expressing mouse TRPV3, indicating reduced mouse TRPV3 expression level on plasma membrane. Consistent with the results in HEK293 cells, 2-APB-induced mouse TRPV3 currents were larger in primary mouse keratinocytes when TMEM79 was deleted. This project will provide a novel insight into understanding the mechanism of itch based on the interaction between TRPV3 and TMEM79, which may lead to a novel therapy for itch and skin disease in the future.

### [2P01-07]
### Effects of the membrane thickness on the gating of the KcsA potassium channel

*Yuka Matsuki[1], Masayuki Iwamoto[1], Masako Takashima[1], Shigetoshi Oiki[1] ([1]*Univ. Fukui* )

The cell membrane contains various types of lipid molecules, and the physical property of the membrane varies substantially in the local chemical compositions of the membrane. Membrane proteins, such as ion channels, are subject to change their action under the influence of the surrounding membrane. At the membrane interface of a channel protein, the hydrophobic part of the transmembrane domain governs the thickness of the hydrophobic core of the membrane, leading to local thinking or thickening of the membrane (hydrophobic mismatch). Here, we examined the single-channel activity of the KcsA potassium channel, reconstituted into lipid bilayers with arbitrary lipid compositions. The contact bubble bilayer (CBB) method allows the formation of asymmetric membranes, and the thickness of the membrane was changed by using phosphatidylcholine with different lengths of acyl chains. The mean membrane thickness was evaluated from the specific capacitance of the membrane. We found that the open probability of the KcsA channel varied substantially depending on the membrane thicknesses. We will discuss the molecular mechanism of the membrane thickness-dependent gating of the KcsA channel.

### [2P01-08]
### Analysis of interaction between voltage-gated sodium channel Nav1.1 and fibroblast growth factor homologous factor

*Ikuo Ogiwara[1], Chengzhu Yin[1], Atsushi Shimohata[1], Mie Gangi[1], Makoto Kaneda[1] ([1]*Department of Physiology, Nippon Medical School*)

Nav1.1 is a brain type of voltage-gated sodium channel that plays critical roles in generation and propagation of action potentials. Loss of Nav1.1 function in GABAergic inhibitory neurons causes epilepsy with developmental delay. Fibroblast growth factor homologous factors (FGF11-14) are intracellular, non-secretory forms of FGF. The FGFs interact with the C-terminal domain of voltage-gated sodium channels, and modulate the subcellular localization and electrophysiological properties of the channels. A recurrent mutation of FGF12 is associated with epilepsy with developmental delay. We here show that Nav1.1 is co-precipitated with FGF in mouse brain lysate, and vice versa. While Nav1.1 contains a putative FGF-binding domain in its C-terminal domain, FGF seems not to bind to the C-terminal domain of Nav1.1. However, FGF interacts with Nav1.1 through the intracellular loop between the transmembrane domains of the channel. We are currently studying whether FGF affects the electrophysiological properties of Nav1.1. Our studies will contribute to the understanding of Nav1.1 and FGF12 in epilepsy with developmental delay, and also provide insights into such neurological disorders.

### [2P01-09]
### RNA-seq analysis focusing on ion channel genes and expression analysis of HCN channels in the heart of the ascidian *Ciona*

*Yuma Fujikake[1,2], Junko Nishino[1,2], Atsuo Nishino[1,2] ([1]*The United Graduate School of Agricultural Science, Iwate University,* [2]*Faculty of Agriculture and Life Science, Hirosaki University*)

Ascidians are marine invertebrates that constitute the sister group of vertebrates in the phylum Chordata. The ascidian heart is composed of a contractile tube called the "heart tube". The ends of the tube are called the hypobranchial end (H end) and the visceral end (V end), respectively. Heart-beatings in the tube are represented by peristaltic contractile waves which are initiated at one of the ends and propagate to the other. An interesting unsolved puzzle is that the direction of the peristaltic heart-beating periodically reverses, such as from "H to V" to "V to H". Although past studies revealed that the heart-beat reversals are caused by intrinsic pacemaker ability within the heart, not by any external factors, details of the mechanism have remained unknown. Our recent analyses on the heart of the ascidian, *Ciona*, revealed that two independent populations of pacemaker cells reside within a 5% region at each end of the heart tube (called P regions) (Fujikake and Nishino, 2018, 2021, Annual Meetings of Zoological Society of Japan). In order to comprehend the molecular mechanisms of the heart-beat reversals, we intend to identify the factors that allow the $P_H$ and $P_V$ cells to shoot periodic action potentials. We collected the $P_H$, $P_V$, and non-P regions of the Ciona heart and performed RNA-seq analyses. We quantitatively evaluated the expression of ion channel genes in those three tissues and found some factors that were highly expressed in both or either of the $P_H$/$P_V$ regions, or in the non-P region. The factors with higher expression in one of the P regions included genes related to HCN channels (called here *Ci-HCNa,b,c*), which play a crucial role in the generation of autonomous action potentials in the mammalian cardiac pacemaker cells. In our RNA-seq data, *Ci-HCNa* and *b* had higher expression levels in the $P_V$ region, while *Ci-HCNc* had a higher expression level in the $P_H$ region. Our *in situ* hybridization analysis revealed the localized expression of *Ci-HCNc* at the end of the $P_H$ region with a ring-like pattern. Our analyses highlight the comparable features between the heart-beating mechanisms in ascidians and that in vertebrates.

### Poster Presentation 2

### [2P02]
### Ion channels, Receptors

March 17(Thu), 12:00 - 14:00, Zoom P2

### [2P02-01]
**Electrophysiological study of PI(4,5)$P_2$ sensitivity of $GABA_A$ receptor channels in Xenopus oocyte**

*Risa Marie Mori[1], Shunichi Sugimoto[1], Daisuke Yoshioka[1], Takafumi Kawai[1], Yasushi Okamura[1] ([1]*Department of Integrative Physiology, Graduate School of Medicine, Osaka University*)

γ-Aminobutyric acid type A receptors ($GABA_ARs$) are pentameric ligand-gated ion channels that are the major mediators of fast inhibitory synaptic currents in the central nervous system. Recent cryo-EM structure of α1β3γ2L type $GABA_AR$ revealed phosphatidylinositol-4,5-bisphosphate, or PI(4,5)$P_2$, bound to the cytosolic side of the α1 transmembrane domain. However, the functional significance of PI(4,5)$P_2$ in $GABA_A$ Rs remains unresolved. Here we show that in a heterologous expression system, PI(4,5)$P_2$ regulates GABA-evoked currents of α1β3γ2L. Two-electrode voltage clamp recordings demonstrated that the depletion of endogenous PI(4,5)$P_2$ in *Xenopus* oocytes via the activation of ascidian voltage-sensing phosphatase (Ci-VSP) results in transient reductions of heterologously co-expressed mouse α1β3γ2L currents. When we mutated positively charged residues in the α1 subunit that interact with PI(4,5)$P_2$, Ci-VSP-induced current reduction became more remarkable. Thus far, our current data suggest that the mutation reduces the PI(4,5)$P_2$ binding affinity of α1β3γ2L type $GABA_ARs$, and that PI(4,5)$P_2$ binding in α1β3γ2L mediates GABA-evoked currents.

### [2P02-02]
**Elucidation of the regulatory mechanism of sensory proteins via modulations of membrane phospholipid**

*Takuto Suito[1], Takaaki Sokabe[1,2], Makoto Tominaga[1,2] ([1]*Div. of Cell Signaling, NIPS,* [2]*Thermal biology, ExCELLS*)

Thermo- and mechano-sensation is the essential biological function for collecting the information from external environments. Various sensory receptor proteins such as transient receptor potential (TRP) channel have been identified, however, other regulatory components for the sensory function remain to be elucidated. In this study, we focused on the function of lipid molecules in the sensory functions. We performed a transcriptome analysis in TRP channels-expressing thermo- and mechano-sensory neurons in Drosophila to identify the lipid metabolic genes expressed in these neurons. We observed the one of the genes involved in the phospholipids synthesis was highly expressed in the sensory neuron. Next, we established the phospholipid-engineered cell lines by knocking-out or overexpression of the phospholipid metabolic gene and analyzed the effect of modulation of the phospholipids on the TRP channel. We also analyzed Thermo- or mechanoreceptive behaviors in *Drosophila* and addressed the effects of the phospholipid metabolic gene knock down. We will discuss the significance of the membrane phospholipids in sensory functions based on the results of both cellular and behavioral analyses in *Drosophila*.

### [2P02-03]
**Intracellular ATP regulates the TRPV1 channel activity via the phosphoinositide signaling**

*Takahiro Shimizu[1], Nobuhiro Yanase[1], Takuto Fujii[1], Haruka Sakakibara[1], Hideki Sakai[1] ([1]*Dept. Pharm. Physiol., Fac. Pharm. Sci., Univ.*)

Transient receptor potential vanilloid 1 (TRPV1) is a $Ca^{2+}$-permeable non-selective cation channel activated by various physical and chemical stimuli. In whole-cell patch-clamp recordings using TRPV1-overexpressing HEK293T cells, basal currents showing outwardly rectification were run down in the absence of intracellular ATP. Intracellular ATP (0.5.5 mM) concentration-dependently induced the basal TRPV1 currents at room temperature in the absence of capsaicin. The rundown of TRPV1 currents under the ATP-free conditions was not improved by intracellular application of 2 mM AMP-PNP, a non-hydrolyzable ATP analog. Interestingly, intracellular application of 20 μM PI(4,5)$P_2$ generated TRPV1 currents in the absence of ATP. In fact, the rundown was observed in the presence of 2 mM ATP plus 300 μM LY294002, a PI4K inhibitor. On the other hand, activation curves of the TRPV1 channel were markedly shifted to a negative direction by intracellular ATP in a concentration-dependent manner. These results suggest that ATP does not directly affect the basal activity of the TRPV1 channels and that ATP-mediated phosphoinositide production contributes to the voltage-dependent gating of the TRPV1 channels.
COI: No

### [2P02-04]
**Functional rescue for the second and third most frequent diseaseassociated CFTR mutants in Japanese CF patients by the therapeutic drug for Caucasian mutants**

*Yoshiro Sohma[1], Rio Kimishima[1], Hikaru Sohma[1], Masahiro Shimizu[1], Shiori Ohkawa[1], Yuka Matsuzawa[1], Suzuna Kaneko[1], Yuki Fukada[1], Kanako Wakabayashi-Nakao[1] ([1]*International University of Health and Welfare*)

Cystic Fibrosis (CF) is the most popular, life-shorten, inheritance disease in Caucasians, which is caused by function-loss mutations in Cystic Fibrosis Transmembrane conductance Regulator (CFTR). The most frequent mutation in Caucasians is F508del classified into the class II (trafficking defect). Recently a few chemical chaperons (correctors) for rescuing the F508del mutant from the trafficking defect by Vertex Inc and approved by FDA in USA. CF is relatively rare in Japanese but does exist. The CFTR mutation profiles in Japanese are different from those in Caucasians. At present, twenty-two mutations were identified from twenty-four Japanese CF patients definitely diagnosed. The first to third most frequent Japanese CF mutations, Δ(G970-T1122), H1085R and L441P are all classified into class II. Our previous study found that unfortunately the most frequent Δ(G970-T1122)-mutant could not be rescued by the Vertex correctors. In this study, we found the Vertex correctors were succeeded to partly rescue H1085R- and L441P-CFTR, and investigated the function-loss mechanisms in these Japanese mutants.
(COI:No)

### [2P02-05]
**Investigation of the possible relation between TRPM2- ion channels**

*Aykut Deveci[1] ([1]*National Institute for Physiological Sciences (NIPS)*)

Divalent cation calcium ($Ca^{2+}$) is described as one of the most important biological cation. It is used by all living cells as an intracellular signaling messenger that controls many biological processes. It is also involved in the pathophysiology of the cells and cell fate. $Ca^{2+}$ is maintained at low levels in the cytosol and is mainly concentrated outside the cell or in intracellular compartments, particularly in the endoplasmic reticulum (ER), mitochondria and Golgi apparatus. Fluxes of $Ca^{2+}$ through the membranes are provided by transporters and ion channels present at the plasma membrane and the intracellular compartments.Among these, TRP Melastatin 2 (TRPM2) is highly expressed in several tissues. It is a $Ca^{2+}$- permeable, non-selective cation channel which exhibits heat sensitivity. It acts as a biosensor of oxidative and osmotic stresses under physiological and pathological conditions. Moreover, $Ca^{2+}$ influx induced by TRPM2 could activate other $Ca^{2+}$-dependent channels like intermediate $Ca^{2+}$-activated potassium channel ($IK_{Ca}1$). Indeed, $IK_{Ca}1$ is activated through calcium binding on calmodulin located on the C-Terminal of this channel. I will show the interaction between TRPM2 and $IK_{Ca}1$ in both HEK293T cells and native cells.

### [2P02-06]
### Identification of amino acids determining the temperature thresholds for heat-evoked activation of mosquito TRPA1

*Hong Dung Thi Nguyen[1], Stella Chapman[2], Claire Saito[1], Tatjana Strom[1], Mio Yasui[3], Makoto Tominaga[1] ([1]*National Institute for Physiological Sciences,* [2]*The University of Tokyo,* [3]*Nagoya City University School of Medicine*)

The mosquito is the world's most important vector for transmission of infectious diseases in tropical and subtropical regions due to its strong drive for blood-feeding. Female mosquitoes utilize multiple cues for their host-seeking behaviors such as $CO_2$, odors, and warm temperatures. Although mosquitoes seek for warm temperatures of homeothermic animals, they avoid noxious high temperatures which is sensed by TRPA1. A recent study by Li *et al.* characterized the properties of TRPA1 channels of several mosquito species and found that they exhibit species-specific difference in temperature thresholds for heat-evoked activation. Temperature thresholds for heat-evoked activation of TRPA1s of mosquitoes inhabiting the tropical regions are higher that that of TRPA1 of mosquito inhabiting the temperate regions. Here we discovered that the N-terminus plays a very important role to determine temperature thresholds for heat-evoked activation of TRPA1 of *Aedes* aegypti, mosquito in the tropical regions and *Culex pipiens pallens*, mosquito in the temperate regions. We also attempted to identify several amino acids in the N-terminus involved in the thermosensitivity of mosquitoes TRPA1. Identification of the amino acids might provide us with a clue to understand the structural basis for the mechanisms of heat-evoked activation of TRPA1 in the future.

### [2P02-07]
### Effect of hydrogen sulfide on the voltage dependence of hyperpolarization-activated current ($I_h$) in cultured rat dorsal root ganglion neurons.

*You Komagiri[1] ([1]*Department of Physiology, School of Medicine, Iwate Medical University*)

Hydrogen sulfide ($H_2S$) and Hyperpolarization-activated cyclic nucleotide-gated (HCN) channels are both thought to be involved in the generation of chronic pain after nerve injury. Although $H_2S$ has been reported to modulate adenylate cyclase and NOS activity, no study has investigated whether $H_2S$ affect HCN channel activation. In this study, the effect of NaHS, an $H_2S$ donor on the voltage dependence of the HCN channel currents ($I_h$) in cultured rat dorsal root ganglion neurons were examined with gramicidin perforated patchclamp technique. When NaHS was applied extracellularly, the half-maximal activation voltage of $I_h$ was shifted about 10 mV toward positive potential. This shift in the voltagedependence of $I_h$ was observed regardless of the sensitivity to capsaicin in cultured rat DRG neurons. SQ-22536, an adenylate cyclase inhibitor did not affect the NaHS-induced activation of $I_h$. These results suggest that $H_2S$ regulates the voltage-dependence of $I_h$ activation without involvement of adenylate cyclase activity in rat DRG neurons. I will further examine the mechanisms underlying the $H_2S$-induced activation of $I_h$.

### [2P02-08]
### Olfactory receptor 78 was expressed in hypothalamic supraoptic/paraventricular nuclei and choroid plexus in the mouse brain

*Noriyuki Nakashima[1], Akiko Nakashima[1], Kie Nakashima[2,3], Makoto Takano[1] ([1]*Department of Physiology, Kurume University School of Medicine,* [2]*Department of Physiology, Faculty of Medicine, Kyoto University,* [3]*Graduate School of Biostudies, Kyoto University*)

Olfactory receptors are comprised of hundreds of genes and respond to a variety of chemicals in olfaction. In addition to the canonical olfactory organ, olfactory receptors are expressed in several extra-olfactory organs and proposed to operate as local chemosensors. Olfactory receptor 78 (Olfr78), also known as prostate-specific G-protein coupled receptor (PSGR), is widely expressed in carotid body, kidney, colon, prostate gland and macrophages. However, the expression patterns of Olfr78 in the central nervous system remain much unknown. Therefore, we demonstrated immunohistochemistry to identify the expression of Olfr78 in the mouse brain. Olfr78 was expressed in the supraoptic nucleus and paraventricular nucleus of hypothalamus and choroid plexus. In the hypothalamus, Olfr78 was expressed in the vasopressin/oxytocin neurons. In the choroid plexus, Olfr78 was expressed in the fine laminar structure beneath the cuboidal epithelium, which was not immunoreactive towards vimentin, a marker for vascular smooth muscle cells. Considering those areas of the brain communicating with vasculature, Olfr78 could be involved in sensing the humoral conditions in the brain.

### [2P02-09]
### Sphingosine-1-Phosphate Induces ATP Release via Volume-Regulated Anion Channels in Breast Cell Lines

*Kishio Furuya[1], Hiroaki Hirata[1], Takeshi Kobayashi[1], Masahiro Sokabe[1] ([1]*Nagoya University, Graduate School of Medicine*)

High interstitial level of ATP and its lysate adenosine in the cancer microenvironment are considered a halo mark of cancer. Adenosine acts as a strong immune suppressor. However, the source of ATP release is unclear. We clarified the release of ATP via volume-regulated anion channels (VRACs) in breast cell lines using an ATP luminescence imaging system. We detected a slowly rising diffuse pattern of ATP release that was only observed in undifferentiated cells, not in differentiated primary cultured cells. This was confirmed by suppression with DCPIB, a blocker of VRACs, and shRNA for LRRC8A, an indispensable subunit of VRACs. We herein demonstrated that the inflammatory mediator sphingosine-1-phosphate (S1P), which exists abundantly in the cancer microenvironment, induced a diffuse pattern of ATP release isovolumetrically. The response was dose-dependent and suppressed by the knock-down of LRRC8A. It was also suppressed by blockers of S1P receptor 1 and 2 (W146 and JTE013, respectively). RTqPCR demonstrated the prominent presence of S1PR1 and S1PR2 mRNAs. We discussed the roles of S1P-induced ATP release in the cancer microenvironment.

### [2P02-10]
### Structural Mechanism underlying that hERG channel inactivation affects drug binding

*Kazuharu Furutani[1,2], Jan Maly[2], Vladimir Yarov-Yarovoy[2] ([1]*Tokushima Bunri University,* [2]*University of California, Davis*)

The voltage gated potassium channel, KV11.1, encoded by the human ether-a-go-go related gene (hERG) is expressed in cardiac myocytes, where it is crucial for the membrane repolarization of the action potential. hERG is implicated in a number of druginduced arrhythmias, caused by long QT syndrome. Gating of hERG is characterized by rapid, voltage-dependent, C-type inactivation, which blocks ion conduction and is suggested to involve constriction of the selectivity filter. To explore conformational changes associated with hERG inactivation, we use RosettaRelax to simulate the effects of hERG mutations. We show that a lateral shift of residue F627 in the selectivity filter into the central channel axis along the ion conduction pathway. Non-inactivating mutations S620T and S641T showed a potential blocking mechanism of F627 rearrangement, preventing it from shifting into the conduction pathway during the proposed inactivation process. Furthermore, drug docking results correlate well with existing experimental evidence of protein-ligand contacts between high-affinity hERG blockers and key residues Y652 and F656 inside the pore cavity, in addition to illuminating potentially new ligand binding interactions in the inactivated state fenestration region.

### Poster Presentation 2

### [2P03]
### Motor function

March 17(Thu), 12:00 - 14:00, Zoom P3

### [2P03-01]
### Suppression of the swallowing reflex by stimulation of the lateral reticular nucleus

*Tomohito Sakazume[1], Yoshihide Satoh[1], Arisa Murakawa[1], Shogo Ohkoshi[1] ([1]*Nippon Dental Univ.*)

The previous study reported that the swallowing reflex was suppressed by stimulation of the red nucleus. Morphological studies have demonstrated that the lateral reticular nucleus (LRN) receives projection fibers from the red nucleus. This study examines whether the swallowing reflex is modulated by stimulation of the LRN. These experiments were performed on rats anesthetized by urethane. The swallowing reflex was evoked by repetitive electrical stimulation of the superior laryngeal nerve (SLN) (0.2 ms duration, 30 Hz, for 10 s). The electromyogram was recorded from the mylohyoid muscle to identify the swallowing reflex. Frist, electrical stimulation was applied to the LRN. During recording sessions, the SLN and the LRN were simultaneously stimulated. As a control, the SLN was solely stimulated before and after the simultaneous stimulation. Second, sodium L-glutamate was injected into the LRN. The swallowing reflexes were recorded at 2 min before the injection and from 0 to 40 min after the injection. After each recording, the stimulus sites were confirmed histologically. The LRN stimulation had suppressive effect on the number of swallowing reflexes. The onset latency of the first swallow during simultaneous electrical stimulation of the SLN and the LRN was significantly increased. The present study suggests that the LRN is involved in the control of the swallowing reflex.

### [2P03-02]
### Neural network changes associated with training exercise in a hypoxic ischemic rat model

*Taichi Goto[1,2], Tomokazu Tsurugizawa[3], Yuji Komaki[4], Ichiro Takashima[1,2], Nobuo Kunori[2] ([1]*Master's and Doctral Programs in Neuroscience, Degree Programs in Comprehensive Human Sciences, Graduate School of Comprehensive Human Sciences, University of Tsukuba,* [2]*Integrative Neuroscience Research Group, Human Informatics Interaction Research Institute, National Institute of Advanced Science and Technology,* [3]*Mental and Physical Functions Modeling Group, Human Informatics and Interatcion Research Institute, National Institute of Advanced Industrial Science and Technology,* [4]*Live Imaging Center, Central Institute for Experimental Animals*)

Neonatal hypoxic ischemia (HI) is a major cause of cerebral palsy and often results in the sensorimotor impairment. Previous studies indicated the importance of plastic changes in the sensorimotor cortex after neonatal HI insult to improve the motor function. However, neural network changes associated with motor recovery is not well known. In this study, we performed diffusion tensor imaging (DTI) tractography in the neonatal HI animal model to compare the structural brain connectivity between the HI animals with and without training exercise. The HI model rats were trained to walk on rotarod to restore their sensorimotor function, and then whole-brain tissues were used for *ex vivo* DTI. In behavioral analysis after the training, the coordinated limb movement evaluated by rotarod test was significantly improved in the trained-HI compared to the untrained-HI. In tractography analysis, the intra-regional connectivity among the sensorimotor related brain areas, such as primary sensorimotor cortex and thalamus, were strengthened in the trained-HI. These results suggest that the dynamic changes in neural connectivity in sensorimotor related brain areas can contribute to motor recovery.

### [2P03-03]
### Neural dynamics of motor cortex of flexible motor selection

*Yosuke Kuroki[2], Ryota Masui[2], Tadashi Isa[2,3], Tomohiko Takei[1,3] ([1]*Brain Science Institute, Tamagawa University,* [2]*Faculty of Medicine, Kyoto University,* [3]*Graduate School of Medicine, Kyoto Univeristy*)

A hallmark of our motor system is how flexibly it can switch the association between sensory input and motor response. This association is called the "control policy" and is assumed to be prepared according to the behavioral context prior to the initiation of action, but the neural mechanism for the preparation of control policy in the motor cortex is still unclear. In this study, we constructed a recurrent neural network model that reproduces the flexible motor response of monkeys to mechanical perturbations applied to the limb, and analyzed the dynamics of the neural state of the network using principal component analysis (PCA). The results showed that the neural state of the trained network deviates in response to contextual signals and waits for the forthcoming mechanical perturbation. Then, after the mechanical perturbation was applied, the neural state deviated further to generate an appropriate motor output. Importantly, the trajectories of the neural states during the preparatory and response phases were orthogonally arranged, suggesting that the preparation of the control policy was achieved in a neural dimension separate from that for the motor output. To investigate whether a similar mechanism exists in the central nervous system of animals, we recorded electrocorticograms (ECoGs) from the primary and premotor cortices of macaque monkeys performing a flexible motor response task to a mechanical disturbance of the limb. The PCA showed that the cortical activity spanned orthogonal dimensions during the preparatory and response phases. These results suggest that the control policy is prepared as a state of motor cortical activity that is separated from the dimension of motor execution.

### [2P03-04]
### Modeling of motor adaptation process with an optimal control model

*Sadataka Fukui[2], Tadashi Isa[2,3], Tomohiko Takei[1,3] ([1]*Brain Science Institute, Tamagawa University,* [2]*Faculty of Medicine, Kyoto University,* [3]*Graduate School of Medicine, Kyoto Univeristy*)

Animals have a remarkable ability to adapt to changes in their environment and body dynamics. Previous studies have shown that motor adaptation can be viewed as an updating process of internal models. However, it is still unclear what neural mechanisms cause such update, especially in relation to online feedback control. In this experiment, we modeled trial-by-trial change in the movements of animals during motor adaptation by using an optimal feedback control (OFC) model. The monkeys were trained to reach a target (8 cm in front) in a constant lateral force field (1N) that started from 1 cm ahead of the starting position and continued until the end of the movement. The results of the OFC simulations showed that the adaptation process of the animal observed in reaching trajectory could be well reproduced as an update of a single parameter, the prediction of the external force. Furthermore, the estimate of external force was obtained through successive updating by sensory prediction error during online motor control. These results suggested that the online estimation and offline prediction of external force are used for motor adaptation in animals, and parameterizing them may be useful for revealing the neural correlates of motor adaptation.

### [2P03-05]
### Combination of Ninjin' yoeito and forced limb use improves motor function after internal capsule hemorrhage in rats

*Naoki Tajiri[1], Shinya Ueno[1], Takeshi Shimizu[1], Eisuke Haneda[2], Keita Mizuno[2], Hideki Hida[1] ([1]*Department of Neurophysiology & Brain Science, Graduate School of Medical Sciences & Medical School, Nagoya City University,* [2]*Tsumura Kampo Research Laboratories, Tsumura and Co*)

Ninjin'yoeito (NYT), a Japanese traditional Kampo medicine, is reported to have various effects on sarcopenia, cognitive dysfunction and so on. On the other hand, intensive forced limb use (FLU)-induced functional recovery was reported in internal capsule hemorrhage (ICH) model rats, showing a causal relationship between the cortico-rubral tract and the functional recovery. We tried to investigate whether the combination of NYT and FLU can promote motor function after ICH. Type IV collagenase (15 units/ml, 1.4 $\mu$l) was injected into the left internal capsule of male rats. One day after the surgery, FLU performed for 7 days and 1% NYT was mixed in chow and fed until 56 days. Five groups were prepared: sham-operated controls, ICH, ICH + FLU, ICH + NYT, ICH + FLU + NYT group. As behavioral evaluations, motor deficit score (MDS) was performed at 12, 20, 28 and 56 days, and horizontal ladder tests including gait time were tested at 28 days. The treated with FLU and NYT group showed significantly better behavioral recovery in MDS compare to ICH group at both 28 and 56 days. The horizontal ladder tests including gait time had a tendency to improve in the FLU and NYT group compared to ICH group at 28 days. These data suggest that NYT and FLU has a potency to improve deteriorated motor function in the rat ICH model. Evaluations for type of skeletal muscle and motor activities will be also presented to know the mechanism of NYT and FLU effects on ICH model.

### [2P03-06]
### Distinct network structures emerge in the primary motor cortex during active and quiet wake

*Takeshi Kanda[1], Takehiro Miyazaki[1], Kotaro Sakamoto[2], Hideitsu Hino[2], Masashi Yanagisawa[1] ([1]*University of Tsukuba,* [2]*The Institute of Statistical Mathematics*)

Active and quiet wake are two main states in wakefulness. These two states have distinct effects on brain functions, but how local cortical networks fluctuate during active and quiet wake is still unknown. Using calcium imaging and statistical machine learning, we investigated the differences between these two states and the homology with sleep states in local cortical functional connectivity. No large differences were observed in individual neural activity between active and quiet wake. Functional connectivity between neurons was sparse in active wake and dense in quiet wake. Unexpectedly, common neurons were connected to each other during quiet wake and NREM sleep. Similarity analysis of functional network structures revealed that, in local cortical networks, NREM sleep was similar to quiet wake, while REM sleep was quite different from both active and quiet wake. Our observations suggest that common functional networks emerging in quiet wake and NREM sleep support the common offline information processing in the brain.

### [2P03-07]
### The effects of zonisamide, an anti-parkinsonian drug, on L-DOPA-induced dyskinesia in Parkinson's disease model mice

*Hiromi Sano[1,2], Atsushi Nambu[1,2] ([1]*National Institute for Physiological Sicences,* [2]*SOKENDAI*)

Parkinson's disease (PD) is caused by the loss of dopaminergic neurons in the midbrain and exhibits motor dysfunctions. Zonisamide (ZNS) has beneficial effects on PD and has been approved as adjunctive therapy for PD. Here, we investigated the behavioral and physiological effects of ZNS on L-DOPA induced dyskinesia (LID) in 6-hydroxydopamineindued PD model mice. Acute ZNS administration to PD model mice during LID gives no effects on duration and severity of LID. However, chronic ZNS and L-DOPA administration to PD model mice increased LID duration. Then, we recorded neuronal activity in the substantia nigra pars reticulata (SNr), the output nucleus of the basal ganglia. In normal mice, motor cortical stimulation induces a triphasic response composed of early excitation, inhibition, and late excitation in the SNr. In PD model mice treated chronically with ZNS and L-DOPA, longer inhibition and reduced late excitation were induced. Inhibition in the SNr is derived from the direct pathway and releases movements, while late excitation is derived from the indirect pathway and stops movements. Thus, increased releasemovements- signals and decreased stop-movements-signals probably underlie increased LID duration with ZNS and L-DOPA.

### [2P03-08]
### Visuomotor transformation in the frontal lobe of a primate model of blindsight

*Saya Kitazume[1], Yusuke Yamamoto[1], Reona Yamaguchi[2], Tadashi Isa[1,2,3] ([1]*Dept Neuroscience, Grad Sch Med, Kyoto Univ.,* [2]*Institute for the Advanced Study of Human Biology (WPI-ASHBi), Kyoto Univ.,* [3]*Human Brain Research Center, Grad Sch Med, Kyoto Univ.*)

Some patients with damage to the primary visual cortex (V1) can respond to visual stimuli without consciously perceiving them. This phenomenon is called blindsight. The neural mechanisms involved in the control of visually guided saccadic eye movements in blindsight monkeys has largely been clarified, however those for the arm movement control are still elusive.
To address this issue, we examined the visuomotor transformation process of macaque monkeys with unilateral V1 lesion and surgically implanted electrocorticogram (ECoG) electrodes performing a two alternative forced choice manual response task. After the V1 lesion, time-frequency analysis of the brain activity showed that low frequency (8-13 Hz: alpha) band activity in dorsal premotor cortex (PMd) significantly increased when the monkey successfully detected the target (Hit trials), compared to the trials in which the monkey failed to detect the target (Miss and/or Error trials).
Granger causality analysis of the brain activity showed that the alpha-band connectivity and high frequency (121-150 Hz: high-gamma) band connectivity from PMd to primary motor cortex (M1) were greater in Hit trials compared to Error trials. These results suggest that the visual information for arm movement control arrives in the PMd and then prevails to M1 when the blindsight monkey successfully performed the manual response task.

### [2P03-09]
### Impaired information flow through the cortico-basal ganglia pathways is responsible for Parkinson's disease symptoms

*Satomi Chiken[1], Atsushi Nambu[1] ([1]*Division of System Neurophysiology, National Institute for Physiological Sciences*)

Parkinson's disease (PD) is a neurodegenerative disorder caused by progressive loss of nigrostriatal dopaminergic neurons and is characterized by motor and non-motor symptoms. To elucidate the pathophysiological mechanism underlying such symptoms, we examined neuronal activity in the internal segment of the globus pallidus (GPi), the output station of the basal ganglia, in PD monkeys. PD monkeys treated with MPTP, dopaminergic neurotoxin, exhibited obvious motor symptoms such as bradykinesia and rigidity. In healthy monkeys, motor cortical stimulation induces a triphasic response composed of early excitation, inhibition and late excitation in GPi neurons. However, in PD monkeys, cortically evoked inhibition in the GPi mediated by the cortico-striato-GPi *direct* pathway was largely diminished. L-DOPA treatment normalized cortically evoked responses and ameliorated PD symptoms. STN blockade by injection of muscimol, $GABA_A$ receptor agonist, unmasked cortically evoked inhibition and ameliorated the motor deficits. These results suggest that information flow through the *direct* pathway responsible for the initiation of movements is largely reduced in PD and fails to release movements, resulting in akinesia/bradykinesia, and that restoration of cortically induced inhibition in the GPi may have beneficial effects.

### [2P03-10]
### Flexible trunk control contributes to trunk-limb coordination during treadmill locomotion in Japanese monkeys : Kinematic and EMG analysis

*Takashi Suzuki[1], Kei Mochizuki[1], Kazunori Morita[1], Yoshiro Suzuki[1], Masahiko Inase[2], Katsumi Nakajima[1] ([1]*Iwate medical university,* [2]*Kindai university*)

Japanese monkeys can walk quadrupedally and bipedally on a treadmill. Dexterous control of the trunk is essential for this behavior. To investigate how the CNS transforms the trunk posture from horizontal to vertical during locomotion, we first analyzed kinematics and EMG activity of the trunk and limbs. During walking, the head and hip positions were cyclically modulated. For quadrupedal gait, these positions were affected by the fore- and hind-limb stepping, respectively. Bilateral back muscles were coactive only around the touchdown events. Contrarily, for the postural transformation, both the head and hip positions depended on the hindlimb stepping, representing trunk sway. Trunk EMG activity gradually increased, i.e., co-activation encompassed the entire step cycle and alternate bursts appeared in the left and right EMGs. Such enhanced EMG activity underlying the trunk sway continued into stable bipedal gait. The results suggest that, for controlling trunk posture during gait, outputs of the monkey's CNS are flexible and context-dependent to achieve trunk limb coordination. This could be an essential clue for understanding CNS mechanisms to integrate trunk and limbs of whole body movements.

### [2P03-11]
### Involvement of the cerebello-rubral tract in functional recovery by intensive use of the paralyzed side after cerebral hemorrhage

*Shinya Ueno[1], Takeshi Shimizu[1], Kenta Kobatashi[2], Naoki Tajiri[1], Hideki Hida[1] ([1]*Nagoya City University, Graduate school of Medical Sciences, Neurophysiology and Brain Science,* [2]*National Institute for Physiological Sciences, Section of Viral Vector Development*)

We reported that the motor function of the paralyzed limb improves after intensive use of the upper limb on the paralyzed side (rehabilitation) after intracerebral hemorrhage, and that the cortico-rubral tract is involved in this functional recovery (J Neurosci 36:455-67, 2016). However, the motor regulatory mechanisms in the recovery of upper limb function after rehabilitation have not been clarified. In the present study, we focused on the cerebellar output system in the lateral nucleus of the cerebellum to the parvocellular part of red nucleus (cerebello-rubral tract), and evaluated upper limb motor function by the DREADD method using double virus vector infection, in order to analyze the changes in motor regulatory systems caused by rehabilitation after cerebral hemorrhage.
To block the cerebello-rubural pathway, AAV-DJ-EF1a-DIO-hM4D(Gi)-mCherry and FuG-EMSCV-Cre were injected into the lateral nucleus of the cerebellum and the parvocellular red nucleus, respectively. This model was forced to use the paralyzed upper limb intensively for 1-8 days after hemorrhage, and the upper limb function was evaluated by single pellet reaching test after 12, 16, 20, and 28 days.
The success rate of the reaching test was improved in the rehabilitation group compared with the non-rehabilitation group. However, the success rate was significantly worsened by neuroleptic treatment with clozapine-N-oxide. Data suggest that the cerebello-rubral tract are involved in the improvement of impaired motor function by intensive use of the upper limb in rats.

### Poster Presentation 2

### [2P04]
### Glia, Plasticity, Neurochemistry

March 17(Thu), 12:00 - 14:00, Zoom P4

### [2P04-01]
### LINC complex regulates the plasticity of axon initial segment

*Koichi Hasegawa[1], Takeshi Matsui[1], Junpei Kondo[1], Sizuka Shoji[1], Noriyuki Hama[1], Ken-ichiro Kuwako[1] ([1]*Department of Neural and Muscular Physiology, Shimane University, School of Medicine*)

The axon initial segment (AIS) is located at the proximal site of axon in neurons and plays a pivotal role in initiation of action potentials. Recent studies have demonstrated that AIS alters its length, position and molecular composition in response to changes in neuronal activity level, indicating that AIS is a highly plastic structure in neurons. The LINC complex, composed of Sun1/2 and Nesprin1/2, plays an important role in connecting the nuclear membrane to the cytoskeleton. LINC complex mediates diverse cellular events, such as nuclear translocation and cell polarity. In the present study, we examined whether LINC complex regulates structural plasticity of AIS in mouse cortical neurons. The forced expression of dominant negative Nesprin-1 (Nesprin-1 DN), that strongly blocks the function of LINC complex, significantly shortened the AIS length in neurons. Furthermore, Nesprin-1 DN-expressing neurons lost the chronic depolarization-induced structural plasticity of AIS. These results indicate that LINC complex is essential to the formation and maintenance of AIS as a linker protein between the nuclear envelope and axon.

### [2P04-02]
### Plastic changes in activities of bitter taste-relaying neurons in the medial amygdala during conditioned taste aversion learning

*Makoto Sugita[1], Chieh-Mei Chang[1] ([1]*Department of Physiology and Oral Physiology, Graduate School of Biomedical and Health Sciences, Hiroshima University*)

An animal tasting saccharin, a novel sweet tastant (conditioned stimulus [CS]), and followed by intraperitoneal injection of lithium chloride (unconditioned stimulus [US]) can acquire one-trial learning of conditioned taste aversion (CTA) to that particular sweet tastant. The association of CS and US in the neurons in the basolateral amygdala (BLA) appears to be required for acquisition of CTA memory by inducing subsequent plastic changes in downstream neurons. However, it remains unknown how the BLA neurons receiving both the CS and US induce the plastic changes in downstream neurons. Here we combined genetic tracing and immunohistochemical analyses to identify the amygdalar neurons that induce plastic changes in responses to saccharin during CTA acquisition within the bitter taste-relaying neurons defined by genetic tracing in mice. Immunohistochemical detection of induction of Zif268, an immediate early gene, revealed that more bitter taste-relaying neurons in the medial amygdala were activated by saccharin after acquiring CTA memory, compared with those observed before CTA, showing their plastic changes in activities during CTA acquisition.

### [2P04-03]
### CMOS-based bio-image sensor reveals spatiotemporal proton dynamics in the living brain

*Hiroshi Horiuchi[1], Masakazu Agetsuma[1], Junko Ishida[1], Dennis Cheung[1], Sawada Kazuaki[2], Junichi Nabekura[1] ([1]*National Insititute for Physiological Sciences*, [2]*Toyohashi University of Technology*)

The regulation of proton concentration (pH) in the brain is important for maintaining normal brain function. In the brains of healthy subjects, intracellular pH is maintained at 6.8-7.0, whereas extracellular pH is maintained at 7.2-7.4. While the homeostatic importance of pH regulation has long been appreciated, more recent studies have shown that protons can also directly participate in neurotransmission. This suggests an added dimension in terms of the relevance of pH changes to brain function under both physiological and pathological conditions. Double barreled and concentric microelectrodes can only measure pH at a single point, thus their utility is limited to correlating proton changes with globalized brain activity during seizures and ischemia. In contrast, magnetic resonance imaging (MRI) is able to simultaneously measure the distribution of protons in the entire brain and is thus able to detect regional variations in pH. However, to further investigate regional and neural activity-dependent proton dynamics in the brain, the development of a device with both wide-area detectability and high temporal-spatial resolution is necessary. Therefore, we developed a novel image sensor with a high spatial-temporal resolution specifically designed for measuring protons *in vivo*. Here, we demonstrate that spatially deferent neural stimulation by visual stimulation induced distinct patterns of proton changes in the visual cortex. This result indicates that our biosensor can detect micrometer and millisecond scale changes of protons across a wide area. To our knowledge, this is the first report showing that a CMOS-based proton image sensor with high spatial and temporal precision can be used to detect pH changes associated with biological events. Thus, we believe that our sensor may have broad applicability in future biological studies.

### [2P04-04]
### The increase of GAD65 expression by PTZ stimulations occurs specifically in SOM-positive interneurons in hippocampal CA1.

*Yuki Kajita[1], Yuki Fukuda[1], Riho Kawamatu[1], Takanori Oyanagi[1], Hajime Mushiake[1] ([1]*Department of Physiology Tohoku University School of Medicine*)

**Introduction:** The GAD67 and GAD65 are necessary for regulating normal neural activity and regarded to play distinct roles. In particular, GAD65 is related to the activity-dependent rapid GABA synthesis and its expression increases after repeated-electroconvulsive shocks, suggesting that GAD65 has crucial role to suppress the epileptic seizure. The GABAergic interneurons have many subpopulations that can be classified using several chemical makers. However, it is unclear which subtypes of interneuron contribute to the acquisition of epileptic resistance. To investigate this issue we examined the GAD65- expression level in each interneuron subtype using chemical stimulations.
**Methods:** To investigate the acquisition of the epileptic resistance, we performed chemical stimulation using GABA-A antagonist, pentylenetetrazole (PTZ). We injected PTZ in Long- Evans adult male rats repeatedly (40 mg/kg, i.p., 10 [short term] or 20 times [long term], one-shot/two days). The control (CTL) group were injected equal volume of saline. We calculated the GAD65-intensity (expression level) in the cell bodies merged with several GABAergic subtype markers across the hippocampal CA1.
**Results:** The GAD65-expression increased in short term group. This tendency was remarkable in somatostatin-positive (SOM+) interneurons compared with other GABAergic subpopulations. On the other hand, in the long term group, the GAD65-expression did not change compared with CTL group.

### [2P04-05]
### Effects of glial insulin signaling deletion on cognitive functions

Ryuhei Tsuji[1], Wei Wang[1], Daisuke Tanokashira[1], Megumi Maruyama[1], Chiemi Kuroiwa[1], *Akiko Taguchi[1] ([1]*Department of Integrative Neuroscience, National Center for Geriatrics and Gerontology*)

Dysfunction of insulin receptor substrate 1 (IRS1) is found in the brains with cognitive impairment and Alzheimer's disease (AD) in animals and human with/without type2 diabetes (T2D). However, it remains unknown how IRS1 is involved in regulating cognitive functions and glucose metabolism. We have found that alternation of astrocytic morphology occurs in the hippocampus of T2D model mice accompanied by IRS1 dysregulation and memory decline. These results suggest involvement of IRS1 in the modulation of memory functions and nutrient metabolism via astrocytes.To investigate this possibility, we have produced astrocyte-specific IRS1 deficient mice. Our results show that deletion of IRS1 in astrocytes leads to impairments of glucose metabolism and spatial memory in young mice despite normal body weight. Moreover, we have found that astrocytic IRS1 deletion causes decreases in protein levels of glucose transporters and mitochondria-related factors in the hippocampus.Our findings suggest that astrocytic IRS1 signaling may be associated with the regulations of systemic nutrient metabolism, cognitive functions, and mitochondrial maintenance.

### [2P04-06]
### Forelimb reaching exercise causes better motor function recovery with adaptive cerebellar oligodendrogenesis after intracerebral hemorrhage in rats

*Takeshi Shimizu[1], Aoi Sato[1], Mitsuki Kokuryoh[1], Shinya Ueno[1], Kenta Kobayashi[2], Hideki Hida[1] ([1]*Department of Neurophysiology and Brain Science, Graduate School of Medical Sciences, Nagoya City University,* [2]*Section of Viral Vector Development, National Institute for Physiological Sciences*)

An effective rehabilitation after stroke is the use of the impaired upper limb. Using intracerebral hemorrhage (ICH) model rats, we previously reported that a switch from the cortico-spinal pathway to the cortico-rubral pathway was observed in the motor execution system by the forced limb use (FLU), leading to functional recovery after ICH. However, it is still unclear whether dynamic change of the cerebellum in the motor regulatory system is also induced by rehabilitation after ICH. As growing number of studies demonstrates that neuronal activity-dependent oligodendrocyte (OL) remodeling is involved in motor learning, we assessed whether forelimb reaching exercise itself influences oligodendrogenesis after ICH and the reaching exercise causes adaptive changes in cerebellum-dependent motor regulatory system. We observed that the reaching exercise led to better recovery of skilled forelimb functions in ICH model rats. We also revealed the increase in the number of newly born OLs and CC1+ mature OLs in the cerebellar nucleus of the exercise group. These data suggest that OL remodeling under reconstruction of injured brain circuits by intervention therapy plays roles in functional recovery.

### [2P04-07]
### Functional oligodendrocytes regulate neural plasticity in activity dependent manner

*Kenji Yoshida[1], Shouta Sugio[1], Daisuke Kato[1], Ryouta Nakano[1], Hiroaki Wake[1] ([1]*Department of Anatomy and Molecular Cell Biology, Nagoya University Graduate School of Medicine*)

Myelin, which is formed by oligodendrocytes (OLs) regulate conduction velocity, thus modulating spike time arrival, that suggest contribution on synapse plasticity through spike timing dependent plasticity. Recent studies have shown that myelin is regulated by neural activity. However, it is unclear how OLs receive neural activity *in vivo*. Here we visualized $Ca^{2+}$ activity of OLs using *in vivo* two photon $Ca^{2+}$ imaging. In addition, we measured conduction velocity of thalamo-cortical projection with OLs manipulation through promotion of neural activity by chemogenic manipulation. We found that $Ca^{2+}$ activity of OLs were dependent on neural activity through AMPA receptor or P2 receptors. We further found the dispersion of conduction velocity was reduced with enhanced neural activity. The dispersion of conduction velocity was significantly increased with AMPA receptor inhibition by Elvax containing AMPA receptor blocker. Our results suggest OLs detect spike timing and regulate conduction velocity via AMPA mediated signals. We anticipate our analysis will be a focus of experimental research and help elucidate higher brain function beyond the synapse. Furthermore, OLs could be a main target to improve cognitive function and neuropsychiatric disease.

### [2P04-08]
### High-speed imaging of calcium elevations and glutamate extractions under Aβ oligomers exposure in primary cultured astrocytes

*Kaito Nakata[1,2], Kohei Otomo[1,2,3,4], Hirokazu Ishii[2,3], Motosuke Tsutsumi[2,3], Ryosuke Enoki[1,2,3], Tomomi Nemoto[1,2,3,5] ([1]*School of Life Science, The Graduate University for Advanced Studies,* [2]*National Institute for Physiological Sciences, National Institutes of Natural Sciences (NINS),* [3]*Exploratory Research Center on Life and Living Systems, NINS,* [4]*Graduate School of Medicine, Juntendo University,* [5]*Research Institute for Electronic Science, Hokkaido University*)

In the early stage of Alzheimer's disease (AD), dendritic spine loss is recognized as a pathological feature [Edwards *et al., Trends Neurosci*, 2019]. It has been hypothesized that dendritic spine loss was induced by astrocytic glutamate release excessively via amyloid-β (Aβ) oligomers-evoked intracellular $Ca^{2+}$ elevations [Liu *et al., Front. Neurosci*, 2019]. For the elucidation of the mechanism of how $Ca^{2+}$ elevations induce excess glutamate release in AD, we performed $Ca^{2+}$ or glutamate imaging in primary cultured astrocytes expressing fluorescent indicators, GCaMP6f-cytosol or iGluSnFR, respectively. Here, we used a home-built two-photon laser-scanning microscope utilizing a spinning disk scanner [Otomo *et al., Anal. Sci.*, 2015]. We found that the application of 5 μM $A\beta_{1\text{-}42}$ oligomers enhanced localized rapid $Ca^{2+}$ elevations. The frequency of the elevation increased from 0.5 to 1.8 times/min and the amplitude of the $Ca^{2+}$ indicator from 0.3 to 1.8 ($\Delta F/F_0$). This application also induced global and slow increases in the glutamate indicator from 0.83 to 41.1 (temporal integral $\Delta F/F_0$*s), while it did not induce a localized rapid increase. These results suggest that local and fast $Ca^{2+}$ elevations by $A\beta_{1\text{-}42}$ oligomers might be involved in the excessive glutamate release.

### [2P04-09]
### Astrocytic control of anxiety

*Wanqin Tan[1], Ko Matsui[1] ([1]*Super-network Brain Physiology, Graduate School of Life Sciences, Tohoku University*)

Emotions such as anxiety are generated to allow quick assessment of the environment. In psychiatric disorders, emotional assessment often becomes unbeneficial for adaptation. Habenula is a nucleus that plays an important role in the control of cognitive and emotional behaviors such as punishment avoidance and anxiety. Elevated steady-state neuronal activity in the habenula leads to aversive and anxious behaviors in mice. We aimed to understand the factors that control the neuronal activity in the habenula. One of the factors that we focused on was the astrocytes. High intensity GFAP staining was observed in the medial habenula suggesting of a presence of intimate neuron-glia interactions. With specific knock-out of a glutamate releasing anion channel in astrocytes, anxiety induced by elevated minus maze was alleviated. Photoactivation of ChR2 and ArchT can induce intracellular acidification or alkalization, respectively. Using fiber optics, ChR2 or ArchT expressed in astrocytes were photoactivated in the vicinity of the habenula as it has been suggested that intracellular pH control glial glutamate release via anion channels. Recently, it has also been reported that some of the psychiatric disorders are associated with gross brain acidification. Whether astrocyte pH in the habenula can dynamically change depending on emotion are currently being investigated with fiber photometry.

### [2P04-10]
### Plasticity of brain environment upon development of epilepsy

*Yoko Ikoma[1], Daichi Sasaki[1], Ko Matsui[1] ([1]*Super network Brain Physiology, Graduate School of Life Sciences, Tohoku University*)

Long-term transition of the mode of action potential firing may underlie physiological process of learning and memory as well as pathogenesis such as epilepsy. Such plastic changes may be governed not only by the well-studied long-term potentiation/depression mechanisms of synaptic transmission but rather by a simple shift in the ambient ion and/or transmitter concentrations. Governing of the local brain environment is the primal function of astrocytes. In this study, plastic change of astrocyte reactions was studied using epileptogenesis as an extreme form of plasticity. Fluorescent sensors for calcium or pH expressed in astrocytes were examined for up to one week by in vivo fiber photometry in freely moving transgenic mice. Using a newly devised method for the analysis, changes in $Ca^{2+}$ and pH in astrocytes and changes in the local brain blood volume (BBV) associated with hippocampal-stimulated epileptic seizures were extracted. Following a transient alkaline shift in the astrocyte triggered by neuronal hyperactivity, a prominent acidic shift appeared in response to intensified seizure which developed with kindling. This acidic shift may trigger additional gliotransmitter release from astrocytes leading to the exacerbation of epilepsy. Controlling the astrocyte pH could be a new therapeutic target for treatment of epilepsy.

### [2P04-11]
### Microglial involvement in the dysregulation of aquaporin-4 after acute adrenergic receptor agonism.

*Emiko Morita[1], Hiromu Monai[1] ([1]*Ochanomizu University*)

Brain fluid dynamics maintain the brain milieu by metabolic wastes clearance. Aquaporin-4 (AQP4) is thought to be a driving force behind the fluid dynamics in the brain. AQP4 is locally expressed on the endfeet of astrocytes. Therefore, the localization of AQP4 plays a crucial role in the flux, which may be dysregulated with age and certain diseases. We have demonstrated that AQP4 polarization is rapidly changed after acute ischemic stroke, but adrenergic receptor (AdR) antagonism preserves the regulation in mice. However, the mechanism by which AdR activation is linked to AQP4 polarization is still unclear.In this paper, we induced AQP4 dysregulation by the topical application of AdR agonist to the anesthetized living mouse cortex. We proposed the analysis method to evaluate the AQP4 polarization using double immunohistochemistry staining with AQP4/Lectin and AQP4/ glutamate synthetase. As a result, we found that AQP4 polarization was significantly decreased by applying beta AdR agonist, isoproterenol, for 3 hours. Furthermore, the application of isoproterenol also induced a significant increase of microglia immunofluorescence, Iba-1, whereas no apparent change in astrocytes reactivity, GFAP. In the poster presentation, we will further discuss minocycline's effect, which inhibits microglial activations on the AQP4 polarization.

### Poster Presentation 2

### [2P05]
### Sensory function, Sensory organ

March 17(Thu), 12:00 - 14:00, Zoom P5

### [2P05-01]
### Activation of TRP channels by an endocannabinoid and other stimuli in *Drosophila* photoreceptor cells

*Takaaki Sokabe[1], Heather Bradshaw[2], Craig Montell[3] (*[1]National Institute for Physiological Sciences, [2]Indiana University, [3]University of California*)

The phototransduction in *Drosophila* represents a classical model for understanding intracellular signaling involving GPCR and TRP channels. Despite numerous efforts over the last three decades, however, the regulation of TRP channel gating downstream of phospholipase C (PLC) activation has not been conclusive. We quantified the amount of lipid metabolites in photoreceptor cells upon illumination and found that an endocannabinoid, 2-linoleoyl glycerol (2-LG), was upregulated in PLC-dependent manner. 2-LG, but not oleic acid or diacylglycerol, enhanced the activity of TRPL channels in the heterologous expression system. 2-LG also activated TRP and TRPL channels in dissociated photoreceptor cells in a light-independent manner. We will also discuss the synergistic effects between 2-LG and other known positive regulators including proton and mechanical stimulation. This study revealed the new role of the endocannabinoid and emphasizes its potential importance in *Drosophila* phototransduction.

### [2P05-02]

### A mathematical model of photoreceptor inner and outer segments estimating the ion transport activities with the Goldman-Hodgkin-Katz equation

*Yuttamol Muangkram[1], Junpei Takita[1], Yukiko Himeno[1], Akira Amano[1] (*[1]Dept. of Bioinformatics, Coll. of Life Sciences, Ritsumeikan Univ.*)

The classical Goldman-Hodgkin-Katz (GHK) theory is widely used for explaining the cell membrane electrophysiological phenomena. In this study, we aimed to construct a new mathematical model based on the GHK current equation to quantitatively describe the photoreceptor electrical responses to a flash of light and voltage-clamp pulses. The currently developed model based on the vertebrate photoreceptor model of Kamiyama et al. (2009) incorporates the GHK constant field equations. The model includes various ion pumps, exchangers, and transporters that are identified in photoreceptor cells: $I_{photo}$, $I_{NCKX}$, $I_h$, $I_{Kv}$, $I_{Ca}$, $I_{Cl(Ca)}$, $I_{K(Ca)}$, $I_{Na}$, $I_{NaCa}$, $I_{PMCA}$, $I_{NKCC1}$, $I_{KCC2}$, and leakage currents. The simulation can quantitatively demonstrate the essential mechanisms underlying the actual biophysical processes of the ion transport activities in photoreceptor inner and outer segments. The electrical characteristics of individual ionic current and the equilibrium potential of ion species and ion channels are in good agreement with experimental and theoretical works. Furthermore, this study can predict the alterations in $K^+$, $Na^+$, and $Cl^-$ over a wide variety of light intensities and voltage-clamp conditions. In addition, this new model can provide an effective tool in investigating the retinal pathologic changes and serve as a practical guideline in developing the new drug research.

### [2P05-03]
### Ion-channel kinetics specifying manner of information transfer in mouse olfactory sensory neurons.

*Tomohiro Noguchi[1], Hitoshi Sasajima[2], Sadaharu Miyazono[2], Kaoru Takakusaki[1] (*[1]Dept. of Physiology, Div. of Neuroscience, Asahikawa Med. Univ., [2]Cent. for Advanced Research and Education, Asahikawa Med. Univ.*)

Previously, our patch-clamp study revealed phasic firing of olfactory sensory neurons (OSNs) and tonic firing of vomeronasal sensory neurons (VSNs) elicited by current injection. However, physiological significance of different firing pattern between OSNs and VSNs remains unclear. Recently, our information analysis on spike encoding showed a complementary manner of the information transfer of OSNs and VSNs. Phasic firing of OSNs can convey information of sinusoidal current stimulus with low amplitude in short cycle duration but tonic firing of VSNs can encode that in long cycle duration. It suggests that the intrinsic firing pattern of OSNs and VSNs regulates information transfer of them. Here, we show intrinsic firing patterns give rise to stimulus dynamics-dependent manners of information transfer. To demonstrate relationship between firing pattern and information transfer, we constructed Hodgkin-Huxley models (HH models) with channel kinetics obtained from voltage-gated $Na^+$ and $K^+$ currents ($I_{Na}$ and $I_K$) of OSNs and VSNs. Our HH models composed of intrinsic channel kinetics exhibited characteristic firing pattern of OSNs and VSNs. In addition, the HH models showed information transfer depending partially on cycle duration of stimulus dynamics. These results suggest that stimulusdynamics dependence of information transfer results from an ensemble of ion channels not only voltage-gated $Na^+$ and $K^+$ channels but also other channels. COI: No.

### [2P05-04]
### P2X3 receptors modulate the visual information processing in the retina.

*Toshiyuki Ishii[1], Atsushi Shimohata[1], Chiaki Suzuki[1], Tomomi Shimogori[2], Makoto Kaneda[1] (*[1]Nippon Medical School, [2]RIKEN*)

Adenosine triphosphate (ATP) acts as a neurotransmitter in the nervous system. We have previously reported that all subtypes of P2X receptors (P2X1 - P2X7) are expressed in the mouse retina, and a non-selective P2X receptor antagonist modulates the firing of retinal ganglion cells. However, the types of P2X receptors contributing the signal processing in the retina have not been well elucidated. In this study, we focused on P2X3 receptors. First, we investigated the localization of the P2X3 receptor by *in situ* hybridization and immunohistochemistry. In the adult mouse retina, P2rx3 mRNA was expressed in the ganglion cell layer (GCL), and the immunoreactivity was found in the inner plexiform layer and GCL. We next examined the physiological function of P2X3 receptors in the retina using an antagonist of P2X3 receptor, A317491. In the electroretinogram, intravitreal injection of A317491 had no effects on the amplitudes of both a- and b-waves, whereas significantly decreased the amplitude of oscillatory potentials. In multielectrode array, application of A317491 modulated the firing rate of ON and OFF-retinal ganglion cells. These results suggest that the P2X3 receptors physiologically work for the visual information processing in the mouse retina.

### [2P05-05]
### Role of N-linked glycosylation on the extracellular domain in mGluR6 cell surface localization

*Takumi Akagi[1], Atsushi Shimohata[1], Ikuo Ogiwara[1], Makoto Kaneda[1] (*[1]Nippon Medical School*)

Metabotropic glutamate receptor 6 (mGluR6) is predominantly expressed in the dendritic tips of retinal bipolar cells, and plays critical roles in the processing of visual signals. We have shown the involvement of the intracellular C-terminal domain in mGluR6 cell surface localization and intracellular signaling (Rai et al. J. Neurochem. 2021), while the importance of the extracellular domain (ECD) on mGluR6 intracellular trafficking has been well recognized. We here examined whether N-linked glycosylation of mGluR6 ECD is required for surface localization. We first showed that an N-glycosylation inhibitor, Tunicamycin, drastically reduced surface levels of mGluR6 expressed in HEK 293T cells. We next showed that mutated mGluR6 with Asn to Gln substitutions at putative N-glycosylation sites (Asn-Xaa-Ser/Thr) within the ECD impaired surface localization. The molecular weight of the mGluR6 mutant is reduced to the level of wild-type mGluR6 expressed in Tunicamycin-treated cells. These findings suggest that mGluR6 is Nglycosylated at Asn residues within the ECD, and that N-glycosylation is indispensable for mGluR6 intracellular trafficking and cell surface localization.

### [2P05-06]

### Relationships between pure-tone response duration and variety of responses to multiple sound stimuli in the primary auditory cortex

*Sohei Chimoto[1] ([1]*Department of Neurophysiology, Division of Medicine, University of Yamanashi*)

The primary auditory cortex (A1) neurons showed a wide variety of the response timecourses from phasic to sustained patterns to pure tone stimuli. Although A1 neurons show different response patterns during various sounds such as click trains, AM sounds, FM sounds, and natural sounds, it remains unknown whether the same neuron responds to pure tone and other kinds of sound in the specific response pattern, or there are many kinds of specific neurons responding to only specific sounds. In this study, response time courses to various artificial sounds (pure tones, click trains, AM sounds, and FM tones) and natural sounds (species-specific sounds, human vowels, environmental sounds) were examined by using long time recording technique of single neurons. Phasic type neurons respond to pure tones and natural sounds with short response duration pattern, to click trains with synchronized pattern, and to AM and FM with edge pattern. Sustained type neurons respond to pure tones and natural sounds with long response duration pattern, to click trains with non-synchronized pattern, and to AM and FM with slope and edge-slope pattern. The presence of only two types of neurons explains the variety of response time courses of A1 neurons for various artificial and natural sounds. That is, the same neuron responds to pure tone and other kinds of sound in the specific response pattern.

### [2P05-07]

### Effects of NMDA receptor antagonist on the change of motor cortex tetanic stimuli-induced neural activity in rostral ventromedial medulla in chronic pain model rats

*Hiromasa Kitazawa[1] ([1]*Dept of Histology and Neuroanatomy, Tokyo Medical University* )

Motor cortex stimuli lead to anti-nociception in both human and experimental animals, however precise mechanism remains to be unknown. In the previous preliminary study using single unit recording methods, we found that the ON cells in the rostroventral medulla (RVM), which are one kind of neurons increasing their activity in response to noxious stimuli, reduced ongoing (spontaneous) activity by application of motor cortex tetanic stimuli in chronic pain model rats (spared nerve injury rats (SNI rats)), suggesting that RVM neurons are involved in motor cortex stimuli-induced anti-nociception in SNI rats. In the present study, we investigated the involvement of the NMDA receptors expressed in the RVM in this anti-nociception mechanism. NMDA receptor antagonist APV was directly injected into the RVM with a micro-syringe prior to motor cortex tetanic stimuli. The injection of APV did not change ongoing activity of the ON cells after motor cortex tetanic stimuli in SNI rats, as APV cancelled the effects of motor cortex tetanic stimuli. RVM NMDA receptors are one of the causes of chronic pain, however, may be involved in motor cortex stimuli-induced pain-relief.

### [2P05-08]

### A near-infrared spectroscopy (NIRS) study on effects of visual information on haptics

*Akitoshi Seiyama[1], Nami Konishi[2], Sayaka Okahashi[1,3], Monte Cassim[4,1] ([1]*Graduate School of Medicine, Kyoto University,* [2]*Department of Nursing, Kyoto Tachibana University,* [3]*National Center for Geriatrics and Gerontology,* [4]*Akita International University*)

[Introduction] It is well known that vision and haptics mutually are affecting as reported as the rubber hand illusion and the mirror box illusion. In the former visual information affects tactile information while the latter is the opposite. The mechanisms, however, are not fully understand. Using a NIRS, therefore, we examined brain activity during tactile tracing on the sine-shape acrylic board to investigate effects of 1) visual information and 2) of spatial frequency of the sine-shape on the brain activity. [Methods] Six of healthy volunteers were participated in the study. We used about 3 and 30 Hz of spatial frequencies as low- and high-tactile-stimuli, respectively. Subjects performed the tracing tasks at 1 Hz of temporal frequency for 20 cm length of the acrylic board and only space moving without touching as a control task. Two types of experiments with and without watching the acrylic board were tested. [Results and Discussion] We found the frequency dependent visual information effect on the neural activation in the sensory area. Further, it is suggested that the effects include not only intensity of neural activity but also activation pattern.

### [2P05-09]

### Evaluation of TRPA1 activators as insect repellents

*Shoma Sato[1,2], Takaaki Sokabe[1,2] ([1]*Thermal Biology Group, Exploratory Research Center on Life and Living Systems, National Institutes of Natural Sciences,* [2]*Division of Cell Signaling, National Institute for Physiological Sciences, National Institutes of Natural Sciences*)

Transient receptor potential (TRP) A1 channels expressed in sensory neurons are activated by various harmful stimuli and play key roles in nociception and aversive responses in many species. Since insect TRPA channels are also known to participate in aversive behaviors, we reasoned that mammalian TRPA1 activators can be utilized as an insect repellent. In this study, we focused on 2-methylthiazoline (2MT) which has been originally reported as an odorant that elicits an innate fear in mice via the activation of TRPA1. First, we examined whether 2MT can induce an aversive response in *Drosophila melanogaster*. We performed a two-choice assay in which starved-flies choose sucrose containing agarose in the presence or the absence of 2MT. We observed strong, dosedependent avoidances against 2MT in the wild-type flies, whereas such avoidance disappeared in the TRPA1 mutant flies. In addition, the application of 2MT induced $Ca^{2+}$ responses in *Drosophila* TRPA1-expressing HEK293 cells. These results indicate that 2MT directly activates *Drosophila* TRPA1 as well as mouse TRPA1. Taken together, we propose a novel strategy for insect pest management aiming at insect TRP channels as a target.

### [2P05-10]

### Acetylcholine release from ON and OFF starburst amacrine cells are regulated by different feedback mechanisms.

*Mie Gangi[1], Takuma Maruyama[1], Toshiyuki Ishii[1], Kaneda Makoto[1] ([1]*Department of Physiology, Nippon medical school*)

Starburst amacrine cells (SACs), which release acetylcholine (ACh) and GABA as transmitters, play a key role in motion detection in the retina. SACs typically comprise two spatially segregated populations that form circuits in the ON or OFF synaptic layers of the inner retina. Because of the easy accessibility to the soma, the signal processing pathway of ON SACs has been well studied. Currently, the signal processing pathway of OFF SACs is assumed to be same as that of ON SACs.
However, in a series of experiments, we showed differences between ON and OFF SACs. The P2X2-purinergic receptors selectively drive OFF SACs, while glycine receptors mainly work in ON SACs. In addition, choline, a precursor of ACh, is transported through different pathways between ON and OFF SACs.
In the present study, therefore, we examined whether such differences between ON and OFF SACs can produce further difference in cholinergic signaling pathways. We found that the GABAergic IPSCs in SACs are regulated with different ACh receptors between ON and OFF SACs. In adult mice, a muscarinic agonist induced IPSCs in ON SACs but not in OFF SACs, while a nicotinic agonist induced IPSCs in both SACs. These results indicate that ON and OFF cholinergic pathways are not mirror-symmetric and might differentially contribute to motion detection in the retina.

### [2P05-11]

### Intercellular communication between trigeminal ganglion neurons and odontoblasts

*Natsuki Saito[1,2], Maki Kimura[2], Takehito Ouchi[2], Rumi Kaneko[1,2], Sejin Kwon[1,2], Tatsuya Ichinohe[1], Yoshiyuki Shibukawa[2] ([1]*Tokyo Dental College Department of Dental Anesthesiology,* [2]*Tokyo Dental College Department of Physiology*)

Inflammatory response following the dental pulp injury and/or infection has implied to induce neuropeptide release from nerve endings to the pulpal tissue and cells, including endothelial cells and/or odontoblasts. This results in the neurogenic inflammation. Since detailed mechanism in occurrence of neurogenic inflammation in dental pulp has not yet been clarified, we examined intracellular cAMP signaling pathway in odontoblasts by activation of $G_s$ protein-coupled receptors, as well as intercellular trigeminal ganglion (TG) neuron-odontoblast communication following direct mechanical stimulation (mimicking tissue pressure increase by dental pulp inflammation) to the TG neurons. Application of an adenylyl cyclase activator showed concentration-dependent increases in the intracellular cAMP level ($[cAMP]_i$) in odontoblasts, showing desensitizing effects. Applications of CGRP, and PTH receptor agonists also increased $[cAMP]_i$. The increases were significantly inhibited by application of each selective receptor antagonist, and an adenylyl cyclase inhibitor. Mechanical stimulation to TG neurons increased $[cAMP]_i$ in nearby odontoblasts to the stimulated neuron, showing distance dependence. These increases in nearby odontoblasts were significantly inhibited by CGRP receptor antagonist. These results suggested that CGRP/PTH receptor activation increased $[cAMP]_i$ by activation of adenylyl cyclase in odontoblasts. Furthermore, CGRP might mediate intercellular communication between TG neurons and odontoblasts.

### Poster Presentation 2

### [2P06]
### Circulation

March 17(Thu), 12:00 - 14:00, Zoom P6

### [2P06-01]
### Eicosapentaenoic acid rescues L-type $Ca^{2+}$ channel remodeling in cardiomyocytes caused by saturated fatty acids

*Masaki Morishima[1], Hanako Murakami[1], Pu Wang[2], Kazuki Horikawa[3], Katsushige Ono[2] ([1] *Kindai University*, [2] *Oita University*, [3] *Tokushima University*)

Excessive uptake of saturated fatty acids is associated with impairment of cardiovascular functions, while polyunsaturated fatty acid including eicosapentaenoic acid (EPA) has been shown to exert protective effects. This study was purposed to investigate the possible beneficial actions of EPA on cardiomyocyte focusing on the L-type $Ca^{2+}$ channel. Neonatal mice cardiomyocytes were cultured with an oleic acid (OA, 250 mM)-palmitate (PA, 500 mM) mixture (OAPA) in the absence or presence of EPA (10 mM) for 24 h. L-type $Ca^{2+}$ channel current, mRNA and the protein expressions of the Cav1.2-L-type $Ca^{2+}$ channel were significantly reduced by OAPA, which was rescued by EPA. OAPA downregulated the phosphorylated component of a transcription factor adenosine-3', 5'-cyclic monophosphate (cAMP) response element binding protein (CREB) in the nucleus, which was also rescued by EPA. Immunocytochemical analysis revealed a distinct downregulation of Cav1.2 and CREB proteins caused by OAPA, which was markedly conserved in the presence of EPA. These results suggest that EPA rescues cellular damages caused by OAPA lipotoxicity through the CREB-mediated pathways.

### [2P06-02]
### The physiological response of free-moving mice during optogenetic-based cardiac pacing

*Jun Kaminosono[1], Yuki Kambe[1], Akihide Tanimoto[1], Tomoyuki Kuwaki[1], Akira Yamashita[1] ([1]*Kagoshima University*)

A cardiac pacemaker is applied for certain kinds of cardiovascular diseases. However, the pacing leads and electric stimuli occasionally cause some complications. Recently, optogenetic-based cardiac pacing enables us to stimulate the cardiac muscle in a noncontact manner. In previous studies, pacing was applied ex vivo or in anesthetized animals. Therefore, the physiologic response of animals during optogenetic pacing remains unknown. Here, we established a method of optogenetic-based cardiac pacing in freelymoving mice for the first time and simultaneously measured electrocardiogram, blood pressure, and respiration. As a result, light-induced myocardial contraction produces blood flow and indirectly affects the respiration rhythm. Additionally, light illumination enabled heart rate recovery in bradycardic mice. These findings may be employed for further medical translational research for patients with a pacemaker. Together, this method may aid in the development of less invasive pacemakers without pacing leads.

### [2P06-03]
### Analysis of telomere length and expression of telomere-related proteins in the human lung with pulmonary hypertension

*Xiaodong Li[1], Lin-Hai Kurahara[1], Keizo Hiraishi[1], Kaori Ishikawa[2], Ryou Ishikawa[3], Yoko Matsuda[4], Katsuya Hirano[1] ([1]*Department of Cardiovascular Physiology, Faculty of Medicine, Kagawa University*, [2]*Department of General Internal Medicine, Kagawa University Hospital*, [3]*Department of Diagnostic Pathology, Kagawa University Hospital*, [4]*Oncology Pathology, Department of Pathology and Host-Defence, Faculty of Medicine, Kagawa University*)

**Background and purpose:** Pulmonary hypertension (PH) is intractable vascular disease with pulmonary artery (PA) remodeling. The telomerase activity is related to cell proliferation, while the human telomerase reverse transcriptase (hTERT) is activated by telomere shortening or phosphorylation by cyclin dependent kinase 1 (CDK1). The telomere length is shortened following cell senescence and mitogenesis. This study analyzed the telomere length and expression of the telomere-related proteins in the human lung with PH. **Methods:** Expression of the active hTERT and the telomere-related proteins was detected with immunofluorescence staining, and the telomeric length were determined with Fluorescence In Situ Hybridization in the lung tissues of PH and non-PH patients. **Results:** Compared to the non-PH lung, the PH lung exhibited greater number of cells with shorter telomere in and around the PA, despite the increased expression of hTERT, which was co-expressed with CDK1. Ninety percent of the hTERT-positive cells around PA were CD44-positive. The cells positive for p16, a senescence marker, were observed mainly in the PA endothelium in the PH lung, and they were additionally observed in the smooth muscle layer in the lung of PH with systemic scleroderma. **Conclusion:** hTERT is suggested to be reactivated in response to telomere shortening due to cellular senescence in the human lung with PH.

### [2P06-04]
### Exercise reverses the sarcomeric and cytoskeletal contributions to diastolic dysfunction in mice with diet-induced insulin resistance and obesity

*James T Pearson[1], Takashi Sonobe[1], Hirotsugu Tsuchimochi[1], Mark Waddingham[1], Nozomi Tokuhara[1], Yuko Iwata[1] ([1]*NCVC*)

We investigated how insulin resistance and obesity due to a high fat high sugar (HFHS) diet affects left ventricle (LV) relaxation in vivo in a non-genetically predisposed mouse model (male B6D2 F1 hybrid) from cardiomyocyte level (X-ray diffraction) to global LV indices (echocardiography). Further, we examined how intervention with exercise training in combination with normal diet affected contractile function-relaxation and cytoskeletalsarcomeric protein modifications. Systolic LV function was not impaired by HFHS diet. However, LV reverse strain rate indices were depressed in HFHS sedentary mice (impaired relaxation) and normalised in exercised mice. This impairment was paralleled by cardiac hypertrophy, increased NF-$\kappa\beta$ and acetylated lysine expression, upregulation of relative detyrosinated and to a lesser extent acetyl $\alpha$-tubulin expression and troponin T phosphoprotein. Even in the absence of chronic inflammation, LV hypertrophy and microtubule-myofilament modifications contribute to reversible diastolic dysfunction in diet-induced insulin resistance.

### [2P06-05]
### Nr4a1 suppressed prostaglandin $E_1$-promoted intimal thickening in rat ductus arteriosus.

*Toru Akaike[1], Takako Yokota[1], Susumu Minamisawa[1] ([1] *The Jikei University*)

**AIM:** Ductus arteriosus (DA), an essential fetal artery, closes right after birth. Intimal thickening caused by prostaglandin E (PGE)-promoted hyaluronan production, cell migration, and cell proliferation play an important role in DA closure. Nr4a1 is known to regulates cell migration and proliferation in pulmonary artery. However, it has not been elucidated the role of Nr4a1 in DA. We then explored the role of Nr4a1 in rat DA closure.

**METHODS AND RESULTS:** We used DA tissues and smooth muscle cells of Wistar rats on embryonic day 21. RT-PCR analysis revealed that Nr4a1 siRNA decreased $PGE_1$-induced *Has2* upregulation in DA smooth muscle cells. Moreover, Nr4a1 siRNA and Nr4a1 inhibitors (DIM-C-pPhOH, DIM-C-pPhCO$_2$ Me) inhibited $PGE_1$ -inducued hyaluronan production in DA SMCs. Next, Fibulin 1 is known to have a promotive effect of cell migration in DA. RT-PCR analysis revealed that Nr4a1 siRNA decreased $PGE_1$-induced *Fbln1* upregulation in DA smooth muscle cells. Finally, Nr4a1 inhibitors suppressed prostaglandin $E_1$-promoted intimal thickening of DA tissues in organ culture.
**CONCLUSION:** Nr4a1 suppressed $PGE_1$-promoted intimal thickening via inhibition of hyaluronan production and cell migration in rat DA.

### [2P06-06]

### Onset mechanisms of spiral wave reentry occurring in a long QT syndrome type 2 model: insights from a simulation study

*Kunichika Tsumoto[1], Takao Shimamoto[2], Yuma Aoji[2], Yu-ichi Kuda[1], Mamoru Tanida[1], Akira Amano[2], Yasutaka Kurata[1] ([1]*Kanazawa Medical University,* [2]*Ritsumeikan Univ.*)

Reentry is a basic mechanism of cardiac arrhythmias, and torsades de pointes (TdP) results from a spiral wave reentry that meanders through the ventricles. It has been suggested that TdPs observed in patients with long QT syndrome type II are triggered by the development of early afterdepolarization (EAD) mediated by an excessive action potential prolongation in ventricular myocytes. However, the role of EAD in the generation of TdP remains unclear. In the present study, we investigated the relationship between EAD and TdP initiation by constructing a 6 × 6 cm sheet model consisting of a human ventricular myocyte model (Kurata et al., Biophys J, 2005) and performing simulations of excitation propagation. The EADs were assumed to occur in islands (clusters) in the ventricular tissue, and the relationship between the number of EAD clusters and the initiation of TdP was investigated. In the case of a single island (4 × 4 cm EAD cluster), TdP was not elicited. Spiral wave reentry was caused by dividing the 4 × 4 cm EAD cluster into 4, 9, and 16 islands. However, no TdP initiation occurred in the 25 clusters. This suggests that not only the spatial distribution of EAD clusters but also the cluster size influences the TdP initiation.

### [2P06-07]

### Modulation of Vascular Inflammation by Prostaglandin $E_3$

*Ryo Ishiwata[1], Yuji Morimoto[1] ([1]*Department of Physiology, National Defense Medical College*)

[Background] Vascular Inflammation is a key feature of vascular diseases. We have previously reported that Prostaglandin $E_2$ ($PGE_2$) exacerbates vascular inflammation by acting on vascular smooth muscle cells (VSMCs). Prostaglandin $E_3$ ($PGE_3$) is a $PGE_2$ analog which is derived from eicosapentaenoic acid (EPA). While the effects of EPA on atherosclerosis are widely documented, the effects of $PGE_3$ on vascular inflammation are elusive. [Methods and Results] We stimulated human aortic smooth muscle cells *in vitro* with $PGE_2$ or $PGE_3$ at 1 mmol/L for 24h. The results of qRTPCR revealed that both $PGE_2$ and $PGE_3$ upregulated mRNA level of *IL6* (3.87±0.88- fold, p=0.01 and 3.44±0.64-fold, p=0.04, respectively). To examine the effects of $PGE_3$ on atherosclerosis, 8 weeks-old male LDLR-/- mice were fed with high fat diet for 12 weeks and were intraperitoneally administered with $PGE_3$ at 20 μg/kg of body weight every 48h. Oil red O staining of aorta showed that the plaque area in aorta was higher in $PGE_3$-treated group than in vehicle-treated group (7.5±1.8% vs. 9.8±1.0%, *n*=6). [Conclusions] $PGE_3$ possibly acts as a proinflammatory mediator in vascular inflammation and promotes atherosclerosis progression.

### [2P06-08]

### Mechanism which determines Ion Concentration Equilibrium of Ventricular Myocyte Mathematical Model under various Cycle Length, Stimulation Current, INa block, and Action Potential Duration

*Ryosuke Hara[1], Koki Koyama[1], Yukiko Himeno[1], Akira Amano[1] ([1]*Ritsumeikan University faculty of Life Sciences*)

Reduction of cycle length (CL), or sodium channel conductance (INa block) causes $Ca^{2+}$ concentration increase in ventricular myocytes, which results in increase of contraction force. For the mathematical model, increase in stimulation current (Istim) is known to decrease $Ca^{2+}$ concentration and decrease contraction force. Understanding of the underlying mechanism of ion concentration equilibrium is thus important for understanding pathological phenomena. In this research, we constructed quantitative explanation of the mechanism which determines the ion concentration equilibrium under various CL, INa block, Istim conditions, by using human ventricular myocyte model O' hara Rudy model. Total $Na^+$ influx during single cardiac cycle can be formulated by constructing fitting function of each $Na^+$ carrying membrane current by using CL, Istim, INa block, APD and $Na^+$ concentration as independent variables, and $Na^+$ efflux can also be formulated similarly. Equilibrium $Na^+$ concentration can be calculated by which equal $Na^+$ influx and efflux are obtained. Since the equations are simple, we can understand how the equilibrium concentration changes with various CL, Istim, INa block and APD.

### [2P06-09]

### Comparative study on transcriptome in heart isolated from mouse, rat, and human.

*Yosuke Okamoto[1], Daigo Okada[2], Kirill Tarasov[3], Yelena Tarasova[3], Bruce Ziman[3], Edward Lakatta[3], Toshiro Io[4], Koji Shinozaki[4], Daigo Kobayashi[1], Kyoichi Ono[1] ([1]*Akita University Graduate School of Medicine,* [2]*Center for Genomic Medicine, Graduate School of Medicine, Kyoto University,* [3]*Institute on Aging, National Institute of Health,* [4]*Ono Pharmaceutical Co., Ltd.*)

The heart is the first organ to function during development, and its function does not stop until death. It is the main organ for the maintenance of individual life and has been the subject of research by many researchers. However, despite the existence of species differences in the response to bioactive substances and the functions of the molecules expressed, there is no unified view on the critical differences between humans and other species, or on the universal expression of molecules across species. In this study, we performed mouse microarrays, rat microarrays, and human RNA-seq on various regions of the heart, namely the left ventricle, right ventricle, left atrium, sinoatrial node, and pulmonary veins, to identify common and different expression patterns in each animal species. Genes with human orthologs were selected from the mouse and rat array data, and universally overexpressed genes were identified from the human orthologs. Species-specific genes were identified including genes other than human orthologs. To confirm the microarray data, we measured the expression of sinus node specific transcription factors in mice and rats by quantitative PCR. We identified genes that are differentially expressed in humans and rodents, and transcription factors that are specifically expressed in each cardiac region throughout the species.

### [2P06-10]

### Role of NOX4-TRPV1 interaction on single cell mechanics in mouse ventricular cardiomyocytes.

*Keiko Kaihara[1], Keiji Naruse[1], Gentaro Iribe[2] ([1]*Dept Cardio Physiol, Grad Sch Med, Okayama Univ.,* [2]*Dept Physiol, Asahikawa Medical Univ.*)

We have previously reported that myocardial stretch increases reactive oxygen species (ROS) derived from NADPH oxidase 4 (NOX4). Although, evidence has shown the interactions between cellular ROS production and activation of transient receptor potential vanilloid type 1 (TRPV1) ion channels, their physiological role in cardiomyocytes is not clear.
To investigate their role during myocardial stretch, ventricular cells were enzymatically isolated from either 10-week-old wild type (WT) or NOX4 knock out (KO) or TRPV1 KO mice hearts. Isolated cells were exposed to 8~10% axial stretch using computercontrolled piezo-manipulated carbon fibers attached to both cell ends. Cellular ROS production was estimated using 2'-7' dichlorofluorescein (DCF). Cellular contractility was evaluated by the slopes of end-systolic force-length relation curves.
Both genetic NOX4 deletion (NOX4 KO) and TRPV1 deletion (TRPV1 KO) reduced cellular contractility, while stretch-induced increase in ROS production was abolished only in NOX4 KO group. The present results suggest that TRPV1 is activated by NOX4-derived ROS during stretch to support cellular contractility.

### [2P06-11]

### A simulation analysis of $Na^+$ and $Ca^{2+}$ dynamics in cardiomyocyte during ischemia and reperfusion

*Satoshi Matsuoka[1,2], Takao Shimayoshi[3], Ayako Takeuchi[1,2] ([1]*Department of Integrative and Systems Physiology, Faculty of Medical Sciences, University of Fukui,* [2]*Life Science Innovation Center, University of Fukui,* [3]*Research Institute for Information Technology, Kyushu University*)

Ischemia and subsequent reperfusion induce severe damages in cardiac excitation-contraction (E-C) coupling. The underlying mechanisms have been extensively studied and the key factors have been elucidated, e.g. acidosis, ATP depletion, and $Na^+$ and $Ca^{2+}$ overload. To get a comprehensive view of the mechanisms underlying cardiac ischemia and reperfusion, we developed a mathematical model of ventricular myocyte, through extending our previous model (Kuzumoto et al., 2008). The model includes membrane excitation, contraction, intracellular ion homeostasis, and mitochondrial oxidative phosphorylation. Ischemia was induced by a gradual decline of extracellular $O_2$ over several tens minutes. The model well reproduced experimentally observed changes in E-C coupling during the ischemia and reperfusion. Cytosolic and mitochondrial pH declined during ischemia, though oxidative phosphorylation was largely maintained till $O_2$ declined below a critical level. Subsequent reperfusion induced large accumulation of cytosolic $Na^+$ and $Ca^{2+}$, resulting in mitochondrial $Ca^{2+}$ overload and dysfunction. Activation of $Na^+$ pump during the reperfusion attenuated the $Na^+$ and $Ca^{2+}$ overload as well as mitochondrial energetics. It was suggested that cytosolic Na+ and $Ca^{2+}$ overload has significant effects on E-C coupling and mitochondrial energetics during cardiac ischemia and reperfusion.

### Poster Presentation 2

### [2P07]
### Respiration, Digestion, Digestive system

March 17(Thu), 12:00 - 14:00, Zoom P7

### [2P07-01]
### Endogenous hydrogen sulfide in the respiratory center is required to maintain respiratory frequency and power

*Minako Okazaki[1,2], Tadachika Koganezawa[1,3] (*[1]Dept Neurophysiol, Fac Med, Univ Tsukuba, [2]Grad Sch Comp Human Sci, Univ Tsukuba, [3]Transborder Med Res Ctr, Univ Tsukuba*)

Hydrogen sulfide is generated in the brain and works as a neuromodulator regulating synaptic transmission. Although the neural network generates a respiratory pattern at the respiratory center that is mainly composed of the preBö tzinger and the Bö tzinger complexes, the role of hydrogen sulfide in these regions is still unclear. Therefore, we aimed to evaluate the effects of the hydrogen sulfide in the respiratory center on the central respiratory pattern generation. We performed *in situ* arterially perfused preparations of decerebrated rats. An inhibitor of hydrogen sulfide-producing enzyme, cystathionine b-synthase (CBS), was locally microinjected into the respiratory center. The central respiratory output was observed by recording the phrenic and vagus nerves activities. When a CBS inhibitor was injected into the preBö tzinger or the Bö tzinger complexes, the phrenic nerve amplitude decreased, and the respiratory frequency increased. The effects on the balance between the inspiratory and expiratory phase durations differed between CBS inhibitions in the preBö tzinger and the Bö tzinger complexes. Inhibition of CBS in the preBö tzinger complex maintained the balance because both phases shortened. On the other hand, CBS inhibition in the Bö tzinger complex altered the balance because of less change in the duration of inspiration than expiration. These results suggested that hydrogen sulfide in the respiratory center can facilitate the power of respiration and sustain the duration. This indicates that hydrogen sulfide may have differential functional roles depending on regions in the respiratory center.

### [2P07-02]
### Activation of the lateral habenula induces stress-induced respiratory responses in rats.

*Riko Mizukami[1,2,3], Masayuki Matsumoto[1,4], Tadachika Koganezawa[2,4] (*[1]Department of Cognitive and Behavioral Neuroscience, Faculty of Medicine, University of Tsukuba, [2]Department of Neurophysiology, Faculty of Medicine, University of Tsukuba, [3]Master's program in Neurosciences, Graduate School of Comprehensive Human Sciences, University of Tsukuba, [4]Transborder Medical Research Center, University of Tsukuba*)

Phycological stress triggers a variety of responses. It induces behavioral reactions such as the fight or flight response and the freezing and produces autonomic changes, cardiovascular and respiratory responses. Recent studies have suggested that the lateral habenula (LHb), excited by aversive stress, is one of the critical brain regions for the autonomic cardiovascular changes in phycological stress. However, the involvement of the LHb in neurogenic respiratory regulation is still unclear. In this study, we hypothesized that the LHb regulates stress-induced respiratory responses by controlling the respiratory center in the brainstem. To approach this hypothesis, we employed urethane-anesthetized rats, activated the LHb by electrical stimulation, and observed the effects on respiratory activity. As a result, activation of the LHb increased the respiratory frequency and the amplitude of inspiratory movement, which is also observed in stress-induced respiratory responses. Moreover, these changes were dependent on stimulus intensity. These results suggested that the LHb neurogenically modulates respiratory activity. The LHb-respiratory center circuits may be essential for respiratory regulation in phycological stress.
(COI: No)

### [2P07-03]
### Effects of PAR1 activation on respiratory rhythm generation in the ventrolateral medulla of newborn rats

*Hiroshi Onimaru[1], Isao Fukushi[2], Keiko Ikeda[3], Itaru Yazawa[4], Kotaro Takeda[5], Yasumasa Okada[6], Masahiko Izumizaki[1] (*[1]Department of Physiology, Showa University School of Medicine, [2]Faculty of Health Sciences, Uekusa Gakuen University, [3]Dept Oral Physiol, Showa Univ. Sch. of Dent., [4]Dept Food & Nutrition, Kyushu Nutrition & Welfare Univ, [5]School of Healthcare, Fujita Health University, [6]Clinical Research Center, Murayama Medical Center*)

Proteinase-activated receptor-1 (PAR1) is expressed in astrocytes of various brain regions, and is involved in the modulation of synaptic activity. Here we report the effects of PAR1- selective agonist TFLLR on respiratory rhythm generation in the brainstem-spinal cord preparation. The preparation was isolated from newborn rats (P0-P4) under deep isoflurane anesthesia, transversely cut at various levels of the rostral medulla, and superfused with the artificial cerebrospinal fluid (25-26℃). The inspiratory C4 ventral root activity was monitored. The cellular responses were detected by calcium imagings or membrane potential recordings. Application of 10 μM TFLLR induced a transient increase of calcium signal in cells of the ventrolateral medulla. More than 70% of responding cells were also activated by low (0.2 mM) $K^+$ solution, suggesting that they were astrocytes. Respiratory related neurons in the ventrolateral medulla close to the cut surface showed transient membrane hyperpolarization (-2 to -4 mV) and C4 burst rate decreased transiently during 10 μM TFLLR. In conclusion, activation of astrocytes via PAR1 resulted in transient hyperpolarization of respiratory related neurons in the ventrolateral medulla in association with C4 rate decrease.
COI: No

### [2P07-04]
### Effect of dexmedetomidine on cardiorespiratory regulation in spontaneously breathing adult rats

*Yoichiro Kitajima[1], Nana Hashizume[1], Chikako Saiki[1], Ryoji Ide[1], Toshio Imai[1], Eishi Nakamura[1] (*[1]Department of Physiology, The Nippon Dental University School of Life Dentistry at Tokyo*)

Abstract
**Purpose** We examined the cardiorespiratory effect of dexmedetomidine, an $\alpha_2$- adrenoceptor / imidazoline 1 ($I_1$)receptor agonist, in spontaneously breathing adult rats.
**Methods** Male rats (226-301g, n = 49) under isoflurane anesthesia had their tail vein cannulated for drug administration and their tail artery cannulated for analysis of mean arterial pressure (MAP), pulse rate (PR), and arterial blood gases ($PaO_2$, $PaCO_2$, pH).After recovery, one set of rats received normal saline for control recording and was then divided into three experimental groups, two receiving dexmedetomidine (5 or 50 $\mu g \cdot kg^{-1}$) and one receiving normal saline (n = 7, per group). Another set of rats was divided into four groups receiving dexmedetomidine (50 $\mu g \cdot kg^{-1}$) followed 5 min later by 0.5 or 1 $mg \cdot kg^{-1}$ atipamezole (selective $\alpha_2$-adrenoceptor antagonist) or efaroxan ($\alpha_2$- adrenoceptor/$I_1$ receptor antagonist) (n = 6 or 8, per group). Recordings were performed 15 min after normal saline or dexmedetomidine administration. **Results** Compared with normal saline, dexmedetomidine (5 and 50 $\mu g \cdot kg^{-1}$) decreased respiratory frequency ($f_R$, p = 0.04 and < 0.01, respectively), PR (both p < 0.01), and $PaO_2$ (p = 0.04 and < 0.01), and increased tidal volume (both p = 0.049). Dexmedetomidine at 5 $\mu g \cdot kg^{-1}$ did not significantly change minute ventilation ($V'_E$) (p = 0.87) or MAP (p = 0.24), whereas dexmedetomidine at 50 $\mu g \cdot kg^{-1}$ significantly decreased $V'_E$ (p = 0.03) and increased MAP (p < 0.01). Only dexmedetomidine at 50 $\mu g \cdot kg^{-1}$ increased $PaCO_2$ (p < 0.01). Dexmedetomidine (5 and 50 $\mu g \cdot kg^{-1}$) significantly increased blood glucose (p < 0.01), and dexmedetomidine at 50 $\mu g \cdot kg^{-1}$ increased hemoglobin (p 0.04). Supplemental atipamezole or efaroxan administration similarly prevented the 50 $\mu g \cdot kg^{-1}$ dexmedetomidine-related cardiorespiratory changes. **Principal conclusion** These results suggest that dexmedetomidine-related and simultaneously observed hypoventilation and hypertension occur predominantly through activation of $\alpha_2$-adrenoceptors, but not $I_1$ receptors, in spontaneously breathing adult rats.

### [2P07-05]
### In vitro generation of goblet cell metaplasia model using iPS cell-derived airway epithelium

*Susumu Yoshie[1], Shingo Tsuji[1], Akihiro Hazama[1] (*[1]Department of Cellular and Integrative Physiology, School of Medicine, Fukushima Medical University*)

[Background] Goblet cell metaplasia caused by asthma and habitual cigarette smoking leads to excessive mucus production and airway obstruction. However, the pathogenic mechanism of goblet cell metaplasia has not yet been fully elucidated. The aim of this study is to generate goblet cell metaplasia model using iPS cell-derived airway epithelium in order to elucidate the pathogenic mechanism of goblet cell metaplasia.[Methods] We generated airway epithelium via spheroid formed from iPS cells based on serum-free conditions. Goblet cell metaplasia model was generated from iPS cell-derived airway epithelium by the use of cigarette smoking solution. [Results] Airway epithelium generated from iPS cells expressed airway epithelium markers and had functional characteristics such as ciliary movement and $Cl^-$ transport. Furthermore, iPS cell-derived airway epithelium treated with cigarette smoking solution strongly expressed goblet cell markers. Mucin-positive cells were also appeared.[Conclusions] We succeeded in the generation of goblet cell metaplasia model from iPS cell-derived airway epithelium.

### [2P07-06]

### Inverse regulation of claudin-2 and claudin-7 expressions by differentiation in colon epithelial cells

Chieko Hirota[1], Yui Takashina[1], Naotaka Ikumi[2], Noriko Ishizuka[2], Hisayoshi Hayashi[2], Yoshiaki Tabuchi[3], Yuta Yoshino[1], Toshiyuki Matsunaga[1], *Akira Ikari[1] ([1]*Gifu Pharmaceutical University,* [2]*University of Shizuoka,* [3]*University of Toyama*)

Colonic epithelial cells are differentiated into absorptive or secretory cells during moving along the crypt villus axis. The absorption of $Na^+$ and $Cl^-$ is regulated by various ion channels and transporters, but the involvement of tight junction remains unknown. Claudin-7 (CLDN7), a tight junctional protein, is mainly located at the surface of crypt, whereas CLDN2 is at the bottom. The expression levels of CLDN2 and CLDN7 were altered depending on the culture days in mouse colonic MCE301 cells. The nuclear levels of p53 and HNF4$\alpha$ were increased depending on the culture days. Tenovin-1 (TEN), a p53 activator, increased the nuclear levels of p53 and HNF4$\alpha$. The mRNA expression and promoter activity of CLDN7 were upregulated by TEN, whereas those of CLDN2 were downregulated. The changes of CLDN2 and CLDN7 expressions were inhibited by siRNA against p53 and HNF4$\alpha$. The interaction of p53 with HNF4$\alpha$ was elevated by TEN. Although paracellular fluxes of FITC-labeled dextran were unchanged by TEN, transepithelial electrical resistance was decreased. Ussing chamber assay showed that TEN increases the ratio of permeability of Cl- to $Na^+$. We suggest that p53 and HNF4$\alpha$ alter the paracellular permeability of $Cl^-$ to $Na^+$ mediated by the inverse regulation of CLDN2 and CLDN7 expression in the crypt of colon.

### [2P07-07]

### Neural targets of dopamine in regulating motility of rat proximal colon

*Hiroyuki Nakamori[1], Hikaru Hashitani[1] ([1]*Department of Cell Physiology, Nagoya City University Graduate School of Medical Sciences*)

In the rat proximal colon, the dopamine reuptake inhibitor GBR 12909 (GBR) dilates colonic segments, while the $D_1$-like receptor antagonist SCH 23390 (SCH) causes a tonic constriction, and thus neurally-released dopamine appears to stimulate inhibitory neurons. Here, precise targets of dopaminergic innervation were investigated. Cannulated segments of rat proximal colon were luminally perfused with 0.9% saline, while serosally perfused with Krebs solution. All drugs were applied serosally. Spatio-temporal maps of diameter changes were constructed from video recordings, and the maximum diameter of colonic segments was measured. GBR increased colonic diameters in an SCH-sensitive manner, but failed to dilate colonic segments that had been pretreated with L-nitro arginine (L-NA), a nitric oxide synthase inhibitor or tetrodotoxin (TTX). In contrast, neither L-NA nor TTX prevented the SCH-induced constriction. In colonic segments isolated from 6- hydroxydopamine treated rats in which enteric dopamine was expected to be depleted, GBR failed to increase the colonic diameter, while SCH was still capable of constricting colonic segments. Thus, enteric dopaminergic neurons may project to nitrergic neurons to dilate the proximal colon via $D_1$-like receptors. In addition, there seems to be constitutively activated enteric $D_1$-like receptors that appear to counteract with colonic constrictions.

### [2P07-08]

### Suppression of gastric reservoir function induced by oxytocin via dorsal medulla.

*Motoi Kobashi[1], Yuichi Shimatani[2], Masako Fujita[1], Yoshihiro Mitoh[1], Ryusuke yoshida[1] ([1]*Dept Oral Physiol, Okayama Univ Grad Sch Med Dent Pharm Sci,* [2]*Dept. Medical Engin., Fac. Sci. Engin., Tokyo City Univ.*)

Our previous studies revealed that appetite-enhancing peptides facilitated phasic contractions of the distal stomach and induced relaxation of the proximal stomach via the dorsal vagal complex (DVC). The enhanced contraction of the distal stomach facilitates gastric emptying, and relaxation of the proximal stomach facilitates the accommodation of swallowed food. Oppositely, anorexigenic peptides, such as oxytocin and glucagon-like peptide-1, suppressed phasic contractions of the distal stomach and increased the intragastric pressure (IGP) of the proximal stomach. In the present study, we examined the microinjection of oxytocin into the DVC to identify the effective sites to induced specific proximal stomach motility in anesthetized rats. Microinjection of oxytocin into the area postrema or the medial part of the nucleus tractus solitarius (mNTS) increased the IGP of the proximal stomach. Obvious change in motility was not observed when oxytocin was injected into the commissural part of the NTS nor the dorsal motor nucleus of the vagus. These results show that oxytocin induced proximal stomach motility via multiple nuclei of the DVC. The experimental protocols were approved by the Okayama University Animal Use Committee. This work was supported by JSPS KAKENHI Grant Number 18K11099. The authors declare no conflicts of interest associated with this manuscript.

### [2P07-09]

### Accessory cholera enterotoxin activates KCNQ channels in intestinal epithelial cells

*Mikio Hayashi[1], Kazi Mirajul Hoque[2] ([1]*Kansai Medical University,* [2]*University of Maryland School of Medicine*)

Vibrio cholera accessory enterotoxin (Ace), as well as cholera toxin and zonula occludens toxin, causes the endemic disease cholera. Ace has been shown to contribute to diarrhea by stimulating $Cl^-$ secretion through anoctamin 6 $Cl^-$ channels. However, the effect of Ace on the intestines has not been extensively investigated. Thus, the present study aimed to identify ion channels involved in the stimulation of secretion by the action of Ace. We performed whole-cell recording in Caco-2 single cells using gramicidin-perforated patch techniques. The application of 1 $\mu$M Ace increased slope conductance, which was inhibited by 5 mM $Ba^{2+}$, a non-specific $K^+$ channel inhibitor. A KCNQ-type $K^+$ channel inhibitor inhibited the outward $K^+$ conductance in a concentration-dependent manner. Immunofluorescence ascribed to KCNQ3 and KCNE2 localized to the luminal membrane of Caco-2 monolayers. In the mouse ileum, immunofluorescence ascribed to KCNQ3, KCNQ4, and KCNE2 localized to the luminal membrane of surface cells. These results indicate that KCNQ-type $K^+$ channels contribute to Ace stimulated $K^+$ secretion in intestinal epithelial cells.

### [2P07-10]

### Heat stable enterotoxin STb increases epithelial barrier function via a neural reflex of enteric nervous system involved in TTX-resistant voltage-gated Na channels $Na_V$s in mice small intestine

*Mao Ikeya[1], Kota Tsukamoto[1], Yuichi Suzuki[2], Shin-ichiro Karaki[1] ([1]*University of shizuoka,* [2]*Sendai Seiyo Gakuin College*)

"Leaky gut syndrome" is thought to be caused by a decrease in intestinal barrier function, and has become a hot topic in relation to disorders, such as allergic and autoimmune diseases. Enteric nervous system (ENS) regulates a variety of intestinal physiological functions. However, little is known about the control of intestinal barrier function by ENS. Therefore, we investigated the control of intestinal barrier function by ENS using the Ussing chamber. Variety of regions of mouse small intestinal mucosa-submucosal preparations containing intact submucosal plexus were mounted on the Ussing chambers with aluminum electrodes for nerve stimulation, and short-circuit current (*Isc*) and tissue conductance (*Gt*) were continuously measured. Electrical field stimulation (EFS) increased Isc but decreased *Gt* in frequency-dependent manners. The most potent decrease in *Gt* by EFS (5 Hz) were observed in middle region of the small intestine (SI) and followed by jejunum > duodenum > ileum. The EFS-evoked *Gt* decrease in the middle SI was resistant to $10^{-6}$ M of tetrodotoxin (TTX), but was suppressed by lidocaine ($10^{-3}$ M). In addition,the heat-stable enterotoxin STb produced by pathogenic *E.coli* evoked an Isc increse and *Gt* decrease when added to the luminal side, simillar to the EFS-induced effects.These responses are suggested to increase water secretion and wash away harmful substances, and might close tight junctions to reduce the permeability of harmful substances for host-defense.

### [2P07-11]

### Short-chain fatty acid-evoked histaminergic transepithelial ion transport in the mice terminal ileum

*Kota Tsukamoto[1], Mao Ikeya[1], Shin-Ichiro Karaki[1] ([1]*Univ. of Shizuoka*)

Reflux of short-chain fatty acids (SCFAs) from cecum to ileum is considered to induce a fluid secretion in the terminal ileum, but the SCFA-induced ion transport in small intestine is not fully understood. We therefore investigated the SCFA-induced ion transport in the mice terminal ileum. Mucosa-submucosal preparations of mouse terminal ileum were mounted on Ussing chambers, and short-circuit current ($I_{sc}$) were measured. Mucosal treatment of acetate and propionate concentration-dependently evoked a biphasic increase in $I_{sc}$ including a fast phase (P-1) achieving peak maximum within 2 min and a second broad phase (P-2) keeping at least more than 30 min. Both phases of the response were insensitive for tetrodotoxin, atropine and piroxicam, but sensitive for amitriptyline and lidocaine, blocking tetrodotoxin-insensitive $Na_V$1.9 channels. Moreover, histamine 1 receptor antagonist pyrilamine attenuated the acetate-evoked P-1 increase in $I_{sc}$, but not histamine 2 receptor antagonist cimetidine. These results suggested that the SCFAinduced P-1 ion transport in terminal ileum is mediated via enteric nervous system involved in $Na_V$1.9 and histaminergic pathway, and different from the response mediated via cholinergic nerve pathway in distal colon.

### Poster Presentation 2

### [2P08]
### Behabior, Biological rhythm, Sleep

March 17(Thu), 12:00 - 14:00, Zoom P8

### [2P08-01]
**Melinjo (*Gnetum gnemon L.*) seed extract improves sleep quality in diet-induced obesity mice**

*Akira Terao[1], Mao Sato[1], Chiaki Sugiura[1] ([1]*Department of Biology, School of Biological Sciences, Tokai University*)

We investigated the effect of dietary melinjo (*Gnetum gnemon L.*) seed extract (MSE) on sleep architecture in high-fat diet (HFD)-induced obese mice. Forty C57BL/6J male mice were fed different diets for 17 weeks: normal diet (ND), ND with 1% MSE (ND+MSE), HFD, and HFD with 1% MSE (HFD+MSE). Body weight and sleep architecture were examined in all mice after the study period. The body weight of HFD-fed mice increased by 50% compared to that of ND-fed mice. Although HFD did not affect the amount of non-REM (NREM) sleep, the average duration of NREM sleep bout was significantly shortened, and the number of NREM sleep bout was significantly increased. These findings indicate fragmented NREM sleep and altered sleep architecture resulted in impaired sleep quality in HFD-fed mice. Dietary MSE did not affect body weight or sleep architecture in the ND+MSE-fed mice. In contrast, the body weight and sleep architecture of HFD+MSE-fed mice were almost identical to those of ND-fed mice, indicating that dietary MSE completely blocked HFD-induced weight gain and sleep fragmentation. Our data provide compelling evidence that MSE is a novel and promising dietary supplement that restores obesity-induced impaired sleep quality in mice.

### [2P08-02]
**Development and experience-dependent modulation of defensive behaviors to visual threat in mice**

*Madoka Narushima[1], Junichi Nabekura[1] ([1] *National Institute for Physiological Sciences, Division of Homeostatic Development*)

Rodents show defensive behaviors as represented by escape or freezing when they recognize looming shadow above them. Although it has been generally accepted that individual's habitat or experience can modulate phenotype of defensive behavior, effects of systematic manipulation of visual experience on vision-guided defensive behaviors have not been studied. We aimed to describe developmental process of defensive behaviors in response to the visual threat and effect of visual deprivation. We found that occurrence probability of escape response, one of the typical defensive behaviors, increased after P21 and peaked at P28 then continued stable. The occurrence probability of freezing response did not change much during development in our colony. When visual experience was perturbed by dark rearing from P21 for one week, the escape probability clearly decreased whereas the freezing probability increased. Intriguingly, exposure to the looming stimuli at P28 can reverse the suppression to the escape probability at P35. These results clearly indicate that development of defensive behaviors to the looming stimuli is affected by their sensory experience.

### [2P08-03]
**Effect of chronic administration of scopolamine on abnormal behavior in aged mice**

*Kana Tomimoto[1], Yu Takahashi[2], Takeshi Ishihara[2], Hiroshi Ueno[1] ([1]*Kawasaki University of Medical Welfare*, [2]*Kawasaki Medical School*)

Currently, the scopolamine-induced amnesia mouse model is used in many studies to elucidate the cause of dementia and subsequently develop therapeutic agents. In those studies, mature mice were used rather than aged mice. Since most people with dementia are elderly, it is appropriate that the mice used in this model are also elderly. Therefore, in this study, we evaluated whether scopolamine-induced abnormal behavior differs depending on the age of the mice.In this study, C57BL/6J mice aged 14 months and 4 months were used. Scopolamine hydrobromide was dissolved in physiological saline and intraperitoneally administered to mice for 4 weeks. Four weeks later, various behavioral experiments (open field test, elevated plus maze test, Y-maze test, etc.) were conducted.The chronic administration of scopolamine caused different behavioral abnormalities in the 14-month- and 4-month-old mice in some behavioral experiments.These results suggest that although the detailed mechanism of action of scopolamine at different ages in mice remains unclear, chronic administration of scopolamine has different effects depending on the age of the mice.

### [2P08-04]
**The role of the nucleus accumbens in positive emotions**

*Shigetaka Kawashima[1], Fan Lou[1], Jingyang Su[1], Ikue Kusumoto[1], Tomoyuki Kuwaki[1] ([1]*Department of Physiology, Graduate School of Medical and Dental Sciences, Kagoshima University*)

Cataplexy is a kind of the symptoms of type 1 narcolepsy characterized by sudden loss of muscle tone. Cataplexy can be used as a behavioral index of positive emotions since it is triggered by laughter in humans. In our previous study using narcoleptic mice (orexin neuron-ablated mice) and chemogenetic techniques, we found that the nucleus accumbens (NAc) is needed for occurrence of chocolate-induced cataplexy. In this study, we investigated functions of the NAc by using optogenetic technique. Photo-activation of the NAc with channel rhodopsin triggered cataplexy whereas inactivation with archaerhodopsin did not. Spontaneous cataplexy was not inhibited by inactivation of the NAc. The duration of cataplexy behavior was not affected by activation or inactivation of the NAc. Immunohistochemical analysis revealed that photo illumination activated channel rhodopsin-expressing NAc neurons. Thus, the activation of the NAc, whether transient (light stimulation) or persistent (chemical stimulation in our previous study), triggers cataplexy and contributes to the induction but not maintenance of cataplexy. On the other hand, result with optogenetic inhibition of the NAc (no effect on cataplexy in this study) was different form that with chemogenetic inhibition (reduction of cataplexy) in our previous study. We conclude that the brain mechanisms for temporary emotion and persistent mood are partly different.

### [2P08-05]
**Physiological function of prostaglandin $E_2$-induced long lasting inhibition of noradrenergic neurons in the locus coeruleus**

*Yasutaka Mukai[1,2], Michael Lazarus[3], Takeharu Nagai[4], Kenji Tanaka[5], Akihiro Yamanaka[1,2] ([1]*Dept of Neuroscience II, RIEM, Nagoya Univ*, [2]*Dept of Neural Regulation, Grad Sch Med, Nagoya Univ*, [3]*WPI-IIIS, Tsukuba Univ*, [4]*Dept Biomol Sci and Eng, ISIR, Osaka Univ*, [5]*Dept of Neuropsychiatry, Sch of Med, Keio Univ*)

Noradrenergic neurons in the locus coeruleus (LC-NA neurons) have multiple physiological functions, such as wakefulness, attention and memory. Time duration required for these function ranges from seconds or minutes to hours or even days. However, bioactive substances which affect the activity in minutes- to hours-time scale have been still elusive.Recently we developed a screening method to identify substances which affect the activity of specific neurons in minutes- to hours-time scale (Mukai *et al.*, *Sci Rep*, 2020). By using the method, we screened 57 substances in LC-NA neurons, and found that prostaglandin $E_2$ ($PGE_2$; 100 $\mu$M) strongly and long lastingly decreased $[Ca^{2+}]_i$ for more than an hour. We further explored for a responsible receptor of $PGE_2$, and found prostaglandin EP3 receptor (EP3R) is involved in the long-lasting decrease of $[Ca^{2+}]_i$ in LC-NA neurons.In the brain, $PGE_2$ is known to be produced by psychological and illness stressors. Therefore, we hypothesized that $PGE_2$ suppresses the activity of LC-NA neurons via EP3R under stress conditions. To reveal the physiological functions, we generated mice in which EP3R in the LCNA neurons is conditionally knocked out (cKO) by using *EP3R*-floxed mice strain crossed with noradrenaline transporter (*NAT*)-*Cre* strain.We performed several behavioral experiments in cKO with application of stressors. Among them, we found that restraint stress induced longer immobile time in female cKO, and the total amount of rapid-eye movement (REM) sleep in the light phase was shorter in male cKO. We would like to introduce and discuss about our ongoing preliminary results.

### [2P08-06]

### Non-photic entrainemnt of the circadian clock in mice by scheduled exposures to a novel environment with a running-wheel

*Yujiro Yamanaka[1], Ren Sato[2] ([1]*Hokkaido University, Fuclty of Education and Graduate School of Education, Laboratory of Life and Health Sciences,* [2]*Hokkaido University, School of Education*)

The aim of the present study was to examine whether entrainment by daily exercise by scheduled exposure to a new cage with a running-wheel (NCRW) entrain the central circadian pacemaker in the suprachiasmatic nucleus (SCN) and internal-temporal order in behavior and circadian clock in the SCN and peripheral tissues. We used adult male *Per1- luc* transgenic mice of C57BL6 background. The mice were individually housed in a cage without a running wheel and kept under LD12:12 for 2 weeks, then released into constant darkness (DD) for 4 weeks.
After the establishment of steady-state free-running, the cage was exchanged with a new cage equipped with a running-wheel for 3-h. This scheduled exposure to NCRW was implemented for 10-12 weeks with a fixed period of 24-h. We measured the *Per1-luc* rhythms in the anterior and posterior SCN, arcuate nucleus, liver, and skeletal muscle under LD, DD after the establishment of steady-state free-running, and steady-state entrainment to the scheduled exposure to the NCRW under DD. As a result, circadian behavior rhythms in all mice could entrain the scheduled exposure to the NCRW. The Phase angle difference between the circadian behavior rhythm and *Per1-luc* peaks in the SCN was stable under LD, DD and entrainment to the NCRW, suggesting that the rhythm of the SCN could entrain to the daily exposure to NCRW under DD (non-photic entrainment). A possible role of 3-h daily exposure to the NCRW was demonstrated as the non-photic zeitgeber.

### [2P08-07]

### Roles of GABA signaling in AVP neurons on female reproductive functions

*Mizuki Sugiyama[1], Jiaxu Chen[1], Michihiro Mieda[2], Takahiro Nakamura[1] ([1]*Laboratory of Animal Physiology, School of Agriculture, Meiji University,* [2] *Department of Integrative Neurophysiology, Faculty of Medicine, Kanazawa University*)

The estrous cycle is 4-5 days in female mice, and it is divided into four stages; metestrus, diestrus, proestrus, estrus. During the late afternoon of proestrus, ovulation is caused by luteinizing hormone (LH) surge following gonadotropin-releasing hormone (GnRH) surge. It has been reported that the LH surge did not occur in rodents when the suprachiasmatic nucleus (SCN) was lesioned. It suggests that the preovulatory GnRH/LH surge requires high levels of estrogen and timing signals from the SCN. Although it has been suggested that peptides-producing neurons such as arginine vasopressin (AVP) and vasoactive intestinal peptide are related to the GnRH/LH surge system, the details of the mechanism are still unclear. In the present study, *Avp-Vgat*$^{-/-}$ (*Avp-Cre; Vgat*$^{flox/flox}$) mice, the vesicular GABA transporter (*Vgat*) gene is specifically deleted in AVP producing neurons, were used. The estrous cycle (vaginal smear and wheel-running activity) of *Avp-Vgat*$^{-/-}$ mice was obscured compared to control (*No-cre;Vgat*$^{flox/flox}$) mice. The obscured estrous cycle of *Avp-Vgat*$^{-/-}$ mice was restored by injection of adeno-associated virus (AAV- *EF1α-DIO-Vgat- mcherry*) to the SCN. These results suggest that GABAergic transmission from AVP neurons in the SCN has important roles on female reproductive functions in mice.

### [2P08-08]

### Roles of TRPV1 mechanism for enhanced ocular surface and intraoral nociception in a rat model of obstructive sleep apnea

*Ayano Katagiri[1], Hiroki Toyoda[1], Takafumi Kato[1] ([1]*Osaka University Graduate School of Dentistry Department of Oral Physiology*)

Obstructive sleep apnea (OSA) is associated with an increased risk of orofacial pain. This study aimed to determine the role of transient receptor potential vanilloid 1 (TRPV1) in mediating enhanced orofacial nocifensive behavior and trigeminal spinal subnucleus caudalis (Vc) neuronal responses to capsaicin stimulation in a rat model of OSA. Rats were subjected to chronic intermittent hypoxia (CIH) during the light phase for 8 or 16 consecutive days. CIH yielded enhanced behavioral responses to capsaicin, a TRPV1 agonist, after application to the ocular surface and intraoral mucosa. The percentage of TRPV1-immunoreactive trigeminal ganglion (TG) neurons was greater in CIH rats than in normoxic rats. The density of TRPV1 positive primary afferents in the superficial laminae of Vc was higher in CIH rats. The number of pERK-immunoreactive cells following capsaicin application to the tongue was significantly greater in the middle portion of the Vc of CIH rats than in normoxic rats. These changes were reversed under normoxic conditions. Present data suggest that CIH is sufficient to transiently enhance pain on the ocular surface and intraoral mucosa via TRPV1-dependent mechanisms.

### [2P08-09]

### Analysis of sleep-wake behavior and c-Fos expression in the brain of African native Nile grass rats (*Arvicanthis niloticus*)

*Shoya Ikeda[1], Nakagomi Haruka[2], Tamogami Sakura[1], Okeya Miho[2], Koizumi Hayato[3], Morioka Eri[1], Mochizuki Takatoshi[1], Ikeda Masayuki[1,3] ([1]*Graduate School of Science and Engineering, University of Toyama,* [2]*School of Science, University of Toyama,* [3]*Graduate School of Innovative Life Science, University of Toyama*)

Currently, most neurobiological and neurophysiological studies of sleep-wake behavior are performed using laboratory rodents (rats and mice), however, they are nocturnal animals and have opposite circadian phase of activity/rest period to diurnal animals including humans. Basic sleep-wake studies in diurnal animals are limited yet, thus more knowledge about neuronal systems regulating "diurnal" sleep-wake behavior is needed. In this study, we successfully established a laboratory colony of African grass rat (*Arvicanthis niloticus*, Nile grass rat), known as a diurnal animal, and analyzed their sleep-wake behavior under 12:12 hour light-dark cycle, with different light intensity at the light period (10, 100, 1000 lux).We also studied c-Fos expression of these animals to characterize sleep- or wake-active neurons in the brain.
We applied and optimized a standard electroencephalogram (EEG) and electromyogram (EMG) recording technique to analyze sleep-wake behavior. Because their EEG power spectra during sleep were similar to those of laboratory rodents, we quantified wake, rapid eye movement (REM) sleep, and non-REM sleep in a standard manner. They had more amount of wake in the light period than the dark period, and the day time wake amount increased in the illuminance-dependent manner. The amount of sleep in the dark period was the same across all illumination conditions. In the light period, they had significant c-Fos expression in the suprachiasmatic nucleus, and more results of histology will be shown in the presentation.

### [2P08-10]

### Search for reliable behavioral indicators capable of detecting abnormalities induced by developmental methylmercury exposure

*Fumihiko Maekawa[1], Toshihiro Endo[2], Masaki Kakeyama[3] ([1]*National Institute for Environmental Studies,* [2]*Phenovance LLC,* [3]*Waseda University*)

As shown in fetal Minamata disease, exposure to methylmercury during the developmental period can interfere with brain development and cause behavioral abnormalities. The current environmental reference values for methylmercury have been determined based on past incidences in pollution cases, but the results of developmental neurotoxicity assessments using animal models have not been utilized in setting values. One of the reasons is the insufficient development of standardized and reliable behavioral tests and behavioral indices that can detect the minimum toxic dose inducing behavioral abnormalities. We believe that the establishment of a more effective behavioral testing framework and behavioral effect assessment endpoints will not only be useful for re-examining known chemicals such as methylmercury, but also for refining the assessment of newly produced chemicals that are of concern for developmental neurotoxicity. In this study, we conducted a behavioral experiment using IntelliCage, which can fully automate the evaluation of mouse behavior, under the condition that three institutions were remotely connected via Internet and controlled simultaneously. Exposure to methylmercury was taken place at the National Institute for Environmental Studies, and pregnant B6 mice were orally exposed twice at three doses (0, 0.5, and 5 mg/kg bw) on gestation days 7 and 14. After the exposed offspring were grown, they were transported and evaluated at each institution. Although the evaluation of the data has not been completed at this time, the data indicate a decrease in several parameters of the activity index group. We have also found that the impulsive index in the behavioral flexibility task may be useful as an endpoint to detect methylmercury effects. We are further investigating a statistical method that can be used to integrate exposure assessment data from the three organizations.

### [2P08-11]

### *Vrk2* deficiency promotes aggressive behavior in female zebrafish

*Ryohei Umeda[1], Nobuyuki Shimizu[2], Kazumasa Hada[2], Hitoshi Teranishi[1], Kenshiro Shikano[1], Ryoko Higa[1], Hirotaro Urushibata[2], Hiroshi Shiraishi[2], Toshikatsu Hanada[2], Reiko Hanada[1] ([1]*Oita university Faculty of Medicine Department of Neurophysiology,* [2]*Oita university Faculty of Medicine Department of Cell Biology*)

Vaccinia-related kinase 2 (VRK2) is a serine/threonine kinase that was originally identified in highly proliferative cells such as thymocytes and fetal liver cells. *VRK2* is also expressed in the brain; however, its molecular function *in vivo* is mostly unknown. Many genome-wide association studies (GWASs) have reported that *VRK2* is a potential candidate molecule for neuropsychiatric diseases such as schizophrenia in humans. However, the pathophysiological relationship between *VRK2* and neuropsychiatric disorders are not clearly understood. In this study, we established *vrk2* deficient (VRK2 KO) zebrafish and found that their forebrain size was larger than that of the control zebrafish. In behavior analysis, it became clear that female VRK2 KO shows more aggressive than wild type zebrafish. Additionally, VRK2 KO female zebrafish showed low gamma-aminobutyric acid (GABA) content in the brain and high density of neuronal dendrites when compared with the wild type zebrafish. These findings suggest that female VRK2 KO zebrafish could be a model of aggression, which may be attributed to the low levels of GABA content in their brain.

### Poster Presentation 2

### [2P09]
### Behabior, Biological rhythm, Sleep, Pathophysiology

March 17(Thu), 12:00 - 14:00, Zoom P9

### [2P09-01]
### Study on the BDNF output from the SCN which is a candidate of diffusible factors to drive the circadian rhythmicity

*Shota Miyazaki[1,2], Wataru Nakamura[3], Takahiro J. Nakamura[1]
([1]*Laboratory of Animal Physiology, School of Agriculture, Meiji University,* [2]*Major in Life Science, Graduate School of Agriculture, Meiji University,* [3]*Department of Oral Chrono-Physiology, Graduate School of Biomedical Sciences, Nagasaki University*)

In mammals, the generation of behavioral and physiological rhythms and the entrainment of these rhythms to the light-dark cycle are mediated by the suprachiasmatic nucleus (SCN) of the hypothalamus. However, the mechanism of the SCN outputs to regulate rhythmic events is still unknown. Recently, we confirmed that circadian rhythms of locomotor and physiological functions did not disappear in "the isolation of the SCN (iSCN) mice" whose neural outputs from the SCN were severed. This suggests that some outputs from the SCN not depending upon physical connections are driving the circadian rhythmicity. In the present study, we examined the brain-derived neurotrophic factor (BDNF) output from the SCN which is known as a diffusible protein in circadian locomotor rhythms. We generated *Bdnf* cKO mice as *Vgat-Cre* mice crossed with *Bdnf-floxed* mice. *Bdnf* cKO mice are lost of the ability of BDNF outputs from the SCN because almost all neurons of the SCN are GABAergic. Contrary to our hypothesis, *Bdnf* cKO mice showed normal circadian locomotor rhythms. There were no significant differences in activity levels and circadian free-running periods between control and *Bdnf* cKO mice. Next, we employed iSCN surgery on *Bdnf* cKO mice, considering the potentials of circadian rhythms by neural connections of the SCN. *Bdnf* cKO: iSCN mice also showed normal circadian locomotor rhythms. These results suggest that the BDNF output from the SCN are not essential for driving the rhythms in locomotor activity, thus BDNF is not a critical diffusible factor to circadian rhythms.

### [2P09-02]
### AVP neurons of the SCN act as the principal circadian pacemaker cells *in vivo*

*Yusuke Tsuno[1], Yubo Peng[1], Takiko Daikoku[2], Shin-ichi Horike[3], Kanato Yamagata[4], Takashi Maejima[1], Michihiro Mieda[1] ([1]*Department of Integrative Neurophysiology, Graduate School of Medical Science, Kanazawa University,* [2]*Division of Animal Disease Model, Research Center for Experimental Modeling of Human Disease, Graduate School of Medical Science, Kanazawa University,* [3]*Division of Integrated Omics research, Research Center for Experimental Modeling of Human Disease, Kanazawa University,* [4]*Child Brain Project, Tokyo Metropolitan Institute of Medical Science*)

The suprachiasmatic nucleus (SCN), the central circadian clock of mammals, is a network consisting of various types of GABAergic neurons, which can be differentiated by the coexpression of specific peptides. We previously demonstrated that lengthened period of cellular clocks in AVP neurons, by the deletion of casein kinase 1 delta (CK1$\delta$) (*Avp-CK1*$\delta^{-/-}$), elongated the free-running period of circadian behavior.
To examine how much AVP neurons contribute to the circadian period-setting, we compared the behavioral free-running period of mice lacking CK1$\delta$ in the whole SCN (*Camk2a-CK1*$\delta^{-/-}$) with that of *Avp-CK1*$\delta^{-/-}$ mice, resulting in no significant difference. However, PER2::LUC reporter rhythm in SCN slices of *Avp-CK1*$\delta^{-/-}$ mice did not fully recapitulate the period lengthening, underscoring the importance of *in vivo* analysis. The *in vivo* $[Ca^{2+}]_i$ of AVP neurons and VIP neurons in the SCN of *Avp-CK1*$\delta^{-/-}$ mice, measured by fiber photometry, demonstrated circadian rhythms with lengthened periods similar to that of behavioral rhythm. These results support the hypothesis that the cellular circadian period of AVP neurons is the primary determinant of the ensemble period of the SCN network.

### [2P09-03]
### Activation of the central amygdala neurons attenuates aversion and increases access to a conditioned aversive taste stimulus in mice

*Tadashi Inui[1], Emi Kikuchi[1,2], Makoto Funahashi[1] ([1]*Department of Oral Physiology, Graduate School of Dental Medicine, Hokkaido University,* [2]*Department of Orthodontics, Division of Dental Medicine, Graduate School of Dental Medicine, Hokkaido University*)

Retrieval of conditioned taste aversion (CTA) suppresses intake of a taste solution (conditioned stimulus, CS). To investigate the role of the central amygdala (CeA) in CTA retrieval, we examined the effects of activation of the CeA neurons on the behavioral responses to the aversive CS. Male C57/BL6 mice were bilaterally injected with AAV8-hSyn-hM3Dq-mCherry, and then received a pairing of 0.2% saccharin solution as a CS with 0.3 M lithium chloride. After the conditioning, they were exposed to the CS 90 min after an i.p. administration of a designer drug deschloroclozapine (DCZ, 50 $\mu$g/kg) or vehicle (1 % DMSO in saline). The mice with DCZ drank a larger volume of the CS than those with the vehicle. The microstructural licking behavior analysis showed the augmentation of burst licking (an index of taste palatability) in the mice with DCZ. Moreover, the approach behavior analysis demonstrated that the DCZ increased the frequency of access to the CS. These results suggest that the activation of the CeA neurons attenuates distaste and fear for the CS during the CTA retrieval.
COI: No

### [2P09-04]
### The inhibition of the neuronal activity in the bed nucleus of the stria terminalis greatly reduces a conditioned stimulus intake on the retrieval of conditioned taste aversion

*Emi Kikuchi[1,2], Tadashi Inui[1], Shaoyi Su[1], Makoto Funahashi[1] ([1]*Department of Oral Physiology, Graduate School of Dental Medicine, Hokkaido University,* [2]*Department of Orthodontics, Division of Dental Medicine, Graduate School of Dental Medicine, Hokkaido University*)

Conditioned taste aversion (CTA) decreases the palatability of a taste solution (a conditioned stimulus, CS) and induces fear of consuming the CS. The bed nucleus of the stria terminalis (BNST) is involved in states of fear. However, the role of the BNST in the CTA remains unclear. We assessed the effects of chemogenetic inhibition of the BNST neurons on the retrieval of CTA. Male mice receiving AAV8-hSyn-hM4Di-mCherry (0.5 $\mu$l/ side) in the BNST were conditioned with a paring of a 0.2% saccharin solution and 0.3 M lithium chloride (2% BW, i.p.) in a chamber. After the conditioning, the i.p. injection of clozapine.N.oxide (CNO; 1 mg/kg, n=7) severely suppressed CS intake compared to the saline injection (n=7) ($p < 0.01$). After the CTA test, we found that the CNO moderately reduced water intake in the chamber where the mice experienced the traumatic event (conditioning), suggesting that the CNO amplified fear for the context. Therefore, the inhibition of BNST neurons greatly suppresses CS intake by enhancing the decreased palatability and contextual fear. These results raise the potential for BNST neurons to mediate aversion and fear on the CTA retrieval.
COI: NO

### [2P09-05]
### Activation of insular cortex and lateral hypothalamus caused by food restriction in mice during food anticipatory period

*Jihao Ma[1], Sakurako Yanase[1], Lisa Udagawa[1], Tomoyuki Kuwaki[1], Ikue Kusumoto-Yoshida[1] ([1]*Department of Physiology, Graduate School of Medical and Dental Sciences, Kagoshima University*)

Disorder of feeding behavior will destroy energy balance of body, and may lead to obesity, diabetes and other serious diseases. Therefore, understanding brain mechanisms that underlie well-regulated food intake is critically important.It has been reported that mice fed a single daily meal at intervals within the circadian range exhibit increased locomotor activity in precedent feeding period. However, the neuronal mechanism of the increased locomotor activity is still under discussion. Insular cortex is known as higher order sensory cortex that integrates multiple modalities and plays an important role for food anticipation. It is also well known that lateral hypothalamus regulates both food intake and energy homeostasis. In this study, we observed neuronal activity of insular cortex neurons and lateral hypothalamic orexin neurons during the period to see the role of them in food anticipatory activity (FAA). In this study mice(adult WT C57BL/6 mice) in restricted feeding between ZT4 and ZT8 showed increased locomotor activity just before food available time. In addition, immunohistochemical c-fos signal mapping of both insular cortex and lateral hypothalamus on food available time was adopted and showed increased c-fos signal positive neurons in insular cortex and orexin neurons in lateral hypothalamus. These results suggest insular cortex neurons and lateral hypothalamic orexin neurons are activated during FAA, and suggests their important role in generating food anticipatory behavior.

### [2P09-06]

### Voluntary exercise and food restriction inhibit hyperglycemia but maintain hypoalgesia in OLETF rats

*Ryosuke Ochi[1], Naoto Fujita[1], Kazuyoshi Hisatsune[1], Son Tien Nguyen[1], Hisao Nishijo[2], Susumu Urakawa[1] ([1] *Department of Musculoskeletal Functional Research and Regeneration, Graduate School of Biomedical and Health Sciences, Hiroshima University,* [2]*System Emotional Science, Faculty of Medicine, University of Toyama*)

Otsuka Long-Evans Tokushima fatty (OLETF) rats develop diabetes due to hyperphagia and show hypoalgesia. We aimed to determine whether prevention of hyperglycemia in OLETF rats by voluntary exercise and food restriction at 4-8 weeks of age reversed hypoalgesia. Both interventions inhibited hyperglycemia in OLETF rats. In the hot plate test, voluntary exercise exacerbated hypoalgesia, although food restriction had no effect on hypoalgesia in OLETF rats. Serum corticosterone levels in OLETF rats were reversed by food restriction and voluntary exercise just before and 2 min after the start of the hot plate exposure for 1 min. The numbers of c-Fos-positive cells after the hot plate exposure in the anterior cingulate and prelimbic cortices were not changed by voluntary exercise and food restriction in OLETF rats, whereas those in the infralimbic cortex were decreased by voluntary exercise and food restriction in OLETF rats. These results indicate that although OLETF rats exhibit hypoalgesia regardless of blood glucose level, different mechanisms might be involved in hypoalgesia in normoglycemic and hyperglycemic OLETF rats.

### [2P09-07]

### Brain state effects on cortical signal transmission

*Yuki Donen[1], Ko Matsui[1] ([1]*Tohoku University*)

Biological computation is a fluctuating process which leads to varying conclusions depending on the state of mind. Here, we investigated whether simple signal transmission of a delta pulse of neuronal excitation across the cortex can be influenced by the state of the brain. Using a transgenic rat that expresses photoactivatable channel protein, ChR2, in neurons, a short light pulse was delivered to the occipital lobe via an optical fiber to produce a concerted excitation of a population of neurons. The signal created propagated from the occipital to the frontal lobe following an endogenous pathway of visual signal transmission. Interestingly, in all of the regions recorded, a 24-hour cycle of fEPSP amplitude changes was observed. Amplitude changes in response to a paired-pulse light stimulation also suggested a circadian rhythm of short-term plasticity. After a highfrequency light stimulation of the occipital lobe, the circadian rhythm characteristics changed, which suggests that the rhythm is also susceptible to long-term plasticity changes. Our data suggest that even the simple building blocks of information processing in the brain are influenced by the time of the day. By revealing the mechanisms affecting the signal transmission, we aim to understand how simple fluctuations of basal concentration of transmitter and/or ions can affect higher cognitive brain functions.

### [2P09-08]

### Uptake of tau-containing exosomes by neuronal cells

*Noriko Isoo[1], Kentaro Kawata[1], Naoyuki Iso-o[2], Yukiko Hori[3], Taisuke Tomita[3], Toshihiro Hayashi[1] ([1]*Department of Physiology, Teikyo University School of Medicine,* [2]*Department of Internal Medicine, Teikyo University Mizonokuchi Hospital,* [3]*Laboratory of Neuropathology and Neuroscience, Graduate School of Pharmaceutical Sciences, University of Tokyo*)

The stereotypical propagation of tau protein aggregates in the brain drives the progression of Alzheimer's disease. Recently, exosomes, a class of extracellular vesicles, were shown to contribute to the cell-to-cell transmission of pathological tau proteins in the brain. However, the majority of tau proteins released from neurons into the extracellular space are membrane-free form. To elucidate the roles of exosome-dependent tau protein propagation in the disease progression, we developed a method for the isolation of taucontaining exosomes secreted from mouse neuroblastoma Neuro2a cells. We generated a monoclonal Neuro2a cell line stably expressing full-length tau with the P301S mutation. Exosomes isolated from the culture medium of these cells using differential centrifugation contained a substantial amount of tau proteins. Thereafter, isolated tau-containing exosomes were labeled by DiI, a lipophilic carbocyanine fluorescent dye. Time-lapse live cell imaging demonstrated that a large amount of the DiI-labeled, tau-containing exosomes were taken up by Neuro2a cells within two hours after addition to the cell culture medium. Taken together, the Neuro2a cell-derived, tau-containing exosomes internalized to neuronal cells might contribute to the transcellular transmission of tau proteins.

### [2P09-09]

### Changes in ATP energy dynamics in the brain

*Kota Furukawa[1], Ko Matsui[1] ([1]*Graduate School of Life Sciences Tohoku University*)

Less than one minute cessation of energy supply to the brain can lead to failure of proper neuronal function. It is assumed that most of the energy is used to recover the imbalance of intra and extracellular ions produced by neuronal action potentials. Here, we observed the dynamics of cellular energy *in vivo* using the fiber photometry method in freely moving mice expressing FRET based fluorescent sensor protein for ATP in neurons. A novel method developed in the lab for analyzing the fluorescent signals was used to calculate not only the ATP concentration dynamics but also the changes in the cytosolic pH and the local brain blood volume (BBV). We show here that neuronal hyper-activity caused by hippocampal electrical stimulation led to rapid BBV decrease and neuronal ATP decrease in the hippocampus. Therefore, it is possible that ATP is depleted from neurons upon seizure due to the reduction in energy supply. In the aged mice, hippocampal stimulation did not lead to observable blood vessel constriction. Surprisingly, very little reduction in ATP was observed albeit the presence of a prominent after discharge. Therefore, ion recovery may not be the major factor determining the ATP concentration. Blood and energy supply may govern the mode of neuronal action potential firing, thus, control of local blood vessel constriction may become a new therapeutic target for treating various brain disorders.

### [2P09-10]

### Cellular phenotypes and causal candidate genes in iPSC-derived neurons of a bipolar disorder family

*Gakuya Takamatsu[1,2], Yoko Manome[4], Dimitar Dimitrov[7], Kae Koganebuchi[6], Kanako Toyama[1], Junseok Lee[1], Kumiko Yanagi[5], Minami Hasegawa[4], Tomoko Hayakawa[8], Tsuyoshi Kondo[2], Tomoyuki Takahashi[7], Tadashi Kaname[5], Hirotaka Okano[4], Ryosuke Kimura[3], Masayuki Matsushita[1] ([1]*Dept of Mol Cell Physiol, Grad Sch Med, Univ of the Ryukyus,* [2]*Dept of Neuropsy, Grad Sch Med, Univ of the Ryukyus,* [3]*Dept of Hum Biol Anat, Grad Sch Med, Univ of the Ryukyus,* [4]*Div of Regen Med, Jikei Univ Sch of Med,* [5]*Dept of Gen Med, Natl Ctr for Chd Hlth Dev,* [6]*Dept of Biol Sci, Grad Sch of Sci, Univ of Tokyo,* [7]*Cel and Mol Syn Func Unit, Okinawa Inst of Sci Tech Grad Univ,* [8]*Dept of Phamc, Jichi Med Univ*)

Bipolar disorder (BD) is a common but severe psychiatric disorder. BD is inheritable with estimated heritability for BD of 70-80%; however, identifying genomic variants that strongly contribute to BD is still unprecedented and its molecular pathophysiology is almost unknown. To elucidate the pathogenesis of BD, we performed comprehensive genetic analysis and cellular phenotype analysis focusing on rare familial cases with potential high-risk genetic factors. First, we conducted a pedigree survey in Okinawa, and we found a three-generation multiplex family with BD. Then, we generated patient-derived induced pluripotent stem cells (iPSCs) from affected individuals of the family. Interestingly, iPSC-derived excitatory neurons from the affected members showed higher frequency of the calcium transient compared with neurons from healthy controls. We analyzed allelic imbalances of transcripts by integration of RNA sequencing and whole genome sequencing, and we found haplotype-specific decreased expression of a key mitochondrial regulator gene in the affected individuals of the family. It might contribute to mitochondrial dysfunction and the development of the disease in the family.
(COI: properly declared)

### [2P09-11]

### Regulated KCC2 phosphorylation is critical for dynamic GABA-mediated inhibition

*Miho Watanabe[1], Kristopher Kahle[2], Atsuo Fukuda[1] ([1]*Department of Neurophysiology, Hamamatsu University School of Medicine,* [2]*Departments of Neurosurgery, Yale School of Medicine*)

The $K^+$/$Cl^-$ cotransporter KCC2 is the main $Cl^-$ extrusion mechanism of CNS neurons, and via its role in $Cl^-$ homeostasis is essential for establishment and maintenance of normal GABA-regulated neurotransmission. KCC2 dysfunction has been implicated in seizures, autism, neuropathic pain, and other disorders. However, the regulatory mechanisms of KCC2 are not entirely understood. KCC2 phosphorylation at Thr906 and Thr1007 underwent dephosphorylation in parallel with the GABA excitatory-inhibitory sequence *in vivo*. We previously reported that knockin mice expressing the homozygous phosphomimetic KCC2 mutations T906E/T1007E ($Kcc2^{E/E}$), which prevented the normal developmentally regulated dephosphorylation of these sites, exhibited early postnatal death from respiratory arrest and touch or pain-evoked status epilepticus associated with impaired KCC2-dependent $Cl^-$ extrusion. To further examine the role of phosphorylation in the regulation of KCC2, we generated knockin mice expressing the homozygous dephosphorylation of KCC2 mutations T906A/T1007A ($Kcc2^{A/A}$). $Kcc2^{A/A}$ mice exhibited reduced anxiety, deficit in social novelty recognition, and reduced startle response with enhanced KCC2-dependent $Cl^-$ extrusion. $\gamma$ band power was reduced in resting EEG and susceptibility to pilocarpine induced seizures was increased. These data demonstrated that precisely regulated KCC2 Thr906/Thr1007 phosphorylation is essential for GABAmediated inhibition.
COI: NO

### Poster Presentation 2

### [2P10]
### Pathophysiology

March 17(Thu), 12:00 - 14:00, Zoom P10

### [2P10-01]
### Pathophysiological mechanisms of hyperkalemia-induced ECG abnormalities

*Rei Na Yeoh[1], Yuika Akiyama[1], Momono Senzaki[1], Itsuro Kazama[1] ([1]*Miyagi University, School of Nursing*)

Hyperkalemia is caused by excessive intake or ineffective elimination of potassium ions ($K^+$), or their excessive release from skeletal muscles. It is characterized by typical electrocardiogram (ECG) findings, such as peaked T waves and the widening of QRS complexes. In the present study, we injected potassium chloride (KCl) solutions (1, 10, 100 mM, and 1 M) intravenously into bullfrogs, thus demonstrating the characteristic ECG abnormalities of hyperkalemia in frog hearts. The widened QRS complexes induced by 100 mM KCl injection were accompanied by an increase in the resting membrane potential in cardiomyocytes and a decreased slope of phase 0 in the action potential. Recording both ECG waveforms and the cardiac action potential enabled us to reveal the mechanisms of hyperkalemia-induced ECG abnormalities. Additionally, pre-treatment with insulin, a powerful stimulator of $Na^+/K^+$-ATPase activity, significantly accelerated the recovery from the widened QRS complexes in the ECG, demonstrating a pronounced shift of extracellular $K^+$ ions into the intracellular space.

### [2P10-02]
### High dose caffeine causes QT interval prolongation in ECG

*Yuika Akiyama[1], Rei Na Yeoh[1], Momono Senzaki[1], Itsuro Kazama[1] ([1]*Miyagi University, School of Nursing*)

Caffeine intoxication is caused by excessive consumption of caffeine from drinks, foods or medications. The neurological symptoms include headache, nervousness, restlessness, excitement and increased anxiety. Regarding the common cardiovascular abnormalities, such as tachycardia, irregular heartbeat and cardiac arrhythmia, clinical studies have shown that caffeine intoxication causes the prolongation of QT intervals in electrocardiogram (ECG), which eventually leads to fatal ventricular tachycardia. In the present study, by intravenously injecting various concentrations of caffeine (1, 10, and 100 mM) into bullfrogs, we actually demonstrated that high dose caffeine causes the prolongation of QT intervals in frog hearts. In simultaneous recordings of the cardiac action potential, high dose caffeine prolonged phase 2 in the action potential, indicating the inhibition of the outward potassium currents during this phase. The dual recordings of ECG waveforms and the action potential in cardiomyocytes enabled us to demonstrate the mechanisms of characteristic ECG abnormalities caused by caffeine intoxication.

### [2P10-03]
### Recording electrocardiogram in bullfrog hearts and reproducing findings of acute myocardial infarction

*Ryo Kuwana[1], Itsuro Kazama[1] ([1]*Miyagi University, School of Nursing*)

By surgically ligating coronary arteries, animal models of ischemic heart disease have been created in rodents. However, the use of these animals has been restricted to highly specialized laboratories due to technical difficulties. Alternatively, using isolated bullfrog hearts, previous studies revealed electrophysiological properties of cardiac muscles. In the present study, by deeply anesthetizing bullfrogs and surgically exposing their hearts, we directly recorded the electrocardiogram (ECG) and the action potential of cardiomyocytes. The frog heart ECG represented an identical pattern to that of humans or rodents, showing its usefulness as a mimic of the human heart. Additionally, by creating burn injuries on the frog heart ventricle, we reproduced prominent ST segment elevation, mimicking the findings of acute myocardial infarction in humans. Thus, bullfrog hearts were thought to be suitable for reproducing ECG changes observed in human heart disease.

### [2P10-04]
### Urinary biomarkers in ischemia/reperfusion-induced renal injury

*Keiko Hosohata[1], Denan Jin[2], Shinji Takai[2] ([1]*Osaka Medical and Pharmaceutical University*, [2]*Osaka Medical and Pharmaceutical University*)

Acute kidney injury (AKI) is associated with risk of developing chronic kidney disease (CKD). In the pathophysiology of AKI, oxidative stress plays a pivotal role. Previously, we reported that vanin-1, which is involved in oxidative stress, is associated with renal tubular injury. This study aims to determine whether urinary vanin-1 is a biomarker for early diagnosis of AKI in two experimental models: in vivo and in vitro. In a rat model of AKI induced by ischemia/reperfusion (I/R), ischemic AKI was induced in uninephrectomized rats by clamping the left renal artery for 45 min and then reperfusing the kidney. After 1 day of the treatment, urinary N-acetyl-β-D-glucosaminidase (NAG) exhibited a significant increase, but decreased on Day 2 in I/R rats. Serum creatinine (SCr) in I/R rats showed higher than sham-operated rats, but did not reach significance. In contrast, urinary vanin-1 significantly increased on Day 1 and remained significant high level on Day 2 in IR rats. Renal vanin-1 protein was decreased on Days 1 and 3. In line with these findings, immunofluorescence staining demonstrated that vanin-1 was attenuated in the renal proximal tubules in I/R rats. In vitro, the supernatant from HK-2 cells under hypoxia/ reperfusion significantly highly included vanin-1 as well as KIM-1 and NGAL. In conclusion, our results suggest that urinary vanin-1 might be a potential novel biomarker for AKI induced by I/R.

### [2P10-05]
### Comprehensive analysis of the gene expression in the lumbar spines of congenital kyphosis rats

*Noriaki Shimokawa[1], Itsuki Takahashi[1], Haku Iizuka[2] ([1]*Takasaki University of Health and Welfare*, [2]*Isesaki Municipal Hospital*)

Spinal kyphosis involves the vertebrae curving excessively backward, beyond their physiological curvature. Surgery is the only radical treatment, if the pathogenic mechanism could be analyzed in detail and a genetic diagnosis made to enable the early detection of the disease, it might prevent the disease from progressing, reduce the pain and burden on the patient, and allow surgery to be avoided. Ishibashi rats (ISRs) are characterized by kyphosis due to malformation of the lumbar spine. ISRs are generally considered useful for studying congenital malformations of the lumbar spine in humans. We adopted a DNA microarray for this purpose. For the DNA microarray analysis, total RNA was extracted from the 3rd to 5th lumbar spine segments, which are the most common areas of deformity in male ISRs, on postnatal day 4. A comprehensive analysis of the RNA/miRNA expression in ISRs was able to identify Trks, the retinol-retinoic acid metabolic pathway, and Pai-1 as deeply involved in kyphosis. Thus, a comprehensive analysis of the RNA/ miRNA expression in IS rats was able to identify several genes that are likely to be responsible for the development of kyphoscoliosis. In this presentation, we summarize the current state of kyphosis research and introduce the molecular and cellular mechanisms associated with the pathogenesis of this disease, based on findings obtained using rats that develop kyphosis. COI: The authors declare that there are no conflicts of interest.

### [2P10-06]
### Modulating astrocytes activation reorganizes noxious circuits in chronic pain

*Ikuko Takeda[1,2], Dennis L. Cheung[2], Hiroaki Wake[1,3], Junichi Nabekura[2] ([1]*Department of Anatomy and Molecular Cell Biology Graduate School of Medicine, Nagoya university,* [2]*Division of Homeostatic Development, National Institute for Physiological Sciences,* [3]*Division of Multicellular Circuit Dynamics, National Institute for Physiological Sciences*)

Current clinical management of chronic pain is suboptimal as patients often continue to experience unpleasant pain. Chronic pain's pathophysiology involves the aberrant formation of noxious "pain causing" circuits in somatosensory cortex (S1). Astrocytes proliferate throughout the central nervous system and are essential for environmental homeostasis.[WH1] More recently, additional crucial roles in controlling synaptogenesis and synaptic maintenance have been reported. Given this growing appreciation for the importance of astrocytes in facilitating neural circuit rewiring, we developed a novel approach for modulating astrocyte activation to drive circuit reorganization in chronic pain. Through transient astrocyte activation in the somatosensory cortex (S1) using tDCS (transcranial direct current stimulation) or DREADD system, established allodynia via prior partial sciatic nerve ligation was alleviated. Spine turnover of L5 pyramidal neurons in S1 cortex increased during and after tDCS and DREADD activation. This therapy fostered S1 spine elimination, presumably corresponding to the dismantling of inappropriate neural connections which induce allodynia. Thus, activated astrocytes, by facilitating S1 circuit reorganization, have the potential to cure chronic pain.

### [2P10-07]
### Molecular mechanism of reduction of cerebral edema by treadmill exercise after cerebral infarction

*Kana Sugimoto[1], Rina Gono[1], Chihpin Yang[1], Yukie Murata[1], Yohei Miyashita[1], Kazuo Harada[1], Ryuichi Katada[1], Hiroshi Matsumoto[1] ([1]*Osaka Univ.*)

Cerebral edema following cerebral infarction can be severe and directly cause death. Exercise therapy can be an effective therapy. However, the molecular mechanism remains unclear. Myokines such as IL-1RA are released during skeletal muscle contraction with effects on other organs. We hypothesized that myokine release during exercise might improve brain edema. Then we clarify the hypothesis using transient middle cerebral artery occlusion (tMCAO) model rats. Rats subjected to tMCAO were divided according to severity of illness and further assigned to exercise and non-exercise groups. Exercise group performed treadmill exercises at a speed of 2-8 m/min for 10 min at 18°C from 1 to 6 days post-reperfusion after tMCAO. The exercise significantly reduced edema and neurological deficits in severely ill rats. In those rats, AQP4 expression in the ischemic core was significantly reduced. In addition, we have shown that blood IL-1RA is reduced after ischemic stroke and that this reduction is alleviated by exercise. Administration of IL-1RA into the lateral ventricles significantly reduced edema and AQP4 expression in the ischemic core. In conclusion, treadmill exercise in the hyperacute phase decreased in blood IL-1RA following stroke and that IL-1RA administration reduced the astrocytic AQP4 expression in the ischemic core, resulting in the suppression of brain edema.

### [2P10-08]
### DNA damage by brain edema.

*Emi Nakamura-Maruyama[v], Naoyuki Himi[1], Mai Ishikawa[2], Takehiro Nakamura[1,2] ([1]*Department of Physiology2, Kawasaki Medical School,* [2]*Graduate School of Kagawa prefectural University of Health Sciences*)

Brain edema develops with various central nerve diseases. It causes increased brain volume and central nerve compression due to increased fluid volume in the brain parenchyma, which would be fatal in severe cases. Various brain disease model animals bring with brain edema, which can be cured by improving the original disease. However, the details of the brain edema itself remain unknown. The water intoxication model could be useful for investigating brain edema itself because it can induce brain edema without other brain damage. This model could be very effective in finding treatments for brain edema and biomarkers. We investigated DNA damage caused by brain edema in order to search for biomarkers of brain edema using water-intoxication model mouse. A model mouse was prepared by intraperitoneally administering sterile distilled water corresponding to 10% of the body weight of each mouse (C57BL / 6J). After 1,2,3,6 hours, the cerebrum and cerebellum were removed, and the water content and the degree of DNA damage (caspase3, 8-OHdG) were measured. We also report the results of comparative studies on age, body weight, and gender differences

### [2P10-09]
### Sulforaphane suppresses metastasis of triple-negative breast cancer cells by targeting the RAF/MEK/ERK pathway to inhibit actin stress fiber formation

*Ying Zhang[1], Qian Lu[1], Nan Li[1], Ming Xu[2], Tatsuo Miyamoto[1], Jing Liu[3] ([1]*Department of Molecular and Cellular Physiology, Yamaguchi University,* [2]*Department of Gastroenterological, Breast and Endocrine Surgery, Yamaguchi University,* [3]*Dalian Medical University*)

Breast cancer metastasis is the main cause of cancer death in women, so far, no effective treatment has inhibited breast cancer metastasis. Sulforaphane (SFN), a natural compound derived from broccoli, has shown potential health benefits in many cancers. However, research on breast cancer metastasis is still insufficient. Here, we showed that SFN, including its two isomers of R-SFN and S-SFN, significantly inhibited TGF-β1-induced migration and invasion in breast cancer cells. Proteomic and phosphoproteomic analysis showed that SFN affected the formation of cytoskeleton. Subsequent experiments confirmed that SFN significantly inhibited TGF-β1-induced actin stress fiber formation and the expression of actin stress fiber formation-associated proteins, including paxillin, IQGAP1, FAK, PAK2, and ROCK. Additionally, SFN directly bound to RAF family proteins (including ARAF, BRAF, and CRAF) and inhibited MEK and ERK phosphorylation. These *in vitro* results indicate that SFN targets the RAF/MEK/ERK signaling pathway to inhibit the formation of actin stress fibers, thereby inhibiting breast cancer cell metastasis.

### [2P10-10]
### Role of macrophages and plasminogen activator inhibitor-1 in delayed bone repair induced by glucocorticoid in mice

*Kiyotaka Okada[1,2], Naoyuki Kawao[2], Yoshitaka Horiuchi[3], Katsumi Okumoto[3], Shinji Kurashimo[3], Yoshimasa Takafuji[2], Osamu Matsuo[2], Hiroshi Kaji[2] ([1]*Department of Arts and Sciences, Kindai University Faculty of Medicine,* [2]*Department of Physiology and Regenerative Medicine, Kindai University Faculty of Medicine,* [3]*Life Science Research Institute, Kindai University of Medicine*)

Plasminogen activator inhibitor-1 (PAI-1) is the principal inhibitor of plasminogen activators and a adipocytokine that regulates metabolism. We previously reported that PAI-1 is involved in delayed bone repair induced by glucocorticoid excess in mice. However, the mechanisms by which glucocorticoids delay bone repair have not yet been clarified. We herein investigated the roles of macrophages in glucocorticoid-induced delayed bone repair after femoral bone injury using PAI-1-deficient female mice by using dexamethasone (Dex). Dex significantly decreased the number of F4/80-positive macrophages at the damaged site 2 days after femoral bone injury. Dex similarly attenuated bone injuryinduced decreases in the number of hematopoietic stem cells in bone marrow in wild-type and PAI-1-deficient mice. PAI-1 deficiency significantly attenuated Dex-induced decreases in macrophage accumulation and macrophage colony-stimulating factor (M-CSF) mRNA levels at the damaged site 2 days after bone injury. PAI-1 deficiency also significantly attenuated Dex-induced inhibition of macrophage phagocytosis at the damaged site. In conclusion, we herein demonstrated that Dex decreases the accumulation of macrophages at the damaged site during early bone repair after femoral bone injury partly through PAI-1 and M-CSF in mice

### [2P10-11]
### Dicalcin suppresses migratory activity of mouse ovarian cancer cells by downregulating Erk activation.

*Naofumi Miwa[1], Kayo Aoba[1], Ryohei Saito[2], Mayu Hanaue[1] ([1]*Department of Physiology, Saitama Medical University,* [2]*Department of Obstetrics and Gynecology, Saitama Medical University*)

Metastasis is a complex series of cellular processes including migration and local invasion in which a variety of molecules are involved in a coordinate manner. We previously identified a non-enzyme $Ca^{2+}$-binding protein (named dicalcin) as a novel suppressor of metastasis. We found that extracellularly administered dicalcin suppressed invasive activity of mouse ovarian cancer cells (OV2944) *in vitro*. A dicalcin-derived peptide (DC-p) suppressed metastasis and prolonged the survival of OV2944-bearing mice. The extracellular signal-regulated kinase 1/2 (Erk1/2) signaling pathway is one of the major signaling pathways activated during cell migration. Here, we examined the effect of DC-p administration on the activity of Erk1/2 in OV2944 cells. Our western analysis showed that OV2944 cells exhibit constitutive activation of Erk1/2, and treatment of the cells with DC-p attenuated Erk activity. The immunohistochemical study revealed Erk activation in OV2944 cells under control conditions; however, treatment with DC-p significantly reduced the level of Erk1/2 activation. To confirm the involvement of Erk1/2 in OV2944 migration, we examined the effect of inhibiting the Erk1/2 pathway with PD0325901, a MEK inhibitor, and found that treatment with PD0325901 abolished intracellular Erk1/2 activity and suppressed migration. These results indicated that dicalcin suppresses the OV2944 migration by downregulating Erk1/2 activity. There are no conflicts of interest to declare.

### Poster Presentation 2

### [2P11]
### Undergaraduate students

March 17(Thu), 12:00 - 14:00, Zoom P11

### [2P11-01]
### Usefulness of NSAIDs and their physiological mechanisms in relieving COVID-19 vaccine-induced systemic side effects

*Momono Senzaki[1], Yuika Akiyama[1], Rei Na Yeoh[1], Itsuro Kazama[1] ([1]*Miyagi University, School of Nursing*)

To help stop the pandemic of coronavirus disease 2019 (COVID-19), the vaccination is the most critical tool. However, the COVID-19 mRNA vaccines frequently cause systemic side effects shortly after the injection, such as fever, headache and generalized fatigue. Although they are usually self-limited, the use of proper medications would relieve the symptoms. In our survey, among 231 people aged 18 to 22 years who got vaccinated, less than 30% developed fever, headache and generalized fatigue after the first dose, which markedly increased up to 80% after the second dose. In most people, the symptoms after the first dose subsided spontaneously within 2 days. However, after the second dose, nearly half of the people with the symptoms required antipyretics, such as acetaminophen and non-steroidal anti-inflammatory drugs (NSAIDs; loxoprofen, aspirin or ibuprofen). Among them, the average duration of the symptoms was significantly shorter in people who took NSAIDs than those who took acetaminophen. In our patch-clamp studies, NSAIDs effectively suppressed the delayed rectifier $K^+$-channel (Kv1.3) currents in T-lymphocytes and thus exerted immunosuppressive effects. Concerning such pharmacological property, the use of NSAIDs would be more effective in relieving vaccineinduced systemic side effects that are attributable to the enhanced cellular immunity.

### [2P11-02]
### Verification of Mechanical Load on Ligament by Mathematical Model Based on Multiphoton Microscope Images

*Yuuki Naya[1,2], Hiroki Takanari[2] ([1]*Graduate School of Medical science,Program in Medical science, Immunology and Parasitology,Tokushima University,* [2]*Institute of Post-LED Photonics, Tokushima University*)

**Background:** Most studies on the mechanical response of ligaments considered them as a single uniform sheet and ignored the individual mechanical properties of their major components, collagen and elastin. We used a mathematical model based on microscopic images to examine the stress on collagen and elastin during mechanical loading.
**Methods:** Collateral ligaments were taken from slaughtered pig knees, and subjected to multiphoton microscopy with an 810 nm laser. Collagen was detected by second harmonic generation at 405 nm, and autofluorescence around 525 nm was detected as elastin. Based on acquired images, simplified mathematical models of collagen and elastin were created using a simulation software COMSOL Multiphysics to evaluate the stress on them when tensile stress was applied.
**Results:** Multiphoton microscopy revealed elastin intercalated between collagen fibers and fibrillar elastin running to connect multiple collagen fibers. Mathematical simulation showed that elastin, which intercalated between collagen or connected multiple collagen fibers, reduced the stress on collagen fibers by up to 40% compared to collagen alone.
**Conclusion:** Elastin disperses the stress applied to collagen fibers.

### [2P11-03]
### EP4 regulates cell migration through calcium signaling in oral cancer cells.

*Megumi Uchino[1], Masanari Umemura[1], Soichiro Ishikawa[1,2], Rina Nakakaji[1,2], Akane Nagasako[1], Kohei Osawa[1,2], Rafikul Islam[1], Kenji Mitsudo[2], Yoshihiro Ishikawa[1] ([1]*Cardiovascular Research Institute (CVRI), Yokohama City University Graduate School of Medicine,* [2]*Department of Oral and Maxillofacial Surgery, Yokohama City University Graduate School of Medicine*)

Introduction
Prostaglandin E2 ($PGE_2$) is an important mediator of fever, pain and inflammation. EP4 prostanoid receptors is one of the four receptor subtypes for $PGE_2$ We previously reported that EP4 formed complexes with Orai1 and increased $Ca^{2+}$ influx via Orai1, resulting in promoting cell migration. However, little information is available regarding EP4 and calcium signaling. Here, we investigated whether EP4 is related to Calcium/calmodulin-dependent protein kinase kinase(CaMKK)2/ AMP-activated protein kinase (AMPK) signaling in oral cancer cells.
Material and Methods
Human-derived tongue squamous cell carcinoma cell lines, HSC-3 were used. ONO-AE1-437 (EP4) was provided by Ono Pharmaceutical Co. In order to ablate Orai1, shRNA was induced with lentiviral infection in this cell line. Migration was examined with the scratch assay and the xCELLigence real-time cell analysis (RTCA) system. Western blotting analysis was performed to evaluate the protein expression and phosphorylation.
Results
The EP4 agonist phosphorylated CaMKK2 and AMPK in a time-dependent manner. Moreover, the EP4 agonist promoted cell migration of oral cancer in a dose-dependent manner. In contrast, the EP4 agonist did not affect cell proliferation in oral cancer cells. On the other hand, knockdown of Orai1 negated the EP4 agonist-induced phosphorylation of CaMKK2 and AMPK. Similarly, knockdown of Orai1 negated the EP4 agonist-induced cell migration.
Conclusion
Our results showed that the EP4 increased CaMKK2 and AMPK phosphorylation, resulting in promoting cell migration via Orai1.

### [2P11-04]
### Enriched environments ameliorate ADHD-like behaviors in Lister hooded rats

*Ryo Utsunomiya[1], Tomomi Doi[1], Kanta Mikami[1], Choudhury Emamussalehin[1], Hajime Yano[1], Junya Tanaka[1] ([1]*Department of Molecular and Cellular Physiology, Ehime University Graduate School of Medicine*)

Attention-deficit/hyperactivity-disorder (ADHD) is a neurodevelopmental disorder characterized with hyperactivity, inattention, and impulsivity. Although it has recently been proposed that adjustment of the nurturing environment for children is therapeutically effective for ADHD, the underlying mechanisms are still unknown. We have previously reported Lister hooded rats (LHRs) as a novel animal model for ADHD. In this study, we investigated ameliorative effects of enriched environments (EE) prepared in a large, tall cage equipped with wooden toys and houses on ADHD-like behaviors of Lister hooded rats (LHRs) that we have previously shown as a novel ADHD model animal. LHRs were reared The effect of increasing the number of animals in a single cage was also examined. LHRs began to be raised in the EE at 3 or 6 weeks of age. Behavioral tests (open field, Morris water maze, and drop tests) were done at 8 weeks of age showed that EE from both 3 and 6 weeks of age significantly ameliorated ADHD-like behaviors. Expression of mRNA for immediate early genes c-Fos, Arc, and Egr2 in the medial prefrontal cortex (mPFC) of LHRs raised in EE, compared to LHRs in the normal cages. Immunohistochemical investigation revealed that the number of neurons expressing c-Fos in the mPFC was also decreased. These results suggest that EE ameliorated ADHD-like behaviors at least partly by suppressing the neuronal activities in the mPFC.

### [2P11-05]
### The new mechanism of doxorubicin-induced heart failure via store-operated calcium entry

*Hiroko Nemoto[1,2], Masanari Umemura[1], Rina Nakakaji[1,3], Akane Nagasako[1], Kagemichi Nagao[1], Yuko Hidaka[1], Rafikul Islam[1], Soichiro Ishikawa[1,3], Yuto Mizuno[1], Megumi Uchino[1], Fumina Suzuki[1], Shinichi Suzuki[2], Yoshihiro Ishikawa[1] ([1]*Cardiovascular Research Institute, Yokohama City University Graduate School of Medicine,* [2]*Department of Surgery, Yokohama City University Graduate School of Medicine,* [3]*Department of Oral and Maxillofacial Surgery, Yokohama City University Graduate School of Medicine*)

Introduction
Doxorubicin (DOX) induces irreversible cardiotoxicity. An impairment in calcium ($Ca^{2+}$) homeostasis causes heart failure. Store-operated $Ca^{2+}$ entry (SOCE) is the mechanism by which the emptying of ER calcium stores causes influx of calcium through the plasma membrane. The most important component in SOCE is Orai1, the $Ca^{2+}$ channel. Orai1 is highly expressed in human cardiac fibroblasts (HCFs). However, it is not well known that Orai1 is related to the DOX-induced heart failure. In this study, we aimed to elucidate the relationship of them.
Materials and Methods
Western blotting was performed. Apoptosis was measured by fluorescence-activated cell sorting (FACS). Furthermore, YM-58483, SOCE inhibitor was used. Orai1-knockdown cells were also established by RNA interference.
Results
To investigate the mechanism of DOX-induced heart failure in HCFs, we focused on p53 protein. p53 is related to apoptosis in cardiac myocytes. We first performed western blotting. DOX significantly increased the protein expression of p53 in HCFs. This result is consistent with the previous reports in cardiac myocytes. We next performed FACS to evaluate apoptosis. It showed that DOX increased early apoptosis. Both YM-58483 and the knockdown of Orai1 attenuated the DOX-induced apoptosis. These results indicated that the $Ca^{2+}$ influx via Orai1 promoted the DOX-induced apoptosis.
Conclusion
These results suggest that SOCE via Orai1 may regulate the DOX-induced cardiotoxicity in HCFs. Orai1 is a new target to prevent DOX-induced heart failure.

### [2P11-06]
### Muscle contraction of the zebrafish larva lacking Nav1.4

*Chifumi Terai[1], Souhei Sakata[1], Fumihito Ono[1] ([1]*Osaka Medical and Pharmaceutical University*)

It is well known that the action potential is essential for the muscle contraction. However, it was reported that frog slow muscles and cat extraocular muscles do not have the action potential, whose neuromuscular junctions (NMJ) are distributed all over the cells. In zebrafish, fast-twitch fibers have the action potential in spite of the scattered distribution pattern of NMJs. To examine the physiological role of the action potential, we generated Nav1.4 knock-out zebrafish(NavKO). Our analysis showed: 1, Swimming capability of NavKO fish was comparable to that of wild-type fish. 2, The cytoplasmic $Ca^{2+}$ ($[Ca^{2+}]_i$) in the NavKO muscle elevated like in wild-type fish. 3, Voltage-dependent sodium current was not recorded from the muscle fibers of NavKO. 4, ($[Ca^{2+}]_i$) was elevated in the wild-type fiber by the acetylcholine stimulation even in the presence of 1 μM tetrodotoxin. 5, Membrane potential simulation showed that the attenuation of depolarization evoked at NMJ was not significant in the zebrafish larva muscle fibers because of its small size. These results indicate that the end plate potential is adequate for normal muscle contraction of zebrafish larvae.

### [2P11-07]
### The role of astrocytes in Cross-modal plasticity

*Izumo Takei[1,2], Ikuko Takeda[2], Yuki Aoyama[2], Hiroaki Wake[2] ([1]*Nagoya University School of Medicine,* [2]*Department of Anatomy and Molecular Cell Biology, Nagoya University Graduate School of Medicine*)

Cross-modal plasticity is an adaptive process that compensates for sensory deficits. The loss of sensory input to a cortical region promotes functional and structural plastic changes in that region and remained sensory regions. Ultimately this drives any residual sensory input to the region to promote the ability of residual sensory system. In this study, we investigated the role of astrocytes in cross-modal plasticity, specifically whether they are mechanistically relevant to residual sensory function improvement.
We studied cross-modal plasticity in mouse primary sensory cortex barrel field (S1BF) and secondary visual cortex (V2L) following monocular deprivation (MD) in 2-week or 5-week mice. We observed increased number of S1BF neurons and V2L astrocytes 5 days after MD in adult MD mice (5W MD), but not in congenital MD mice (2W MD). In addition, number of V2L and S1BF astrocyte contacting on neuronal soma increased in 5W MD. We further studied the underlying mechanism by measuring EdU positive astrocyte and found neuron contacting EdU positive astrocyte was increased in S1BF of 5W MD. We next studied astrocytic funtions and their contribution on neuronal circuit remodeling by in vivo $Ca^{2+}$ imaging. 5W MD astrocyte in V2L showed low synchronicity and low frequency of $Ca^{2+}$ activities compared with control mice which was not seen in 2W MD. These results suggest that cross-modal plasticity proceeds via differing mechanisms that are specific to the age of vision-loss. We are studying the effect of contacted astrocytes $Ca^{2+}$ activities on neuronal activities. The mechanisms underpinning cross-modal plasticity will be important for developing an effective approach for driving the construction of circuits which benefit the restoration of sensory deficits.

### [2P11-08]
### Inhibitory modulation of spinal activity under 5-HT administration

*Yuki Kosaka[1], Naoko Masutani[1], Chiaki Uchida-Nishiyama[1], Hirotaka Ooka[1], Akiko Arata[1] ([1]*Dept. of Physiome,Hyogo College of Medicine* )

It is known that autistic patients in childhood develop epileptic seizures, and there are also clinical reports that decrease in body movement increases the risk of autism. In addition, it has been suggested that valproic acid administration may be involved in autism as well as epilepsy. In the present study, we used spinal body movement activity as a marker to investigate the relationship between the inhibitory mechanisms of GABAA or glycine and 5- HT, which causes spontaneous activity. Recordings were taken from the C4 and L4 ventral roots of spinal cord isolated from 0.2-day-old-rat and recorded as respiratory and body movement activity respectively. First, we studied the effect of GABA or glycine on respiratory and body movement activity. Both the GABAA blocker bicuculline and the glycine blocker strychnine increased the frequency of respiratory activity, and both decreased when 5-HT was administered in the presence of inhibitory neurotransmitters (bicuculline and strychnine). However, the body movement activity did not differ significantly between bicuculline and strychnine application, but when 5-HT was applied under bicuculline, another periodic rhythm was shown in L4. This L4 rhythm was decreased amplitude during development. While, when 5-HT was perfused under strychnine, body movement activity became more regular. The spinal rhythms produced by 5-HT under bicuculline may be involved in the occasion of juvenile myoclonus epilepsy; GABA stabilized a variety of spinal body movement activities, while glycine stabilizes spinal circuits by regulating the frequency of body movement activities

### [2P11-09]
### Effects of Color-Specific Light on Schizophrenia

*Yuka Ito[1], Midori Shibushita[1], Ikuko Takeda[1,3], Ako Ikegami[1], Hiroaki Wake[1,2] ([1]*Department of Anatomy and Molecular Cell Biology, Nagoya University Graduate school of Medicine,* [2]*Division of Multicellular Circuit Dynamics, National Institute for Physiological Sciences,* [3]*Division of Homeostatic Development, National Institute for Physiological Sciences*)

The alternation of mood by light is well known. Especially, light therapy is a treatment to regulate body-mind rhythms by exposing them to sunlight or white light, which is reported to be effective in improving cognitive functions in dementia and seasonal depression. Currently,light therapy has used white light, which is a mixture of many colors. However, it has become clear that each colors have specific effect on mood and pathologies, such as green light helping to reduce pain. To clarify the connection between color specificity and behavior in a model of psychiatric disorders, we focused on red light, which is expected to cause stress and agitation, on the schizophrenia onset, in which stress factors are important for disease onset.In this study, we used a maternal immune activation (MIA) model mice as a model for schizophrenia and keptthem under blue or red light during the daytime. As a result, mice exposed to red light tended to exhibit behaviors correspond to schizophrenia, such as decreased anxiety and increased activity, compared to mice exposed to blue light. This suggests that red light may induce stress, which is one of the factors of schizophrenia, while blue light may relieve stress. Based on this finding, we will use in vivo two-photon microscopy to visualize neural activity in TLR mice kept in red light and blue light to clarify the relationship between neuronal circuitry and behavioral changes.

### Poster Presentation

### Day 3
(March 18, 12:00～14:00)

[3P01] Sensory function, Sensory organ
[3P02] Higher brain function
[3P03] Circulation
[3P04] Urinary organ, Renal function, Urination,Reproduction
[3P05] Muscle
[3P06] Muscle, Physical fitness and sportsmedicine, Oral physiology
[3P07] Physical fitness and sports medicine,Stress, Drug Action, Pharmacology
[3P08] Behabior, Biological rhythm, Sleep, Others
[3P09] Oral physiology, Study Methodology,Others
[3P10] Oral physiology
[3P01] Sensory function, Sensory organ
[3P11] Undergaraduate students
[3P12] WPI-IIIS Joint Symposium

### Poster Presentation 3

### [3P01]
### Sensory function, Sensory organ

March 18(Fri), 12:00 - 14:00, Zoom P1

### [3P01-01]
### Functional interaction between the limbic system and the frontal regions could play a significant role in olfactory recognition.

*Kei Sakikawa[1,2], Yuri Masaoka[1], Motoyasu Honma[1], Masaki Yoshida[3], Akira Yoshikawa[4], Shotaro Kamijo[1], Sawa Kamimura[1,2], Hitome Kobayashi[2], Masahiko Izumizaki[1] (*[1]Department of Physiology,Showa University School of Medicine, [2]Department of Otolaryngology,Showa University School of Medicine, [3]Department of Ophthalmology, Jikei Medical University, Tokyo, Japan, [4]Department of Physiology,Showa University School of Nursing and Rehabilitation*)

Olfactory impairment has been reported in patients with Alzheimer's disease and mild cognitive impairment. This olfactory impairment is caused by pathological changes in the amygdala (AMG) and hippocampus (HI), especially in the parahippocampal gyrus (para-HI) and entorhinal cortex (ENT), which play an important role in olfactory recognition. In this study, we measured the structural volumes and blood-oxygen-level-dependent (BOLD) signal of olfactory regions, including AMG, HI, para-HI, ENT, and orbitofrontal cortex (OFC) sub-regions, and investigated which areas of volume reductions or lower BOLD signal levels were the most associated with olfactory decline. Twenty-two subjects aged 62-84 years were tested recognition levels with T&T olfactometer, and subjects were measured whole brain T1- weighted magnetic resonance images (MRI), and functional magnetic resonance images (fMRI) during applying the odor of β-phenyl ethyl alcohol which was used in the preceding olfactory test. Stepwise multiple regression was used to assess associations between the olfactory recognition scores (independent variable) and each olfactory regions' volume and those of BOLD signals (dependent variables). The multiple regression analysis showed BOLD signal of the AMG and the medial frontal region of the OFC were associated with olfactory recognition levels. A functional interaction between the AMG and the medial frontal region of the OFC might be an important role foran olfactory ability in elderly subjects.

### [3P01-02]
### Side effects of anticancer drug on bitter and umami taste sensitivities

*Rie Fujiyama[1] (*[1]Department for Clinical Education in General Dentistry, Nagasaki University*)

Taste disorders are a common side effect of chemotherapy. Dysgeusia can lead to a patient's inability to taste food, which in turn can lead to decreased food intake and associated malnutrition. In addition, the sense of taste is very important to maintain quality of life and motivate patients to continue treatment during chemotherapy. However, the pathogenesis of chemotherapy-induced taste disorder is still unclear. In the present study, we conducted a two-bottle choice test to analyze the effects of the plant alkaloid paclitaxel on the taste sensitivities for bitter and umami tastes. We used 0.03 mM Q-HCl solution for bitter taste and 0.1 M monosodium glutamate for umami taste. Rats were used for the experiment, and all were individually reared. The rats were provided deionized water for 4 days before the two-bottle choice test. Subsequently, two bottles of bitter or umami solution and deionized water were set in each cage for 24 hours, and the amount of water consumed was measured. After one week of data collection, paclitaxel was administered intraperitoneally for 5 consecutive days per cycle, followed by a 9-day rest period, for two cycles. The control group received the same amount of saline per body weight. In the paclitaxel-treated group, a slight decrease in taste sensitivity (increase in the ratio of drinking water to bitter solution) was observed from the start of the second cycle of administration; however, this improved to the pre-administration level on days 3-4 after discontinuation of paclitaxel administration. In addition, the amount of water consumed by rats during the test for umami taste was almost the same as that of deionized water consumed before the anticancer drug was administered; however, the preference for umami taste increased after the first cycle of paclitaxel administration and remained unchanged after the withdrawal of the drug, even after the end of the second administration cycle. These results suggest that paclitaxel causes different changes in the sensitivities for bitter and umami tastes in rats. (COI: No)

### [3P01-03]
### Exploring of mouse's preferred textures using place preference tests

*Mahito Ohkuma[1], Takashi Nakano[2], Takayuki Yamashita[1] (*[1]Department of Physiology, Fujita Health University School of Medicine, [2]Department of Computational Biology, Fujita Health University School of Medicine*)

It remains poorly explored what kind of tactile stimuli mice would prefer innately. In this study, we performed place preference tests to examine this issue. First, we used a test chamber with three compartments where we pasted sandpapers with various roughnesses (P40, P400, P1000, and P2000) on the floor of one compartment. During 15 min trials, mice specifically spent more time at a compartment with P1000 over the control compartment with a smooth acrylic floor ($n = 9$ mice, $p = 0.00052$), whereas mice did not show such preference for other textures ($n = 6$ mice for each). Pre-conditioning of mice with P1000 floor did not abolish their preference for P1000. Next, we used a semirectangular tube chamber (diameter, 5 cm) with a size of ~24 cm x ~32 cm where we pasted a small piece of various fabrics made of cotton or modacrylic fur. Mice showed obvious preferences for any of these fabrics over control (no fabrics) ($n = 6$ mice, $p < 0.01$). Pre-conditioning of mice with a piece of fabric did not attenuate the preference for the fabric. In contrast, mice showed no preference for aluminum foil ($n = 3$ mice) and exhibited avoidance against a plastic board with push pins ($n = 6$ mice, $p < 0.001$). Thus, our results suggest that mice strongly prefer slightly rough and fluffy textures for a floor. We are currently searching for the brain regions responsible for such tactile preferences by cFos immunostaining and perturbational experiments.

### [3P01-04]
### Changes in nociceptor activities and dorsal horn microglia in response to persistent hindlimb immobilization in rats

*Toru Taguchi[1,2], Hiroki Ota[1,2], Haruna Takebe[1] (*[1]Department of Physical Therapy, Faculty of Rehabilitation, Niigata University of Health and Welfare, [2]Institute for Human Movement and Medical Sciences, Niigata University of Health Welfare*)

Persistent cast immobilization of a limb develops pain in response to somatic stimuli. The neural and glial mechanisms are not fully elucidated. Here we examined alterations in nociceptive afferents and dorsal horn microglia in a rat immobilization-induced pain model. Under sufficient depth of anesthesia, unilateral hindlimb was immobilized with a plaster cast. The immobilization for 4 weeks resulted in increased pain-related behaviors to mechanical and heat stimuli. Single-fiber electrophysiological recordings revealed that neither general characteristics (i.e., conduction velocity, spontaneous discharge, and distribution of the receptive field), nor the responsiveness of cutaneous C-fibers to mechanical and heat stimuli were changed in the model rats. On the other hand, the number and cell diameter of microglia significantly increased in laminae I-II of the lumbar dorsal horn in the model rats. These results suggest that activated dorsal horn microglia, rather than nociceptor-mediated peripheral mechanisms is involved in nociceptive hypersensitivity observed in rats after persistent hindlimb immobilization. This work was supported by JSPS KAKENHI (JP19H03987), and partly by the AMED Grant (JP21gm0810010h0606). There were no conflicts of interest in this study.

### [3P01-05]
### The role of appetite-stimulating signaling molecules in the olfactory cortical region in the odor-induced feeding behavior in mice

*Md Monjurul Ahasan[1], Yoshihiro Murata[1], Mutsuo Taniguchi[1], Masahiro Yamaguchi[1] (*[1]Department of Physiology, Kochi Medical School*)

Feeding is tightly coupled with olfaction, and odor-guided feeding behavior is regulated by neuromodulatory signals. The expression of feeding-related neuromodulatory signaling molecules were examined in the olfactory system including the olfactory bulb, olfactory tubercle (OT), and the other olfactory cortical area in mice, by quantitative real-time PCR. The OT was further divided into attraction-related anteromedial OT, aversion-related lateral OT and remaining central OT. Many molecules showed higher expression in the OT, especially in the anteromedial and central OT. Among the molecules examined we first chose orexin, an orexigenic neuropeptide produced in the hypothalamus, for functional analysis because its receptor is abundantly expressed in the attraction-related anteromedial OT. Suppression of orexin signals in the amOT but not in the lateral OT or nucleus accumbens by local injection of the receptor antagonist reduced attraction and conversely induced aversion to the food-associated cue odor, indicating the crucial role of appetitive signal in the amOT in the odor-guided feeding behavior.

### [3P01-06]
### Neuron-like cells expressing the neuronal-type voltage gated sodium channels are present in the chick spinal accessory lobes.

*Yumi Matsushita[1,2], Naoki Kitamura[2], Masashi Higuchi[2], Yoshinao Hosaka[2], Izumi Shibuya[2] ([1]*National Institutes for Quantum Science and Technology, Institute for Quantum Medical Science, Department of Functional Brain Imaging*, [2]*The United Graduate School of Veterinary Science, Yamaguchi University*)

In the avian spinal cord, ten pairs of protrusions called accessory lobes (ALs) are located at both lateral sides of the lumbosacral spinal cords near the dentate ligament. It is predicted that ALs act as a sensory organ of equilibrium during bipedal walking. Previously, we demonstrated that cells isolated from chick ALs showed voltage-gated $Na^+$ and $K^+$ currents and action potentials using electrophysiological method. However, it is unclear which isoform of voltage-gated sodium channel (VGSC) are expressed in ALs and whether cells having neuronal morphology in ALs express VGSCs. To clear up these points, we performed RT-PCR and immunohistochemical experiments. In ALs, the specific PCR products of $Na_v$ 1.1-1.7 were detected by RT-PCR analysis, and $Na_v$ 1.1 and 1.6 isoforms showed strong intensities among them. In immunohistochemical experiments, cytoplasm and/or cell membranes of the AL contained cells having neuron-like morphology were stained by anti-VGSC antibody and filament-like structure showed GFAP-like immunoreactivity. The VGSC- and GFAP-like immunoreactivities did not overlap. These results show that neuronal-type VGSCs are mainly expressed and that neuron-like morphology cells express VGSCs in the ALs. Our present and previous findings indicate that AL neurons generate action potentials and support the hypothesis that ALs act as organ sensing body balance during walking on the ground.

### [3P01-07]
### Vasopressin V1a receptor activation suppresses the reciprocal currents in the mouse accessory olfactory bulb partially through inhibition of high-voltage-activated $Ca^{2+}$ channels of the granule cells

*Mutsuo Taniguchi[1], Yoshihiro Murata[1], Masahiro Yamaguchi[1], Hideto Kaba[1] ([1]*Dept. Physiol., Kochi Med. School, Kochi Univ.*)

Central vasopressin (AVP) facilitates social recognition and modulates many complex social behaviors in mammals. By measuring the reciprocal synaptic currents (IPSCs) from mitral cells (MC) in the accessory olfactory bulb (AOB), the first relay in the vomeronasal system, we have demonstrated that AVP significantly reduced the IPSCs via V1a receptors. The reciprocal transmission, however, contains both glutamatergic transmission from MCs to granule cells (GCs) and GABAergic one from GCs to MCs. Thus, it is unclear whether AVP acts on the excitatory and/or the inhibitory transmissions.
In the present study, to investigate the role of V1a receptors in the GABAergic transmission, AOB slices were prepared from 23- to 35-day-old Balb/c mice. Using the whole-cell voltage clamps, the current response of GCs or MCs was recorded in the presence of antagonists for glutamatergic transmission, CNQX and AP5. Recording from GCs, an extracellular application of AVP slightly diminished the $Ca^{2+}$ currents. With analysis of its I-V relationship, the present results suggest that AVP reduces the GABAergic transmission to some extent through the inhibition of high-voltage-activated $Ca^{2+}$ channels on GCs.

### [3P01-08]
### Feasibility of infrared laser auditory prosthesis: evaluation of perception evoked by trans-tympanic laser stimulation

*Miku Uenaka[1], Yuta Tamai[1], Aya Okamoto[1], Keito Hosokawa[1], Yuki Ito[1], Tomohiro Miyasaka[1], Koji Toda[2], Shizuko Hiryu[1], Kohta Kobayasi[1] ([1]*Doshisha Univ.*, [2]*Keio Univ.*)

Infrared neural stimulation has been studied as a potential alternative to electric stimulation of a cochlear implant. No study, however, has proven that the laser stimulation evokes auditory perception. The purpose of this study was to examine laser-evoked auditory perception using classical conditioning in head-fixed Mongolian gerbils (*Meriones unguiculatus*) for demonstrateing the feasibility of laser auditory prosthesis. A click-train of 4000 Hz (sound pressure level: 70 dB SPL) was presented as a conditioned stimulus for a reward (a drop of water), and licking behavior was recorded as a conditioned response. After the training was completed, an optical fiber was inserted into the gerbil' s ear canal, and the lateral side of the cochlea was irradiated with a 4000 Hz pulsed laser from an outer ear (radiant energy: 0.2, 10.1, 19.1 $mJ/cm^2$) without the paired water. As a result, laser stimulation increased the licking rate in the same way as auditory stimulus. Furthermore, an increase in the licking rate was observed with radiant energy of laser stimuli increased, which was similar to the increase in the licking rate with the sound pressure level of auditory stimuli increased. These results indicate that the infrared laser irradiation to the cochlea can evoke auditory perception, and the perceptual level could be controlled by changing the radiant energy. This research will be an essential step for the clinical application of laser stimulation to cochlear implants.

### [3P01-09]
### The dynamics of acetylcholine in the whole cortex of the mouse performing the visual detection task.

*Akinori Y Sato[1], Ryosuke Takeuchi[1], Kei Ito[1], Masahiro Yamaguchi[1], Fumitaka Osakada[1,2,3] ([1]*Lab. of Cellular Pharmacology, Grad. Sch. of Pharmaceut. Sci., Nagoya Univ.*, [2]*Lab. Neural Info. Proc., Inst. Adv. Res., Nagoya Univ.*, [3]*NLS, Inst. Inn. Fut. Soc., Nagoya Univ.*)

Our visual perception is generated by visual information processing in the brain. The information processing in the brain dynamically changes depending on behavioral contexts and psychological states. The dynamics are regulated primarily by neuromodulators, such as acetylcholine, regulating various brain functions. However, the neural mechanism of the modulation remains unclear because little research focused on other brain regions than the primary visual cortex (Pinto et al., 2013). This study aims to examine the relationship between neuromodulation and visual perception with a focus on the brain-wide effects of acetylcholine. We monitored the distribution and concentrations of acetylcholine across the mouse brain using wide-field imaging of a genetically encoded fluorescent sensor of acetylcholine, iAChSnFR (Borden et al., 2020) . To measure the visual perception of mice, we developed a visual detection task for head-fixed mice. Fluorescence imaging of iAChSnFR during the task demonstrated that the response was modulated depending on whether the stimulus was presented on the left or right side and whether mice licked the spout to report the stimulus detection. We are currently comparing the performance of the task with the acetylcholine dynamics and neural activity.

### [3P01-10]
### Olfactory learning-dependent plasticity of neuronal connection from piriform cortex to olfactory tubercle in mice

*MD. Fazley Rabbi Sha[1], Yuriko Koga[1], Yoshihiro Murata[1], Mutsuo Taniguchi[1], Masahiro Yamaguchi[1] ([1]*Kochi Medical School,Kochi University*)

Previous study suggests that the olfactory tubercle (OT), which belongs to the olfactory cortex and ventral striatum, has functional domains that represent odor-guided motivated behaviors. Learning of odor-guided attractive and aversive behavior activates anteromedial (am) and lateral (l) domain of olfactory tubercle (OT), respectively. However, the mechanism of learning-dependent activation of specific OT domains remains unknown. We hypothesized that neuronal connectivity of OT domains plastically changes through olfactory experience. To examine the plastic potential of synaptic connections to OT domains, inputs from the piriform cortex to OT were optogenetically stimulated in mice in association with food reward for attractive learning and with electrical foot shock for aversive learning. The size of photo-activated axon boutons preferentially increased in amOT compared to lOT for attractive behavior learned mice and increased in lOT compared to amOT for aversive behavior learned mice. These results indicate the learningdependent plasticity of synaptic connection to OT domains. Authors have no COI with regard to the presentation.

### [3P01-11]
### The afferent origin of the globus pallidus receiving auditory information

*Yasuharu Hirai[1], Fumino Fujiyama[1] ([1]*Laboratory of Histology and Cytology, Faculty of Medicine, Hokkaido University*)

The globus pallidus externalis (simply the globus pallidus in rodents; GP) serves as the inhibitory center of the basal ganglia nuclei, which distributes their axons to whole basal ganglia structures. In the classical basal ganglia scheme, GP neurons only relay the striatum outputs to their innervation targets with receiving the inhibition from the GABAergic medium spiny neurons of the striatum. However, a number of research successively indicated that the GP anatomy and its functional role are more complicated and significant than the old "relay" conception. Previously, we demonstrated the excitatory acoustic responses of the GP neurons located the caudolateral part of the GP. To explore the candidates conveying the auditory information to the GP especially with excitation, we injected retrograde tracers into the auditory input recipient region of the GP. The results indicated that many cortical inputs were originated not only from auditory related area but also limbic area including insular and parahippocampal cortices. In addition, retrogradely labeled cells were found in posterior paralaminar thalamic nuclei. Further studies based on this anatomical finding might elucidate the role of the GP neurons for sensory processing.

### Poster Presentation 3

### [3P02]
### Higher brain function

March 18(Fri), 12:00 - 14:00, Zoom P2

### [3P02-01]
### Temporal order judgment task between basic tastes: an initial study

*Makoto Wada[1], Kouji Takano[1], Tatsu Kobayakawa[2] ([1]*Research Institute of NRCD*, [2]*National Institute of Advanced Industrial Science and Technology*)

Latency of gustatory evoked magnetic fields (GEMs) of salty tastant occurs faster than those of sweet tastant by approximately 100 ms (Kobayakawa et al., 1999). This would be caused by differences in receptor's type (i.e., ionotropic or metabotropic). In this study, we aimed to clarify whether human participants could percept the delay by testing temporal order judgment between salty and sweet. Participants (n = 3) were required to contact tips of their tongues to a small hole in a Tygon tube that purified water was running. Tastants (Salty: 0.5M NaCl, Sweet: 1M Sucrose) sectioned with air bubbles were sequentially delivered to the participants, and then the participants were required to reproduce the orders of them by pressing buttons. The participants correctly judged the orders in most of the trials (91.7 %; 55 of 60 trials). When mixture of the tastants was delivered, the participants tended to judge that the salty tastant came first (83.3 %; 25 of 30 trials). This result suggests the participants could percept the delay due to the receptor's type. In future studies, we will investigate relationships between the temporal characters of taste perception and unbalanced diets.

### [3P02-02]
### The active spots movement of responses to the FM sounds were dependent upon sound intensity in AI of guinea pigs observed by optical recording.

*Yutaka Hosokawa[1] ([1]*Dept. of Systems Physiol., Grad. Sch. Univ. of Ryukyus*)

The influence of sound presure on the active spots movement of responses to the FM sounds in the primary auditory cortex (AI) and DC field of the guinea pig were investigated using optical imaging with a voltage-sensitive dye (RH795). Eight guinea pigs were anesthetized with ketamine (80 mg/kg) and xylazine (40 mg/kg). Activity patterns to the FM sounds (the linear sweep: the start and end frequency, 16 and 0.5 kHz in 16-64 ms duration) and tones (0.5-16 kHz, 200 ms duration) at 55-85 dB SPL were recorded from the AI and DC field on both sides. When the sound pressure level is high, the active-spots to the downward FM sounds were appeared at the 16kHz-frequency band (FB) in the AI and DC field and along the 16kHz-FB and spread to lower-FB. On the lower sound pressure, the active-spots were also appeared and spread along 16kHz-FB but disappeared. Then, the active-spots to the real frequency of FM sound were appeared and moved to the lower-FB. These active-spots movements were observed in the middle area of the AI. These results show that the responses to the downward FM were calicultted in AI and represented the active-spots on the middle of AI.

### [3P02-03]
### Integration of temporal information on auditory and visual stimuli in monkey prefrontal neurons

*Atsushi Chiba[1], Kazunori Morita[2], Ken-ichi Oshio[1], Masahiko Inase[1] ([1]*Dept Physiol., Faculty Med., Kindai Univ.*, [2]*Dept Physiol., Sch Med., Iwate Med Univ.*)

To clarify neuronal processing involved in the integration of auditory and visual signals for time perception, neuronal activity was examined in prefrontal cortex (PFC) of macaque monkeys during a duration discrimination task with auditory and visual cues. In the task two cues were consecutively presented for different duration between 0.2 and 1.8 sec. Each cue was either auditory or visual, and followed by a delay period. After the second delay period subjects indicated which cue, the first or second cue, was presented longer. PFC neurons responded during the cue and delay periods. Cue-responsive neurons mostly responded to either auditory or visual cue, and to either the first or second cue. The first delay-responsive neurons showed activity that changed depending on the first cue duration and were mostly sensitive to cue modality. The second delay-responsive neurons exhibited activity that represented which cue, the first or second cue, was presented longer. Nearly half of this activity representing order-based duration was sensitive to cue modality. These results suggest that PFC was likely to process temporal information with visual and auditory signals separately in the early stage of duration discrimination and integrate the information in the later stagefor the final decision.

### [3P02-04]
### Temporal perception bias in Japanese monkeys similar to human subjects: Behavioral model analysis.

*Kei Mochizuki[1], Akira Murata[2], Masahiko Inase[2] ([1]*Dept Physiology, Sch Medicine, Iwate Medical Univ*, [2]*Dept Physiology, Faculty Medicine, Kindai Univ*)

Human subjects possess a cognitive bias to underrate temporal intervals between one's action and its consequence. To investigate this phenomenon in animal experiments, we established a behavioral task for macaque monkeys. The monkey judged the temporal interval between a pair of tones as short or long, compared with the predetermined criterion. To evaluate the influence of animal's motor intention on temporal perception, the tones were either contingent on monkey's voluntary button press or presented just passively.

For each experimental day, we randomly used different set of intervals and a criterion. However, the monkey performed the task well by learning temporary criterion. Using computational models, we evaluate this learning process by formulating it as successive updates of internal criterion for short/long judgments. In the models, the criterion was updated based either solely on objective task conditions, or also based on the difference of voluntary/passive presentation. Of those, latter models suited better to the monkey's behavior. This implies a shared cognitive bias in temporal perception between human and non-human primates, and opened future animal experiments.

### [3P02-05]
### Roles of basolateral nucleus of the amygdala in episode-processing by hippocampal CA1

*Junko Ishikawa[1], Dai Mitsushima[1] (*1. Yamaguchi University Graduate School of Medicine; Yamaguchi,*)

Episodic memory contains "what-where-when" information of personal experience. There are innumerable episodes, but how the brain processes each episode remains unknown. We found episode-specific synchronized activities by multiple neurons in the hippocampal CA1, which were characterized by super burst, ripple-firings, and no-firing silent period. We also found that generation of these synchronized activities were prominent after strong-emotional episodes. In the present study, we investigated the influence of basolateral nucleus of the amygdala (BLA) during experiencing episodes on memory formation and synchronized activities my multiple CA1 neurons. Inactivation of BLA weakened memory formation for negative-emotional episode (restraint stress), whereas positive-emotional episode (contact with the opposite sex) or other episodes (contact with the same sex or novel object) were not influenced by BLA inactivation. Generation of super burst and ripple-firings induced by restraint stress were also partially blocked by BLA inactivation. These observations indicate that processing for negative-emotional episode by CA1 involves BLA neural activity, and the brain mechanisms of episode processing were experience type specific.

### [3P02-06]
### Encoding of episodic-experiences by ripple-firings in the hippocampal CA1

*Satoshi Kunimoto[1], Junko Ishikawa[1], Dai Mitsushima[1] ([1] *Yamaguchi university*)

Ripple-firings are thought to be important for spatial learning and memory formation, however, it is still unclear how they are involved in their function. In our previous study, we found that episodic experiences change the features of ripple-firings, which are composed of multiple-unit activity in the CA1, and furthermore, the changes were different in an episode-specific manner. These observations indicate that ripple-firings encode episode information. In the present study, we calculated Euclidean Distance with Dynamic Time Warping to investigated the similarity of the ripple-firings converted to a 0/1 signal generated before and after episodic experiences. We found that many of ripple-firings occurred before episodic experiences were similar to each other, whereas a novel ripple-firings appeared after episode that differ from any other ripple-firings after episode. Our results indicate that the deep leaning of ripple-firings generated a novel ripple-firings after episode may enable us to decipher the encrypted brain code for episodic memory.

### [3P02-07]
### Distinct roles of ventral medial frontal and dorsolateral prefrontal cortex in regulating social behavior in monkeys

*Sachihiro Shirahama[1], Hitoshi Nagano[1], Shinya Nakamura[1], Ken-ichiro Tsutsui[1] ([1]*Laboratory of Systems Neuroscience, Graduate School of Life Sciences, Tohoku University*)

Macaque monkeys live in flocks, which are known to be based on complex relationships between their members, and in particular, male monkeys form strict hierarchical relationships. It is well known that monkeys can change their behaviors depending on whether the opponent's position is superior or inferior to them, but the neural background of such social behavior remains unveiled. In this study, we focused on the ventral medial frontal cortex (vMFC) and the dorsolateral prefrontal cortex (dlPFC) as brain areas that may be related to such social behaviors. We used three Japanese monkeys, which were not of equal social status, and the second monkey from the top was used as the stimulation target. We put two of the three monkeys facing each other and let them compete to pick up pieces of sweet potato, each of which was baited in the individual wells of a board between them (competitive food-picking task). We applied 1 or 10 Hz rTMS, which are known to have an inhibitory or facilitatory effect, respectively, bilaterally to the vMFC or dlPFC of the monkey, and evaluated the change of its task performance. The inhibitory (1Hz) rTMS to vMFC and the excitatory (10Hz) rTMS to dlPFC changed the social attitude of the stimulated monkey to be more self-effacing and assertive, respectively. Neither the excitatory rTMS to vMFC nor inhibitory rTMS to dlPFC affected the behavior. These results suggest distinct roles of vMFC and dlPFC in regulating social behavior.

### [3P02-08]
### The area of mouse brain damaged by depression studied using Mn MRI method

*Akio Inoue[1], Yuriko Inoue[2], Hiromitsu Ezure[2], Naruhito Otsuka[2], Chika Sawa[2], Koichi Shiraishi[3], Yoshinobu Manome[4] ([1]*Human Brain Research Center, Graduate School of Medicine, Kyoto University*, [2]*Dep. Anat., Showa Univ. Sch. Med.,*, [3]*Dev. Med. Eng. Jikei Uni. Med.*, [4]*Div. Mol. Cell Biol. Res. Cent. for Med. Sci., Jikei Uni. Med.,*)

Mn ions enter into nerve cells through membrane Ca-channel, depending on nerve activity, and Mn ions induce the increase of T1 signal of MRI. So, Mn-MRI was used to measure the nerve activity in vivo. However, Mn ions do not stay in nerve cells stably for long time. We found that Mn ions inside the cells are released and disappeared by nerve activation. This study suggests that mouse should be activated only when Mn ions were charged. When Mn ions were injected into *abdominal cavity, Mn ions enter into blood vessel and finally Mn ions enter into brain through* chorioid plexus. *Mn concentration in the brain is maintained for few hours. Therefore, we applied restraint stress for three hours. Next day, we measured T1 signal of mouse brain* using Bruker 9.4T MRI machine. We found that mouse brain is strongly activated by restraint stress. Furthermore, we found that restraint stress dependent staining of brain by Mn ions was disappeared when the second application of restraint stress was applied. When restraint stress for three hours was repeated for three days, mouse becomes depression. We applied Mn ions at final restraint stress. After they become depression we applied restraint stress to release Mn ions in the brain. The area where the activity of brain was damaged by depression was not erased by restraint stress. Using this method we found that several place of brain was damaged when they become depression.

### [3P02-09]
### Evaluation method of VR immersion using event-related potential

*Kaito Kageyama[1], Ryo Ogawa[1], Yasushi Nakatani[2], Yumie Ono[3], Shingo Murakami[1] ([1]*Department of Electrical, Electronic, and Communication ,Faculty of Science and Engineering ,Chuo University*, [2]*Faculty of Economics, Chuo University*, [3]*Department of Electronics and Bioinformatics, School of Science and Technology, Meiji University*)

The sense of immersion is one of the major features of virtual reality (VR) and improving the sense of immersion is essential for producing high-quality VR content, although the immersiveness has been evaluated subjectively. To provide objective criteria for VR content, we constructed an EEG-based method to evaluate the sense of immersion quantitatively. Ten healthy adult males participated in this study and watched 6-minute VR movies with a 3D image presented on the HMD, with a 2D image and a still image presented on the LCD. EEG signals were recorded during the viewing and after viewing the images, the subjects were asked to answer a questionnaire for subjective evaluation. An oddball task was used to measure P300 of event-related potential ERP and to evaluate the immersiveness with the mental workload for the task. In this study, a total of 230 stimuli were presented to subjects at 1000 ms intervals in random order: standard stimuli of 70% of the total stimuli at 1800 Hz, target stimuli of 15% at 2000 Hz, deviant stimuli of 15% at 500 Hz. The results were measured and analyzed from three perspectives: subjective, behavioral, and physiological. For the subjective measure, we analyzed the results of the subjective questionnaire, for the behavioral measure, we analyzed the response to the oddball task, and for the physiological perspective measure, we analyzed the measured P300 waveforms. We confirmed the different degrees of immersion from the subjective questionnaire, and we showed that the amplitude of P300 in response to deviant stimuli during the oddball task is effective in quantifying the degree of immersion.

### [3P02-10]
### Different timing of behavioral event representation in the hippocampus and entorhinal cortex

*Shogo Soma[1,2], Shinya Ohara[3,4], Satoshi Nonomura[2,5], Junichi Yoshida[2,6], Naofumi Suematsu[7], Yutaka Sakai[2], Ken-Ichiro Tsutsui[3], Yoshikazu Isomura[2,5] ([1]*Department of Molecular Cell Physiology, Kyoto Prefectural University of Medicine*, [2]*Brain Science Institute, Tamagawa University*, [3]*Laboratory of Systems Neuroscience, Tohoku University Graduate School of Life Sciences*, [4]*PRESTO, JST Sakigake*, [5]*Department of Physiology and Cell Biology, Graduate School of Medical and Dental Sciences, Tokyo Medical and Dental University*, [6]*Dominick P. Purpura Department of Neuroscience, Albert Einstein College of Medicine*, [7]*Department of Bioengineering, University of Pittsburgh*)

The entorhinal cortex is the main interface between the hippocampus and the neocortex, and plays a critical role in learning and memory. This hippocampal-entorhinal circuit has been anatomically investigated in detail. EC neurons in the superficial layers innervate the hippocampus: EC layer II and III neurons send their projections to hippocampal DG/CA3 and CA1, respectively. Since CA1 neurons are innervated by CA3 terminals, signals from the EC would be directly or indirectly integrated/put together within CA1 neurons. In turn CA1 neurons directly or indirectly (via subiculum) send back the signals to the deep layers of EC. Thus, anatomical evidence showing the existence of the hippocampal-entorhinal circuit has been accumulated. However, the temporal dynamics of this circuit are still unknown. To this end, we trained rats on the simplest operant task without instructive cues under a head-fixed condition. We recorded extracellularly CA1 and LEC neurons from the pre- and post-trained rats in this task and examined the relationship between behavioral events and spiking activities on a subsecond scale.

### [3P02-11]
### Simultaneous in vivo recording of the local field potential and the signal of fluorescent calcium indicator in the hippocampus of Altzheimer's mouse model.

*Munenori Ono[1], Harunori Ohmori[1], Shinji Muramoto[1], Sachiko Yamaki[1], Feng Xu[1], Yoshie Horie[1], Ianlan Ma[1], Nobuo Kato[1] ([1]*Kanazawa Medical University*)

It is well known that Alzheimer disease (AD) patients have severe learning disabilities. In the early stage of AD, several studies have suggested that soluble amyloid beta induced the disorder in the neural activity and the intracellular calcium dynamics, which are very likely to be relevant to the learning disability as well as the progression of cell death in AD. However, the neurophysiological disorder in early stage of AD has not been fully elucidated. In this study, to examine the disorder in the neural activity and calcium dynamics in vivo, we utilized a photometric patch electrode (PME, Hirai et al., 2015), with which simultaneous recording of fluorescence and electrical signals were available. Using a PME, we recorded the local field potential (LFP), spike activities, and the signals of fluorescent calcium indicator in the hippocampus, where is closely related to learning ability and spatial cognition. The recordings were performed in dorsal hippocampal CA1 region of model mice of AD (3xTg), with which spatial cognitive defects were confirmed in the early stage of AD. During the PME recording, the mouse was placed on a treadmill to monitor the movement, and head-fixed via a metal rod. The spatial learning ability of the individual animal was assessed by Morris Water Maze (MWM) test in advance of the PME recording. In both WT and AD model mice, the increase of Ca signal was observed in dorsal hippocampus CA1 when the animal moved. During locomotion theta oscillation was observed in LFP in both animals, however the frequency was lower in AD model than WT. In addition the signals accompanied with the movements, the transient rises of the calcium signal were also seen sporadically while the animal was stationary. During the rise of the Ca signal, high frequency oscillation was observed in LFP. In some AD model animals, the power of high frequency oscillation during the calcium increase decreased, and its degree was correlated with MWM score.

### Poster Presentation 3

### [3P03]
### Circulation

March 18(Fri), 12:00 - 14:00, Zoom P3

### [3P03-01]
### Systems analysis on the effects of vericiguat on the baroreflex-mediated sympathetic arterial pressure regulation

*Toru Kawada[1], Meihua Li[1], Shohei Yokota[1], Midori Kakuuchi[1], Keita Saku[1] ([1] *Department of Cardiovascular Dynamics, National Cerebral and Cardiovascular Center*)

**Background:** Vericiguat is a stimulator of soluble guanylate cyclase (sGC) and increases cyclic guanosine monophosphate (cGMP) in target cells. **Aim:** We examined the effects of vericiguat on baroreflex-mediated sympathetic arterial pressure (AP) regulation. **Methods:** Carotid sinus baroreceptor regions were isolated from the systemic circulation in anesthetized rats (n = 6). The carotid sinus pressure (CSP) was changed stepwise from 60 to 180 mmHg to delineate the baroreflex-mediated sympathetic AP regulation over a wide input range. The sympathetic nerve activity (SNA) and AP responses were examined before and during the administration of vericiguat at 10 $\mu$g/kg/min. **Results:** Vericiguat significantly reduced the response range (81.0 ± 6.9 vs. 39.1 ± 3.0 mmHg, $P < 0.01$) and the lower asymptote (70.7 ± 4.0 vs. 61.0 ± 2.1 mmHg, $P < 0.01$) of the relationship between CSP and AP, whereas it did not significantly affect the response range (80.5 ± 4.9% vs. 77.0 ± 3.1%) or the lower asymptote (22.7 ± 5.7% vs. 23.7 ± 8.7%) of the relationship between CSP and SNA. **Conclusion:** Vericiguat reduces AP mainly through its peripheral vasodilatory effect without significantly affecting SNA.

### [3P03-02]
### A competitive permeation of $Ca^{2+}$ and $Na^+$ in $Ca_V1.3$ L-type calcium channels

*Futoshi Toyoda[1], Akinori Noma[2], Yukiko Himeno[2], Wei-Guang Ding[1], Mariko Omatsu-Kanbe[1], Hiroshi Matsuura[1] ([1] *Department of Physiology, Shiga University of Medical Science,* [2] *Department of Bioinformatics, College of Life Sciences, Ritsumeikan University,*)

It is generally believed that L-type $Ca^{2+}$ channels are highly $Ca^{2+}$ selective and hardly permeable to $Na^+$ under physiological condition. Nevertheless, we have recently identified $Ca_V1.3$ L-type $Ca^{2+}$ channel as a molecular determinant for the sustained inward $Na^+$ current ($I_{st}$) that is a key player in cardiac pacemaker activity. Here, we report experimental and theoretical realization of the competitive permeation of $Ca^{2+}$ and $Na^+$ through $Ca_V1.3$ channels. In patch-clamp experiments, $Ca_V1.3$ typically evoked L-type $Ca^{2+}$ current ($I_{Ca,L}$) in the presence of external $Ca^{2+}$ at 1.8 mM, which was gradually decreased as the $[Ca^{2+}]_o^+$ was lowered but bottomed out at around 0.1 mM, suggesting a switch of conducting ion from $Ca^{2+}$ to $Na^+$. Impressively, a large $Na^+$flux was suddenly relieved from $Ca^{2+}$ block when the $[Ca^{2+}]_o^+$ was reduced to submicromolar levels. Theoretical analysis using a classical permeation model (Almers & McCleskey, *J Physiol,* 1984) well explained the experimental observation of the anomalous mole-fraction dependence of $I_{Ca,L}$ through $Ca_V1.3$ channels, but predicted the presence of two permeation modes with distinct $Ca^{2+}$ sensitivity. The $Ca^{2+}$ block kinetics will be investigated for each mode in single channel recordings and computer simulation.

### [3P03-03]
### Comparison of $Ca^{2+}$ dynamics in excitation-contraction coupling of quail and rat isolated cardiomyocytes

*Ogura Yuhei[1], Ito Hiroaki[1], Sugita Shukei[1], Nakamura Masanori[1], Ujihara Yoshihiro[1] ([1] *Nagoya Institute of Technology* )

Mammals and birds both have fast heart rates. Mammalian cardiomyocytes have T-tubule membranes, which are effective in rapidly changing $Ca^{2+}$ concentrations. In contrast, bird cardiomyocytes do not have T-tubule membranes. In this study, we compared the contraction-relaxation behavior and $Ca^{2+}$ transient of cardiomyocytes isolated from adult quails and rats to investigate the mechanism of fast heart rate without the T-tubule membrane in birds. Isolated cardiomyocytes from quails were significantly narrower than those from rats. Although the contraction and relaxation times of quails tended to be longer than those of rats, the differences were not large. When $Ca^{2+}$ transients in the entire cardiomyocyte were measured using Fura-2 AM, the time to peak tended to be longer in quails than in rats. On the other hand, the decay time was markedly shorter in quails than in rats. As a result, the total time from the beginning of the $Ca^{2+}$ increase to the return to the base $Ca^{2+}$ concentration was shorter in quails than in rats. These results suggest that quails may achieve fast heart rates by enhancing $Ca^{2+}$ removal ability.

### [3P03-04]
### Transporter-dependent regulation of myocardial interstitial serotonin levels in the rat heart

*Takashi Sonobe[1], Tsuyoshi Akiyama[1], James Pearson[1] ([1] *Department of Cardiac Physiology, National Cerebral and Cardiovascular Center Research Institute*)

The roles of serotonin (5-HT) transporters, serotonin transporter (SERT) and plasma membrane monoamine transporter (PMAT) in the regulation of myocardial interstitial 5-HT levels remains unclear. To investigate the roles of two transporters in 5-HT uptake and its metabolism in the heart, we monitored myocardial interstitial levels of 5-HT and 5-HIAA, a major metabolite of 5-HT by monoamine oxidase (MAO), in anesthetized rats using microdialysis technique. Fluoxetine (Flu, an inhibitor of SERT), decynium-22 (D22, an inhibitor of PMAT), or their mixture (Flu + D22) was locally administered by reversemicrodialysis. At 60 min after administration of 5-HT transport inhibitors, pargyline (a MAO inhibitor) was co-administered to examine 5-HT metabolism by MAO. Flu rapidly increased dialysate 5-HT concentration, while D22 gradually increased dialysate 5-HT concentration. Flu + D22 induced a larger increase in dialysate 5-HT concentration compared to Flu or D22 alone. Flu increased dialysate 5-HIAA concentration, and this increase was abolished by pargyline. D22 and Flu + D22 did not change dialysate 5-HIAA concentration, which were not affected by pargyline. Both SERT and PMAT regulate myocardial interstitial 5-HT levels by uptake, however 5-HT uptake via SERT contributes less to 5-HT metabolism by MAO.

### [3P03-05]
### Cerebrovascular function in several models of pregnancy induced hypertension in rats

*Hirotsugu Tsuchimochi[1], Hisashi Maeda[1], James Pearson[1] ([1] *National Cerebral and Cardiovascular Center*)

Pregnancy induced hypertension (PIH) is a disease characterized by hypertension and proteinuria during pregnancy, usually occurring in late pregnancy and worsening over time. The pathogenesis of PIH is primarily based on vascular endothelial dysfunction. We hypothesized that a history of PIH would be a risk factor for future cerebrovascular disease, and therefore compared cerebrovascular function in postpartum rats in several models of PIH. As models of PIH, we used the continuous angiotensin or vasopressin administration model, the chronic hypoxia exposure model, and the Dahl-Iwai saltsensitive rat model. Although varying levels, all models of PIH showed an increase in urinary microalbumin, indicating possible renal dysfunction. The autoregulatory ability of cerebral blood flow in response to changes in blood pressure or in $PaCO_2$ measured under isoflurane anesthesia were different among PIH model animals, normal delivery, and non-pregnant animals. It remains to be investigated how long the vascular endothelial dysfunction lasts and whether it is related to the risk of stroke.

### [3P03-06]

### Synchronous spontaneous $Ca^{2+}$ transients in mucosal capillary pericytes in rectum of NG2-GCaMP mouse.

*Retsu Mitsui[1], Hikaru Hashitani[1] ([1]*Nagoya City University*)

**Aims:** Capillary pericytes play a fundamental role in maintaining mucosal blood flow in the intestine. Here we examined $Ca^{2+}$ dynamics in mouse intestinal capillary pericytes. **Methods:** Intracellular $Ca^{2+}$ imaging of NG2-expressing capillary pericytes was conducted using mucosa/submucosa preparations of NG2-GCaMP6 mouse rectum. **Results:** Rectal capillary pericytes exhibited synchronous spontaneous $Ca^{2+}$ transients. Carbenoxolone (3 μM; gap junction blocker), CPA (10 μM; endoplasmic reticulum $Ca^{2+}$-ATPase inhibitor), 2- APB (100 μM; $IP_3$ receptor inhibitor) or the $Ca^{2+}$-activated Cl- channel (CaCC) blocker niflumic acid (100 μM) suppressed $Ca^{2+}$ transients and also disrupted their intercellular synchrony. Nifedipine (1 μM) induced heterogenous suppression of the $Ca^{2+}$ transients amongst pericytes without affecting their synchrony. Immunostaining showed that pericytes had bipolar long processes on the capillary endothelium. **Conclusion:** Capillary pericytes in the rectal mucosa develop synchronous spontaneous $Ca^{2+}$ transients primarily arising from endoplasmic reticulum $Ca^{2+}$ release. Subsequent CaCC-dependent depolarisation in pericytes would effectively spread to their neighbours via gap junctions to maintain their intercellular synchrony.

### [3P03-07]

### The existence of the left ventricular viscosity apparently decreases the left ventricular end-systolic elastance in the failed hear

*Shuji Shimizu[1], Yasuhiro Kotani[2], Toshiaki Shishido[1] ([1]*National Cerebral and Cardiovascular Center*, [2]*Okayama University*)

A time-varying elastance model of cardiac chambers is often used for computational hemodynamic simulation. However, every substance has viscosity as well as elasticity. In this study, we developed a computational cardiovascular model combined with a time-varying visco-elastance model of cardiac chambers and a modified 3-element Windkessel vasculature model to examine the effect of the left ventricular viscosity on the hemodynamics of a failed heart. The time-varying visco-elastance model of the left ventricle was made based on the Kelvin-Voigt viscoelasticity model. The existence of the left ventricular viscosity required higher stressed blood volume (SBV) and increased the ventricular-arterial coupling (VAC). Compared with the normal heart with Ees of 3.0 mmHg/ml (SBV: +3.0%, VAC: +2.3%), the existence of the left ventricular viscosity largely affected SBV and VAC in the failed heart with Ees of 1.5 mmHg (SBV: +7.0%, VAC: +5.1%). The existence of the left ventricular viscosity apparently decreased the left ventricular end-systolic elastance and the impacts of viscosity were larger in the failed heart.

### [3P03-08]

### Mechanisms of Automaticity and Early Afterdepolarization in HL-1 Mouse Atrial Myocytes

*Yuhichi Kuda[1], Yasutaka Kurata[1], Takayuki Ikeda[2], Mamoru Tanida[1], Kunichika Tsumoto[1], Toshishige Shibamoto[1], Hideto Yonekura[2]
([1]*Department of Physiology II, Kanazawa Medical University*, [2]*Department of Biochemistry II, Kanazawa Medical University*)

**Aim:** HL-1 mouse atrial myocytes, often used for systematic electrophysiological studies, exhibit automaticity and are useful for investigating ionic and dynamical mechanisms of pacemaker activity. In this study, we determined the characteristics of $I_{K1}$ channel currents and their involvement with automaticity in HL-1 cells using the superforated-patch technique (Shioya, 2020), which is much more efficient than the conventional perforated-patch technique. **Methods:** The superforated-patch recording using nystatin (0.3mg/mL) were performed to determine $I_{K1}$ dynamics and the effects of an $I_{K1}$ blocker, $Ba^{2+}$, and caffeine that depletes $Ca^{2+}$ in the SR on action potentials (APs) or automaticity. We also tested whether an $I_{Kr}$ inhibitor, E-4031, could induce early afterdepolarization (EAD) in HL-1 cells. **Results:** 1) $I_{K1}$ dynamics determined by the superforated-patch recording were essentially the same as those determined by the conventional whole-cell patch clamp. 2) $I_{K1}$ block by $Ba^{2+}$ at 2 mM induced automaticity. 3) Caffeine attenuated intracellular $Ca^{2+}$ transients, slowed pacemaking, and abolished automaticity. 4) Co-administration of E-4031 (5 mM) and isoproterenol (10 mM) induced EADs in both stimulus-evoked and spontaneous APs. **Conclusions:** The superforated-patch technique enabled more efficient recordings than the conventional methods. HL-1 cells showed two types of pacemaker activity, i.e., $I_{K1}$ block-induced and SR $Ca^{2+}$ release-dependent pacemaking. EAD could be induced by the combination of $I_{Kr}$ block and β-adrenergic stimulation in HL-1 cells. Thus, HL-1 cells are useful for systematically investigating the mechanisms of cardiac automaticity and EAD formation.

### [3P03-09]

### Effects of restrain stress and exercise on brain-bone marrow interactions

*Thu Van Nguyen[1,4], Ko Yamanaka[1], Keisuke Tomita[1], Jasenka Zubcevic[2], Sabine Gouraud[3], Hidefumi Waki[1] ([1]*Graduate school of Health and Sports Science, Juntendo University*, [2]*Department of Physiology and Pharmacology, University of Toledo*, [3]*College of Liberal Arts, International Christian University*, [4]*Department of Military Occupational Medicine, Vietnam Military Medical University* )

**Background.** Recent studies have reported that chronic stress (CS) induces brain inflammation through activation of bone marrow (BM)-derived microglia (BM-Mic). We hypothesized that CS induces hypertension via inflammatory responses (IFRs) trigered by migrated BM-Mic in the paraventricular nucleus of hypothalamus (PVN) and daily exercise prevents IFRs in the PVN. **Method.** Wistar rats were devided into 3 groups: CS, CS+daily voluntary exercise, and control. CS was produced by immobilization (1 hour/day, 5 days/ week, 3 weeks). Gene expression (GE) of inflammatory factors (IFs) in the BM and PVN, population of blood inflammatory cells (BICs), and BM-Mic in PVN were investigated. **Result.** CS induced IFRs represented by altered GE profiles in the BM and population of BICs of CS group. Moreover, the number of BM-Mic in the PVN increased in CS group compared to control group. Exercise reduced this number and suppressed GE of cell migration factors in PVN, although it did not improve the stress-induced IFRs of BM and blood. **Conclusions.** These results suggest that CS induces IFRs by BM-Mic migration in the PVN, whereas exercise prevents these responses by alteration of GE profiles of hypothalamus. Further studies require to understand whether these events contribute to blood pressure levels.

### [3P03-10]

### Elucidation of mammalian heart evolution by multiscale analysis of the lobster heart

*Akira Hanashima[1], Misaki Kimoto[1], Kai Ihara[1], Momoko Ohira[1], Yuu Usui[1], Ken Hashimoto[1], Satoshi Mohri[1] ([1]*First Department of Physiology, Kawasaki Medical school*)

The blood filling system by the atria to the ventricles is common in vertebrates, but the arthropod heart has only ventricles. In this study, we integrally analyzed the lobster heart from the molecular to the organ level, and discussed the differences from the evolutionary strategy of the vertebrate heart. The movement of the ventricular wall was observed by echocardiography, and the ventricles collapsed when the shell was opened, suggesting that the blood inflow into the ventricles was due to the elastic tissue attached to the ventricles. The systolic ventricular pressure was about 10 mmHg. Histological staining showed that, as in amphibians, there were no coronary vessels and the myocardial tissue was coarse, and electron microscopic images showed that the sarcomere length was similar to that of mammals, but the myosin filaments were low in density. These differences may be due to the fact that lobsters have an open vasculature and do not require a large ventricular contractile force. In addition, the connectin molecules that determine ventricular extensibility was localized in the I-band of the sarcomere, and the spring region was as short as in mammals. These results suggest that arthropods that did not choose a system in which blood is pumped passively by the atria did not need to extend their ventricles easily, and the spring region of the connectin was consistently short throughout evolution. The atria, which were important in the amphibian era, are becoming unnecessary as the ventricles acquire the ability to draw blood, and the spring region of the connectin may have been shortened through a different history than in mammals.

### Poster Presentation 3

### [3P04]
### Urinary organ, Renal function, Urination, Reproduction

March 18(Fri), 12:00 - 14:00, Zoom P4

### [3P04-01]
### Severe hypertension with aging causes detrusor underactivity-like bladder dysfunction in rats

*Shogo Shimizu[1], Yoshiki Nagao[2], Atsushi Kurabayashi[3], Takahiro Shimizu[1], Youichirou Higashi[1], Takaaki Aratake[1,4], Suo Zou[1], Motoaki Saito[1] ([1]*Department of Pharmacology, Kochi Medical School, Kochi University,* [2]*Department of Pediatrics, Kochi Medical School, Kochi University,* [3]*Department of Pathology, Kochi Medical School, Kochi University,* [4]*JSPS Research Fellow* )

**Purpose:** Detrusor underactivity (DU) is a contraction of reduced strength and/or duration, resulting in a failure to achieve complete bladder emptying within a normal time. Aging is known as an obvious risk factor for DU. However, the pathophysiological mechanism is not well clarified. High blood pressure causes various bladder dysfunctions. We investigated the effect of aging on bladder functions in spontaneously hypertensive rat (SHR).
**Materials and Methods:** Male SHRs at the age of 18, 36, 54 or 72 weeks (wk) were used. Wistar Kyoto rats (WKYs) were used as normotensive control.
**Results:** SHRs at 72 wk showed significant increases in mean blood pressure, bladder weight/body weight ratio (BBR), urine volume, single voided volume, post voiding residual urine volume (PVR), bladder capacity (BC), intercontraction interval (ICI), and decreases in bladder blood flow (BBF) and voiding efficiency (VE) compared to SHRs at 18 wk. However, there were no significant differences on mean blood pressure, BBR, BBF or voiding parameters in WKYs among each age.
**Conclusion:** Aged SHRs demonstrated DU-like bladder dysfunction (increases in PVR, BC and ICI, and a decrease in VE). Severe hypertension with aging can cause DU-like bladder dysfunction via polyuria and bladder ischemia.
**Coflict of Interest (COI):** Authors have no COI to disclose.

### [3P04-02]
### Effects of intermittent cold stimulation of the skin on voiding efficiency in anesthetized rats

*Kaori Iimura[1], Nobuhiro Watanabe[1], Harumi Hotta[1] ([1]*Department of Autonomic Neuroscience, Tokyo Metropolitan Institute of Gerontology*)

[Aim] The aim of this study is to clarify whether cold stimulation of the skin increases voiding efficiency.
[Methods] In anesthetized female rats, saline was infused to the emptied bladder via a catheter until the spontaneous voiding was induced. The voiding efficiency was calculated from the voided volume and bladder capacity (infusion volume). Intermittent cold stimulation (25℃/ every 20 s) was applied to the lower back and rump skin during saline infusion to the bladder. We compared voiding efficiency without and with cold stimulation.
[Results] Voiding efficiency during cold stimulation was 10~15% higher than that without cold stimulation. When the skin area for stimulation was pre-treated with local anesthetic lidocaine, the increase in voiding efficiency during cold stimulation was not induced. During voiding, the maximum bladder micturition pressure was unchanged, whereas the urethral relaxation time was prolonged by cold stimulation.
[Conclusion] Our results suggest that the excitation of the skin afferent nerve induced by intermittent cold stimulation of the lower back and rump skin promotes relaxation of the urethra reflexively, thereby increasing voiding efficiency.

### [3P04-03]
### Action of baclofen on micturition and parasymapathetic preganglionic neurons in the sacral spinal cord

*Yoshitaka Nakamura[1], Ayumi Nakamura[1], Keisuke Koga[1], Hidemasa Furue[1] ([1]*Dept Neurophysiol, Hyogo College of Medicine*)

Coordination between urine storage and voiding functions is precisely mediated by a complex neuronal control system in the central nervous system (CNS) including afferent and efferent pathways. However, the detail central neuronal control mechanisms remain unclear. In this study, I investigated effects of baclofen, a GABAB receptor agonist, on neuronal activities in the superficial dorsal horn (SDH) and sacral parasympathetic nucleus (SPN) in the spinal cord at the single-cell and synaptic levels. Simultaneous recordings of intravesical pressure (IVP) and spinal activity in the SPN enabled us to show detailed actions of baclofen on the voiding functions. SNP episodic firings followed by IVP increases, and baclofen inhibited the number of action potentials in SPN neurons. In some occasions, it increased the frequency of the IVP with small voiding volumes and shorter oscillatory period at the peak of IVP increase.

### [3P04-04]
### Suppression of PAN-induced nephropathy in rats by novel selective TRPC3/6 channel blocker

*Reiko Sakaguchi[1], Ryo Okada[1,2], Yoshimune Matsuda[1,3], Marie Konomi[1,3], Ryu Nagata[4], Masayuki Mori[1] ([1]*Bio-materials and Chemistry, School of Medicine, University of Occupational and Environmental Health,* [2]*Human, Information and Life Sciences, School of Health Sciences, University of Occupational and Environmental Health,* [3]*Faculty of Environmental Engineering, The University of Kitakyushu,* [4]*Graduate School of Pharmaceutical Sciences, Osaka University*)

Nephrotic syndrome is a dysfunction of kidney that is diagnosed by high urinary protein and low serum albumin caused by the defect of podocytes in glomerulus. It has been reported that Transient Receptor Potential Canonical 6 (TRPC6) mutations found in patients with focal segmental glomerulosclerosis (FSGS), a steroid-resistant nephrotic syndrome with no effective medication, often cause hyperactivated channel currents. Regarding this mechanism, we have proposed that the disruption of $Ca^{2+}$-dependent inactivation in TRPC6 channel led to prolonged cation influx and disorganized cytoskeleton in the podocytes.
Here, we developed "L862", a novel selective TRPC3/6 blocker. This compound has a superior pharmacokinetic property compared to previously reported drugs, which allows it to be administered orally. We investigated the effect of L862 in normal and puromycin aminonucleoside (PAN)-induced nephrotic rats. This blocker exerted significant improvement of proteinuria in PAN-induced nephrotic rats, while no apparent toxicities were observed in normal rats. These results suggest that L862 would be a promising therapeutic compound for channel-related diseases such as steroid-resistant nephrotic syndrome.

### [3P04-05]
### Functional analysis of $Ca^{2+}$-dependent inactivation in TRPC6 channel may predict the risk and the age at onset of Nephrotic syndrome

*Tatsuya Komaki[1,2], Ryo Okada[2,3], Reiko Sakaguchi[2], Takanori Kihara[1], Masayuki Mori[2] ([1]*Department of Life and Environment Engineering, Faculty of Environmental Engineering, The University of Kitakyushu,* [2]*Bio-materials and Chemistry, School of Medicine, UOEH, Japan,* [3]*Human, information and Life Sciences, School of Health Sciences, Occupational and Environmental Health*)

Transient Receptor Potential Canonical 6 (TRPC6) gene encodes a non-selective cation channel and expresses in the various types of cells including podocytes in kidney glomerulus. TRPC6 gene mutations are found in the two major causes of nephrotic syndrome (NS), namely minimal change and focal segmental glomerular sclerosis (FSGS). We have previously reported that a negative feedback regulation triggered by cellular calcium elevation, $Ca^{2+}$-dependent inactivation (CDI), is impaired in NS-associated TRPC6 mutations. However, the pathophysiological significance of this impaired CDI is largely unknown. Here, we analyzed the degree of CDI reported in NS-associated TRPC6 mutations within the N-terminal ankyrin repeat or coiled-coil domains and its relationship with the development of NS. The patch-clamp recording confirmed that the inactivation of TRPC6 channel currents was significantly delayed for the cells expressing NS-associated channels compared to that of wild-type. Furthermore, TRPC6 mutations associated with the early-onset NS (such as G109D, R175W, R895L) and the late-onset along with poor prognosis (P112Q, K874X) exhibited increased integration of the current density compared to that of mutations associated with the late-onset NS (G109S, R175Q). These findings provide that evaluation of the CDI in TRPC6 may contribute to pathological prediction to be the development of NS as well as its age at onset and prognosis.

### [3P04-06]
### Integration of Barrington's nucleus input and visceral afferent input in the lumbosacral pregagnlionic neurons

*Masahiro Kawatani[1,2,3,4], William Chet de Groat[5], Keiichi Itoi[6], Katsuya Uchida[6], Kenji Sakimura[7], Akihiro Yamanaka[1,2], Takayuki Yamashita[4], Masahito Kawatani[3] ([1] *Nagoya University, RIEM,* [2] *Nagoya University, Graduate School of Medicine,* [3] *Akita University, Graduate School of Medicine,* [4] *Fujita Health University, School of Medicine,* [5] *University of Pittsburgh,* [6] *Tohoku University, Graduate School of Medicine,* [7] *Niigata University, Brain Research Institute*)

Barrington's nucleus (Bar), which controls micturition behavior through downstream projections to the spinal cord, contains two types of projection neurons, $Bar^{CRH}$ and $Bar^{ESR1}$, that have different functions and target different spinal circuitry. Both types of neurons project to the L6-S1 spinal intermediolateral (IML) nucleus, whereas $Bar^{ESR1}$ neurons also project to the dorsal commissural nucleus (DCN). We used patch-clamp recording in spinal slices from adult mice in combination with optogenetic stimulation of Bar terminals. Recording of opto-evoked excitatory postsynaptic currents (oEPSCs) in 1,10-dilinoleyl-3,3,30,30-etramethylindocarbocyanine, 4- chlorobenzenesulfonate (DiI)-labeled lumbosacral preganglionic neurons (LS-PGNs) revealed that both Bar neuronal populations make strong glutamatergic monosynaptic connections with LSPGNs, whereas $Bar^{ESR1}$ neurons also elicited smaller-amplitude glutamatergic polysynaptic oEPSCs or polysynaptic opto-evoked inhibitory postsynaptic currents (oIPSCs) in some LS-PGNs. Optical stimulation of $Bar^{CRH}$ and $Bar^{ESR1}$ terminals also elicited monosynaptic oEPSCs and polysynaptic oIPSCs in sacral DCN neurons, some of which must include interneurons projecting to either the IML or ventral horn. Application of capsaicin increased opto-evoked firing during repetitive stimulation of Bar terminals through the modulation of spontaneous postsynaptic currents in LS-PGNs. In conclusion, our experiments have provided insights into the synaptic mechanisms underlying the integration of inputs from Bar to autonomic circuitry in the lumbosacral spinal cord that may control micturition.

### [3P04-07]
### Physiological function of CDKAL1 in kidney

*Hiroko Nagata[1], Yu Nagayoshi[1], Takeshi Chujo[1], Hitoshi Nakazato[2], Kazumoto Tomizawa[1] ([1]*Department of Molecular Physiology, Faculty of Life Sciences, Kumamoto University,* [2]*Department of Pediatric Health Education, Faculty of Life Sciences, Kumamoto University,* )

A genome-wide association study reported that Cdk5 regulatory associated protein1-like1 (CDKAL1) as a causative gene of type 2 diabetes. We have reported that CDKAL1 is an RNA modification enzyme that thiomethylates $tRNA^{Lys}$ (UUU) at position 37. Moreover, the mutation of CDKAL1 gene causes impairment of insulin processing and reduction of insulin secretion in pancreatic β cells. In addition, the mutation of CDKAL1 gene has reported one of the risk factors for progression of chronic kidney disease (CKD). We found that Cdkal1 localizes in mouse glomerulus using immunostaining by anti-Cdkal1 antibody. These reports and results suggest that the CDKAL1 gene may have the important role in kidney function. However, the physiological role of CDKAL1 in kidney is almost unclear.
We generated Cdkal1 knockout mice and examined the urine biochemistry test. As a result, the concentration of albumin is significantly elevated in urine of Cdkal1 KO adult mice. However, the urine of pancreatic β-cell-specific Cdkal1 knockout mice did not show the phenotypes of albuminuria. Urine albumin is a biomarker of podocyte damage. Thus, these results suggest that kidney Cdkal1 is important for podocyte function, anti-proteinuria effect, through efficacy of Lysine translation.

### [3P04-08]
### Mechanisms of excretion of bacterial-specific modified nucleosides in urine and its physiological significance

*Kayo Nishiguchi[1], Yu Nagayoshi[1], Ryosuke Yamamura[1], Takeshi Chujo[1], Kazuhito Tomizawa[1] ([1]*Department of Molecular Physiology Kumamoto University Faculty of Life Sciences*)

RNAs are essential molecules for protein synthesis. Recently, various post-transcriptional chemical modifications are reported in RNA especially, transfer RNA. Interestingly, the types of these chemical modifications are different between mammals and bacteria. We have found that modified nucleosides, the metabolites of modified RNA, are not reused in cytosol and excreted to extracellular spaces, especially urine. From these backgrounds, we make the hypothesis that bacterial-specific modified nucleotides are excreted into urine of patients with bacterial infection. First, we analyzed bacteria-specific modified nucleotides by mass spectrometry using extracted RNA from 12 bacterial strains including gram-positive and negative bacteria. We found that 2-methyladenosine ($m^2A$) was detected from all strains. Next, we performed co-culture experiment with bacteria and macrophages, and $m^2A$ was detected from phagosomes of macrophages.
Finally, we analyzed modified nucleotides in urine samples from patients with bacterial or viral infections. As a result, urinary $m^2A$ was significantly elevated in patients with bacterial infections. These results suggest that $m^2A$ could be a novel diagnostic marker for bacterial infections.

### [3P04-09]
### Alteration of the excretion of modified nucleosides in the urine of renal disease model mice

*Tomoya Sakamoto[1], Yu Nagayoshi[1], Hitomi Kaneko[1], Takeshi Chujo[1], Kazuhito Tomizawa[1] ([1]*Department of Molecular Physiology, Faculty of Life Sciences, Kumamoto University*)

tRNA is an important molecule that plays a central role in protein translation. Recently, there are various chemical modifications on these tRNAs, and we have reported that defects in these modifications cause various diseases. Our previous studies have shown that modified RNAs are finally degraded to single modified nucleosides and excreted into urine.However, the influence of kidney diseases against excretion are almost unknown. We collected urine samples from db/db mice, which are representative mouse model of chronic kidney disease. We examined the concentrations of modified nucleosides in the urine using LC-MS.As a result, five types of modified nucleosides were elevated in the urine of db/db mice.
Next, we examined the effect of losartan potassium, which is a representative angiotensin receptor blocker (ARB) and has the beneficial effect on renal disease on the excretion of the elevated modified nucleosides in the urine of db/db mice. As a result, the drug decreased the excretion of two kinds of the modified nucleosides. The two modified nucleosides were correlated with urinary albumin concentration. These results may suggest that the two modified nucleosides represent the reabsorptive function of tubules in kidney and the remaining three modified nucleosides depend on the glomerulus damage.

### [3P04-10]
### Suppressive effect of dicalcin on *in vitro* trophoblast attachment using human cell lines.

Ryohei Saito[1], *Hiromasa Satoh[2], Kayo Aoba[2], Hajime Hirasawa[2], Naofumi Miwa[2] ([1] *Dept. Obstet. Gynecol., Saitama Med. Univ.,* [2] *Dept. Physiol., Saitama Med. Univ.*)

Implantation is a critical process for the normal development of the embryo, beginning with the close interaction between a competent blastocyst and a receptive uterus. To contribute to the study of implantation, we investigated the action of dicalcin on the attachment of human choriocarcinoma cells (BeWo cells) on a monolayer of human endometrial carcinoma cells (Ishikawa cells). Extracellularly administered dicalcin binds to the cell surface of BeWo cells. Pretreatment of BeWo spheroids with dicalcin inhibited the attachment of BeWo spheroids onto Ishikawa monolayer. We identified the partial amino acid sequence of human dicalcin that exhibited maximum suppression for BeWo spheroid attachment. Transmission electron microscopy analysis revealed that the dicalcin-derived peptide caused a widening of the intracellular junction between BeWo and Ishikawa cells. Our results demonstrated that dicalcin suppresses *in vitro* attachment of BeWo spheroids onto Ishikawa monolayer and suggested that dicalcin may be a novel suppressor of attachment between the embryo and the maternal uterus at implantation. There are no COIs to declare.

### [3P04-11]
### Selection of frozen mouse early embryos by membrane potential measurement

*Masao Miyake[1], Susumu Yoshie[1], Satoru Kaneko[2], Akihiro Hazama[1] ([1]*Department of Cellular and Integrative Physiology, Fukushima Medical University School of Medicine,* [2]*Ichikawa General Hospital, Tokyo Dental College*)

Morphological inspection is the most commonly used technique to pick quality oocytes and embryos for artificial fertilization. To raise reproductive ratio, a new selection method from a new point of view is needed. The membrane potential reflects expression of ion channels and completeness of cell membrane, it may evaluate ovum quality. We previously showed that there was a wide dispersion of membrane potential among eggs without morphological difference. It implied this technique could be applied for quality selection. In this study, we analyzed the relationships between embryogenic outcome and membrane potential of mouse embryos after freeze-thaw cycle. Single-cell, two-cell and four-cell embryos were applied to the freeze-thaw cycle, and measured membrane potential. Some embryos performed good morphological characteristics, and could reach blastocysts. But most embryos which performed near zero voltage stopped development. The near zero voltage embryos are possible to be scratched during conventional protocol. This method may be applicable to ignore damaged embryos. All experiments were planned toward institutional guidelines and reviewed by institutional animal care and use committee.(COI:NO)

### Poster Presentation 3

### [3P05]
### Muscle

March 18(Fri), 12:00 - 14:00, Zoom P5

### [3P05-01]
### Chronic kidney disease induces impaired muscle fatigue resistance with different metabolic changes between fast- and slow-twitch skeletal muscles.

*Hiroyori Fusagawa[1,2], Tatsuya Sato[1], Takashi Yamada[3], Yuki Ashida[3], Atsushi Teramoto[2], Ogon Izaya[1], Toshihiko Yamashita[2], Noritsugu Tohse[1] (*[1]Department of Cellular Physiology and Signal Transduction, Sapporo Medical University School of Medicine, [2]Department of Orthopedic Surgery, Sapporo Medical UnivSapporo Medical University School of Medicine, [3]Graduate School of Health Sciences, Sapporo Medical University*)

**Background:** It is known that impaired muscle fatigue resistance in chronic kidney disease (CKD)-related cachexia increases risks of reduced activity of daily living and increased mortality. Although energy metabolism plays an important role in muscle endurance, physiological characteristics of muscle fatigue resistance and changes in energy metabolism for each fast- and slow-twitch muscle in CKD-related cachexia remain unclear. **Methods and Results:** CKD was induced by 5/6 nephrectomy (Nx) in male Wistar rats, and sham-operated rats served as controls (Sham). In *situ* torque measurement in plantar flexors using in *vivo* electrical stimulation revealed that the number of 70 Hz-repeated tetani until peak torque was decreased to 50% of the starting value and was significantly reduced by 15% in Nx compared with Sham. Ex *vivo* relative force analysis assessed by a percent reduction from the initial force during electrical fatigue stimulations revealed that a significant force reduction in Nx compared with that in Sham occurred in 40 seconds in a fast-twitch extensor digitorum longus (EDL) muscle and in 120 seconds in a slow-twitch soleus muscle (SOL). Measurement using an extracellular flux analyzer revealed that state 3 mitochondrial respiration was significantly decreased in isolated mitochondria from the SOL but not in isolated mitochondria from a fast-twitch plantaris muscle (PLA). Metabolome analysis in the PLA revealed that the intermediate metabolites of the TCA cycle were reduced in Nx compared with that in Sham at the point of fatigue, when the tetanus compared with the resting state was 50% of the initial tension, suggesting that substrate availability in the TCA cycle is impaired in Nx compared with that in Sham in response to fatigue induction in a fast-twitch muscle. **Conclusions:** The results suggest that different metabolic changes between fast- and slow-twitch muscles underlie impaired muscle fatigue resistance in CKD-related cachexia.

### [3P05-02]
### Mechanisms of slowing effect of innosine triphosphate on skinned smooth muscle relaxation

*Masaru Watanabe[1], Yasuyuki Naraki[1] (*[1]Graduate School, Tokyo Metropolitan University*)

Inosine triphosphate (ITP) is known to induce force development independent on myosin light chain phosphorylation in skinned smooth muscle. We previously reported that nucleoside triphosphate other than ATP, including ITP, slowed relaxation process by $Ca^{2+}$ removal after $Ca^{2+}$-induced force development in skinned taenia cecum. To elucidate the mechanisms of slowing effects of inosine triphosphate on skinned smooth muscle relaxation, we examined the relaxation process under the inhibition of various kinase/phosphatase activities, since ITP is a poor substrate for kinase/phosphatase reactions, but acts as a substrate for myosin ATPase activity. Inhibition of activity of myosin light chain kinase, creatine kinase, AMP kinase of myosin phosphatase 2A did not affect the slowing effects of ITP on relaxation process. A kinetic analysis indicated that ITP slowed the translation rate of fast detaching cross-bridge to formation of slow cycling (latch like) bridge. These results suggest that ITP accelerates formation of slow cycling bridge by direct changes in ATPase cycling.

### [3P05-03]
### Effects of cytochalasin D on actin-myosin bind or dissociation on relaxation process of skinned smooth muscle

*Satoko Mihashi[1], Masaru Watanabe[1] (*[1]Tokyo Metropolitan University*)

Cytochalasin D is known to inhibit actin polymerization and also to suppress smooth muscle contraction. To clarify mechanism of cytochalasin D induced acceleration of skinned (cell membrane permeabilized) smooth muscle relaxation, the relaxation process of skinned smooth muscle both in the absence or presence of cytochalasin D was analyzed based on our kinetic model (Mihashi et al., 2020). Cytochalacin D significantly enhanced force decay during relaxation induced by Ca ion removal after the maximal Ca ion induced contraction during relaxation both in skinned taenia cecum and carotid artery. The data fitting analysis of the relaxation process indicates that cytochalacin D accelerates slow (latch-like) bridge dissociation. On the other hand, in the absence of nucleoside triphosphate, cytochalasin D affected any kinetic parameters neither in carotid artery or taenia cecum. During the relaxation process, cytochalsin D inhibits actin polymerization, which promotes slow cycling (latch-like) bridge dissociation and accelerates relaxation, however in absence of nucleoside triphosphate, actin is tightly bound to myosin and cytochalasin D has little effect.

### [3P05-04]
### Strategy of extraocular muscle to achieve super-fast shortening velocity: an x-ray diffraction study.

*Maki Yamaguchi[1], Toru Kurihara[2], naoya nakahara[1], Hideki Yamauchi[1], Kazuhiro Hirano[1], Mai Yamaguchi[1], Tetsuo Ohno[3], Toshiko Yamazawa[1], Shigeru Takemori[1] (*[1]The Jikei University School of Medicine, [2]Nihon Med Univ, [3]Teikyo Heisei Univ*)

Saccadic eye movements to catch the object image in one's sight are achieved by extraocular muscle (EOM) which expresses super-fast type myosin heavy chain and contracts with the highest shortening velocity in the body. To elucidate structural bases to achieve the fastest shortening velocity, we have performed x-ray diffraction study carried out in High Energy Research Institute (KEK). Diffraction patterns obtained from skinned fibers of EOM showed no sampling peaks at 0.05 $nm^{-1}$ on the first myosin layer line, which is well distinguished in the diffraction patterns of fast leg muscle fibers that express ordinary fast type myosin heavy chain. This indicated that a larger population of myosin heads of super-fast EOM had higher mobility compared with the fast leg muscle. Next, we obtained diffraction patterns in the presence of BDM, which is known to shift myosin heads from a mobile intermediate to a stable one. EOM showed no sampling peaks on the first myosin layer line at 0.05 $nm^{-1}$ even in the presence of BDM. However, there appeared sampling peaks at 0.04 $nm^{-1}$, which is reported to be observed in slow leg muscle. This suggested a possibility that myosin heads of EOM would achieve the fastest shortening velocity with a relatively low free-energy cost in the myofilament lattice partially adopting the strategy of slow leg muscle.

### [3P05-05]
### Slow muscle-type nicotinic acetylcholine receptor in zebrafish shows high $Ca^{2+}$ permeability.

*Buntaro Zempo[1], Fumihito Ono[2], Koichi Nakajo[1] (*[1]Jichi medical university, [2]Osaka Medical and Pharmaceutical University*)

Skeletal muscle in vertebrates consists of two types of muscle fibers: slow muscle and fast muscle. A recent study in zebrafish found that the subunit composition of nicotinic acetylcholine receptor (AChR) in neuromuscular junction of slow muscle is different from fast muscle. In zebrafish as well as in mammals, AChR in fast muscle is composed of $\alpha$, $\beta$, $\delta$, and $\varepsilon$ (or $\gamma$) subunits. On the other hand, AChR in the slow muscle of zebrafish lacks $\varepsilon$ (or $\gamma$) and is composed of only $\alpha$, $\beta$ and $\delta$ subunits. However, the physiological significance of slow muscle-type AChR has not been understood. In the present study, we compared the channel properties of slow and fast muscle-type AChRs expressed in *Xenopus* oocytes by two-electrode voltage clamp. We found that the slow muscle-type AChR shows much higher $Ca^{2+}$ permeability than the fast muscle-type one. To clarify the physiological functions of the $Ca^{2+}$ influx through the slow muscle-type AChR, we mutated Glu (E) of channel pore in $\delta$ subunit, which is considered to be a key amino acid residue for $Ca^{2+}$ permeability, to Gln (Q). We confirmed that the AChR containing the mutant $\delta$ subunit lost the $Ca^{2+}$ permeability in *Xenopus* oocytes. Then we generated a transgenic zebrafish line that expresses the mutant $\delta$ subunit in skeletal muscle to analyze the physiological roles of the $Ca^{2+}$ influx through slow muscle-type AChR on locomotor activity.

### [3P05-06]
### Regional differences in effect of acupuncture stimulation on excitability of spinal motor neurons

*Akira Nihonmatsu[1] ([1]*Hokkaido College of Oriental Medicine*)

In this study, we examined the effect of acupuncture stimulation on electromyogram F wave to determine the effect of acupuncture stimulation of various areas (Hand, Face, Leg) on excitability of spinal motor neuron. Ten healthy right-handed adults participated in this study. The experiments were performed under four conditions ; acupuncture stimulation of right LI4, acupuncture stimulation of right ST4, acupuncture stimulation of right ST36, cold noxious stimulation. An acupuncture needle (40mm long 0.18mm in diameter) was inserted a depth of 10mm at the right LI4, ST4, ST36. F wave was induced in the ulnar nerve by supramaximal stimulus eliciting M wave, and was recorded from the right first dorsal interosseous muscle. F wave were measured before and after stimulation. We analyzed the amplitude ratio of F wave / M wave. Acupuncture stimulation to the LI4 or ST36 was significantly increased Amplitude ratio of F/M, decreased in ST4. Furthermore, cold noxious stimulation was significantly increased Amplitude ratio of F/M. A positive correlation was observed between changes in the amplitude ratio of F/M induced by acupuncture stimulation and changes in the amplitude ratio of F/M induced by cold noxious stimulation. Acupuncture stimulation to the LI4 or ST36 increased excitability of spinal motor neuron, decreased in ST4. Thus, application of acupuncture to the extremity region increases the excitability of spinal motor neuron, acupuncture to the facial region decreases the excitability of spinal motor neuron.

### [3P05-07]
### Effects of EMD57033, an activator of the actomyosin ATPase activity, on the relaxation process of cell membrane permeabilized carotid artery and taenia cecum from guinea pig

*Yasuyuki Naraki[1], Masaru Watanabe[1] ([1]*Tokyo Metropolitan University*)

EMD57033, an activator of the actomyosin ATPase activity, is known to bind to an allosteric pocket in the myosin motor domain and to increase contractile force in cardiac muscle. However, the effect of EMD57033 on the smooth muscle contraction and relaxation process is not still unclear. In order to clarify the regulatory mechanism of EMD57033 on smooth muscle in detail, we examined the effects of EMD57033 on relaxation process by $Ca^{2+}$ removal after $Ca^{2+}$- induced contraction of β-escin skinned (cell membrane permeabilized) carotid artery and taenia cecum preparations from guinea pigs. EMD57033 at 30 μM and 100 μM suppressed the force decay during relaxation both in skinned carotid artery and taenia cecum. In the rigor solution (without ATP), EMD57033 suppressed the force decay during relaxation both in skinned carotid artery and taenia cecum, but the inhibitory effect of EMD57033 in carotid artery was higher than taenia cecum. These results suggest that EMD57033 suppressed the relaxation process with and without ATP because it affects to conformation of the myosin and increases directly actin affinity to bind to an allosteric pocket in the smooth muscle domain.

### [3P05-08]
### Effect of beta-adrenergic receptor and CGRP receptor on expression of myosin heavy chain class II (MyHCII) mRNA in mouse skeletal myocytes

*Junko Yamaji[1], Reiko Hiroshima[2], Yoshiaki Mori[2] ([1]*Dept. of Nutr. Sci., Kansai Univ. of Welf. Sci.*, [2]*Dept. of Rehab., Kansai Univ. of Welf. Sci.*)

Neurotransmitter receptors including beta-2 adrenergic receptor and calcitonin gene related peptide (CGRP) receptor on skeletal muscle cells receive neurotransmitters from motor nerves at neuromuscular junctions and also stimulates adenylate cyclase and the cAMP pathways in skeletal muscle cells. However, the effect of these receptors and the cAMP-PKA pathway on mRNA expression of MyHC and myokine including iinterleukin (IL) -6 in skeletal muscle remains unclear. In the present study, we examined that beta-2 agonists and CGRP on mRNA expression of MyHCIIb and myokine in murine skeltal muscle cells. Then our results are as follows: (1) The IL-6 mRNA level was not also affected by medium supplemented with forskolin, with beta-2 agonists and with PKA inhibitor. (2) The $MyHCII_b$ mRNA level was significantly increased by IL-6 induced by calcineurin activatiors and was significantly attenuated by calcineurin inhibitor. (3) The $MyHCII_b$ mRNA level was not affected by medium supplemented with forskolin, with PKA inhibitor and with beta-2 agonists. These results expected that production of IL-6 by calcineurin activation upregulated $MyHCII_b$ mRNA but that but that PKA-dependent cAMP pathway is not affected for IL-6 mRNA level and $MyHCII_b$ in C2C12 cells.

### [3P05-09]
### Effect of 17β-estradiol on sepsis-induced muscle weakness and atrophy in mice

*Shunya Takeshita[1], Kazuho Sakamoto[1], Chihiro Hibi1, Honoka Yamashita[1], Junko Kurokawa[1] ([1]*Dept. Bio-Info. Pharmocol., Univ. Shizuoka*)

Infectious diseases such as sepsis induce myopathy characterized by reductions in muscle force-generation and mass leading to severe difficulties not only in activities of daily living but also in respiration. Since males are more prone to sepsis, we investigated the effect of 17β-estradiol (E2), a major ingredient of estrogen, on septic symptoms in skeletal muscle in an *in vivo* and *in vitro*. E2 treatment attenuated cecal ligation puncture (CLP)-induced loss of grip strength in ovariectomized (OVX) mice and preserve contractility of extensor digitorum longus muscle from OVX mice underwent CLP. Furthermore, E2 significantly attenuated lipopolysaccharide (LPS)-induced atrophy of C2C12 myotubes. Our quantitative PCR analysis suggested that E2 attenuated LPS-induced induction of TNFα, an inflammatory cytokine mRNA but not muscle specific ubiquitin ligase atrogin-1 in myotubes. Furthermore, E2 fail to attenuate the LPS-induced increase of LC3II/I ratio, a marker of autophagy. Our results suggested that E2 attenuates inflammatory responses in myotubes which may be also involved in muscle protection from sepsis-induced muscle weakness.

### [3P05-10]
### EMD57033 enhance $Ca^{2+}$ -induded contraction of skinned smooth muscle.

*Yuka Suzuki[1], Watanabe Masaru[1] ([1]*Tokyo Metroplitan*)

Background
EMD57033, a myosin activator, is known to increase $Ca^{2+}$ sensitivity for the force in cardiac muscle. To investigate the EMD57033 action on smooth muscle contractility, we examined the agent effect on $Ca^{2+}$ -induced contraction of βescin skinned smooth muscle.
Materials and methods
A small stirp of taenia cecum from guinea pig (0.8-1.0 mm long and 0.2 mm wide) was attached to a force transduce apparatus. The preparation was skinned (cell membrane permeabilized with 200 μM βescin and 10 μA23187). The preparation was activated with 10-5.7 to 10-5.0 M $Ca^{2+}$ with 1 μM calmodulin in the absence or presence of EMD57033. The develop force of $Ca^{2+}$-induced contraction was normalized with that of the control contraction induced with 10-5 M $Ca^{2+}$ and 1 μM calmodulin.
Results
EMD57033 significantly enhanced the force of $Ca^{2+}$-induced contraction when $Ca^{2+}$ concentration was 3 μM and higher. However, EMD57033 had little effects on 2 μM $Ca^{2+}$- induced the submaximal contractile force.
Discussion
The present results indicate that EMD57033 enhances $Ca^{2+}$ induced force development by direct changes in myosin structure and/or myosin ATPase activity rather than increase in myosin light chain phosphorylation.

### [3P05-11]
### Participation of cAMP/PKA-mediated signaling pathways in upregulation of MyHC I mRNA in C2C12 skeletal muscle cells.

*Yoshiaki Mori[1], Junko Yamaji[2], Reiko Hiroshima[1], Manabu Miyamoto[1] ([1]*Dept of Rehabil Sci, Kansai Univ of Welf Sci*, [2]*Dept of Nutr Sci, Kansai Univ of Welf Sci*)

Our previous study using C2C12 cells indicated that calcineurin activation upregulates myosin heavy chain type I (MyHC I) mRNA level through production of interleukin-6 (IL-6). In this study, we examined the contribution of cAMP-dependent pathways on the upregulation of MyHC I mRNA levels in C2C12 cells. C2C12 cells were induced to differentiate to myotubes by medium exchange to D-MEM containing 2%FBS. The cells were incubated in D-MEM containing 2%FBS with chemical compounds at the beginning of differentiation and removed after 24hr, and were maintained in differentiation medium for 3 days. MyHC I mRNA expression level was measured by the real-time PCR method. MyHC I mRNA level was significantly increased by the administration of β-agonist, isoproterenol. The effects of forskolin and 8Br-cAMP on the MyHC I mRNA level were identical to that of isoproterenol. Additionally, the effect of forskolin on MyHC I mRNA expression was significantly inhibited by the co-administration of PKA inhibitor, H-89. However, MyHC I mRNA expression levels were not affected by the application of cAMP response element binding protein (CREB) inhibitor, 665-15. These results suggest that the upregulation of MyHC I mRNA level by the stimulation of β-agonist is not involved in cAMP/CREBmediated but in cAMP/PKA-mediated mechanisms in C2C12 cells.

### Poster Presentation 3

### [3P06]
### Muscle, Physical fitness and sports medicine, Oral physiology

March 18(Fri), 12:15 - 14:15, Zoom P6

### [3P06-01]
### Distribution of skeletal muscle fiber types in back muscle of a mammalian hibernator, Syrian hamster.

*Nanaka Matsuoka[1], Yoshifumi Yamaguchi[1,2] ([1] *Graduate School of Environmental Science,* [2] *Institute of Low Temperature Science Hokkaido university*)

**Distribution of skeletal muscle fiber types in back muscle of a mammalian hibernator, Syrian hamster.**

Hibernation allows animals survive during cold season in a chronic immobilization state. During the hibernation period that lasts for about two to six months, small mammalian hibernators, including Syrian hamsters (*Mesocricetus auratus*), undergoes a transition between two states, deep torpor, a hypothermic state of immobility lasting several days, and periodic arousal, a euthermic state of activity lasting about one day. In non-hibernators, such long-lasting immobility state leads to skeletal muscle atrophy, whereas it was reported that some hibernators maintain skeletal muscle function until the end of hibernation for survival during and after hibernation.
We also found previously that Syrian hamsters lose body weight and skeletal muscle mass during pre-hibernation period and maintain them during hibernation with a decrease in fast-twitch fiber Type IIb in their back muscle (*latissimus dorsi*). To understand where and how muscle atrophy occurs in back muscle before hibernation period, it would be important to clarify unknown distribution pattern of fiber-types in back muscle of Syrian hamsters. In this study, this point was examined, and the data will be presented.

### [3P06-02]
### Cancelled

### [3P06-03]
### Effects of omecamtiv mecarbil (OM) on the contractile properties of skinned porcine left atrial and ventricular muscles

*Tomohiro Nakanishi[1], Kotaro Oyama[2], Fuyu Kobirumaki[1], Takako Terui[3], Norio Fukuda[1] ([1]*Dept Cell Physiol, Jikei univ,* [2]*Takasaki Adv Rad Res Inst, QST,* [3]*Dept Anesth, Jikei univ*)

**Background:** OM has been developed to treat heart failure by activating cardiac myosin. **Results:** The force-pCa protocol was performed in skinned porcine left atrial (LA) and ventricular (LV) fibers to derive the midpoint of the force-pCa curve ($pCa_{50}$) as an index of $Ca^{2+}$ sensitivity. OM (0.5 and 1.0 $\mu$M) left-shifted $pCa_{50}$ in both LA and LV in a concentration-dependent manner, with a greater magnitude in LV. The $Ca^{2+}$-sensitizing effect of OM was diminished by ~50% in association with an increase in thin filament (TF) cooperative activation in both LA and LV, directly via TF reconstitution with fast skeletal troponin and indirectly via strongly-bound cross-bridges (+MgADP). Inorganic phosphate (Pi) markedly (~75%) inhibited the effect of OM in both LA and LV. **Discussion:** We interpret the findings as follows: First, the $Ca^{2+}$-sensitizing effect of OM is diminished when TF cooperativity is increased, due to a decrease in the fraction of "recruitable" cross-bridges. Second, Pi inhibits the effect of OM by blocking myosin binding to TF. It is therefore suggested that in both LA and LV, OM prolongs the myosin duty ratio, which increases TF cooperativity and promotes the binding of neighboring myosin to TF, and accordingly, force is augmented at submaximal $Ca^{2+}$ levels.

### [3P06-04]
### Involvement of potassium channels in purinergic regulation of esophageal motility in rats

*Takahiko Shiina[1], Kazuhiro Horii[1], Tomoya Sawamura[1], Yasutake Shimizu[1] ([1] *Laboratory of Veterinary Physiology, Faculty of Applied Biological Sciences, Gifu University*)

The external muscle layer of the mammalian esophagus consists of striated muscle fibers and smooth muscle fibers. Striated muscle is mainly regulated by cholinergic signaling, whereas smooth muscle is regulated by cholinergic and non-cholinergic signaling in the esophagus. ATP is a representative non-cholinergic extracellular transmitter, which control smooth muscle motility in the blood vessels and gastrointestinal tracts via purinergic receptors. We have demonstrated that exogenous application of ATP evokes relaxation of smooth muscle in the muscaris mucosa of the rat esophagus. In the present study, the aim was to clarify involvement of potassium channels in purinergic relaxation of the esophageal smooth muscle. An isolated segment of the rat esophagus was placed in an organ bath and the mechanical responses were recorded using a force transducer. After contraction of esophageal smooth muscle was induced by carbachol, we applied ATP, which evoked relaxation of smooth muscle. On the other hand, ATP did not affect high-potassium induced contraction. Pre-application of an antagonist of ATP-dependent potassium channels ($K_{ATP}$ channels) blocked ATP-induced relaxation, but not voltage-gated potassium channel blockers. These findings indicate that KATP channels might be involved in purinergic regulation of the motor activity in the esophageal smooth muscle.

### [3P06-05]
### Increased expression of TACAN in the rat muscle after lengthening contractions

*Hiroki Ota[1,2], Rihito Oi[1], Kimiaki Katanosaka[3], Toru Taguchi[1,2] ([1]*Dept. Phys. Ther., Fac. Rehabil., Niigata Univ. Health Wel., Niigata,* [2]*Inst. Human Move. Med. Sci., Niigata Univ. Health Wel., Niigata,* [3]*Dept. Biomed. Sci., Col. Life Health Sci., Chubu Univ., Kasugai*)

Pain or mechanical hyperalgesia is common after unaccustomed strenuous muscular work including lengthening contractions (LC). Recently, TACAN (also referred to TMEM120A) has been reported as an ion channel involved in sensing mechanical pain (Beaulieu- Laroche et al., *Cell*, 2020), and the channel plays a role in mechanical hyperalgesia after inflammation in the skin. However, the involvement of TACAN in pain after exercise is unknown. Here we measured the expression level of TACAN in the muscle in a rat LC-induced pain model. Under isoflurane anesthesia, male Sprague-Dawley rats were exposed to repetitive LC on the lower leg extensor muscles [mainly tibialis anterior (TA) muscle]. A shallow layer of the TA and crucial fascia (CF) covering the TA were excised for sampling 0, 6, 24, 48 and 120 h after LC. The expression level of TACAN mRNA was measured using real-time RT-PCR. In the TA, the mRNA expression was significantly increased 6, 24 and 48 h after LC, compared to naïve control. The time course of the upregulation was in parallel with nociceptive behaviors (Hayashi et al., *Eur J Pain*, 2017). In the CF, TACAN mRNA was not upregulated. These results suggest that TACAN upregulated in the muscle is involved in mechanical hyperalgesia after exercise. This work was supported by JSPS KAKENHI (JP19H03987 and JP20K11246), and partly by the AMED Grant (JP21gm0810010h0606). There were no conflicts of interest in this study.

### [3P06-06]

### Role of TRPC6 channels on stretch-induced change in contractile force in mouse cardiomyocytes

*Yohei Yamaguchi[1], Toshiyuki Kaneko[1], Gentaro Iribe[1] ([1]*Department of Physiology, Asahikawa Medical University*)

An increase in preload, namely the ventricular wall stretch, rapidly augments contractile force in the heart because of the Frank-Starling mechanism. However, its underlying mechanism is yet to be fully elucidated. We have previously reported that TRPC6, which are mechanosensitive non-selective cation channels, are involved with myocardial longterm stretch-induced changes in contractile force. In the present study, we investigated a role of TRPC6 on short-term stretch-induced changes in contractile force. Ventricular myocytes, isolated from the heart of either wild type (WT) or *Trpc6*$^{-/-}$ mice, were electrically stimulated at 4 Hz in normal Tyrode solution at 37 ℃. The slope of the endsystolic force-length relation curve (ESFLR), an indicator of cellular contractility, was significantly steeper in the *Trpc6*$^{-/-}$ mouse cardiomyocytes compared with the WT mice cells. Furthermore, a DNA microarray analysis was conducted to adress the impact of the absence of TRPC6 on gene expression. Several genes including collagen type I and metallothionein 2, which is a regulator of the Zn signaling pathway, increased in *Trpc6*$^{-/-}$ mouse ventricular muscle compared with WT mice. Further investigation is required to clarify the involvement of these factors in the short-term stretch-induced changes in contractile force.
(COI: No)

### [3P06-07]

### In vivo skeletal muscle x-ray diffraction study on the cause of reduced contractility after blood flow interruption

*Naoya Nakahara[1], Hideki Yamauchi[1], Maki Yamaguchi[1], Tomonori Hayashi[1], Kazuhiro Hirano[1], Shigeru Takemori[1] ([1]*The Jikei University School of Medicine*)

To monitor the progress of structural changes in muscle sarcomere with repetitive contraction, x-ray diffraction is a potentially suitable technique. However, dissection of muscle tissue limits the diffusion of gases and solutes to exacerbate metabolic deterioration. Therefore, we tried to obtain x-ray diffraction patterns from in vivo muscle with maintained blood supply during contraction. X-ray diffraction patterns from extensor digitorum longus muscle of anesthetized 6-month female ICR mice were taken at KEK. Every 30 sec, 100 Hz-stimulation was applied for 0.5 sec through nerve to contract the muscle, and the x-ray diffraction patterns were observed during contraction. After 10 stimuli, the muscles with preserved blood flow retained 90% of their contractile force of the first stimulation. Subsequent interruption of blood supply to the muscle dropped the contractile force by 40% in 10 stimuli. The 1,1/1,0 intensity and the first layer line of the patterns during contraction showed no significant difference from the muscles of preserved blood flow. These results suggest that ischemic muscle exhibits smaller tension without a failure in end-plate transmission and E-C coupling.

### [3P06-08]

### Effects of estrogen on resistance training induced myosin heavy chain isoform shifts in female rat skeletal muscle

*Yung-Li Hung[1], Ayami Sato[2], Yuka Takino[2], Akihito Ishigami[2], Shuichi Machida[1] ([1] *Juntendo University,* [2] *Tokyo Metropolitan Institute of Gerontology*)

Myosin heavy chain (MHC) isoform shifts are regulated by several physiological conditions, such as menopause and exercise. However, the effects of estrogen ($E_2$) on resistance training-induced MHC isoform shifts in females are unclear. Ten-week-old female rats were divided into 6 groups: sham sedentary (S-S), sham training, ovariectomy sedentary, ovariectomy training, ovariectomy $E_2$ treatment sedentary, and ovariectomy $E_2$ treatment training. Eight weeks after the operation, the rats in both the training groups were trained to climb a ladder while bearing a load. $E_2$ was administered using subcutaneous insertion of a 17 β-estradiol pellet. The flexor hallucis longus (FHL) muscles were collected and analyzed after 8 weeks. The FHL muscle possesses an MHC composition (3.5% MHCI, 22.8% MHCIIa, 41.7% MHCIIx and 31.9% MHCIIb) for S-S rats. The results showed that all the training groups revealed a higher proportion of MHC IIa and IIx compared with the sedentary groups. In particular, there was no MHC IIb in all the training groups. $E_2$ increased MHC IIx and reduced MHC IIb composition and the MHC gene expressions in the sedentary groups but not in the training groups. Our data showed that $E_2$ did not affect resistance training-induced MHC shifts.

### [3P06-09]

### The neuroprotective effect of nucleic acid analog, COA-Cl in acute phase after spinal cord injury.

*Naoyuki Himi[1], Issei Sakamoto[2], Emi Nakamura-Maruyama[1], Ikuko Tsukamoto[3], Osamu Miyamoto[4], Takehiro Nakamura[1] ([1]*Dept. Physiol. 2, Kawasaki Med. Sch.,* [2]*Dept. Orthop. Surg., Okayama Kyokuto Hosp.,* [3]*Dept. Pharm.-bio-Inform., Kagawa Univ.,* [4]*Dept. Med. Eng., Kawasaki Univ. Med. Welf.*)

Spinal cord injury (SCI) induces severe motor dysfunction. We previously showed the neuroprotective effects of COA-Cl, a novel synthesized adenosine analog, in a rat stroke model. In this study, we evaluated the neuroprotective effects of COA-Cl on SCI. SCI was induced by dropping a rod onto the spine of an anesthetized rat. Immediately after SCI, at a dose of 6 mg/kg COA-Cl per day for 5 days was given to the acute group. On the other hand, COA-Cl was administered from 4 days after SCI in the subacute group. Acute and subacute vehicle groups were injected saline as same as the injection of COA-Cl. Motor functions were evaluated by BBB scoring and inclined plane test and were significantly improved in the acute COA-Cl group compared to the other groups at 2 and 4 weeks after SCI. Histological study showed that cavity volume was significantly decreased in the acute COA-Cl group compared with other groups at 4 weeks after SCI. TUNEL staining revealed that apoptosis in the dorsal column was significantly decreased in the acute COA-Cl group at 7 days after SCI. In addition, phosphorylation of ERK1/2 at the rostral side of the epicenter of injury was significantly increased in the acute COA-Cl group at 7 days after SCI. These results suggest that the neuroprotective effects of COA-Cl exerts via the phosphorylation of ERK only in the acute phase after SCI, resulting in the decrease of cavity volume of spinal cord and recovery of motor function.

### [3P06-10]

### Contribution of peripheral adenosine receptor to analgesia by intramuscular injection of drugs

*Teruaki Nasu[1], Yuhei Hibino[2], Kimiaki Katanosaka[1,2], Kazue Mizumura[3] ([1]*College of Life and Health Sciences, Chubu University,* [2]*Graduate School of Life and Health Sciences, Chubu University,* [3]*Department of Physiology, Nihon University School of Dentistry*)

Intramuscular injection (i.m.), such as trigger point injection, is used for the treatment of chronic myofascial pain. To elucidate the mechanism of this analgesic effect, we investigated the contribution of adenosine-related molecules, which are known to be involved in analgesia, using the Repeated Cold Stress (RCS) model. We examined antagonists for adenosine receptor (AR) to the analgesic effect on i.m. of lidocaine (Lido), Neurotropin (NTP) and each antagonist were administered to the right gastrocnemius muscle of RCS rats under isoflurane inhalation anesthesia, then analgesic effect was evaluated based on the muscular withdrawal threshold (MWT) by the Randall-Selitto method. RCS significantly decreased MWT and Lido or NTP reversed this muscular hyperalgesia. MWT on the contralateral side was reversed as well. The prior administration of caffeine, an AR non-selective inhibitor abolished the analgesic effect of Lido. On the other side, prior administration of caffeine only partially inhibited analgesic effect of NTP. Pre-administration of Rolofylline, selective antagonist for adenosine 1 receptor, had a similar effects. Analgesia by intramuscular injection of Lido or NTP disappeared after treatment of inhibitors of the AR, suggesting that peripheral AR is involved in analgesia by intramuscular drug injection.

### [3P06-11]

### Analgesic and antioxidant effects of *Yokukansan*, a Japanese traditional Kampo formula, in rats with interstitial cystitis

*Mana Tsukada[1], Tatsuki Inoue[1,2], Yoshiki Tsunokawa[1,2], Takayuki Okumo[1], Tadashi Hisamitsu[1], Masataka Sunagawa[1] ([1]*Department of Physiology, School of Medicine, Showa University,* [2]*Department of Urology, School of Medicine, Showa University*)

Interstitial cystitis (IC) is a non-infectious inflammatory disease of unknown cause, and the Hannah-type in particular is difficult to treat, being designated an intractable disease in Japan. Yokukansan (YKS), a Kampo formula, is originally known to be effective in treating symptoms of mental diseases, such as neurosis and insomnia; however, recently, YKS has been reported to be effective against pain disorders. In this study, we investigated the analgesic effect of YKS on Toll Like Receptor 7 (TLR7) agonist-induced IC model rats and the involvement of the antioxidant action of YKS as a mechanism of action. Preadministration of YKS significantly suppressed hyperalgesia and elevated markers of oxidation in the bladder wall. Furthermore, when the antioxidant activity of YKS itself was examined, YKS was observed to have hydroxyl radical (•OH)-scavenging activity. Reactive oxygen species have been reported to be involved in the development of cystitis. These findings suggest that the antioxidant activity of YKS is involved in the analgesic effect in IC model rats.

### Poster Presentation 3

### [3P07]
### Physical fitness and sports medicine, Stress, Drug Action, Pharmacology

March 18(Fri), 12:15 - 14:15, Zoom P7

### [3P07-01]
**Pharmacokinetics/Pharmacodynamics modeling of the effect of E-4031 on cardiac action potentials in guinea pig**

*Azumi Sagehashi[1], Yuna Nakanishi[1], Rina Sato[1], Chinatsu Kobayashi[1], Yukiko Himeno[1], Akira Amano[1] ([1]*Ritsumeikan Univ.*)

Pharmacokinetics/Pharmacodynamics (PK/PD) modeling has been proposed to estimate drug concentration in the plasma and characterize the time course of drug effects through the application of mathematical modeling to experimental data. We investigated the effect of a hERG channel blocker, E-4031, on the action potentials (APs) recorded by the suction electrode method from the ventricular wall of guinea pigs and developed a PK/PD model of the effect. Firstly, the time course of the plasma concentration of the drug was estimated using PK/PD model whose parameters were determined referring to data from literatures as well as that from our *in situ* experiment. By comparing the time course of the AP prolongation obtained experimentally with that of the plasma concentration of the drug calculated using the PK/PD model, it was revealed that there was a delay in the effect of the AP prolongation. Secondly, the mechanism of the drug effect on the hERG channel was modeled using experimental data obtained from Langendorff heart and voltage clamp data recorded from hERG expressed cells in the literatures. As a result, the PK/PD model developed in this study successfully calculated the time course of drug effects and reproduced the delayed development of the AP prolongation.

### [3P07-02]
**Fuhalol-type phlorotannins from *Sargassum carpophyllum* are effective against the secretion of allergic mediators from antigen-stimulated rat basophilic leukemia cells**

*Takuya Matsui[1] ([1]*Aichi medical university, School of medicine, Department of Physiology*)

The phlorotannins 2-[2-(3,5-dihydroxyphenoxy)-3,5-dihydroxyphenoxy]-1,3,5-benzenetriol (**1**), 2,2'-[[2-(3,5-dihydroxyphenoxy)-5-hydroxy-1,3-phenylene]bis(oxy)]bis(1,3,5- benzenetriol) (**2**), and 2-[2-[4-[2-(3,5-dihydroxyphenoxy)-3,5-dihydroxyphenoxy]-3,5- dihydroxyphenoxy]-3,5-dihydroxyphenoxy]-1,3,5-benzenetriol (**3**) were isolated from *S. carpophyllum*. Here, we evaluated the anti-allergic activities of these compounds and comprehensively explored their effects on intracellular protein levels. Immunoglobulin E-sensitized rat basophilic leukemia cells pretreated with any of these three compounds exhibited reduced $\beta$-hexosaminidase, prostaglandin D2, and tumor necrosis factor-a secretion compared with dinitrophenyl-human serum albumin (DNP-HSA)-stimulated cells. Reduction of $\beta$-hexosaminidase release was dose-dependent but the half-maximal inhibitory concentrations of the compounds were similar (36-51 $\mu$M). Proteomics analysis revealed that the three compounds up-regulated 25 proteins and down-regulated 33 proteins compared with DNP-HSA stimulation alone, and slightly suppressed proteasome 5 expression linked to the regulation of IkB. These results demonstrate that these phlorotannins are potentially useful for preventing immediate hypersensitivity. *S. carpophyllum* may be a functional food.

### [3P07-03]
**Arrhythmia induction by a noninvasive indirect oral drug administration in freely behaving mice**

*Shinichi Sato[1] ([1]*Division of Behavioral Sleep Medicine, Iwate Medical University*)

**Purpose:** Previously, we reported the possible arrhythmia induction by transdermal absorption through mouse footpad skin after a pretreatment of walking on the paper soaked with 2% pilocarpine solution for 10 min. However, we reconsidered thereafter that the cause of arrhythmia might have been the glooming behavior, which resulted in oral intake of pilocarpine. Therefore, we assessed the transdermal drug absorption with avoiding oral intake. **Methods:** Four groups of anesthetized (urethane 1.6 g/kg, i.p.) mice were put on a heater pad and underwent lead-II ECG recording (ATC-402; Unique Medical Co., Ltd.); group 1: anesthesia only, pilocarpine (group 2) or metoprolol (group 3) solution was dropped on their paws, group 4: metoprolol transdermal patches (Bisono tape 8 mg; TOA EIYO LTD.) were adhered on their paws. **Results:** The heart rate time courses were similar in group 1 and 2. The group 4 but not group 3 mice showed significant decreases in heart rate. **Conclusion:** The present study suggests that the pretreatment enables noninvasive indirect oral drug (pilocarpine) administration, which causes arrhythmia induction in unanesthetized freely behaving mice.

### [3P07-04]
**Effect of clenching on spinal alignment**

*Mutsumi Takahashi[1], Yogetsu Bando[2], Takuya Fukui[3,4], Akiko Maruyama[3,5], Sugita Masaaki[6], Yoshihide Satoh[1] ([1]*Dept Physiol, Nippon Dent Univ,* [2]*BANDO Dental Clinic,* [3]*Dept Sport Sciences, Facul Sport Sci, Kanazawa Gakuin Univ,* [4]*JGA, Tra, Commit,* [5]*JGA, Tra, Reinforce,* [6]*Facul Sport Sci, Nippon Sport Sci Univ*)

The aim of this study was to clarify the effect of occlusion on the spinal alignment. Participants were healthy men with no medical history involving the stomatognathic system and no musculoskeletal, neurological, or orthopedic problems. Thoracic kyphosis angle (TKA), lumbar lordosis angle (LLA), sacral slope angle (SSA), and spinal inclination angle (SIA) were measured using a spinal shape analyzer. Measurements were performed under two conditions: while clenching and while relaxing, that were carried out in a static standing and in a standing forward-bending. In each posture, the difference in the spinal alignment depending on the clenching conditions were analyzed by the paired t-test or the Wilcoxon signed rank test. In the static standing posture, there was no difference in the spinal alignment depending on clenching condition. However, in the standing forward-bending posture, LLA, SSA, and SIA values were significantly higher while relaxed than that while clenching. As a result of this study, it was clarified that clenching influences the alignment of the spine during trunk flexion and restricts the flexion of the body, thereby contributing to the stability of the trunk.

### [3P07-05]
**Effects of optokinetic stimulation with a see-through head-mounted display on postural stability and leg muscle activity**

*Junya Komagata[1], Atsushi Sugiura[2], Atsuya Otsuka[1], Kitama Toshihiro[2] ([1]*Dept of Physical Therapy, Health Science University,* [2]*Center for Life Science Research, Univ of Yamanashi*)

Weight-bearing adjustment and muscle strengthening are critical for stroke rehabilitation. This study examined the effects of optokinetic stimulation (OKS) on postural stability and electromyography (EMG) in the legs of normal subjects. Four healthy students were asked to keep a static balance posture under OKS via a see-through Head Mounted Display (HMD). For the OKS, a random dots pattern in a virtual 3D space was continuously moved in horizontal and torsional directions. Postural stability was evaluated by the center of pressure position, sway path length (SP), and sway area (SA). EMG was recorded using bipolar surface electrodes from four leg muscles—tibialis anterior (TA), gastrocnemius (GC), quadriceps femoris (QF), and femoral biceps (FB). During both the OKS, SP and SA were higher than that during stationary condition. TA and QF activity clearly increased in the side of OKS direction during OKS in two subjects, whereas no clear changes were found in GC and FB. These results suggested that OKS induces an increase in EMG activity and weight-bearing shift. OKS through HMD could be applied in stroke rehabilitation.

### [3P07-06]
### Effects of medium- and high-intensity sustained exercise with vocalization on ventilatory dynamics and muscle oxygen status - cases of subjects unaccustomed to vocalization during exercise -

*Hajime Arikawa[1], Tomoyoshi Terada[2], Kanako Yamada[2], Teppei Takahashi[3], Hajime Imai[2], Seiichi Era[4] ([1]*Chubu Gakuin Univ.*, [2]*Gifu Univ.*, [3]*Takahashi Dental Clinic*, [4]*Japanese Red Cross Gifu Hospital*)

[Aims] A tendency of increasing $O_2$ supply to the active muscles is reported in sustained upper-body exercise with vocalization due to ventilation suppression at 80%$VO_2$peak load. However, those unaccustomed to vocalization may not be able to continue the same. This study aimed to investigate the effects of vocalization during exercise on ventilatory dynamics and muscle oxygen status of active muscles in subjects unaccustomed to vocalization. [Methods] Six men, not engaged in any exercise with vocalization (e.g., kendo), performed sustained upper-body exercises (60% or 80%$VO_2$peak load) in two trials: with or without vocalization. We measured the ventilation indexes, including the minute ventilation (VE) and end-tidal carbon dioxide concentration ($FetCO_2$), triceps brachii muscle oxygen status (TSI%) from the start till the end of the exercise program, and the rate of perceived exertion (RPE) after exercise. [Results] At 80%$VO_2$peak load, ΔVE was not suppressed. Further, Δ$FetCO_2$ showed a significantly higher value with no difference in ΔTSI%. Therefore, we considered that blood $CO_2$ levels in this study did not affect oxygen dissociation. At 60%$VO_2$peak load, differences in ΔVE, Δ$FetCO_2$, and ΔTSI% were not observed. At both intensities, an increase in RPE was observed, implying the occurrence of "dyspnea" associated with vocalization. [Conclusions] We speculated that "dyspnea" accompanying vocalization can cause insufficient vocalization in subjects unaccustomed to vocalization.
(COI: NO)

### [3P07-07]
### Mouse plasma after acute high intensity interval exercise enhance mitochondrial maximal respiration of C2C12 myotube

*Shunsuke Sugiyama[1], Takanaga Shirai[3,4], Riku Tanimura[2], Tohru Takemasa[4] ([1]*School of Physical Education, Health and Sport Sciences, University of Tsukuba*, [2]*Graduate School of Comprehensive Human Sciences, University of Tsukuba*, [3]*Research Fellow of the Japan Society for the Promotion of Science*, [4]*Faculty of Health and Sports Sciences, University of Tsukuba*)

Exercise has beneficial effects for health such as prevention of disease and extension of healthy life span, and they are known to vary greatly depending on the modality. Exerkines (secretory factors that evoked by exercise) may be one of the factors that contribute for the health benefits of exercise. We examined whether exercise modalities affect the respiratory capacity of cultured skeletal muscle cells. In this study, we used 8-week-old ICR male mice and collected blood and muscle samples immediately after exercise. Mice were divided into four groups by exercise modality: control (Con), resistance exercise (RE), endurance exercise (EE), and high-intensity interval exercise (HIIE). C2C12 myotubes were treated with media containing 10% plasma from mice in each group. The cells added HIIE plasma showed the highest maximal respiration compared with those from mice in other groups. These results suggest that HIIE increase mitochondrial maximal respiration by altering exerkines in the blood.

### [3P07-08]
### Evaluating mouse defecation behavior by using Shannon entropy of the spatial distribution of fecal pellets

*Kensaku Nomoto[1], Kenji Kansaku[1] ([1]*Dokkyo Medical Univ.*)

A wide variety of animal species, from birds to mammals, are known to have designated areas for defecation and urination. It is anecdotally observed that this behavior is disrupted in stressed mice, which is a characteristic of self-neglect. Nevertheless, due to the lack of objective and quantitative methods for defecation behavior in mice, the effect of chronic stress on defecation behavior has not been experimentally verified. In this study, we developed a new method to quantify defecation clutteredness by calculating the Shannon entropy of the spatial distribution of fecal pellets, which we defined as a clutter index. C57BL/6N mice were exposed to 0.1 mg/ml corticosterone dissolved in 1% ethanol solution (CORT mice) or 1% ethanol solution (VEH mice) via the drinking water for at least three weeks. After corticosterone administration, we found that the clutter index of CORT mice was significantly higher than that of VEH mice, suggesting that defecation in CORT mice was more disorganized. Furthermore, we found that CORT mice made poorer nests, compared to VEH mice, which may also be a characteristic of self-neglect. These results suggest that the clutter index is useful for evaluating defecation behavior in mice.

### [3P07-09]
### Relationships between resilience against anxiety-related behavior and the psychological characteristic in undergraduate students

*Ken-ichi Tanaka[1], Konno Michiko[1] ([1]*Saitama Prefectural University*)

We investigated to clarify between resilience against anxiety-related behavior and the psychological characteristic using questionnaire methods in undergraduate students. For 167 university students (64 male, 103 female), we carried out this study with the approval of the Saitama Prefectural University Ethical Review Board (28095, 21055). For this study, we examined association and the correlation with the resilience mainly against anxiety-related behavior using the questionnaire method (SHRT: Sukemune-Hiew Resilience Test) and the psychological characteristic based on resilience systems existing evaluation five kinds (GHQ-30: General Health Questionnaire, STAI: State-Trait Anxiety Inventory-JYZ, SACL: Stress Arousal Check List, RSES: Rosenberg Self Esteem Scale, RRS: Rumination Response Scale). As a result, the correlation with some psychological characteristic considered to participate in the process of acquisition of resilience was accepted. In addition, from the result according to five psychological characteristics, the possibility that resilience against anxiety-related behavior correlated rumination or positive self-esteem. Thus, the possibility as the evaluation standard that we detected the power of resilience related some psychological characteristic, especially rumination or positive self-esteem.

### [3P07-10]
### The response to lower intestinal peristalsis evoked by stimulation of the hypothalamic stress center in the rat

*Naoya Kikuchi[1], Mio Matsuyama[2], Nao Suzuki[2], Yuka Ichinotsuka[2], Joji Horiuchi[1,2] ([1]*Department of Biomedical Engineering, Toyo University*, [2]*Department of Biomedical Engineering, Toyo University*)

Psychological stress induces the sympathoexcitatory cardiovascular responses. On the other hand, an irritable bowel syndrome, one of stress-related diseases, has symptoms of diarrhea or constipation, and either mechanism has not been elucidated. In particular, the diarrhea may be evoked by an increased intestinal motility consequence of parasympathetic dominance and is inconsistent with the stress-induced cardiovascular response. We hypothesized that psychological stress may disrupt the balance of the autonomic nervous system and affect intestinal motility. To test this, the hypothalamic stress center, the dorsomedial hypothalamic area (DMH) was chemically stimulated and recorded a bowel peristalsis. Suppression or hyperactivity of intestinal motility reaction was observed during the DMH stimulation. In case of the hyperactivity, the onset time was not uniform. Injection sites where were increased the intestinal motility were distributed in the medial area of the DMH, while sites of the suppressing intestinal motility were observed in the latera area of the DMH. Therefore, these results suggest that 2 different types of neural population may be involved in the control of the bowel peristalsis during psychological stress.

### [3P07-11]
### Enrich environment causes the emotional and behavioral changes to the mouse model of autism spectrum disorder

*Shuhei Koeda[1], Honami Yanagimachi[1], Misaki Mikami[1], Chihiro Sato[1], Junko Yamada[1] ([1]*Hirosaki University Graduate School of Health Sciences*)

Autism spectrum disorder (ASD) is a neurodevelopmental disorder characterized by impairments in social communication. In this study, we performed Enrich Environment (EE) as treatment for the valproic acid (VPA) exposed ASD model mice and examined effect on emotion and behavior.
C57BL6J pregnant female mice were administered VPA (600 mg/kg) or saline (CTL) at gestational day 12.5. After weaning, male pups were divided into four groups; CTL(n=5), CTL+EE(n=8), VPA(n=7) and VPA+EE(n=7). They were tested for Open Field Test, Elevated Plus Maze, Eight-arm radial maze test and Three Chamber Test before and after intervention. The EE mice were raised in the cage with the running wheel, tunnel, nest, nest building materials for 5weeks. In addition, the corticosterone concentrations was measured after the intervention (ELISA).
To evaluate the effect of EE, data were analyzed before and after intervention. VPA+EE group had increasing value than before intervention in the stranger zone stay ratio of Three Chamber Test and the central stay rate of Open Field Test ($p<0.05$).
As a result of EE intervention, the improvement of the behavior test was found in EE groups. It is thought that voluntary exercise and security by the EE intervention was associated with the improvement of the behavior test. In this study, it was shown that the EE was effective for treatment of ASD.

### Poster Presentation 3

### [3P08]
### Behabior, Biological rhythm, Sleep, Others

March 18(Fri), 12:00 - 14:00, Zoom P8

### [3P08-01]
### Chronic kidney disease correlates with uremic indoxyl sulfate levels to reduce cognitive function.

*Hirobumi Tada[1,2], Akinori Tokunaga[3,2], Kazutaka Akagi[4,2], Ayano Konagai[5] ([1]*Shigakkan University,* [2]*National Center for Geriatrics and Gerontology,* [3]*University of Fukui,* [4]*University of Toyama,* [5]*Kureha Corporation, Central Research Laboratories*)

The mechanism of dementia exacerbation through peripheral tissues has been emphasized. Many of the risk factors for dementia originate from peripheral tissue damage due to aging and changes in the living environment, and strategies to treat dementia by redeploying drugs that target the periphery have recently begun in earnest.In this study, we analyzed the effects of chronic kidney disease (CKD) on cognitive function, focusing on renal function, which is strongly affected by aging and changes in the living environment. In the 5/6 nephrectomy CKD model mice, the spatial working memory using the Y-maze test was impaired when blood levels of the uremic toxin indoxyl sulfate increased. Furthermore, analysis of the cognitive function of CKD mice fed a diet containing the CKD therapeutic agent, adsorbed charcoal AST120 (CKD-AST120 mice), showed that the CKD-AST120 mice had suppressed blood indoxyl sulfate levels and improved spatial working memory deficits. These results suggest that cognitive function is impaired in relation to the amount of blood indoxyl sulfate.

### [3P08-02]
### Chemogenetic activation of endogenous arginine vasopressin exerts anorexigenic effects via central nesfatin-1/NucB2 pathway

*Kenya Sanada[1,2], Mitsuhiro Yoshimura[1], Makiko Shimizu[1], Naofumi Ikeda[1], Kazuhiko Baba[1], Takashi Maruyama[1], Tetsu Miyamoto[2], Masaharu Kataoka[2], Yoichi Ueta[1] ([1]*Department of Physiology, School of Medicine, University of Occupational and Environmental Health, Japan,* [2]*Second department of Internal Medicine, School of Medicine, University of Occupational and Environmental Health, Japan*)

We examined whether the chemogenetic activation of endogenous arginine vasopressin (AVP) affects central nesfatin-1/NucB2 neurons, using a transgenic rat line that was previously generated. Saline (1 mL/kg) or clozapine-N-oxide (CNO, 1 mg/mL/kg), an agonist for hM3Dq, was subcutaneously administered in adult male AVP-hM3Dq-mCherry transgenic rats (300-370 g). Food and water intake were significantly suppressed after subcutaneous (s.c.) injection of CNO, alongside with developing aberrant circadian rhythmicity. The percentages of Fos expression in nesfatin-1/NucB2 immunoreactive neurons were significantly increased in the hypothalamus and brainstem at 120 min after s.c. injection of CNO. Suppressed food intake that was caused by chemogenetic activation of endogenous AVP was ablated after intracerebroventricularly administered nesfatin-1/ NucB2-neutralizing antibody in comparison with vehicle, without any alteration of water intake nor circadian rhythmicity. These results suggest that chemogenetic activation of endogenous AVP affects, at least in part, central nesfatin-1/NucB2 neurons and may exert anorexigenic effects in the transgenic rats.

### [3P08-03]
### Establishment of a Model Mouse Pedigree with hypersomnia and obesity by EEG/EMG-Based Forward Genetic Screening

*Chika Miyoshi[1], Noriko Hotta[1], Satomi Kanno[1], Aya Ikkyu[1], Miyo Kakizaki[1], Masashi Yanagiaswa[1,2], Hiromasa Funato[1,3] ([1]*IIIS, University of Tsukuba,* [2]*University of Texas Southwestern Medical Center,* [3]*Toho University*)

Sleep disorder such as insomnia is related to mood disorder such as depression and metabolic syndrome. Abnormal sleep/wake behavior and impaired eating behavior are thought to be closely correlated, but their molecular mechanisms are not clear. The network of genes and molecules that govern sleep and wakefulness remains largely unknown. We conducted a large-scale screening system using EEG/EMG-based sleep/wake monitoring, and have established a Sleepy mutant pedigree, which shows a significant reduction in awakening time (Funato, Miyoshi et al., Nature 2016). Sleepy have a mutation in the Salt-inducible kinase 3(Sik3) gene belonging to AMP-activated kinase-related kinase (AMPK-RK) and show a shortened wake time as a typical phenotype. Sleepy is also accompanied by significant weight gain. This mouse can be a model animal having both the hypersomnia and obesity phenotypes that are considered to be socially important phenotypes. We created not only systemic sleepy mutant SIK3 mice, but also flox mice that conditionally express it, and tried an integrated analysis of sleep and metabolic systems.

### [3P08-04]
### Optical recording of mitochondrial $Ca^{2+}$ dynamics in the central circadian clock

*Sota Hiro[1,2], Tomomi Nemoto[1,2,3], Ryosuke Enoki[1,2,3] ([1]*Department of Physiological Sciences, School of Life Science, The Graduate University for Advanced Studies, SOKENDAI,* [2]*Division of Biophotonics, National Institute for Physiological Sciences, National Institute of Natural Sciences,* [3]*Biophotonics Research Group, Exploratory Research Center on Life and Living Systems, National Institutes of Natural Sciences*)

In mammals, physiological functions like the sleep-wake cycle, hormone secretion, core body temperature show the circadian rhythm. The central circadian oscillator locates in the suprachiasmatic nucleus (SCN) of the hypothalamus, containing 20,000 neurons. There is autonomous and spatiotemporally organized circadian rhythm in firing activity, gene expression, and intracellular $Ca^{2+}$ concentration. The circadian rhythm of intracellular $Ca^{2+}$ concentration (Circadian $Ca^{2+}$ Rhythm: CCR) contributes to the robustness and temperature compensation of self-sustaining oscillation. However, the mechanism of how SCN neurons generate CCR remains unclear. Recently, it has been showed that a $Ca^{2+}/H^{+}$ antiporter LETM1, an inner mitochondrial membrane protein, might have critical roles in the cytoplasmic CCR. To further investigate the mitochondrial $Ca^{2+}$ dynamics in the SCN neuron, we here performed time-lapse fluorescence imaging for 3 to 5 days by a genetically-encoded mitochondria-specific $Ca^{2+}$ probe, CEPIAmt. As a result, the series of images clearly showed the existence of CCR in the mitochondria. Noticeably, cytoplasmic and mitochondrial CCRs visualized simultaneously by the two different color $Ca^{2+}$ probes showed that the mitochondrial CCR was antiphasic to the cytosolic one. These results suggest that the mitochondria might involve the cytoplasmic CCR by supplying or taking up $Ca^{2+}$.

### [3P08-05]
### Orally continuous administration of Kamikihito improved social memory of *Oxytocin gene* deficient mice

*Shizu Hidema[1], Yuko Maejima[1], Kenju Shimomura[1], Keita Mizuno[2], Katsuhiko Nishimori[1] ([1]*Fukushima Medical University,* [2]*Tsumura Kampo Research Laboratories, Tsumura & Co.*)

Nonapeptide oxytocin (Oxt) is known as neuromodulator and neurotransmitter, and has been revealed to play essential roles in various types of social behaviors, such as feeding behavior, anxiety, stress response, and thermoregulation in CNS. Oxt neurons extend axons from PVN and SON to various parts of the nuclei expressing Oxt receptor (Oxtr) and release Oxt from their synaptic terminals. Oxt exerts various physiological functions via the Oxtr. Kamikihito (KKT) is a traditional Japanese medicine used to treat insomnia, anemia, anxiety and neurosis. Pharmacological studies have shown that KKT is effective for behavioral abnormalities and cognitive dysfunction, suggesting that KKT has an effect on CNS. Recently we reported that KKT could directly activate OXT neurons via Oxtr in PVN and induced Oxt secretion by intraperitoneally and orally administrated KKT to rat (Maejima et al., 2021). In this study, we focused on the effect of KKT on social behavior of *Oxt gene*-deficient mice with impaired social memory. The social memory of *Oxt gene*-deficient mice, measured by 3 chamber-test, was not affected by a single dose administration of KKT, but improved by continuous administration of KKT for 21 days. We're continuing to analyze the neural activation, especially in the regions related to social memory in the brain, to understand the pharmacological and physiological mechanisms about the amelioration of social memory observed after the continuous administration of KKT to the tested mice.

### [3P08-06]
### Longitudinal analysis of primate progressive Parkinson's disease model revealed slowing in saccade reaction time.

*Wajd Amly[1], Chih-Yang Chen[1,2], Hirotaka Onoe[3], Tadashi Isa[1,2,3] ([1] *Department of Neuroscience, Graduate School of Medicine, Kyoto University,* [2] *Institute for the Advanced Study of Human Biology (WPI-ASHBI), Kyoto University,* [3] *Human Brain Research Center, Graduate School of Medicine, Kyoto University*)

The oculomotor system is not just a motor or a visual system, but also reflects the cognitive state and thus, is widely used to probe brain functions and neural disorders in humans. The fact that internally and externally driven saccades are generated by different brain structures and have different kinematics has motivated us to investigate the pathophysiology underlying Parkinson's disease (PD) by comparing the internally and externally driven saccades in the same animal. To approach this, we used and trained three common marmosets on the externally driven saccadic tasks; step and gap tasks, and on the internally driven one; the oculomotor delayed response task (ODR). After collecting baseline data, we induced PD by injecting $\alpha$-Synuclein fibrils into the olfactory bulb (OB) bilaterally, following Braak's dual hit hypothesis. With a longitudinal follow-up, we found that saccade reaction time (SRT) gradually got slower with the disease progression in both tasks. We also found that SRT in the step saccade task was less affected than in the gap task. Furthermore, we found that the level of attention and arousal of the marmoset decreased with the progression of the disease in the ODR task. In conclusion, the OB $\alpha$- Synuclein disease model showed a change in SRT and attention level as PD progressed with time.

### [3P08-07]
### Behavioral evaluation of rat with focal cerebral infarction by 3D kinematic analysis

*Tatsuro Kumada[1], Akira Yoshikawa[2], Saho Morishita[1], Kazuya Hokamura[3], Kazuo Umemura[3] ([1] *Tokoha University,* [2] *Showa Univ.,* [3] *Hamamatsu Univ. Sch. Med.*)

Precise assessment of behavior allows to reveal abnormalities in the rodent disease model and is required for investigating the role of neurorehabilitation. While a battery of behavioral tests can assess multiple aspects of behavior, there is a technical limitation to detecting motor deficits. Thus, we have evaluated the motor ability in a Spatio-temporal manner through three-dimensional (3D) kinetic analysis methods. Previously, we found that rats with focal motor cortex infarction showed minor motor deficits, which were only detected by beam-walking tests. Here, we further addressed the motor deficits by 3D kinematic analysis. There were no significant differences in parameters during a step cycle between pre-and post-operated rats. However, kinematical analysis of each joint on different planes revealed significant differences in certain parameters in PIT-operated rats compared to pre-operated ones. Especially, these differences were apparent in the distal portion. These results suggest that a more precise motor evaluation by 3D motor analysis should unmask the motor deficits in focal motor infarction.

### [3P08-08]
### The circadian phenotype of newly developed *Vipr2* knock-out rats

*Yoichi Minami[1,2], Mamoru Nagano[2], Satoshi Koinuma[2], Xiaonan Xie[5], Atsuko Kubo[2], Takamitsu Morimoto[2], Atsuhiro Tatemizo[3], Kentaro Egawa[3], Masayuki Iigo[4,5], Yasufumi Shigeyoshi[2] ([1] *Graduate School of Medicine, Department of Systems Pharmacology, The University of Tokyo,* [2] *Department of Anatomy and Neurobiology, Faculty of Medicine, Kindai University,* [3] *Central Research Facilities, Faculty of Medicine Center for Animal Experiment, Kindai University,* [4] *Department of Applied Biological Chemistry, School of Agriculture, Utsunomiya University,* [5] *Bioscience Education and Research Center, Utsunomiya University*)

The circadian clock is an endogenous mechanism for making a day. The period length depends on the species; the period lengths of clocks in humans and rats are longer than 24 hours, whereas that in mice is shorter than 24horus. The period determination mechanism has yet to be elucidated partly because of no good non-mouse gene modified animal model available. Vasoactive intestinal peptide (VIP) is a peptide hormone and is known to function to synchronize the cellular clocks in the suprachiasmatic nucleus (SCN) of the clock center. Both mice lacking *Vip* or its receptor *Vpac2* (also known as *Vipr2*) show severe defects in behavioral rhythm and the circadian clock oscillations in the SCN. Here, we report our newly developed *Vipr2* deficient rats using CRISPR/Cas9 methods. This *Vipr2* KO rats showed similar behavioral phenotype to *Vpac2* KO mice; no rhythmicity or short period rhythm with low amplitude. Spatio-temporal analysis by *in situ* hybridization revealed that *Vipr2* disruption affected the *Per2* expression patterns differently in the ventrolateral and dorsomedial SCN. By crossing the animals to *Per2*-dLuc transgenic rat, we observed that the clock oscillation dampened sooner in the SCN of *Vipr2* KO rat. In this meeting, we would like to discuss the potential of our *Vipr2* KO rats as a new circadian clock dysfunction model.

### [3P08-09]
### Suppressive Effect of *Boiogito* on MMP-13 Production by Fibroblastlike Synoviocyte from Knee Osteoarthritis *In Vitro*

*Takayuki Okumo[1,2], Midori Mochizuki[1], Hideshi Ikemoto[1], Naoki Adachi[1], Taro Kimura[1,2], Yasunori Takayama[1], Mana Tsukada[1], Masataka Sunagawa[1] ([1] *Department of Physiology, Showa University School of Medicine,* [2] *Department of Orthopedic Surgery, Showa University Fujigaoka Hospital*)

Synovitis is profoundly involved in the initial pathophysiology of osteoarthritis (OA) and is also a very important therapeutic target. We previously reported that boiogito (BO), a Japanese traditional kampo medicine, prevented the progression of knee OA in a trauma-induced knee OA rat model and hypothesized that BO indirectly suppresses chondrocyte degeneration by alleviating synovitis. Therefore, we developed an *in vitro* synovitis model of fibroblast-like synoviocytes derived from knee OA patients (HFLS-OA) and investigated the therapeutic effect of BO. BO was administered before stimulating culture cells with 10 ng/ml IL-1$\beta$. Twenty-four hours after applying IL-1$\beta$, the HFLS-OA were examined for cell viability by an MTT assay and MMP-13 as catabolic markers of OA both by qRT-PCR and ELISA. The cell viability did not differ between IL-1$\beta$ and BO administration, and HFLSOA stimulated by IL-1$\beta$ showed an increased production of MMP-13, which were inhibited by BO. These findings thus suggest that BO may alleviate the pathogenesis of synovitis and inhibit OA progression.

### [3P08-10]
### Analgesic effect of voluntary running on persistent inflammatory pain via inhibition of microglia activation and BDNF/TrkB signaling

*Ikemoto Hideshi[1], Adachi Naoki[1], Takahara-Yamauchi Risa[1], Horikawa Hiroyuki[2], Okumo Takayuki[1], Chuluunbat Oyunchimeg[1], Hisamitsu Tadashi[1], Sunagawa Masataka[1] ([1] *Department of Physiology, School of Medicine, Showa University,* [2] *Faculty of Arts and Sciences at Fujiyoshida, Showa University*)

We investigated whether microglial activation and brain-derived neurotrophic factor (BDNF)/tropomyosin-related kinase B (TrkB) signaling in the spinal cord are involved in the analgesic effect of voluntary running (VR) on persistent inflammatory pain. Male rats were assigned to the control group, non-running group (NOR) after formalin injection, and VR group after formalin injection. Inflammation was induced by injection of formalin (50$\mu$l, 1%) into a hind paw. VR was applied for 7 days after injection. The von Frey test was performed to determine inflammatory sensitization. To investigate the involvement of activation of microglia and BDNF/TrkB signaling, the expression levels of Iba1, phosphorylated p38 mitogen-activated protein kinase (p-p38), and TrkB in the spinal cord were evaluated. In the NOR group, the withdrawal latency was shortened and the expression levels of Iba1, p-p38, and TrkB were significantly increased compared to the control group, while VR significantly suppressed these changes. These results suggest that VR may prevent central sensitization caused by formalin injection via suppressing activation of microglia and BDNF/TrkB signaling.

### [3P08-11]
### Yokukansan Suppresses the Development of Morphine Tolerance by Regulating Synaptic Functions in DRG Neurons

*Naoki Adachi[1], Yusuke Ohashi[1], Fatma Zahra Sakhri[1,2], Hideshi Ikemoto[1], Wakako Yogi[1], Takayuki Okumo[1], Masataka Sunagawa[1] ([1] *Department of Physiology, School of Medicine, Showa University,* [2] *Université des Frères Mentouri-Constantine 1, Département de Biologie Animale*)

Opioids, most notably morphine, continue to be the gold standard for the treatment of acute and chronic pain. However, prolonged use of morphine in pain treatment decreases efficacy and increases sensitivity. Recent studies have shown that opioids alter the properties of mu-opioid receptor (MOR)-expressing neurons and nociceptive circuits at the level of the dorsal root ganglia (DRG) and spinal dorsal horn. Yokukansan (YKS), a Kampo medicine, consisting of seven herbs has been used to treat emotional instability, neurosis, and insomnia, and has been reported to weaken morphine tolerance.In the present study, we determined the effect of YKS on morphine tolerance and its mechanisms in rats, focusing on the synaptic functions between DRG neurons and spinal dorsal horn secondary neurons.We found that pretreatment with YKS for 7 days significantly inhibited the development of morphine tolerance. And our results indicate that YKS suppressed morphine tolerance by inhibiting the enhancement of presynaptic function of DRG neurons projecting to spinal dorsal horn neurons caused by continuous morphine administration.

### Poster Presentation 3

### [3P09]
### Oral physiology, Study Methodology, Others

March 18(Fri), 12:00 - 14:00, Zoom P9

### [3P09-01]
**Disentangling the parallel pathways from the ventromedial frontal cortex to the amygdala: An anterograde tracing study in rodents**

*Paola Aleman Andrade[1], Christine L. Marchena[1], Shinya Ohara[2], Ken-Ichiro Tsutsui[1,2] ([1] *Laboratory of Systems Neuroscience, Tohoku University Graduate School of Medicine,* [2] *Laboratory of Systems Neuroscience, Tohoku University Graduate School of Life Sciences*)

Projections from the ventromedial frontal cortex (vMFC) to amygdala (AMG) have been focus of research due to their involvement in emotional regulation. In traditional rodent studies, vMFC consisted of infralimbic (IL) cortex. The most ventrally located vMFC region, the dorsal peduncular (DP) cortex, has been largely overlooked. Some recent studies suggest that IL plays an important role in fear extinction and that DP may drive sympathetic stress responses. The first step to fully identify their roles in emotional modulation may be to unravel their projections to AMG subareas.
To examine the anatomical connectivity from vMFC to AMG, we injected anterograde chemical tracers (either PHA-L or BDA) in IL and DP of both mice and rats. We evaluated the distribution of labeled axons among the AMG nuclei. The results show that DP mainly projects to the anterior and posterior parts of the basomedial AMG (BMAa, BMAp) whereas IL targets mainly the anterior part of basolateral AMG (BLAa) and BMAp. The described IL and DP parallel pathways to AMG may play important and distinct roles in emotional regulation.

### [3P09-02]
**Compact and lightweight data logger for recording neural activity in monkeys**

*Ryoi Tamura[1] ([1] *Department of Physiology, Faculty of Medicine, University of Toyama*)

We have recorded neural activity from the hippocampus of freely moving monkeys. In the experiment we sent the recorded signals to the post-processing equipment via a transmission cable attached to the head of the monkey, which often caused troubles such as the monkey damaging the cable, the cable interfering with the monkey's behavior, etc. Telemetries and data loggers are possible devices to overcome these problems. While telemetry is suitable for recording in a relatively narrow environment where reflection of the electromagnetic wave is unlikely to occur, stable recording is difficult for freely behaving animals in a wide environment with electrically interfering objects. Therefore, we have recently developed a data logger and completed a prototype 2 years ago. However, it was relatively large, weighing about 100 g excluding the battery and with a volume of about 120 $cm^3$, so this time we tried to make it smaller and lighter. The data logger can be roughly divided into an amplifier part and a data collection/storage part. In the amplifier part, the input signals (4Ch) were amplified by instrumentation amplifiers, band-pass filtered, and amplified to desired magnification by post stage amplifiers (total gain: x1000-x2000). In the data collection/storage part, the output signals from the amplifier part were AD-converted (50 kHz/Ch) by a PIC microcontroller and stored on a micro SD card. As a result of designing and fabricating a dedicated printed circuit board and using small surface-mounted components, we were able to achieve an 80% reduction in size and weight (20 g, 18 $cm^3$) while maintaining the same basic performance as the prototype.

### [3P09-03]
**An easy-to-use light-needle creating device for single scan volumetric imaging in thick tissue specimens**

*Ching-Pu Chang[1,2,3], Kohei Otomo[4,1,3], Yuichi Kozawa[5], Hirokazu Ishii[3,1], Miwako Yamasaki[2], Masahiko Watanabe[2], Shunichi Sato[5], Ryosuke Enoki[1,3,6], Tomomi Nemoto[1,3,6] ([1] *National Institute for Physiological Sciences, National Institutes of Natural Sciences,* [2] *Graduate School of Medicine, Hokkaido University,* [3] *Exploratory Research Center on Life and Living Systems (ExCELLS), National Institutes of Natural Sciences,* [4] *Graduate School of Medicine, Juntendo University,* [5] *Institute for Multidisciplinary Research for Advance Materials, Tohoku University,* [6] *Graduate School of Advanced Studies Sciences (SOKENDAI)*)

The biological tissues and therein networks often change dynamically across a large volume. Understanding the operations of the networks requires monitoring their activities three-dimensionally with a single-cell resolution. In this sense, several researchers have proposed volumetric imaging technologies. However, most proposals require a complicated optical setup and deep expertise for microscopic mechanisms, resulting in a high threshold for usage. Here, we propose a light-needle creating device that enables easy Bessel beam scanning on conventional two-photon microscopy systems for volumetric imaging. The developed device was placed in a filter cube position in the turret that most standard laser microscopes equip, warrant to simultaneously excited fluorophores placed throughout over 200-μm thickness fixed specimens by single-scanning an excitation laser light beam. In G-CaMP7 expressing somatostatin interneurons in 250-μm acute mouse brain slices, this volumetric method successfully visualized spontaneous $Ca^{2+}$ transients and evoked ones at 7.5 Hz temporal resolution. Due to the simplicity of the device, the method can be applied broadly for 3D imaging.

### [3P09-04]
**3D microelectrode array with optical stimulation for modulating neural network dynamics in the monkey motor cortex during a reaching task**

*Hidenori Watanabe[1], Kazutaka Takahashi[2], Kenta Kobayashi[3,4], Nicholas Hatsopoulos[2], Hajime Mushiake[1] ([1] *Department of Physiology, Graduate School of Medicine, Tohoku University,* [2] *Department of Organismal Biology and Anatomy, The University of Chicago,* [3] *Section of Viral Vector Development, National Institute for Physiological Sciences,* [4] *The Graduate University for Advanced Studies (SOKENDAI)*)

Investigation about modulation of neural activities by stimulation is crucial for decoding the functional connections among the neural activities and understanding those physiological meaning. Integration of optogenetics with microelectrode array has capable for monitoring spatiotemporal evolution of functional neural network in real time by modulating the neural activates after optical stimulation. Here we present three-dimensional (3D) microelectrode array integrated with optical fiber to characterize cortical depthdependent profiles of spatiotemporal neural activities in the monkey motor cortex. The monkey was trained to control a cursor onto a screen to use a single arm to perform a reaching. The monkey kept the cursor at resting targets for at least 3.4 seconds as an awake resting state before target-cue randomly indicating one of the three positions, then reached to the target. A 128-channel electrode arrays with optic fiber in the center (Matrix Array™, NeuroNexus, US) were chronically implanted in the M1 forelimb region after AAV vectors carrying CAG-hChR2 (H134R)/EYFP were injected into the same region. The array consisted of an ECoG grid (32 channels), a 3D intracortical local filed potential (LFP) probes (96 channels), and the optic fiber protruded 0.8 mm from the platform. The optical stimuli were applied during the resting states. Optical stimulation-induced evoked potentials appeared in 35% of the channels. Current source analysis revealed in current-sinks at shallower depths followed by ones at deeper depths in a closest probe from the optic fiber after the stimuli. β frequency oscillation with a prominent peak at 24 Hz appeared in the awake resting states across channels. Phase locking (PL) in β oscillation across the trials disappeared in 35% of the LFP channels, in which were mainly located surround the optical fiber. There were 10% of the LFP channels that was induced PL by the optical stimulation. They were in distal locations apart from the optical fiber. Our array was available for probing spatiotemporal dynamics of neural activities by modulating neural oscillations in the primate cortical 3D networks.

### [3P09-05]
**Volumetric $Ca^{2+}$ imaging in living mouse brain utilizing multipoint scanning two-photon microscopy**

*Mitsutoshi Ataka[1,2,3], Kohei Otomo[2,3,4], Ryosuke Enoki[1,2,3], Tomomi Nemoto[1,2,3] ([1] *School of Life Science, SOKENDAI, the Graduate University for Advanced Studies,* [2] *Exploratory Research Center on Life and Living Systems, National Institutes of Natural Sciences,* [3] *National Institute for Physiological Science, National Institutes of Natural Sciences,* [4] *Graduate School of Medicine, Juntendo University*)

Recently various volumetric imaging techniques have been devised for visualizing the neuronal populations distributed over large volumes simultaneously. In spinning-disk multipoint scanning microscopy, multiple beams of the excitation laser light split by the rotating microlens array simultaneously scan the focal plane. An advantage is that the imaging speed is not limited by increasing the number of pixels or by a low repetition rate of excitation laser pulses, unlike single-point scanning laser microscopy. Recently, we developed a novel two-photon spinning-disk confocal microscopy (Otomo *et al.*, 2015), achieving *in vivo* multi-plane $Ca^{2+}$ imaging in the mouse brain (Ataka *et al.*, 2019). This study introduced a continuous axial scanning mechanism into our system by utilizing an electrically tunable liquid lens (ETL). 4D (*xyz-t*) images were reconstructed based on the axial projection range corresponding to each *xy-t* frame. Then we observed spontaneous activity in the visual cortex using a red $Ca^{2+}$ indicator (Cal-590 AM). As a result, we successfully observed $Ca^{2+}$ activity in ~200 x 200 x 35 $\mu m^3$ FOV as 9 segments along z-axis up to 120 μm depth at 1.5 volume/sec. The developed method might visualize activities of individual neurons across cortical layer 2/3.

### [3P09-06]
### In-house manufacture of an inexpensive large field-of-view two-photon microscope

*Riichiro Hira[1], Yuji Yamauchi[1], Yoshikazu Isomura[1] ([1]*Tokyo medical and dental university*)

Two-photon microscope has been used to observe the deep structures of scattering biological tissues such as the brain, but its production cost has been very high. There have been several problems in its widespread use. One is the light source, a femtosecond pulsed laser in the near-infrared, for which the only option has been the expensive Ti:sapphire laser. Another is the manufacturing cost of the optical system, including high-quality objective lenses for two-photon excitation, associated tube lenses, and relay lenses for near-infrared light. Here, we fabricated an open-source two-photon microscope Trepan2p (Stirman et al. 2016) by using a relatively inexpensive fixed-wavelength fiber laser ALOCR920 ($<$100 fs, 2 W) and assembling the individual lenses in-house to achieve a field of view of 3.5 mm. The lens subassemblies (relay lenses and objective lenses) were made with an accuracy of less than 0.1 mm by using telecentric lenses to precisely measure the distance between the individual lenses as they were fixed to the tube. These subassemblies were incorporated into the cage system, and the entire optical pathway was constructed by properly positioning the scanners (galvano scanner, resonance scanner, and steering mirror) in the cube so that they could be reassembled individually. This allowed us to construct a focal point under the objective lens. This study shows that a two-photon microscope with a large field-of-view can be fabricated at a relatively low cost, which will contribute to its widespread use in the field of physiology as a method to visualize a wide range of biological tissues.

### [3P09-07]
### Functional analysis of a kidney-on-a-chip using human renal proximal tubular epithelial cells and human umbilical vein endothelial cells

*Yin Liang[1], Mengxue Wang[1], Yun Liu[1], Keiji Naruse[1], Ken Takahashi[1] ([1]*Department of Cardiovascular Physiology, Graduate School of Medicine, Dentistry and Pharmaceutical Sciences, Okayama University*)

The kidney is an important organ that controls the reabsorption and excretion of substances such as glucose, urea nitrogen, and creatinine. To develop a model of human kidney, we used human renal proximal tubular epithelial cells (RPTECs) and human umbilical vein endothelial cells (HUVECs) to recapitulate the renal proximal tubule of the nephron. RPTECs and HUVECs were seeded on a two-channel microfluidic chip connected to a peristaltic pump. To evaluate the barrier integrity of the cell layers, we measured permeability using Texas Red-conjugated dextran (MW: 3000). The apparent permeability of the RPTEC-HUVEC bilayer was ~2 × $10^{7}$ cm/s, suggesting sufficient barrier integrity. Next, we performed functional analysis of the kidney chip using urea nitrogen as an index. The concentration of urea nitrogen was ~0.7 mg/dl in the RPTEC channel, while it was ~0.3 mg/dl in the HUVEC channel, suggesting transport of urea nitrogen from the microvascular endothelial side to the kidney epithelial tubular side was active. However, the concentration of glucose was ~110 mg/dl in the RPTEC channel, while it was ~110 mg/ dl in the HUVEC channel, suggesting no significant transport of glucose. These results suggest that medium composition should be optimized to assess the renal function of the kidney-on-a-chip.

### [3P09-08]
### DIDS-sensitive ionic currents in human odontoblasts

*Yoshiaki Furusawa[1,2], Eri Kitayama[1,2], Ryo Nakajima1[2], Maki Kimura[2], Takehito Ouchi[2], Yoshiyuki Shibukawa[2], Masahiro Furusawa[1] ([1]*Tokyo Dental College Department of Endodontics,* [2]*Tokyo Dental College Department of Physiology*)

**Purpose:** We have previously reported expressions of various cation channels, such as voltage-dependent $Na^{+}$ channels, $Ca^{2+}$-activated $K^{+}$ channels, and voltage-dependent $K^{+}$ channels, as well as anion channels such as DIDS- and SITS-sensitive $Cl^{-}$ channels in odontoblasts. Potassium conductance contributes to the maintenance of the resting membrane potential of rat odontoblasts in association with partial contribution of Clconductances. The aim of this study is to investigate the detailed expression patterns and properties of anion transporters including $Cl^{-}$ channels in human odontoblasts (HOB cells).
**Materials & Methods:** We measured ionic currents using a whole-cell patch-clamp recording with gramicidin paerforated-mode. Krebs solution was used as a standard extracellular solution (ECS). Standard intracellular solution (ICS) was composed as following (in mM) ; 140 KCl, 10 NaCl and 10 HEPES (pH7.2). We prepared $Ca^{2+}$-free ECS solution by removing extracellular $Ca^{2+}$ (as 0 mM) from standard ECS. Additionally, we used a 4,4'-diisothiocyanatostilbene-2,2'-stilbenedisulfonic acid (DIDS, Sigma) as a $Cl^{-}$channel blocker.
**Results & Conclusion:** Application of voltage ramp protocol from a holding potential (Vh) of -70 mV with voltage ranging from -100mV to +100mV (0.04mV/ms) elicited inward and outward currents. When we removed $Ca^{2+}$ from the extracellular solution, the amplitudes of outward current was reduced in the membrane potential over 0 mV toward depolarizing potential. The outward current was also inhibited by 100 $\mu$M DIDS at voltage ranging from -20 mV to +100 mV, suggesting that odontoblasts express DIDS-sensitive $Cl^{-}$ channels.

### [3P09-09]
### Piezo1-Yap signaling regulates human cementoblast mineralization and proliferation

*Toshihiro Hasegawa[1,2,3], Takehiro Ouchi[1], Maki Kimura[1], Yoshiyuki Shibukawa[1], Taneaki Nakagawa[2] ([1]*Department of Physiology, Tokyo Dental College,,* [2]*Department of Dentistry and Oral Surgery, Keio University School of Medicine,* [3]*Department of Oral and Maxillofacial Surgery, Tokyo Metropolitan Cancer and Infectious Diseases Center Komagome Hospital*)

Cementoblasts have essential roles in secretion of both non-collagenous and collagenous matrix proteins to produce cementum. The cementum is a mineralized tissue having deposited-layers on the surface of tooth root, and is always loaded by mechanical stress such as chewing during lifetime. However, the detailed mechanosensitive mechanisms of cementoblasts are still remain to be clarified. To address those, we conducted immunofluorescence studies on human cementoblasts (HCEM). We also performed mineralization assay and colony forming unit fibroblast (CFU-F) assay to observe the mineralization and proliferation abilities, respectively, of HCEM. HCEM were immunopositive for mechanosensitive ion channel, Piezo1, and mechanotransducer, Yap. Yap was positive within nuclear location. When Piezo1 channels in HCEM were knocked down by gene silencing, Yap expression was translocated to extranuclear space. Mineralization efficiencies were impaired by Piezo1 silencing in HCEM, compared to the cells without Piezo1-knocking down. Piezo1 inhibitor, GsMTx4, also induced impairment of mineralization. CFU-F assay revealed that pharmacological inhibitors of Piezo1 activated proliferation in HCEM, compared to the cells without any application of Piezo1 inhibitors. These data suggest that Piezo1 upregulates mineralization, but downregulates proliferation of cementoblasts through Piezo1-Yap signaling.

### [3P09-10]
### Piezo1 channel activation increases $Ca^{2+}$ influx via TRPV1 channel in rat odontoblasts

*Ryuya Kurashima[1], Maki Kimura[2], Takehito Ouchi[2], Yoshiyuki Shibukawa [2] ([1]*Tokyo Dental Coll. Suidobashi Hospital,* [2]*Dept. Physiol. Tokyo Dental Coll.* )

We have previously reported that direct mechanical stimulation activated the transient receptor potential (TRP) channel subfamilies (TRPV1, TRPV2, TRPV4 and TRPA1) and mechanosensitive ion channel, Piezo1 channels. Activation of these channels increased the intracellular free $Ca^{2+}$ concentration ($[Ca^{2+}]_i$) in odontoblasts, which are dentin forming and mechano-sensory receptor cells. In endothelial cells and pancreatic acini cells, it has been reported that Piezo1 channel regulates mechanosensitive TRPV4 channel activation. However, the detailed mechanism of such crosstalk among TRP channel and Piezo1 channel in odontoblasts remain to be clarified. In this study, we investigated $Ca^{2+}$ signaling to clarify the crosstalking machinery between TRPV1 and Piezo1 channels in acutely isolated rat odontoblasts by measuring $[Ca^{2+}]_i$ using fura-2. In the presence of extracellular $Ca^{2+}$, application of Yoda1 (1.5 min in duration), a pharmacological Piezo1 channel activator, increased $[Ca^{2+}]_i$ showing biphasic responses. Simultaneous application of Dooku1, that antagonizes Yoda1-evoked activation of Piezo1 channel, and Yoda1 did not affect on the first phase of the Yoda1-induced increases, but significantly showed inhibitory effects on the second phase of them. Furthermore, sustained application of Yoda1 (10 min in duration) induced persistent increases in $[Ca^{2+}]_i$, which were inhibited by A784168, TRPV1 channel antagonist. These results suggested functional Piezo1 and TRPV1 channel crosstalk in odontoblasts.
(COI:NO )

### [3P09-11]
### Somatosensory and gustatory input differences between the hemodynamics of major salivary glands in rats

*Toshiya Sato[1], Yunosuke Okada[1], Ratna Ramadhani[1], Hisayoshi Ishii[1] ([1]*Div. of Physiol., Dept. of Oral Biol., Sch. of Dent., Health Sci. Univ. Hokkaido*)

We have demonstrated that trigeminal sensory stimulus induces marked increases in blood flow (BF) in the major salivary gland (MSG) mediated by parasympathetic nerves (PN) as well as salivation. This supports the importance of PN activation linked with orofacial sensory input in salivation and glandular hemodynamics. Mechanical input from the oral cavity is known to increase the rate of salivation in the parotid gland (PG), and gustatory input on the tongue in rats promotes salivation in the submandibular gland (SMG) rather than PG. It is speculated that the glandular hemodynamics are also regulated according to differences in sensory input. To clarify this relationship between stimulus types and glandular parasympathetic BF increase, we analyzed the hemodynamics of the MSG during electrical stimulation of the inferior alveolar nerve (IAN; somatosensory input) or taste nerve (TN; gustatory input) in urethane-anesthetized rats. IAN stimulus induced frequency-dependent BF increases in the PG and SMG, and the increases in the PG were significantly higher than those in the SMG. The TN stimulus induced frequency-dependent BF increase in the SMG, but no significant BF increase was observed in the PG. Therefore, our results indicate that there is a difference in parasympathetic BF increase in the MSG depending on the type of sensory input, which suggests that this difference is important for the variation in relative secretion ratios of salivary glands.

### Poster Presentation 3

### [3P10]
### Oral physiology

March 18(Fri), 12:00 - 14:00, Zoom P10

### [3P10-01]
**Activation of adenylyl cyclase induced intracellular cAMP increase and $Ca^{2+}$ influx in odontoblasts**

*Sachie Nomura[1], Maki Kimura[1], Takehito Ouchi[1], Hidetaka Kuroda[2,1], Kyosuke Kono[1], Masayuki Ando[1], Yoshiyuki Shibukawa[1] ([1]*Dept. Physiol., Tokyo Dental Coll.*, [2]*Dept. Dental Anesthesiol., Kanagawa Dental Univ.*)

Intracellular $Ca^{2+}$ signaling in odontoblasts participates in tertiary dentin formation and generation of dentinal pain. In this study, we examined the crosstalk between intracellular cyclic AMP (cAMP) and $Ca^{2+}$ signaling in odontoblasts by measuring intracellular cAMP levels and $Ca^{2+}$ concentrations ($[Ca^{2+}]_i$). In the presence of extracellular $Ca^{2+}$, forskolin (FSK), an adenylyl cyclase (AC) activator, or isoproterenol (ISO), an agonist of the Gs protein-coupled beta-2 adrenergic receptors, dose-dependently increased intracellular cAMP levels. The increases were inhibited by an AC inhibitor. FSK or ISO increased intracellular cAMP levels and $[Ca^{2+}]_i$. In the absence of extracellular $Ca^{2+}$, FSK or ISO increased intracellular cAMP levels, but not $[Ca^{2+}]_i$. In the presence of extracellular $Ca^{2+}$, FSK-induced $[Ca^{2+}]_i$ increase was not sensitive to TRPV1, TRPV4, Piezo1, nonselective $Ca^{2+}$, and cyclic nucleotide-gated (CNG) channel antagonists, and a $Na^+$-$Ca^{2+}$ exchanger (NCX) inhibitor. These results suggested that AC activation induced increase in intracellular cAMP levels and $Ca^{2+}$ influx from extracellular medium in odontoblasts. In addition, the $Ca^{2+}$ influx is not $[Ca^{2+}]_i$ increase via TRPV1, TRPV4, Piezo1, and CNG channels, and reverse mode of NCX in odontoblasts.
(COI: No.)

### [3P10-02]
**Sex difference of neuropathic pain mechanism in the trigeminal nervous system in mice**

*Yoshiyuki Tsuboi[1] ([1]*Nihon Univ.*)

The purpose of this research was to clarify the sex difference of neuropathic pain mechanism in the trigeminal nervous system. ICR mice were subjected to neuropathy that pressured the left lingual nerve with 30 g of force for 30 seconds under isoflurane inhalation anesthesia. The sham group was made to have an incision but not to press. Under light anesthesia, withdrawal thresholds (WT) were measured by stimulating with a pincher (Bioseb) and thermal stimulator (Intercross). On the third day after surgery, Minocycline (Mino) or Pioglitazone (Pio) was injected into Cisterna Magna and the WT were measured. Then, we obtained the following results. In males and females, the WT were significantly lower than in the sham group on the third day after surgery. In the males, the lowering of the WT was significantly suppressed in the Mino administration group. The lowering of the WT was significantly suppressed in the female Group in the Pio administration group. These results suggest that there is a gender difference in the neuropathic pain mechanism in the trigeminal nervous system.

### [3P10-03]
**Physiological responses associated with sweet taste transduction in humans is a hyperpolarizing potential on the lingual epithelium**

*Satoshi Fukuda[1], Naoyuki Murabe[1], Haruno Mizuta[2], Takashi Yamamoto[2], Takatoshi Nagai[1,3] ([1]*Dept. of Physiol., Teikyo Univ. Sch. of Medicine*, [2]*Dept. of Nutrition, Faculty of Health Sciences, Kio Univ.*, [3]*Emeritus Professor of Keio Univ.*)

The lingual surface potential (LSP), which hyperpolarizes in response to salt and bitter stimuli, is thought to be a bioelectrical signal associated with taste transduction in humans. In contrast, a recent study reported sweet and sour stimuli to evoke a depolarization of the LSP. We questioned the origin of such a depolarization because liquid junction potentials (JPs), which arise at the interfaces of recording electrode and taste solutions, are neglected in the report. We recorded the LSPs to sucrose on the human tongue using an Ag/AgCl electrode. To estimate JPs generated by each taste solution, we made an agar model to simulate the human tongue. The lingual surface was rinsed with a 10 mM NaCl solution that mimics the sodium content of the lingual fluid. In the human tongue, sucrose dissolved in distilled water evoked a depolarizing LSP that could be attributed to JPs, resulting from the change in electrolyte concentration of the taste solution. Sucrose dissolved in 10 mM NaCl solution evoked a hyperpolarizing LSP which became more negative in a concentration-dependent manner (300-1500 mM). Lactisole (3.75 mM), an inhibitor of sweet taste, significantly reduced the LSPs and decreased perceived intensity of sweetness by human subjects. When the electrolyte environment on the lingual surface is controlled for JPs, the bioelectrical signal associated with sweet taste transduction is a hyperpolarizing potential.

### [3P10-04]
**The effect of dexamethasone on the cisplatin-induced CTA in rats with area postrema lesions and bilateral subdiaphragmatic afferent vagotomy.**

*Shaoyi Su[1], Zimo Wei[1], Helai Huang[1], Tomohiko Yoshizawa[1], Tadashi Inui[1], Makoto Funahashi[1] ([1]*Hokkaido Univ.*)

Cisplatin-induced emesis can be suppressed by vagotomy and area postrema lesions in other species, such as dogs, cats, ferrets, and shrews. However, the induction mechanism of nausea is still unclear. We investigated cisplatin-induced conditioned taste aversion (CAT) in rats to demonstrate the central mechanism of nausea. Male Sprague-Dawley rats (6-7 weeks old) were divided into 2 groups: 1) a group of intact rats, 2) a group of rats with area postrema lesions (APX), and the bilateral subdiaphragmatic vagotomy (VX). We measured CTA to 0.1 % saccharin solution with a single administration of cisplatin (3mg/ kg, i.p., 1% BW) or dexamethasone (1mg/kg, i.p.,0.1%BW), and administration of cisplatin after dexamethasone injection. The intact group showed a significant reduction of saccharin intake after conditioning with cisplatin (n=10), and pretreatment of dexamethasone failed to block the CTA acquisition (n=5). Although cisplatin-induced CTA was still produced in rats with APX and VX(n=6), pre-treatment with dexamethasone significantly suppressed cisplatin-induced CTA on test days 1 and 2 (n = 6). We found that rats with APX and VX can acquire the cisplatin-induced CAT, suggesting the different induction mechanism between nausea and vomiting. These results suggest that the involvement of higher brain regions other than the area postrema in which dexamethasone acts.
The authors declare no conflict of interest associated with this manuscript.

### [3P10-05]
**Development of a sensor array for noninvasive measurement of the total length of the laryngeal movement during swallowing**

*Makito Iizuka[1], Takahiko Mori[2], Kazuhide Tomita[3], Naohiko Watanabe[4], Reiko Takeshima[5], Masahiko Izumizaki[1] ([1]*Dept Physiol, Showa Univ Sch Med*, [2]*Dept Electrical and Electronic Engineering, Shonan Inst Tech*, [3]*Dept Phys Ther, Ibaraki Pref Univ Hlth Sci*, [4]*Dept Civil Engineering, NIT, Gifu College*, [5]*Ctr Med Sci, Ibaraki Pref Univ Hlth Sci*)

During swallowing, the larynx rises adequately and closes trachea tube with epiglottis. Previously, we developed a piezo sensor array that was able to detect the laryngeal movement noninvasively (Iizuka et al, J Physiol Sci, 68: 837-846, 2018). However, the total length of the laryngeal movement was not able to obtain due to its small number of sensors (5 sensors, 3 mm interval). Here, we increased the number of sensors to 25, and lined these sensors with 3.0 mm interval accurately, and embedded in the middle of polyurethane gel sheet (length 88 mm, width 80 mm, thickness 5mm). The sensor sheet was lightly attached to the ventral surface of the neck near the laryngeal prominence. Then the subject was instructed to swallow 3 ml water ten times. The sensor on the predicted initial position of the laryngeal prominence showed trough at the beginning of the swallowing. The sensors locating where the laryngeal prominence should cross have showed two peaks. In these sensors, the sensors at higher position showed smaller interval between the first and second peak. Thus, the first peak should correspond to the time the laryngeal prominence going up at the sensor, and the second peak should correspond to the time the laryngeal prominence going down at the sensor. We assumed the laryngeal prominence reached at the position of the sensor which was rostrally adjacent to the highest sensor that showed two peaks. In conclusion, we succeeded to develop a new sensor array to measure the total length of the laryngeal movement noninvasively at the special resolution of 3.0 mm, and this array will be useful to evaluate the swallowing function.
(COI: No.)

### [3P10-06]

### Association between the Gly16Arg polymorphism of human beta2-adrenergic receptor gene and food preferences

*Kohei Narita[1], Tada-aki Kudo[1], Guang Hong[2], Kanako Tominami[1], Satoshi Izumi[1], Yohei Hayashi[3,4], Junichi Nakai[1] ([1]*Division of Oral Physiology, Tohoku University Graduate School of Dentistry,* [2]*Division for Globalization Initiative, Liaison Center for Innovative Dentistry, Tohoku University Graduate School of Dentistry,* [3]*Cell Resource Center for Biomedical Research, Institute of Development, Aging and Cancer, Tohoku University,* [4]*Graduate School of Life Sciences, Tohoku University*)

The Gly16Arg polymorphism with a G to C single nucleotide mutation in the human beta2-adrenergic receptor (*ADRB2*) gene might have a relationship with obesity. However, the substitution's effects on food preference (FP) are mostly unknown; thus, we tested this among healthy young adults (mean age, 23.4; $n$=52). For the FP evaluation, preferences for four food types (sweet, salty, sour, and bitter) and high-fat foods were scored with a self-reported questionnaire. The polymorphism was genotyped and both male ($n$=28) and female subjects ($n$=28) were further divided into three groups (the subjects with genotypes of homozygote (GG, CC) and heterozygote, GC). Sour FP of GG group was higher than CC group in female (p < 0.05). When sweet foods were divided into high-fat (HF) and low-fat (LF) subgroups, FP for HF sweet foods in the GG group was higher than that for LF sweet foods in both male and female subjects ($p$< 0.05). The HF-FP degree in the GG group was higher than other groups in male ($p$< 0.05). These results suggest that the polymorphism might be related with FP. Thus, to reveal the relation of the *ADRB2* substitution to FP will be valuable for tailor-made obesity prevention.

### [3P10-07]

### Expression of ACE2, TMPRSS2 and neuropilin-1 in rat geniculate and trigeminal ganglia.

*Takeshi Suwabe[1], Toshiaki Yasuo[1], Noritaka Sako[1], Fumihiko Nakamura[1] ([1]*Department of Oral Physiology, Division of Oral Functional Sciences and Rehabilitation, School of Dentistry, Asahi University*)

Severe acute respiratory syndrome coronavirus 2 (SARS-CoV-2) is a neurotropic virus with the capacity to infect and replicate in neuronal cells. SARS-CoV-2 binds to the SARSCoV receptor angiotensin converting enzyme 2 (ACE2) for host cell entry. Fusion of the viral membrane with host cell membrane is induced by the transmembrane serine protease 2 (TMPRSS2). Neuropilin-1 (NRP1) is a coreceptor for host cell entry of SARS-CoV-2. In this study, to examine the possibility of SARS-CoV-2 entry into cranial sensory ganglia innervating the oral cavity, ACE2, TMPRSS2 and NRP1 in the geniculate and trigeminal ganglia of rats. Geniculate and trigeminal ganglia were collected from anesthetized rats, total RNA was extracted from the ganglia, and cDNA was synthesized from the RNA template by reverse transcription. Gene expression levels were determined by real-time PCR. ACE2, TMPRSS2 and NRP1 genes were expressed in the ganglia. This result suggests that SARS-CoV-2 may invade sensory neurons of the ganglia. It also suggests that the downregulation of ACE2 associated with the binding of SARS-CoV-2 to ACE2 may affect the activity of the sensory neurons.

### [3P10-08]

### The effect of Temperature-Controlled Repeated Thermal Stimulation on osteoblast differentiation in MC3T3-E1 Cells

*Kanako Tominami[1], Tada-aki Kudo[1], Guang Hong[2], You-Ran Luo[2], Satoshi Izumi[1], Yohei Hayashi[3,4], Takuya Noguchi[5], Atsushi Matsuzawa[5], Junichi Nakai[1] ([1]*Division of Oral Physiology, Tohoku University Graduate School of Dentistry,* [2]*Division for Globalization Initiative, Tohoku University Graduate School of Dentistry,* [3]*Cell Resource Center for Biomedical Research, Institute of Development, Aging and Cancer, Tohoku University,* [4]*Graduate School of Life Sciences, Tohoku University,* [5]*Laboratory of Health Chemistry, Graduate School of Pharmaceutical Sciences, Tohoku University*)

Osteoblasts are crucially important for bone remodeling processes. This study was aimed to examine the effects of temperature-controlled repeated thermal stimulation (TRTS) on the osteoblast differentiation in murine pre-osteoblastic MC3T3-E1 cells. A heating plate was used to apply thermal stimulation, and the correlation of culture-medium temperature with the surface temperature of the heating plate was monitored. Plated MC3T3-E1 cells were exposed to TRTS at three different temperatures via the heating plate (preset surface temperature of the heating plate, 39.5°C, 42°C and 45°C; the corresponding medium temperatures, 38.4°C, 40.3°C and 42.7°C, respectively) in growth or differentiating medium for up to 18 h/day. Alkaline phosphatase (ALP) activity assay and alizarin red staining assay were conducted to assess the osteoblastic differentiation. While the TRTS at 39.5°C did not decrease the growth rate of cells up to 18 h/day, it was revealed that the TRTS under the above condition caused increased ALP activity and enhanced mineralization. These results suggest that TRTS can induce the osteoblast differentiation in the cells and might offer an effective technique for bone regeneration.

### [3P10-09]

### Physiological and morphological characterization of Phox2b-expressing neurons in the rat rostral parvocellular reticular formation

*Risa Kajiwara[1,2], Shiro Nakamura[2], Keiko Ikeda[2], Hiroshi Onimaru[3], Atsushi Yoshida[4], Yumi Tsutsumi[4], Kiyomi Nakayama[2], Ayako Mochizuki[2], Masanori Dantsuji[2], Takehiko Iijima[1], Tomio Inoue[2] ([1]*Department of Perioperative Medicine, Division of Anesthesiology, Showa University School of Dentistry,* [2]*Department of Oral Physiology, Showa University School of Dentistry ,* [3]*Department of Physiology, Showa University School of Medicine,* [4]*Department of Oral Anatomy and Neurobiology*)

Neurons in the parvocellular reticular formation (PCRt) of the medulla play a crucial role in a variety of oromotor functions, including suckling, chewing, and swallowing. Recent studies have shown that neurons expressing the transcription factor Phox2b are distributed in the PCRt. In this study, we examined the properties of Phox2b$^+$ neurons in the rostral PCRt (rPCRt) using postnatal day (P)2-11 Phox2b-EYFP rats. In situ hybridization showed that the majority of Phox2b$^+$ rPCRt neurons were glutamatergic, but not GABAergic or glycinergic. Whole-cell recording revealed that more than half of Phox2b$^+$ rPCRt neurons showed low-frequency firing, and approximately 20% were spontaneously active. The postsynaptic currents (PSCs) were observed in approximately half of the Phox2b$^+$ rPCRt neurons in response to stimulation of the supratrigeminal nucleus. Some Phox2b$^+$ rPCRt neurons sent their axons to the trigeminal motor nucleus. We then tested the connectivity from Phox2b$^+$ rPCRt neurons to parasympathetic neurons in the superior salivatory nucleus (SSN), which regulated salivation using P7-11 Phox2b-ChRFR rats. We found that the PSCs were evoked in parasympathetic SSN neurons responding to photostimulation of the rPCRt. Phox2b$^+$ rPCRt neurons may be related to multiple oral functions, such as jaw movement and salivary secretion.

### [3P10-10]

### Sustained stimulation saliva from minor salivary glands fluctuates diurnally and secretion is reduced by smoking

*Akira Furuyama[1] ([1]*Department of Oral Function and Molecular Biology, Ohu University School of Dentistry*)

It has been reported that saliva secretion continues to increase for more than 15 minutes after umami stimulation. There have been no studies examining the effects of circadian rhythms and smoking on this sustained stimulation saliva from minor salivary glands. To clarify whether salivary secretion in the lower lip gland, which is promoted at rest, immediately after umami stimulation, and continuously after umami stimulation, (1) shows diurnal variation and (2) is affected by chronic smoking. There was no significant difference in resting saliva between morning and afternoon and the results were the same as previous studies (Wang et al. 2015). There was no significant difference between smokers and non-smokers as well. There was no significant difference between smokers and nonsmokers in saliva secretion immediately after stimulation (2 minutes after stimulation), which is generally regarded as stimulating saliva, in the morning and afternoon. However, there was a significant difference in saliva secretion between morning and afternoon, and between smokers and non-smokers in the case of continuous stimulating saliva that persisted after umami stimulation (more than 14 minutes). In the continuous stimulated saliva, the difference was significant between smokers and non-smokers. It is suggested that continuous stimulated saliva produced by umami stimulation may reflect changes in physiological state more sensitively than conventional resting saliva or stimulated saliva.

### [3P10-11]

### Eugenol activates outward currents in human odontoblasts

*Ryo Nakajima[1,2], Eri Kitayama[1,2], Yoshiaki Furusawa[1,2], Maki Kimura[2], Takehito Ouchi[2], Yoshiyuki Shibukawa[2], Masahiro Furusawa[1] ([1]*Tokyo Dental College Department of Endodontics,* [2]*Tokyo Dental College Department of Physiology*)

**Purpose:** Odontoblasts are tall columnar cells which are derived from the cranial neural crest and located at the border between dentin and dental pulp. Odontoblast plays pivotal roles in sensory transduction sequence for generation of dentinal pain following dentin stimuli as a sensory receptor cell. In addition, the odontoblast also plays important roles in physiological and tertiary dentin (reparative and reactionary dentin) formation. Eugenol, which is an allyl chain-substituted guaiacol, is widely used as a drug for analgesic, anti-inflammatory and antiseptic effects on the dental pulp inflammation in the clinical settings of dentistry. We have previously reported expression of various subtypes of transient receptor potential (TRP) channels in odontoblasts. Although eugenol is known as an activator on the TRPV1 channel, detailed mechanisms of eugenol in activation of it in odontoblasts remain to be clarified. We thus aimed to investigate the pharmacological effects of eugenol on the whole-cell ionic currents recorded from human odontoblasts.

**Methods:** Ionic currents were recorded from human odontoblast cell line (HOB cells) using a conventional-mode of whole-cell patch-clamp method. The Krebs solution (pH 7.4) was used as a standard extracellular solution (standard ECS), and the solution consisted by 140 mM KCl, 10 mM NaCl, and 10 mM HEPES (pH 7.2) was used as the standard intracellular solution (standard ICS).

**Results and discussion:** At a holding potential (Vh) of -70 mV with the standard ECS/ICS, we observed an outward rectifying current activated by depolarizing voltage-ramp from -100 mV to +100 mV. Administration of 10 mM eugenol reversibly increased the amplitudes of the outward currents at positive membrane potential, suggesting that eugenol is capable to activate ligandgated ionic channels in odontoblasts.

### Poster Presentation 3

### [3P11]
### Undergaraduate students

March 18(Fri), 12:00 - 14:00, Zoom P11

### [3P11-01]
### Physiological analysis of doxorubicin-induced cardiotoxicity in H9C2 cells

*Fumina Suzuki[1], Masanari Umemura[1], Hiroko Nemoto[1,2], Megumi Uchino[1], Akane Nagasako[1], Yuko Hidaka[1], Yoshihiro Ishikawa[1] ([1]*Cardiovascular Research Institute (CVRI), Yokohama City University Graduate School of Medicine,* [2]*Department of Surgery, Yokohama City University Graduate School of Medicine*)

**Introduction**
Doxorubicin (DOX) is used as a broad-spectrum anti-tumor anthracycline to treat various cancers in the world. The serious adverse effects of DOX on cardiotoxicity restrict its usage in clinical. Several reports investigated that calcium overload is one of the factors of DOX-induced cardiotoxicity in cardiomyocytes. Therefore, we focus on the relationship between DOX and $Ca^{2+}$ homeostasis, store-operate calcium entry (SOCE) which is a major pathway for calcium signaling in particular.
**Material and Methods**
H9c2 cell, which is derived from embryonic rat cardiomyocytes was used as an alternative for cardiomyocytes. XTT assay and xCELLigence real-time cell analysis (RTCA) system were performed to evaluate the cell viability. Western blotting (WB) analysis was performed to evaluate the protein expression and phosphorylation. YM-58483, store-operated $Ca^{2+}$ entry (SOCE) inhibitor was used to inhibit calcium influx from extracellular space.
**Results**
To evaluate cell proliferation, we performed XTT assay and RTCA. Both of these experiments showed that DOX decreased cell proliferation in dose-dependent manner 24 hours after the stimulation in H9C2. WB analysis showed that DOX increased the protein expression of cleaved caspase-3, p53 and signal transducer and activator of transcription 3(STAT3), which are related to apoptosis. YM58483 negated DOX-induced p53 phosphorylation in H9C2.
**Conclusion**
Our results showed that DOX suppressed the cell proliferation and induced the apoptosis in H9C2. YM58983 may be a candidate treatment to prevent DOX-induced cardiotoxicity in clinical for the future.

### [3P11-02]
### Distinct subtypes of the mouse lateral amygdala neurons in fear memory formation

*Sota Matsumura[1], Mieko Morishima[1], Suguru Tohyama[1], Ayako Watabe[1] ([1]*Institute of Clinical Medicine and Research, Research Center for Medical Sciences, The Jikei University School of Medicine*)

The lateral amygdala (LA) plays a crucial role in fear learning. The information about a conditioned stimulus (CS) and an unconditioned stimulus (US) converge in the LA. Following the pairing of CS and US, CS acquires the ability to elicit conditioned responses. The auditory fear conditioning is known to induce synaptic plasticity in the excitatory neurons in LA. However, little is known how the neurons are modulated by the input from the auditory thalamus to the LA pathway conveying the CS. To address this question, we performed whole-cell patch-clamp recording with the brain slices from the fear-conditioned mice, and applied optogenetic manipulation of that pathway. We identified excitatory neurons in the LA based on the soma size and the firing patterns, and found the distinct subtypes of excitatory neurons with a wide range of input resistance. Then, we examined the synaptic plasticity of those neurons, and found some changes in the feed-forward inhibitory response between the fear-conditioned and control mice. These results suggest that some subtypes of LA excitatory neurons might be involved in fear learning.

### [3P11-03]
### Glutaminergic stimulation of the dorsomedial hypothalamus enhances colorectal motility by activating spinal defecation center via the medullary raphe and the A11 nucleus in rats.

*Natsufu Yuki[1], Kazuhiro Horii[2,3], Tomoya Sawamura[1], Takahiko Shiina[1,2], Yasutake Shimizu[1,2] ([1]*Laboratory of Veterinary Physiology, Faculty of Applied Biological Sciences, Gifu University,* [2]*Department of Basic Veterinary Science, Laboratory of Physiology, United Graduate School of Veterinary Sciences, Gifu University,* [3]*Division of Biological Principles, Department of Physiology, Graduate School of Medicine, Gifu University*)

The mechanism by which acute stress elicits defecation reflex is unknown. It has been reported that a glutamatergic pathway from the prefrontal cortical area to the dorsomedial hypothalamus (DMH) is related to thermogenic and cardiovascular responses to stress. The purpose of this study was to verify whether DMH is also involved in defecation reflex pathway. Colorectal intraluminal pressure and expelled fluid volume were recorded in anesthetized rats. When glutamate receptor agonists, AMPA and NMDA, were administered to DMH, a marked increase in colorectal motility was observed. Owing to the enhanced colorectal motility in response to DMH stimulation was suppressed by prior administration of serotoninergic and dopaminergic inhibitors into the spinal cord. Considering that descending serotonergic neurons projecting from the medullary raphe nucleus and dopaminergic neurons from the A11 nucleus to the spinal defecation center enhance colorectal motility, our findings suggest that the DMH activation enhances colorectal motility by activating spinal defecation center via the medullary raphe nucleus and the A11 nucleus. This pathway may be related to stress-induced defecation.

### [3P11-04]
### Corticospinal fibers from the somatosensory cortex run in rostrocaudal direction through the lamina III and IV of the spinal cord.

*Rin Iwasawa[1], Naoyuki Murabe[1], Toshihiro Hayashi[1], Masaki Sakurai[1] ([1]*Teikyo university*)

Corticospinal (CS) fibers originate not only from the motor but also from the somatosensory cortices, projecting to the ventral and dorsal spinal gray matter in the transverse plane, respectively. However, little attention has been paid to how the CS fibers project in the longitudinal plane of the spinal gray matter. To investigate the CS projection patterns from the motor and somatosensory cortices in the spinal gray matter, we anterogradely labeled the CS fibers from an each area using adeno-associated virus expressing channel rhodopsin-2 fused with fluorescent proteins and quantitatively analyzed the CS fiber orientation in the gray matter of the lower cervical cord in both transverse and longitudinal planes. We found that the somatosensory CS fibers ran in the lamina III and IV in parallel to the rostro-caudal axis, while the motor CS fibers extended perpendicularly to this axis in the intermediate and ventral zones. A characteristic feature of CS organizations from the somatosensory and motor cortices found in this study would give a novel insight into their underlying functions.

### [3P11-05]
### Projections of the paraventricular thalamic nucleus neurons to the basolateral amygdala during the retrieval of conditioned taste aversion

*Koji Hosoda[1], Tadashi Inui[2], Makoto Funahashi[2] ([1]*School of Dental Medicine, Hokkaido University,* [2]*Department of Oral Physiology, Graduate School of Dental Medicine, Hokkaido University*)

To detect the inputs from central neurons to the basolateral amygdala (BLA) during the retrieval of conditioned taste aversion (CTA), we performed behavioral and histological studies in Fos-eGFP rats. Fos-eGFP rats that had an injection of a retrograde tracer Fluoro- Gold (FG) into the BLA were conditioned with a pairing of intraorally infused 0.1% saccharin solution (0.5 ml/min, 8 min) and an i.p. administration of 0.15 M LiCl (CTA group, n = 4). Some rats were conditioned with i.p. injection of saline instead of LiCl (SHAM group, n = 3). At 3 h after the CTA retrieval test, the rats were perfused with 4 % paraformaldehyde. The rats in the CTA group but not SHAM group showed aversive taste reactivity responsesand GFP-expressing neurons in the paraventricular thalamic nucleus (PVT). FG-labeled cells were also found in the PVT. The GFP expression and the FG labeling overlapped in some neurons. In the SHAM group, a few GFP-expressing neurons were detected in the PVT. As the Fos protein expression is a marker of depolarization of neurons, we suggest that the neuronal pathway from the PVT to the BLA is activated during the CTA retrieval.
(COI: No. )

### [3P11-06]
### Grial origin of epilepsy

*Ichinosuke Onishi[1], Shun Araki[2], Ko Matsui[3] ([1]*School of medicine, Tohoku University,* [2]*Super-network Brain Physiology, Graduate School of Medicine, Tohoku University,* [3]*Super-network Brain Physiology, Graduate School of Life Sciences, Tohoku University*)

The role of astrocytes in seizure generation and in epileptogenesis was studied. Astrocytes are known to have the power to alter the local brain environment, thus, astrocyte activity can lead to neuronal function modulation. Partial seizure is known to originate from a specific region called the epileptogenic focus. Abnormal astrocytic activity at or close to the focus may lead epileptic seizures where a large number of neurons oscillate in a coordinated manner. In our study, epileptic mouse model was created by kainic acid injection or by copper implantation at the hippocampal dentate gyrus. Astrocyte activity was assessed by the fluorescence changes of a calcium sensor protein expressed selectively in astrocytes using in vivo fiber photometry method. Interestingly, a strong astrocytic $Ca^{2+}$ activity was observed prior to the occurrence of spontaneous EEG discharges. No apparent changes in the EEG waveforms accompanied these $Ca^{2+}$ waves. In about a couple of weeks after the surgery, frequent spontaneous epileptic discharges were observed. Surprisingly, large $Ca^{2+}$ waves ceased to occur at this stage. These results suggest that the primal role of astrocytes is not to trigger individual seizure but rather to induce plastic changes of the brain circuit that leads to the generation of epileptogenic focus. New preventative medicine could target glial cells to suppress exacerbation of epilepsy.

### [3P11-07]
### Regulation of migration by CaMKK2 inhibition on EP4-Orai1- $Ca^{2+}$ signaling in oral cancer

*Soichiro Ishikawa[1,2], Masanari Umemura[2], Megumi Uchino[2], Rina Nakakaji[1,2], Akane Nagasako[2], Kohei Osawa[1,2], Rafikul Islam[2], Kenji Mitsudo[1], Yoshihiro Ishikawa[2] ([1]*Department of Oral and Maxillofacial Surgery, Yokohama City University Graduate School of Medicine,* [2]*Cardiovascular Research Institute (CVRI), Yokohama City University Graduate School of Medicine*)

Introduction
Oral cancer accounts for 2-4% of all cancers. The number of patients with oral cancer has been increasing in recent years. Lymph node metastasis caused by the migration of oral cancer cells is one of the important factors that affects the prognosis of life. We previously reported that EP4, one of the receptors for Prostaglandin E2 (PGE2), regulates $Ca^{2+}$ influx from extracellular to intracellular space in oral cancer cells, resulting in regulating cell migration. However, it is not well known that how EP4 regulates the downstream signaling. In the current study, we focus on the mechanism of cell migration via $Ca^{2+}$ signaling, especially calcium/calmodulin-dependent protein kinase kinase 2 (CaMKK2).
Material and Methods
STO-609 was used as CaMKK2 inhibitor. ONO-AE1-437 was used as EP4 agonist. Human oral squamous cell carcinoma cell line, HSC-3 was used. The effect of cell proliferation was evaluated by XTT Assay. The cell migration ability was also evaluated by scratch assay.
Results
EP4 agonist promoted the cell migration of oral cancer cells. STO-609, a CaMKK2 inhibitor, negated the EP4 agonist-induced cell migration in HSC-3 cells. In contrast, EP4 agonist (1 μM or 5 μM) did not suppress the cell proliferation.
Conclusion
Our finding showed EP4 -$Ca^{2+}$-CaMKK2 signaling as a novel EP4 downstream pathway. Our results suggest that EP4 and CaMKK2 are promising candidates as targets for the treatment in oral cancer metastasis.

### [3P11-08]
### Impact of Vitamin B1 contained in cardioplegia on cardioprotection during cardiac arrest

*Kakeru Okawa[1,2], Takahiro Inoue[1], Yoichiro Kusakari[1], Susumu Minamisawa[1] ([1]*Department of Cell Physiology, The Jikei University School of Medicine,* [2]*4th-year medical student*)

Background: We lately reported that Vitamin B1 preserved cardiac function against ischemic injury by maintaining ATP level. However, the impact of Vitamin B1 contained in a cardioplegia during cardiac arrest is still unclear.
Materials and Methods: The hearts were dissected from wild-type C57BL/6 mice and perfused with saline containing potassium (NSK) or standard cardioplegia (MT: ® Miotecter, $K^+$ 16.0mEq/ L) with/without 3.0 x $10^{-4}$ mol/L of thiamine pyrophosphate (TPP) into coronaries retrogradely (NSK vs NSK+TPP and MT vs MT+TPP, n=3/each group). Coronary perfusion by each cardioplegia was employed every 20 minutes and the hearts were separated into right and left ventricle (RV, LV) at 60 minutes after initiation. Real-time PCR was conducted with affected RV and LV muscle to assess mRNA levels of several associated proteins (Caspase-3, -8, -9, AIF, Bad, Bim, Bcl-2, p70S6K, p90RSK, Jak).
Results: There was no significant difference in associated mRNA levels among respective groups. However, in RV muscle, the tendency of lower expressions of Caspase-8, -9, AIF and Bcl-2, and the tendency of higher expression of p70S6K and Jak were observed in TPP-treated groups. In addition, in LV muscle, relative expressions of Caspase-3, -8, -9 and AIF showed a downward trend and p70S6K showed an upward trend in TPP-treated groups.
Conclusion: On mRNA levels, minimal effect of Vitamin B1 on cardioprotection during cardiac arrest was confirmed. The phosphorylation levels of ischemia-related proteins are necessary to be determined. Also, evaluations of Vitamin B1 treatment should be provided after reperfusion.
(COI:No)

### Poster Presentation 3

### [3P12]
### WPI-IIIS Joint Symposium

March 18(Fri), 13:00 - 14:30, Zoom P12

### [3P12-01]
**Development of photocaged adenosine$_{2A}$ receptor activator**

*Tsuyoshi Nagase Saitoh[1], Shuji Ioka[1], Mustafa Korkutata[1], Yohei Chitose[2], Manabu Abe[2], Mihael Lazarus[1], Hiroshi Nagase[1] ([1]*University of Tsukuba*, [2]*Hiroshima University*)

Adenosine (Ad) is an endogenous somnogen, which strongly promotes sleep through the activation of the adenosine$_{2A}$ receptor ($A_{2A}R$) in the nucleus accumbens (NAc).[1] Although the increased accumulation of Ad during wakefulness leads to induce sleep, the detailed temporal and spatial mechanisms of adenosinergic control of the NAc have not been clarified. Though photocaged $A_{2A}R$ activator has been expected as a novel optical tool to control the single receptor activity by light irradiation, the chemical tools based on the known $A_{2A}R$ agonist have an issue for separation of the endogenous and exogenous effects due to the background activity. We recently discovered a novel positive allosteric modulator (PAM) YNT378. [2] YNT378 enhanced the sensitivity of $A_{2A}R$ to endogenous Ad without showing agonist activity and induced slow-wave sleep in mice. These findings led us to develop the photocaged $A_{2A}R$ PAM (opto-YNT378) to facilitate the optopharmacology study of $A_{2A}R$.
The firth generation opto-YNT378, carrying 6-nitroveratryl (Nv) group as a photolabile protecting group, had problematic properties such as water-insolubility, slow photoresponse, and short absorption maximum. To improve the photochemical and physicochemical properties of Nv group, we developed a novel photolabile protecting group A400 based on 3-aryl coumarine chromophore. [3] The 2nd generation opto-YNT378 bearing A400 exhibited high water solubility, fast photoresponse, and an absorption maximum at 420 nm. Moreover, the time-dependent photoactivation of opto-YNT378 was observed in the cell-based assay.

**References** (1) Oishi, Y. *et al. Nat. Commun.* **2017**, 734, 1; (2) M. Korkutata *et al. Neuropharmacology* **2019**, 144, 122; (3) Chitose, Y. *et al. Org. Lett.*, **2017**, 19, 2622.

### [3P12-02]
**Opto-chemical control of sleep in the nucleus accumbens using a photocaged adenosine A2A receptor allosteric modulator**

*Koustav Roy[1], Shuji Loka[1], Mao Amezawa[1], Yoan Chitose[1], Hiroshi Nagase[1], Masashi Yanagisawa[1], Kaspar Vogt[1], Manabu Abe[2], Tsuyoshi Saitoh & Michael Lazarus*[1] ([1]*University of Tsukuba*, [2]*Hiroshima University* )

Photopharmacology may offer the possibility of curing diseases and alleviating symptoms while preventing uncontrolled drug activity, i.e., the drug is active only at the times and places where it exerts its therapeutic effect. Although chemical photo switches have been used extensively in vitro, their use in vivo has been slow, largely because of the difficulties in applying these probes in mammalian models. We revealed a prominent role of indirect pathway neurons in the nucleus accumbens (NAc) in sleep/wake regulation and proposed that the NAc links motivation and sleep. This brain circuit may explain why we feel sleepy in the absence of motivating stimuli, i.e., when we are bored. Adenosine is a plausible candidate molecule for activating NAc indirect pathway neurons to induce slow-wave sleep (SWS) because caffeine, the most widely consumed psychostimulant in the world, produces its arousal effect in the NAc by blocking adenosine $A_2A$ receptors ($A_2AR$) on indirect pathway neurons. However, the ability of adenosine in controlling NAc indirect pathway neurons for sleep induction remains to be elucidated. We recently reported the first positive allosteric $A_2AR$ modulator, named $A_2AR$ PAM, that evokes $A_2AR$ responses in the brain and developed a visible-light photoactivatable derivative of $A_2AR$ PAM (opto-$A_2AR$ PAM). Opto-$A_2AR$ PAM showed remarkable water solubility (>10 mM) and has an absorption maximum at 415 nm in aqueous solution. SWS was significantly increased for 5h in wild-type mice after intraperitoneal administration of opto-$A_2AR$ PAM and stimulation with violet light (405 nm) for 1 h after drug treatment, whereas no effect was observed in the absence of light exposure or in $A_2AR$ knockout mice. By using opto-$A_2AR$ PAM, we induced sleep for the first time in freely behaving mice by photopharmacologic allosteric $A_2AR$ modulation, suggesting that extracellular adenosine is involved in the regulation of sleep in the NAc.

### [3P12-03]
**Chemogenetic suppression of histamine receptor cells produces slow-waves in mice**

*Hikari Yashiki[1], Michael Lazarus[1], Yo Oishi[1] ([1]*International Institute for Integrative Sleep Medicine (WPI-IIIS), University of Tsukuba*)

Sleep in mice is divided into 2 stages: non-rapid eye movement (NREM) sleep and rapid eye movement (REM) sleep. Electroencephalogram (EEG) shows typical waves during these phases. The delta-range frequency (1-4 Hz) of EEG is dominant during NREM sleep, while theta-range frequency (4-8 Hz) is dominant during REM sleep. Although these criteria are used generally, the mechanism to generate these typical waves is not clear. Antihistamines induce sleepiness/sleep in humans. So, they have been used for sleep-promoting effect. Antihistamines work as inverse agonists of histamine $H_1$ receptors ($H_1R$, Gq-coupled receptors), causing pharmacological effects opposite to those of agonist. At present, the neuronal mechanisms of sleepiness/sleep-promoting effect of antihistamines are unknown and it is also unclear whether suppression of $H_1R$-expressing cells affects on sleep/wake-behaviors. In this study, we examined the effect of neuronal suppression in H1R-expressing cells on sleep/wake-behaviors in mice by a chemogenetic method. After the chemogenetic suppression, the EEG with high amplitude and low frequency was observed for a few hours even during movement. These results suggest that suppression of $H_1R$-expressing cells produces slow waves in mice.

### [3P12-04]
**Mechanisms of hippocampal adult-born neurons for memory consolidation during sleep**

*Iyo Koyanagi[1,2], Yuteng Wang[1], Sakthivel Srinivasan[1], Jiahui Yu[1], Toshie Naoi[1], Akinobu Ohba[1], Deependra Kumar[1], Pei-Hsi Wu[1], Shinji Matsuda[3], Yoan Cherasse[1], Takeshi Sakurai[1], Masashi Yanagisawa[1], Kaspar Vogt[1], Michisuke Yuzaki[4], Masanori Sakaguchi[1] ([1]*International Institute for Integrative Sleep Medicine, Univ. of Tsukuba*, [2]*Degree Programs in Comprehensive Human Sciences, Graduate School of Comprehensive Human Sciences, Univ. of Tsukuba*, [3]*Dep. of Engineering Science, Graduate School of Informatics and Engineering, The Univ. of Electro-Communications*, [4]*Dep. of Physiology, Keio Univ. School of Medicine*)

Sleep plays critical roles in memory consolidation, yet the mechanisms are still unclear. Young adult-born neurons (ABNs) in the hippocampal dentate gyrus (DG) bestow unique plasticity to the memory circuit. We found that ABNs exhibit sparse activity during REM sleep, which is necessary for memory consolidation (Kumar and Koyanagi et al., Neuron, 2020). A prominent synchronous neural activity (i.e., theta rhythm) appears in the DG during REM sleep. Theta rhythm coordinates both synaptic plasticity and memory consolidation. To reveal the functions of the theta rhythm with ABNs during REM sleep, we examined hippocampal theta-phase specific ABN activity for memory consolidation by closed-loop optogenetic manipulation. This study provides insights into how the unique cellular plasticity in the adult brain operates synchronously with brain rhythm for memory consolidation during sleep.

### [3P12-05]
**Adult-born neuron activity in the establishment of fear generalization**

*Jiahui Yu[1], Pablo Vergara[1], Toshie Naoi[1], Yuteng Wang[1], Yoan Cherasse[1], Takeshi Sakurai[1], Masanori Sakaguchi[1] ([1]*WPI-IIIS, University of Tsukuba*)

Overgeneralization of fear in safe contexts is considered a hallmark of post-traumatic stress disorder. Traditional behavior paradigm to elicit fear generalization requires more than ten days, which prevents the understanding of how fear generalization establishes in parallel with memory processing, including memory consolidation. It is known that adult-born neurons (ABNs) continuously generated in the hippocampus play a key role in contextual fear generalization. We have elucidated the dynamic activity change of ABNs in REM sleep and their necessity in memory consolidation. However, their activity patterns responsible for fear generalization are completely unknown. In this study, we first constructed a robust behavior paradigm to analyze the process of contextual fear generalization. To reveal the temporal and spatial activity of ABNs, we employ calcium imaging in freely moving mice. This study provides new insights into understanding the mechanisms of establishing contextual fear generalization.

### [3P12-06]
### Synchronous young and matured neuron activity for memory consolidation

*Yuteng Wang[1], Pablo Vergara[1], Sakthivel Srinivasan[1], Jiahui Yu[1], Toshie Naoi[1], Yoan Cherasse[1], Takeshi Sakurai[1], Masanori Sakaguchi[1] ([1]*WPI-IIIS, Tsukuba Univ.*)

Brain plasticity shifts from cellular to synaptic during development. However, cellular plasticity remains largely functional in the hippocampal dentate gyrus (DG) throughout life; adult-born neurons (ABNs) are continuously generated in DG that play pivotal roles in memory. Indeed, we found that the sparse activity of ABNs during REM sleep is necessary for memory consolidation (Kumar et al., Neuron, 2020). It suggests synchronized ensemble activity between ABNs and the developmentally-born matured neurons for memory consolidation during sleep. However, the activity and its functional significance remain completely unknown. To tackle this issue, we have tested various methods utilizing the Calcium-imaging and miniaturized microscope we developed. Our study aims to pave a way to uncover how cellular plasticity plays distinct roles in concert with already existing circuits in the adult brain.

### [3P12-07]
### CaliAli: A tool for inter-session alignment of 1-photon calcium imaging data allowing tracking neurons in the non-rigidly moving brain

*Pablo Vergara[1], Sakthivel Srinivasan[1], Toshie Naoi[1], Yoan Cherasse[1], Takeshi Sakurai[1], Masanori Sakaguchi[1] ([1]*International Institute for Integrative Sleep Medicine (WPI-IIIS), University of Tsukuba*)

Endoscopic calcium imaging allows monitoring the activity of neurons across days and weeks. Current algorithms to track neurons across video sessions rely on the alignment of spatial footprints of neurons independently extracted from each session. However, because the brain is a non-rigid structure, movements across sessions are often not expressed as a simple translation transformation. In this scenario, the spatial footprints of neurons are commonly not sufficient to align imaging sessions, especially when neurons overlap with each other, are sparsely distributed, or when their activities remap (i.e., neurons disappear in some recording sessions). To address this issue, we developed CaliAli (Calcium imaging Inter-session Alignment), a tool that utilizes blood vessels in addition to spatial cues of neurons to align video sessions automatically. Because CaliAli aligns video sessions before neural extractions, it is possible to extract weak calcium signals that would be otherwise undetected by independent analysis of each recording session. Moreover, CaliAli outperforms other tracking approaches in conditions of high neural overlap or remapping of neuron activities and can be applied to highly condensed populations such as the granule cells in the dentate gyrus and also sparsely distributed population such as the hippocampal adult-born neurons.

### [3P12-08]
### Transient recruitment of an adult-born neuron ensemble for fear memory consolidation in REM sleep

*Sakthivel Srinivasan[1], Iyo Koyanagi[1], Yuteng Wang[1], Pablo Vergara[1], Jiahui Yu[1], Toshie Naoi[1], Kaspar E. Vogt[1], Yoan Cherasse[1], Takeshi Sakurai[1], Keita Iio[1], Noriki Kutsumrura[1], Hiroshi Nagase[1], Masanori Sakaguchi[1] ([1]*WPI-IIIS, University of Tsukuba*)

Memory replay during sleep is suggested to contribute to memory consolidation during sleep. The dentate gyrus (DG) in the hippocampus encodes contextual fear memory trace. Moreover, we showed that the activity of the adult-born DG neurons (ABNs) during REM sleep is necessary for memory consolidation (Kumar and Srinivasan et al., Neuron, 2020). Therefore, we examined whether re-activation of context encoding ABNs during REM sleep is necessary for memory consolidation. Our study reveals the coding mechanisms critical for fear memory consolidation during sleep.

### [3P12-09]
### Whole brain mapping and manipulation of activated neuronal populations by exhausted exercise

*Shingo Soya[1], Saki Yamada[1], Takashi Matsui[1], Takeshi Sakurai[1] ([1]*Tsukuba Univ*)

Exhausted exercise induces a variety of physiological responses including increased core body temperature, glycogen reduction, hypoglycemia, described with so-called "Fatigue". However, neural mechanism that integrates these physiological alterations has been largely unknown. To depict the neural populations which are active during exhausted exercise, we utilized TRAP (Targeted Recombination in Active Populations) system. We crossed *TRAP2*-mice with *Rosa26-CAG-lsl-hM3Dq* (*hM4Di*)*-mCherry* mice and used motor-driven treadmill for capturing and manipulating neurons activated in exhausted exercise in whole brain. We found that exhausted exercise induced increased expression of hM3Dq-mCherry in several brain regions including the POA, DMH, PVN, PH, Pons, PAG. Chemogenetic activation of the trapped neurons by intraperitoneal injection of CNO (Clozapine-N-Oxide) showed reduction of body temperature and energy expenditure with immobility. Chemogenetic inactivation of these neurons showed no change in energy expenditure and body temperature. We found surface body temperature decreased during exhausted exercise on treadmill. These results suggest that neurons which are activated during exhausted exercise might regulate surface body temperature to maintain core body temperature during exhausted exercise.

### [3P12-10]
### Capturing and manipulating neurons activated by exhausted exercise

*Saki Yamada[1], Shingo Soya[1], Takashi Matsui[1], Takeshi Sakurai[1] ([1]*University of Tsukuba*)

Exhausted exercise induces a variety of physiological responses including increased core body temperature, glycogen reduction, hypoglycemia, described with so-called "Fatigue". However, neural mechanism that integrates these phenomena is largely unknown. First, we utilized TRAP (Targeted Recombination in Active Populations) system by using *TRAP2*-mice which express *iCre* under the c-fos promoter. We crossed *TRAP2*-mice with *Rosa26-CAG-lsl-hM3Dq-mCherry* and utilized motor-driven treadmill for capturing and manipulating neurons activated in exhausted exercise in whole brain. We found that exhausted exercise induces increased expression of hM3Dq-mCherry in several brain regions. Activation of these neurons by intraperitoneal injection of CNO (Clozapine-N-Oxide) showed reduction of body temperature and energy expenditure with increased immobility. To search the brain regions which controls these phenomena, utilized Cre-dependent AAV expressing hM3Dq-mCherry with *TRAP2*-mice. We injected AAV into the AVPe, DMH, PH regions to capture and manipulate neurons which are activated during exhausted exercise. Chemogenetic activation of AVPe neurons showed decreased body temperature and energy expenditure with immobility. On the other hand, activation of DMH and PH neurons showed increased energy expenditure and body temperature accompanied with tail vasodilation. Also, we found body temperature decreases during exhausted exercise on treadmill. These results suggest that neurons which are activated during exhausted exercise showed opposite effect on body temperature and energy expenditure regulation.

### [3P12-11]
### Rapid eye movement sleep is initiated by dopamine signaling in the basolateral amygdala in mice

*Emi Hasegawa[1,2], Ai Miyasaka[1], Katsuyasu Sakurai[1], Yoan Cherasse[1], Yulong Li[3], Takeshi Sakurai[1,2,4] ([1]*International Institute for Integrative Sleep Medicine (WPI-IIIS), University of Tsukuba*, [2]*Faculty of Medicine, University of Tsukuba*, [3]*State Key Laboratory of Membrane Biology, Peking University School of Life Sciences*, [4]*Life Science Center for Tsukuba Advanced Research Alliance (TARA), University of Tsukuba*)

The sleep cycle alternates between REM (rapid eye movement) and NREM (non-rapid movement) sleep, which is a highly characteristic feature of sleep. However, the mechanisms by which this cycle is generated have not been well-characterized. We found that a periodic transient increase of dopamine (DA) level in the basolateral amygdala (BLA) during non-rapid eye movement (NREM) sleep terminates NREM sleep and initiates REM sleep. DA acts on dopamine receptor D2 (Drd2)-expressing neurons in the BLA to induce a transition from NREM to REM sleep. This mechanism also plays a role in cataplectic attack, which is a pathological intrusion of REM sleep into wakefulness in narcoleptics. These results show a critical role of DA signaling in the amygdala in REM sleep regulation and provide a neuronal basis of sleep cycle generation.

### [3P12-12]
### GABAergic neurons in the ventrolateral periaqueductal gray are implicated in cataplexy of narcoleptic mice.

*Sol Jeong[1], Shuntaro Uchida[2], Shingo Soya[1], Takeshi Sakurai[1] ([1]*University of Tsukuba,* [2]*International Institute for Integrative Sleep Medicine*)

The loss of orexinergic neurons causes narcolepsy, a sleep disorder characterized by chronic sleepiness and cataplexy. Cataplexy is a sudden muscle atonia during wakefulness, which is considered as the intrusion of REM sleep into wakefulness. The midbrain region, especially ventrolateral periaqueductal gray(vlPAG) is known to regulate REM sleep. However, the role of vlPAG in cataplexy is poorly understood. We identified vlPAG and several brain regions as the upstream of glutamatergic neurons in sublaterodorsal tegmental nucleus(SLD) projecting to ventromedial medulla (VMM; Glu$^{SLD\rightarrow VMM}$), which is known as the common pathway of cataplexy and REM atonia (Uchida et al., 2021). Based on the previous research, we injected Cre-dependent AAV expressing hM3Dq-mCherry into the vlPAG in *vGAT-IRES-Cre* mice. Chemogenetic activation of vlPAG GABAergic neurons decreased the amount of cataplexy in narcoleptic *orexin-ataxin3* mice. These observations suggest that vlPAG GABAergic neurons suppressed cataplexy through SLD, and a lack of orexin signaling might induce the abnormal activity of vlPAG GABAergic neurons leading to muscle atonia.

### [3P12-13]
### Visualizing input-output architecture of orexin neurons with doublecolor projection-selective retrograde tracing

*Yuki Cleo Saito[1], Yoan Cherasse[1], Yasutaka Niwa[1], Takeshi Sakurai[1] ([1]*University of Tsukuba*)

Orexin-producing neurons (orexin neurons), located in the lateral hypothalamic area (LHA), play a highly important role in maintaining wakefulness. Orexin neurons send widespread projections to nuclei containing monoaminergic neurons such as VTA, LC, TMN, and raphe nuclei, all of which contain monoaminergic neurons. We previously identified input neurons that make direct synaptic contacts to orexin neurons with modified rabies vector-based retrograde tracing. That study showed orexin neurons received input from various regions of the brain. In this study, we used projection sites-specific rabies-mediated monosynaptic retrograde tracing to identify the neuronal inputs to the orexin neurons with projections to particular regions. In addition, we used newly generated orexin-iCre KI mice to analyze the input-output relationship of orexin neuronal circuits using the modified multi-color simple-cTRIO method. This new method allowed us to detect more than two different input-output pathways in the same brain. This study revealed that orexin neurons projecting to each output region also send projections to all brain regions we previously examined. However, we found some biased input and output architectures. Orexin$^{LHA\rightarrow LC}$, Orexin$^{LHA\rightarrow VTA}$, and Orexin$^{LHA\rightarrow DR}$ neurons have mostly overlapping but partly distinct distributions of input neurons.

### [3P12-14]
### Delineation of Neural Circuits of Galaninergic Neurons in the VLPO Implicated in Regulation of Sleep

*Kseniia Prokofeva[1,2], Yuki C. Saito[2], Arisa Hirano[2,3], Takeshi Sakurai[2,3,4] ([1]*Grad Sch Comprehens Hum Sci, Univ of Tsukuba,* [2]*WPI-IIIS, Univ of Tsukuba,* [3]*Fac Med, Univ of Tsukuba,* [4]*LS-TARA Cent, Univ of Tsukuba*)

Sleep and wakefulness are regulated through dynamic reciprocal interactions between sleep- and arousal-driving neuronal networks. In this intricated circuitry, orexin neurons located in the lateral hypothalamus (LH) play a pivotal role in maintenance of wakefulness, while GABAergic and galaninergic (GAL) neurons in the ventrolateral preoptic nucleus (VLPO) participate in initiation and maintenance of sleep. Previous studies showed that orexinergic neurons are innervated by GABAergic neurons in the VLPO. However, connection and functional interaction between orexin neurons and GAL$^{VLPO}$ neurons has not been examined. The aim of this study is to identify sleep-implicated neural circuits comprising of the GAL$^{VLPO}$ and orexinergic neurons. We conducted monosynaptic retrograde rabies-mediated tracing from orexin neurons using newly generated *Orexin-iCre* knockin mice and visualized galanin (*Gal*) and vesicular GABA transporter (*Vgat*) mRNAs in the VLPO inputs implementing FISH. We found that *Gal*-positive VLPO neurons, mainly co-expressing *Vgat*, make direct synaptic inputs to orexin neurons. We next performed optogenetic stimulation of the axons of GA L$^{VLPO}$ neurons in the LH. Our results suggest that activation of the GAL$^{VLPO\rightarrow LH}$ pathway promoted NREM sleep. Further, we conducted projection-specific rabies-mediated tracing from GAL$^{VLPO\rightarrow LH}$ neurons and identified that they receive monosynaptic inputs from various functionally diverse brain areas. These findings uncover connectivity of GAL$^{VLPO}$ neurons and the role of their circuits in governance of sleep.

### [3P12-15]
### Mechanism by which the BNST→DpMe pathway induces arousal

*Zhongwen Zhang[1,2], Yoan Cherasse[2], Takeshi Sakurai[2] ([1]*Faculty of medicine, University of Tsukuba,* [2]*International Institute for Integrative Sleep Medicine (WPI-IIIS), University of Tsukuba*)

Sleep disorders are known as one of the core symptoms of depression, anxiety disorders and PTSD patients. Previous study demonstrated that the optogenetic stimulation of GABAergic neurons in the bed nucleus of the stria terminalis (BNST) triggered immediate transition from nonrapid eye movement (NREM) sleep to wakefulness in male mice. Dense innervation from GABA$^{BNST}$ neurons to the deep mesencephalic nucleus (DpMe), one of the regions implicated in promoting arousal was observed. We demonstrated here that optogenetic stimulation of GLUT$^{DpMe}$ neurons resulted in immediate transition from NREM sleep to wakefulness. We hypothesized that some GABA$^{DpMe}$ interneurons might inhibit GLUT$^{DpMe}$ neurons and receive inhibitory projections by GABA$^{BNST}$ neurons. We also performed retrograde neuronal circuit tracing with a modified rabies virus vector to examine the neuronal connectivity between the BNST and DpMe. Combined with in situ hybridization, we concluded that GABA$^{BNST}$ neurons do not make direct synaptic connections to GLUT$^{DpMe}$ neurons. However, we found that GABA$^{DpMe}$ neurons receive direct synaptic input from GABAergic and glutamatergic neurons in the DpMe as well as GABA$^{BNST}$ neurons. This research revealed the neuronal circuits involving GABA$^{BNST}$ and GLUT$^{DpMe}$ neurons that play a crucial role in quick switching from NREM sleep to wakefulness upon emotional stimuli. This research sheds light on the mechanisms of stress- or fear-related insomnia symptoms observed in some psychiatric disorders, like post-traumatic stress disorder (PTSD) and social anxiety disorder (SAD) in the future.

### [3P12-16]
### Gastrin-releasing Peptide Producing Neurons in the Suprachiasmatic Nucleus play an Essential Role in Regulating Circadian Rhythm

*Ruth Li[1,2], Yoan Cherasse[2], Ran Inoue[3], Hisashi Mori[3], Arisa Hirano[2,4], Takeshi Sakurai[2,4] ([1] *University of Tsukuba, Graduate School of Comprehensive Human Sciences,* [2] *University of Tsukuba, International Institute for Integrative Sleep Medicine (IIIS),* [3] *University of Toyama, Department of Molecular Neuroscience,* [4]*University of Tsukuba, Faculty of Medical Sciences*)

Most organisms exhibit behavioral, physiological, and molecular fluctuations in cycles of around 24 hours, known as circadian rhythm, which is regulated by the circadian master clock, or the hypothalamic suprachiasmatic nucleus (SCN) in mammals. The SCN contains distinct subtypes of neurons expressing different neurotransmitters, such as arginine vasopressin (AVP), vasoactive intestinal peptide (VIP), and gastrin-releasing peptide (GRP). Studies assessing the role of each neurotransmitter have been conducted over the past few decades, but little is known about the different neurons that express each peptide. In this study, we focused on unraveling the role of gastrin-releasing peptide producing neurons (GRP neurons) in regulating circudian rythmicity, as little research has been done regarding these neurons. We used a newly generated mouse line, Grp-iCre knock-in (KI) mice and Cre-dependent adeno associate virus (AAV) to specifically manipulate SCN GRP neurons. We discovered that when we inhibit the SCN GRP neurons with tetanus toxin light chain (TeNTLC), mice display attenuated behavioral rhythmicity compared to control mice that express only green fluorescent protein (GFP). When we examined the core clock gene expression rhythm within the SCN of mice with inhibited SCN GRP neurons, we discovered that the oscillation amplitude of PER2 expression is drastically lower in these mice than in control mice. These results suggest that SCN GRP neurons are crucial for sustaining SCN cellular rhythmicity and in turn, is important for regulating behavioral rhythm.

### [3P12-17]
### Induction of mouse hibernation-like state using high sensitive optogenetics

*Tohru Takahashi[1,2], Hirano Arisa[1,2], Sakurai Takeshi[1,2] ([1]*Fucul of Med, Univ of Tsukuba,* [2]*WPI-IIIS, Univ of Tsukuba*)

Although a variety of useful optogenetic tools are currently available, none have been reported that can be effectively used for continuous stimulation of neurons for a long time (e.g., 24 hours). We generated an opsin (here termed HPN1) and applied it for inducing a hibernation-like hypometabolic state, the Q neurons-induced hypometabolic state (QIH) in mice. Excitation of Q neurons by HPN1 induced deep hypothermia for a long time by illuminating light with very low intensity, with a high temporal resolution. The HPN1- mediated QIH recapitulated some kinetics of physiological changes observed in the natural hibernation. The extremely high-sensitive optogenetics will allow us to do transcranial optogenetics as well as localized light stimulation with minimal damages in tissues. This could also enable proper optogenetic manipulation that would be affected by light or heat, such as research on circadian rhythms and thermoregulation.

### [3P12-18]

### Roles of monoamines and their regulatory systems in motivation and arousal

*Yoan Cherasse[1], Shingo Soya[1], Yuki Cleo Saito[1], Hiroshi Ichinose[2], Takeshi Sakurai[1] ([1]*University of Tsukuba,* [2]*Tokyo Institute of Technology School of Life Science and Technology*)

The exact mechanisms by which monoamines (MA) such as dopamine, serotonin, histamine and noradrenaline regulate human behaviours and how their abnormalities result in several psychiatric disorders have been incompletely understood. Our project aims at producing a constructive understanding between monoamines and psychiatric disorders, with a particularly focus on the depression-related symptoms of motivation and arousal. We generated genetically modified mice expressing Cre recombinase under the control of MA promoters with a conditional KO mouse for GTP cyclohydrolase 1 (GCH1), the gene indispensable for production of MA. We then evaluated psychiatric disorders in addition to sleep and circadian rhythm. Our experiments on serotonin deficient mice showed a reduced wheel-running activity compared to control groups. The noradrenergic deficient mice exhibited unusual increase of electromyogram during NREM sleep. Mice deficient in dopamine showed a drastic increase of wakefulness during the dark phase compared to control mice, accompanied by a massive reduction of sleep and low anxiety. Finally, mice depleted in MA of neurons projecting to the prefrontal cortex exhibited an abnormal response to fear conditioning. We also analyzed effect of icv orexin on sleep/wakefulness behavior in MA-deficient mice.

### [3P12-19]

### The anatomical and functional understanding of neural dynamics of VTA dopaminergic neurons in female sexual behavior

*Katsuyasu Sakurai[1], Kai Yashiro[1], Qinghua Liu[1], Takeshi Sakurai[1] ([1]*University of Tsukuba*)

Understanding women's sexual motivation is essential to achieving gender equality. However, the underlying neural mechanisms of female sexual motivation have remained unclear. To understand the neural basis of female sexual motivation, we focused on dopamine which is recognized as a crucial neural substrate for sexual behavior in both male and female animals. Here, we examined the neural activities of VTA dopaminergic neurons in a female mouse during sexual behavior by using in vivo imaging system, fiber photometry. We discovered the dramatic surge of the neural activity of VTA dopaminergic neurons in the female mouse immediately after male mouse ejaculation. Currently, we are trying to reveal the function or biological meaning of this dramatic dopamine surge of the female mouse at the timing of male mouse ejaculation by applying neural circuit-specific manipulation methods involving histological, optogenetic, and chemogenetic strategies. We will introduce those data at the meeting.

### [3P12-20]

### Dopamine dynamics in NAc-vs control male sexual behaviors in mice

*Ai Miyasaka[1], Yuka Terakoshi[1], Katsuyasu Sakurai[1], Takeshi Sakurai[1], Qinghua Liu[1,2] ([1]*University of Tsukuba, WPI-IIIS,* [2] *National Institute of Biological Sciences, Beijing.*)

In male mice, sexual behaviors consist of a series of stereotypic steps, such as sniffing, mounting, intromission, and ejaculation. However, the neural mechanisms by which these behaviors are regulated and coordinated have remained unclear. By using GRAB-DA sensor to monitor dopamine (DA) signaling in the nucleus accumbens (NAc), we found that DA dynamics in the NAc ventral shell region (NAc-vs) corresponded nicely to sequential steps of male sexual behaviors: 1) DA release is increased at the initiation of mounting: 2) DA level fluctuates rhythmically during intromission: 3) DA level is decreased right before ejaculation. To characterize the functional importance of DA dynamics in NAc-vs, we optogenetically manipulated the NAc-vs projecting dopaminergic neurons in ventral tegmental area. Interestingly, inhibiting the dopaminergic neurons at the onset of sniffing or mounting stopped sexual behaviors. Moreover, inhibition of the dopaminergic neurons during intromission induced ejaculation, whereas activation during intromission prolonged ejaculation latency. These results suggest that DA dynamics in NAc-vs contribute critically to the regulation of sexual behaviors in male mice.

### [3P12-21]

### Analyses of the effect of prior social stress on brain activity during social interaction in mice using a novel semi-automated c-Fos mapping program

*Hibiki Okamura[1,2], Shinnosuke Yasugaki[1,3], Yoshifumi Arai[1,4], Katsuyasu Sakurai[1], Masashi Yanagisawa[1], Hotaka Takizawa[5], Yu Hayashi[1,6] ([1]*WPI-IIIS, Univ. of Tsukuba,* [2]*Ph. D. Program in Humanics, Univ. of Tsukuba,* [3]*Doctoral Program in Biomedical Sciences, Graduate School of Comprehensive Human Sciences, Univ. of Tsukuba,* [4]*Master's Program in Medical Science, Graduate School of Comprehensive Human Science, Univ. of Tsukuba,* [5]*Department of Computer Science, Univ. of Tsukuba,* [6]*Graduate School of Medicine, Kyoto Univ.*)

Depression is a major psychiatric disorder worldwide. Stressful social experience is a factor for depression. However, the precise mechanism that links social stress to depression remains unclear. Mice exhibit depression-like phenotypes following chronic stress. Social defeat stress (SDS) is one of the methods to apply chronic stress to mice. Here, we investigated how chronic SDS affects BALB/c mice. While BALB/c mice are reported to show higher stress susceptibility compared to the widely used C57BL/6 mice, there are few studies that applied SDS to BALB/c mice. We found that BALB/c mice showed depression-like states including reduced social interest after chronic SDS and the effects remained even after 2 weeks. Furthermore, we aimed to investigate the brain regions that show altered activity following social interaction in mice that underwent SDS by means of c-Fos mapping. To this end, we established a novel method to conduct whole-brain c-Fos mapping based on semi-automated image processing, and the screening revealed several brain regions that showed different c-Fos expression levels in stressed mice compared to the control mice. These results contribute to understanding how prior social stress affects the brain activity during social interaction and provide a novel method for efficient c-Fos mapping across various brain regions.

### [3P12-22]

### Is increased REM sleep an adaptive response or an exacerbating factor in stress resilience?

*Shinnosuke Yasugaki[1,2], Mitsuaki Kashiwagi[1], Mika Kanuka[1], Masashi Yanagisawa[1], Yu Hayashi[1,3] ([1]*WPI-IIIS, Univ of Tsukuba,* [2]*Doc Program in Biomed Sci, Grad Sch of Comprehensive Hum Sci, Univ of Tsukuba,* [3]*Dep of Hum Health Sci, Grad Sch of Med, Kyoto Univ*)

Our sleep consists of two stages: rapid-eye-movement (REM) sleep and non-REM (NREM) sleep. Currently, the function of REM sleep is largely unknown. Here, we focused on depression. Patients with depression almost inevitably exhibit sleep disorders, and in particular, abnormalities in REM sleep, most commonly increases in REM sleep amounts, are frequently observed. There are controversies as to whether increased REM sleep helps recovery from depression or rather contributes to worsening of the depression-related symptoms. As a first step to address this in mice, we aimed to analyze the effects of 10 days of social defeat stress on sleep in mice. Similar to patients with depression, stress exposure in mice increased REM sleep. Moreover, the increase in REM sleep that observed after acute stress exposure tended to attenuate after chronic stress exposure. Based on these results, we next investigated the effects of artificially increased REM sleep at specific timings by chemogenetic activation of the REM sleep-promoting neurons recently identified in our laboratory. Surprisingly, we found that repeated activation of this neurons has antidepressant effects on a decrease in social interests induced by chronic social defeat stress. Now we are trying to reveal how artificially increased REM sleep contributed to those behavioral phenotypes. Our future work will provide new insights to address the causal relations between stress resilience in mice and REM sleep.

### [3P12-23]

### Characterization of the neuronal activity of a newly identified REM sleep-regulating neuron using glass pipette extracellular unit recording combined with optogenetics

*Yoshifumi Arai[1,2], Mitsuaki Kashiwagi[1], Mika Kanuka[1], Takaya Suganuma[1], Kaeko Tanaka[1], Masanori Sakaguchi[1], Masashi Yanagisawa[1], Yoshimasa Koyama[3], Yu Hayashi[1,4] ([1]*WPI-IIIS, Univ. of Tsukuba,* [2]*Master's Program in Medical Sciences, Grad school of Human Comprehensive Sciences, Univ. of Tsukuba,* [3]*Dept Sci Technol, Fukushima Univ.,* [4]*Grad school of Medicine, Kyoto Univ.*)

Human beings spend about one-third lifetime in sleep. Mammalian sleep comprises nonrapid eye movement sleep (NREM) sleep and rapid eye movement (REM) sleep. Previous studies revealed that the brainstem plays a crucial role in the regulation of REM sleep. However, the mechanism of REM is still largely unknown. Glass pipette extracellular unit recording, a conventional electrophysiological technique, has been used to characterize neuronal activity in the sleep/wake cycle. This method is robust in terms of temporal resolution and can record even a small neuron. A new REM sleep-regulating subtype neuron has been identified in the dorsal pons and the activity of this neuron in the sleep/ wake cycle should be characterized. However, it has been demanding to record a specific subtype neuron and a neuron that fires at low frequency in the method. To overcome this limitation, we combined the opto-tagging method with glass pipette extracellular unit recording and made a cell-type-specific unit recording. We found that some of the REM sleep-regulating neurons showed increased activity during REM sleep and these neurons may play important role in REM sleep regulation.

### [3P12-24]

### Analysis of the mechanism of REM sleep behavior disorder with focus on Parkinson's disease

*Ami Kaneko[1,2], Shinnosuke Yasugaki[1,3], Hideki Hayakawa[4], Kensuke Ikenaka[4], Cesar Aguirre[4], Masashi Yanagisawa[1], Kousuke Baba[4], Hideki Mochizuki[4], Yu Hayashi[1,5] ([1]*WPI-IIIS, Univ. of Tsukuba*, [2]*Ph. D. Program in Humanics, Univ. of Tsukuba*, [3]*Doctoral Program in Biomedical Sciences, Grad School of Comprehensive Human Sciences, Univ. of Tsukuba*, [4]*Department of Neurology, Osaka Univ. Grad School of Medicine, Suita, Japan*, [5]*Grad school of Medicine, Kyoto Univ.*)

During rapid eye movement (REM) sleep, the cerebral cortex becomes activated and produces vivid dreams. Yet, it normally does not lead to motor output owing to expression of muscle atonia. However, patients with REM sleep behavior disorder (RBD) exhibit impaired muscle atonia during REM sleep and frequently act out of their dreams. For example, they talk loudly or exhibit violent movements such as hitting and kicking. RBD patients often wake up following such movements, which can lead to reduced sleep quality.
A majority of RBD patients co-suffer from synucleinopathies including Parkinson's disease and dementia with Lewy bodies or eventually develop these diseases within 10-14 years. Thus, RBD is considered as a prodromal of synucleinopathies. Here, we aimed to understand the mechanisms underlying the link between RBD and synucleinopathies and establish an RBD mouse model for future development of effective treatment for RBD. A rare G51D α-synuclein mutation leads to production of toxic α-synuclein fibril and patients carrying this mutation show rapid progression of Parkinson's disease. We produced and injected G51D α-synuclein fibrils into the brainstem pontine tegmental area in mice, and observed effects on atonia during REM sleep, behavior and motor symptoms.

### [3P12-25]

### Effects of chronic cell ablation in the preoptic area on sleep-wake cycles

*Ayako Imamura[1,2], Koichi Miyatake[2], Akiko Ihara[2], Sakiko Honjoh[2] ([1]*Ph.D. Program in Humanics, University of Tsukuba*, [2]*IIIS, University of Tsukuba*)

Sleep and wakefulness are controlled by specific brain regions. These regions have been identified by classic electric stimulation of non-specific cell types and acute neural activity manipulation. However, despite many experiments that show that acute manipulation of these regions has a temporary effect on the sleep-wake cycles, chronic inhibition of neural activity often has no effect.
This is thought to be due to adaptation and compensation by other brain regions. In this study, we focused on the preoptic area (POA), which has been reported to promote sleep . With conventional methods such as electrolytic lesions, axons passing through the region are also affected, so functional inhibition is likely not brain region-specific. The effect of brain region-specific cell ablation in the POA on sleep-wake cycles is unclear. Then, we investigated the effect of brain region-specific cell ablation in the POA on the sleep-wake cycle. In this method, we injected taCasp3 AAV, which overexpresses procaspase-3 in a Cre-dependent manner and induces apoptosis, in the POA -specific to avoid the effects of passing axons and ablate the cells in the brain region. As a result, the fragmentation of sleep-wake cycles was observed, the frequency of transitions from wakefulness to sleep increased, and the duration of sleep decreased. This effect was sustained for more than four weeks, and no compensation by other brain regions was observed. These results suggest that POA is necessary for consolidated sleep-wake cycles.

### [3P12-26]

### Toward label-free molecular imaging of whole brain and brain cells using ultra-broadband multiplex CARS microspectroscopy

*Yusuke Murakami[1,2], Shinichi Miyazaki[1,2], Philippe Leproux[3,4], Sakiko Honjoh[2], Hideaki Kano[5] ([1]*Ph.D. Program in Humanics, University of Tsukuba*, [2]*International Institute for Integrative Sleep Medicine (WPI-IIIS)*, [3]*University of Limoges*, [4]*University of Poitiers*, [5]*Department of Chemistry, Faculty of Science, Kyushu University*)

Mapping the distribution of chemical bonds throughout brain tissue is inevitable for understanding brain functions. We applied label-free ultra-broadband multiplex CARS (MCARS) spectroscopic imaging system to construct the whole brain chemical molecular map. Our experimental setup enabled us to acquire the MCARS spectrum with the spectral coverage of approximately 3000 $cm^{-1}$ from the macro (tissue level) and micro (cellular level) scales. As a result, the MCARS images of the whole brain slice at $CH_2$ stretching and $CH_2$ scissoring bands were assigned as lipids, which mainly visualize the white matter. Next, we visualized the hippocampus and cortex on micro-scale. The ratio of the vibrational band due to $CH_3$ stretching to that of OH stretching indicates that the water content of these neurons is more abundant than other surrounding regions. In the cortex, localized lipid-rich areas were also observed. This probably corresponds to the distribution of lipid-rich glial cells such as astrocytes and oligodendrocytes, which are one of the most abundant cells in the brain. These results were suggesting that MCARS spectroscopic images can be used to distinguish cell types in the brain. In conclusion, the whole brain label-free molecular imaging with single-cell resolution will allow a comprehensive analysis of molecules in the brain and will lead to a better understanding of brain function and structure.

### [3P12-27]

### The role of hypothalamic supraoptic nucleus in maintaining wakefulness

*Olga Malyshevskaya[1] ([1]*University of Tsukuba*)

This project arisen from accidental finding that photostimulation of supraoptic (SO) nuclei resulted in prolonged wake state. Based on previous in-situ hybridization data we assumed that SO nucleus is involved in cannabinoid convulsive effects and therefore investigated the effect of optogenetic activation of SO nucleus. Bilateral photostimulation of SO nuclei did not induce electrographic seizures, however, we discovered that prolonged photostimulation (4 hours) resulted in increased wakefulness (time in wake) with complete absence of sleep (NREM and REM) during stimulation, which was highly significant and resulted in sleep rebound accompanied by increased NREM sleep amount and delta NREM amount immediately after the end of stimulation. We have also examined the effect of 8 hrs photostimulation, which resulted in normal wake behavior in the absence of sleep for the first 4 hours and significant decrease of sleep for the rest of photostimualtion time. This suggests that SO nucleus is strongly involved in inducing and possibly maintaining arousal state. To our knowledge this data has not been reported by anyone yet. We are planning to throroughly investigate how activation of SO nuclei produces arousal, what mechanism underlie this phenomenon and to anatomically dissect inputs and outputs of the structures causing the arousal effect.

### [3P12-28]

### Recovering EEG Generators from Their Collective Stochastic Interference

*Javier Diaz[1] ([1]*University of Tsukuba*)

Mainstream descriptions of EEG rely on its analysis in the frequency domain and basically explain the observed EEG oscillations as emerging from neuronal synchronization processes. Traditionally, these oscillations are categorized into spectral bands whose correlates with different brain states are well established. Recently, however, some alternative views of EEG emphasize its arrhythmic nature, focusing on global properties of the spectrum as, for example, the observed 1/f spectrum. Following this line of thought, in the present study we modeled EEG as a stochastic process emerging from the superposition of arrhythmic pulses described by arbitrary functions. Even though this kind of superposition generates colored Gaussian noise, from our simulations we discovered that it is still possible to statistically recover the shape of the underlying pulse. Applying these concepts to actual EEG, unique patterns characterizing NREM sleep, REM sleep, quiet wake, and active wake can be identified. Remarkably, using the presented methodology, all these behavioral states can be determined directly from EEG, not requiring EMG. In addition, although it is well-known atropine induces EEG delta waves that can be confused with NREM sleep EEG, our method shows a distinctive pattern for the atropine effect, very different from those patterns associated with NREM sleep or any other physiologic state.

### [3P12-29]

### Dihydropyridine calcium blockers do not interfere with slow wave sleep

*GoEun Han[1], Javier Diaz[2], Sumire Matsumoto[3], Kaspar Vogt[2], Robert Greene[4] ([1]*Human Biology Program, University of Tsukuba, IIIS*, [2]*IIIS, University of Tsukuba*, [3]*Tohoku University*, [4]*University of Texas Southwestern*)

Slow wave or non-rapid eye movement (NREM) sleep is tightly homeostatically regulated and essential for survival. Slow waves are observed in the electro-encephalogram as oscillations in the delta range. Slow wave activity is to date the best indicator for homeostatic sleep regulation; it is increased after prolonged waking and slowly reduced during NREM sleep. The precise mechanisms underlying sleep homeostasis and the generation of slow waves are unknown. Activity-dependent neuronal calcium influx has been hypothesized to play an important role in generating slow oscillations and might be involved downstream signaling that mediates sleep function. Dihydropydirine blockers of L-type voltage gated calcium channels (VGCCs) are in wide clinical use to treat hypertension and other cardiovascular disorders and are readily blood-brain-barrier penetrant. We therefore investigated their potential effect on slow wave generation and homeostatic NREM sleep regulation. In-vivo two-photon imaging of cortical neurons showed larger spontaneous calcium transients in slow wave sleep compared to waking. Application of the dihydropyridine calcium blocker nicardipine significantly reduced cortical calcium transients without affecting slow wave generation. Time spent in slow wave sleep and episode duration were also not affected. We conclude that despite evidence that neuronal calcium influx may be involved in NREM sleep function, block in calcium entry through L-type VGCCs does not interfere with slow wave generation or regulation.

### [3P12-30]

### The effects of olfactory stimulation during REM sleep on dream emotionality: a study focusing on individual difference in olfactory perception

*Satomi Okabe[1,2,3], Takashi Abe[1,2] ([1]*University of Tsukuba,* [2]*International Institute for Integrative Sleep Medicine (WPI-IIIS),* [3]*JSPS Research Fellowship for Young Scientists*)

Odors are useful stimuli in sleep research because they rarely cause arousal. In addition, odor has another unique characteristic, as there are large individual differences in perception. We have examined, through a series of studies, how the effects of olfactory stimulations during REM sleep on dreams differed according to individual differences in odor perception. We focused on preference and familiarity in Studies 1 and 2. In these studies, we found that the effects of odor stimulation on dreams were stronger for subjects who preferred or were familiar with the odor, and their dream became more negative. However, no such effect was found for individual difference in subjective intensity, which was examined in Study 3. Multiple regression analysis using data from these studies also confirmed that preferred or familiar odor was related to dreams becoming more negative, but this was not the case in subjective intensity. In our presentation, we will present these results and explain why preferred and familiar odor induced negative dreams and subjectively intense odor did not.

### [3P12-31]

### Recovery from systemic-inflammation-induced altered sleep is potentiated by sevoflurane preconditioning

*Tsuyoshi Nemoto[1], Yoko Irukayama-Tomobe[2], Genki Takahashi[1], Hiromu Tanaka[1], Yuki Hirose[3], Satoshi Takahashi[4], Masashi Yanagisawa[1], Takashi Kanbayashi[1,5] ([1]*University of Tsukuba,* [2]*Medical Mycology Research Center Chiba University,* [3]*Department of Anesthesiology University of Tsukuba Hospital,* [4]*Department of Anesthesiology Katsuta Hospital,* [5]*Ibaraki Prefectural Medical Centre of Psychiatry*)

*Background:* Despite extensive evidence on the organ protective effects of sevoflurane, its effect on disturbed sleep remains unclear. We hypothesized that sevoflurane preconditioning positively impacts disturbed sleep caused by systemic inflammation. *Methods:* A mouse model of lipopolysaccharide (LPS)-induced systemic inflammation was employed to investigate the effects of sevoflurane on sleep recovery. We evaluated symptoms recovery through electroencephalography/electromyography (EEG/EMG) and histological studies. The mice were exposed to 2% sevoflurane before and after peritoneal injection of LPS. The EEG/EMG were recorded for 24 h after the procedure. Brain tissue was harvested after the sevoflurane/LPS procedure and was immunostained using individual antibodies against choline acetyltransferase (ChAT) and Fos. We quantitatively analyzed the ChAT-positive and ChAT/Fos double-positive cells in the pedunculopontine tegmental nucleus and laterodorsal tegmental nucleus (PPTg/ LDTg). *Results:* Mice preconditioned with sevoflurane showed a significant recovery in rapid eye movement (REM) sleep following the LPS challenge. They also demonstrated shorter REM latency, indicating an early recovery from LPS-altered sleep. We observed more ChAT/Fos double-positive cells in the PPTg/LDTg in the sevoflurane preconditioning plus LPS group than in the LPS-only group. *Conclusions:* Sevoflurane preconditioning promotes recovery from altered sleep induced by systemic inflammation. Activation of PPTg/LDTg is considered a mechanism underlying sleep reintegration. The recovery phenomenon shows potential for clinical application in cases of sleep disturbances induced by systemic inflammation.

### [3P12-32]

### Validation experiment using in-home sleep EEG and its clinical application

*Shigeru Chiba[1,2], Yoko Suzuki[1], Takashi Abe[1], Hideaki Kondo[3], Takashi Kambayashi[1,2], Masashi Yanagisawa[1] ([1]*University of Tsukuba,* [2]*Ibaraki Prefectural Medical Center of Psychiatry,* [3]*Shunkaikai Inoue Hospital*)

To construct a system that can evaluate sleep as accurately as polymsomnografy (PSG), the gold standard for sleep evaluation, we developed an in-home EEG. We conducted a series of experiments to validate the accuracy of the device. The first study included 50 healthy subjects and verified the validity of sleep evaluation for in-home EEG using simultaneous measurements of in-home EEG, PSG, and Fitbit Charge 3, an accelerometer with HRV analysis, in the subjects. The mean ± standard deviation (SD) of the accuracy and kappa coefficient of the in-home EEG, in which the scoring result of the PSG for the correct answer, was 86.8% ± 3.8% and 0.80 ± 0.05, respectively. The lowest accuracy and kappa coefficient were 77.4% and 0.65, respectively. The accuracy in stage N1 was 59.5%, which was low, but good accuracy was obtained in the other sleep stages. The mean ± SD of the accuracy and kappa coefficient of the Fitbit was 69.5 ± 8.3% and 0.50 ± 0.14, respectively, and the accuracy and kappa coefficient of the Fitbit sleep stage scoring was significantly lower ($P < 0.05$) than those of the in-home EEG. Another empirical study of sleep assessment including 100 patients with sleep disorders of breathing (SDB) using in-home EEG, has just begun. A high proportion of patients have been diagnosed as obstructive sleep apnea syndrome (OSAS), and simultaneous measurement of their sleep using in-home EEG and PSG showed that the accuracy of in-home EEG depends largely on their severity of the disease. In patients with 5-15 AHI (apnea and hypopnea index), the accuracy of the EEG was almost as high as that in healthy subjects. On the other hand, in patients with over 30 AHI, who are considered to have severe OSAS, the accuracy was around 70%.

### [3P12-33]

### Automatic sleep stage and arousal assessment of sleep recordings from a portable IoT EEG device

*Juan Carlos Neira[1], Leo Ota[2], Kazumasa Horie[2], Masashi Yanagisawa[3], Hiroyuki Kitagawa[3] ([1]*PhD program in Humanics, University of Tsukuba,* [2]*Center for Computational Sciences (CCS), University of Tsukuba,* [3]*International Institute for Integrative Sleep Medicine (IIIS), University of Tsukuba*)

Since its inception, EEG recording and scoring has become a standard procedure for sleep research and sleep diagnosis. EEG has traditionally been recorded in a laboratory setting, as part of the Polysomnogram (PSG), but recently it is possible to record it in other spaces thanks to the advent of portable devices. EEG is scored following the R&K or AASM guidelines, which requires the visual inspection of each sleep epoch in a whole sleep recording, a very time-consuming process that is prone to error. With the interest of automatizing this process, different machine learning models have been proposed, but they are limited to specific tasks, such as stage scoring or arousal scoring but not both; therefore, limiting the possibility to evaluate other sleep variables. For this reason, we are proposing a model called Sleep Arousal Automatic Scoring (SAAS) U-Net, for the simultaneous assessment of sleep stages, arousal events and other sleep variables using the recordings of a portable IoT EEG device, with the purpose of improving the early diagnosis of sleep disorders, benefitting the users of this device and at the same time, contributing to the promotion of better sleep.

### [3P12-34]

### Metabolomic and pharmacologic analyses of brain substances associated with sleep need in mice

*Haruka Suzuki-Abe[1,2], Kazuhiro Sonomura[3], Shinya Nakata[1], Kazuya Miyanishi[1], Asmaa Mahmoud[1], Noriko Hotta-Hirashima[1], Chika Miyoshi[1], Taka-Aki Sato[2,3], Hiromasa Funato[1,6], Masashi Yanagisawa[1,4,5] ([1]*International Institutes for Integrative Sleep Medicine (WPI-IIIS), University of Tsukuba,* [2]*Research and Development Center for Precision Medicine, University of Tsukuba,* [3]*Life Science Research Center, Technology Research Laboratory, Shimadzu Corporation,* [4]*Department of Molecular Genetics, University of Texas Southwestern Medical Center,* [5]*Life Science Center for Survival Dynamics, Tsukuba Advanced Research Alliance, University of Tsukuba,* [6]*Department of Anatomy, Faculty of Medicine, Toho University*)

Sleep need is accumulated during wakefulness and dissipated during sleep. Sleep deprivation (SD) has been used as a method to investigate the molecular changes under high sleep need. However, SD induces changes reflecting not only increased sleep need but also inevitable stresses and prolonged wake state itself. The *Sleepy* mutant mice exhibit constitutively high sleep need despite sleeping longer, and have been useful as a model of high sleep need. Here we conducted a cross-comparison of brain metabolomic profiles between SD versus ad lib slept mice, as well as *Sleepy* mice versus littermate wild-type mice. 203 metabolite were quantified in total, of which 43 metabolites showed significant changes in SD, whereas 3 did in *Sleepy* mice. The large difference in the number of differential metabolites highlighted limitations of SD as methodology. The cross-comparison revealed that a decrease in betaine and an increase in imidazole dipeptides (IDs) are associated with high sleep need in both models. Furthermore, we found that central injection of IDs increased subsequent NREM sleep time, suggesting the possibility that IDs may participate in the regulation of sleep in response to homeostatic sleep need in mice.

### [3P12-35]

### The novel intracellular signaling pathways for the regulation of homeostatic sleep need

*Tomohiro Kitazono[1], Noriko Hotta[1], Kanako Iwasaki[1], Tomoyuki Fujiyama[1], Hiromasa Funato[1,2], Masashi Yanagisawa[1,3,4] ([1]*WPI-IIIS, Univ. of Tsukuba,* [2]*Dept of Anat., Fac. of Med., Toho Univ.,* [3]*Dept. of Mol. Genet., Univ. of Texas Southwestern Med. Cent.,* [4]*TARA, Univ. of Tsukuba*)

Sleep is an ubiquitously conserved behavior, and homeostatic sleep need is the fundamental element for sleep regulation. However, the molecular/cellular basis of sleep need remains unknown. Our recent study suggested that changes of sleep need can be translated into cumulative phosphorylation of certain brain proteins, and SIK3 kinase has an essential role in this regulatory mechanisms. In this study, to reveal the molecular/ cellular basis of sleep need, we aim to identify upstream and downstream components of SIK3. As a possible upstream regulator of SIK3, we focused on LKB1 kinase. We generated postnatal neuron-specific LKB1 knockout (nKO) mice, and LKB1 nKO mice showed weaker sleep pressure. Furthermore, this phenotype is recovered by constitutively active mutation of SIK3. These results suggest that LKB1 regulates sleep/wake behavior by acting upstream of SIK3. To identify the downstream signaling pathway of SIK3, we conducted in vitro substrate screening of SIK3 by kinase-oriented substrate screening (KIOSS) method (Nishioka et al., 2015) and the pathway analysis. These results suggest that Rho-GTPase signaling pathway may function downstream of SIK3 in sleep/wake regulation.

### [3P12-36]

### Natural history study of sleep disturbances in CDKL5 deficiency disorder mice

*Liqin Cao[1], Tingting Lou[1], Xin Zhang[1], Jing Ma[2], Zhiqiang Wang[2], Teruyuki Tanaka[3], Hiromasa Funato[1], Masashi Yanagisawa[1], Qinghua Liu[1,4] (*[1]University of Tsukuba, [2]Harbin Institute of Technology, [3]University of Tokyo, [4]National Institute of Biological Sciences*)

CDKL5 deficiency disorder (CDD) is an X-linked severe neurodevelopmental disorder caused by pathogenic mutations in the Cyclin-dependent kinase-like 5 (*CDKL5*) gene. Patients with CDD display a variety of clinic symptoms including early-onset seizures, intellectual disability, and autism spectrum disorder. The majority (>86%) of patients have sleep problems such as difficulty falling asleep, sleep fragmentation, frequent night awakenings, night screaming and excessive daytime sleepiness. Sleep disturbances impair neural plasticity and exacerbate illness state in patients with autism and epilepsy. However, little is known about the sleep phenotypes in *Cdkl5* mutant mice. To this end, we characterized baseline sleep and recovery sleep after sleep deprivation in young and older *Cdkl5* knock-out (KO) male mice and their wildtype littermates using electroencephalography (EEG) and electromyography (EMG) recording. At both baseline and following sleep deprivation, young and older *Cdkl5* KO mice exhibited significantly increased sleep latency, shorter sleep episode duration and frequent transitions between sleep and wakefulness compared with their littermate controls, which resemble sleep disturbances observed in human CDD patients. The results suggest that *Cdkl5* KO mouse model may be a valuable genetic model for studying sleep disruptions in CDD patients.

### [3P12-37]

### OX2R-selective orexin agonism is sufficient to suppress narcoleptic symptoms, cataplexy and wake fragmentation, without inducing drugseeking behavior in mouse models

*Hikari Yamamoto[1], Yasuyuki Nagumo[1], Yukiko Ishikawa[1], Yoko Irukayama-Tomobe[1], Tsuyoshi Nemoto[1], Hiromu Tanaka[1], Genki Takahashi[1], Tsuyoshi Saitoh[1], Hiroshi Nagase[1], Hiromasa Funato[1], Masashi Yanagisawa[1] (*[1]Tsukuba Univ. International Institute for Integrative Sleep Medicine*)

Acquired loss of orexin neurons causes a chronic sleep disorder narcolepsy-cataplexy. Narcoleptic symptoms are characterized by cataplexy and sleep/wake fragmentation. Orexin receptor agonist is expected as a mechanistic treatment of narcolepsy. However, it has been unclear whether the activation of only OX2R, or both OX1R and OX2R, is required to replace the endogenous orexin functions in the brain. To examine whether the selective activation of OX2R is sufficient to ameliorate the cataplexy and sleep/wake fragmentation, we compared the therapeutic efficacy by peptidic orexin in narcolepric model mice. Here we concluded that OX2R selective orexin agonism is sufficient to ameliorate both cataplexy and wake fragmentation without reward or reinforcing effects. These findings provide a proof-of-concept for a safer mechanistic treatment of narcolepsy-cataplexy through OX2R-selective agonism.

### [3P12-38]

### Pre-narcoleptics are more prone to suvorexant-induced cataplexy.

*Mahesh Kumar Kaushik[1], Kosuke Aritake[1], Yoan Cherasse[1], Aya Imanishi[2], Takashi Kanbayashi[1], Yoshihiro Urade[3], Masashi Yanagisawa[1,4,5,6] (*[1] International Institute for Integrative Sleep Medicine (WPI-IIIS), University of Tsukuba, [2]Akita University Graduate School of Medicine, [3]Isotope Science Center, The University of Tokyo, [4]Depart. Mol. Genet., University of Texas Southwestern Medical Center, [5]Life Science Centre, Tsukuba Advanced Research Alliance, University of Tsukuba, [6]R & D Center for Frontiers of MIRAI in Policy and Technology, University of Tsukuba*)

Orexins/hypocretins are key neuropeptides responsible for the regulation of the central arousal and reward circuits. Suvorexant, a dual orexin receptor antagonist, induces sleep in mice, dogs, and humans. Suvorexant induces sleep by reducing the orexin synthesis. We hypothesize that individuals with low brain orexin levels (pre-narcoleptics), maybe at a higher risk of cataplexy by suvorexant. Heterozygous orexin knockout ($Ox\text{-}KO^{(-/+)}$) mice could mimic a perfect pre-narcoleptic condition. As expected, the biochemical analysis showed that $Ox\text{-}KO^{(-/+)}$ mice had a significantly low level of orexin peptide as compared to wild-type mice. The behavioral analysis showed that suvorexant administration in $Ox\text{-}KO^{(-/+)}$ mice induces narcolepsy as evident by the appearance of sleep onset REM (SOREM) sleep and cataplexy (direct transition from wake to REM sleep) in a dose-dependent manner. Sleep/wake did not show major changes. Our data suggest that $Ox\text{-}KO^{(-/+)}$ mice have insufficiently low brain orexin to show behavioral narcolepsy. However, upon suvorexant administration brain orexin may further decrease, which along with the inhibition of the orexin receptors, results in the appearance of narcoleptic symptoms in $Ox\text{-}KO^{(-/+)}$ mice.

### [3P12-39]

### Orexin receptor antagonists ameliorate the symptoms of REM sleep behavior disorder in a novel mouse model and in human patients

*Mari Hondo[1], Takuro Endo[2], Ayako Mochizuki[3], Masanori Dantsuji[3], Takeshi Kanda[1], Makito Sato[1], Fusae Kawana[4], Yukiko Ishikawa[1], Kazue Suenaga[2], Shiro Nakamura[3], Manabu Abe[5], Tomio Inoue[3], Kenji Sakimura[5], Hiromasa Funato[1], Takeshi Sakurai[1], Masashi Yanagisawa[1] (*[1]International Institute for Integrative Sleep Medicine (WPI-IIIS), The University of Tsukuba, [2]Tokyo Sleep Medical Center, Sleep Clinic Chofu, [3]Department of Oral Physiology, School of Dentistry Showa University, [4]Cardiovascular Respiratory Sleep Medicine, Juntendo University, [5]Department of Cellular Neurobiology, Brain Research Institute, Niigata University*)

Rapid eye movement (REM) sleep behavior disorder (RBD), a parasomnia characterized by the loss of REM atonia, is associated with a high probability of later developing $\alpha$-synuclein diseases. Novel transgenic models of RBD are useful for promoting the discovery of new RBD therapeutics. Here we generated transgenic mouse lines lacking the glycine receptor alpha1 subunit in cholinergic neurons (ChGlyR-KO) or in a subset of somatomotor neurons (MnGlyR-KO). Both ChGlyR-KO and MnGlyR-KO mice displayed excessive body and limb movements during REM sleep, including jerking, kicking, punching, and chewing behaviors that resemble human RBD. This phenotype was ameliorated by the administration of clonazepam, a benzodiazepine often used clinically to treat RBD symptoms in human, indicating that the ChGlyR-KO mouse is a good animal model for studying RBD. We also found the dual orexin receptor antagonist DORA22 was effective for treating the RBD phenotype of ChGlyR-KO mice. Further, in human RBD patients, the clinically available orexin antagonist suvorexant significantly reduced the loss of REM atonia. Our observations indicate that orexin signal blockade could be a potential treatment for RBD symptoms.

### [3P12-40]

### Learning and memory deficit in adult dreamless mice

*Deependra Kumar[1], Tomoyuki Fujiyama[1], Takeshi Kanda[1], Chika Miyoshi[1], Miyo Kakizaki[1], Masanori Sakaguchi[1], Hiromasa Funato[1], Masashi Yanagisawa[1] (*[1]University of Tsukuba, IIIS*)

Earlier REM sleep deprivation studies in rodents showed severe consequences of loss of REM sleep on learning and memory. However, finding of those studies is likely to affect by stressful sleep deprivation procedures that are difficult to bypass. To address this issue, we used Dreamless mice, which have inherently reduced REM sleep (Funato et al., 2016). Further, we used genetically encoded tools for neural circuit dissection to relevant cellular populations at learning and memory stages in dreamless mice. We crossed dreamless mice with TRAP2 (Targeted Recombination in Active Population) and Ai27 (tdTomato) to label activated (TRAPed) cells at memory encoding, consolidation, and retrieval stages. Adult dreamless triple transgenic mice (3X: Drl -TRAP2-Ai27) and their littermates double transgenic (2X: TRAP2-Ai27) underwent a classical contextual fear conditioning paradigm. Next day when mice were placed in same context, dreamless 3X mice showed a significantly low (half) freezing percentage compared to littermates 2X mice. Histological examination of dreamless brain obtained at learning stage showed an overall low number of TRAPed cells in hippocampus, particularly in Dentate Gyrus region, indicating low hippocampal activation. Hippocampal activation is required for successful memory formation and storage, which is probably affected due to low REM sleep amount in dreamless mice.

### [3P12-41]

### NALCN in the forebrain and pons-medulla regions have distinct roles in REM sleep regulation

*Tomoyuki Fujiyama[1], Satomi Kanno[1], Noriko Hotta[1], Miyo Kakizaki[1], Aya Ikkyu[1], Ayako Mochizuki[1], Patricia Seoane-Collazo[1], Kanako Iwasaki[1], Fuyuki Asano[1], Staci Kim[1], Yousuke Tsuneoka[2], Seiya Mizuno[3], Manabu Abe[4], Chika Miyoshi[1], Takeshi Kanda[1], Masahiko Watanabe[1,6], Kenji Sakimura[4], Satoru Takahashi[3], Hiromasa Funato[1,2], Masashi Yanagisawa[1,5] (*[1]WPI-IIIS, Univ. of Tsukuba, [2]Dept of Anat, Toho Univ., [3]LARC, Univ. of Tsukuba, [4]Dept of Cell Neurobiol, Niigata Univ., [5]UT Southwestern Medical Center, [6]Dep of Anat & Embryol, Hokkaido Univ.*)

Although REM sleep (REMS) is ubiquitous in mammals, the molecular/neural mechanism of REMS regulation remains unknown. We performed a large-scale screening of ENU-mutagenized mice to identify genes regulating sleep/wake behavior, and established the Dreamless mutant pedigree exhibiting ~50% reduction in 24-h REMS amount. We identified a single nucleotide substitution specific to Dreamless mutant mice within the exon 9 of the Nalcn gene. The mutation leads to a single amino acid substitution (N315K) of the NALCN protein, a voltage-independent, non-selective leak cation channel. Introducing the same point mutation in wild-type mice through genome editing confirmed that the mutation was responsible for REMS abnormality, suggesting an important role of NALCN in REMS regulation. To elucidate the responsible brain regions and neuronal subtypes through which NALCN regulates REMS, we generated flox and FLEx (flip-excision) knock-in mice bearing Cre-dependent loss-of-function and gain-of-function Nalcn alleles, respectively. In Nalcn- FLEx mice, we confirmed that the mice crossed with a systemic Cre-expressing line Actb-iCre phenocopied the Dreamless mice on electroencephalogram and electromyogram (EEG/EMG) analyses. In Nalcn-flox mice, we confirmed a neuronal subtype-specific deletion of Nalcn mRNA in adult brain tissues. Recently we observed that NALCN has distinct roles in forebrain and pons-medulla regions for REM sleep regulation, with Foxg1-IRES-Cre or En1-Cre lines. Now we are analyzing the detailed sleep phenotype of these two Nalcn genetically-modified mice with sleep stage scoring.

### [3P12-42]

### *Sleepy* mouse as a model of idiopathic hypersomnia.

*Asmaa Elhosainy Mohamed Mahmoud[1], Haruka Suzuki-Abe[1], Mahesh Kaushik[1], Staci Jakyong Kim[1], Tsuyoshi Saitoh[1], Yukiko Ishikawa[1], Noriko Hotta-Hirashima[1], Chika Miyoshi[1], Hiromasa Funato[1], Masashi Yanagisawa[1] ([1]*University of Tsukuba*)

Idiopathic hypersomnia (IH) is a neurologic disorder characterized by excessive daytime sleepiness, long night sleep and severe sleep inertia which presents as "sleep drunkenness." Daytime sleep latency is shortened in IH patients, as typically assessed by multiple sleep latency test (MSLT). The *Sleepy* mutant mouse, a mouse pedigree with a splicing mutation in the Sik3 protein kinase gene, has longer sleep time and increased sleep need compared with wild-type mice. Therefore, we aimed to validate the *Sleepy* mouse as a mouse model for IH. In a mouse version of MSLT, *Sleepy* mice showed normal sleep latency compared with wild-type mice in the light phase and shorter sleep latency for the first 3 trials of the MSLT in the dark phase. They also showed reduced decay of EEG delta density during wakefulness, possibly reflecting an elevated sleep inertia. Then, we aimed to evaluate the effectiveness of orexin agonists in treating the sleepiness symptom of the *Sleepy* mouse. Intracerebroventricular injection of orexin-A promoted wakefulness for 2 h and 3 h after injection in *Sleepy* mice and wild-type mice, respectively. Intraperitoneal injection of the small-molecule orexin agonist YNT-185 promoted wakefulness for 2 h in *Sleepy* mice. These results indicate that *Sleepy* mouse is a valid model for idiopathic hypersomnia, and that orexin agonists are effective in treating sleepiness due to causes other than orexin deficiency.

### [3P12-43]

### Electrophysiological analysis of ion channel mutations in mice with REM sleep abnormalities

*Yuki Taira[1] ([1]*Univ. of Tsukuba, International Institute of Informatics and Systemics: IIIS*)

We will perform a functional analysis of a novel mouse family with REM sleep abnormalities that was discovered in a previous study by random mutagenesis and EEG analysis.
The gene responsible for the sleep abnormalities in this mouse family has been identified as an ion channel, but how the mutation changed the biophysical properties of the ion channel as a result of the mutation is still unknown. The purpose of this study is to characterize the biophysical properties of this mutant ion channel using the patch clamp method.

### [3P12-44]

### Sleep/wakefulness and body weight growth from infancy to adulthood in a hypersomnia model, *Sleepy* mutant mouse

*Noriko Hotta[1], Chika Miyoshi[1], Aya Ikkyu[1], Miyo Kakizaki[1], Satomi Kanno[1], Kumi Ebihara[1], Hiromasa Funato[1,2], Masashi Yanagisawa[1,3,4] ([1]*WPI-IIIS, Univ. of Tsukuba*, [2]*Dept. of Anat., Fac. of Med., Toho Univ.*, [3]*Dept. of Mol. Genet., Univ. of Texas Southwestern Med. Cent.*, [4]*TARA, Univ. of Tsukuba*)

For technical reasons, few studies have been conducted to examine sleep/wakefulness during infancy using electroencephalogram and electromyogram (EEG/EMG). Therefore, the significance of sleep during infancy remains enigmatic and there are many unresolved issues regarding sleep during postnatal development, such as the onset of sleep abnormalities observed in adult mice. Here, we developed a method to record EEG/EMG from P21 immediately after weaning to P57 every 4 days. Using this system, we examined when SIK3 *Sleepy* mutant mice (*SLP*) start to exhibit the long sleep time observed in adulthood. In addition, EEG/EMG recordings were performed on these *SLP* mutant mice and their wild-type littermates at 10-13 weeks and 30-33 weeks of age. At P21, when sleep/wake behavior was immature, there was no significant difference in NREMS time between *SLP* mutant and wild-type littermates. Subsequently, the *SLP* mice showed a constant increase in NREM sleep time from P25, while the wild type showed a slight decrease. Furthermore, weekly weight measurements from 4 to 30 weeks of age confirmed that *SLP* mice begin to become obese after the onset of hypersomnia.

### [3P12-45]

### SIK3 in different hypothalamic areas mediates whole-body energy balance

*Patricia Seoane-Collazo[1,2], Masashi Yanagisawa[1], Hiromasa Funato[1,3] ([1]*Tsukuba Univ.*, [2]*Santiago de Compostela Univ.*, [3]*Toho Univ.*)

In addition to its role in determining sleep need, Salt-inducible kinase 3 (Sik3) is likely to regulate energy homeostasis since mice mutant for exon 13 of the Sik3 gene (*Sleepy*) display an obese phenotype. The main objective of this project is to identify the role of SIK3 in the central nervous system in the regulation of energy balance. To achieve this, neuron-specific mutant mice lines for the *Sleepy* gene were generated, as well as viral genetic approaches in Sik3-ex13 floxed mice were used to identify the brain regions involved in SIK3 actions. Our data showed that Vglut2-specific *Sleepy* mice also show an obese phenotype, suggesting dependency of glutamatergic signaling. SIK3 gain of function, by AAV injection in Sik3-ex13 floxed mice, in the ventromedial nucleus of the hypothalamus induced feeding-independent increase in body weight, associated with altered glucose homeostasis. On the other hand, SIK3 in the paraventricular nucleus of the hypothalamus caused hyperphagia and reduced energy expenditure leading to an obese phenotype. Overall, these data suggest a role of SIK3 in the regulation of energy metabolism in a nucleus-specific manner.

### [3P12-46]

### Where does Sleepy mutation of SIK3 cause sleep phenotypes?

*Kanako Iwasaki[1], Tomoyuki Fujiyama[1], Shinya Nakata[1], Minjeong Park[1], Chika Miyoshi[1], Noriko Hotta-Hirashima[1], Aya Ikkyu[1], Miyo Kakizaki[1], Yukiko Ishikawa[1], Fumihiro Sugiyama[2], Seiya Mizuno[2], Manabu Abe[3], Kenji Sakimura[3], Satoru Takahashi[2], Hiromasa Funato[1,4], Masashi Yanagisawa[1,5,6] ([1]*International Institute for Integrative Sleep Medicine (WPI-IIIS), University of Tsukuba*, [2]*Laboratory Animal Resource Center, University of Tsukuba*, [3]*Department of Animal Model Development, Brain Research Institute, Niigata University*, [4]*Department of Anatomy, Faculty of Medicine, Toho University*, [5]*Department of Molecular Genetics, University of Texas Southwestern Medical Center*, [6]*Life Science Center, Tsukuba Advanced Research Alliance, University of Tsukuba*)

Recently, we found a gain-of-function *Sleepy* (*Slp*) mutation in the *Sik3* gene, which produces the mutant SIK3(SLP) protein, increases sleep amount and non-REM sleep (NREMS) EEG delta density, an index of sleep need. However, it remains to be elucidated where SIK3(SLP) increase sleep amount and NREMS EEG delta density, since SIK3 is expressed in various tissues.
Here, we investigated whether SIK3(SLP) in mature neurons is sufficient for the sleep phenotypes of *Sleepy* mutant mice with $Synapsin1^{CreERT2}$; $Sik3^{Sleepy\text{-}flox}$ mice. Tamoxifen administrated $Synapsin1^{CreERT2}$; $Sik3^{Sleepy\text{-}flox}$ mice exhibited increased NREMS time and NREMS EEG delta density. It suggested that SIK3 plays roles regulating sleep amounts and sleep need in mature neurons.
Furthermore, we explored neural populations which increase NREMS time upon SIK3(SLP) expression with AAV vectors. We found that SIK3(SLP) expression in medial parts of hypothalamus increased NREMS, but not NREMS EEG delta density. It implies that neural populations that increase NREMS and NREMS EEG delta power in *Sleepy* mutant mice are not identical.

### [3P12-47]

### *Sik3* regulates sleep need via glutamatergic neurons in cerebral cortex

*Fuyuki Asano[1], Tomoyuki Fujiyama[1], Chika Miyoshi[1], Tomohiro Kitazono[1], Noriko Hotta-Hirashima[1], Miyo Kakizaki[1], Aya Ikkyu[1], Satomi Kanno[1], Shinya Nakata[1], Nodoka Asama[1], Seiya Mizuno[2], Fumihiro Sugiyama[2], Satoru Takahashi[1,2], Hiromasa Funato[1,3], Masashi Yanagisawa[1,4,5] ([1]*International Institute for Integrative Sleep Medicine, University of Tsukuba*, [2]*Laboratory Animal Resource Center, Transborder Medical Research Center, University of Tsukuba*, [3]*Department of Anatomy, Faculty of Medicine, Toho University*, [4]*Life Science Center, Tsukuba Advanced Research Alliance, University of Tsukuba*, [5]*Department of Molecular Genetics, University of Texas Southwestern Medical Center*)

SIK3 is a member of AMP-activated protein kinase family, implicated in sleep need and circadian rhythm regulation. The gain-of-function *Sleepy* mutation in the *Sik3* gene increases NREM time and EEG delta power during NREMS with comparable period length. Most of systemic *Sik3* null mice die neonatally, but the few survived mice, while suffering from severe growth retardation and malnutrition, exhibit longer circadian period lengths. It remains unknown as to whether endogenous SIK3 is involved in sleep/wake regulation. Here, we established various neuron subtypes specific SIK3 deficient mice and examined the sleep/wakefulness behavior. SIK3 deficiency in glutamatergic neurons in cerebral cortex decreased NREM sleep amounts and delta power during NREM sleep, which is a marker of sleep need, throughout the day. We also confirmed that SIK3 deficiency in GABAergic neurons showed a phase delay in sleep/wake pattern and longer period length. Our results indicate that SIK3 regulates homeostatic sleep need through glutamatergic neurons in the cerebral cortex, and sleep need regulation was independent from the regulation of circadian rhythms by endogenous SIK3.

### [3P12-48]

### Molecular mechanisms for SIK3(*Sleepy*)-mediated sleep/wake regulation

*Shinya Nakata[1], Haruna Abe-Komiya[1], Tomoyuki Fujiyama[1], Chika Miyoshi[1], Aya Ikkyu[1], Fuyuki Asano[1], Seiya Mizuno[2], Fumihiro Sugiyama[2], Satoru Takahashi[2], Hiromasa Funato[1,3], Masashi Yanagisawa[1] (*[1]WPI-IIIS, University of Tsukuba, [2]Faculty of Medicine, University of Tsukuba, [3]Department of Anatomy, Toho University*)

Sleep is regulated by sleep need in a homeostatic manner. We recently identified *Sik3* as a novel gene regulating sleep/wakefulness through forward-genetics approach. The pedigree, *Sleepy*, which has a splicing mutation *Sik3 (Slp)* in *Sik3* resulting in the skipping of exon 13, exhibits increased sleep need and prolonged sleep time. On the other hand, the SIK3 kinase activity is tightly regulated by phosphorylation of T221 in the kinase domain T-loop. Interestingly, the phosphorylation of T221 is increased in wild-type mice after sleep deprivation, indicating that SIK3 kinase activity increases in mice with a higher sleep need. However, how SIK3 kinase activity is involved in sleep regulation remains unknown.

Here, we report the functional analysis of SIK3 kinase activity for sleep/wake regulation using mice expressing a non-phosphorylatable T221A or phosphomimetic T221E mutant of SIK3(WT) or SIK3(SLP), respectively. Our EEG-EMG-based sleep analysis of those mutant mice and biochemical assays of those mutant SIK3 proteins indicate that SIK3 kinase activity regulates sleep need and is required to increase sleep amount following another functional alternation such as protein-protein interaction.

### [3P12-49]

### Sleep/wake behavior of mice lacking PKA phosphorylation site in SIK3

*Minjeong Park[1], Takato Honda[1], Chika Miyoshi[1], Tomoyuki Fujiyama[1], Miyo Kakizaki[1], Aya Ikkyu[1], Seiya Mizuno[2], Satoru Takahashi[2], Hiromasa Funato[3], Masashi Yanagisawa[1] (*[1]International Institute for Integrative Sleep Medicine, University of Tsukuba, [2]Laboratory Animal Resource Center, University of Tsukuba, [3]Department of Anatomy, Toho University School of Medicine*)

We previously identified a kinase, SIK3, as an important sleep regulator through screening of randomly mutagenized mice. Mice that express mutant SIK3 lacking the 52 amino acids encoded by exon 13 showed a decrease in wake time and an increase in NREM sleep time. SIK3 is an AMPK-family protein kinase containing a well-conserved protein kinase A (PKA)-phosphorylation site, serine 551. The skipping of exon 13 results in a deletion of 52 amino acids including S551. Also, *Sik3 S551A* knock-in mice showed reduced total wake time and increased sleep need. These results suggest that the existence of S551, a PKA recognition site, is crucial for the normal sleep/wake regulation and maintenance of daily sleep need. In SIK3, there are three PKA recognition sites, threonine 469, serine 551, and serine 674. To examine whether the phosphorylation of T469 and S674 of SIK3 is required for proper sleep/wake behavior, we generated mutant mice in which SIK3 T469 and SIK3 S674 were substituted by alanine through the CRISPR/Cas9 method. *Sik3 T469A* mice showed increased NREM sleep time and NREM sleep delta power, an index for sleep need. *Sik3 S674A* mice showed no changes in NREM sleep time and NREM sleep delta power. These findings indicate the PKA recognition sites of SIK3, especially T469 and S551 are required for the regulation of sleep/wake behavior.

### [3P12-50]

### Loss of canonical *Hdac4* signaling leads to disregulated NREMS

*Staci Jakyong Kim[1], Noriko Hotta-Hirashima[1], Nodoka Asama[1], Matsuoka Taeko[1], Tomoki Tsukamoto[1], Aya Ikkyu[1], Miyo Kakizaki[1], Satomi Kanno[1], Seiya Mizuno[2], Satoru Takahashi[2], Chika Miyoshi[1], Hiromasa Funato[1,4], Masashi Yanagisawa[1,3,5] (*[1]Intl. Inst. for Integrative Sleep Med. (WPI-IIIS), [2]Transborder Med. Res. Ctr., Univ. of Tsukuba, [3]Life Sci. Center, Tsukuba Advanced Res. Alliance, Univ. of Tsukuba, [4]Dept. of Anatomy, Fac. of Med., Toho Univ., [5]Dept. of Mol. Genet., Univ. of Texas Southwestern Med. Ctr.*)

Sleep is observed universally across various species from nematodes to mammals and the properly maintained sleep is directly linked to the general well-being of the organism. Much advances has been made in the identification of the distinct groups of neuronal population that are involved in the transitions between sleep and wake states. These networks of neurons can drive vigilance stage switches and changes in neuronal activity related to sleep and wakefulness. Despite the growing interest and findings in the maintenance of sleep/wakefulness, the intracellular regulatory mechanism of the response to sleep need changes remains largely unknown.

Our EEG/EMG- based forward genetics approach in mice sleep/wake behavior has successfully identified several sleep regulatory genes. *Sleepy2* is one of such gene locus with a single nucleotide change in the splice acceptor site of *Hdac4* exon23 that leads to out-of-frame mutation of encoded protein. The heterozygous mutant mice with the resulting splice variants showed increase in both daily non-REM sleep (NREMS) time and slow-wave activity during NREMS. Interestingly, a phosphodeficient mutant type mice showed a complete opposite trend in sleep/wake behavior. These mice showed increase in daily wake time and significant decrease in the slow-wave activity during NREMS. These results suggest canonical signaling of *Hdac4* gene is involved in the regulation of NREMS.

For further understanding of specific neuronal network where the identified sleep regulators may function, we employed a neuron type-specific manipulation engineered by Cre-loxP recombination. From the comparison of various Cre reporter lines, distinct neuronal types were identified to regulate the NREMS time and the slow-wave activity. It is plausible Hdac4 signaling modulates the amount and quality of sleep discretely neuronal network in type- and region-specific manner.

### Student Presentation

### Student Presentation 1

March 16(Wed), 11:15 - 12:15, Room H

### [ST01-01]

### The effect of TRPA1 on the respiratory regulation in the Pons

*Naoko Masutani[1], Yuki Kousaka[1], Akiko Arata[1] ([1]*Dept. of Physiology, Div. of Physiome, Hyogo College of Medicine*)

TRPA1, one of the temperature-sensitive TRP channels, is involved in a variety of physiological processes, including nociception and mediation of cytokine release. Moreover, TRPA1 is known to coexist with TRPV1 that concerned pain and hot temperature. In our previous experiments with TRPV1, we reported that capsaicin, a TRPV1 agonist, facilitated respiratory rate in the pons-medulla-spinal cord preparation isolated from neonatal rat. We therefore tested the effects of TRPA1 which coexists with TRPV1 and responsible for the same pain sensation on the pain-respiratory reflex. First, we compared the respiratory responses of TRPA1 in the with pons and without pons preparation. The cinnamaldehyde (CNA), a TRPA1 agonist, decreased the respiratory rate and amplitude with pons preparation, while the respiratory rate did not change in the without pons. The optical imaging response was found the parabrachial nucleus of the pons when sensory afferent stimulation of the dorsal root of the spinal cord at the C8 level. Parabrachial nucleus is the inspiratory-expiratory phase switching center using GABAergic system. So, we examined the involvement of the GABAergic inhibition to respiratory depression using TRPA1 in the parabrachial nucleus. Since the respiratory inhibitory effect was blocked by the GABAA blocker bicuculline, we hypothesized that TRPA1 is involved in the GABA inhibitory mechanism in the parabrachial nucleus and may have a descending inhibitory effect in the pons in response to painful stimulation.

### [ST01-02]

### Differentiation of hypothalamic paraventricular neurons from direct reprogramming by gene transfer using mouse ES cells

*Yoshinari Mera[1], Shunya Tsukamoto[1], Tsukushi Suzuki[1], Miho Kawata[1], Yu Kodani[1], Kanako Saito[1], Akira Nakashima[2], Toshiki Kameyama[1], Hiroshi Nagasaki[1] ([1]*Department of Physiology Fujita Health University school of medicine*, [2]*Department of Physiological Chemistry Fujita Health University school of medicine*)

The hypothalamus is the command center for hormone secretion. In the hypothalamus, cell bodies specialized for each role are assembled to form neural nuclei, which form a network with each other.The hypothalamus is involved in the regulation of pituitary hormones as well as the secretion of AVP and oxytocin. When the hypothalamus is disturbed, central enuresis, sleep disorders, eating disorders and dysfunction of the anterior pituitary gland occur. Since the hypothalamus is directly related to the maintenance of human life and it is difficult to collect cells directly as specimens, the direct differentiation of ES cells into the hypothalamic neurons may contribute greatly to the field of regenerative medicine for enuresis. Several differentiation-inducing factors have been identified the hypothalamus,and each of them expresses specifically in the developing hypothalamus. However, The function of all the genes remains unknown. We believe that elucidating the role of these genes in the differentiation of hypothalamic neurons in early development will greatly contribute to understand the mechanism of brain development and regenerative medicine.We attempted to directly differentiate mouse Embryonic stem cells into hypothalamic neurons by introducing a combination of specific transcription factor.

### [ST01-03]

### Abnormal neuronal activity in the basal ganglia of drug dependence model mice.

*Yuya Taguchi[1], Atsushi Nambu[2], Hiromi Sano[2], Satomi Chiken[2] ([1]*Nagoya City University*, [2]*Division of System Neurophysiology, National Institute for Physiological Sciences*)

To clarify the neuronal mechanism underlying abnormal behaviors of drug addiction, we recorded neuronal activity in the basal ganglia (BG) of methamphetamine (METH)-dependent mice during the awake state, especially in the external segment of the globus pallidus (GPe) and substantia nigra pars reticulata (SNr), the connecting and output nuclei of the BG. In naive mice, cortical stimulation induced a triphasic response composed of early excitation, inhibition, and late excitation in the GPe and SNr. Locomotor activity was increased after repeated METH administration. In METH-dependent mice after acute METH administration, cortically induced late excitation was reduced in the GPe and SNr. Their firing rates were increased, and their firing patterns became bursty. Without acute METH administration, late excitation was mildly reduced as well. Cortically induced late excitation in the SNr is mediated by the cortico-striato-GPe-subthalamo-SNr indirect pathway, which may stop movements. In drug dependent state, neurotransmission through the indirect pathway is disrupted, and movements cannot be stopped, resulting in abnormal behaviors, such as drug-seeking behaviors.

### [ST01-04]

### Dorsal Raphe 5-HT Neurons Exhibits Oscillatory Activity during NREM sleep in vivo

*Yo Nakahara[1], Tomonobu Kato[1], Norio Takata[1], Kenji Tanaka[1] ([1]*Div. Brain Sci., Inst. Advanced Med. Res., Keio Univ. Sch. Med.*)

Introduction
The dorsal raphe (DR) serotonergic (5-HT) neurons modulate various physiological activities including sleep-wake regulation. Previous studies using single-unit recordings showed that most 5-HT neurons are active during wakefulness, less active during NREM sleep, and silent during REM sleep. However, the dynamics of the population activity rather than the single-cell activity should be addressed prior to the understanding of how DR 5-HT neurons affects entire brain activity and sculpts sleep-wake structure. In particular, we questioned whether the population activity of DR 5-HT neurons could decrease linearly, in step-by-step manner, or even in an oscillatory manner during NREM sleep.
Method
We used transgenic mice that express the $Ca^{2+}$ indicator YC-Nano50 specifically in 5-HT neurons. After optic fiber implantation (AP, -4.3 mm; ML, 0.0 mm; and DV, 3.0 mm) and EEG/EMG electrode settlement, mice were allowed to recover for at least 5 days. Mice was then habituated 1 hour per day in the recording chamber for at least 2 days until REM sleep was observed. During recording sessions, mice was connected to a ratiometric fiber photometry system to monitor the population activity of DR 5-HT neurons. EEG and EMG signals were also recorded for sleep-wake analysis.
Result
We confirmed that the population activity of DR 5-HT neurons was highest during wakefulness (e.g., basal level), and lowest during REM sleep, consistent with previous data from single-unit recordings. We found an oscillatory DR 5-HT neuron activity during NREM sleep; the bottom level of each wave gradually went down but each wave always ended by bouncing back to the basal level. If the wave went down and stayed at the bottom, REM sleep was initiated. If the wave bounced back and did not go down again, mice woke up. Interestingly, during NREM sleep, we found transient and periodical wideband EEG power surge, and that such EEG power surge coincided with the decreased population DR 5- HT neuron activity.
Discussion
We succeeded in identifying how the population activity of 5-HT neurons would decrease during NREM sleep, which turned out to be in an oscillatory manner. Also, it became clear that the population activity of DR 5-HT neurons had inverse correlation with wideband EEG power, implying that the sleep state during NREM sleep is not static but is constantly fluctuating in accordance with the 5-HT oscillation. To further investigate the causal relationship between DR 5-HT neuronal activity and sleep-wake regulation, we plan to manipulate the population activity of these neurons during NREM sleep by optogenetics.

### [ST01-05]

### Induction of mouse ES cells into hypothalamic ventral medial nucleus and arcuate nucleus nerves by gene transfer of transcription factors

*Shunya Tsukamoto[1], Yoshinari Mera[1], Tsukushi Suzuki[1], Miho Kawata[1], Yu Kodani[1], Kanako Saito[1], Akira Nakashima[2], Toshiki Kameyama[1], Hiroshi Nagasaki[1] ([1]*Department of Physiology, Fujita Health University School of Medicine*, [2]*Department of Physiological Chemistry, Fujita Health University School of Medicine*)

The hypothalamus is a small region located in the diencephalon, but has many functions to maintain homeostasis in the body as the center of the autonomic nervous system. Among the hypothalamic neuronal nuclei, the ventral medial nucleus (VMN) and the arcuate nucleus (ARC) especially regulate feeding behavior and energy metabolism. These nuclei have been the focus of attention as targets for drugs related with energy metabolism and hypertension. To differentiate VMN and ARC neurons *in vitro* would lead to a better understanding of the complex functions of the hypothalamus, and open the possibility of regenerative medicine for hypothalamic-pituitary dysfunction.SFEBq and monolayer culture methods have been used to induce neuronal differentiation from embryonic stem cells (ES cells) in vitro. Though such organoid culture methods can differentiate multiple types of neurons and glia consisting of neuronal nuclei, it is difficult to differentiate specific subtypes of the neuron.In this study, we have selected some transcription factors related to the differentiation of VMN and the ARC, and transferred them to mouse ES cells. We would like to report some result of the combinations of transcription factors and culture conditions to efficiently differentiate ventral hypothalamus neurons. We hope this study will contribute to the scientific and therapeutic researches on hypothalamus.

### Student Presentation 2

March 16(Wed), 11:15 - 12:15, Room I

### [ST02-01]

### Molecular Mechanisms of Sugar-Induced Modulation of Salt Preference in *C. elegans*

*Yusuke Umemura[1], Yutaro Ueoka[1], Chihiro Uchiyama[2], Risshun Chin[2], Keita Katae[2], Yuichi Iino[2], Masahiro Tomioka[2] ([1]*Department of Biophysics and Biochemistry, Faculty of Science, The University of Tokyo,* [2]*Department of Biological Sciences, Graduate School of Science, The University of Tokyo*)

It is important for animals to modulate their behavior depending on past experiences to seek optimal environments. To achieve this, they first need to get the information of ambient conditions, and then to learn whether the conditions are favorable or not. In this sense, sensory reception and memory formation are both critical for behavioral plasticity. We have reported that the nematode *Caenorhabditis* elegans shows attraction toward sodium ions ($Na^+$), whereas this response disappears after cultivation in the absence of $Na^+$. Here, we found that the nematode shows negative preference for $Na^+$ after cultivation in the presence of glucose, which is sensed by the same sensory neuron ASEL as $Na^+$. Although various behaviors such as thermotaxis and chemotaxis are largely regulated by PKC-1, a protein kinase C-$\varepsilon$ isotype, PKC-1 is found to play a partial role in the modulation of $Na^+$ preference. This indicates that an unknown mechanism for learning underlies this behavioral plasticity. Besides, it is unclear how glucose is received in the ASEL neuron. To discover novel molecules required for glucose reception and memory formation, we conducted a forward genetic screening for mutants deficient in this behavioral plasticity. We have obtained a few mutants and are currently working to identify the causal genes for their mutant phenotypes.

### [ST02-02]

### Low-birthweight rat due to embryonic undernutrition-changes in body composition after fasting-refeeding

*Yuki Morita[1], Yoshihiko Kakinuma[1], Takahiro Nemoto[1] ([1]*Dept. physiology, Nippon Medical School* )

Background & Aim: According to the thrifty phenotype hypothesis proposed by Hales and Baker (*Br Med Bull*, **60**, 5-20, 2001), birth at low-bodyweight due to trade-offs that alter its metabolic and endocrine systems when exposed to undernutrition. Acquire a thrifty phenotype and become a small body size. The thrifty phenotype favors survival under oligotrophic conditions, but it is thought that the risk of developing disease increases due to the mismatch between the thrifty TAISHITSU and the eutrophic environment. However, low-birthweight infants do not necessarily exhibit obesity, and the characters of the thrifty phenotype are not fully understood. Therefore, we generated embryonic malnutrition model rats and investigated changes in bodyweight and body composition after fast and re-feeding. METHODS: Low-birthweight rats (LC) with embryonic malnutrition were produced according to previously reported (*Sci Rep*, **10**, 1339). Male rats 10-12 weeks old were reared in individual cages and refeeding experiments were performed. In this experiment, they were fasted for 48 hours under free drinking water and then fed a standard diet *ad libitum*. Body composition was measured by an impedance method, and blood corticosterone and IGF-1 concentrations were measured using ELISA kits. RESULTS: The 24-hour fast reduced the bodyweight of the control rats by 8.9 ± 0.9% compared to before the fasting, while the LC decreased by 7.04 ± 0.6%, which was significantly lower than controls. Furthermore, at the 48-hour fast, the control body weight was significantly reduced by 13.9 ± 1.0% compared to before the fast, while the LC was significantly reduced by 10.6 ± 0.7%. After 48 hours of re-feeding, the bodyweight of control rats recovered to 98.4 ± 1.5% before fasting, while LC remained at 94.3 ± 1.1%. At this time point, the body fat percentage was 39.9 ± 5.4% in the control, while the LC was 38.9 ± 2.1%, and there was no difference between the two groups. Blood corticosterone levels were significantly higher in LC and significantly lower in IGF-1 than in controls. Conclusion: We showed here that our model rats due to undernutrition during the embryonic period may have a thrifty TAISHITSU that is not easy to gain lean weight but is difficult to lose weight. We are going to investigate skeletal muscle remodeling after refeeding in our model rats.

### [ST02-03]

### Postexercise cooling promotes p38 MAPK-mediated mitochondrial Drp1 (Ser616) phosphorylation in rat skeletal muscle.

*Taiki Kudo[1], Tatsuya Sato[1], Hiroyori Fusagawa[1,2], Hiroya Yamazaki[1], Nobutoshi Ichose[1], Izaya Ogon[1,2], Noritsugu Tohse[1] ([1]*Department of Cellular Physiology and Signal Transduction, Sapporo Medical University School of Medicine,* [2]*Department of Orthopedic Surgery, Sapporo Medical UnivSapporo Medical University School of Medicine*)

**Background**: Recent studies have raised concerns that cooling of muscles after exercise may be associated with delayed recovery from muscle fatigue with impaired metabolic adaptations. Although mitochondria are responsible for aerobic energy metabolism, it remains unclear whether postexercise cooling promotes phosphorylation of Dynamin-related protein-1 (Drp1), which regulates mitochondrial fission. In the present study, we examined the effects of temperature intervention on Ser616 phosphorylation of Drp1 in rats after endurance exercise. **Methods**: Eight-week-old male Wistar rats were exercised on a treadmill for 30 min, and after exercise, the lower limbs were subjected to temperature intervention in three groups: room temperature (25℃), cooling (16℃ water), and warming (42℃ water) for 30 min under isoflurane anesthesia. Plantaris muscles were collected immediately after euthanasia. The phosphorylation status of Drp1 and that of its upstream regulators were assessed by immunoblots. **Results**: Compared to resting, the Ser616 phosphorylation level of Drp1, which enhances mitochondrial fission, increased after exercise in plantaris muscles. Cooling intervention further increased the Ser616 phosphorylation level of Drp1, while warming intervention did not increase its phosphorylation level. In contrast to Ser616, Ser637 phosphorylation of Drp1, which inhibits mitochondrial fission, was not altered by exercise or temperature intervention. Finally, Thr180/Tyr182 phosphorylation level of p38 MAPK, which is known to be induced by exercise and cold stimulation and can promote Ser616 phosphorylation of Drp1, was increased by postexercise cooling. **Conclusions**: The findings indicate that postexercise cooling promotes Ser616 phosphorylation of Drp1 with activated p38 MAPK signaling in rat skeletal muscle, possibly leading to excessive mitochondrial fission and impaired metabolic adaptation.

### [ST02-04]

### Roles of spinal GABA in sex differences of the ghrelin agonist-induced enhancement of colorectal motility in rats

*Yuta Uto[1], Tomoya Sawamura[2], Natsufu Yuki[2], Takahiko Shiina[2], Hikaru Hashitani[1], Yasutake Shimizu[2] ([1]*Nagoya City University,* [2]*Gifu University*)

Ghrelin agonists (GA) are known to enhance the colonic motility by acting at the spinal defecation center. However, since the function of the defecation center display sex differences, it remains to be explored whether the existing knowledge obtained from male rats may also be applicable to females. This study aimed to elucidate if GA may enhance the colonic motility in female rats. In anesthetized rats, saline was infused via a cannula that was inserted into the distal colon and drained from another cannula that was inserted into the anus. Colonic motility was evaluated by measuring change in the colonic luminal pressure, while the fluid volume expelled by colonic peristalsis were measured. The drugs were administered either intravenously (iv) or intrathecally (it). A GA (5-20 mg/kg, iv) dose-dependently enhanced the colonic motility in males, while 10 mg/kg of the agonist was required in females. Since the function of the defecation center is considered to be suppressed by GABA in females, effects of bicuculline, a $GABA_A$ receptor inhibitor (1 nmol, it), on the GA-induced enhancements of the colonic motility were examined. In bicuculline-treated female rats, the GA (5 mg/kg) was capable of enhancing the colonic motility. Effect of GA on the colonic motility exhibited sex differences that appears attributable to the difference in GABA-induced inhibition at the spinal defecation center.

### Student Presentation 3

March 17(Thu), 10:45 - 11:45, Room H

### [ST03-01]

### Characteristics of sarcomere formation and expression patterns of myofibrillar components in rat embryonic heart primordium after heartbeat initiation.

*Hiroya Yamazaki[1], Nobutoshi Ichise[1], Tatsuya Sato[1], Taiki Kudo[1], Hiroyori Fusagawa[1,2], Izaya Ogon[1,2], Noritsugu Tohse[1] ([1] *Department of Cellular Physiology and Signal Transduction, Sapporo Medical University School of Medicine,* [2] *Department of Orthopedic Surgery, Sapporo Medical UnivSapporo Medical University School of Medicine*)

**Background**: Sarcomeres are essential structures in striated myocytes including cardiomyocytes and are closely related to excitation-contraction coupling and muscle force production. However, the association between sarcomere assembly and expression patterns of myofibrillar components in embryonic hearts at around the time of the initial heartbeat remains unclear. **Methods**: We divided heart primordia in embryos of Wistar rats into three groups: heart primordium before and after heartbeat initiation at embryonic day 10.0 (E10.0) and heart primordium at E11.0. Myofibrils and sarcomere structures were observed by a transmission electron microscope, and expression levels of genes associated with myofibrillar components were assessed by microarray analysis. **Results**: There were no typical structures of sarcomeres in both the heart primordium before and that after the initiation of heartbeat at E10.0. Only myofibril-like structures were scattered in cells of the heart primordium after heartbeat initiation at E10.0, while sarcomere structures with obvious Z-lines were observed in the heart primordium at E11.0. Consistent with these structural changes, expression levels of genes associated with major components of thick and thin filaments were gradually increased with development, and expression levels of $\alpha$-actinin-4, desmin, and myomesin-1, which are major constituent molecules of Z-lines, were increased only in the heart primordium at E11.0 compared with those before and after heartbeat initiation at E10.0. **Conclusions**: The findings indicate that sarcomeres are not necessary for the initial heartbeat but are formed in the process of subsequent cardiogenesis with expression of genes of the constituent molecules of Z-lines.

### [ST03-02]

### Empagliflozin, a sodium glucose cotransporter 2 inhibitor, reduces acute stretch-induced ROS production in mice ventricular cardiomyocytes

*Shuta Kanai[1], Masamichi Itakura[1], Yumiko Chiba[2], Gentaro Iribe[2] ([1] *School of Medicine, Asahikawa Medical University,* [2] *Department of Physiology, Asahikawa Medical University*)

Myocardial acute stretch increases NADPH oxidase (NOX) 2-derived ROS production in ventricular cardiomyocytes. It has been also reported that cytosolic ROS levels are reduced by acute administration of empagliflozin (EMPA), a sodium glucose cotransporter 2 (SGLT2) inhibitor, in diabetes mellitus cardiomyocytes. Herein, we hypothesized that acute stretch-induced increase in ROS production is mediated by sodium myo-inositol cotransporter 1 (SMIT1), which is one of the SGLT subtypes expressed in the heart. To test this hypothesis, ventricular cardiomyocytes were enzymatically isolated from 9-15 week old mice heart. Isolated cells were exposed to 8-10 % axial stretch using carbon fibers, attached to both cell ends. Cellular ROS production was estimated using 2'-7'dichlorofluorescein (DCF). We found EMPA significantly suppresses acute stretch-induced ROS production. To examine whether SMIT1 stimulation without stretch reproduces the response to stretch, we applied myo-inositol (MI) instead of stretch. Application of MI increased ROS production immediately, and the response was abolished in the presence of EMPA. The present results suggest that acute stretch-induced ROS production is mediated by SMIT1.

### [ST03-03]

### Maternal administration of resveratrol constricted the rat ducts arteriosus in fetuses.

*Masashi Kogo[1], Daiki Seya[1], Takahiro Inoue[1], Toru Akaike[1], Susumu Minamisawa[1] ([1] *The Jikei University School of Medicine*)

**Background**: Previous case reports have demonstrated that intake of resveratrol, a kind of polyphenol, during pregnancy can induce premature constriction of the ductus arteriosus (DA) in the fetus.
**Purpose**: We aimed to investigate the mechanism how resveratrol intake during pregnancy promoted premature constriction of the DA.
**Methods**: First, we administered resveratrol to pregnant rats at 50mg/kg, once a day, for four days (from 17 to 20 day) by intraperitoneal administration. We then measured the diameter ratio of the DA to the aorta at 20 days with Image J by rapid whole-body freezing method. Second, we added resveratrol (10, 50, 100 $\mu$M) to cultured DA smooth muscle cells, and we took RNA from them. Using qPCR analysis, we determined the expression levels of cyclooxygenase2 (Cox2) (involved in production of prostaglandin $E_2$) and EP4(prostaglandin receptors type 4 expressed in DA).
**Result**: We found that maternal administration of resveratrol constricted DA in fetal rats(the DA/Ao ratio was 0.80 in the control group and 0.54 in the resveratrol group. p<0.0001). The qPCR analysis revealed that the expression levels of Cox2 and EP4 mRNAs were significantly decreased in the resveratrol group.
Conclusion: Our results suggested that maternal administration of resveratrol decreased the expression levels of Cox2 and EP4 mRNAs in the fetal DA, which may contribute to the premature constriction of the DA.

### [ST03-04]

### Wild-type troponin T overexpression on troponin T mutant-induced dilated cardiomyopathy partially rescued its phenotypes.

*Hirofumi Maetani[1], Yuya Yamaguti[1], Jun Tanihata[1], Shunsuke Baba[1], Sachio Mormoto[2], Susumu Minamisawa[1] ([1] *The Jikei University School of Medicine,* [2] *International University of Health and Welfare School of Health Sciences at Fukuoka*)

**Introduction**
Dilated cardiomyopathy (DCM) is characterized by cardiac dilation and pump failure. We reported that overexpression of normal human TNNT2 in cardiac troponin T (TNNT2) amino acid mutation (ΔK210) knock-in mice (DCM model mice) apparently prolonged the lifespan of DCM mice. However, in our last report, there was no significant improvement in cardiac function or left ventricular wall thickness. Therefore, we hypothesized that the improvement in arrhythmia, the main cause of death in DCM mice, might have extended their lifespan.
**Methods and Results**
First, we generated human TNNT2 overexpression mice (hTNNT2 Tg: Tg) and confirmed that human TNNT2 was overexpressed in hTNNT2 Tg mice without any adverse effects on the heart. Then, we mated Tg mice with DCM mice to generate Tg/DCM mice. The number of severe arrhythmias was decreased in the Tg/DCM mice compared with DCM mice.
**Conclusion**
The results suggest that overexpression of wild-type TNNT2 in DCM mice partially improved arrhythmia. We need to increase the number of analysis data.

### [ST03-05]

([OP11-05])

### The space flight induces the morphological changes of the lipid droplet in the liver hepatocyte of mouse.

*Takanobu Haraguchi[1], Hiroki Bochimoto[1], Daisuke Kondoh[2], Susumu Minamisawa1 ([1] *Division of Aerospace Medicine, Department of Cell Physiology, The Jikei University School of Medicine,* [2] *Laboratory of Veterinary Anatomy, Obihiro University of Agriculture and Veterinary Medicine*)

**Abstract**
**Background**: Although space flight affects the lipid metabolism, it is equivocal whether microgravity directly causes the changes. To solve the problem, we analyzed the ultrastructural changes occurred in the lipid droplet (LD) in hepatocytes reflecting with their lipid metabolism of mice on board the international space station (ISS).
**Methods**: Six of C57BL/6 J male mice were kept under microgravity (MG) or on artificial earth-gravity by using centrifugation cages (AEG) in ISS for 35 days. In addition, 6 mice were kept on ground as control. Two days after landing, all mice were euthanized and dissected, the liver tissues were excised and fixed by fixative of 4 % paraformaldehyde, and were morphologically analyzed by electron microscopy.
**Results**: The number of LD in hepatocytes were increased after spaceflight under both of MG and AEG groups compared with control group. However, the LD size of hepatocytes was increased in only MG group.
**Conclusion**: Our data suggest that the non-microgravity-related stress of space flight increased the number of LD in hepatocytes. Additionally, exposure to artificial earth-gravity may reduce the risk of the perturbation of lipid metabolism in ISS because only the microgravity of spaceflight lead to the increase in the size of LD in hepatocytes.

### Educational Program

### Educational Program 1

[EP01] Educational lecture 1

March 17(Tsu), 8:30 - 10:30, Room C

### Educational Program 2

[EP02]
Educational Workshop

March 17(Thu), 16:00 - 18:00, Room J

**[EP01-01]**
**Excitation-Contraction Coupling**

*Junichi Nakai[1] ([1]*Tohoku University Graduate School of Dentistry*)

**[EP01-02]**
**Circadian rhythm research based on basic and applied approach**

*Shigenobu Shibata[1] ([1]*School of Advanced Science and Engineering, Waseda University*)

**[EP01-03]**
**Curse of homeothermy -think about thermoregulation in human beings-**

*Kei Nagashima[1] ([1]*Body temperature and fluid laboratory, Faculty of Human Sciences, Waseda University*)

**[EP02-01]**
***Cancelled***

### Educational Program 3

[EP03]
Model lessons for instructors of physiology

March 18(Fri), 8:30 - 10:30, Room C

### [EP03-01]
**Let's try to clarify all factors for producing edema using physiological logical thinking**

*Toshio Ohhashi[1] ([1]*Shinshu University, School of Medicine, Department of Innovation of Medical and Health Sciences*)

### [EP03-02]
**Fetal Circulation and Changes at Birth**

*Utako Yokoyama[1] ([1]*Department of Physiology, Tokyo Medical University*)

### [EP03-03]
**Heart failure; pathophysiology and treatment**

*Teruyuki Yanagisawa[1] ([1] *Professor Emeritus (Pharmacology), Tohoku University*)

### [EP03-04]
***commentator***

*Yasushi Matsuyama[1] ([1]*Medical Education Center, Jichi Medical University*)

### Educational Program 4

[EP04]
Educational lecture 2

March 18(Fri), 14:15 - 16:15, Room C

### [EP04-01]
**Environmental factors and pain: Effects of changes in temperature and barometric pressure on pain**

*Jun Sato[1] ([1]*Department of Physical Therapy, College of Life and Health Sciences*)

### [EP04-02]
**Why humans become obese ? - Unraveling the myth of nutrient metabolism**

*Tomohiro Tanaka[1] ([1]*Nagoya City University*)

### [EP04-03]
**Understanding neurogenesis required for brain development and function**

*Noriko Osumi[1] ([1]*Tohoku University Graduate School of Medicine*)

### WPI-IIIS Joint Symposium

### WPI-IIIS Joint Symposium Session 1

March 18(Fri), 8:45 - 10:15, Room K

### [WJS1-01]

### Computations in neuron-glia circuits for controlling behavioral states

*Misha Ahrens[1] ([1]*Howard Hughes Medical Institute*)

### [WJS1-02]

### Optical tools for studying the brain

*Adam Cohen[1] ([1]*Harvard University*)

Optical tools for simultaneous perturbation and measurement of membrane potential enable spatially resolved mapping of neural activity with high resolution in space and time, in behaving animals. I will describe some advances in voltage indicators, microscope systems, and analysis software. With these advanced tools, we are studying the dynamics of microcircuits involved in control of attention and the sub-cellular details of dendritic integration. I will also describe some new approaches to storing brain-wide records of neural activity via intracellular protein "ticker tapes".

### [WJS1-03]

### Controlling the Fate and Function of Proteins with Proximity Photopharmacology

*Dirk Trauner[1] ([1]*New York University*)

Photopharmacology endeavors to control biological function with synthetic photoswitches that can be attached covalently or non-covalently to their targets - or nearby. I will discuss potential applications of photopharmacology in biology and medicine, in particular with respect to controlling signal transduction and targeted protein degradation. I will make a case that "Proximity Photopharmacology" is a particularly effective strategy.

### WPI-IIIS Joint Symposium Session 2

March 18(Fri), 10:30 - 12:15, Room K

### [WJS2-01]

### Inner workings of channelrhodopsins and brains

*Karl Deisseroth[1] ([1]*Stanford University*)

### [WJS2-02]

### Mechanical interactions of dendritic-spine synapses

*Haruo Kasai[1] ([1]*The University of Tokyo*)

The majority of excitatory glutamatergic synapses are made on dendritic spines which enlarge during learning. Since dendritic spines and the presynaptic terminals are tightly connected with the synaptic cleft, the enlargement may have mechanical effects on presynaptic functions. We found that the fine and transient pushing of the boutons by a glass pipette markedly promoted an evoked neurotransmitter release and the assembly of soluble *N*-ethylmaleimide-sensitive factor attachment protein receptor (SNARE) proteins, whose Förster resonance transfer (FRET) was measured with fluorescence lifetime imaging (FLIM) in rat slice culture preparations. Surprisingly, both effects persisted over 20 min. The increased presynaptic FRET was independent of cytosolic calcium ($Ca^{2+}$), but dependent on the assembly of SNARE proteins and actin polymerisation in the boutons. Importantly, a low hypertonic sucrose solution (20 mM) caused facilitatory effects on both the FRET and evoked release without inducing spontaneous release, making a striking contrast with a high hypertonic sucrose solution (300 mM) which induced exocytosis by itself. Finally, the spine enlargement, induced by the two-photon glutamate uncaging, enhanced evoked release and FRET only when the spines pushed the boutons by their elongation. Thus, we have found a mechano-sensory and transduction mechanism in the presynaptic boutons.

### [WJS2-03]

### How synaptic plasticity mediates learning and memory in vivo: an optogenetic approach

*Michisuke Yuzaki[1] ([1]*Keio University*)

Long-term potentiation (LTP) and long-term depression (LTD) of excitatory neurotransmission has been proposed as a cellular substrate for learning and memory in vivo. Although LTP and LTD are widespread phenomena expressed at every excitatory synapse in the mammalian brain, it is not completely understood whether and how LTP/LTD at specific synapses are causally linked to learning and memory in vivo. This is mainly because it is unknown whether LTP/LTD are induced in vivo in similar stimulus conditions that are used for the induction of LTP/LTD in acute slice preparations. Further, genetic engineering in mice could induce compensatory mechanisms that could modify synaptic plasticity in the remaining circuits. To circumvent these problems, we developed new optogenetic tools, termed PhotonSABER and LysopH-up, which enabled the temporal, spatial, and cell type-specific inhibition of LTD and LTP, respectively, while the basal synaptic properties and other forms of synaptic plasticity were unaffected. Using these tools at parallel fiber-Purkinje cell synapses in the cerebellum, we will show how LTD/LTP at these synapses are causally linked to the cerebellum-dependent oculomotor learning in vivo.

### WPI-IIIS Joint Symposium Session 3

March 18(Fri), 14:30 - 16:00, Room K

### [WJS3-01]

### Induction of hypometabolic and hypothermic states in mice

*Takeshi Sakurai[1] ([1]*University of Tsukuba*)

We found that chemogenetic/optogenetic excitation of Qrfp-expressing neurons (Q neurons) in a region of the mouse hypothalamus (anterior ventral periventricular nucleus) induces sustained hypothermia and hypometabolism, which we named QIH. The QIH was accompanied by a significant decrease in body temperature and oxygen consumption rate. A battery of behavioral tests was performed on the QIH-experienced and QIH-naïve groups, but no differences were found between the two groups, nor were there any differences in histological observations of the brain, heart, muscles, or other organs. The fact that QIH can be repeated in the same individual suggests that QIH is a reversible and safe hypometabolic state, i.e., a hypometabolic state similar to hibernation. Histological and photogenetic analyses suggested that Q neurons operate mainly through the DMH. QRFP is widely conserved in mammals, suggesting that Q neurons may be a hypometabolism-inducible neural pathway that is widely conserved in mammals. Physiologically, Q neurons may be involved in rapid shifts in body temperature set points and have been shown to be involved in circadian control of body temperature. Animals in hibernation are in a state of hypothermia, hypometabolism, and low activity, but even under these conditions, they can adapt to changes in the environment and spontaneously return to their original state without any tissue damage. If the oxygen demand of animals could be safely lowered as in hibernating animals, various applications are possible, and we would like to discuss the medical application of QIH. We will also discuss the induction of QIH using a new photogenetic method with mutant OPN4.

### [WJS3-02]

### Spying on neuromodulation by constructing a toolbox of genetically encoded fluorescent sensors

*Yulong Li[1] ([1]*Peking University*)

Diverse neuromodulators in the brain, such as acetylcholine, monoamines, lipids and neuropeptides, play important roles in a plethora of physiological processes including reward, movement, attention, sleep, learning and memory. Dysfunction of the neuromodulatory system is associated with a range of diseases, such as epilepsy, addition, neurodegenerative and psychiatric diseases. A longstanding yet largely unmet goal is to measure the dynamics of different neuromodulators reliably and specifically with high spatiotemporal resolution, particularly in behaving animals. To achieve this goal, we develop a series of genetically encoded GPCR-activation-based (GRAB) sensors for the detection of acetylcholine, dopamine, norepinephrine, adenosine, ATP, serotonin, histamine, endocannabinoids and neuropeptides, and validate the performance of these sensors in multiple preparations *in vitro* and *in vivo*. The GRAB sensor toolbox provides new insights into the dynamics and mechanism of neuromodulatory signaling both in health and disease.

### [WJS3-03]

### Neural circuits underlying sleep structure and functions

*Antoine Adamantidis[1] ([1]*University of Bern*)

The activity of multiple brain circuits is strongly modulated during sleep states. Some of these are implicated in the temporal control of the sleep-wake cycle, while others support sleep-dependent functions including memory consolidation. In this lecture, I will summarize our recent work investigating a role for REM sleep in modulating cellular dynamics of neural circuits controlling of goal-oriented behaviours and its implication for the maintenance of innate behaviour.
